# Terminal Cyclopropylsilyl Alcohols as Useful Key Units to Access 2,3,4,6-Tetrasubstituted Tetrahydropyran Scaffolds by Stereocontrolled Prins Cyclization

**DOI:** 10.1021/acs.orglett.4c01806

**Published:** 2024-06-10

**Authors:** Laura F. Peña, Asunción Barbero

**Affiliations:** Department of Organic Chemistry, Campus Miguel Delibes, University of Valladolid, 47011 Valladolid, Spain

## Abstract

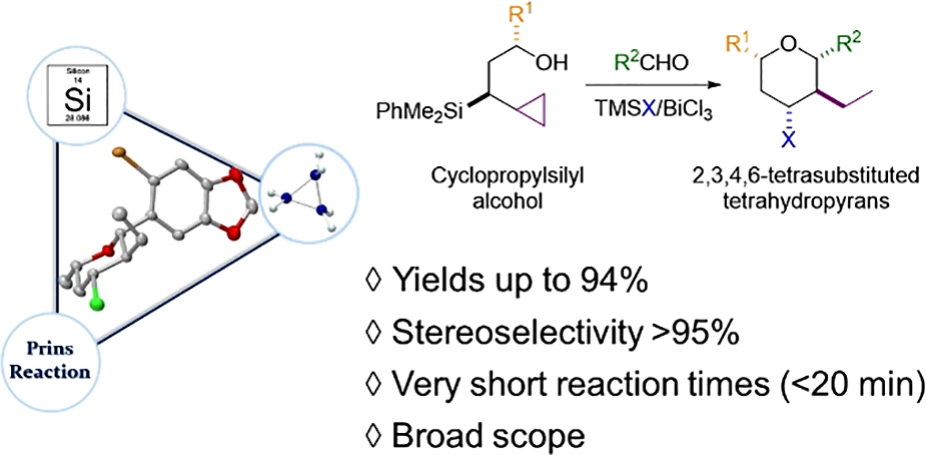

In this study, the use of terminal cyclopropylsilyl alcohols in Prins cyclization is reported as a very efficient methodology for the preparation of polysubstituted tetrahydropyrans, in which three new stereogenic centers have been created in a single pot. The reaction is general for a wide variety of aldehydes (alkylic, vinylic, aromatic, or dialdehydes), different types of alcohols, and halogenation agents providing high yields and excellent diastereoselectivity 2,3,4,6-tetrasubstituted tetrahydropyranyl frameworks. Interestingly, diastereomeric alcohols provide the same tetrahydropyranyl derivatives, showing that the reaction mechanism proceeds through common intermediates.

Saturated six-membered oxacycles are important scaffolds abundantly dispersed in the structure of naturally occurring compounds.^1^ Within the plethora of oxacycles of varying substitution patterns found in nature, the occurrence of 2,3,4,6-tetrasubstituted tetrahydropyranyl frameworks in relevant bioactive natural products is attracting particular interest. Among them, clavosolide A,^2^ isolated from the marine sponge *Myriastra clavosa*, and polycavernoside A^3^ (isolated from red alga, *Gracilaria edulis)* are representative examples which contain a common 2,4,6-*cis*-3-*trans*-tetrahydropyranyl moiety and have a marine origin (Figure 1).

**Figure 1:**
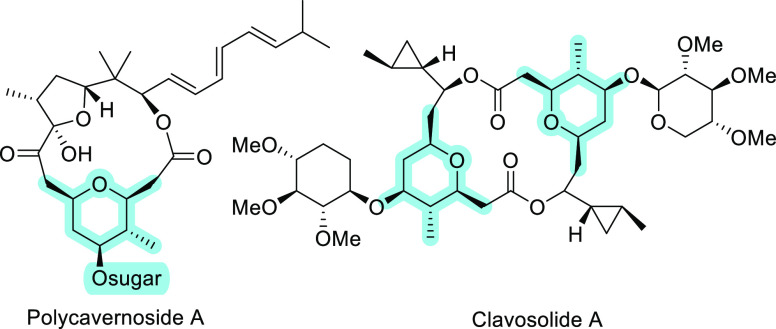
Bioactive natural products containing 2,3,4,6-tetrasubstituted tetrahydropyranyl frameworks.

Among the various methods developed for the synthesis of substituted tetrahydropyrans, the acid-mediated intramolecular condensation of alkenols with aldehydes, known as Prins cyclization, has been demonstrated as a powerful approach.^4,5^ The reaction implies the addition of a π-nucleophilic alkene to an electrophilic oxocarbenium ion, formed in situ, by an *endo* cyclization, which affords the desired oxacycle. In order to broaden the versatility of this atom-economy methodology a variety of nucleophilic and electrophilic components have been tested. Within the π-nucleophilic component, the use of alkenyl silanes, as enhanced nucleophiles, has enabled faster and more selective processes due to the formation of a stabilized β-silyl carbocation intermediate.^6^ Different π-nucleophilic silanes have shown great efficiency for the selective formation of various sized oxa- and azacycles. In this field, we have reported both the use of allylsilyl alcohols for the synthesis of 7- and 8-membered oxacycles^7^ and azacycles,^8^ as well as the use of vinylsilyl alcohols for the preparation of tetrahydropyrans.^9,10^ However, almost no attention has been paid to the possibilities of cyclopropyl moieties as highly strained structures in Prins cyclizations. Within the insignificant number of reports on cyclopropanes used as π-nucleophilic components in Prins cyclizations, Yadav has reported the acid-catalyzed reaction of either 2-silylmethylcyclopropylmethanols^11^ or 2-arylcyclopropylmethanols^12^ with aldehydes to generate the corresponding 2,4,6-substituted tetrahydropyrans. In both cases, the reaction mechanism implies an initial dehydration process to give a stabilized carbocation (either a β-silyl or benzylic carbocation). Further attack by the aldehyde provides an intermediate oxocarbenium ion that readily undergoes Prins cyclization. In another example, Shi^13^ uses 2-(arylmethylene)cyclopropylcarbinol as a convenient aldehyde partner to obtain disubstituted methylenetetrahydropyrans in moderate to high yield and good stereoselectivity. In a similar approach, Cha^14^ has described the stereoselective preparation of 2,4,6-*cis*-trisubstituted tetrahydropyrans from cyclopropanols (Scheme 1). A common drawback of all of these strategies is the very limited number of compounds reported and the exclusive access to 2,4,6-substituted tetrahydropyrans.

**Scheme 1:**
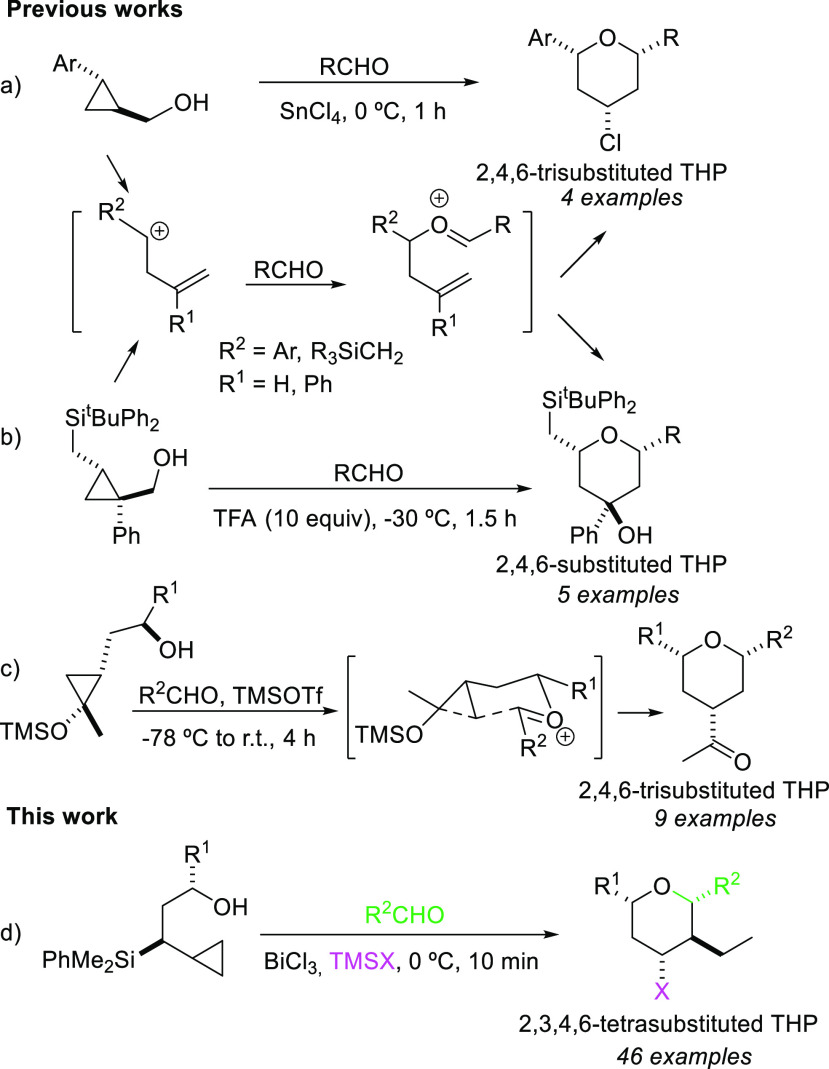
Prins Cyclization with Cyclopropyl Alcohols

Considering the few methodologies reported to date on Prins cyclization with cyclopropanes, their limited scope, and the sole use of cyclopropylmethanols or ethanols as starting materials, we decided to study the outcome of this interesting approach using cyclopropylpropanols in which a silyl group is bonded to the chain.

The substrate chosen for the initial cyclization studies was cyclopropylsilyl alcohol **3a**, which was readily obtained in two steps from phenyldimethylallylsilane using the methodology shown in Scheme 2. Thus, deprotonation of phenyldimethylallylsilane followed by reaction of the corresponding α-silyl carbanion with methyloxirane gave a mixture of the α- and γ-addition products. After separation of the isomers, allylsilyl alcohols **1a** and **1b** were converted (either using Simmons–Smith or Yamamoto cyclopropanation conditions) into cyclopropylsilyl alcohols **3a** and **3b** in good yields.

**Scheme 2:**
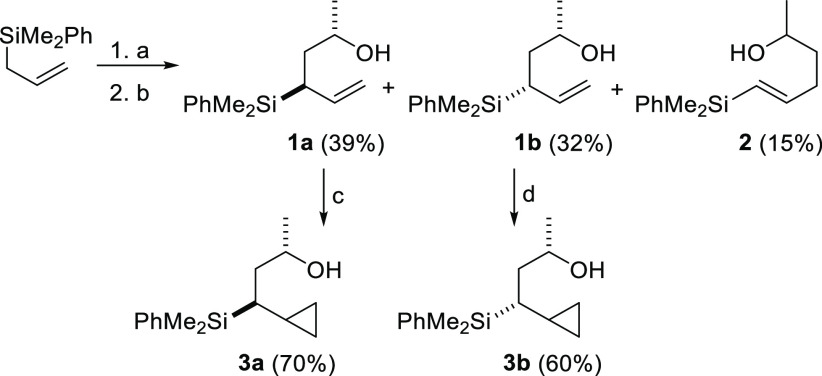
Synthesis of Cyclopropylsilyl Alcohols Reaction conditions: (a) ^n^BuLi (1.2 equiv), TMEDA (1.6 equiv), THF, 0 °C, 2 h; (b) then methyloxirane, −78 °C, 30 min; (c) Et_2_Zn (3.5 equiv), CH_2_I_2_ (3.5 equiv), CH_2_Cl_2_, 0 °C to r.t., 20 h; (d) Me_3_Al (2 equiv), CH_2_I_2_ (2 equiv), CH_2_Cl_2_, 0 °C to r.t., 20 h.

We then chose cyclopropylsilyl alcohol **3a** and (*E*)-cinnamaldehyde, as starting materials, to evaluate Prins cyclization with a variety of Lewis acids in CH_2_Cl_2_ under mild conditions. As shown in Table 1, using TMSOTf or BiCl_3_ as promoters the reaction did not occur or gave complex mixtures (Table 1, entries 1 and 2), while the reaction mediated by AlCl_3_ or TMSCl mainly provided tetrahydrofuran **6**, which is the product of the acid-catalyzed cyclization of **3a** (Table 1, entries 3 and 4). The product of a Prins cyclization was finally obtained under FeCl_3_ catalysis in moderate yield as an 88:12 mixture of epimers. Fortunately, the use of either catalytic BiCl_3_ or FeCl_3_ together with stoichiometric amounts of TMSCl (as the chloride source) produced high yields of the tetrahydropyranyl derivative (Table 1, entries 6 and 7). From both, we chose BiCl_3_/TMSCl for further studies since under these conditions a single diastereoisomer **4f** was obtained (Table 1, entry 7).

**Table 1:**
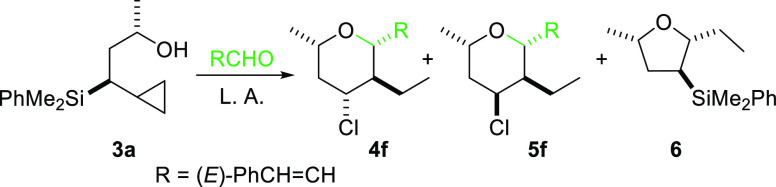
Optimization of the Silyl-Prins Cyclization from Cyclopropylsilyl Alcohols

entry	Lewis acid (equiv)	conditions	**4**+**5:6**^a^	**4**:**5**^a^	product^b^ (yield, %)
1	TMSOTf (1)	–78→0 °C, 4 h			CM^c^
2	BiCl_3_ (0.6)	0 °C, 3 h			n.r.^d^
3	AlCl_3_ (0.6)	0 °C, 2 h	9:91		**6** (45)
4	TMSCl (1.2)	0 °C, 20 h	9:91		**6** (60)
5	FeCl_3_ (0.6)	0 °C, 3 h	100:0	88:12	**4f**+**5f** (58)
6	TMSCl (1.2)/ FeCl_3_ (0.1)	0 °C, 20 min	100:0	94:6	**4f** (75)
7	TMSCl (1.2)/ BiCl_3_ (0.1)	0 °C, 20 min	100:0	>95:5	**4f** (72)

a Determined by NMR analysis of the crude mixture.

b Isolated yield.

c CM stands for complex mixtures.

d n.r. stands for “no reaction”.

We then decided to study the scope and generality of this reaction leading to 2,3,4,6-tetrasubstituted tetrahydropyrans, using various cyclopropyl alcohols and a broad range of aldehydes. The results are shown in Table 2.

**Table 2:**
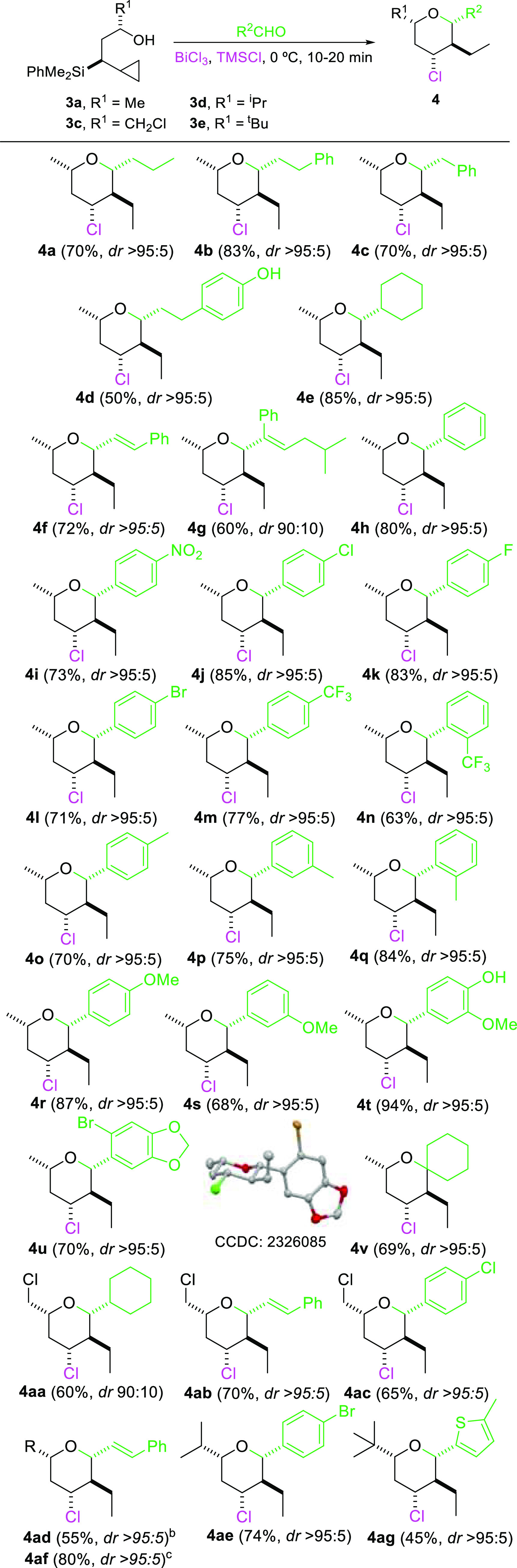
Prins Cyclization from Cyclopropylsilyl Alcohols^a^

a Reaction conditions: compound **3** (0.32 mmol), aldehyde (0.39 mmol), BiCl_3_ (0.032 mmol), TMSCl (0.39 mmol), DCM, 0 °C, 10–30 min. *dr* was determined by ^1^H NMR spectroscopy.

b R = ^i^Pr.

c R = ^t^Bu.

As shown, the use of either saturated, unsaturated, or aromatic aldehydes gave good yields of the corresponding tetrahydropyranyl structures. Substituted aromatic aldehydes with a variation in both the electronic nature and the position of the substituent with respect to the aldehyde were tested. Interestingly *para*-substituted aldehydes with either electron withdrawing or electron donating substituents were shown to be great reaction partners in this process. Moreover, the same type of substituents at the *ortho* or *meta* position were also tolerated with not significant changes in either yield or selectivity (**4o**-**4q**). Noteworthy, the use of alcohol **3c** (derived from epichloridrine) furnishes tetrahydropyrans (**4aa-4ac**) with two derivatizable groups. The reaction is also compatible with the presence of very bulky groups in the neighborhood of the alcohol (**3d**–**e**). Excellent diastereoselectivity toward the formation of a single or very major diasteroisomer was observed in all cases. The stereochemical assignments were performed with the help of ^1^H NMR and NoESY experiments. Definitive confirmation was obtained thanks to the X-ray crystal structure of compound **4u** (Table 2).

To further expand the scope of this efficient methodology, we examined the reaction using an aromatic dialdehyde that could undergo either single or double Prins cyclization. To our delight, under controlled conditions we could obtain, in moderate yields, either the product of double Prins^15^ cyclization or the product of single addition, although in the last case the acid-catalyzed cyclization of **3a** to give **6** was shown to be a competitive reaction (Scheme 3).

**Scheme 3:**
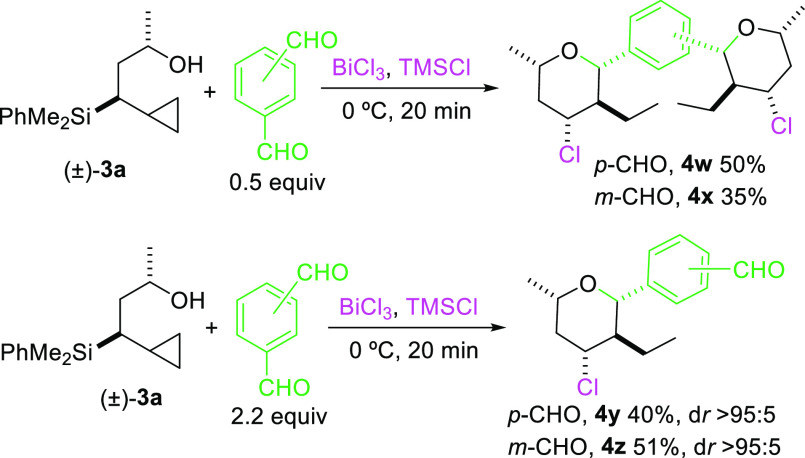
Prins Cyclization with Aromatic Dialdehydes

With these promising results in hand, we next decided to evaluate the effect on the outcome of the process of the relative configuration of the starting alcohol at the silicon bearing carbon. Thus, reaction of cyclopropylsilyl alcohol **3b** with either alkylic, vinylic, or arylic aldehydes resulted in the formation of the same adducts previously obtained with **3a**, which implies that both reaction pathways go through common intermediates (Scheme 4).

**Scheme 4:**
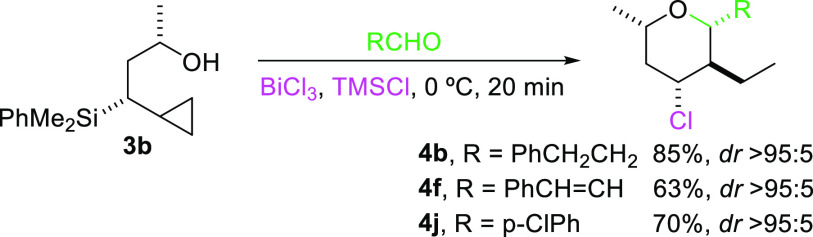
Influence of the Configuration of the Starting Alcohol

To explain these interesting results, we hypothesize that in the presence of small amounts of HCl (a byproduct of the silylation of the alcohol), an initial protodesilylation process will occur. During this process, the acid-catalyzed opening of the cyclopropylsilane will take place in a preferred conformation where the hydrogen at the stereogenic center adjacent to the cyclopropane is oriented toward the ring. The known preference for an antiperiplanar arrangement between the silane and the leaving group in elimination reactions would allow the formation of a β-silyl carbocation stabilized by hyperconjugation between the C–Si bond and the parallel p empty orbital. The final loss of the silyl group would then produce the *trans* alkene **I**. Presumably, the configuration of the distant carbinol will not interfere with this arrangement. The formation of a single (*E*)-alkene in this step is interesting, since previous findings by Wilson et al. have shown that solvolysis of [(trimethylsilyl)alkyl]cyclopropanes occurs with a moderate control of the olefin geometry.^16^ Further Prins cyclization of intermediate **I**, in the presence of the Lewis acid, would initially provide an (*E*)-oxocarbenium ion **II** and then the tetrahydropyranyl cation **III**, through a chairlike transition state in which all the substituents will be equatorial. The final equatorial trapping of the secondary tetrahydropyranyl cation will afford the shown compound (Scheme 5).

**Scheme 5:**
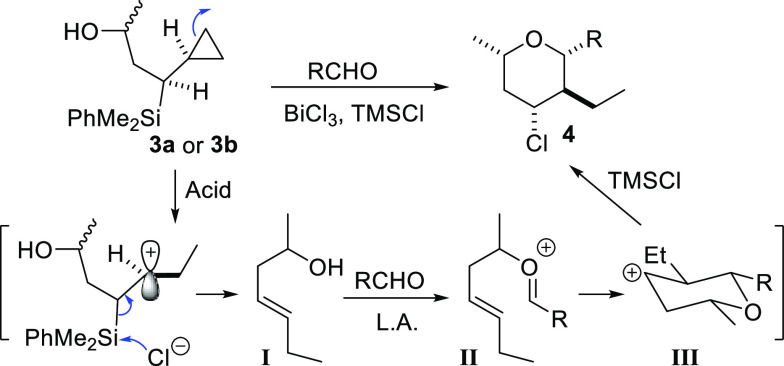
Mechanistic Proposal

The formation of intermediate **I** during the course of this cyclization was unambiguously confirmed by treating compound **3a** with BiCl_3_ and TMSCl for 5 min. Under these conditions the reaction mixture yielded a single compound, identified as (*E*)-hept-4-en-2-ol **I**.^17^ Moreover, when **I** was submitted to reaction with butyraldehyde (in the presence of BiCl_3_ and TMSCl) tetrahydropyran **4a** was obtained as a single product (65%, *dr* > 95:5)

It is important to highlight that within the limited number of examples detailing the synthesis of 2,3,4,6-tetrasubstituted tetrahydropyrans through Prins cyclization of homoallylic alcohols (with an internal double bond similar to **I**),^18^ various challenges have been reported.^19,20^ Notably, none of these secondary reactions were observed in our cyclization, resulting in a single 2,3,4,6-tetrasubstituted tetrahydropyran with excellent chemo- and stereoselectivity and good yield.

We next studied the full potential of this methodology using a tertiary alcohol **3f**, which are not commonly used in Prins cyclization due to their reduced nucleophilicity and higher ease of dehydration (Scheme 6).

**Scheme 6:**
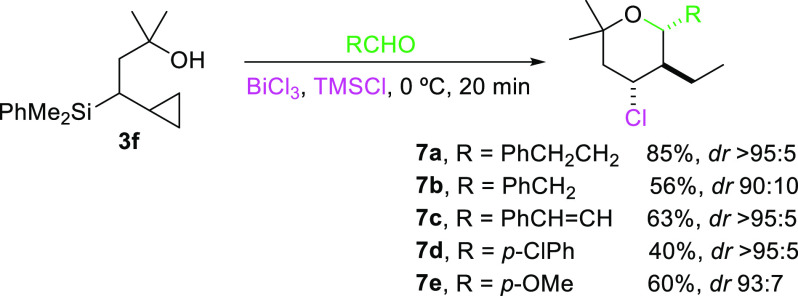
Prins Cyclization with Tertiary Alcohol 3c

Interestingly, the reaction is effective with either alkylic, vinylic, or aromatic aldehydes (both electron-rich and electron-poor) giving moderate to good yields of the corresponding tetrahydropyrans **7**. Moreover, excellent diasteroselectivity is observed toward the shown isomer.

Finally, to expand the chemical tool box of this process, we explored the introduction of different halogens at C4. Interestingly, the use of the appropriate reagent enabled the introduction of different halides, such as bromide or iodide, at C4. Thus, treatment of **3a** with aldehydes in the presence of catalytic BiCl_3_ and stoichiometric TMSBr provided the 4-bromo-tetrahydropyranyl derivatives **8a**–**g** with high efficiency. Moreover, a reaction on larger scale (300 mg, 1.21 mmol of **3a**) using TMSBr and cinnaldehyde was conducted, providing compound **8b** in good yield (235 mg, 63%) and stereocontrol (>95:5). On the other hand, the best reagent for the preparation of 4-iodo derivatives was shown to be TMSI in combination with catalytic BiCl_3_. The results are shown in Table 3. For both halides, the cyclization is again general for various types of aldehydes (alkylic, vinylic, or arylic) and proceeds in high yield and excellent stereoselectivity toward a single 2,4,6-*cis*-3-*trans*-tetrasusbstituted tetrahydropyran. Interestingly, the formation of only one C4 halogenated tetrahydropyran, in which the halogen atom is always transferred from the TMSX reagent, seems to indicate that BiCl_3_ acts as the Lewis acid and TMSX acts as the external halogen source.

**Table 3:**
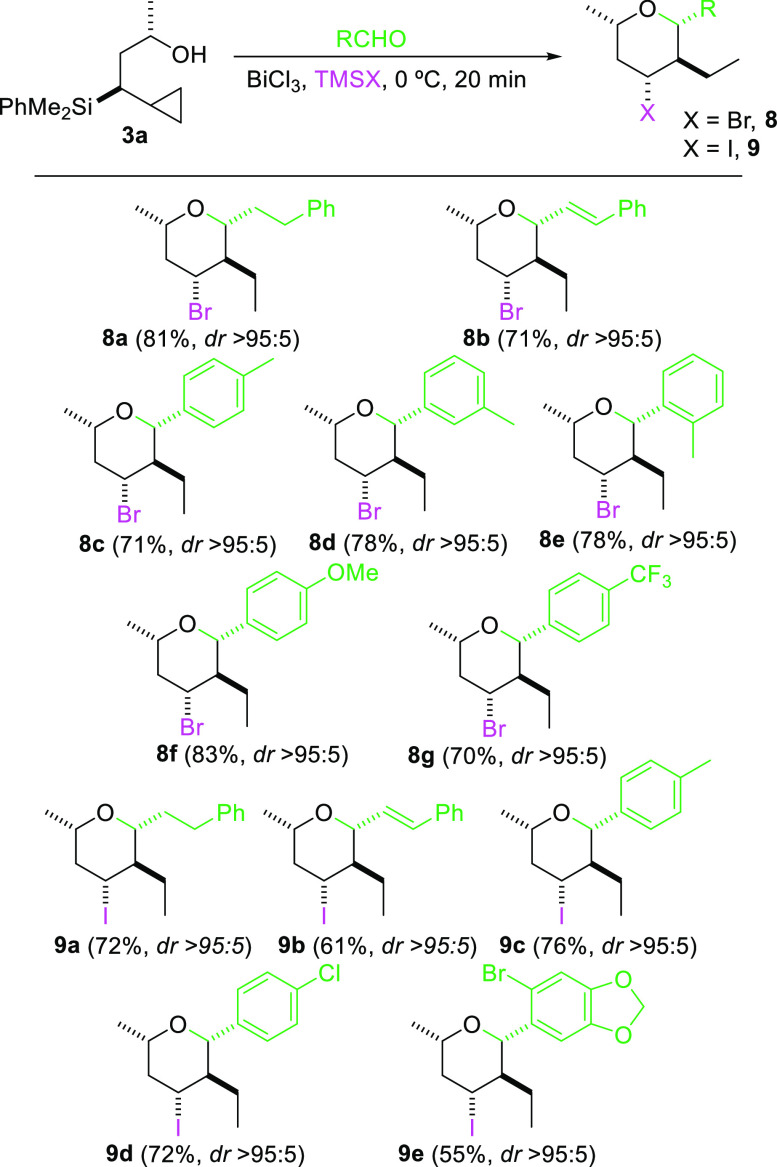
Prins Cyclization from Cyclopropylsilyl Alcohols^a^^,^^b^

a Reaction conditions: compound **3a** (0.32 mmol), aldehyde (0.39 mmol), BiCl_3_ (0.032 mmol), TMSX (0.39 mmol), DCM, 0 °C, 20 min.

b *dr* was determined by ^1^H NMR spectroscopy.

In conclusion, the use of terminal cyclopropylsilyl alcohols in Prins cyclization has been shown as a very effective methodology for the synthesis of 2,4,6-*cis*-3-*trans*-tetrasubstituted tetrahydropyrans. The reaction proceeds in short time, high yield, and excellent chemo- and stereoselectivity to provide a single tetrahydropyran for a wide range of alcohols, aldehydes, and halogenated Lewis acids. Interestingly the cyclization of diastereomeric alcohols provides a common tetrahydropyranyl derivative, showing that the reaction mechanism goes through common intermediates.

## Data Availability

The data underlying this study are available in the published article and its Supporting Information.

## Supporting Information Available

Detailed experimental procedures, spectroscopic characterization, ^1^H and ^13^C NMR spectra for new starting materials and products, and X-ray crystal structure data for compound **4u** (PDF)

## References

[ref1] Martin S. F. Natural Products and Their Mimics as Targets of Opportunity for Discovery. J. Org. Chem. 2017, 82, 10757–10794. 10.1021/acs.joc.7b01368. 28738152 PMC5653958

[ref2] aErickson K. L.; Gustafson K. R.; Pannell L. K.; Beutler J. A.; Boyd M. R. New Dimeric Macrolide Glycosides from the Marine Sponge *Myriastra clavos*a. J. Nat. Prod. 2002, 65, 1303–1306. 10.1021/np020193z. 12350152

[ref3] Yotsu-Yamashita M.; Haddock R. L.; Yasumoto T. Polycavernoside A: A Novel Glycosidic Macrolide from the Red Alga Polycaremosatsudai (Gracilariaedulis). J. Am. Chem. Soc. 1993, 115, 1147–1148. 10.1021/ja00056a048.

[ref4] aOlier C.; Kaafarani M.; Gastaldi S.; Bertrand M. P. Synthesis of tetrahydropyrans and related heterocycles via prins cyclization; extension to aza-prins cyclization. Tetrahedron 2010, 66, 413–445. 10.1016/j.tet.2009.10.069.

[ref5] Budakoti A.; Mondal P. K.; Verma P.; Khamrai J. Prins cyclization-mediated stereoselective synthesis of tetrahydropyrans and dihydropyrans: an inspection of twenty years. Beilstein J. Org. Chem. 2021, 17, 932–963. 10.3762/bjoc.17.77. 33981366 PMC8093554

[ref6] Díez-Poza C.; Barbero A. Synthesis of O- and N-Heterocycles by Silyl-Prins Cyclization of Allylsilanes. Eur. J. Org. Chem. 2017, 2017, 4651–4665. 10.1002/ejoc.201700644.

[ref7] aDiez-Varga A.; Barbero H.; Pulido F. J.; González-Ortega A.; Barbero A. Competitive Silyl–Prins Cyclization versus Tandem Sakurai–Prins Cyclization: An Interesting Substitution Effect. Chem.-Eur. J. 2014, 20, 14112–14119. 10.1002/chem.201403421. 25196494

[ref8] Barbero A.; Diez-Varga A.; Pulido F. J.; González-Ortega A. Synthesis of Azepane Derivatives by Silyl-aza-Prins Cyclization of Allylsilyl Amines: Influence of the Catalyst in the Outcome of the Reaction. Org. Lett. 2016, 18, 1972–1975. 10.1021/acs.orglett.6b00538. 27074135

[ref9] Díez-Poza C.; Barbero A. Unexpected Domino Silyl-Prins/Aryl Migration Process from Geminal Vinylsilyl Alcohols. Org. Lett. 2021, 23, 8385–8389. 10.1021/acs.orglett.1c03121. 34615353 PMC8576834

[ref10] Díez-Poza C.; Fernández-Peña L.; González-Andrés P.; Barbero A. Changing the Reaction Pathway of Silyl-Prins Cyclization by Switching the Lewis Acid: Application to the Synthesis of an Antinociceptive Compound. J. Org. Chem. 2023, 88, 6776–6783. 10.1021/acs.joc.3c00050. 37220201 PMC10242753

[ref11] Yadav V. K.; Vijaya Kumar N. Highly Stereoselective Prins Cyclization of Silylmethyl-Substituted Cyclopropyl Carbinols to 2,4,6-Trisubstituted Tetrahydropyrans. J. Am. Chem. Soc. 2004, 126, 8652–8653. 10.1021/ja048000c. 15250708

[ref12] Yadav V. K.; Verma A. K.; Kumar P.; Hulikal V. 2-Arylcyclopropylmethanol as a substitute for homoallyl aryl alcohol in the construction of cis-2,6-disubstituted tetrahydropyran: synthesis of (±)-centrolobine. Chem. Commun. 2014, 50, 15457–15460. 10.1039/C4CC07796B. 25354489

[ref13] Tian G.-Q.; Shi M. Brønsted Acid-Mediated Stereoselective Cascade Construction of Functionalized Tetrahydropyrans from 2-(Arylmethylene)cyclopropylcarbinols and Aldehydes. Org. Lett. 2007, 9, 2405–2408. 10.1021/ol0709026. 17503842

[ref14] Lee H. G.; Lysenko I.; Cha J. K. Stereoselective Synthesis of 2,4,6-Trisubstituted Tetrahydropyrans by the Use of Cyclopropanols as Homoenols. Angew. Chem., Int. Ed. 2007, 46, 3326–3328. 10.1002/anie.200700172. 17385770

[ref15] A mixture of the two possible stereoisomers of bis-tetrahydropyrans **4w** and **4x** (e.g., both 1-(2*R**,*3S**,4*S**,*6R**)-tetrahydro-2*H*-pyran-2-yl)-4-(2*S**,3*R**,4*R**,6*S**)-tetrahydro-2*H*-pyran-2-yl)benzene and 1-(2*R**,*3S**,4*S**,*6R**)-tetrahydro-2*H*-pyran-2-yl)-4-(2*R**,3*S**,4*S**,6*R**)-tetrahydro-2*H*-pyran-2-yl)benzene for **4w**) would be expected when racemic **3a** reacts with 0.5 equiv of either terephthaldehyde or isophthaldehyde. The observation of a single NMR distinguishable diastereoisomer in the reaction mixture could be explained by the high symmetry and the remote position of the stereocenters on these molecules.

[ref16] Wilson S. R.; Zucker P. A. Silicon-mediated skipped diene synthesis. Application to the melon fly pheromone. J. Org. Chem. 1988, 53, 4682–4693. 10.1021/jo00255a007.

[ref17] Naef R.; Velluz A.; Jaquier A. New Volatile Sulfur-Containing Constituents in a Simultaneous Distillation-Extraction Extract of Red Bell Peppers (Capsicum annuum). J. Agric. Food Chem. 2008, 56, 517–527. 10.1021/jf072493y. 18163560

[ref18] Kataoka K.; Ode Y.; Matsumoto M.; Nokami J. Convenient synthesis of highly optically active 2,3,4,6-tetrasubstituted tetrahydropyrans via Prins cyclization reaction (PCR) of optically active homoallylic alcohols with aldehydes. Tetrahedron 2006, 62, 2471–2483. 10.1016/j.tet.2005.12.054.

[ref19] Tadpetch K.; Rychnovsky S. D. Rhenium(VII) Catalysis of Prins Cyclization Reactions. Org. Lett. 2008, 10, 4839–4842. 10.1021/ol8019204. 18816133 PMC2665917

[ref20] Biermann U.; Lützen A.; Metzger J. O. Synthesis of Enantiomerically Pure 2,3,4,6-Tetrasubstituted Tetrahydropyrans by Prins-Type Cyclization of Methyl Ricinoleate and Aldehydes. Eur. J. Org. Chem. 2006, 2006, 2631–2637. 10.1002/ejoc.200500701.

