# Supplementary material for: Terminal Cyclopropylsilyl Alcohols as Useful Key Units to Access 2,3,4,6-Tetrasubstituted Tetrahydropyran Scaffolds by Stereocontrolled Prins Cyclization
Source: Org Lett. 2024 Jun 10;26(24):5202–7. doi: 10.1021/acs.orglett.4c01806 (PMC11197095; doi:10.1021/acs.orglett.4c01806)
Supplement: Supplementary file 1 — ol4c01806_si_001.pdf [file ol4c01806_si_001.pdf]

Supporting Information

Terminal cyclopropylsilyl alcohols as useful key units to access 2,3,4,6-tetrasubstituted tetrahydropyran scaffolds by stereocontrolled Prins cyclization

Laura F. Peña and Asunción Barbero*

Department of Organic Chemistry, Campus Miguel Delibes, University of Valladolid, 47011 Valladolid, Spain

email: asuncion.barbero@uva.es

List of Contents

1. **General Procedures** **S1**

2. **Experimental Section** **S2**

   2.1. Synthesis of cyclopropylsilyl alcohols **3a-f** **S2**

   2.2. TMSCl/$BiCl_3$-promoted cyclization of cyclopropylsilyl alcohols **3a-e** **S5**

   2.3. TMSCl/$BiCl_3$-promoted cyclization of cyclopropylsilyl alcohols **3f** **S16**

   2.4. TMSBr/$BiCl_3$-promoted cyclization of cyclopropylsilyl alcohols **3a** **S18**

   2.5. TMSI/$BiCl_3$-promoted cyclization of cyclopropylsilyl alcohols **3a** **S21**

3. **X-Ray Crystallographic Data for compound 4u** **S23**

4. **Copies of NMR Spectra** **S25**

5. **References** **S137**

1. GENERAL PROCEDURES

Unless otherwise noted, experiments were carried out with dry solvents under nitrogen atmosphere. Dichloromethane was dried with preactivated molecular sieves. Flash column chromatography was performed using Silica Gel 60 (230-400 mesh ASTM). Thin layer chromatography (TLC) was performed using aluminium backed plate, pre-coated with silica gel (0.20 mm, silica gel 60) with a fluorescent indicator (254 nm) from Macherey. NMR spectra were recorded at nuclear magnetic resonance service of the Laboratory of Instrumental Techniques (L.T.I., www.laboratoriotecnicasinstrumentales.es) University of Valladolid at Varian 400 MHz (^{1}H, 399.85 MHz; ^{13}C, 100.6 MHz), Varian 500 MHz (^{1}H, 500.12 MHz; ^{13}C, 126 MHz) spectrometers at room temperature (25 °C). Chemical shifts (δ) were reported in parts per million (ppm) relative to the residual solvent peaks recorded, rounded to the nearest 0.01 for ^{1}H-NMR and 0.1 for ^{13}C-NMR (reference: $CDCl_3$ [^{1}H: 7.26, ^{13}C: 77.2]). Spin-spin coupling constants (*J*) in ^{1}H-NMR were given in Hz to the nearest 0.1 Hz, and peak multiplicity was indicated as follows s (singlet), d (doublet), t (triplet), q (quartet), m (multiplet) and br (broad). ^{13}C NMR were recorded with complete proton decoupling. Carbon types, structure assignments and attribution of peaks were determined from two-dimensional correlation experiments (HSQC, COSY and HMBC). Relative stereochemistry was assigned based on the 2D-NOE experiments. High-resolution mass spectra (HRMS) were measured at mass spectrometry service of the Laboratory of Instrumental Techniques, University of Valladolid, using a quadrupole spectrometer equipped with a TOF analyzer, on a UPLC-MS system (UPLC: Waters ACQUITY H-class UPLC; MS: Bruker Maxis Impact) by positive electrospray ionization (ESI+) and at mass spectrometry service of the University of Burgos, on a 6545 Q-TOF Agilent LC-MS mass spectrometer (positive electrospray ionization mode, ESI (+)). X-ray diffraction studies were performed at University of Burgos on a Bruker D8 VENTURE diffractometer.

2. EXPERIMENTAL SECTION

2.1. Synthesis of cyclopropylsilyl alcohols **3a-f**

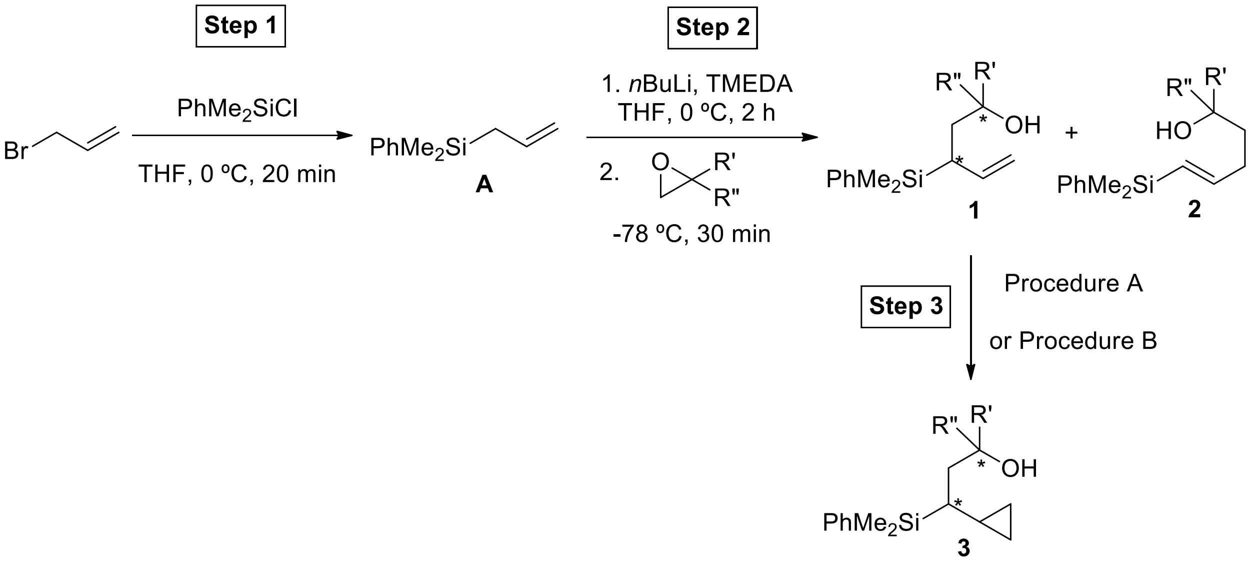

Step 1. 3-bromoprop-1-ene (7.8 mL, 90 mmol, 1.5 equiv) and of chloro(dimethyl)phenylsilane (10.1 mL, 60 mmol, 1.0 equiv) were added dropwise to a suspension of Zn (5.9 g, 90 mmol, 1.5 equiv) in 30 mL of dry THF (3 M) at 0 ºC under nitrogen atmosphere. The mixture was stirred at 0 ºC for twenty minutes and then the reaction was quenched with 30 mL of NH_4Cl sat. The phases were separated and the aqueous phase was extracted three times with diethyl ether (3 x 25 mL). The organic phases were combined, washed with NaCl sat. (60 mL) and dried over anhydrous Na_2SO_4. The solvent was then evaporated under reduced pressure. The crude mixture was analysed by NMR and then purified by column chromatography in silica gel, using hexane to afford allyldimethyl(phenyl)silane **A** as a colourless oil in 92% yield.

Step 2. *n*-BuLi (21.3 mL of a 1.6 M solution in hexane, 34.1 mmol, 1.2 equiv) and TMEDA (7.0 mL, 46.9 mmol, 1.65 equiv) were added dropwise to solution of allyldimethyl(phenyl)silane **A** (5.0 g, 28.4 mmol, 1.0 equiv) in 30 mL of dry THF (1.05 M) at 0 ºC under nitrogen atmosphere. The mixture was stirred at 0 ºC for two hours and then cooled to -78 ºC before adding the corresponding epoxide (28.4 mmol, 1.0 equiv). When starting material was consumed (around 30 minutes after the addition of the epoxide), the reaction was quenched with 25 mL of NH_4Cl sat. The phases were separated and the aqueous phase was extracted three times with diethyl ether (3 x 20 mL). The organic phases were combined, washed with NaCl sat. (50 mL) and dried over anhydrous Na_2SO_4. The solvent was then evaporated under reduced pressure. The crude mixture was analysed by NMR and then purified by column chromatography in silica gel, using a mixture of hexane-ethyl acetate (4:1) and yielding alcohols **1** and **2**.

Step 3.

Procedure A. Furuwaka conditions. Diethylzinc (23.4 mL of a solution 0.9 M, 21 mmol, 3.5 equiv) was added dropwise to a solution of CH_2I_2 (1.7 mL, 21 mmol, 3.5 equiv) in 10 mL of dry CH_2Cl_2 (2.1 M) at 0 ºC under nitrogen atmosphere. After 10 minutes, the corresponding allylsilyl alcohol **1** (6 mmol, 1.0 equiv) was dissolved in 6 mL of dry CH_2Cl_2 (1 M) under nitrogen atmosphere and added into the reaction. The resulting mixture was stirred for twenty hours while the temperature was gradually rising to r.t. The reaction was quenched at 0 ºC with 15 mL of HCl 1 M. The phases were separated and the aqueous phase was extracted three times with dichloromethane (3 x 15 mL). The organic phases were combined, washed with NaCl sat. (30 mL) and dried over anhydrous Na_2SO_4. The solvent was then evaporated under reduced pressure. The crude mixture is analysed by NMR and then purified by column chromatography in silica gel, using a mixture of hexane-ethyl acetate to afford cyclopropylsilyl alcohols **3**.

Procedure B. Yamamoto conditions. Trimethylaluminum (10.0 mL of a 1.0 M solution, 10 mmol, 2.0 equiv) were added dropwise to solution of CH_2I_2 (0.81 mL, 10.0 mmol, 2.0 equiv) in 8 mL of dry CH_2Cl_2 (1.25 M) at -60 ºC under nitrogen atmosphere. After 10 minutes, the corresponding allylsilyl alcohol **1b** (5 mmol, 1.0 equiv) was dissolved in 5 mL of dry

CH_2Cl_2 (1 M) under nitrogen atmosphere and added into the reaction. The resulting mixture was stirred for twenty hours while the temperature was gradually rising to r.t. Then, the reaction was quenched at 0 ºC with $NaHCO_3$ (sat). The phases were separated and the aqueous phase was extracted three times with dichloromethane (3 x 12 mL). The organic phases were combined, washed with NaCl sat. (25 mL) and dried over anhydrous Na_2SO_4. The solvent was then evaporated under reduced pressure. The crude mixture is analysed by NMR and then purified by column chromatography in silica gel, using a mixture of hexane-ethyl acetate to afford cyclopropylsilyl alcohol **3b**.

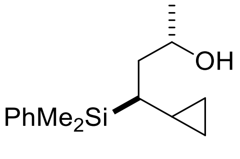

(2*S**,4*S**)-4-cyclopropyl-4-(dimethyl(phenyl)silyl)butan-2-ol (**3a**) was obtained following the procedure A for the cyclopropanation reaction, from the corresponding allylsilane in 20 hours, to give, after column chromatography (hexane/EtOAc: 5:1), a yellow oil in 70% chemical yield (1.0 g from 6 mmol of the corresponding allylsilyl alcohol). **^{1}H NMR (500 MHz, $CDCl_3$)** δ (ppm) 7.55 – 7.50 (m, 2H, Ar-*H*), 7.38 – 7.32 (m, 3H, Ar-*H*), 4.18 – 4.06 (m, 1H, *H*C-OH), 1.53 – 1.40 (m, 2H, CH_2), 1.13 (d, *J* = 6.3 Hz, 3H, CH_3), 0.53 – 0.46 (m, 1H, C*H*H), 0.46 – 0.39 (m, 3H), 0.34 (s, 3H, Si-CH_3), 0.33 (s, 3H, Si-CH_3), 0.18 – 0.12 (m, 1H), 0.01 – (-)0.05 (m, 1H). **^{13}C NMR (101 MHz, $CDCl_3$)** δ (ppm) 138.8 (C), 134.1 (CH), 129.0 (CH), 127.8 (CH), 65.8 (HC-OH), 41.0 (CH_2), 26.9 (CH), 24.5 (CH_3), 12.2 (CH), 6.3(CH_2), 4.5 (CH_2), -3.4 (Si-CH_3), -4.1 (Si-CH_3). **HRMS (ESI+)** m/z calc. for $C_{15}H_{24}NaOSi$ ($[M+Na]^+$): 271.1489, found 271.1492.

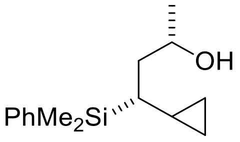

(2*R**,4*S**)-4-cyclopropyl-4-(dimethyl(phenyl)silyl)butan-2-ol (**3b**) was obtained following the procedure B for the cyclopropanation reaction, from the corresponding allylsilane in 20 hours, to give, after column chromatography (hexane/EtOAc: 5:1), a yellow oil in 60% chemical yield (745 mg from 5 mmol of the corresponding allylsilyl alcohol). **^{1}H NMR (500 MHz, $CDCl_3$)** δ (ppm) 7.56 – 7.50 (m, 2H, Ar-*H*), 7.37 – 7.33 (m, 3H, Ar-*H*), 3.97 – 3.88 (m, 1H, *H*C-OH), 1.59 – 1.53 (m, 2H, CH_2), 1.08 (d, *J* = 6.2 Hz, 3H, CH_3), 0.62 – 0.54 (m, 1H), 0.50 – 0.45 (m, 2H), 0.36 (s, 3H, Si-CH_3), 0.35 (s, 3H, Si-CH_3), 0.23 (ddd, *J* = 10.7, 8.6, 5.8 Hz, 1H, Si-CH), 0.06 – 0.02 (m, 2H). **^{13}C NMR (101 MHz, $CDCl_3$)** δ (ppm) 138.8 (C), 134.0 (CH), 129.0 (CH), 127.8 (CH), 67.9 (HC-OH), 41.4 (CH_2), 28.8 (CH), 23.3 (CH_3), 13.0 (CH), 6.1 (CH_2), 5.7 (CH_2), -3.2 (Si-CH_3), -4.2 (Si-CH_3). **HRMS (ESI+)** m/z calc. for $C_{15}H_{24}NaOSi$ ($[M+Na]^+$): 271.1489, found 271.1493.

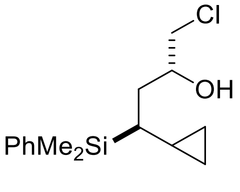

(2*R**,4*S**)-1-chloro-4-cyclopropyl-4-(dimethyl(phenyl)silyl)butan-2-ol (**3c**) was obtained following the procedure A for the cyclopropanation reaction, from the corresponding allylsilane in 20 hours, to give, after column chromatography (hexane/EtOAc: 8:1), a yellow oil in 75% chemical yield (440 mg from 2 mmol of the corresponding allylsilyl alcohol). **^{1}H NMR (400 MHz, $CDCl_3$)** δ (ppm) 7.54 – 7.50 (m, 2H, Ar-*H*), 7.37 – 7.32 (m, 3H, Ar-*H*), 4.22 – 3.12 (m, 1H, *H*C-OH), 3.56 (dd, *J* = 11.0, 3.4 Hz, 1H, C*H*H-Cl), 3.39 (dd, *J* = 11.0, 7.3 Hz, 1H, CH*H*-Cl), 1.96 (d, *J* = 4.7 Hz, 1H, OH), 1.60 (ddd, *J* = 14.2, 9.9, 3.4 Hz, 1H, C*H*H), 1.45 (ddd, *J* = 14.2, 11.0, 3.0 Hz, 1H, CH*H*), 0.58 – 0.55 (m, 2H), 0.48 – 0.38 (m, 2H), 0.36 (s, 3H, Si-CH_3), 0.35 (s, 3H, Si-CH_3), 0.26 – 0.16 (m, 1H), 0.05 – -0.04 (m, 1H). **^{13}C NMR (101 MHz, $CDCl_3$)** δ (ppm) 138.4 (C), 134.1 (CH), 129.1 (CH), 127.8 (CH), 69.3 (HC-OH), 51.5 (CH_2-

Cl), 35.9 (CH_2), 26.5 (CH), 11.9 (CH), 6.2 (CH_2), 4.3 (CH_2), -3.4 (Si-CH_3), -4.2 (Si-CH_3). **HRMS (ESI+)** m/z calc. for $C_{15}H_{23}ClNaOSi$ ($[M+Na]^+$): 305.1099, found 305.1095.

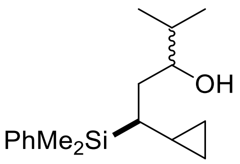

(1*S**)-1-cyclopropyl-1-(dimethyl(phenyl)silyl)-4-methylpentan-3-ol (**3d**) was obtained as a mixture of diastereomers following the procedure A for the cyclopropanation reaction, from the corresponding allylsilanes in 20 hours, to give, after column chromatography (hexane/EtOAc: 8:1), a yellow oil in 55% chemical yield (304 mg from 2 mmol of the corresponding allylsilyl alcohols). **^{1}H NMR (400 MHz, $CDCl_3$)** δ (ppm) 7.56 – 7.50 (m, 2H, Ar-*H*), 7.38 – 7.31 (m, 3H, Ar-*H*), 3.68 (dt, *J* = 7.9, 5.1 Hz, 1H, *H*C-OH), 1.60 – 1.51 (m, 1H, CH), 1.50 – 1.40 (m, 2H, CH_2), 0.88 (d, *J* = 6.8 Hz, 3H, CH_3), 0.85 (d, *J* = 6.8 Hz, 3H, CH_3), 0.53 – 0.39 (m, 4H), 0.35 (s, 3H, Si-CH_3), 0.34 (s, 3H, Si-CH_3), 0.20 – 0.14 (m, 1H), 0.01 – -0.05 (m, 1H). **^{13}C NMR (101 MHz, $CDCl_3$)** δ (ppm) 138.9 (C), 134.1 (CH), 128.9 (CH), 127.8 (CH), 74.3 (HC-OH), 35.7 (CH_2), 34.3 (CH), 26.5 (CH), 19.1 (CH_3), 17.5 (CH_3), 12.2 (CH), 6.2 (CH_2), 4.4 (CH_2), -3.3 (Si-CH_3), -4.1 (Si-CH_3). **HRMS (ESI+)** m/z calc. for $C_{17}H_{28}NaOSi$ ($[M+Na]^+$): 299.1802, found 299.1799.

Distinctive signals of minor isomer: **^{1}H NMR (500 MHz, $CDCl_3$)** δ (ppm) 3.39 (dt, *J* = 8.4, 4.2 Hz, 1H, *H*C-OH), 1.67 (ddd, *J* = 14.3, 6.4, 4.2 Hz, 1H, CH), 0.84 (d, *J* = 6.8 Hz, 3H, CH_3), 0.75 (d, *J* = 6.8 Hz, 3H, CH_3), 0.37 (s, 3H, Si-CH_3), 0.36 (s, 3H, Si-CH_3). **^{13}C NMR (101 MHz, $CDCl_3$)** δ (ppm) 139.0 (C), 134.0 (CH), 129.0 (CH), 127.8 (CH), 76.1 (HC-OH), 36.2 (CH_2), 33.0 (CH), 28.9 (CH), 19.2 (CH_3), 16.4 (CH_3), 13.5 (CH), 6.3 (CH_2), 5.9 (CH_2), -2.9 (Si-CH_3), -4.3 (Si-CH_3).

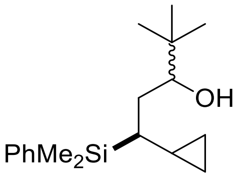

(1*S**)-1-cyclopropyl-1-(dimethyl(phenyl)silyl)-4,4-dimethylpentan-3-ol (**3e**) as a mixture of diastereomers following the procedure A for the cyclopropanation reaction, from the corresponding allylsilanes in 20 hours, to give, after column chromatography (hexane/EtOAc: 8:1), a yellow oil in 52% chemical yield (302 mg from 2 mmol of the corresponding allylsilyl alcohols). **^{1}H NMR (400 MHz, $CDCl_3$)** δ (ppm) 7.56 – 7.52 (m, 2H, Ar-*H*), 7.38 – 7.33 (m, 3H, Ar-*H*), 3.60 (dd, *J* = 10.5, 4.2 Hz, 1H, *H*C-OH), 1.56 – 1.58 (m, 1H, C*H*H), 1.44 – 1.37 (m, 1H, CH*H*), 0.85 (s, 9H, CH_3), 0.53 – 0.42 (m, 4H), 0.35 (s, 6H, Si-CH_3), 0.22 – 0.17 (m, 1H), 0.00 – -0.07 (m, 1H). **^{13}C NMR (101 MHz, $CDCl_3$)** δ (ppm) 139.0 (C), 134.1 (CH), 128.9 (CH), 127.8 (CH), 77.3 (HC-OH), 35.0 (C), 32.7 (CH_2), 26.6 (CH), 25.9 (CH_3), 12.2 (CH), 6.2 (CH_2), 4.0 (CH_2), -3.4 (Si-CH_3), -4.0 (Si-CH_3). **HRMS (ESI+)** m/z calc. for $C_{18}H_{30}NaOSi$ ($[M+Na]^+$): 313.1958, found 313.1955.

Distinctive signals of minor isomer: **^{1}H NMR (500 MHz, $CDCl_3$)** δ (ppm) 3.04 (dd, *J* = 10.0, 4.1 Hz, 1H, *H*C-OH), 1.80 – 1.73 (m, 1H, C*H*H), 1.37 – 1.31 (m, 1H, CH*H*), 0.78 (s, 9H, CH_3), 0.38 (s, 3H, Si-CH_3), 0.37 (s, 3H, Si-CH_3). **^{13}C NMR (101 MHz, $CDCl_3$)** δ (ppm) 139.3 (C), 134.0 (CH), 129.0 (CH), 127.8 (CH), 79.7 (HC-OH), 35.2 (C), 33.7 (CH_2), 29.6 (CH), 25.8 (CH_3), 14.1 (CH), 7.0 (CH_2), 5.6 (CH_2), -2.6 (Si-CH_3), -4.2 (Si-CH_3).

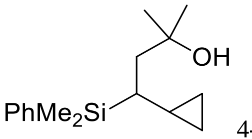

4-cyclopropyl-4-(dimethyl(phenyl)silyl)-2-methylbutan-2-ol (**3f**) was obtained following the procedure A for the cyclopropanation reaction, from the corresponding allylsilane in 20 hours, to give, after column chromatography (hexane/EtOAc: 6:1), a yellow oil in 70% chemical yield (1.1 g from 6 mmol of the corresponding allylsilyl alcohol). **^{1}H NMR (500 MHz, $CDCl_3$)** δ (ppm) 7.55 – 7.50 (m, 2H, Ar-*H*), 7.38 – 7.32 (m, 3H, Ar-*H*), 1.67 (dd,

J = 14.6, 3.4 Hz, 1H, C*H*H), 1.54 (dd, J = 14.6, 8.8 Hz, 1H, CH*H*), 1.13 (s, 3H, CH_3), 1.10 (s, 3H, CH_3), 0.68 – 0.61 (m, 1H, CH), 0.59 – 0.53 (m, 1H, C*H*H), 0.51 – 0.48 (m, 1H, CH*H*), 0.47 – 0.43 (m, 1H, Si-CH), 0.35 (s, 3H, Si-CH_3), 0.35 (s, 3H, Si-CH_3), 0.17 – 0.14 (m, 2H, CH_2). **^{13}C NMR (101 MHz, $CDCl_3$)** δ (ppm) 139.0 (C), 134.1 (CH), 129.1 (CH), 127.9 (CH), 71.61 (C), 43.8 (CH_2), 30.5 (CH_3), 29.1 (CH_3), 26.1 (CH), 14.3 (CH), 6.9 (CH_2), 6.8 (CH_2), -3.0 (Si-CH_3), -4.0 (Si-CH_3). **HRMS (ESI+)** m/z calc. for $C_{16}H_{26}NaOSi$ ($[M+Na]^+$): 285.1645, found 285.1648.

2.2. TMSCl/$BiCl_3$-promoted cyclization of cyclopropylsilyl alcohols **3a-e**

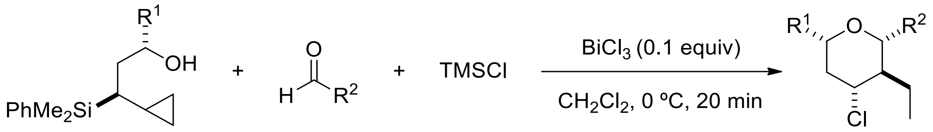

3a-e (1 equiv) (1.2 equiv) (1.2 equiv) **4**

A solution of $BiCl_3$ (0.1 equiv) and the corresponding aldehyde (1.2 equiv) in dry dichloromethane (0.05 M) was cooled to 0 °C (under nitrogen). Then, the corresponding alcohol **3** (80 mg, 1.0 equiv) was dissolved in 1 mL of dry dichloromethane and added into the reaction. Finally, the Lewis acid, TMSCl (1.2 equiv) was added dropwise. The mixture was stirred at 0 ºC while monitored by TLC. When starting materials were consumed, it was hydrolyzed with 5 mL of $NaHCO_3$ (sat). Phases are then separated, extracting the aqueous phase three times with dichloromethane (3 x 10 mL). The organic phases are combined, washed with NaCl sat. (20 mL) and dried over anhydrous Na_2SO_4. The solvent is then evaporated under reduced pressure. The crude mixture is analyzed by NMR and then purified by column chromatography in silica gel, using mixtures of hexane-ethyl acetate and yielding tetrahydropyrans **4**.

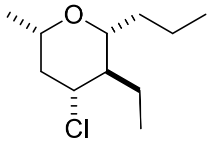

(2*R**,3*R**,4*R**,6*S**)-4-chloro-3-ethyl-6-methyl-2-propyltetrahydro-2*H*-pyran (**4a**) was obtained following the general procedure, from cyclopropylsilyl alcohol **3a** (80 mg, 0.32 mmol) and butyraldehyde in 25 minutes, to give, after column chromatography (hexane/EtOAc: 50:1), a colourless oil (46 mg, 70%). **^{1}H NMR (500 MHz, $CDCl_3$)** δ (ppm) 3.96 (td, J = 11.4, 4.6 Hz, 1H, *H*C-Cl), 3.45 – 3.46 (m, 1H, *H*C-O), 3.17 (ddd, J = 9.9, 8.4, 2.4 Hz, 1H, O-C*H*), 2.17 (ddd, J = 12.7, 4.7, 1.9 Hz, 1H, C*H*H), 1.82 – 1.72 (m, 1H), 1.72 – 1.61 (m, 1H, CH*H*), 1.60 – 1.53 (m, 2H), 1.52 – 1.29 (m, 4H), 1.19 (d, J = 6.2 Hz, 3H, CH_3), 0.91 (t, J = 7.1 Hz, 3H, CH_3), 0.86 (t, J = 7.6 Hz, 3H, CH_3). **^{13}C NMR (101 MHz, $CDCl_3$)** δ (ppm) 78.9 (O-CH), 72.6 (HC-O), 61.0 (HC-Cl), 49.1 (CH), 45.1 (CH_2), 35.3 (CH_2), 21.6 (CH_3), 20.4 (CH_2), 18.7 (CH_2), 14.2 (CH_3), 9.2 (CH_3). **HRMS (ESI+)** m/z calc. for $C_{11}H_{21}ClNaO$ ($[M+Na]^+$): 227.1173, found 227.1176.

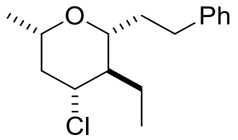

(2*R**,3*R**,4*R**,6*S**)-4-chloro-3-ethyl-6-methyl-2-phenethyltetrahydro-2*H*-pyran (**4b**) was obtained following the general procedure, from cyclopropylsilyl alcohols **3a** or **3b** (80 mg, 0.32 mmol) and 3-phenylpropanal in 10 minutes, to give, after column chromatography (hexane/EtOAc: 30:1), a colourless oil (71 mg, 83% from cyclopropylsilyl alcohol **3a** or 73 mg, 85% from cyclopropylsilyl alcohol **3b**). **^{1}H NMR (500 MHz, $CDCl_3$)** δ (ppm) 7.31 – 7.26 (m, 2H, Ar-*H*), 7.21 – 7.16 (m, 3H, Ar-*H*), 3.92 (td, J = 11.3, 4.7 Hz, 1H, *H*C-Cl), 3.44 – 3.36 (m, 1H, *H*C-O), 3.11 (td, J = 9.6, 2.6 Hz, 1H, O-C*H*), 2.86 (ddd, J = 13.8, 9.4, 4.6 Hz, 1H, C*H*H-Ph), 2.67 (ddd, J = 13.8, 9.1, 7.6 Hz, 1H, CH*H*-Ph), 2.19 (ddd, J = 12.8, 4.7, 1.9 Hz, 1H, C*H*H), 1.99 – 1.90 (m, 1H), 1.79 – 1.65 (m, 3H), 1.60 – 1.45 (m, 2H,), 1.24 (d, J = 6.2 Hz, 3H, CH_3), 0.78 (t, J = 7.6 Hz, 3H, CH_2-CH_3). **^{13}C NMR (101 MHz, $CDCl_3$)** δ (ppm) 142.4 (C), 128.7

(CH), 128.4 (CH), 125.9 (CH), 78.0 (O-CH), 72.6 (HC-O), 60.8 (HC-Cl), 49.1 (CH), 45.0 (CH_2), 34.8 (CH_2), 31.7 (CH_2), 21.6 (CH_3), 20.3 (CH_2), 9.1 (CH_3). **HRMS (ESI+)** m/z calc. for $C_{16}H_{23}ClNaO$ ($[M+Na]^+$): 289.1331, found 289.1330.

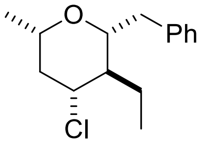

(2*R**,3*R**,4*R**,6*S**)-2-benzyl-4-chloro-3-ethyl-6-methyltetrahydro-2*H*-pyran (**4c**) was obtained following the general procedure, from cyclopropylsilyl alcohol **3a** (80 mg, 0.32 mmol) and phenylacetaldehyde, in 10 minutes, to give, after column chromatography (hexane/EtOAc: 30:1), a colourless oil (57 mg, 70%). **^{1}H NMR (500 MHz, $CDCl_3$)** δ (ppm) 7.28 – 7.25 (m, 4H, Ar-*H*), 7.24 – 7.18 (m, 1H, Ar-*H*), 3.98 (td, *J* = 11.4, 4.6 Hz, 1H, *H*C-Cl), 3.42 (ddd, *J* = 10.7, 8.5, 2.5 Hz, 1H, O-C*H*), 3.38 – 3.31 (m, 1H, *H*C-O), 3.01 (dd, *J* = 14.4, 2.5 Hz, 1H, C*H*H-Ph), 2.69 (dd, *J* = 14.4, 8.5 Hz, 1H, CH*H*-Ph), 2.16 (ddd, *J* = 12.8, 4.6, 2.0 Hz, 1H, C*H*H), 1.93 – 1.83 (m, 1H, C*H*H-CH_3), 1.74 – 1.63 (m, 2H), 1.57 – 1.50 (m, 1H, CH), 1.15 (d, *J* = 6.2 Hz, 3H, CH_3), 0.93 (t, *J* = 7.6 Hz, 3H, CH_2-CH_3). **^{13}C NMR (101 MHz, $CDCl_3$)** δ (ppm) 139.3 (C), 129.7 (CH), 128.1 (CH), 126.2 (CH), 79.9 (O-CH), 72.4 (HC-O), 60.7 (HC-Cl), 48.7 (CH), 44.9 (CH_2), 39.4 (CH_2), 21.5 (CH_3), 20.5 (CH_2), 9.1 (CH_3). **HRMS (ESI+)** m/z calc. for $C_{15}H_{21}ClNaO$ ($[M+Na]^+$): 275.1173, found 275.1168.

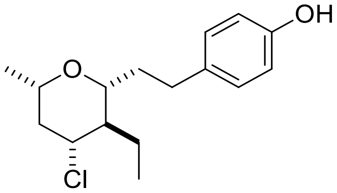

4-(2-((2*R**,3*R**,4*R**,6*S**)-4-chloro-3-ethyl-2-(4-hydroxyphenethyl)-6-methyltetrahydro-2*H*-pyran (**4d**) was obtained following the general procedure, from cyclopropylsilyl alcohol **3a** (80 mg, 0.32 mmol) and 3-(4-hydroxyphenyl)propanal in 30 minutes, to give, after column chromatography (hexane/EtOAc: 4:1), a colourless oil (45 mg, 50%). **^{1}H NMR (500 MHz, $CDCl_3$)** δ (ppm) 7.06 (d, *J* = 8.4 Hz, 2H, Ar-*H*), 6.75 (d, *J* = 8.4 Hz, 2H, Ar-*H*), 4.52 (br s, 1H, OH), 3.92 (td, *J* = 11.3, 4.6 Hz, 1H, *H*C-Cl), 3.43 – 3.34 (m, 1H, *H*C-O), 3.08 (td, *J* = 9.7, 2.6 Hz, 1H, O-C*H*), 2.78 (ddd, *J* = 13.8, 9.3, 4.6 Hz, 1H, C*H*H-Ph), 2.61 (dt, *J* = 13.8, 8.4 Hz, 1H, CH*H*-Ph), 2.19 (ddd, *J* = 12.7, 4.6, 1.9 Hz, 1H, C*H*H), 1.93 – 1.85 (m, 1H), 1.77 – 1.64 (m, 3H), 1.60 – 1.44 (m, 2H), 1.24 (d, *J* = 6.2 Hz, 3H, CH_3), 0.77 (t, *J* = 7.6 Hz, 3H, CH_2-C*H_3*). **^{13}C NMR (101 MHz, $CDCl_3$)** δ (ppm) 153.8 (C), 134.4 (C), 129.8 (CH), 115.4 (CH), 78.1 (O-CH), 72.7 (HC-O), 60.7 (HC-Cl), 49.1 (CH), 45.0 (CH_2), 35.0 (CH_2), 30.8 (CH_2), 21.6 (CH_3), 20.3 (CH_2), 9.1 (CH_3). **HRMS (ESI+)** m/z calc. for $C_{16}H_{23}ClNaO_2$ ($[M+Na]^+$): 305.1279, found 305.1277.

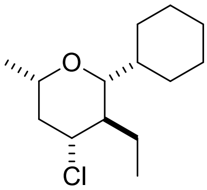

(2*R**,3*R**,4*R**,6*S**)-4- chloro-2-cyclohexyl-3-ethyl-6-methyltetrahydro-*2H*-pyran (**4e**) was obtained following the general procedure, from cyclopropylsilyl alcohol **3a** (80 mg, 0.32 mmol) and cyclohexanecarbaldehyde, in 25 minutes, to give, after column chromatography (hexane/EtOAc: 50:1), a colourless oil (66 mg, 85%). **^{1}H NMR (500 MHz, $CDCl_3$)** δ (ppm) 3.98 (td, *J* = 11.4, 4.6 Hz, 1H, *H*C-Cl), 3.39 – 3.31 (m, 1H, *H*C-O), 2.99 (dd, *J* = 10.2, 1.4 Hz, 1H, O-C*H*), 2.15 (ddd, *J* = 12.7, 4.6, 2.0 Hz, 1H, C*H*H), 1.80 – 1.71 (m, 3H), 1.68 – 1.61 (m, 2H), 1.61 – 1.54 (m, 3H), 1.54 – 1.49 (m, 2H), 1.44 – 1.39 (m, 1H, CH*H*), 1.31 – 1.21 (m, 3H), 1.21 – 1.17 (m, 1H, CH*H*), 1.16 (d, *J* = 6.2 Hz, 3H, CH_3), 0.86 (t, *J* = 7.6 Hz, 3H, CH_2-C*H_3*). **^{13}C NMR (101 MHz, $CDCl_3$)** δ (ppm) 83.0 (O-CH), 72.7 (HC-O), 61.7 (HC-Cl), 45.5 (CH), 45.2 (CH_2), 39.0 (CH), 31.2 (CH_2), 27.1 (CH_2), 26.6 (2*CH_2), 25.2 (CH_2), 21.6 (CH_3), 20.0 (CH_2), 9.0 (CH_3). **HRMS (ESI+)** m/z calc. for $C_{14}H_{25}ClNaO$ ($[M+Na]^+$): 267.1492, found 267.1489.

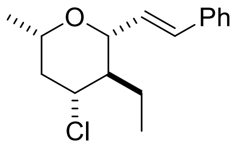

(2R*,3R*,4R*,6S*)-4-chloro-3-ethyl-6-methyl-2-((E)-styryl)tetrahydro-2H-pyran (**4f**) was obtained following the general procedure, from cyclopropylsilyl alcohols **3a** or **3b** (80 mg, 0.32 mmol) and (E)-cinnamaldehyde in 20 minutes, to give, after column chromatography (hexane/EtOAc: 25:1), a colourless oil (61 mg, 72% from cyclopropylsilyl alcohol **3a** or 53 mg, 63% from cyclopropylsilyl alcohol **3b**). **^{1}H NMR (500 MHz, $CDCl_3$)** δ (ppm) 7.41 – 7.38 (m, 2H, Ar-H), 7.34 – 7.29 (m, 2H, Ar-H), 7.27 – 7.23 (m, 1H, Ar-H), 6.64 (d, J = 15.9 Hz, 1H, =CH), 6.16 (dd, J = 15.9, 8.0 Hz, 1H, HC=), 4.02 (ddd, J = 11.8, 10.8, 4.5 Hz, 1H, HC-Cl), 3.84 (dd, J = 9.9, 8.0 Hz, 1H, O-CH), 3.61 – 3.53 (m, 1H, HC-O), 2.23 (ddd, J = 12.9, 4.6, 2.0 Hz, 1H, CHH), 1.81 – 1.71 (m, 2H), 1.67 – 1.57 (m, 2H), 1.26 (d, J = 6.2 Hz, 3H, CH_3), 0.89 (t, J = 7.5 Hz, 3H, CH_3). **^{13}C NMR (101 MHz, $CDCl_3$)** δ (ppm) 136.6 (C), 133.4 (=CH), 128.9 (CH), 128.0 (HC=), 128.0 (CH), 126.8 (CH), 81.2 (O-CH), 72.6 (HC-O), 60.1 (HC-Cl), 49.5 (CH), 44.8 (CH_2), 21.6 (CH_3), 20.6 (CH_2), 9.3 (CH_3). **HRMS (ESI+)** m/z calc. for $C_{16}H_{21}ClNaO$ ($[M+Na]^+$): 287.1173, found 287.1179.

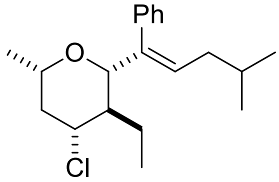

(2R*,3R*,4R*,6S*)-4-chloro-3-ethyl-6-methyl-2-((E)-4-methyl-1-phenylpent-1-en-1-yl)tetrahydro-2H-pyran (**4g**) was obtained following the general procedure, from cyclopropylsilyl alcohol **3a** (80 mg, 0.32 mmol) and (E)-5-methyl-2-phenylhex-2-enal in 30 minutes, to give, after column chromatography (hexane/EtOAc: 25:1), a yellow oil (62 mg, 60%). **^{1}H NMR (500 MHz, $CDCl_3$)** δ (ppm) 7.35 – 7.25 (m, 5H, Ar-H), 5.77 (t, J = 7.4 Hz, 1H, =CH), 4.02 (td, J = 11.4, 4.7 Hz, 1H, HC-Cl), 3.86 (d, J = 10.2 Hz, 1H, O-CH), 3.555 – 3.48 (m, 1H, HC-O), 2.13 (ddd, J = 12.7, 4.5, 2.0 Hz, 1H, CHH), 1.95 – 1.81 (m, 2H, CH_2), 1.80 – 1.71 (m, 1H, CHH-CH_3), 1.70 – 1.62 (m, 1H, CH), 1.64 – 1.55 (m, 1H, CHH), 1.53 – 1.44 (m, 2H), 1.24 (d, J = 6.1 Hz, 3H, CH_3), 0.89 (d, J = 6.6 Hz, 3H, CH_3), 0.85 (d, J = 6.6 Hz, 3H, CH_3), 0.83 (t, J = 7.6 Hz, 3H, CH_3). **^{13}C NMR (101 MHz, $CDCl_3$)** δ (ppm) 139.8 (C=), 138.2 (C), 133.2 (=CH), 129.9 (CH), 127.8 (CH), 126.9 (CH), 85.7 (O-CH), 72.6 (HC-O), 60.2 (HC-Cl), 46.1 (CH), 44.8 (CH_2), 37.7 (CH_2), 29.0 (CH), 22.7 (CH_3), 22.5 (CH_3), 21.8 (CH_3), 20.3 (CH_2), 8.2 (CH_3). **HRMS (ESI+)** m/z calc. for $C_{20}H_{29}ClNaO$ ($[M+Na]^+$): 343.1799, found 343.1808.

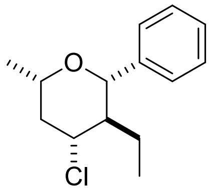

(2R*,3R*,4R*,6S*)-4-chloro-3-ethyl-6-methyl-2-phenyltetrahydro-2H-pyran (**4h**) was obtained following the general procedure, from cyclopropylsilyl alcohol **3a** (80 mg, 0.32 mmol) and benzaldehyde in 25 minutes, to give, after column chromatography (hexane/EtOAc: 25:1), a colourless oil (61 mg, 80%). **^{1}H NMR (500 MHz, $CDCl_3$)** δ (ppm) 7.37 – 7.33 (m, 4H, Ar-H), 7.32 – 7.27 (m, 1H, Ar-H), 4.16 (d, J = 10.2 Hz, 1H, O-CH), 4.10 (td, J = 11.4, 4.6 Hz, 1H, HC-Cl), 3.67 – 3.58 (m, 1H, HC-O), 2.29 (ddd, J = 13.0, 4.6, 1.9 Hz, 1H, CHH), 191 – 1.81 (m, 2H), 1.64 – 1.55 (m, 1H, CHH-CH_3), 1.25 (d, J = 6.2 Hz, 3H, CH_3), 1.24 – 1.16 (m, 1H, CHH-CH_3), 0.69 (t, J = 7.6 Hz, 3H, CH_3). **^{13}C NMR (101 MHz, $CDCl_3$)** δ (ppm) 140.4 (C), 128.7 (CH), 128.3 (CH), 127.6 (CH), 127.6 (CH), 83.2 (O-CH), 73.2 (HC-O), 60.5 (HC-Cl), 50.5 (CH), 45.0 (CH_2), 21.7 (CH_3), 20.5 (CH_2), 9.1 (CH_3). **HRMS (ESI+)** m/z calc. for $C_{14}H_{19}ClNaO$ ($[M+Na]^+$): 261.1017, found 261.1019.

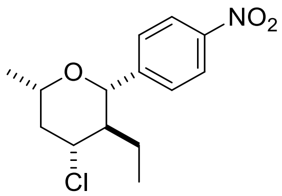

(2*R**,3*R**,4*R**,6*S**)-4-chloro-3-ethyl-6-methyl-2-(4-nitrophenyl)tetrahydro-2*H*-pyran (**4i**) was obtained following the general procedure, from cyclopropylsilyl alcohol **3a** (80 mg, 0.32 mmol) and 4-nitrobenzaldehyde in 25 minutes, to give, after column chromatography (hexane/EtOAc: 15:1), a yellow solid (66 mg, 73%). **^{1}H NMR (500 MHz, $CDCl_3$)** δ (ppm) 8.22 (d, *J* = 8.8 Hz, 2H, Ar-*H*), 7.53 (d, *J* = 8.8 Hz, 2H, Ar-*H*), 4.28 (d, *J* = 10.2 Hz, 1H, O-C*H*), 4.08 (td, *J* = 11.4, 4.6 Hz, 1H, *H*C-Cl), 3.70 – 3.61 (m, 1H, *H*C-O), 2.32 (ddd, *J* = 13.0, 4.6, 1.9 Hz, 1H, C*H*H), 1.87 (dt, *J* = 13.0, 11.5 Hz, 1H, CH*H*), 1.82 – 1.76 (m, 1H, C*H*), 1.67 – 1.58 (m, 1H, C*H*H-CH_3), 1.26 (d, *J* = 6.3 Hz, 3H, CH_3), 1.23 – 1.13 (m, 1H, CH*H*-CH_3), 0.71 (t, *J* = 7.5 Hz, 3H, CH_3). **^{13}C NMR (101 MHz, $CDCl_3$)** δ (ppm) 147.8 (C), 147.6 (C), 128.5 (CH), 123.8 (CH), 81.9 (O-CH), 73.4 (HC-O), 59.6 (HC-Cl), 50.6 (CH), 44.6 (CH_2), 21.5 (CH_3), 20.2 (CH_3), 9.2 (CH_3). HRMS (ESI+) m/z calc. for $C_{14}H_{18}ClNNaO_3$ ($[M+Na]^+$): 306.0867, found 306.0866. **Melting point** = 90.3 – 92.0 °C.

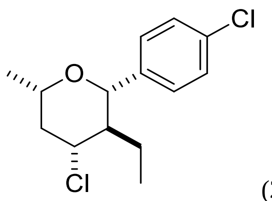

(2*S**,3*R**,4*R**,6*S**)-4-chloro-2-(4-chlorophenyl)-3-ethyl-6-methyltetrahydro-*2H*-pyran (**4j**) was obtained following the general procedure, from cyclopropylsilyl alcohols **3a** or **3b** (80 mg, 0.32 mmol) and 4-chlorobenzaldehyde in 30 minutes, to give, after column chromatography (hexane/EtOAc: 20:1), a pale pink solid (74 mg, 85% from cyclopropylsilyl alcohol **3a** or 61 mg, 70% from cyclopropylsilyl alcohol **3b**). **^{1}H NMR (500 MHz, $CDCl_3$)** δ (ppm) 7.33 (d, *J* = 8.5 Hz, 2H, Ar-*H*), 7.28 (d, *J* = 8.5 Hz, 2H, Ar-*H*), 4.14 (d, *J* = 10.2 Hz, 1H, O-C*H*), 4.07 (td, *J* = 11.4, 4.6 Hz, 1H, *H*C-Cl), 3.66 – 3.57 (m, 1H, *H*C-O), 2.28 (ddd, *J* = 12.9, 4.6, 1.9 Hz, 1H, C*H*H), 1.84 (dt, *J* = 12.9, 11.5 Hz, 1H, CH*H*), 1.82 – 1.74 (m, 1H, C*H*), 1.66 – 1.55 (m, 1H, C*H*H-CH_3), 1.25 (d, *J* = 6.2 Hz, 3H, CH_3), 1.24 – 1.14 (m, 1H, CH*H*-CH_3), 0.69 (t, *J* = 7.6 Hz, 3H, CH_3). **^{13}C NMR (101 MHz, $CDCl_3$)** δ (ppm) 138.9 (C), 134.0 (C), 129.0 (CH), 128.8 (CH), 82.3 (O-CH), 73.2 (HC-O), 60.2 (HC-Cl), 50.5 (CH), 44.8 (CH_2), 21.6 (CH_3), 20.4 (CH_2), 9.2 (CH_3). **HRMS (ESI+)** m/z calc. for $C_{14}H_{18}Cl_2NaO$ ($[M+Na]^+$): 295.0627, found 295.0631. **Melting point** = 51.4 – 53.1 °C.

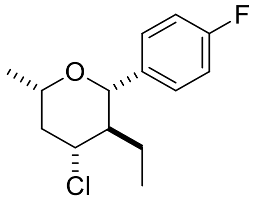

(2*S**,3*R**,4*R**,6*S**)-4-chloro-3-ethyl-2-(4-fluorophenyl)-6-methyltetrahydro-2*H*-pyran (**4k**) was obtained following the general procedure, from cyclopropylsilyl alcohol **3a** (80 mg, 0.32 mmol) and 4-fluorobenzaldehyde in 20 minutes, to give, after column chromatography (hexane/EtOAc: 25:1), a pale pink solid (68 mg, 83%). **^{1}H NMR (500 MHz, $CDCl_3$)** δ (ppm) 7.35 – 7.29 (m, 2H, Ar-*H*), 7.07 – 7.00 (m, 2H, Ar-*H*), 4.15 (d, *J* = 10.2 Hz, 1H, O-C*H*), 4.07 (td, *J* = 11.4, 4.6 Hz, 1H, *H*C-Cl), 3.67 – 3.57 (m, 1H, *H*C-O), 2.28 (ddd, *J* = 12.9, 4.6, 1.9 Hz, 1H, C*H*H), 1.87 (dt, *J* = 12.9, 11.5 Hz, 1H, CH*H*), 1.82 – 1.74 (m, 1H, C*H*), 1.65 – 1.55 (m, 1H, C*H*H-CH_3), 1.25 (d, *J* = 6.2 Hz, 3H, CH_3), 1.24 – 1.15 (m, 1H, CH*H*-CH_3), 0.69 (t, *J* = 7.6 Hz, 3H, CH_3). **^{13}C NMR (101 MHz, $CDCl_3$)** δ (ppm) 162.7 (d, $^{1}J_{C-F}$ = 246.2 Hz, C), 136.3 (d, $^{4}J_{C-F}$ = 3.2 Hz, C), 129.2 (d, $^{3}J_{C-F}$ = 8.1 Hz, CH), 115.6 (d, $^{2}J_{C-F}$ = 21.4 Hz, CH), 81.4 (O-CH), 73.2 (HC-O), 60.3 (HC-Cl), 50.6 (CH), 44.9 (CH_2), 21.7 (CH_3), 20.5 (CH_2), 9.2 (CH_3). **HRMS (ESI+)** m/z calc. for $C_{14}H_{18}ClFNaO$ ($[M+Na]^+$): 279.0922, found 279.0924. **Melting point** = 62.9 – 64.5 °C.

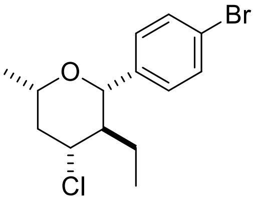

2S*,3R*,4R*,6S*)-2-(4-bromophenyl)-4-chloro-3-ethyl-6-methyltetrahydro-2H-pyran (**4l**) was obtained following the general procedure, from cyclopropylsilyl alcohol **3a** (80 mg, 0.32 mmol) and 4-bromobenzaldehyde in 25 minutes, to give, after column chromatography (hexane/EtOAc: 20:1), a white solid (71 mg, 71%). **^{1}H NMR (500 MHz, $CDCl_3$)** δ (ppm) 7.48 (d, J = 8.4 Hz, 2H, Ar-H), 7.23 (d, J = 8.4 Hz, 2H, Ar-H), 4.13 (d, J = 10.3 Hz, 1H, O-CH), 4.07 (td, J = 11.4, 4.6 Hz, 1H, HC-Cl), 3.66 – 3.57 (m, 1H, HC-O), 2.28 (ddd, J = 13.0, 4.6, 1.9 Hz, 1H, CHH), 1.89 – 1.79 (m, 1H, CHH), 1.81 – 1.74 (m, 1H, CH), 1.65 – 1.55 (m, 1H, CHH-CH_3), 1.24 (d, J = 6.2 Hz, 3H, CH_3), 1.24 – 1.15 (m, 1H, CHH-CH_3), 0.70 (t, J = 7.6 Hz, 3H, CH_2-CH_3). **^{13}C NMR (101 MHz, $CDCl_3$)** δ (ppm) 139.4 (C), 131.8 (C), 129.3 (CH), 122.2 (CH), 82.4 (O-CH), 73.3 (HC-O), 60.1 (HC-Cl), 50.4 (CH), 49.9 (CH_2), 21.7 (CH_3), 20.4 (CH_2), 9.2 (CH_3). **HRMS (ESI+)** m/z calc. for $C_{14}H_{18}ClBrNaO$ ($[M+Na]^+$): 339.0122, found 339.0110. **Melting point** = 53.1 – 54.6 °C.

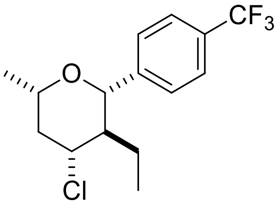

2S*,3R*,4R*,6S*)-4-chloro-3-ethyl-6-methyl-2-(4-(trifluoromethyl)phenyl)-tetrahydro-2H-pyran (**4m**) was obtained following the general procedure, from cyclopropylsilyl alcohol **3a** (80 mg, 0.32 mmol) and 4-trifluoromethylbenzaldehyde in 25 minutes, to give, after column chromatography (hexane/EtOAc: 25:1), a colourless oil (76 mg, 77%). **^{1}H NMR (500 MHz, $CDCl_3$)** δ (ppm) 7.62 (d, J = 8.0 Hz, 2H, Ar-H), 7.47 (d, J = 8.0 Hz, 2H Ar-H), 4.23 (d, J = 10.3 Hz, 1H, O-CH), 4.09 (td, J = 11.4, 4.6 Hz, 1H, HC-Cl), 3.68 – 3.60 (m, 1H, HC-O), 2.30 (ddd, J = 12.9, 4.6, 2.0 Hz, 1H, CHH), 1.91 – 1.81 (m, 1H, CHH), 1.85 – 1.78 (m, 1H, CH), 1.67 – 1.57 (m, 1H, CHH-CH_3), 1.26 (d, J = 6.2 Hz, 3H, CH_3), 1.23 – 1.12 (m, 1H, CHH-CH_3), 0.71 (t, J = 7.6 Hz, 3H, CH_2-CH_3). **^{13}C NMR (101 MHz, $CDCl_3$)** δ (ppm) 144.3 (C), 130.6 (q, $^{2}J_{C-F}$ = 32.4 Hz, C), 128.0 (CH), 125.7 (q, $^{4}J_{C-F}$ = 3.2 Hz, CH), 124.2 ((q, $^{1}J_{C-F}$ = 271 Hz, C), 82.4 (O-CH), 73.4 (HC-O), 59.9 (HC-Cl), 50.4 (CH), 44.9 (CH_2), 21.6 (CH_3), 20.3 (CH_2), 9.1 (CH_3). **HRMS (ESI+)** m/z calc. for $C_{15}H_{18}ClF_3NaO$ ($[M+Na]^+$): 329.0890, found 329.0895.

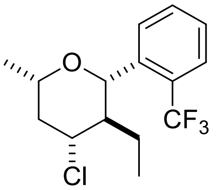

2S*,3R*,4R*,6S*)-4-chloro-3-ethyl-6-methyl-2-(2-(trifluoromethyl)phenyl)-tetrahydro-2H-pyran (**4n**) was obtained following the general procedure, from cyclopropylsilyl alcohol **3a** (80 mg, 0.32 mmol) and 2-trifluoromethylbenzaldehyde in 25 minutes, to give, after column chromatography (hexane/EtOAc: 20:1),a pale yellow crystalline solid (62 mg, 63%). **^{1}H NMR (500 MHz, $CDCl_3$)** δ (ppm) 7.71 – 7.65 (m, 2H, Ar-H), 7.60 (t, J = 7.6 Hz, 1H, Ar-H), 7.42 (t, J = 7.6 Hz, 1H Ar-H), 4.57 (d, J = 10.3 Hz, 1H, O-CH), 4.08 (td, J = 11.3, 4.6 Hz, 1H, HC-Cl), 3.71 – 3.62 (m, 1H, HC-O), 2.30 (ddd, J = 13.0, 4.6, 1.9 Hz, 1H, CHH), 2.10 – 2.02 (m, 1H, CH), 1.91 – 1.81 (m, 1H, CHH), 1.55 – 1.43 (m, 1H, CHH-CH_3), 1.40 – 1.29 (m, 1H, CHH-CH_3), 1.23 (d, J = 6.2 Hz, 3H, CH_3), 0.64 (t, J = 7.6 Hz, 3H, CH_3). **^{13}C NMR (101 MHz, $CDCl_3$)** δ (ppm) 138.6 (q, $^{3}J_{C-F}$ = 1.3 Hz, C), 132.43 (CH), 129.0 (q, $^{2}J_{C-F}$ = 29.8 Hz, C), 128.4 (CH), 126.0 (q, $^{3}J_{C-F}$ = 5.8 Hz, CH), 124.4 (q, $^{1}J_{C-F}$ = 274 Hz, C), 77.7 (O-CH), 73.5 (HC-O), 61.2 (HC-Cl), 49.6 (CH), 44.9 (CH_2), 21.6 (CH_3), 20.8 (CH_2), 10.2 (CH_3). **HRMS (ESI+)** m/z calc. for $C_{15}H_{18}ClF_3NaO$ ($[M+Na]^+$): 329.0890, found 329.0895. **Melting point =** 67.6 – 69.8 °C.

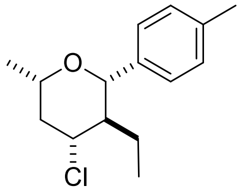

(2*S**,3*R**,4*R**,6*S**)-4-chloro-3-ethyl-6-methyl-2-(*p*-tolyl)tetrahydro-2*H*-pyran (**4o**) was obtained following the general procedure, from cyclopropylsilyl alcohol **3a** (80 mg, 0.32 mmol) and 4-methylbenzaldehyde in 20 minutes, to give, after column chromatography (hexane/EtOAc: 30:1), a pale pink solid (57 mg, 70%). **^{1}H NMR (500 MHz, $CDCl_3$)** δ (ppm) 7.22 (d, *J* = 8.1 Hz, 2H, Ar-*H*), 7.15 (d, *J* = 8.1 Hz, 2H, Ar-*H*), 4.12 (d, *J* = 10.2 Hz, 1H, O-C*H*), 4.09 (td, *J* = 11.4, 4.6 Hz, 1H, *H*C-Cl), 3.64 – 3.57 (m, 1H, *H*C-O), 2.33 (s, 3H, CH_3), 2.27 (ddd, *J* = 12.9, 4.6, 1.9 Hz, 1H, C*H*H), 1.89 – 1.70 (m, 2H), 1.64 – 1.56 (m, 1H, C*H*H-CH_3), 1.26 – 1.16 (m, 1H, CH*H*-CH_3), 1.24 (d, *J* = 6.2 Hz, 3H, CH_3), 0.69 (t, *J* = 7.6 Hz, 3H, CH_3). **^{13}C NMR (101 MHz, $CDCl_3$)** δ (ppm) 138.0 (C), 137.4 (C), 129.3 (CH), 127.5 (CH), 83.0 (O-CH), 73.2 (HC-O), 60.6 (HC-Cl), 50.3 (CH), 45.0 (CH_2), 21.7 (CH_3), 21.3 (CH_3), 20.6 (CH_2), 9.1 (CH_3). **HRMS (ESI+)** m/z calc. for $C_{15}H_{21}ClNaO$ ($[M+Na]^+$): 275.1173, found 275.1175. **Melting point** = 44.6 – 45.1 °C.

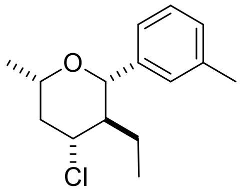

(2*S**,3*R**,4*R**,6*S**)-4-chloro-3-ethyl-6-methyl-2-(*m*-tolyl)tetrahydro-2*H*-pyran (**4p**) was obtained following the general procedure, from cyclopropylsilyl alcohol **3a** (80 mg, 0.32 mmol) and 3-methylbenzaldehyde in 10 minutes, to give, after column chromatography (hexane/EtOAc: 20:1), a yellow solid (61 mg, 75%). **^{1}H NMR (500 MHz, $CDCl_3$)** δ (ppm) 7.23 (t, *J* = 7.5 Hz, 1H, Ar-*H*), 7.16 (s, 1H, Ar-*H*), 7.12 (t, *J* = 7.5 Hz, 2H, Ar-*H*), 4.13 (d, *J* = 10.3 Hz, 1H, O-C*H*), 4.10 (td, *J* = 11.4, 4.5 Hz, 1H, *H*C-Cl), 3.66 – 3.58 (m, 1H, *H*C-O), 2.36 (s, 3H, C*H₃*), 2.28 (ddd, *J* = 12.9, 4.6, 1.9 Hz, 1H, C*H*H), 1.91 – 1.80 (m, 2H), 1.65 – 1.56 (m, 1H, C*H*H-CH_3), 1.25 (d, *J* = 6.2 Hz, 3H, CH_3), 1.24 – 1.16 (m, 1H, CH*H*-CH_3), 0.70 (t, *J* = 7.6 Hz, 3H, CH_3). **^{13}C NMR (101 MHz, $CDCl_3$)** δ (ppm) 140.2 (C), 138.3 (C), 129.1 (CH), 128.5 (CH), 128.2 (CH), 124.8 (CH), 83.2 (O-CH), 73.2 (HC-O), 60.5 (HC-Cl), 50.2 (CH), 45.0 (CH_2), 21.7 (CH_3), 21.6 (CH_3), 20.5 (CH_2), 9.1 (CH_3). **HRMS (ESI+)** m/z calc. for $C_{15}H_{21}ClNaO$ ($[M+Na]^+$): 275.1173, found 275.1175. **Melting point** = 23.0 – 24.5 °C.

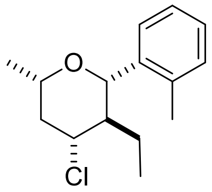

(2*S**,3*R**,4*R**,6*S**)-4-chloro-3-ethyl-6-methyl-2-(*o*-tolyl)tetrahydro-2*H*-pyran (**4q**) was obtained following the general procedure, from cyclopropylsilyl alcohol **3a** (80 mg, 0.32 mmol) and 2-methylbenzaldehyde in 20 minutes, to give, after column chromatography (hexane/EtOAc: 20:1), a viscous yellow oil (68 mg, 84%). **^{1}H NMR (400 MHz, $CDCl_3$)** δ (ppm) 7.42 – 7.32 (m, 1H, Ar-*H*), 7.24 -7.13 (m, 3H, Ar-*H*), 4.43 (d, *J* = 10.5 Hz, 1H, O-C*H*), 4.10 (td, *J* = 11.3, 4.6 Hz, 1H, *H*C-Cl), 3.69 – 3.59 (m, 1H, *H*C-O), 2.42 (s, 3H, C*H₃*), 2.29 (ddd, *J* = 12.8, 4.6, 1.9 Hz, 1H, C*H*H), 2.10 – 1.98 (m, 1H, C*H*), 1.87 (dt, *J* = 12.8, 11.5 Hz, 1H, CH*H*), 1.62 – 1.48 (m, 1H, C*H*H-CH_3), 1.36 – 1.26 (m, 1H, CH*H*-CH_3), 1.25 (d, *J* = 6.2 Hz, 3H, CH_3), 0.70 (t, *J* = 7.6 Hz, 3H, CH_3). **^{13}C NMR (101 MHz, $CDCl_3$)** δ (ppm) 138.2 (C), 136.1 (C), 132.2 (CH), 130.7 (CH), 128.0 (CH), 126.5 (CH), 77.5 (O-CH), 73.3 (HC-O), 61.1 (HC-Cl), 49.5 (CH), 45.0 (CH_2), 21.7 (CH_3), 20.8 (CH_2), 19.8 (CH_3), 9.9 (CH_3). **HRMS (ESI+)** m/z calc. for $C_{15}H_{21}ClNaO$ ($[M+Na]^+$): 275.1173, found 275.1175.

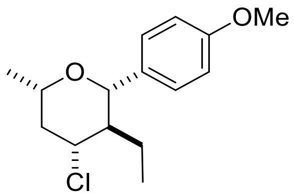

(2*S**,3*R**,4*R**,6*S**)-4-chloro-3-ethyl-2-(4-methoxyphenyl)-6-methyltetrahydro-2*H*-pyran (**4r**) was obtained following the general procedure, from cyclopropylsilyl alcohol **3a** (80 mg, 0.32 mmol) and 4-methoxybenzaldehyde in 25 minutes, to give, after column chromatography (hexane/EtOAc: 10:1), a pale pink solid (75 mg, 87%). **^{1}H NMR (500 MHz, $CDCl_3$)** δ (ppm) 7.26 (d, *J* = 8.8 Hz, 2H, Ar-*H*), 6.88 (d, *J* = 8.8 Hz, 2H, Ar-*H*), 4.11 (d, *J* = 10.2 Hz, 1H, O-C*H*), 4.08 (td, *J* = 11.4, 4.6 Hz, 1H, *H*C-Cl), 3.80 (s, 3H, O-C*H$_3$*), 3.65 – 3.57 (m, 1H, *H*C-O), 2.27 (ddd, *J* = 13.0, 4.6, 1.9 Hz, 1H, C*H*H), 1.88 – 1.79 (m, 2H), 1.63 – 1.55 (m, 1H, C*H*H-CH_3), 1.26 – 1.18 (m, 1H, CH*H*-CH_3), 1.24 (d, *J* = 6.1 Hz, 3H, C*H$_3$*), 0.71 (t, *J* = 7.6 Hz, 3H, CH_3). **^{13}C NMR (101 MHz, $CDCl_3$)** δ (ppm) 159.6 (C), 132.7 (C), 128.7 (CH), 114.0 (CH), 82.7 (O-CH), 73.1 (HC-O), 60.6 (HC-Cl), 55.4 (O-CH_3), 50.4 (CH), 45.0 (CH_2), 21.7 (CH_3), 20.6 (CH_2), 9.2 (CH_3). **HRMS (ESI+)** m/z calc. for $C_{15}H_{21}ClNaO_2$ ([M+Na]$^+$): 291.1122, found 291.1126. **Melting point** = 83.8 – 85.1 °C.

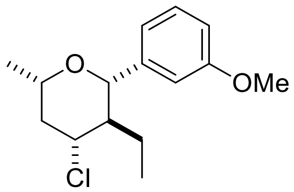

(2*S**,3*R**,4*R**,6*S**)-4-chloro-3-ethyl-2-(3-methoxyphenyl)-6-methyltetrahydro-2*H*-pyran (**4s**) was obtained following the general procedure, from cyclopropylsilyl alcohol **3a** (80 mg, 0.32 mmol) and 3-methoxybenzaldehyde in 15 minutes, to give, after column chromatography (hexane/EtOAc: 10:1), a colourless oil (58 mg, 68%). **^{1}H NMR (500 MHz, $CDCl_3$)** δ (ppm) 7.26 (dd, *J* = 8.3, 7.6 Hz, 1H, Ar-*H*), 6.93 (dd, *J* = 7.6, 1.5 Hz, 1H, Ar-*H*), 6.89 (dd, *J* = 2.6, 1.5 Hz, 1H, Ar-*H*), 6.84 (ddd, *J* = 8.3, 2.6, 1.0 Hz, 1H, Ar-*H*), 4.13 (d, *J* = 10.2 Hz, 1H, O-C*H*), 4.07 (td, *J* = 11.4, 4.6 Hz, 1H, *H*C-Cl), 3.82 (s, 3H, O-C*H$_3$*), 3.66 – 3.57 (m, 1H, *H*C-O), 2.28 (ddd, *J* = 12.9, 4.6, 1.9 Hz, 1H, C*H*H), 1.90 – 1.79 (m, 2H), 1.66 – 1.55 (m, 1H, C*H*H-CH_3), 1.28 – 1.17 (m, 1H, CH*H*-CH_3), 1.25 (d, *J* = 6.1 Hz, 3H, C*H$_3$*), 0.71 (t, *J* = 7.6 Hz, 3H, CH_2-*CH$_3$*). **^{13}C NMR (101 MHz, $CDCl_3$)** δ (ppm) 159.9 (C), 141.9 (C), 129.7 (CH), 120.1 (CH), 113.8 (CH), 113.2 (CH), 83.1 (O-CH), 73.2 (HC-O), 60.5 (HC-Cl), 55.4 (O-CH_3), 50.4 (CH), 45.0 (CH_2), 21.7 (CH_3), 20.5 (CH_2), 9.2 (CH_3). **HRMS (ESI+)** m/z calc. for $C_{15}H_{21}ClNaO_2$ ([M+Na]$^+$): 291.1122, found 291.1121.

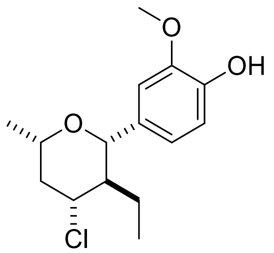

4-((2*S**,3*R**,4*R**,6*S**)-4-chloro-3-ethyl-2-(4-hydroxy-3-methoxyphenyl)-6-methyltetrahydro-2H-pyran (**4t**) was obtained following the general procedure from cyclopropylsilyl alcohol **3a** (80 mg, 0.32 mmol) and 4-hydroxy-3-methoxybenzaldehyde, in 20 minutes, to give, after column chromatography (hexane/EtOAc: 4:1), a yellow oil (86 mg, 94%). **^{1}H NMR (500 MHz, $CDCl_3$)** δ (ppm) 6.89 – 6.85 (m, 2H, Ar-H), 6.82 (dd, *J* = 8.0, 1.9 Hz, 1H, Ar-*H*), 5.58 (s, 1H, O*H*), 4.15 – 4.04 (m, 2H, O-C*H* + *H*C-Cl), 3.91 (s, 3H, O-C*H$_3$*), 3.65 – 3.56 (m, 1H, *H*C-O), 2.28 (ddd, *J* = 12.9, 4.6, 2.0 Hz, 1H, C*H*H), 1.89 – 1.77 (m, 2H), 1.64 – 1.55 (m, 1H, C*H*H-CH_3), 1.28 – 1.21 (m, 1H, CH*H*-CH_3), 1.25 (d, *J* = 6.2 Hz, 3H, CH_3), 0.70 (t, *J* = 7.6 Hz, 3H, CH_3). **^{13}C NMR (101 MHz, $CDCl_3$)** δ (ppm) 146.8 (*C*-OCH_3), 145.7 (*C*-OH), 132.3 (C), 121.0 (CH), 114.2 (CH), 109.6 (CH), 83.1 (O-CH), 73.1 (HC-O), 60.6 (HC-Cl), 56.1 (O-CH_3), 50.4 (CH), 44.9 (CH_2), 21.7 (CH_3), 20.6 (CH_2), 9.2 (CH_3). **HRMS (ESI+)** m/z calc. for $C_{15}H_{21}ClNaO_3$ ([M+Na]$^+$): 307.1071, found 307.1078.

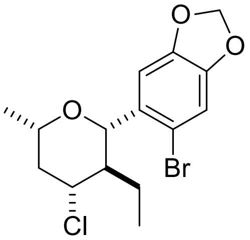
(2*S**,3*R**,4*R**,6*S**)-2-(6-bromobenzo[d][1,3]dioxol-5-yl)-4-chloro-3-ethyl-6-methyltetrahydro-2*H*-pyran (**4u**) was obtained following the general procedure, from cyclopropylsilyl alcohol **3a** (80 mg, 0.32 mmol) and 6-bromopiperonal, in 20 minutes, to give, after column chromatography (hexane/EtOAc: 6 :1), a white crystalline solid (81 mg, 70%). **^{1}H NMR (500 MHz, $CDCl_3$)** δ (ppm) 6.98 (s, 1H, Ar-*H*), 6.93 (s, 1H, Ar-*H*), 5.99 – 5.95 (m, 2H, O-CH_2-O), 4.66 (d, *J* = 10.3 Hz, 1H, O-C*H*), 4.08 (td, *J* = 11.3, 4.6 Hz, 1H, *H*C-Cl), 3.67 – 3.61 (m, 1H, *H*C-O), 2.27 (ddd, *J* = 12.9, 4.6, 1.9 Hz, 1H, C*H*H), 1.86 – 1.76 (m, 2H), 1.68 – 1.57 (m, 1H, C*H*H-CH_3), 1.34 – 1.26 (m, 1H, CH*H*-CH_3), 1.23 (d, *J* = 6.2 Hz, 3H, C*H₃*), 0.77 (t, *J* = 7.6 Hz, 3H, CH_3). **^{13}C NMR (101 MHz, $CDCl_3$)** δ (ppm) 148.3 (C), 148.1(C), 133.0 (C), 114.9 (C), 112.4 (CH), 108.6 (CH), 102.0 (CH_2), 80.7 (O-CH), 73.2 (HC-O), 60.5 (HC-Cl), 50.7 (CH), 44.9 (CH_2), 21.6 (CH_3), 20.5 (CH_2), 10.3 (CH_3). **HRMS (ESI+)** m/z calc. for $C_{15}H_{18}BrClNaO_3$ ($[M+Na]^+$): 383.0020, found 383.0017. **Melting point** = 107.8 – 109.8 °C.

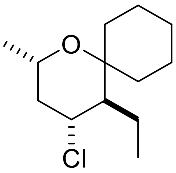
(2*S**,4*R**,5*R**)-4-chloro-5-ethyl-2-methyl-1-oxaspiro[5.5]undecane (**4v**) was obtained following the general procedure, from cyclopropylsilyl alcohol **3a** (80 mg, 0.32 mmol) and cyclohexanone in 60 minutes, to give, after column chromatography (hexane/EtOAc: 60:1), a yellow oil (51 mg, 69%). **^{1}H NMR (500 MHz, $CDCl_3$)** δ (ppm) 4.04 (td, *J* = 11.7, 4.6 Hz, 1H, HC-Cl), 3.63 – 3.54 (m, 1H, HC-O), 2.15 (ddd, *J* = 12.7, 4.6, 2.2 Hz, 1H, C*H*H), 1.88 – 1.82 (m, 1H, C*H*H), 1.74 (qt, *J* = 13.0, 3.7 Hz, 1H, Cy), 1.66 – 1.57 (m, 3H), 1.50 – 1.44 (m, 3H), 1.43 – 1.39 (m, 2H), 1.36 (qt, *J* = 12.8, 3.4 Hz, 1H, Cy), 1.28 (dt, *J* = 11.5, 4.1 Hz, 1H, C*H*), 1.25 – 1.21 (m, 1H, Cy), 1.17 (d, *J* = 6.1 Hz, 3H, C*H₃*), 1.12 (qt, *J* = 12.9, 4.1 Hz, 1H, Cy), 1.05 (t, *J* = 7.6 Hz, 3H, CH_2-*CH₃*). **^{13}C NMR (101 MHz, $CDCl_3$)** δ (ppm) 78.0 (C), 64.3 (HC-O), 62.2 (HC-Cl), 55.7 (CH), 45.8 (CH_2), 37.0 (CH_2), 26.2 (CH_2), 25.4 (CH_2), 22.7 (CH_2), 22.0 (CH_3), 21.5 (CH_2), 20.5 (CH_2), 15.6 (CH_3). **HRMS (ESI+)** m/z calc. for $C_{13}H_{23}ClNaO$ ($[M+Na]^+$): 253.1330, found 253.1327.

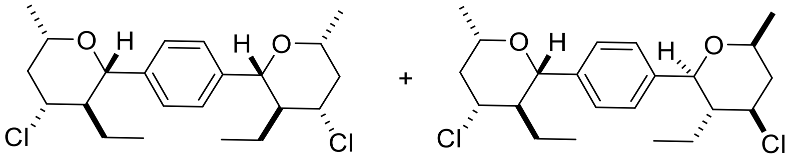
1-((2*R**,3*S**,4*S**,6*R**)-4-chloro-3-ethyl-6-methyltetrahydro-2*H*-pyran-2-yl)-4-((2*S**,3*R**,4*R**,6*S**)-4-chloro-3-ethyl-6-methyltetrahydro-2*H*-pyran-2-yl)benzene and 1-((2*R**,3*S**,4*S**,6*R**)-4-chloro-3-ethyl-6-methyltetrahydro-2*H*-pyran-2-yl)-4-((2*R**,3*S**,4*S**,6*R**)-4-chloro-3-ethyl-6-methyltetrahydro-2*H*-pyran-2-yl)benzene (**4w**) were obtained following the general procedure, from cyclopropylsilyl alcohol **3a** (80 mg, 0.32 mmol) and terephthalaldehyde in 20 minutes, to give, after column chromatography (hexane/EtOAc: 5:1), a white solid (64 mg, 50%). **^{1}H NMR (500 MHz, $CDCl_3$)** δ (ppm) 7.32 (s, 4H, Ar-*H*), 4.17 (d, *J* = 10.4 Hz, 2H, O-C*H*), 4.09 (td, *J* = 11.4, 4.5 Hz, 2H, *H*C-Cl), 3.66 – 3.56 (m, 1H, *H*C-O), 2.28 (ddd, *J* = 13.4, 4.5, 1.9 Hz, 2H, C*H*H), 1.92 – 1.77 (m, 4H), 1.66 – 1.54 (m, 2H, C*H*H-CH_3), 1.25 (d, *J* = 6.2 Hz, 6H, CH_3), 1.22 – 1.11 (m, 2H, CH*H*-CH_3), 0.70 (t, *J* = 7.5 Hz, 6H, CH_2-*CH₃*). **^{13}C NMR (101 MHz, $CDCl_3$)** δ (ppm) 140.4 (C), 127.8 (CH), 82.7 (O-CH), 73.2 (HC-O), 60.3 (HC-Cl), 50.3 (CH), 45.0 (CH_2), 21.7 (CH_3), 20.3 (CH_2), 8.9 (CH_3). **HRMS (ESI+)** m/z calc. for $C_{22}H_{32}Cl_2NaO_2$ ($[M+Na]^+$): 421.1672, found 421.1677. **Melting point** = 104.0 – 106.2 °C.

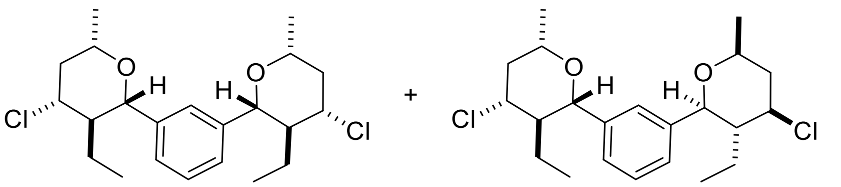 1-((2*R**,3*S**,4*S**,6*R**)-4-chloro-3-ethyl-6-methyltetrahydro-2*H*-pyran-2-yl)-3-((2*R**,3*S**,4*S**,6*R**)-4-chloro-3-ethyl-6-methyltetrahydro-2*H*-pyran-2-yl)benzene and 1-((2*R**,3*S**,4*S**,6*R**)-4-chloro-3-ethyl-6-methyltetrahydro-2*H*-pyran-2-yl)-3-((2*S**,3*R**,4*R**,6*S**)-4-chloro-3-ethyl-6-methyltetrahydro-2*H*-pyran-2-yl)benzene (**4x**) were obtained following the general procedure, from cyclopropylsilyl alcohol **3a** (80 mg, 0.32 mmol) and isophthalaldehyde in 40 minutes, to give, after column chromatography (hexane/EtOAc: 5:1), a white solid (45 mg, 35%). **^{1}H NMR (500 MHz, $CDCl_3$)** δ (ppm) 7.37 – 7.27 (m, 4H, Ar-*H*), 4.17 (d, *J* = 10.2 Hz, 1H, O-C*H*), 4.15 (d, *J* = 10.2 Hz, 1H, O-C*H*), 4.13 – 4.05 (m, 2H, *H*C-Cl), 3.65 – 3.57 (m, 2H, *H*C-O), 2.28 (ddd, *J* = 12.9, 4.6, 1.9 Hz, 2H, C*H*H), 1.90 – 1.76 (m, 4H), 1.64 – 1.54 (m, 2H, C*H*H-CH_3), 1.24 (d, *J* = 6.2 Hz, 6H, CH_3), 1.23 – 1.14 (m, 2H, CH*H*-CH_3), 0.70 (t, *J* = 7.6 Hz, 6H, CH_2-*CH_3*), 0.68 (t, *J* = 7.6 Hz, 6H, CH_2-*CH_3*). **^{13}C NMR (101 MHz, $CDCl_3$)** δ (ppm) 140.6 (C), 140.5 (C), 128.9 (CH), 127.5 (CH), 126.9 (CH), 83.2 (O-CH), 82.7 (O-CH), 73.2 (HC-O), 73.2 (HC-O), 60.7 (HC-Cl), 60.4 (HC-Cl), 50.7 (CH), 50.5 (CH), 45.0 (CH_2), 21.7 (CH_3), 20.5 (CH_2), 20.4 (CH_2), 9.4 (CH_3), 9.0 (CH_3). **HRMS (ESI+)** m/z calc. for $C_{22}H_{32}Cl_2NaO_2$ ($[M+Na]^+$): 421.1672, found 421.1678. **Melting point** = 92.4 – 95.0 °C.

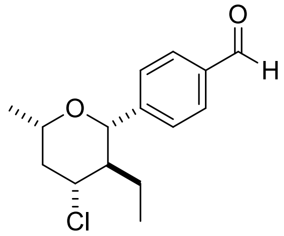 4-((2*S**,3*R**,4*R**,6*S**)-4-chloro-3-ethyl-6-methyltetrahydro-2*H*-pyran-2-yl)benzaldehyde (**4y**) was obtained following the general procedure, from cyclopropylsilyl alcohol **3a** (80 mg, 0.32 mmol) and terephthalaldehyde terephthalaldehyde in 20 minutes, to give, after column chromatography (hexane/EtOAc: 5:1), a yellow oil (34 mg, 40%). **^{1}H NMR (400 MHz, $CDCl_3$)** δ (ppm) 10.02 (s, 1H, CHO), 7.88 (d, *J* = 8.2 Hz, 2H, Ar-*H*), 7.53 (d, *J* = 8.2 Hz, 2H, Ar-*H*), 4.25 (d, *J* = 10.2 Hz, 1H, O-C*H*), 4.09 (td, *J* = 11.4, 4.5 Hz, 1H, *H*C-Cl), 3.70 – 3.60 (m, 1H, *H*C-O), 2.31 (ddd, *J* = 13.0, 4.6, 2.0 Hz, 1H, C*H*H), 1.94 – 1.75 (m, 2H), 1.67 – 1.56 (m, 1H, C*H*H-CH_3), 1.26 (d, *J* = 6.2 Hz, 3H, CH_3), 1.24 – 1.13 (m, 1H, CH*H*-CH_3), 0.70 (t, *J* = 7.6 Hz, 3H, CH_2-*CH_3*). **^{13}C NMR (101 MHz, $CDCl_3$)** δ (ppm) 192.1 (CHO), 146.3 (C), 136.4 (C), 130.6 (CH), 128.3 (CH), 82.6 (O-CH), 73.4 (HC-O), 60.1 (HC-Cl), 50.5 (CH), 44.8 (CH_2), 21.6 (CH_3), 20.3 (CH_2), 9.2 (CH_3). **HRMS (ESI+)** m/z calc. for $C_{15}H_{19}ClNaO_2$ ($[M+Na]^+$): 289.0966, found 289.0975.

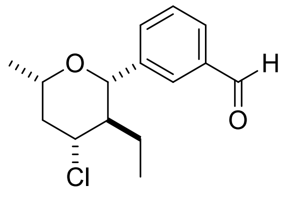 3-((2*S**,3*R**,4*R**,6*S**)-4-chloro-3-ethyl-6-methyltetrahydro-2*H*-pyran-2-yl)benzaldehyde (**4z**) was obtained following the general procedure, from cyclopropylsilyl alcohol **3a** (80 mg, 0.32 mmol) and isophthalaldehyde in 40 minutes, to give, after column chromatography (hexane/EtOAc: 5:1), a colourless oil (43 mg, 51%). **^{1}H NMR (500 MHz, $CDCl_3$)** δ (ppm) 10.03 (s, 1H, CHO), 7.88 (t, *J* = 1.6 Hz, 1H, Ar-*H*), 7.83 (dt, *J* = 7.6, 1.6 Hz, 1H, Ar-*H*), 7.63 (dt, *J* = 7.6, 1.6 Hz, 1H, Ar-*H*), 7.53 (t, *J* = 7.6, 1H, Ar-*H*), 4.26 (d, *J* = 10.3 Hz, 1H, O-C*H*), 4.10 (td, *J* = 11.4, 4.5 Hz, 1H, *H*C-Cl), 3.68 – 3.61 (m, 1H, *H*C-O), 2.31 (ddd, *J* = 13.0, 4.6, 1.9 Hz, 1H, C*H*H), 1.92 – 1.80 (m, 2H), 1.67 – 1.57 (m, 1H, C*H*H-CH_3), 1.26 (d, *J* = 6.3 Hz, 3H, CH_3), 1.23 – 1.14 (m, 1H, CH*H*-CH_3), 0.70 (t, *J* = 7.6 Hz, 3H, CH_3). **^{13}C NMR (101 MHz, $CDCl_3$)** δ (ppm) 192.3 (CHO), 141.7 (C), 136.8 (C), 133.7 (CH), 129.9 (CH), 129.4 (CH),

128.7 (CH), 82.5 (O-CH), 73.4 (HC-O), 60.0 (HC-Cl), 50.5 (CH), 44.8 (CH_2), 21.6 (CH_3), 20.4 (CH_2), 9.2 (CH_3). **HRMS (ESI+)** m/z calc. for $C_{15}H_{19}ClNaO_2$ ($[M+Na]^+$): 289.0966, found 289.0974.

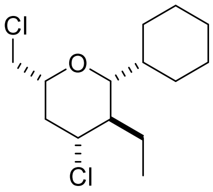

(2*R**,3*R**,4*R**,6*R**)-4-chloro-6-(chloromethyl)-2-cyclohexyl-3-ethyltetrahydro-2*H*-pyran (**4aa**) was obtained following the general procedure, from cyclopropylsilyl alcohol **3c** (80 mg, 0.28 mmol) and cyclohexanecarbaldehyde in 45 minutes, to give, after column chromatography (hexane/EtOAc: 60:1), a yellow oil (47 mg, 60). **^{1}H NMR (500 MHz, $CDCl_3$)** δ (ppm) 3.99 (td, *J* = 11.3, 4.6 Hz, 1H, *H*C-Cl), 3.52 (dd, *J* = 10.2, 5.4 Hz, 1H, *H*HC-Cl), 3.49 – 3.44 (m, 1H, *H*C-O), 3.42 (dd, *J* = 10.2, 5.0 Hz, 1H, H*H*C-Cl), 3.05 (dd, *J* = 10.3, 1.6 Hz, 1H, O-C*H*), 2.33 (ddd, *J* = 12.6, 4.6, 1.9 Hz, 1H), 1.82 – 1.72 (m, 3H), 1.71 – 1.62 (m, 3H), 1.61 – 1.56 (m, 1H), 1.55 – 1.49 (m, 3H), 1.47 – 1.40 (m, 1H), 1.31 – 1.20 (m, 2H), 1.21 – 1.13 (m, 2H), 0.86 (t, *J* = 7.6 Hz, 3H, CH_2-CH_3). **^{13}C NMR (101 MHz, $CDCl_3$)** δ (ppm) 83.3 (O-CH), 76.2 (HC-O), 60.7 (HC-Cl), 46.7 (CH_2), 45.4 (CH), 40.7 (CH_2), 38.9 (CH), 31.0 (CH_2), 27.0 (CH_2), 26.6 (2xCH_2), 25.1 (CH_2), 19.9 (CH_2), 8.9 (CH_3). **HRMS (ESI+)** m/z calc. for $C_{14}H_{24}Cl_2O$ ($[M+H]^+$): 279.1277, found 279.1273.

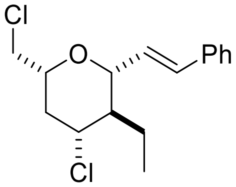

(2*R**,3*R**,4*R**,6*R**)-4-chloro-6-(chloromethyl)-3-ethyl-2-((*E*)-styryl)tetrahydro-2*H*-pyran (**4ab**) was obtained following the general procedure, from cyclopropylsilyl alcohols **3c** (80 mg, 0.28 mmol) and (*E*)-cinnamaldehyde in 40 minutes, to give, after column chromatography (hexane/EtOAc: 20:1), a yellow oil (58 mg, 70%). **^{1}H NMR (500 MHz, $CDCl_3$)** δ (ppm) 7.42 – 7.39 (m, 2H, Ar-*H*), 7.35 – 7.30 (m, 2H, Ar-*H*), 7.28 – 7.24 (m, 1H, Ar-*H*), 6.66 (d, *J* = 15.9 Hz, 1H, =C*H*), 6.16 (dd, *J* = 15.9, 7.9 Hz, 1H, *H*C=), 4.04 (td, *J* = 11.5, 4.6 Hz, 1H, *H*C-Cl), 3.89 (dd, *J* = 10.1, 7.9 Hz, 1H, O-C*H*), 3.70 – 3.64 (m, 1H, *H*C-O), 3.60 (dd, *J* = 11.2, 5.2 Hz, 1H, C*H*H-Cl), 3.51 (dd, *J* = 11.2, 5.8 Hz, 1H, CH*H*-Cl), 2.43 (ddd, *J* = 12.8, 4.6, 2.0 Hz, 1H, CH*H*), 1.91 – 1.81 (m, 1H), 1.81 – 1.73 (m, 1H), 1.73 – 1.52 (m, 2H), 0.90 (t, *J* = 7.5 Hz, 3H, CH_3). **^{13}C NMR (101 MHz, $CDCl_3$)** δ (ppm) 136.4 (C), 133.8 (=CH), 128.7 (CH), 128.2 (HC=), 127.3 (CH), 126.8 (CH), 81.5 (O-CH), 75.9 (HC-O), 59.3 (HC-Cl), 49.5 (CH), 46.3 (CH_2), 40.3 (CH_2), 20.5 (CH_2), 9.3 (CH_3). **HRMS (ESI+)** m/z calc. for $C_{16}H_{21}Cl_2O$ ($[M+H]^+$): 299.0964, found 299.0977.

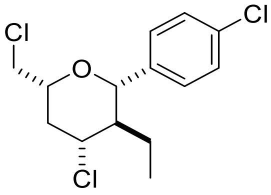

(2*S**,3*R**,4*R**,6*R**)-4-chloro-6-(chloromethyl)-2-(4-chlorophenyl)-3-ethyltetrahydro-2*H*-pyran (**4ac**) was obtained following the general procedure, from cyclopropylsilyl alcohols **3c** (80 mg, 0.28 mmol) and 4-chlorobenzaldehyde in 40 minutes, to give, after column chromatography (hexane/EtOAc: 15:1), a yellow oil (56 mg, 65%). **^{1}H NMR (500 MHz, $CDCl_3$)** δ (ppm) 7.34 (d, *J* = 8.5 Hz, 2H, Ar-*H*), 7.28 (d, *J* = 8.5 Hz, 2H, Ar-*H*), 4.19 (d, *J* = 10.3 Hz, 1H, O-C*H*), 4.09 (td, *J* = 11.4, 4.5 Hz, 1H, *H*C-Cl), 3.76 – 3.68 (m, 1H, *H*C-O), 3.58 (dd, *J* = 11.3, 5.0 Hz, 1H, *H*HC-Cl), 3.51 (dd, *J* = 11.3, 5.9 Hz, 1H, H*H*C-Cl), 2.48 (ddd, *J* = 12.8, 4.5, 2.0 Hz, 1H, C*H*H), 1.96 (dt, *J* = 12.8, 11.6 Hz, 1H, CH*H*), 1.85 – 1.77 (m, 1H, C*H*), 1.66 – 1.57 (m, 1H), 1.26 – 1.18 (m, 1H), 0.71 (t, *J* = 7.6 Hz, 3H, CH_3). **^{13}C NMR (101 MHz, $CDCl_3$)** δ (ppm) 138.3 (C), 134.3 (C), 128.9 (CH), 128.9 (CH), 82.5 (O-CH), 76.4 (HC-O), 59.3 (HC-

Cl), 50.5 (CH), 46.3 (CH_2), 40.3 (CH_2), 20.3 (CH_2), 9.1 (CH_3). **HRMS (ESI+)** m/z calc. for $C_{14}H_{18}Cl_3O$ ($[M+H]^+$): 307.0418, found 307.0423.

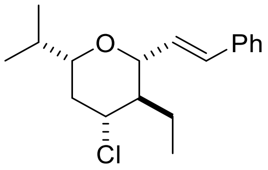

(2*R**,3*R**,4*R**,6*R**)-4-chloro-3-ethyl-6-isopropyl-2-((*E*)-styryl)tetrahydro-2*H*-pyran (**4ad**) was obtained following the general procedure, from cyclopropylsilyl alcohols **3d** (80 mg, 0.29 mmol) and (*E*)-cinnamaldehyde in 20 minutes, to give, after column chromatography (hexane/EtOAc: 40:1), a white solid (47 mg, 55%). **^{1}H NMR (500 MHz, $CDCl_3$)** δ (ppm) 7.42 – 7.38 (m, 2H, Ar-*H*), 7.35 – 7.30 (m, 2H, Ar-*H*), 7.27 – 7.22 (m, 1H, Ar-*H*), 6.63 (d, *J* = 15.9 Hz, 1H, =C*H*), 6.19 (dd, *J* = 15.9, 7.6 Hz, 1H, *H*C=), 4.03 (td, *J* = 11.3, 4.6 Hz, 1H, *H*C-Cl), 3.80 (dd, *J* = 9.7, 7.6 Hz, 1H, O-C*H*), 3.12 (ddd, *J* = 11.3, 6.2, 1.8 Hz, 1H, *H*C-O), 2.24 (ddd, *J* = 12.7, 4.6, 1.8 Hz, 1H, C*H*H), 1.84 – 1.70 (m, 3H), 1.67 – 1.56 (m, 2H), 0.96 (d, *J* = 6.8 Hz, 3H, CH_3), 0.93 (d, *J* = 6.8 Hz, 3H, CH_3), 0.90 (t, *J* = 7.5 Hz, 3H, CH_3). **^{13}C NMR (101 MHz, $CDCl_3$)** δ (ppm) 136.8 (C), 132.7 (=CH), 128.7 (CH), 128.4 (HC=), 127.9 (CH), 126.8 (CH), 81.4 (HC-O), 81.1 (O-CH), 61.1 (HC-Cl), 50.1 (CH), 39.7 (CH_2), 32.9 (CH), 20.6 (CH_2), 18.9 (CH_3), 18.2 (CH_3), 9.4 (CH_3). **HRMS (ESI+)** m/z calc. for $C_{18}H_{26}ClO$ ($[M+H]^+$): 293.1667, found 293.1679. **Melting point** = 63.2 – 65.1 °C.

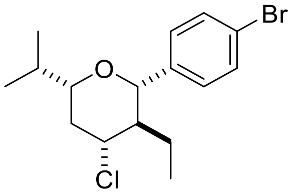

(2*S**,3*R**,4*R**,6*R**)-2-(4-bromophenyl)-4-chloro-3-ethyl-6-isopropyltetrahydro-2*H*-pyran (**4ae**) was obtained following the general procedure, from cyclopropylsilyl alcohol **3d** (80 mg, 0.29 mmol) and 4-bromobenzaldehyde in 20 minutes, to give, after column chromatography (hexane/EtOAc: 20:1), a colourless oil (74 mg, 74%).**^{1}H NMR (500 MHz, $CDCl_3$)** δ (ppm) 7.48 (d, *J* = 8.4 Hz, 2H, Ar-*H*), 7.22 (d, *J* = 8.4 Hz, 2H, Ar-*H*), 4.11 (d, *J* = 10.3 Hz, 1H, O-C*H*), 4.07 (td, *J* = 11.4, 4.6 Hz, 1H, *H*C-Cl), 3.21 (ddd, *J* = 11.4, 5.6, 1.9 Hz, 1H, *H*C-O), 2.26 (ddd, *J* = 12.7, 4.6, 1.9 Hz, 1H, C*H*H), 1.88 – 1.70 (m, 3H), 1.65 – 1.55 (m, 1H, C*H*H-CH_3), 1.25 – 1.16 (m, 1H, CH*H*-CH_3), 0.92 (d, *J* = 6.8 Hz, 3H, CH_3), 0.91 (d, *J* = 6.8 Hz, 3H, CH_3), 0.71 (t, *J* = 7.6 Hz, 3H, CH_2-*CH_3*). **^{13}C NMR (101 MHz, $CDCl_3$)** δ (ppm) 139.9 (C), 131.6 (C), 129.2 (CH), 122.0 (CH), 82.2 (O-CH), 81.7 (HC-O), 61.1 (HC-Cl), 51.1 (CH), 39.4 (CH_2), 32.7 (CH), 20.4 (CH_2), 18.5 (CH_3). 18.1 (CH_3). 9.2 (CH_3). **HRMS (ESI+)** m/z calc. for $C_{16}H_{23}BrClO$ ($[M+H]^+$): 345.0615, found 345.0622.

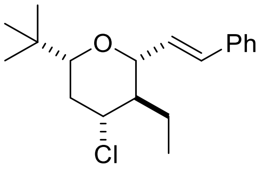

2*R**,3*R**,4*R**,6*R**)-6-(*tert*-butyl)-4-chloro-3-ethyl-6-methyl-2-((*E*)-styryl)tetrahydro-2*H*-pyran (**4af**) was obtained following the general procedure, from cyclopropylsilyl alcohols **3e** (80 mg, 0.27 mmol) and (*E*)-cinnamaldehyde in 20 minutes, to give, after column chromatography (hexane/EtOAc: 30:1), a white solid (68 mg, 80%). **^{1}H NMR (500 MHz, $CDCl_3$)** δ (ppm) 7.43 – 7.39 (m, 2H, Ar-*H*), 7.35 – 7.20 (m, 2H, Ar-*H*), 7.27 – 7.22 (m, 1H, Ar-*H*), 6.62 (d, *J* = 15.9 Hz, 1H, =C*H*), 6.20 (dd, *J* = 15.9, 7.2 Hz, 1H, *H*C=), 4.03 (td, *J* = 11.3, 4.6 Hz, 1H, *H*C-Cl), 3.78 (dd, *J* = 9.8, 7.2 Hz, 1H, O-C*H*), 3.00 (dd, *J* = 11.5, 1.6 Hz, 1H, *H*C-O), 2.23 (ddd, *J* = 12.5, 4.5, 1.6 Hz, 1H, C*H*H), 1.82 – 1.68 (m, 2H), 1.66 – 1.54 (m, 2H), 0.93 (s, 9H, CH_3), 0.90 (t, *J* = 7.5 Hz, 3H, CH_3). **^{13}C NMR (101 MHz, $CDCl_3$)** δ (ppm) 137.0 (C), 132.0 (=CH), 128.7 (CH), 128.7 (HC=), 127.8 (CH), 126.7 (CH), 84.1 (HC-O), 80.8 (O-CH), 61.8 (HC-Cl),

50.2 (CH), 37.8 (CH_2), 34.4 (C), 26.2 (CH_3), 20.6 (CH_2), 9.5 (CH_3). **HRMS (ESI+)** m/z calc. for $C_{19}H_{28}ClO$ ($[M+H]^+$): 307.1823, found 307.1830. **Melting point** = 70.4 – 72.7 °C.

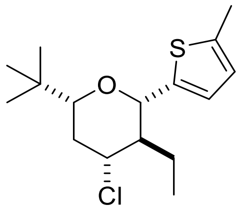

(2*S**,3*R**,4*R**,6*R**)-6-(*tert*-butyl)-4-chloro-3-ethyl-6-methyl-2-(5-methylthiophen-2-yl)tetrahydro-2*H*-pyran (**4ag**) was obtained following the general procedure, from cyclopropylsilyl alcohols **3e** (80 mg, 0.27 mmol) and 5-methylthiophene-2-carbaldehyde in 30 minutes, to give, after column chromatography (hexane/EtOAc: 45:1), a white solid (37 mg, 45%). **^{1}H NMR (500 MHz, $CDCl_3$)** δ (ppm) 6.74 (d, *J* = 3.5 Hz, 1H, =C*H*), 6.58 (dq, *J* = 3.5, 1.0 Hz, 1H, *H*C=), 4.34 (d, *J* = 9.9 Hz, 1H, O-C*H*), 4.05 (td, *J* = 11.3, 4.6 Hz, 1H, *H*C-Cl), 3.04 (dd, *J* = 11.5, 1.8 Hz, 1H, *H*C-O), 2.46 (d, *J* = 1.0 Hz, 3H, CH_3), 2.24 (ddd, *J* = 12.4, 4.4, 1.8 Hz, 1H, C*H*H), 1.86 – 1.76 (m, 1H, CH*H*), 1.76 – 1.64 (m, 2H), 1.42 – 1.32 (m, 1H), 0.90 (s, 9H, CH_3), 0.77 (t, *J* = 7.5 Hz, 3H, CH_3). **^{13}C NMR (101 MHz, $CDCl_3$)** δ (ppm) 142.0 (C), 139.5 (C), 124.8 (CH), 128.2 (CH), 84.4 (HC-O), 78.4 (O-CH), 61.4 (HC-Cl), 51.9 (CH), 37.7 (CH_2), 34.3 (C), 26.1 (CH_3), 20.8 (CH_2), 15.6 (CH_3), 9.5 (CH_3). **HRMS (ESI+)** m/z calc. for $C_{16}H_{26}ClOS$ ($[M+H]^+$): 301.1387, found 301.1400. **Melting point** = 53.3 – 55.2 °C.

2.3. <u>TMSCl/$BiCl_3$-promoted cyclization of cyclopropylsilyl alcohols **3f**</u>

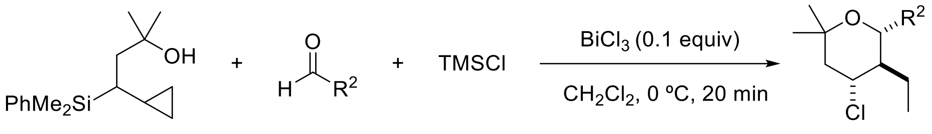

3f (1 equiv) (1.2 equiv) (1.2 equiv) **7**

A solution of $BiCl_3$ (9.5 mg, 0.030 mmol, 0.1 equiv) and the corresponding aldehyde (0.37 mmol, 1.2 equiv) in 5 mL of dry dichloromethane (0.05 M) was cooled to 0 °C (under nitrogen). Then, 80 mg of the alcohol **3f** (0.30 mmol, 1.0 equiv) was dissolved in 1 mL of dry dichloromethane and added into the reaction. Finally, 0.046 mL of the Lewis acid, TMSCl (0.37 mmol, 1.2 equiv) was added dropwise. The mixture was stirred at 0 ºC for 20 min while monitored by TLC. When starting materials were consumed, it was hydrolyzed with 5 mL of $NaHCO_3$ (sat). Phases are then separated, extracting the aqueous phase three times with dichloromethane (3 x 10 mL). The organic phases are combined, washed with NaCl sat. (20 mL) and dried over anhydrous Na_2SO_4. The solvent is then evaporated under reduced pressure. The crude mixture is analyzed by NMR and then purified by column chromatography in silica gel, using mixtures of hexane-ethyl acetate and yielding tetrahydropyrans **7**.

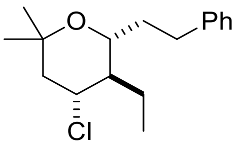

(4*R**,5*R**,6*R**)-4-chloro-5-ethyl-2,2-dimethyl-6-phenethyltetrahydro-2*H*-pyran (**7a**) was obtained following the general procedure, from cyclopropylsilyl alcohol **3f** (80 mg, 0.30 mmol) and 3-phenylpropanal in 30 minutes, to give, after column chromatography (hexane/EtOAc: 30:1), a yellow oil (73 mg, 85%). **^{1}H NMR (500 MHz, $CDCl_3$)** δ (ppm) 7.30 – 7.25 (m, 2H, Ar-*H*), 7.22 – 7.14 (m, 3H, Ar-*H*), 4.09 (td, *J* = 11.3, 4.6 Hz, 1H, *H*C-Cl), 3.35 (td, *J* = 9.9, 2.5 Hz, 1H, O-C*H*), 2.83 (ddd, *J* = 13.8, 9.2, 4.7 Hz, 1H, C*H*H-Ph), 2.67 (ddd, *J* = 13.8, 8.7, 7.6 Hz, 1H, CH*H*-Ph), 2.09 (dd, *J* = 12.8, 4.6 Hz, 1H, C*H*H), 1.99 – 1.90 (m, 1H), 1.81 (t, *J* = 12.8 Hz, 1H, CH*H*), 1.77 – 1.64 (m, 2H), 1.60 – 1.49 (m, 1H, CH*H*-CH_3), 1.47 – 1.38 (m, 1H, CH), 1.26 (s, 3H, CH_3), 1.14 (s, 3H, CH_3), 0.80 (t, *J* = 7.6 Hz, 3H, CH_3). **^{13}C NMR (101 MHz, $CDCl_3$)** δ (ppm) 142.5 (C), 128.8 (CH), 128.4 (CH), 125.8 (CH), 72.6 (O-CH), 71.3 (HC-O), 59.5 (HC-

Cl), 49.7 (CH), 47.9 (CH_2), 35.1 (CH_2), 31.7 (CH_2), 31.6 (CH_3), 22.6 (CH_3), 20.5 (CH_2), 9.2 (CH_3). **HRMS (ESI+)** m/z calc. for $C_{17}H_{25}ClNaO$ ($[M+Na]^+$): 303.1492, found 303.1491.

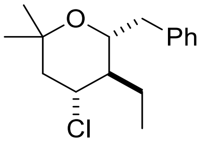

(4*R**,5*R**,6*R**)-4-chloro-5-ethyl-2,2-dimethyl-tetrahydro-2*H*-pyran (**7b**) was obtained following the general procedure, from cyclopropylsilyl alcohol **3f** (80 mg, 0.30 mmol) and phenylacetaldehyde in 20 minutes, to give, after column chromatography (hexane/EtOAc: 30:1), a pale yellow solid (45 mg, 56%). **^{1}H NMR (500 MHz, $CDCl_3$)** δ (ppm) 7.28 – 7.25 (m, 4H, Ar-*H*), 7.22 – 7.17 (m, 1H, Ar-*H*), 4.17 (ddd, *J* = 12.2, 11.3, 4.6 Hz, 1H, *H*C-Cl), 3.69 (ddd, *J* = 10.4, 8.0, 2.6 Hz, 1H, O-C*H*), 2.98 (dd, *J* = 14.4, 2.6 Hz, 1H, C*H*H-Ph), 2.67 (dd, *J* = 14.4, 8.0 Hz, 1H, CH*H*-Ph), 2.07 (dd, *J* = 12.7, 4.6, Hz, 1H, C*H*H), 1.93 – 1.83 (m, 1H, C*H*H-CH_3), 1.80 (t, *J* = 12.7 Hz, 1H, CH*H*), 1.73 – 1.63 (m, 1H, CH*H*-CH_3), 1.50 – 1.43 (m, 1H, CH), 1.20 (s, 3H, CH_3), 1.09 (s, 3H, CH_3), 0.95 (t, *J* = 7.6 Hz, 3H, CH_2-*CH_3*). **^{13}C NMR (101 MHz, $CDCl_3$)** δ (ppm) 139.4 (C), 129.7 (CH), 128.0 (CH), 126.1 (CH), 73.2 (O-CH), 72.6 (C-O), 59.3 (HC-Cl), 48.9 (CH), 47.7 (CH_2), 39.6 (CH_2), 31.4 (CH_3), 22.4 (CH_3), 20.5 (CH_2), 9.1 (CH_3). **HRMS (ESI+)** m/z calc. for $C_{16}H_{23}ClNaO$ ($[M+Na]^+$): 289.1330, found 289.1336. **Melting point** = 39.2 – 40.8 °C.

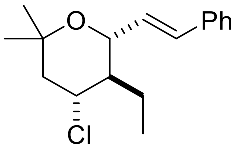

(4*R**,5*R**,6*R**)-4-chloro-5-ethyl-2,2-dimethyl-6-((*E*)-styryl)tetrahydro-2*H*-pyran (**7c**) was obtained following the general procedure, from cyclopropylsilyl alcohol **3f** (80 mg, 0.30 mmol) and (*E*)-cinnamaldehyde in 20 minutes, to give, after column chromatography (hexane/EtOAc: 25:1), a yellow oil (53 mg, 63%). **^{1}H NMR (500 MHz, $CDCl_3$)** δ 7.41 – 7.38 (m, 2H, Ar-*H*), 7.33 – 7.28 (m, 2H, Ar-*H*), 7.26 – 7.21 (m, 1H, Ar-*H*), 6.62 (d, *J* = 15.9 Hz, 1H, =C*H*), 6.13 (dd, *J* = 15.9, 8.0 Hz, 1H, *H*C=), 4.20 (ddd, *J* = 12.2, 11.0, 4.5 Hz, 1H, *H*C-Cl), 4.06 (ddd, *J* = 10.3, 7.9, 0.8 Hz, 1H, O-C*H*), 2.14 (dd, *J* = 12.9, 4.5 Hz, 1H, C*H*H), 1.81 – 1.71 (m, 1H, CH*H*-CH_3), 1.66 – 1.55 (m, 2H), 1.90 (dd, *J* = 12.9, 12.2 Hz, 1H, CH*H*), 1.30 (s, 3H, CH_3), 1.28 (s, 3H, CH_3), 0.91 (t, *J* = 7.5 Hz, 3H, CH_3). **^{13}C NMR (101 MHz, $CDCl_3$)** δ 136.7 (C), 133.3 (=CH), 128.8 (HC=), 128.6 (CH), 128.0 (CH), 126.8 (CH), 75.3 (O-CH), 73.3 (C-O), 58.6 (HC-Cl), 50.0 (CH), 47.7 (CH_2), 31.5 (CH_3), 22.7 (CH_3), 20.6 (CH_2), 9.4 (CH_3). **HRMS (ESI+)** m/z calc. for $C_{17}H_{23}ClNaO$ ($[M+Na]^+$): 301.1335, found 301.1339.

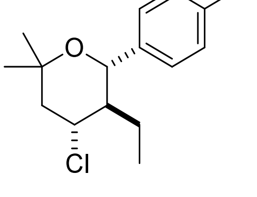

(4*R**,5*R**,6*S**)-4-chloro-6-(4-chlorophenyl)-5-ethyl-2,2-dimethyltetrahydro-2*H*-pyran (**7d**) was obtained following the general procedure, from cyclopropylsilyl alcohol **3f** (80 mg, 0.30 mmol) and 4-chlorobenzaldehyde in 20 minutes, to give, after column chromatography (hexane/EtOAc: 20:1), a pale yellow solid (34 mg, 40%). **^{1}H NMR (500 MHz, $CDCl_3$)** δ (ppm) 7.32 (d, *J* = 8.4 Hz, 2H, Ar-*H*), 7.29 (d, *J* = 8.4 Hz, 2H, Ar-*H*), 4.38 (d, *J* = 10.4 Hz, 1H, O-C*H*), 4.25 (ddd, *J* = 12.2, 11.1, 4.4 Hz, 1H, *H*C-Cl), 2.20 (dd, *J* = 12.9, 4.5 Hz, C*H*H), 1.98 (t, *J* = 12.9 Hz, 1H, CH*H*), 1.75 – 1.67 (m, 1H, C*H*), 1.65 – 1.56 (m, 1H, C*H*H-CH_3), 1.30 (s, 6H, $(CH_3)_2$), 1.25 – 1.16 (m, 1H, CH*H*-CH_3), 0.73 (t, *J* = 7.6 Hz, 3H, CH_3). **^{13}C NMR (101 MHz, $CDCl_3$)** δ (ppm) 139.5 (C), 134.0 (C), 129.1 (CH), 128.8 (CH), 76.2 (O-CH), 73.7 (C-O), 58.6 (HC-Cl), 51.2 (CH), 47.8 (CH_2), 31.5 (CH_3), 22.6 (CH_3), 20.4 (CH_2), 9.3 (CH_3). **HRMS (ESI+)** m/z calc. for $C_{15}H_{20}Cl_2NaO$ ($[M+Na]^+$): 309.0783, found 309.0786. **Melting point** = 52.6 – 54.0 °C.

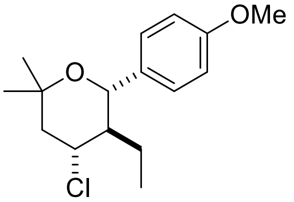

(4*R**,5*R**,6*S**)-4-chloro-5-ethyl-6-(4-methoxyphenyl)-2,2-dimethyltetrahydro-2*H*-pyran (**7e**) was obtained following the general procedure, from cyclopropylsilyl alcohol **3f** (80 mg, 0.30 mmol) and 4-methoxybenzaldehyde in 20 minutes, to give, after column chromatography (hexane/EtOAc: 10:1), a viscous colourless oil (51 mg, 60%). **^{1}H NMR (500 MHz, $CDCl_3$)** δ (ppm) 7.27 (d, *J* = 8.7 Hz, 2H, Ar-*H*), 6.87 (d, *J* = 8.7 Hz, 2H, Ar-*H*), 4.38 (d, *J* = 10.5 Hz, 1H, O-C*H*), 4.25 (dt, *J* = 11.4, 4.4 Hz, 1H, *H*C-Cl), 3.80 (s, 3H, O-CH_3), 2.10 (dd, *J* = 12.9, 4.4 Hz, 1H, C*H*H), 1.98 (t, *J* = 12.9 Hz, 1H, CH*H*), 1.79 – 1.70 (m, 1H, C*H*), 1.66 – 1.54 (m, 1H, C*H*H-CH_3), 1.30 (s, 3H, CH_3), 1.29 (s, 3H, CH_3), 1.28 – 1.18 (m, 1H, CH*H*-CH_3), 0.73 (t, *J* = 7.6 Hz, 3H, CH_3). **^{13}C NMR (101 MHz, $CDCl_3$)** δ (ppm) 159.6 (C), 133.2 (C), 128.8 (CH), 114.0 (CH), 76.4 (O-CH), 73.4 (C-O), 59.1 (HC-Cl), 55.5 (OCH_3), 51.2 (CH), 48.0 (CH_2), 31.6 (CH_3), 22.6 (CH_3), 20.6 (CH_2), 9.3 (CH_3). HRMS (ESI+) m/z calc. for $C_{16}H_{23}ClNaO_2$ ($[M+Na]^+$): 305.1284, found 305.1288.

2.4. TMSBr/$BiCl_3$-promoted cyclization of cyclopropylsilyl alcohol **3a**

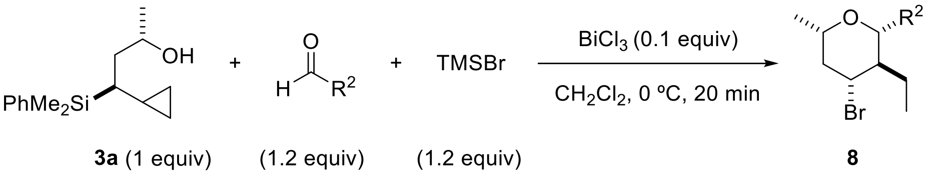

A solution of $BiCl_3$ (10.1 mg, 0.032 mmol, 0.1 equiv) and the corresponding aldehyde (0.39 mmol, 1.2 equiv) in 5 mL of dry dichloromethane (0.05 M) was cooled to 0 °C (under nitrogen). Then, alcohol **3a** (80 mg, 0.32 mmol, 1.0 equiv) was dissolved in 1 mL of dry dichloromethane and added into the reaction. Finally, the Lewis acid, TMSBr (0.051 mL, 0.39 mmol, 1.2 equiv), was added dropwise. The mixture was stirred at 0 ºC while monitored by TLC. When starting materials were consumed, it was hydrolyzed with 5 mL of $NaHCO_3$ (sat). Phases are then separated, extracting the aqueous phase three times with dichloromethane (3 x 10 mL). The organic phases are combined, washed with NaCl sat. (20 mL) and dried over anhydrous Na_2SO_4. The solvent is then evaporated under reduced pressure. The crude mixture is analyzed by NMR and then purified by column chromatography in silica gel, using mixtures of hexane-ethyl acetate and yielding tetrahydropyrans **8**.

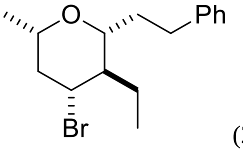

(2*R**,3*R**,4*R**,6*S**)-4-bromo-3-ethyl-6-methyl-2-phenethyltetrahydro-2*H*-pyran (**8a**) was obtained following the general procedure, from cyclopropylsilyl alcohols **3a** (80 mg, 0.32 mmol) and 3-phenylpropanal in 10 minutes, to give, after column chromatography (hexane/EtOAc: 25:1), a colourless oil (80 mg, 81%). **^{1}H NMR (500 MHz, $CDCl_3$)** δ (ppm) 7.28 – 7.24 (m, 2H, Ar-*H*), 7.21 – 7.12 (m, 3H, Ar-*H*), 4.08 (td, *J* = 11.5, 4.7 Hz, 1H, *H*C-Br), 3.41 – 3.31 (m, 1H, *H*C-O), 3.10 (td, *J* = 9.6, 2.6 Hz, 1H, O-C*H*), 2.83 (ddd, *J* = 13.9, 9.4, 4.6 Hz, 1H, C*H*H-Ph), 2.65 (ddd, *J* = 13.9, 9.1, 7.6 Hz, 1H, CH*H*-Ph), 2.29 (ddd, *J* = 12.8, 4.7, 2.0 Hz, 1H, C*H*H), 2.00 – 1.82 (m, 2H), 1.80 – 1.66 (m, 2H), 1.66 – 1.48 (m, 2H), 1.21 (d, *J* = 6.2 Hz, 3H, CH_3), 0.75 (t, *J* = 7.5 Hz, 3H, CH_2-C*H*$_3$). **^{13}C NMR (101 MHz, $CDCl_3$)** δ (ppm) 142.43 (C), 128.7 (CH), 128.5 (CH), 125.9 (CH), 78.4 (O-CH), 73.6 (HC-O), 54.4 (HC-Br), 49.4 (CH), 46.3 (CH_2), 35.1 (CH_2), 31.7 (CH_2), 21.9 (CH_2), 21.5 (CH_3), 8.9 (CH_3). **HRMS (ESI+)** m/z calc. for $C_{16}H_{24}BrO$ ($[M+H]^+$): 311.1005, found 311.1007.

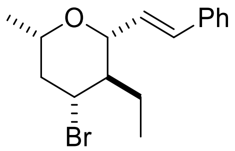

($2R^*,3R^*,4R^*,6S^*$)-4-bromo-3-ethyl-6-methyl-2-((*E*)-styryl)tetrahydro-2*H*-pyran (**8b**) was obtained following the general procedure, from cyclopropylsilyl alcohols **3a** (80 mg, 0.32 mmol and 300 mg, 1.21 mmol) and (*E*)-cinnamaldehyde in 10 minutes, to give, after column chromatography (hexane/EtOAc: 20:1), a yellow oil (70 mg, 71% and 235 mg, 63%). **^{1}H NMR (500 MHz, $CDCl_3$)** δ (ppm) 7.42 – 7.37 (m, 2H, Ar-*H*), 7.34 – 7.29 (m, 2H, Ar-*H*), 7.27 – 7.21 (m, 1H, Ar-*H*), 6.64 (d, *J* = 15.9 Hz, 1H, =C*H*), 6.17 (dd, *J* = 15.9, 8.0 Hz, 1H, *H*C=), 4.18 (td, *J* = 11.4, 4.6 Hz, 1H, *H*C-Br), 3.86 (dd, *J* = 9.3, 8.0 Hz, 1H, O-C*H*), 3.60 – 3.50 (m, 1H, *H*C-O), 2.36 (ddd, *J* = 13.0, 4.6, 2.0 Hz, 1H, C*H*H), 1.98 (dt, *J* = 13.0, 11.4 Hz, 1H, CH*H*), 1.82 – 1.69 (m, 2H), 1.70 – 1.57 (m, 1H), 1.25 (d, *J* = 6.2 Hz, 3H, CH_3), 0.88 (t, *J* = 7.4 Hz, 3H, CH_3). **^{13}C NMR (101 MHz, $CDCl_3$)** δ (ppm) 136.6 (C), 133.4 (=CH), 128.7 (CH), 128.1 (HC=), 128.0 (CH), 126.8 (CH), 81.6 (O-CH), 73.5 (HC-O), 53.2 (HC-Br), 49.7 (CH), 46.0 (CH_2), 22.1 (CH_2), 21.5 (CH_3), 9.0 (CH_3). **HRMS (ESI+)** m/z calc. for $C_{16}H_{22}BrO$ ($[M+H]^+$): 309.0849, found 309.0847.

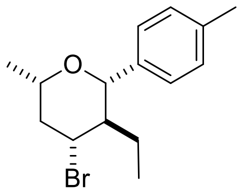

($2S^*,3R^*,4R^*,6S^*$)-4-bromo-3-ethyl-6-methyl-2-(*p*-tolyl)tetrahydro-2*H*-pyran (**8c**) was obtained following the general procedure, from cyclopropylsilyl alcohol **3a** (80 mg, 0.32 mmol) and 4-methylbenzaldehyde in 15 minutes, to give, after column chromatography (hexane/EtOAc: 25:1), a white solid (67 mg, 71%). **^{1}H NMR (500 MHz, $CDCl_3$)** δ (ppm) 7.22 (d, *J* = 8.1 Hz, 2H, Ar-*H*), 7.15 (d, *J* = 8.1 Hz, 2H, Ar-*H*), 4.25 (td, *J* = 11.6, 4.6 Hz, 1H, *H*C-Br), 4.14 (d, *J* = 10.1 Hz, 1H, O-C*H*), 3.64 – 3.54 (m, 1H, *H*C-O), 2.40 (ddd, *J* = 12.9, 4.6, 1.9 Hz, 1H, C*H*H), 2.33 (s, 3H, C*H*$_3$), 2.11 – 2.01 (m, 1H, CH*H*), 2.01 – 1.92 (m, 1H, CH), 1.67 – 1.55 (m, 1H, C*H*H-CH_3), 1.27 – 1.15 (m, 1H, CH*H*-CH_3), 1.23 (d, *J* = 6.2 Hz, 3H, CH_3), 0.68 (t, *J* = 7.6 Hz, 3H, CH_3). **^{13}C NMR (101 MHz, $CDCl_3$)** δ (ppm) 138.0 (C), 137.5 (C), 129.3 (CH), 127.4 (CH), 83.2 (O-CH), 74.0 (HC-O), 53.8 (HC-Br), 50.5 (CH), 46.2 (CH_2), 22.0 (CH_2), 21.6 (CH_3), 21.3 (CH_3), 8.8 (CH_3). **HRMS (ESI+)** m/z calc. for $C_{15}H_{22}BrO$ ($[M+H]^+$): 297.0849, found 297.0853. **Melting point** = 36.3 – 38.0 °C.

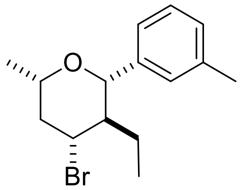

($2S^*,3R^*,4R^*,6S^*$)-4-bromo-3-ethyl-6-methyl-2-(*m*-tolyl)tetrahydro-2*H*-pyran (**8d**) was obtained following the general procedure, from cyclopropylsilyl alcohol **3a** (80 mg, 0.32 mmol) and 3-methylbenzaldehyde in 15 minutes, to give, after column chromatography (hexane/EtOAc: 20:1), a yellow oil (74 mg, 78%). **^{1}H NMR (500 MHz, $CDCl_3$)** δ (ppm) 7.23 (t, *J* = 7.5 Hz, 1H, Ar-*H*), 7.16 (s, 1H, Ar-*H*), 7.14 – 7.09 (m, 2H, Ar-*H*), 4.26 (td, *J* = 11.6, 4.6 Hz, 1H, *H*C-Br), 4.15 (d, *J* = 10.1 Hz, 1H, O-C*H*), 3.65 – 3.55 (m, 1H, *H*C-O), 2.41 (ddd, *J* = 12.9, 4.6, 1.9 Hz, 1H, C*H*H), 2.36 (s, 3H, C*H*$_3$), 2.13 – 2.03 (m, 1H, CH*H*), 2.02 – 1.94 (m, 1H, CH), 1.68 – 1.55 (m, 1H, C*H*H-CH_3), 1.28 – 1.17 (m, 1H, CH*H*-CH_3), 1.25 (d, *J* = 6.2 Hz, 3H, CH_3), 0.69 (t, *J* = 7.6 Hz, 3H, CH_3). **^{13}C NMR (101 MHz, $CDCl_3$)** δ (ppm) 140.3 (C), 138.3 (C), 129.1 (CH), 128.5 (CH), 128.1 (CH), 124.8 (CH), 83.5 (O-CH), 74.1 (HC-O), 53.7 (HC-Br), 50.4 (CH), 46.2 (CH_2), 21.9 (CH_2), 21.6 (CH_3), 21.6 (CH_3), 8.8 (CH_3). **HRMS (ESI+)** m/z calc. for $C_{15}H_{22}BrO$ ($[M+H]^+$): 297.0849, found 297.0853.

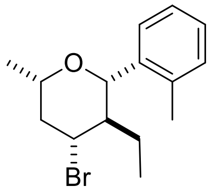

(2*S**,3*R**,4*R**,6*S**)-4-bromo-3-ethyl-6-methyl-2-(*o*-tolyl)tetrahydro-2*H*-pyran (**8e**) was obtained following the general procedure, from cyclopropylsilyl alcohol **3a** (80 mg, 0.32 mmol) and 2-methylbenzaldehyde in 15 minutes, to give, after column chromatography (hexane/EtOAc: 25:1), a pale yellow solid (74 mg, 78%). **^{1}H NMR (400 MHz, $CDCl_3$)** δ (ppm) 7.43 – 7.30 (m, 1H, Ar-*H*), 7.24 -7.11 (m, 3H, Ar-*H*), 4.44 (br s, 1H, O-C*H*), 4.25 (td, *J* = 11.5, 4.5 Hz, 1H, *H*C-Br), 3.66 – 3.54 (m, 1H, *H*C-O), 2.41 (ddd, *J* = 12.8, 4.6, 1.9 Hz, 1H, C*H*H), 2.40 (s, 3H, C*H_3*), 2.21 – 2.12 (m, 1H, C*H*), 2.07 (dt, *J* = 12.8, 11.5 Hz, 1H, CH*H*), 1.64 – 1.49 (m, 1H, C*H*H-CH_3), 1.35 – 1.24 (m, 1H, CH*H*-CH_3), 1.23 (d, *J* = 6.2 Hz, 3H, CH_3), 0.68 (t, *J* = 7.6 Hz, 3H, CH_3). **^{13}C NMR (101 MHz, $CDCl_3$)** δ (ppm) 138.3 (C), 136.1 (C), 130.8 (CH), 128.0 (CH), 127.4 (CH), 126.5 (CH), 75.7 (O-CH), 74.2 (HC-O), 54.1 (HC-Br), 49.7 (CH), 46.2 (CH_2), 22.1 (CH_2), 21.6 (CH_3), 19.8 (CH_3), 9.6 (CH_3). **HRMS (ESI+)** m/z calc. for $C_{15}H_{22}BrO$ ($[M+H]^+$): 297.0849, found 297.0845. **Melting point** = 35.4 – 37.1 °C.

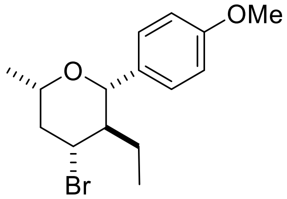

(2*S**,3*R**,4*R**,6*S**)-4-bromo-3-ethyl-2-(4-methoxyphenyl)-6-methyltetrahydro-2*H*-pyran (**8f**) was obtained following the general procedure, from cyclopropylsilyl alcohol **3a** (80 mg, 0.32 mmol) and 4-methoxybenzaldehyde in 20 minutes, to give, after column chromatography (hexane/EtOAc: 10:1), a white solid (83 mg, 83%). **^{1}H NMR (500 MHz, $CDCl_3$)** δ (ppm) 7.26 (d, *J* = 8.7 Hz, 2H, Ar-*H*), 6.88 (d, *J* = 8.7 Hz, 2H, Ar-*H*), 4.25 (td, *J* = 11.7, 4.6 Hz, 1H, *H*C-Br), 4.13 (d, *J* = 10.1 Hz, 1H, O-C*H*), 3.80 (s, 3H, O-C*H_3*), 3.64 – 3.55 (m, 1H, *H*C-O), 2.40 (ddd, *J* = 12.9, 4.5, 1.9 Hz, 1H, C*H*H), 2.10 – 2.02 (m, 1H, CH*H*), 1.98 – 1.91 (m, 1H, CH), 1.65 – 1.56 (m, 1H, C*H*H-CH_3), 1.28 – 1.18 (m, 1H, CH*H*-CH_3), 1.23 (d, *J* = 6.2 Hz, 3H, C*H_3*), 0.68 (t, *J* = 7.6 Hz, 3H, CH_3). **^{13}C NMR (101 MHz, $CDCl_3$)** δ (ppm) 159.6 (C), 132.8 (C), 128.7 (CH), 114.0 (CH), 82.9 (O-CH), 71.0 (HC-O), 55.4 (O-CH_3), 53.8 (HC-Br), 50.7 (CH), 46.2 (CH_2), 22.0 (CH_2), 21.6 (CH_3), 8.9 (CH_3). **HRMS (ESI+)** m/z calc. for $C_{15}H_{22}BrO_2$ ($[M+H]^+$): 313.0798, found 313.0794. **Melting point** = 79.3 – 81.9 °C.

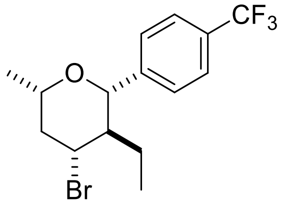

(2*S**,3*R**,4*R**,6*S**)-4-bromo-3-ethyl-6-methyl-2-(4-(trifluoromethyl)phenyl)-tetrahydro-2*H*-pyran (**8g**) was obtained following the general procedure, from cyclopropylsilyl alcohol **3a** (80 mg, 0.32 mmol) and 4-trifluoromethylbenzaldehyde in 15 minutes, to give, after column chromatography (hexane/EtOAc: 20:1), a colourless oil (79 mg, 70%). **^{1}H NMR (500 MHz, $CDCl_3$)** δ (ppm) 7.62 (d, *J* = 8.0 Hz, 2H, Ar-*H*), 7.47 (d, *J* = 8.0 Hz, 2H Ar-*H*), 4.25 (td, *J* = 11.4, 4.6 Hz, 1H, *H*C-Br), 4.25 (d, *J* = 10.1 Hz, 1H, O-C*H*), 3.66 – 3.59 (m, 1H, *H*C-O), 2.43 (ddd, *J* = 13.0, 4.6, 2.0 Hz, 1H, C*H*H), 2.08 (dt, *J* = 13.0, 11.4, 1H, CH*H*), 1.98 – 1.90 (m, 1H, C*H*), 1.69 – 1.59 (m, 1H, C*H*H-CH_3), 1.25 (d, *J* = 6.2 Hz, 3H, CH_3), 1.23 – 1.14 (m, 1H, CH*H*-CH_3), 0.70 (t, *J* = 7.6 Hz, 3H, CH_2-*CH_3*). **^{13}C NMR (101 MHz, $CDCl_3$)** δ (ppm) 144.4 (C), 130.6 (q, $^2J_{C\text{-}F}$ = 32.4 Hz, C), 127.9 (CH), 125.7 (q, $^4J_{C\text{-}F}$ = 3.8 Hz, CH), 124.2 (q, $^1J_{C\text{-}F}$ = 272.2 Hz, C), 82.6 (O-CH), 74.2 (HC-O), 52.8 (HC-Br), 50.6 (CH), 46.0 (CH_2), 21.7 (CH_3), 21.5 (CH_2), 8.8 (CH_3). **HRMS (ESI+)** m/z calc. for $C_{15}H_{18}BrF_3NaO$ ($[M+Na]^+$): 373.0385, found 373.0385.

2.5. TMSI/$BiCl_3$-promoted cyclization of cyclopropylsilyl alcohol **3a**

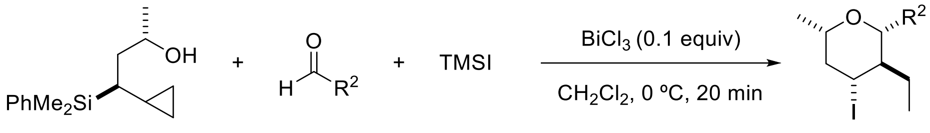

3a (1 equiv) (1.2 equiv) (1.2 equiv) **9**

A solution of $BiCl_3$ (10.1 mg, 0.032 mmol, 0.1 equiv) and the corresponding aldehyde (0.39 mmol, 1.2 equiv) in 5 mL of dry dichloromethane (0.05 M) was cooled to 0 °C (under nitrogen). Then, alcohol **3a** (80 mg, 0.32 mmol, 1.0 equiv) was dissolved in 1 mL of dry dichloromethane and added into the reaction. Finally, the Lewis acid, TMSI (0.054 mL, 0.39 mmol, 1.2 equiv), was added dropwise. The mixture was stirred at 0 ºC while monitored by TLC. When starting materials were consumed, it was hydrolyzed with 5 mL of $Na_2S_2O_3$ (sat). Phases are then separated, extracting the aqueous phase three times with dichloromethane (3 x 10 mL). The organic phases are combined, washed with NaCl sat. (20 mL) and dried over anhydrous Na_2SO_4. The solvent is then evaporated under reduced pressure. The crude mixture is analyzed by NMR and then purified by column chromatography in silica gel, using mixtures of hexane-ethyl acetate and yielding tetrahydropyrans **9**.

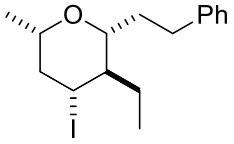

(2*R**,3*R**,4*R**,6*S**)-3-ethyl-4-iodo-6-methyl-2-phenethyltetrahydro-2*H*-pyran (**9a**) was obtained following the general procedure, from cyclopropylsilyl alcohols **3a** (80 mg, 0.32 mmol) and 3-phenylpropanal in 20 minutes, to give, after column chromatography (hexane/EtOAc: 40:1), a yellow oil (83 mg, 72%). **^{1}H NMR (500 MHz, $CDCl_3$)** δ (ppm) 7.32 – 7.26 (m, 2H, Ar-*H*), 7.21 – 7.17 (m, 3H, Ar-*H*), 4.25 (ddd, *J* = 12.3, 11.0, 4.5 Hz, 1H, *H*C-I), 3.43 – 3.30 (m, 1H, *H*C-O), 3.16 (td, *J* = 9.3, 2.6 Hz, 1H, O-C*H*), 2.84 (ddd, *J* = 13.8, 9.3, 4.6 Hz, 1H, C*H*H-Ph), 2.67 (ddd, *J* = 13.8, 9.3, 7.6 Hz, 1H, CH*H*-Ph), 2.47 (ddd, *J* = 13.0, 4.5, 2.0 Hz, 1H, C*H*H), 2.17 (dt, *J* = 13.0, 11.0, 1H, CH*H*), 2.01 – 1.92 (m, 1H, C*H*H), 1.81 – 1.73 (m, 1H, CH*H*), 1.72 – 1.65 (m, 2H), 1.64 – 1.54 (m, 1H), 1.21 (d, *J* = 6.2 Hz, 3H, CH_3), 0.74 (t, *J* = 7.5 Hz, 3H, CH_2-CH_3). **^{13}C NMR (126 MHz, $CDCl_3$)** δ (ppm) 142.3 (C), 128.7 (CH), 128.4 (CH), 125.9 (CH), 78.4 (O-CH), 75.1 (HC-O), 49.8 (CH), 48.9 (CH_2), 35.4 (HC-I), 34.9 (CH_2), 31.7 (CH_2), 25.0 (CH_2), 21.2 (CH_3), 8.6 (CH_3). **HRMS (ESI+)** m/z calc. for $C_{16}H_{24}IO$ ($[M+H]^+$): 359.0866, found 359.0867.

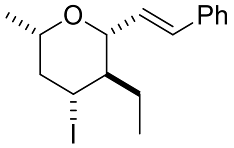

(2*R**,3*R**,4*R**,6*S**)-3-ethyl-4-iodo-6-methyl-2-((*E*)-styryl)tetrahydro-2*H*-pyran (**9b**) was obtained following the general procedure, from cyclopropylsilyl alcohols **3a** (80 mg, 0.32 mmol) and (*E*)-cinnamaldehyde in 10 minutes, to give, after column chromatography (hexane/EtOAc: 20:1), a yellow oil (70 mg, 61%). **^{1}H NMR (500 MHz, $CDCl_3$)** δ (ppm) 7.43 – 7.37 (m, 2H, Ar-*H*), 7.34 – 7.29 (m, 2H, Ar-*H*), 7.28 – 7.22 (m, 1H, Ar-*H*), 6.63 (d, *J* = 15.9 Hz, 1H, =C*H*), 6.17 (dd, *J* = 15.9, 8.0 Hz, 1H, *H*C=), 4.31 (td, *J* = 11.9, 4.4 Hz, 1H, *H*C-I), 3.89 (dd, *J* = 9.7, 8.0 Hz, 1H, O-C*H*), 3.57 – 3.49 (m, 1H, *H*C-O), 2.51 (ddd, *J* = 13.1, 4.4, 2.0 Hz, 1H, C*H*H), 2.23 (dt, *J* = 13.1, 11.9, Hz, 1H, CH*H*), 1.86– 1.77 (m, 1H, CH), 1.76 – 1.60 (m, 2H, CH_2-CH_2), 1.22 (d, *J* = 6.2 Hz, 3H, CH_3), 0.84 (t, *J* = 7.5 Hz, 3H, CH_3). **^{13}C NMR (126 MHz, $CDCl_3$)** δ (ppm) 136.5 (C), 133.2 (=CH), 128.7 (CH), 128.3 (HC=), 128.0 (CH), 126.8 (CH), 81.3 (O-CH), 74.8 (HC-O), 50.1 (CH), 48.6 (CH_2), 33.0 (HC-I), 24.9 (CH_2), 21.2 (CH_3), 8.6 (CH_3). **HRMS (ESI+)** m/z calc. for $C_{16}H_{22}IO$ ($[M+H]^+$): 357.0710, found 357.0711.

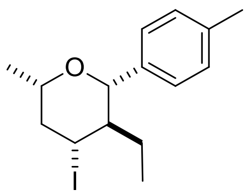

(2*S**,3*R**,4*R**,6*S**)-4-iodo-3-ethyl-6-methyl-2-(*p*-tolyl)tetrahydro-2*H*-pyran (**9c**) was obtained following the general procedure, from cyclopropylsilyl alcohol **3a** (80 mg, 0.32 mmol) and 4-methylbenzaldehyde in 20 minutes, to give, after column chromatography (hexane/EtOAc: 15:1), a yellow oil (84 mg, 76%). **^{1}H NMR (500 MHz, $CDCl_3$)** δ (ppm) 7.22 (d, *J* = 8.1 Hz, 2H, Ar-*H*), 7.15 (d, *J* = 8.1 Hz, 2H, Ar-*H*), 4.09 (td, *J* = 11.9, 4.4 Hz, 1H, *H*C-I), 4.18 (d, *J* = 10.0 Hz, 1H, O-C*H*), 3.61 – 3.52 (m, 1H, *H*C-O), 2.56 (ddd, *J* = 13.0, 4.4, 2.0 Hz, 1H, C*H*H), 2.38 – 2.27 (m, 1H, CH*H*), 2.34 (s, 3H, CH_3), 2.05 – 1.97 (m, 1H, CH), 1.61 – 1.51 (m, 1H, C*H*H-CH_3), 1.28 – 1.18 (m, 1H, CH*H*-CH_3), 1.21 (d, *J* = 6.1 Hz, 3H, CH_3), 0.66 (t, *J* = 7.6 Hz, 3H, CH_3). **^{13}C NMR (126 MHz, $CDCl_3$)** δ (ppm) 138.0 (C), 137.8 (C), 129.3 (CH), 127.3 (CH), 83.0 (O-CH), 75.5 (HC-O), 51.1 (CH), 48.8 (CH_2), 33.7 (HC-I), 24.8 (CH_2), 21.3 (CH_3), 21.3 (CH_3), 8.4 (CH_3). **HRMS (ESI+)** m/z calc. for $C_{15}H_{22}IO$ ($[M+H]^+$): 345.0710, found 345.0704.

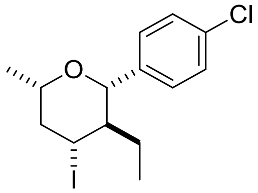

(2*S**,3*R**,4*R**,6*S**)-2-(4-chlorophenyl)-3-ethyl-4-iodo-6-methyltetrahydro-*2H*-pyran (**9d**) was obtained following the general procedure, from cyclopropylsilyl alcohols **3a** (80 mg, 0.32 mmol) and 4-chlorobenzaldehyde in 15 minutes, to give, after column chromatography (hexane/EtOAc: 20:1), a yellow oil (84 mg, 72%). **^{1}H NMR (500 MHz, $CDCl_3$)** δ (ppm) 7.32 (d, *J* = 8.5 Hz, 2H, Ar-*H*), 7.27 (d, *J* = 8.5 Hz, 2H, Ar-*H*), 4.35 (td, *J* = 11.9, 4.4 Hz, 1H, *H*C-I), 4.19 (d, *J* = 10.0 Hz, 1H, O-C*H*), 3.61 – 3.53 (m, 1H, *H*C-O), 2.56 (ddd, *J* = 13.1, 4.4, 2.0 Hz, 1H, C*H*H), 2.30 (dt, *J* = 13.1, 11.9 Hz, 1H, CH*H*), 1.98 – 1.90 (m, 1H, CH), 1.61 – 1.51 (m, 1H, C*H*H-CH_3), 1.25 – 1.15 (m, 1H, CH*H*-CH_3), 1.21 (d, *J* = 6.2 Hz, 3H, CH_3), 0.65 (t, *J* = 7.6 Hz, 3H, CH_3). **^{13}C NMR (126 MHz, $CDCl_3$)** δ (ppm) 139.2 (C), 134.0 (C), 128.8 (CH), 128.8 (CH), 82.3 (O-CH), 75.6 (HC-O), 51.1 (CH), 48.6 (CH_2), 32.8 (HC-I), 24.7 (CH_2), 21.2 (CH_3), 8.5 (CH_3). **HRMS (ESI+)** m/z calc. for $C_{14}H_{19}ClIO$ ($[M+H]^+$): 365.0164, found 365.0162.

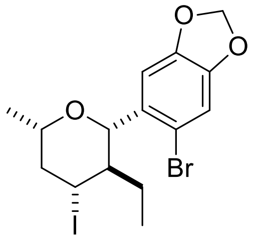

(2*S**,3*R**,4*R**,6*S**)-2-(6-bromobenzo[d][1,3]dioxol-5-yl)-3-ethyl-4-iodo-6-methyltetrahydro-*2H*-pyran (**9e**) was obtained following the general procedure, from cyclopropylsilyl alcohol **3a** (80 mg, 0.32 mmol) and 6-bromopiperonal in 15 minutes, to give, after column chromatography (hexane/EtOAc: 20:1), as a white solid (79 mg, 55%). **^{1}H NMR (500 MHz, $CDCl_3$)** δ (ppm) 6.97 (s, 1H, Ar-*H*), 6.93 (s, 1H, Ar-*H*), 5.98 – 5.94 (m, 2H, O-CH_2-O), 4.71 (d, *J* = 10.0 Hz, 1H, O-C*H*), 4.35 (td, *J* = 11.9, 4.4 Hz, 1H, *H*C-I), 3.64 – 3.55 (m, 1H, *H*C-O), 2.55 (ddd, *J* = 13.1, 4.4, 1.9 Hz, 1H, C*H*H), 2.26 (dt, *J* = 13.1, 11.9 Hz, 1H, CH*H*), 2.01 – 1.91 (m, 1H, CH), 1.65 – 1.55 (m, 1H, C*H*H-CH_3), 1.31 – 1.22 (m, 1H, CH*H*-CH_3), 1.20 (d, *J* = 6.1 Hz, 3H, C*H_3*), 0.73 (t, *J* = 7.6 Hz, 3H, CH_3). **^{13}C NMR (126 MHz, $CDCl_3$)** δ (ppm) 148.3 (C), 148.1 (C), 133.3 (C), 114.6 (C), 112.4 (CH), 108.6 (CH), 102.0 (CH_2), 80.6 (O-CH), 75.5 (HC-O), 51.4 (CH), 48.6 (CH_2), 32.9 (HC-I), 24.4 (CH_2), 21.2 (CH_3), 9.6 (CH_3). **HRMS (ESI+)** m/z calc. for $C_{15}H_{19}BrIO_3$ ($[M+H]^+$): 452.9557, found 452.9553. **Melting point** = 106.7 – 108.3 °C.

3. **X-RAY CRYSTALLOGRAPHIC DATA FOR COMPOUND 4u**

The crystal structure has been deposited at the Cambridge Crystallographic Date Center and allocated the deposition number CCDC: 2326085. This data can be obtained free of charge from the Cambridge Crystallographic Date Center via www.ccdc.cam.ac.uk/data_request/ci

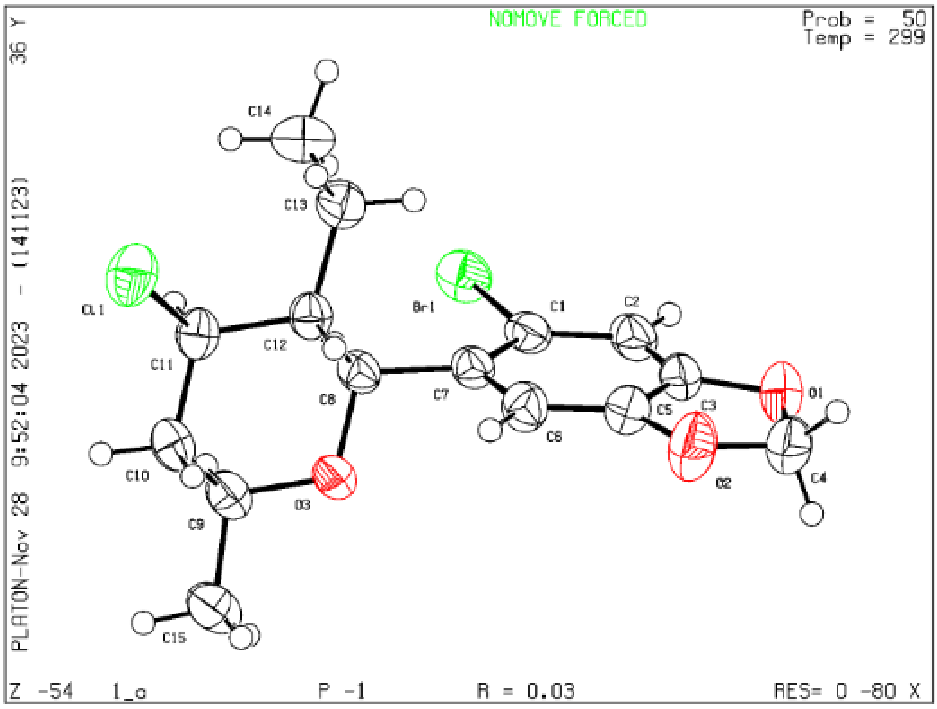

Figure 1. *X-ray crystal structure of (2S*,3R*,4R*,6S*)-2-(6-Bromobenzo[d][1,3]dioxol-5-yl)-4-chloro-3-ethyl-6-methyltetrahydro-2H-pyran (**4u**).*

Experimental.

Single crystals of $C_{15}H_{18}BrClO_3$ (**4u**) were grown from a dichloromethane saturated solution, by slow evaporation.

A colourless specimen of $C_{15}H_{18}BrClO_3$, approximate dimensions 0.030 mm x 0.050 mm x 0.100 mm, was used for the X-ray crystallographic analysis. The X-ray intensity data were measured (λ = 1.54184 Å). A total of 1655 frames were collected. The total exposure time was 4.60 hours. The frames were integrated with the Bruker SAINT software package using a narrow-frame algorithm. Data integration, scaling, and empirical absorption correction were performed using the CrysAlisPro software package.[1] The structure was solved by direct methods and refined by full-matrix-least-squares against F2 with SHELX[2] in OLEX2.[3] Non-hydrogen atoms were refined anisotropically, and hydrogen atoms were placed at idealized positions and refined using the riding model. Graphic was made using MERCURY.[4]

X-Ray crystallographic data of **4u** (Figure 1). Crystal data and structure refinement for **4u**

Bond precision: C-C = 0.0036 A Wavelength=1.54178

Cell:	a=7.9865(4)	b=9.4209(4)	c=10.9005(5)
	alpha=103.608(1)	beta=92.939(1)	gamma=105.462(1)
Temperature:	299 K		

	Calculated	Reported
Volume	762.57(6)	762.57(6)
Space group	P -1	P -1
Hall group	-P 1	-P 1
Moiety formula	C15 H18 Br Cl O3	C15 H18 Br Cl O3
Sum formula	C15 H18 Br Cl O3	C15 H18 Br Cl O3
Mr	361.64	361.65
Dx,g cm-3	1.575	1.575
Z	2	2
Mu (mm-1)	5.327	5.327
F000	368.0	368.0
F000'	368.20	
h,k,lmax		9,11,13
Nref		2859
Tmin,Tmax	0.763,0.852	0.428,0.754
Tmin'	0.559	

Correction method= # Reported T Limits: Tmin=0.428 Tmax=0.754
AbsCorr = MULTI-SCAN

Data completeness= Theta(max)= 72.079

R(reflections)= 0.0339(2708) wR2(reflections)= 0.0773(2859)

S = 1.076 Npar= 183

4. COPIES OF NMR SPECTRA

Compound 3a

^{1}H NMR (500 MHz, $CDCl_3$)

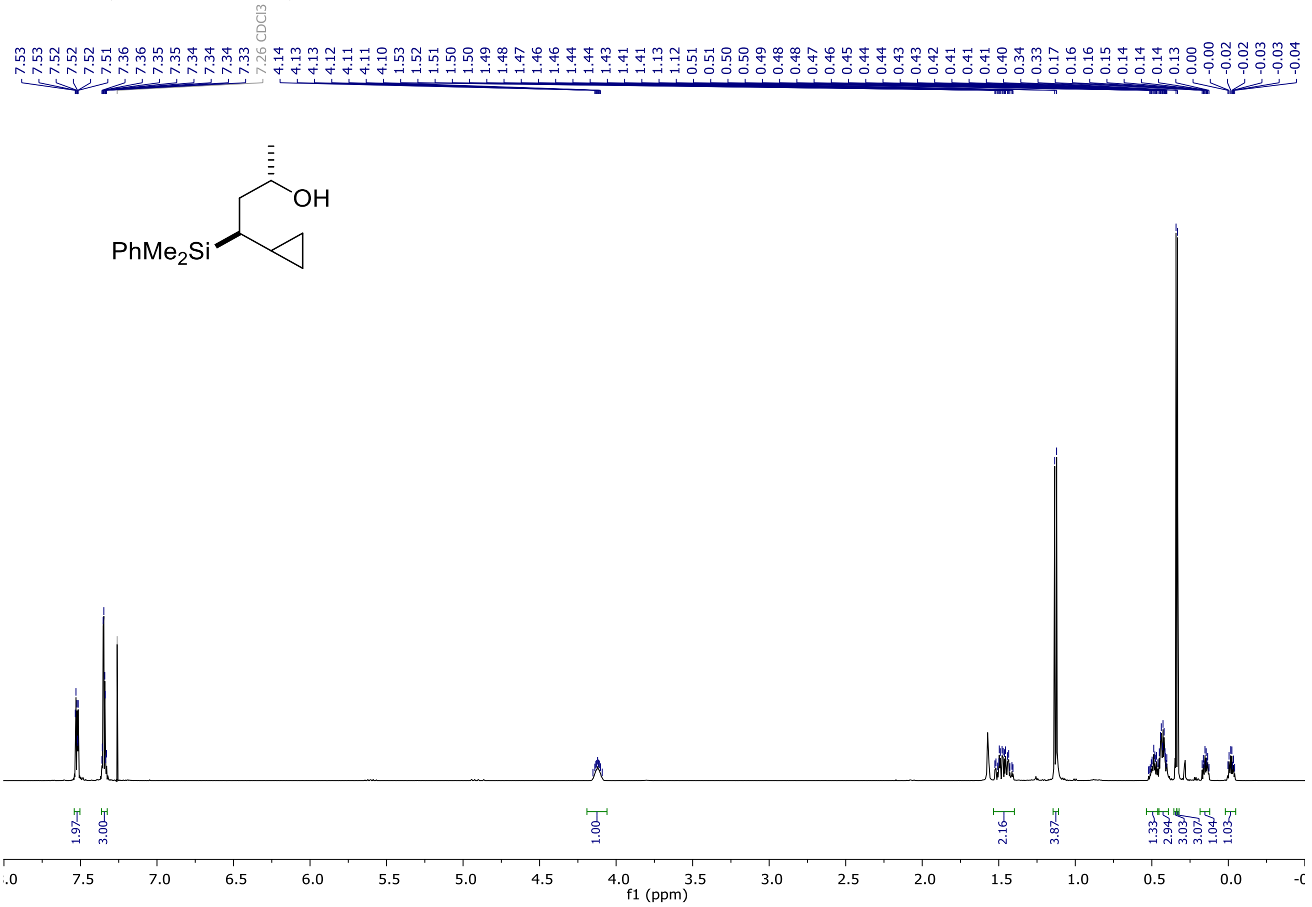

^{13}C NMR (101 MHz, $CDCl_3$)

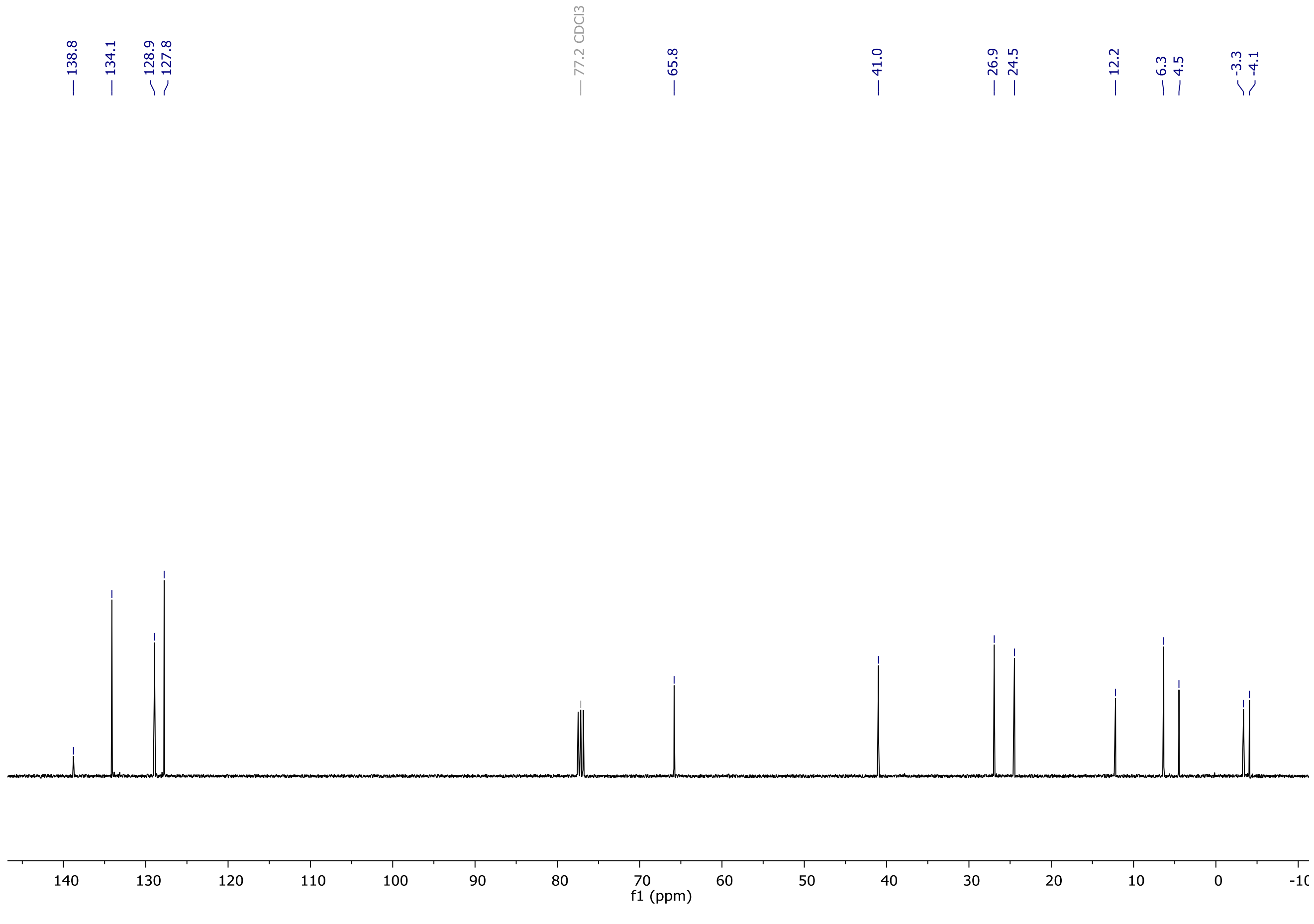

2D-COSY

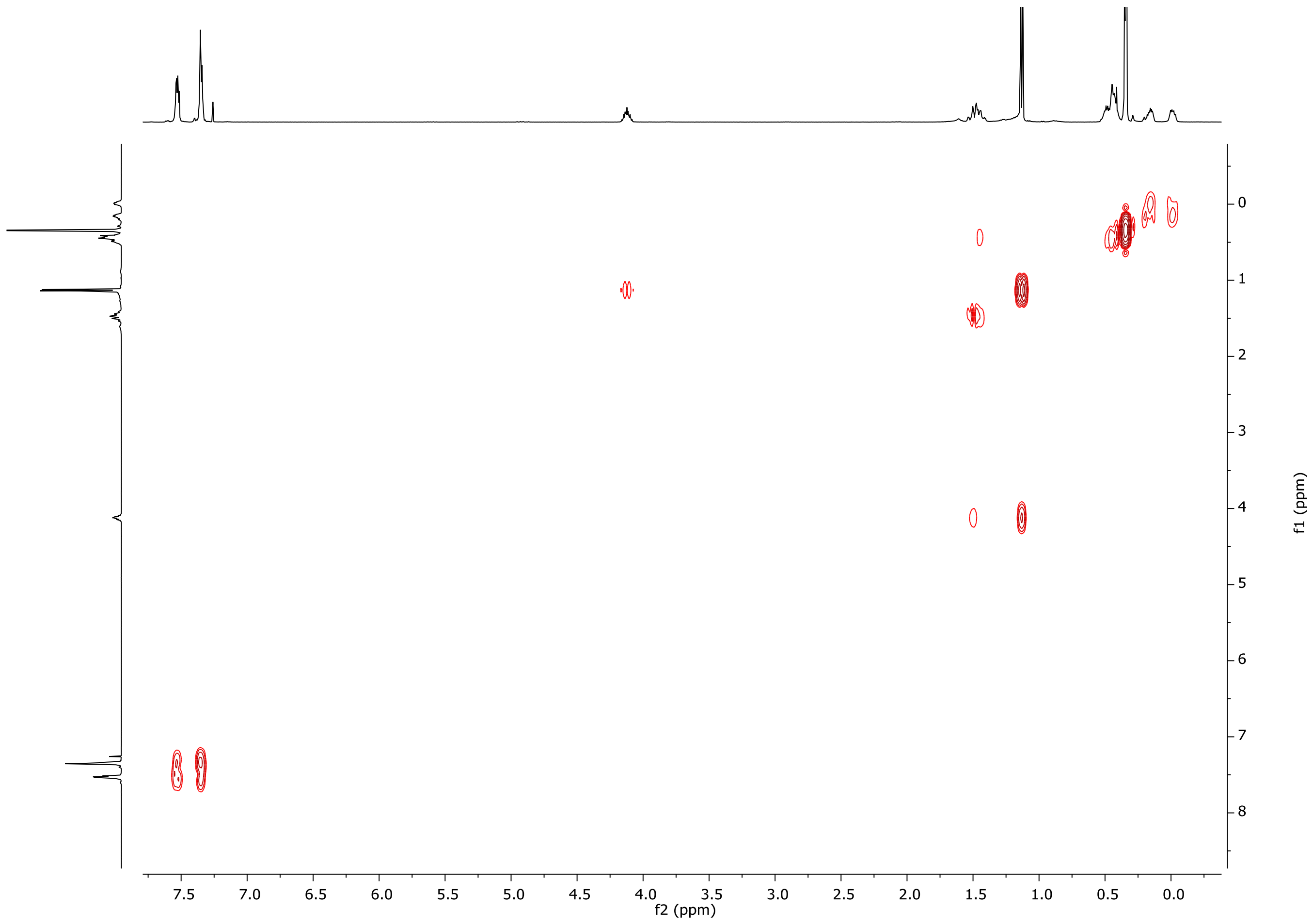

2D-HSQC

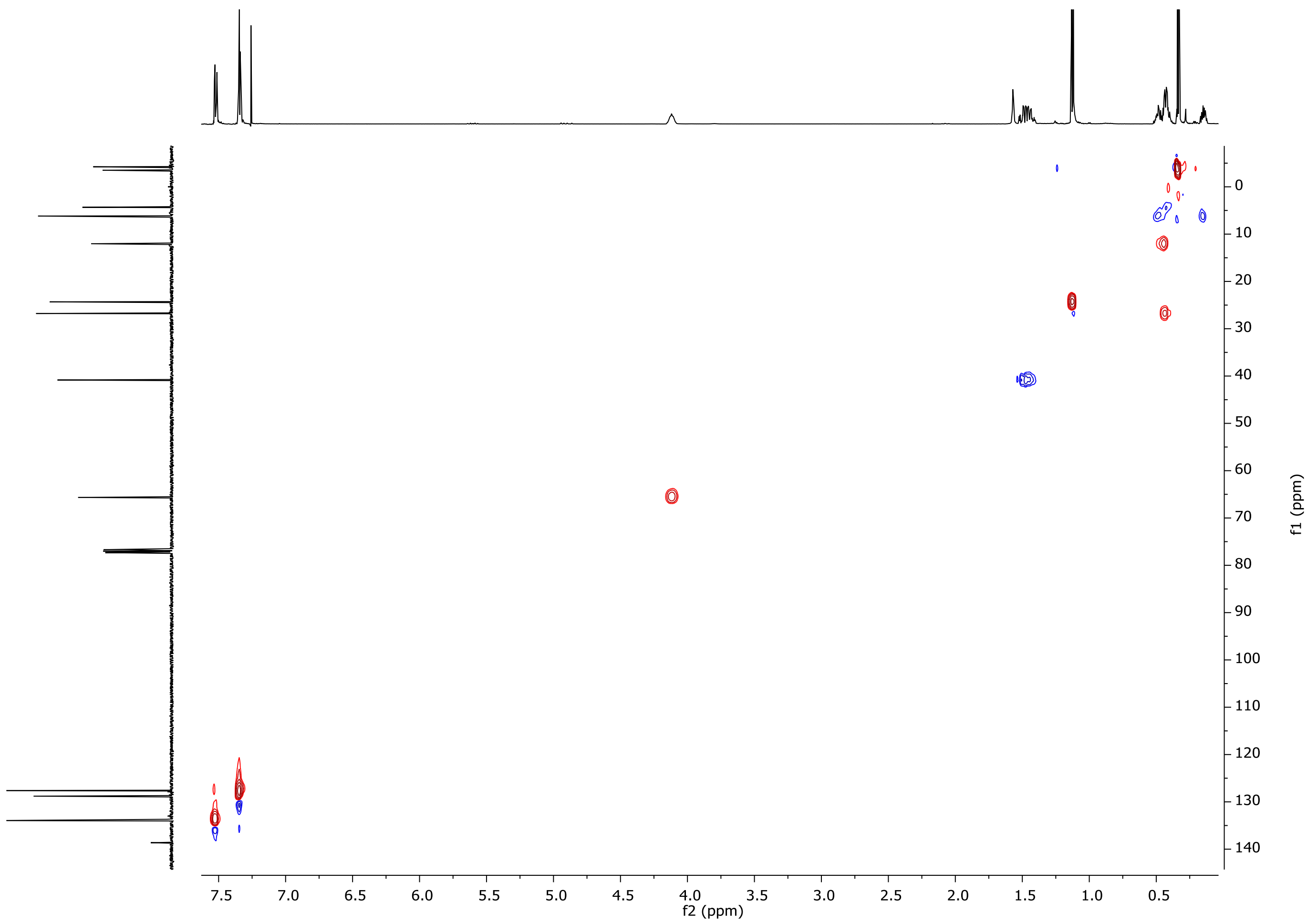

Compound 3b

^{1}H NMR (500 MHz, $CDCl_3$)

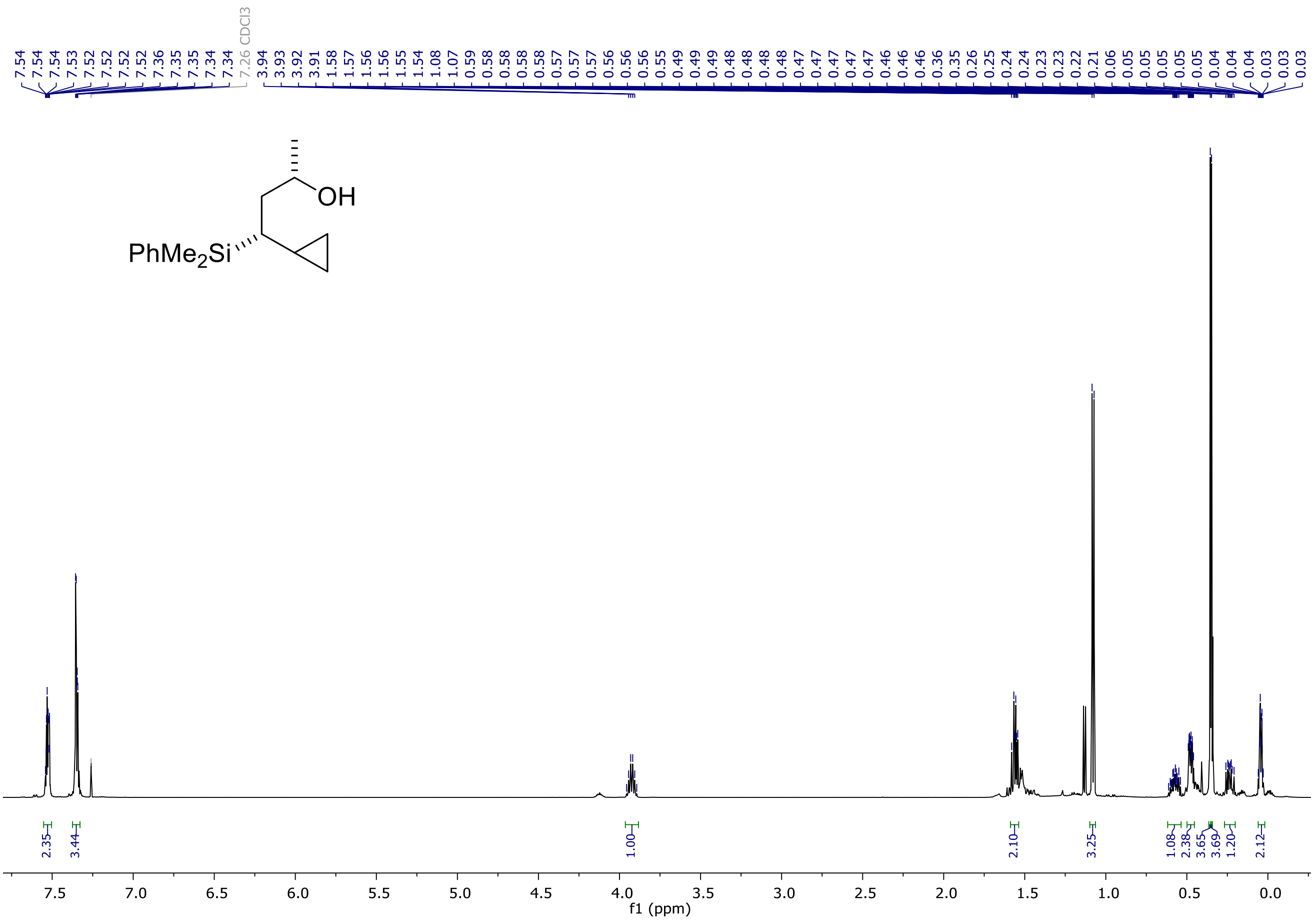

^{13}C NMR (101 MHz, $CDCl_3$)

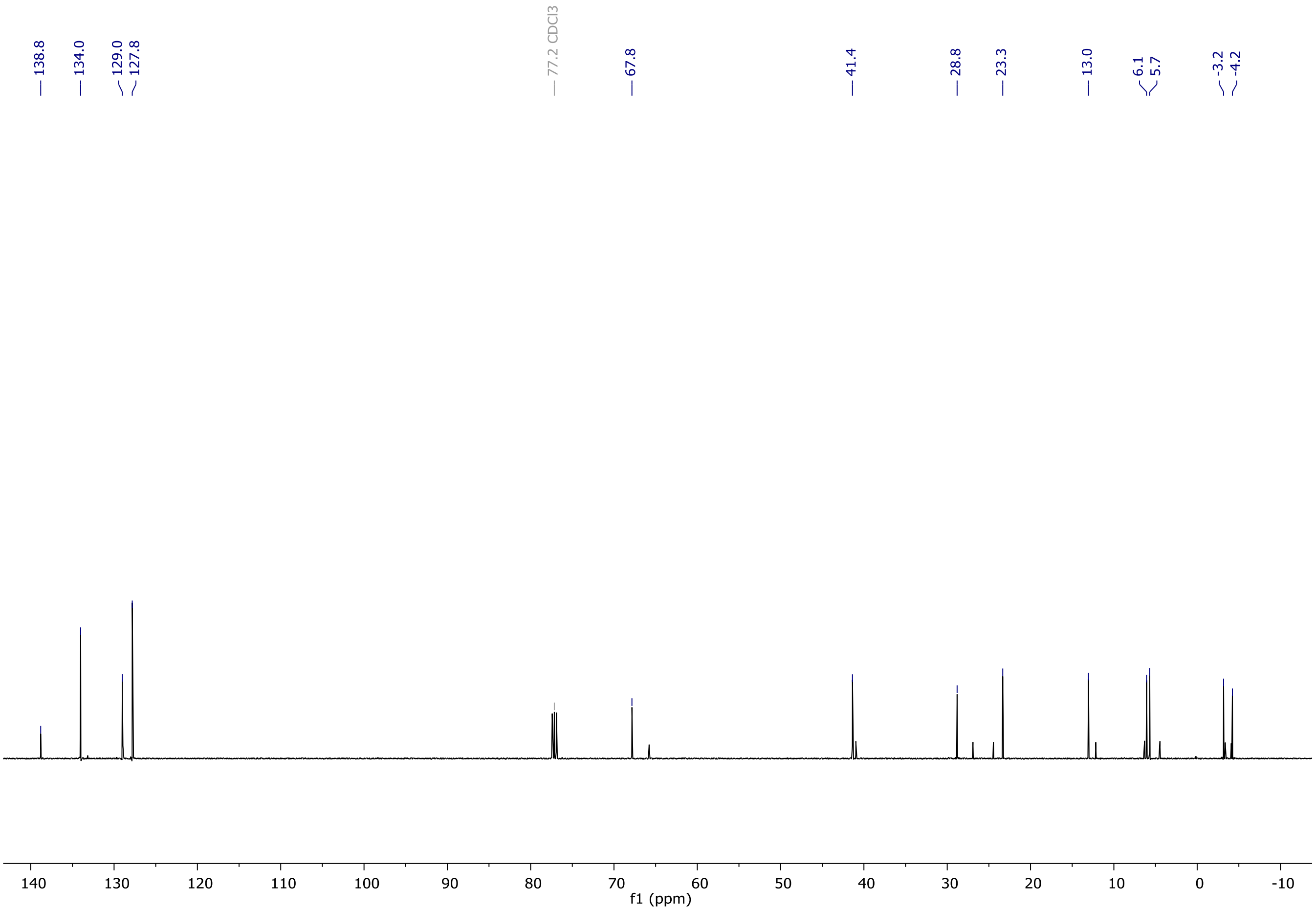

2D-COSY

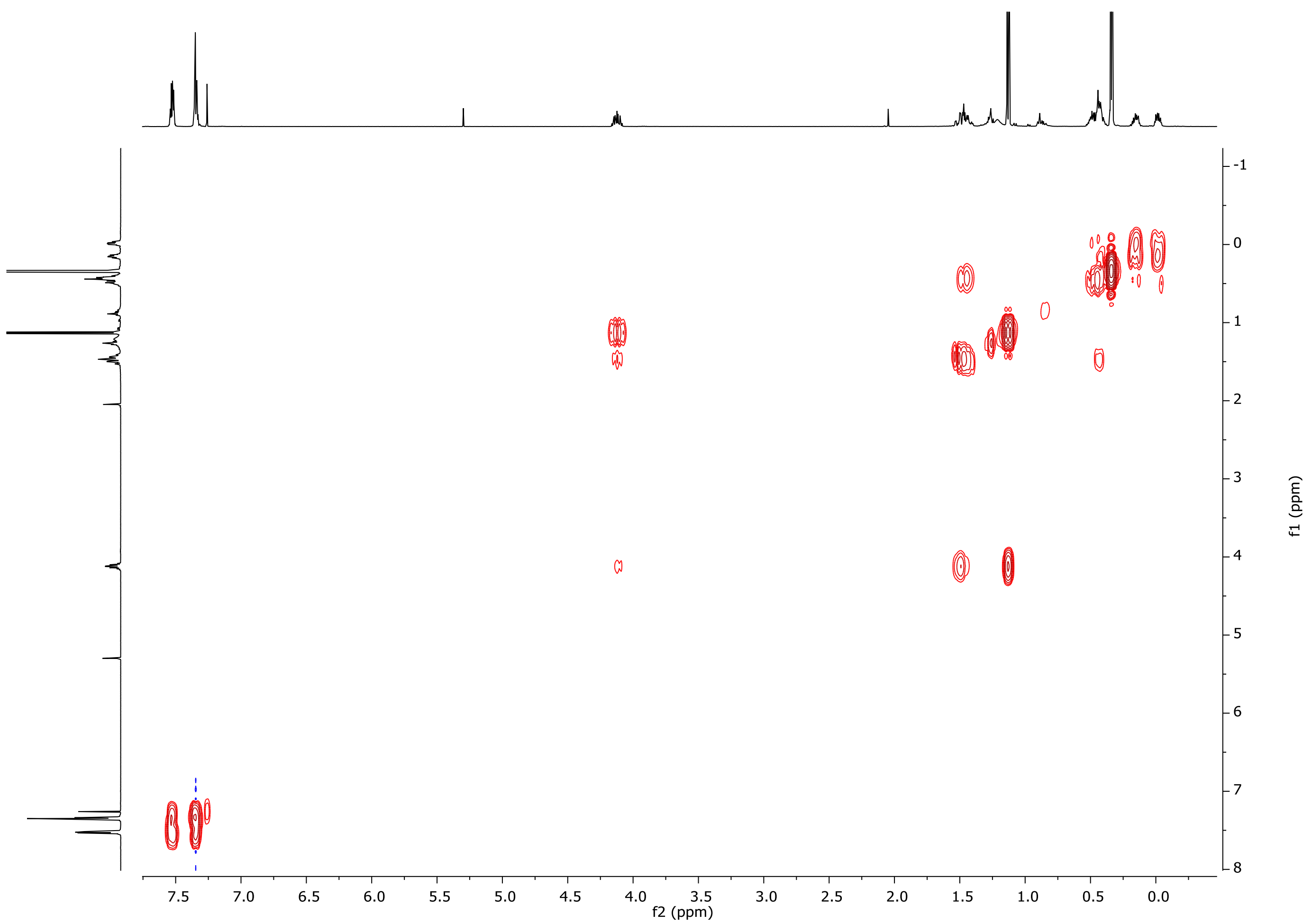

2D-HSQC

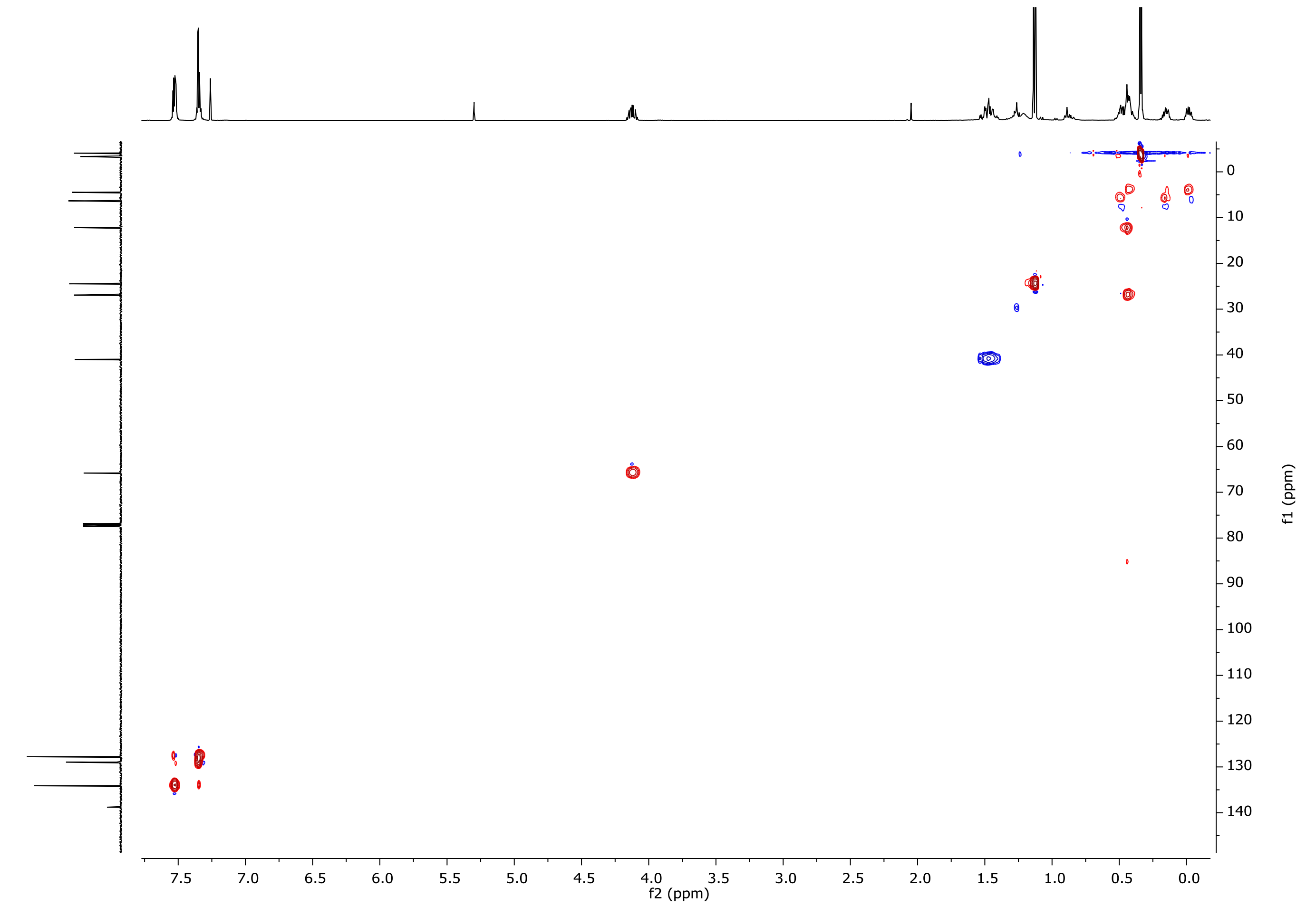

Compound 3c

^{1}H NMR (400 MHz, $CDCl_3$)

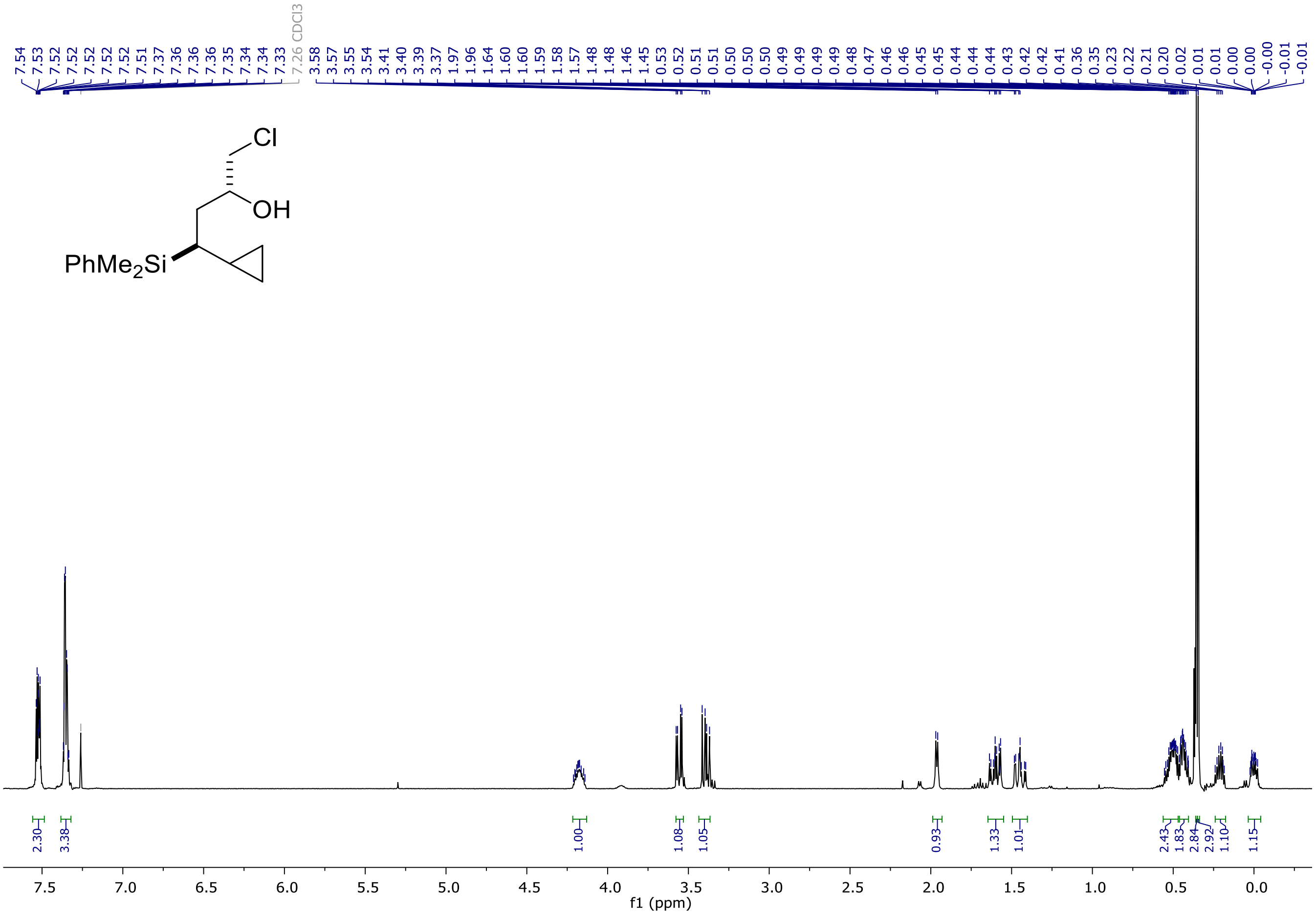

^{13}C NMR (101 MHz, $CDCl_3$)

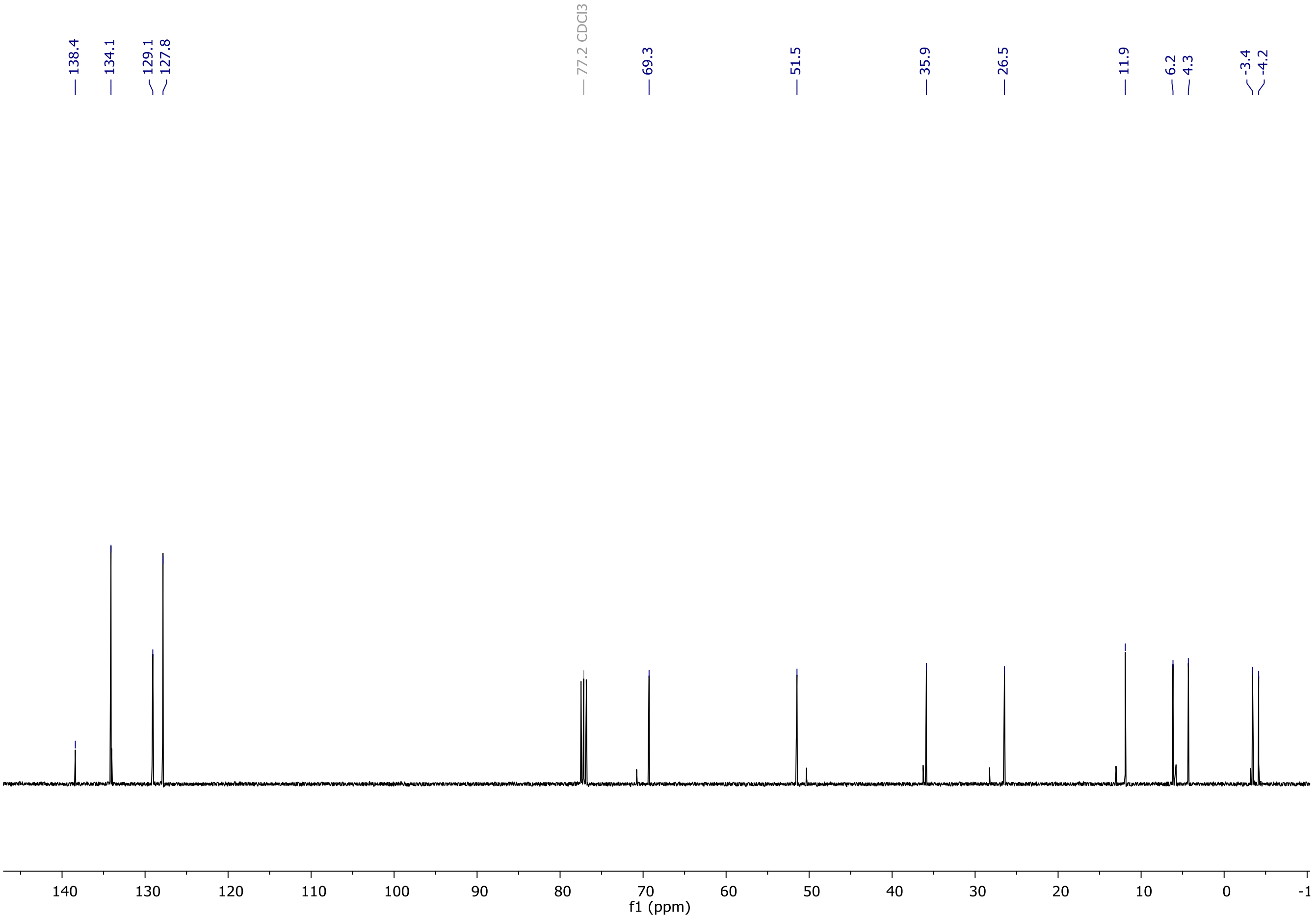

2D-COSY

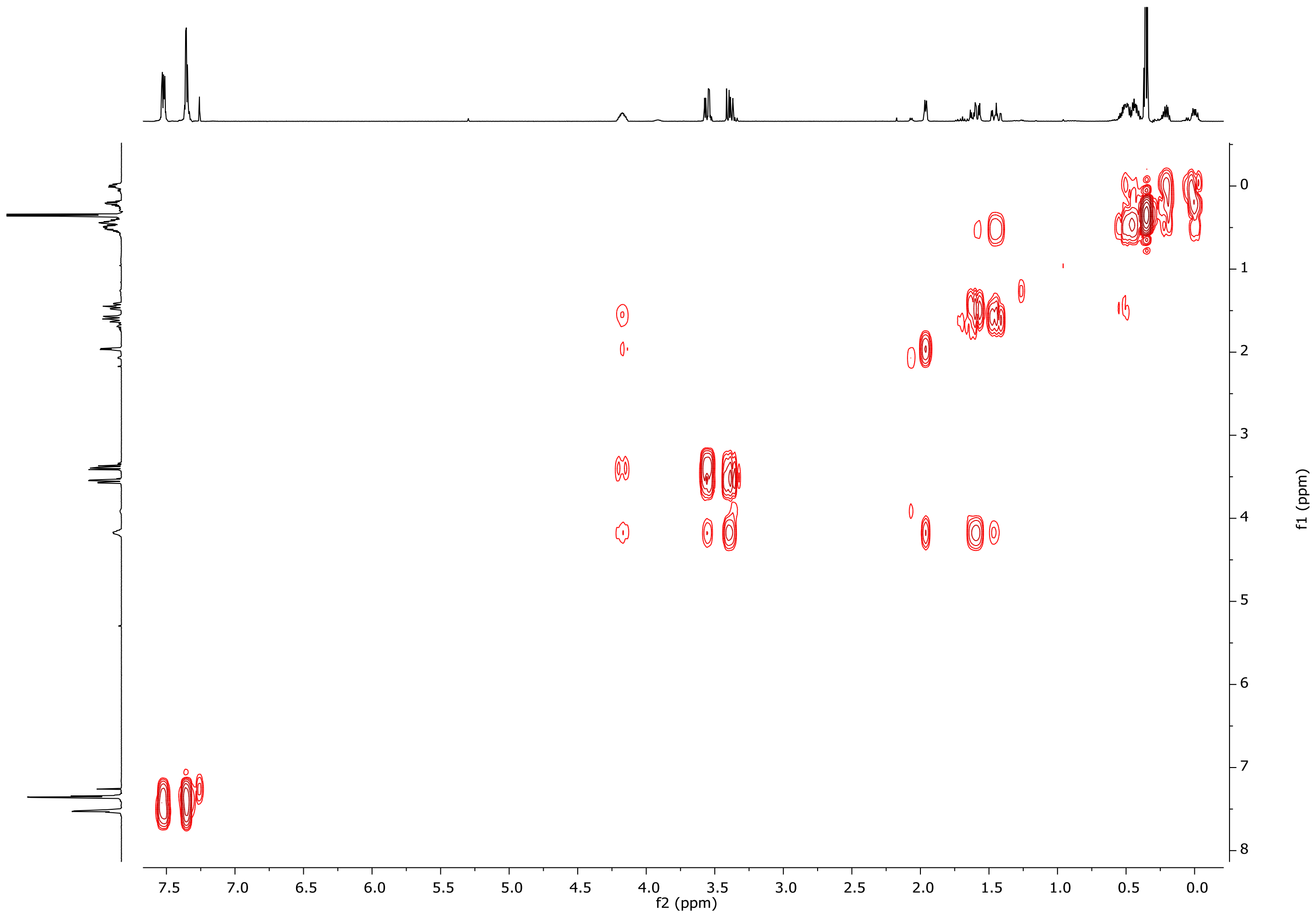

2D-HSQC

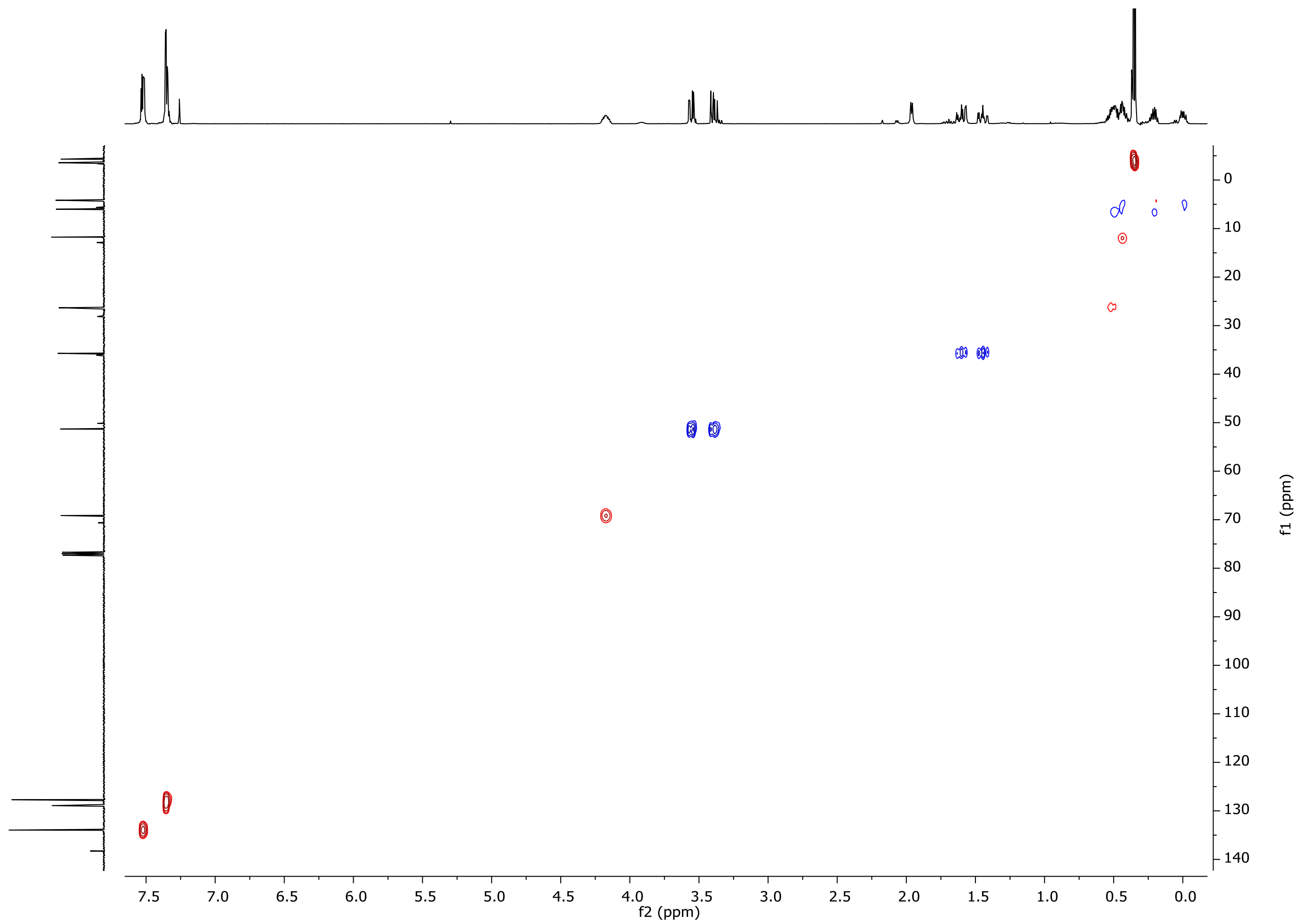

Compound 3d

^{1}H NMR (400 MHz, $CDCl_3$)

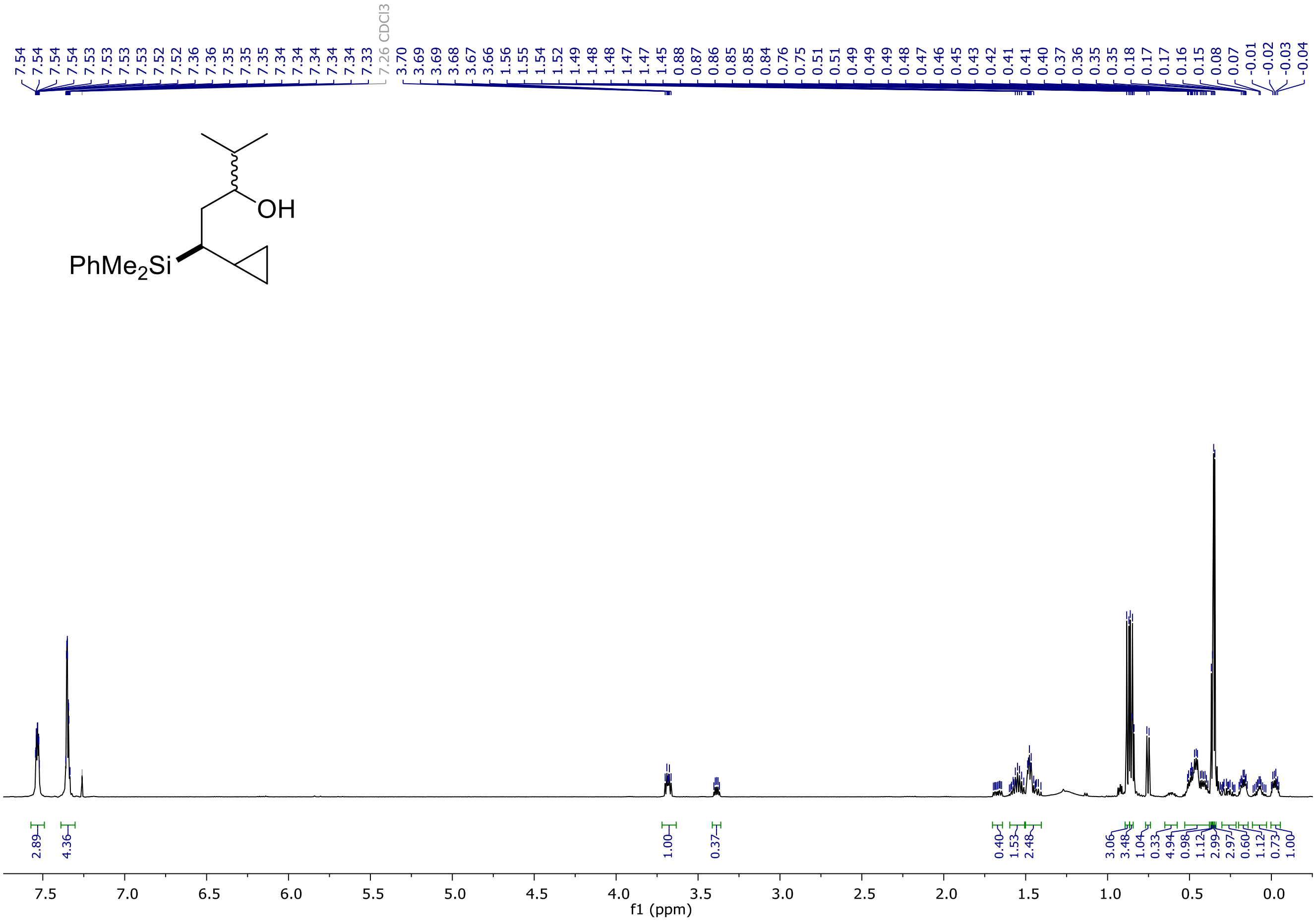

^{13}C NMR (101 MHz, $CDCl_3$)

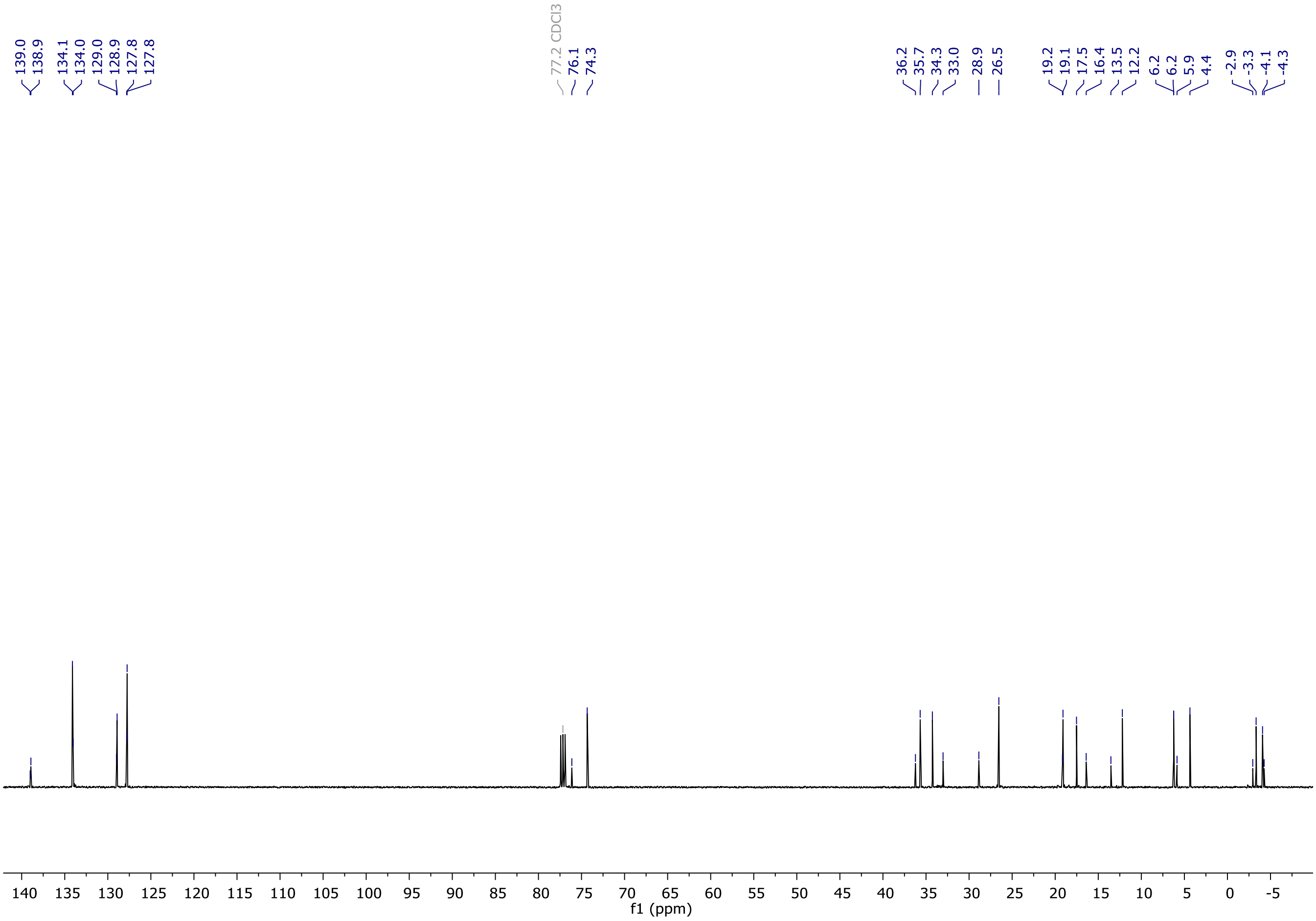

2D-COSY

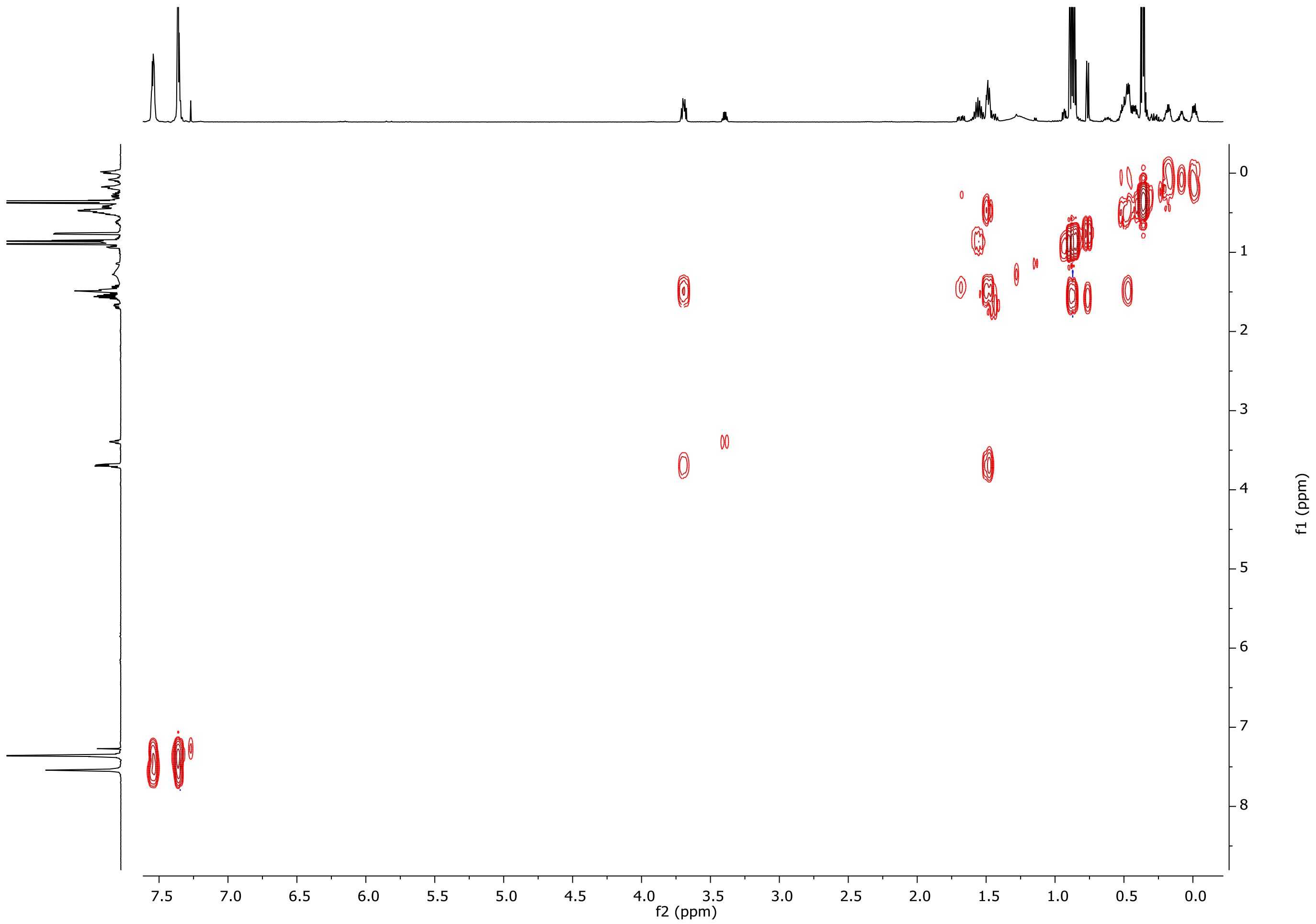

2D-HSQC

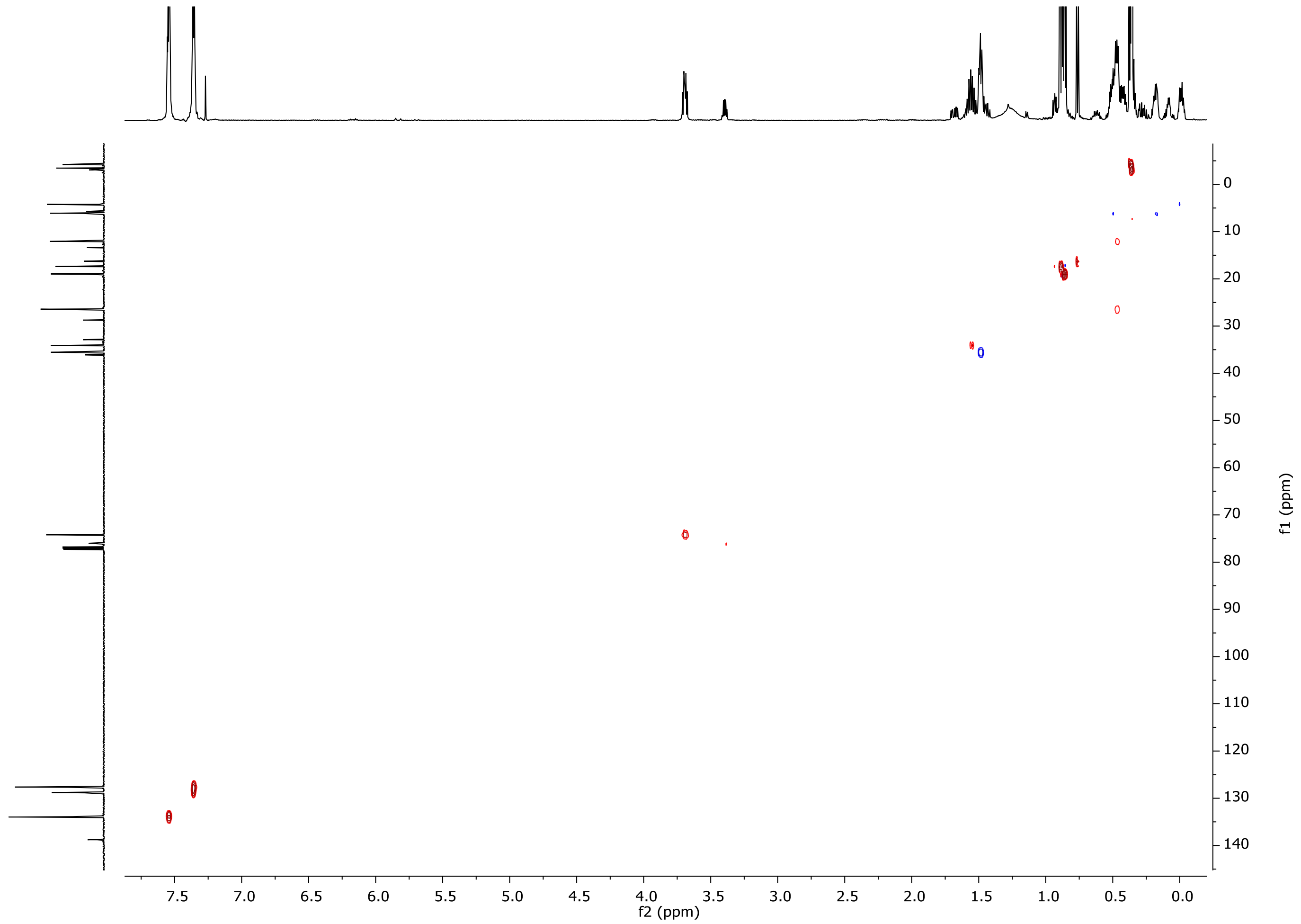

Compound 3e

^{1}H NMR (400 MHz, $CDCl_3$)

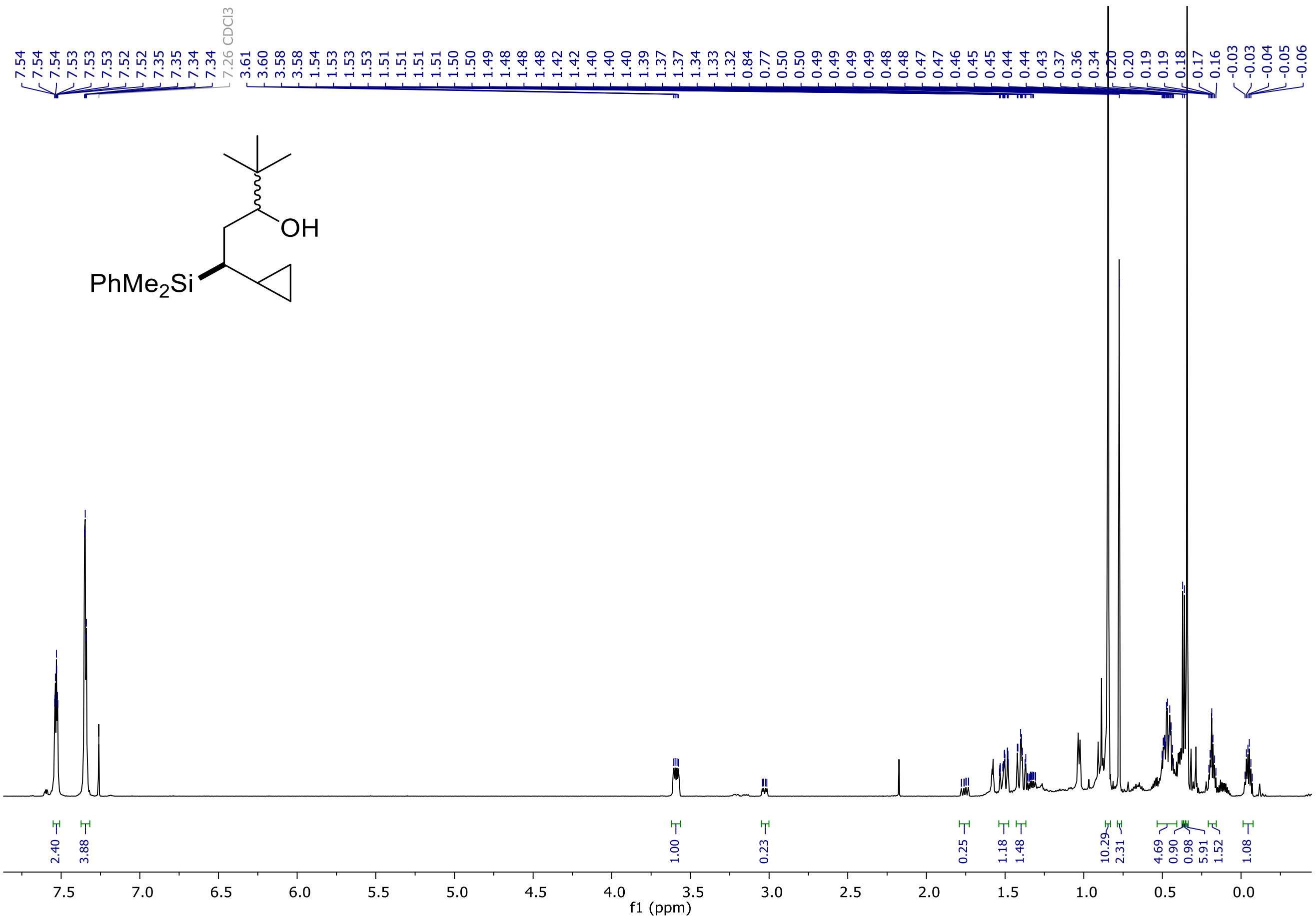

^{13}C NMR (101 MHz, $CDCl_3$)

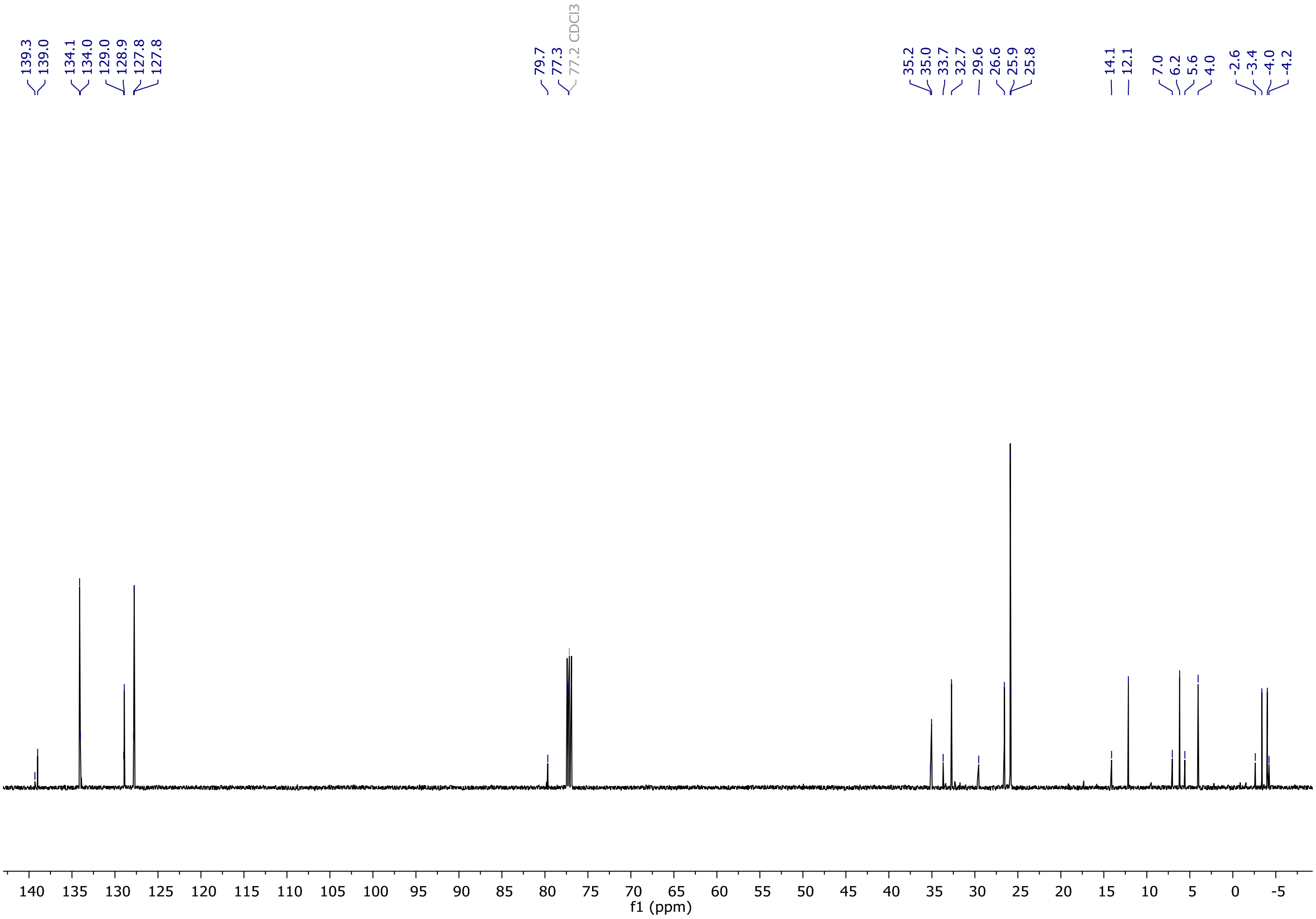

2D-COSY

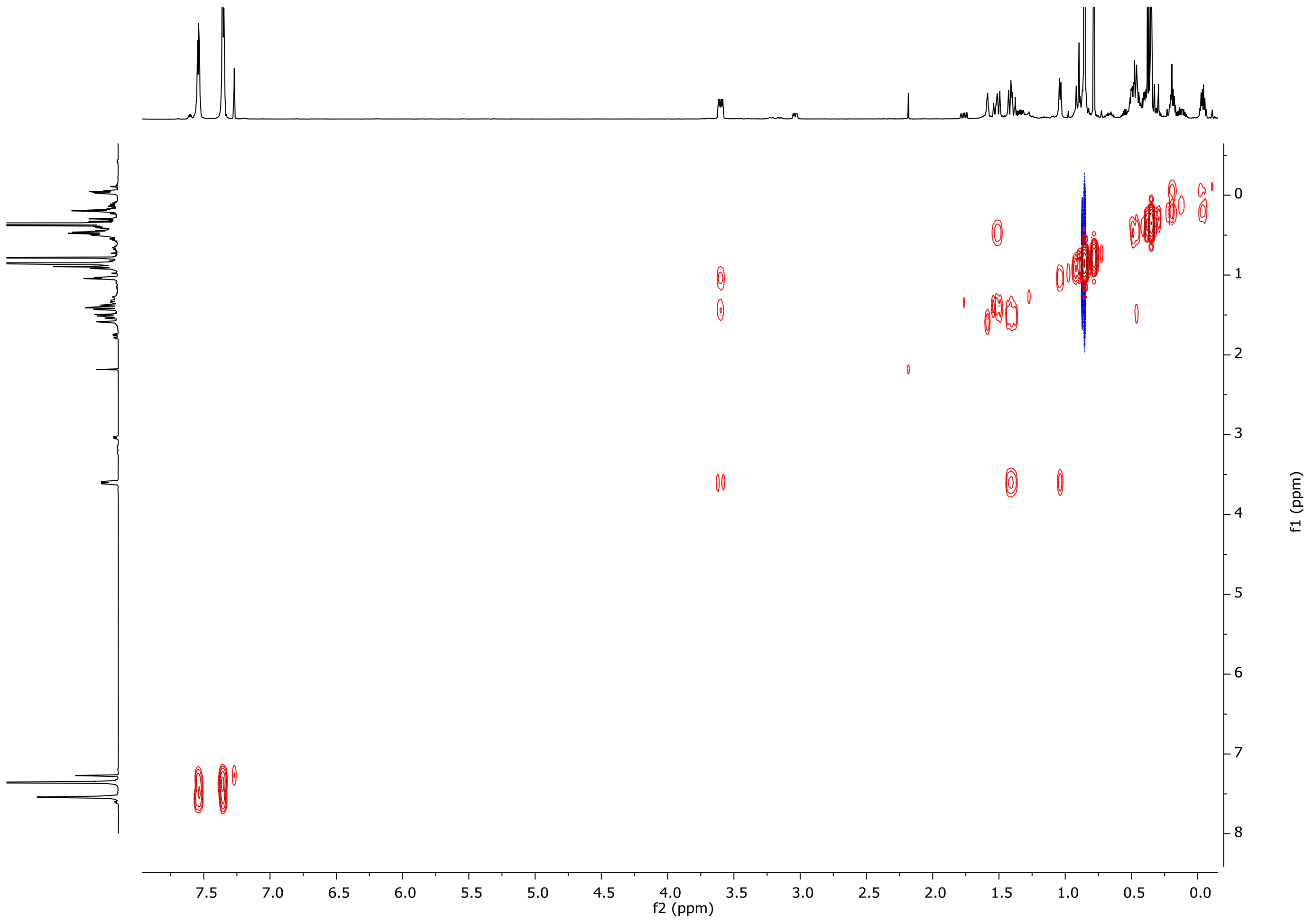

2D-HSQC

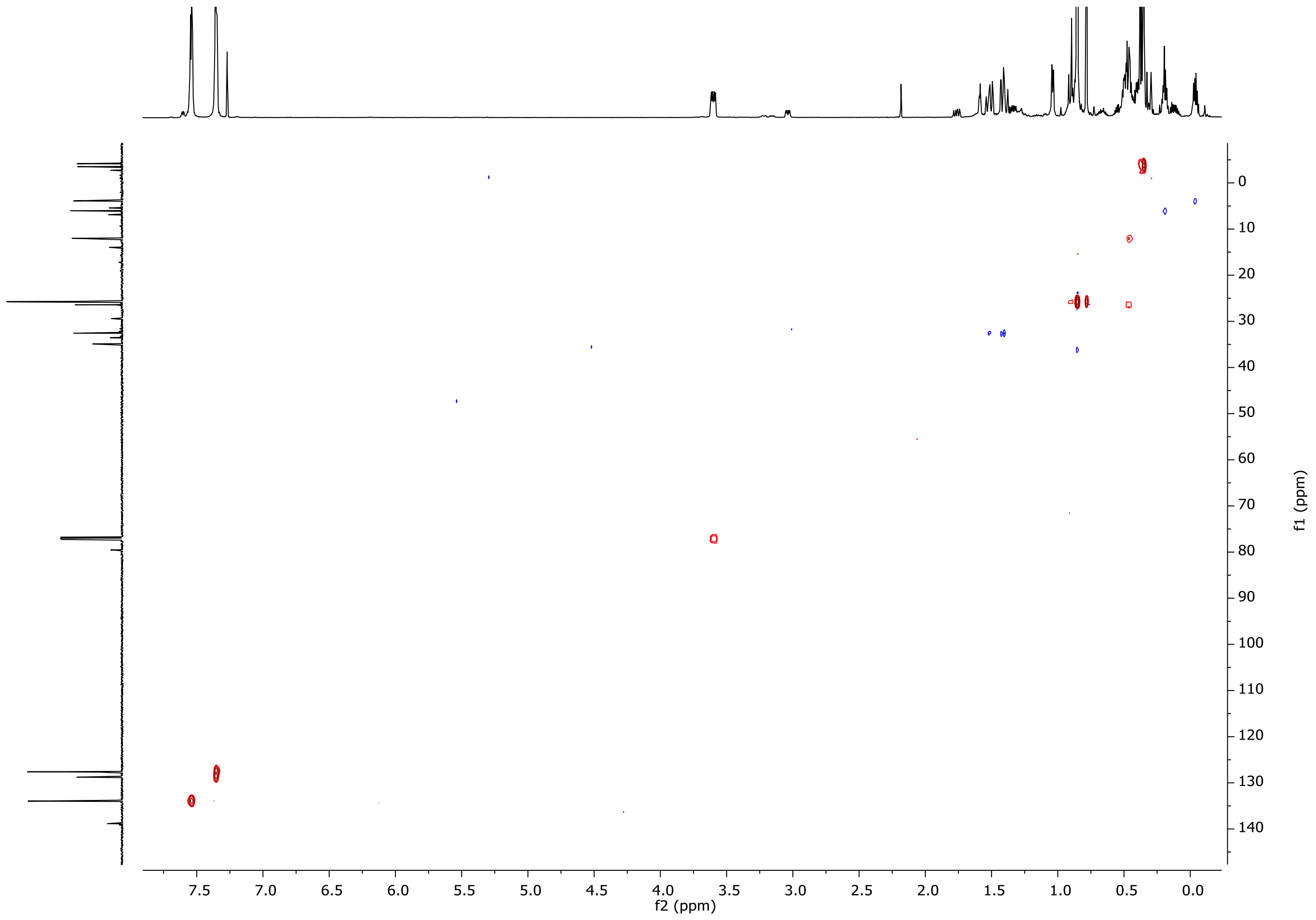

Compound 3f

^{1}H NMR (500 MHz, $CDCl_3$)

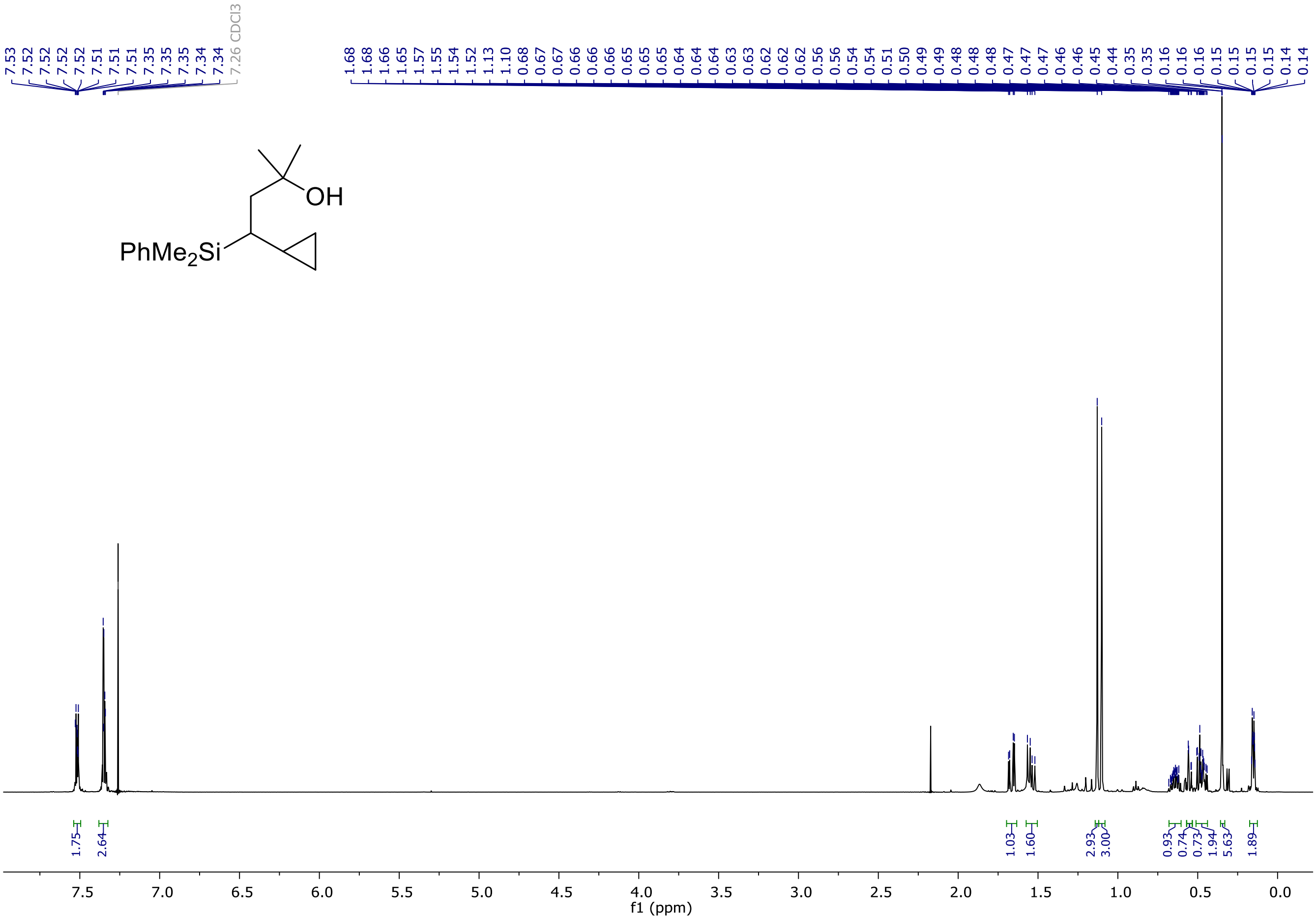

^{13}C NMR (101 MHz, $CDCl_3$)

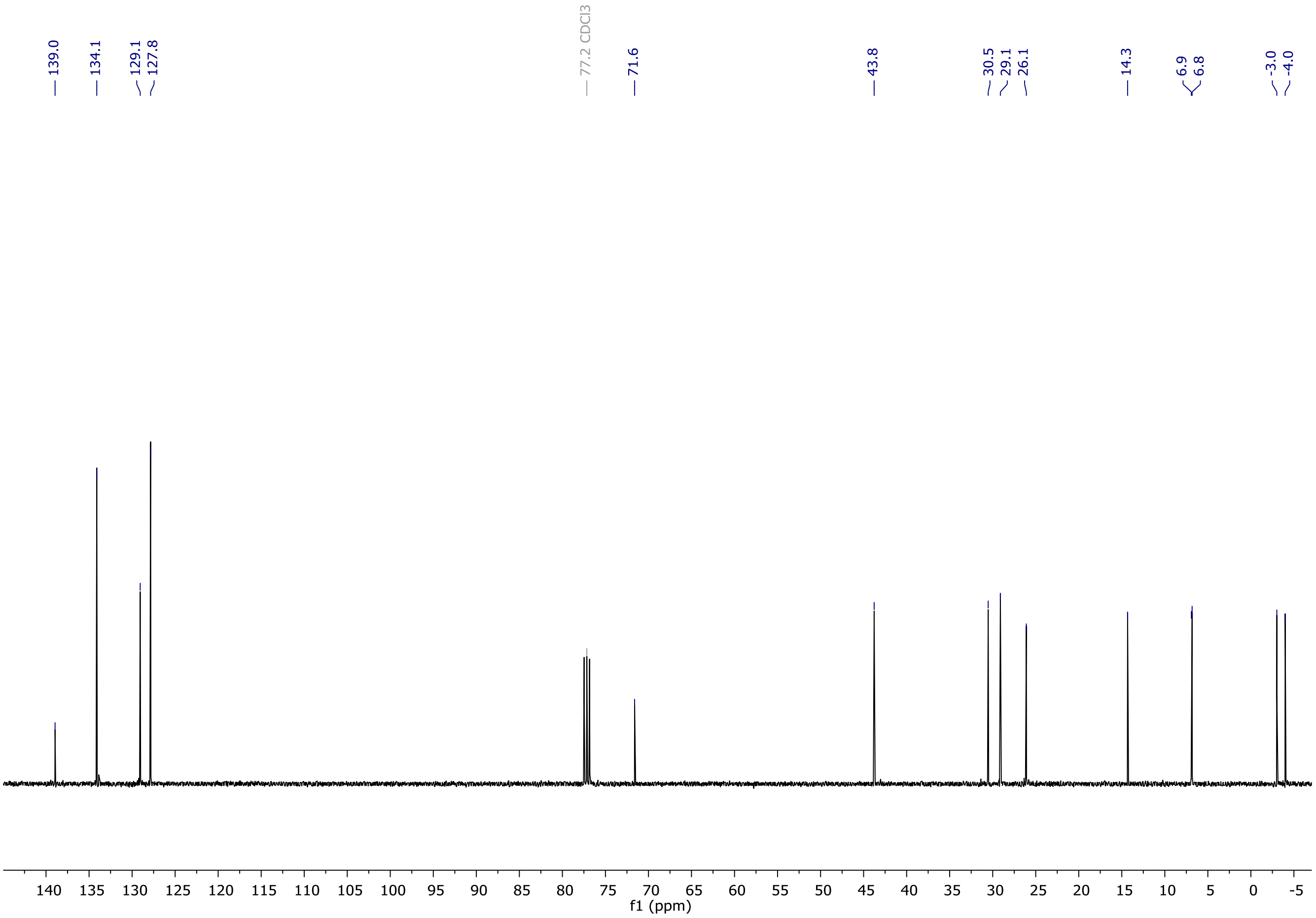

2D-COSY

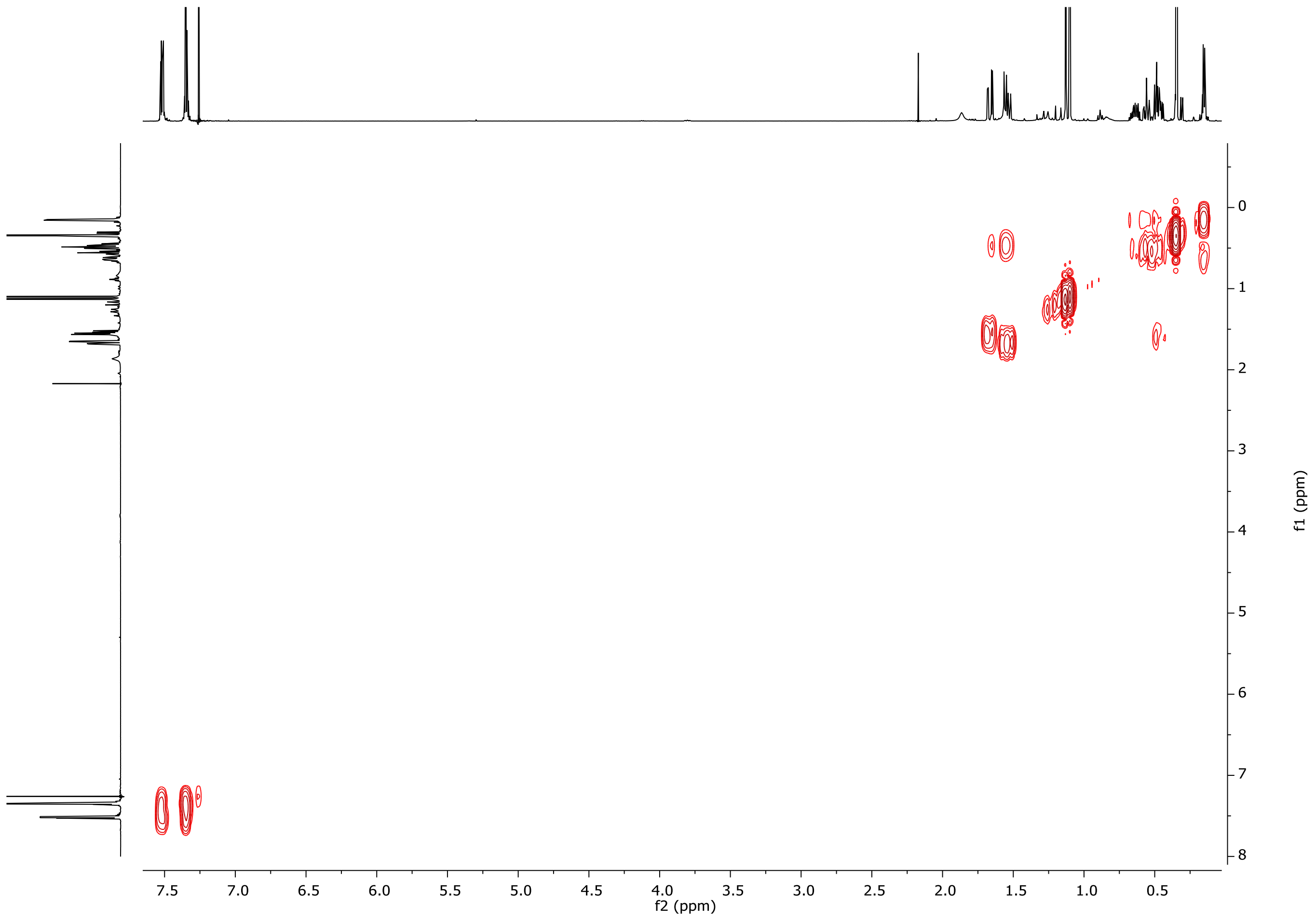

2D-HSQC

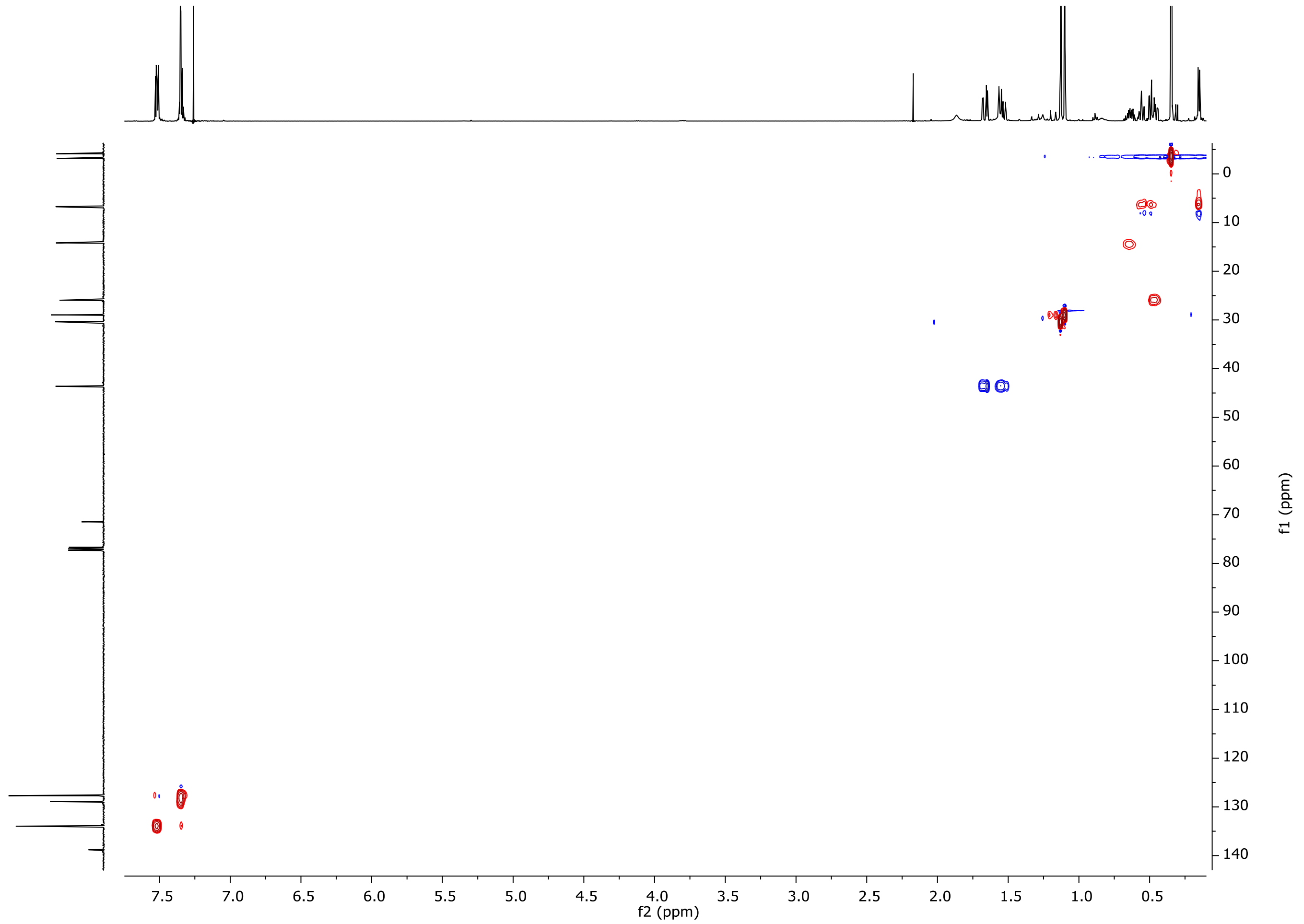

Compound 4a

^{1}H NMR (500 MHz, $CDCl_3$)

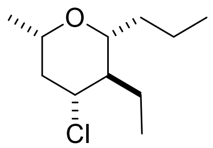

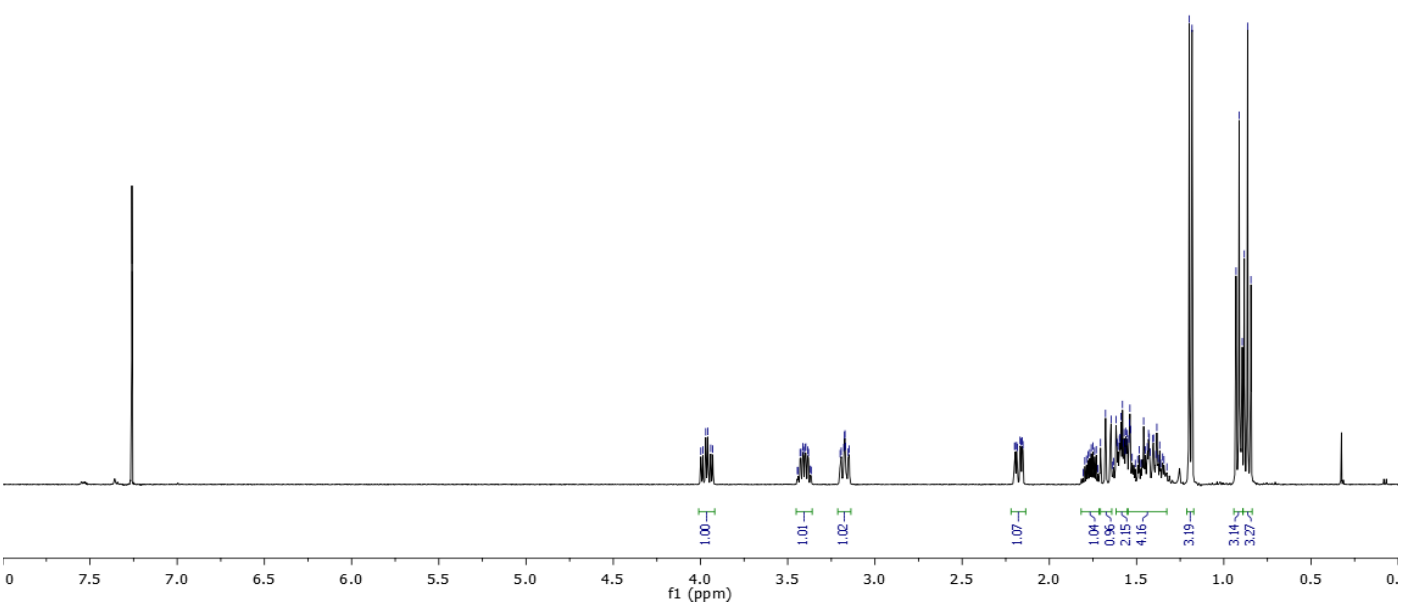

^{13}C NMR (101 MHz, $CDCl_3$)

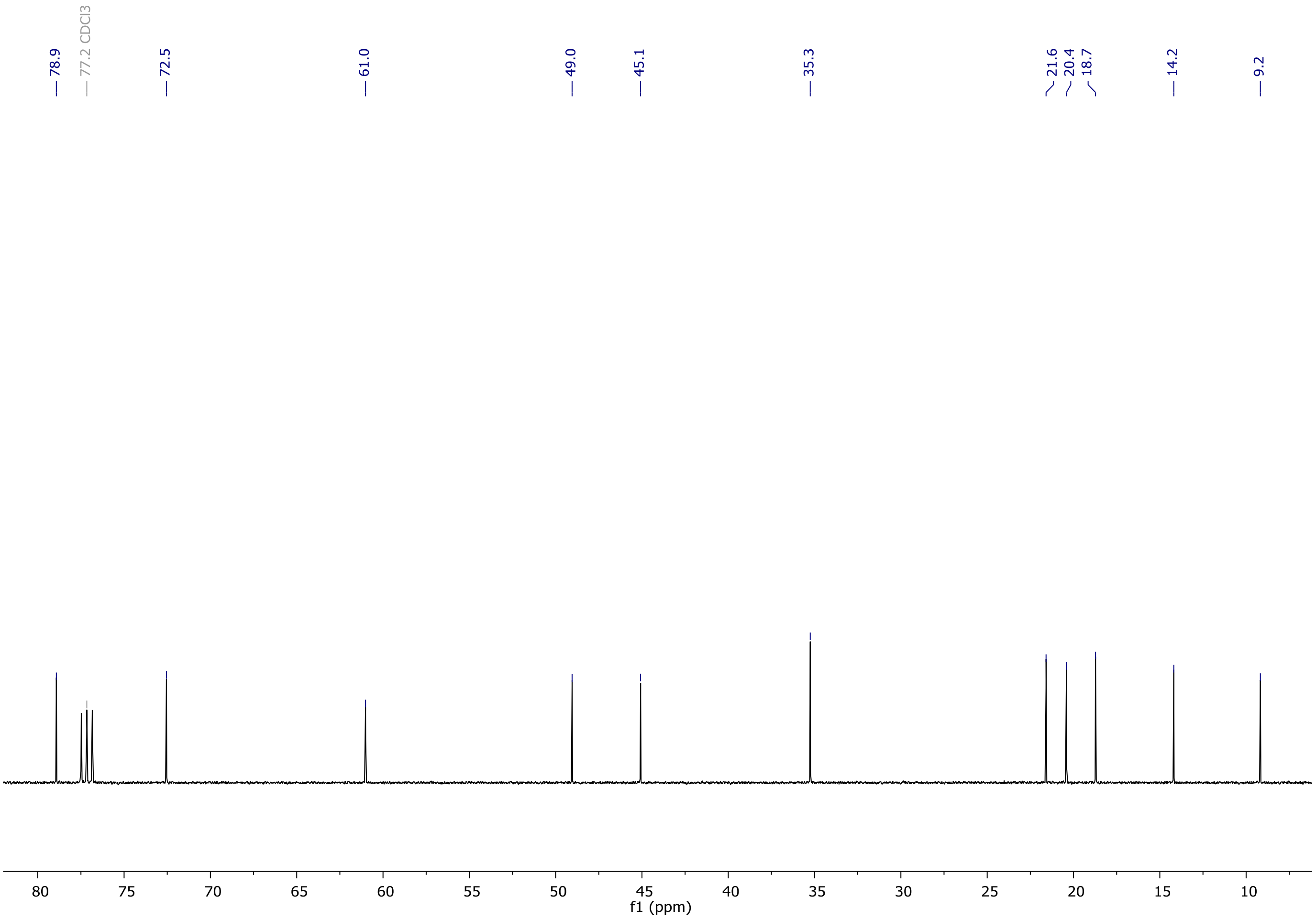

2D-COSY

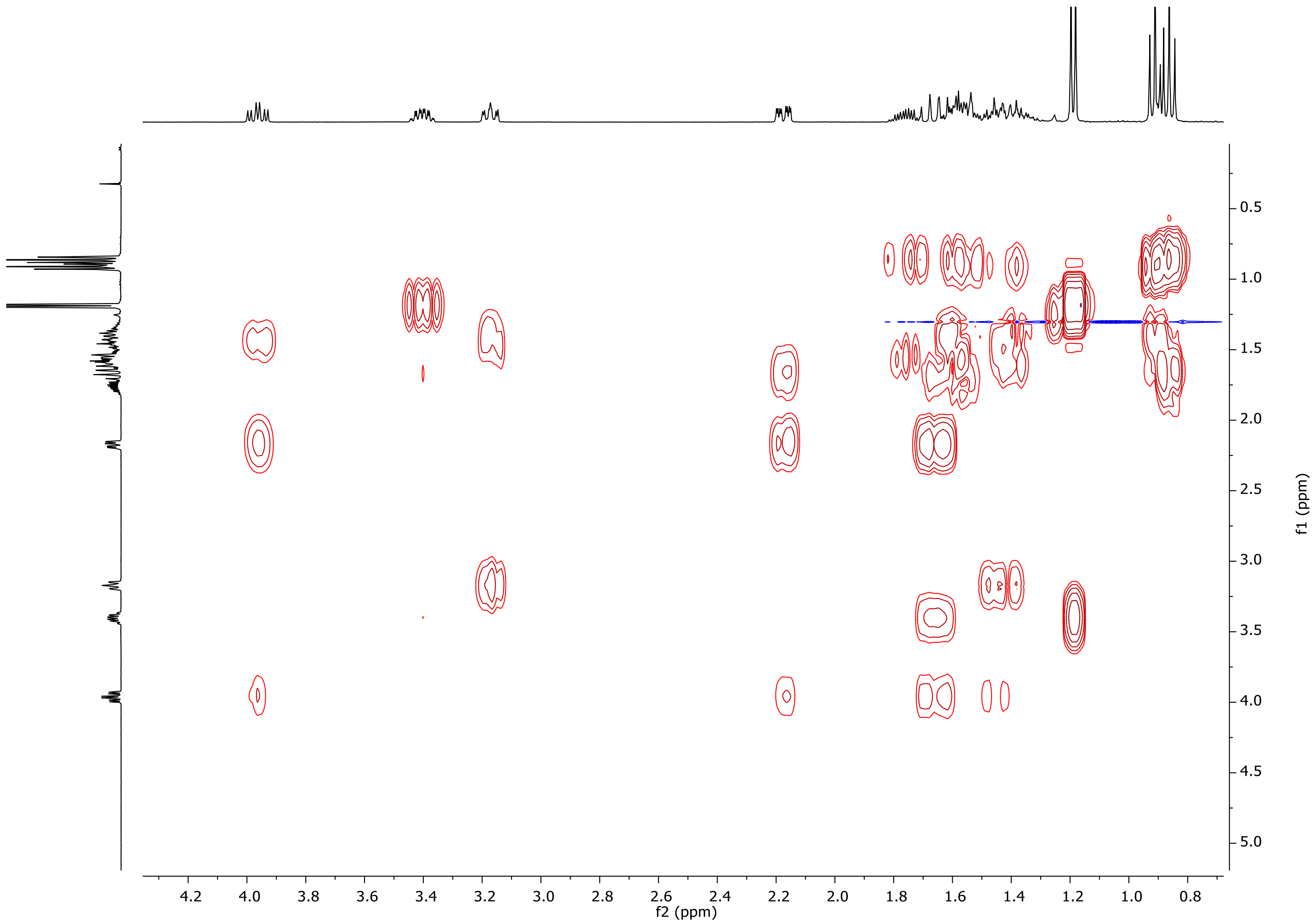

2D-HSQC

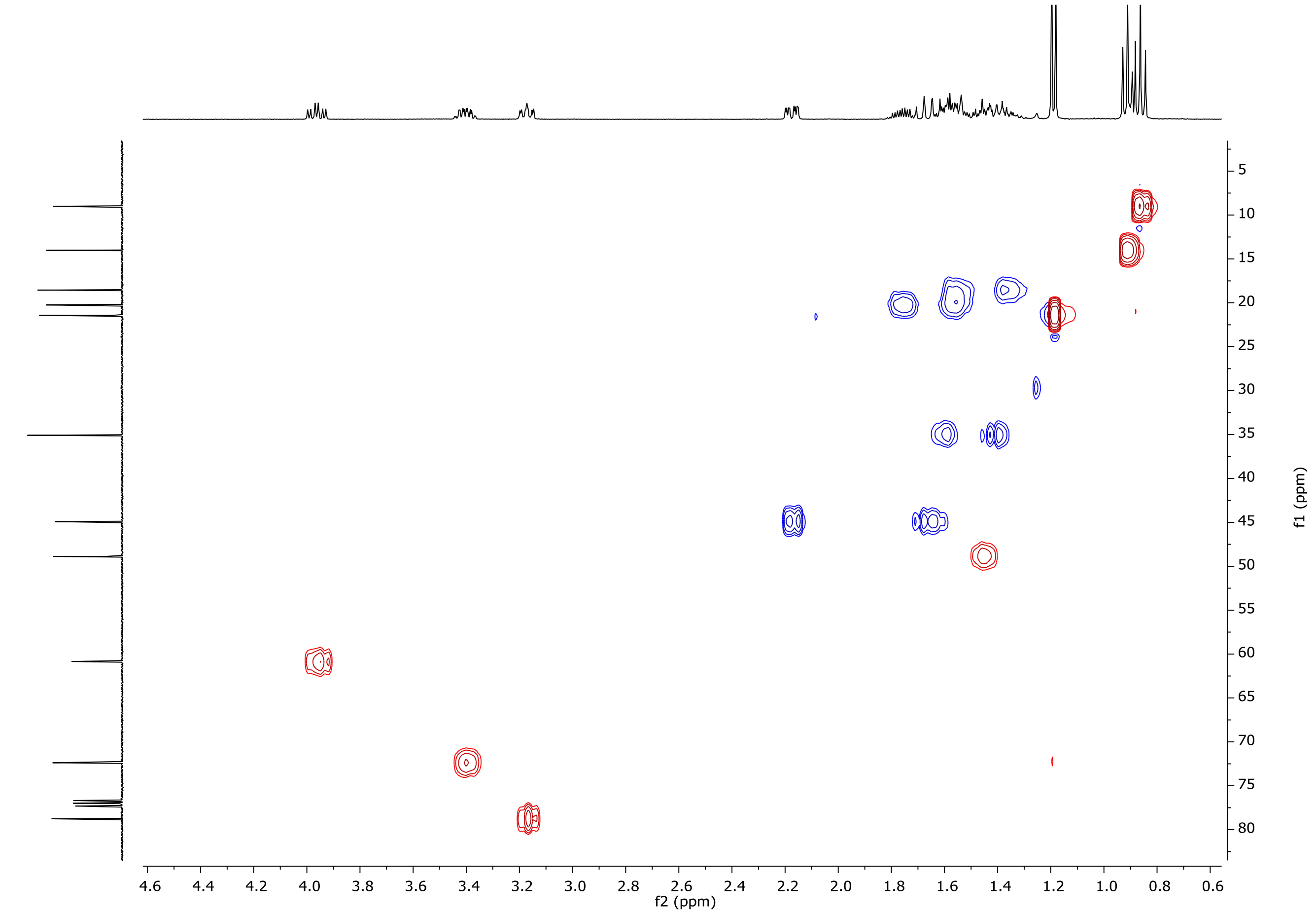

Compound 4b

^{1}H NMR (500 MHz, $CDCl_3$)

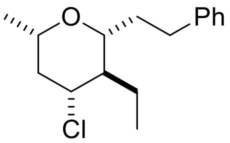

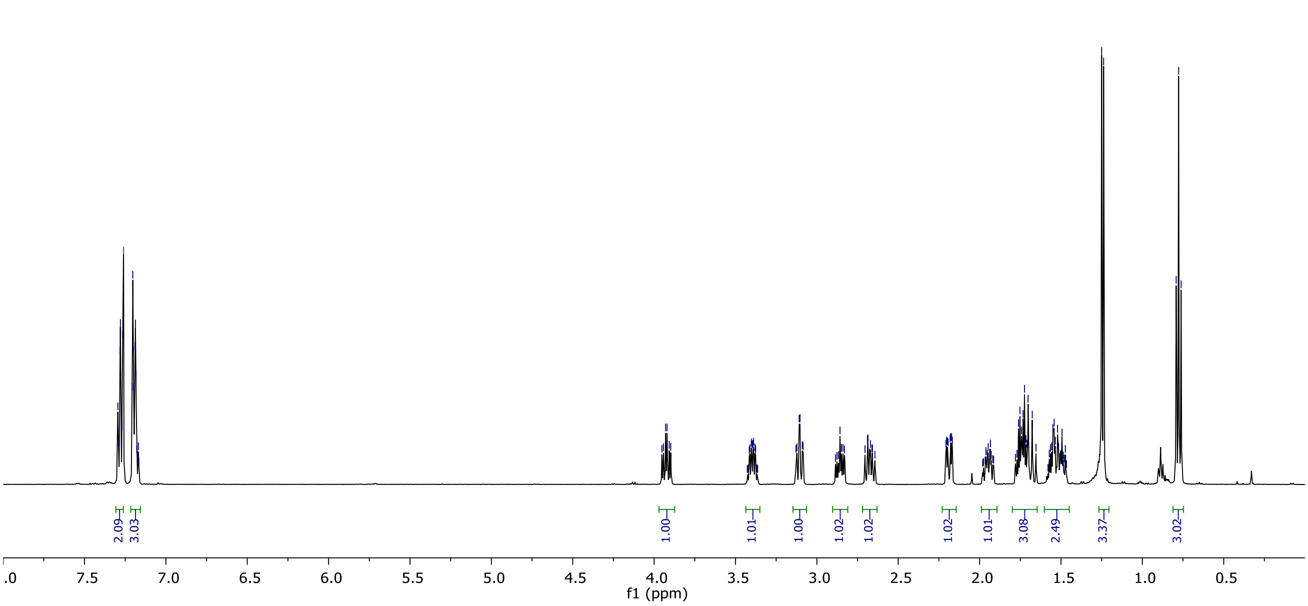

^{13}C NMR (101 MHz, $CDCl_3$)

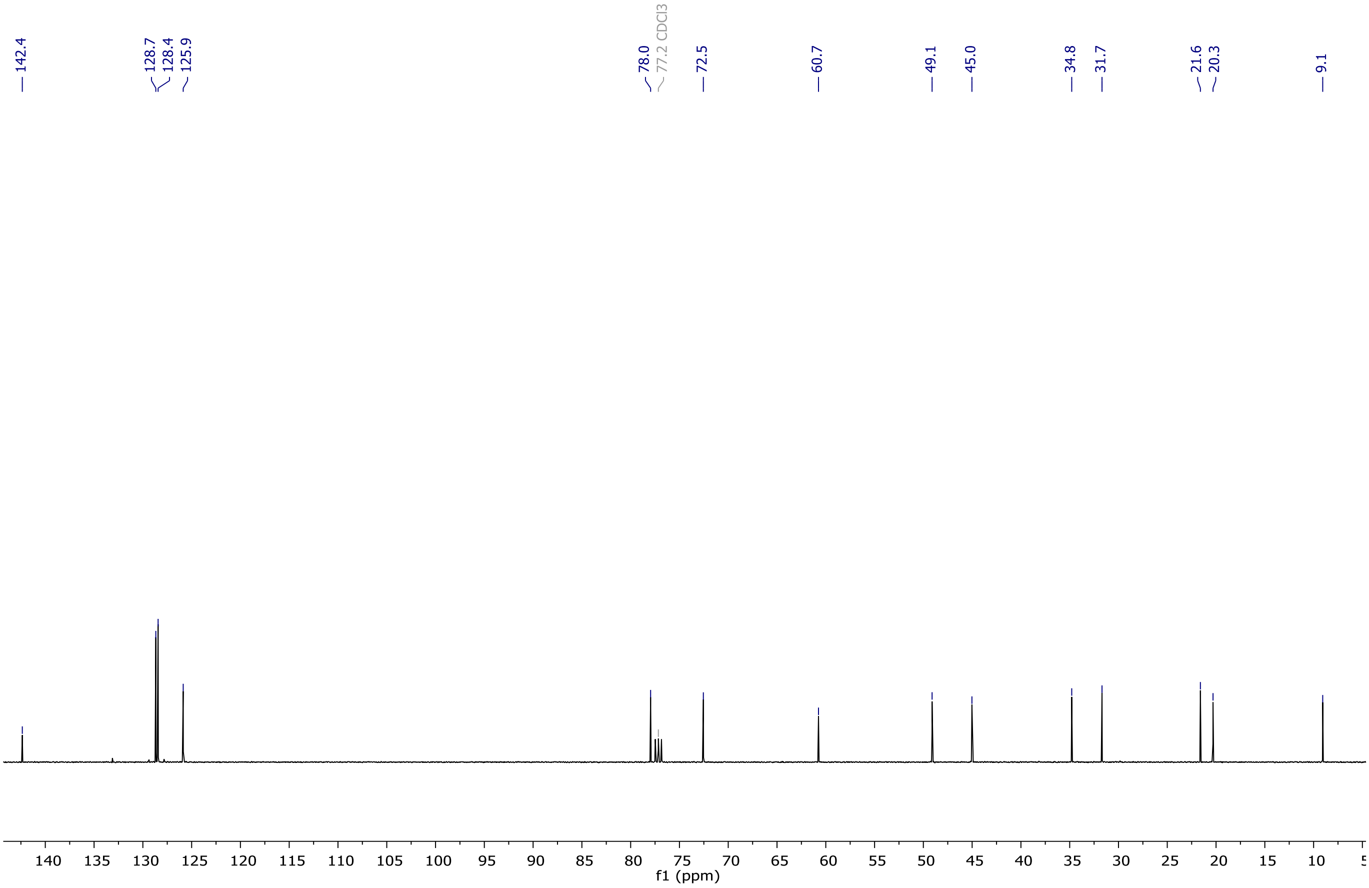

2D-COSY

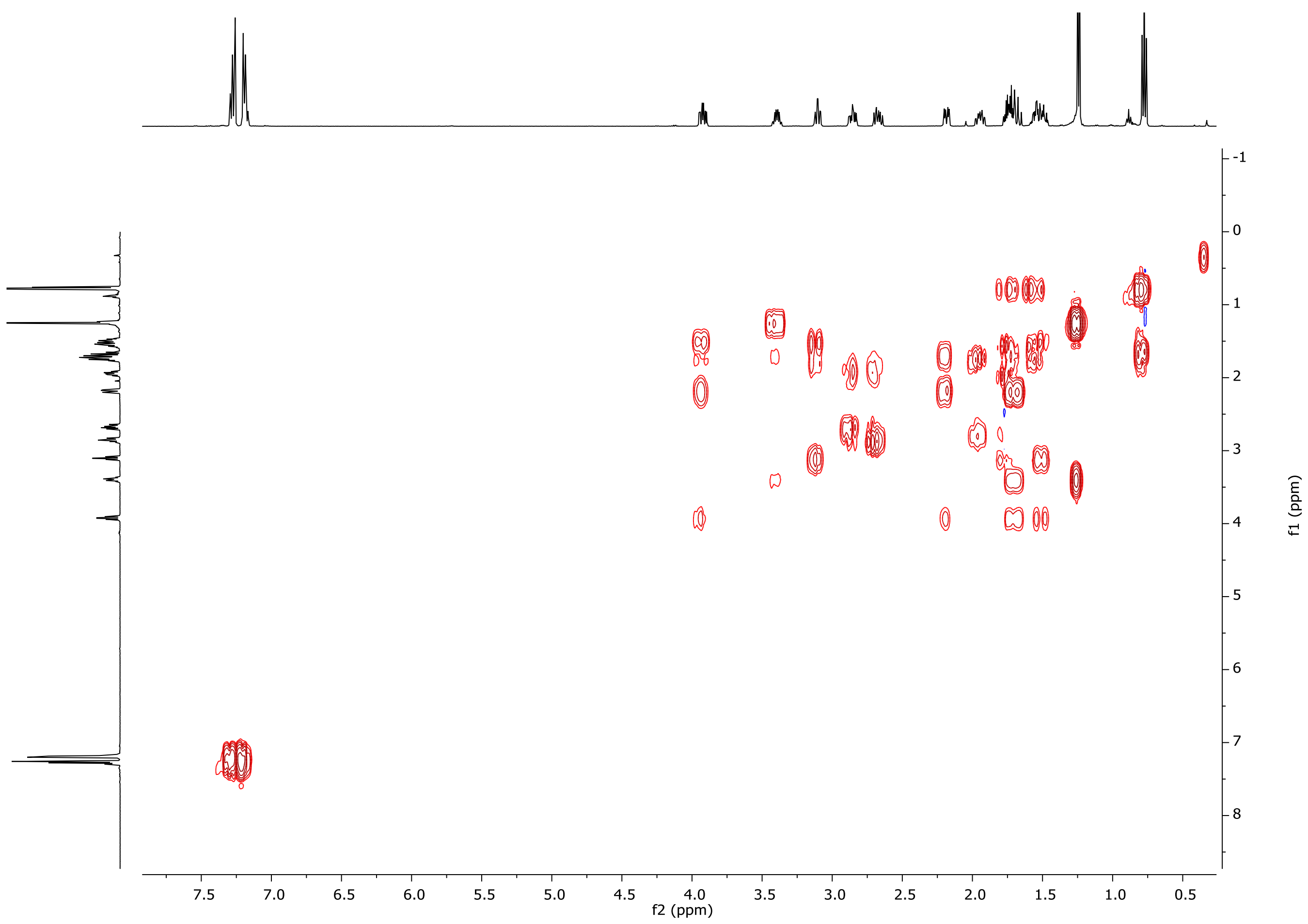

2D-HSQC

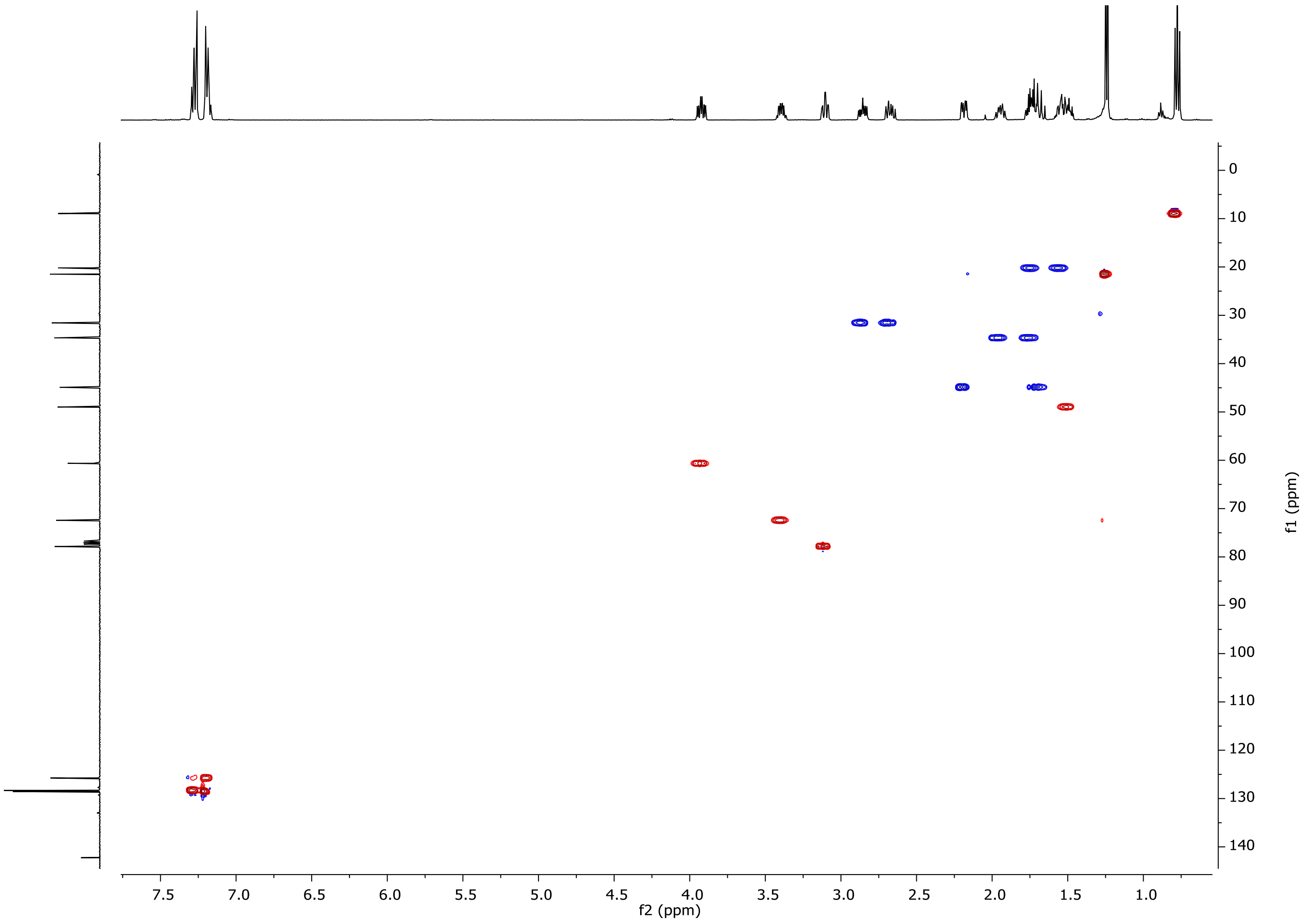

Compound 4c

^{1}H NMR (500 MHz, $CDCl_3$)

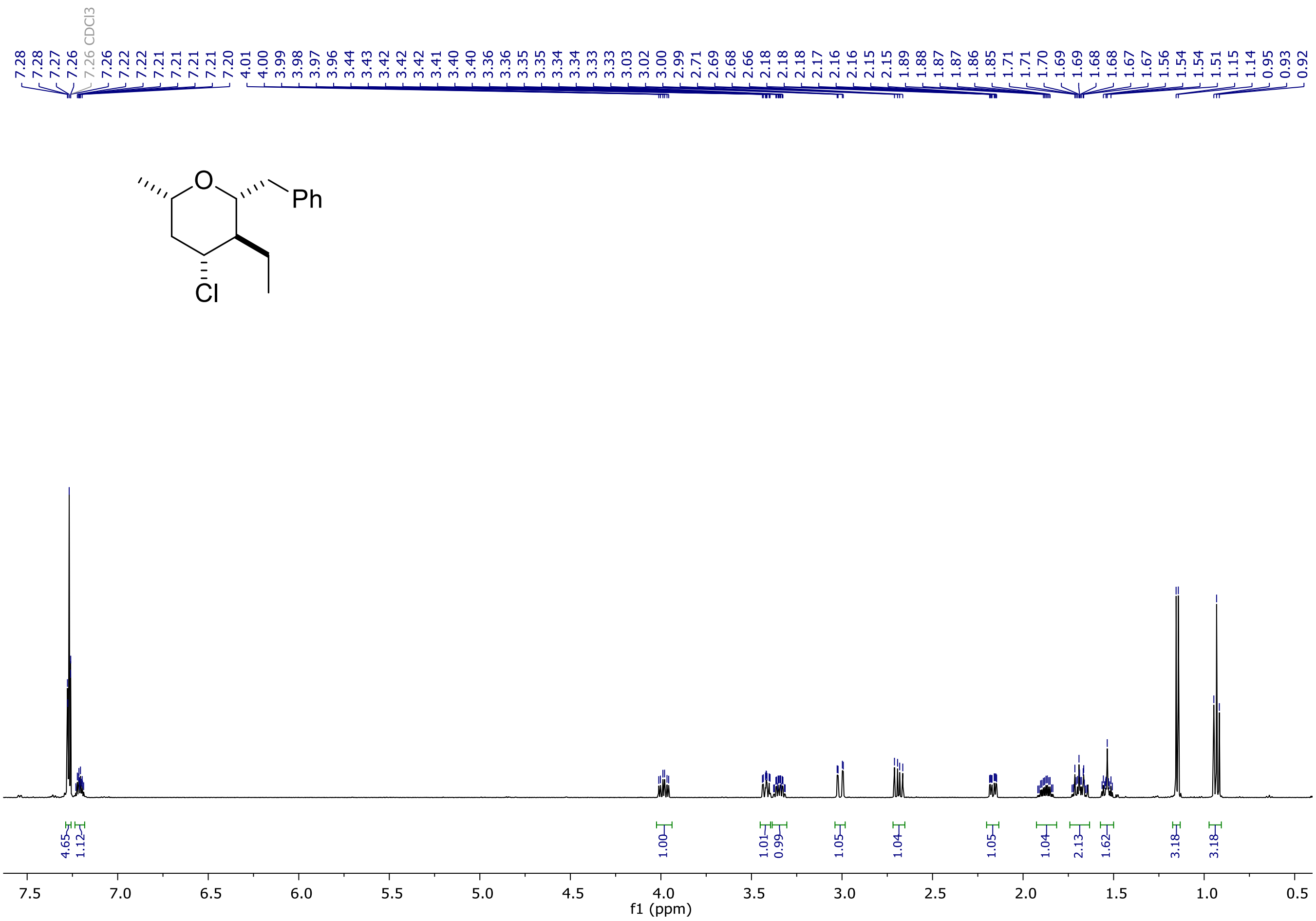

^{13}C NMR (101 MHz, $CDCl_3$)

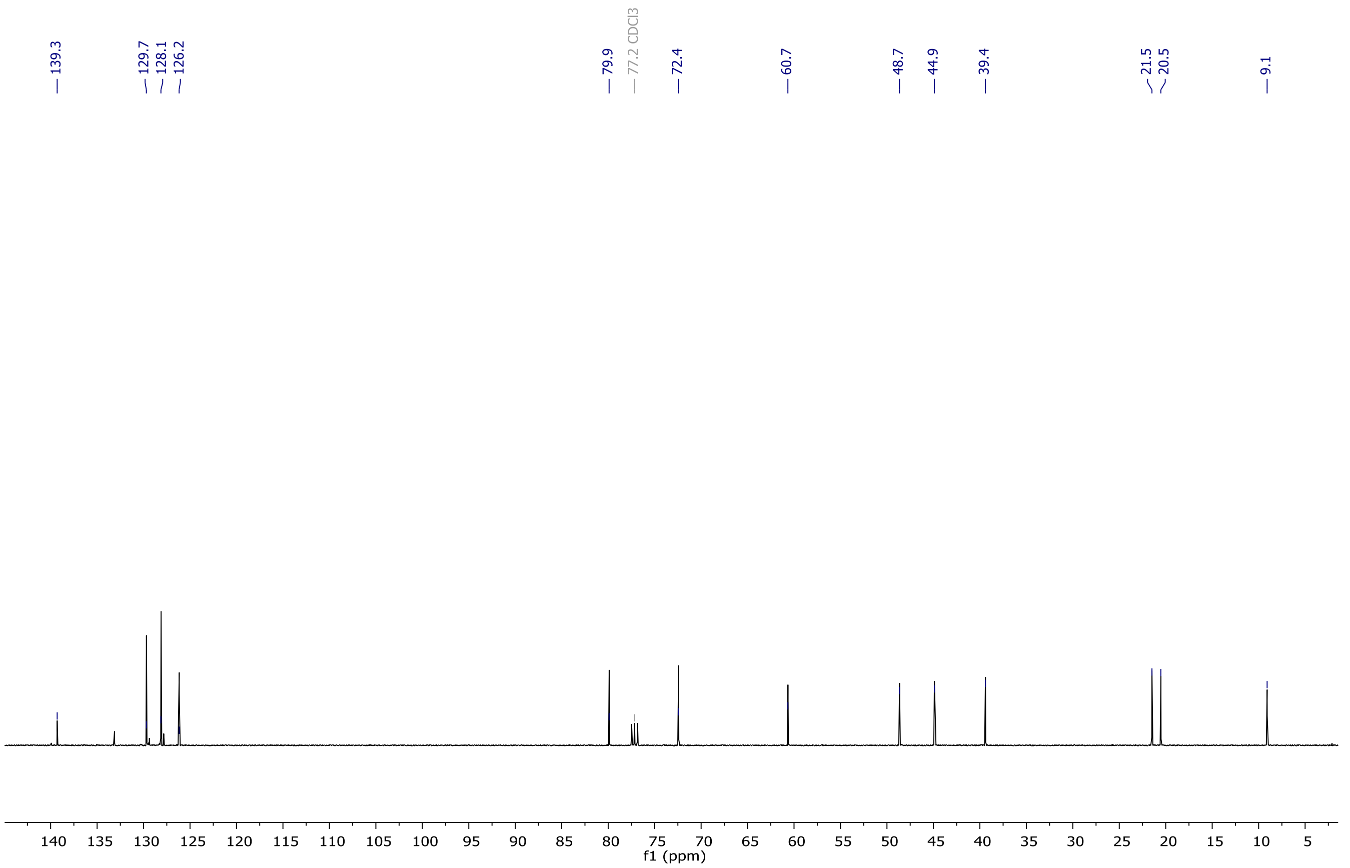

2D-COSY

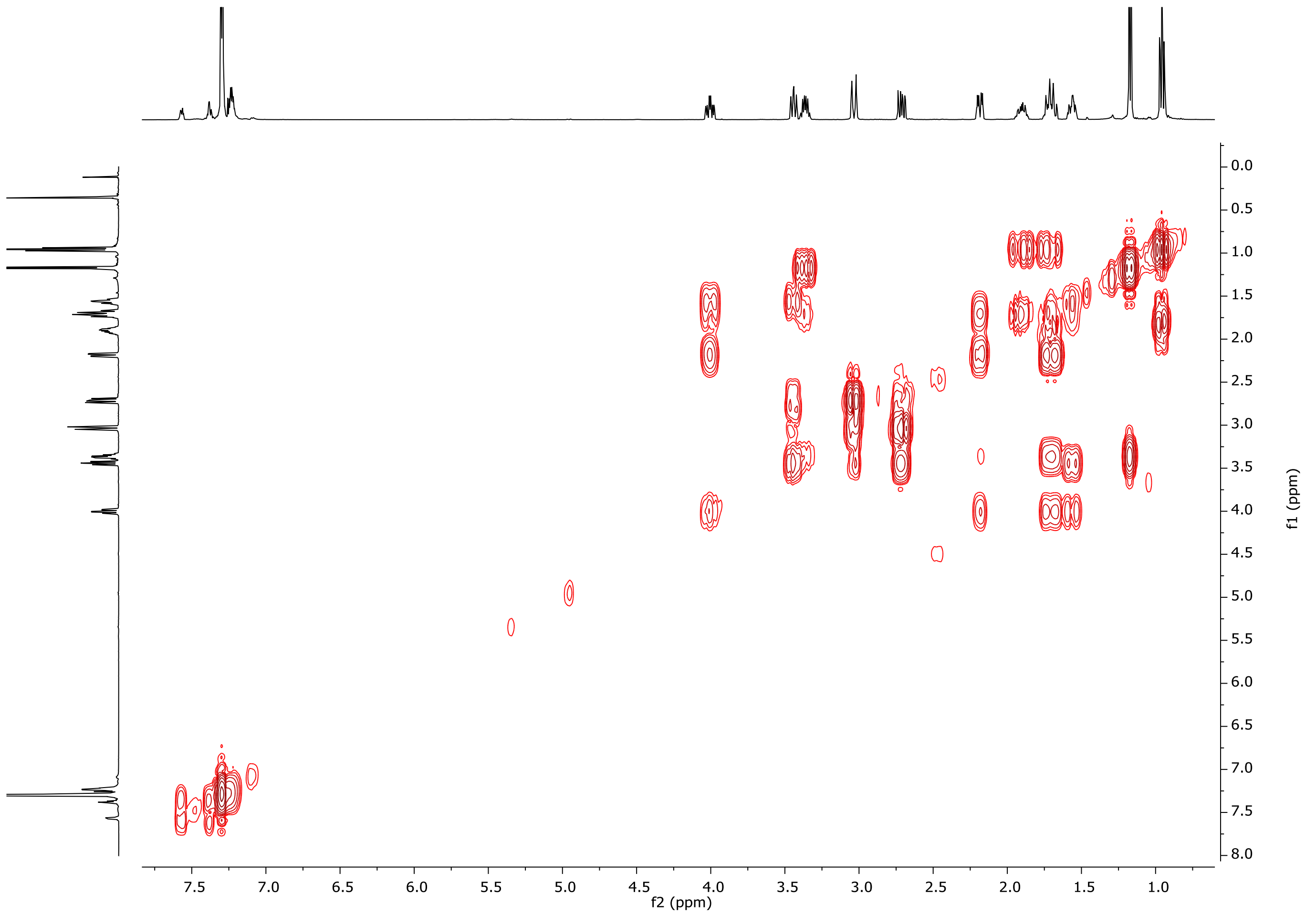

2D-HSQC

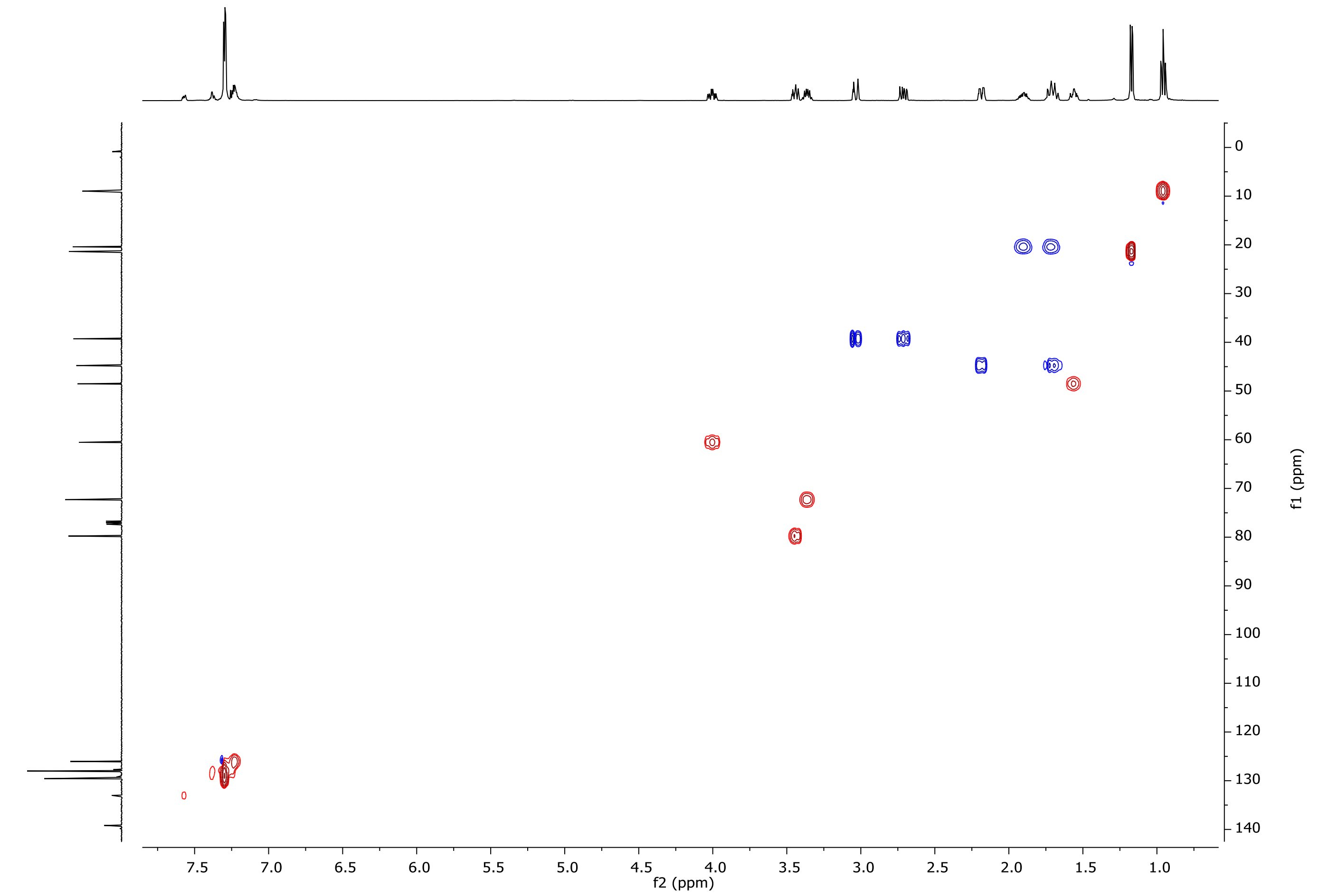

Compound 4d

^{1}**H NMR** (500 MHz, $CDCl_3$)

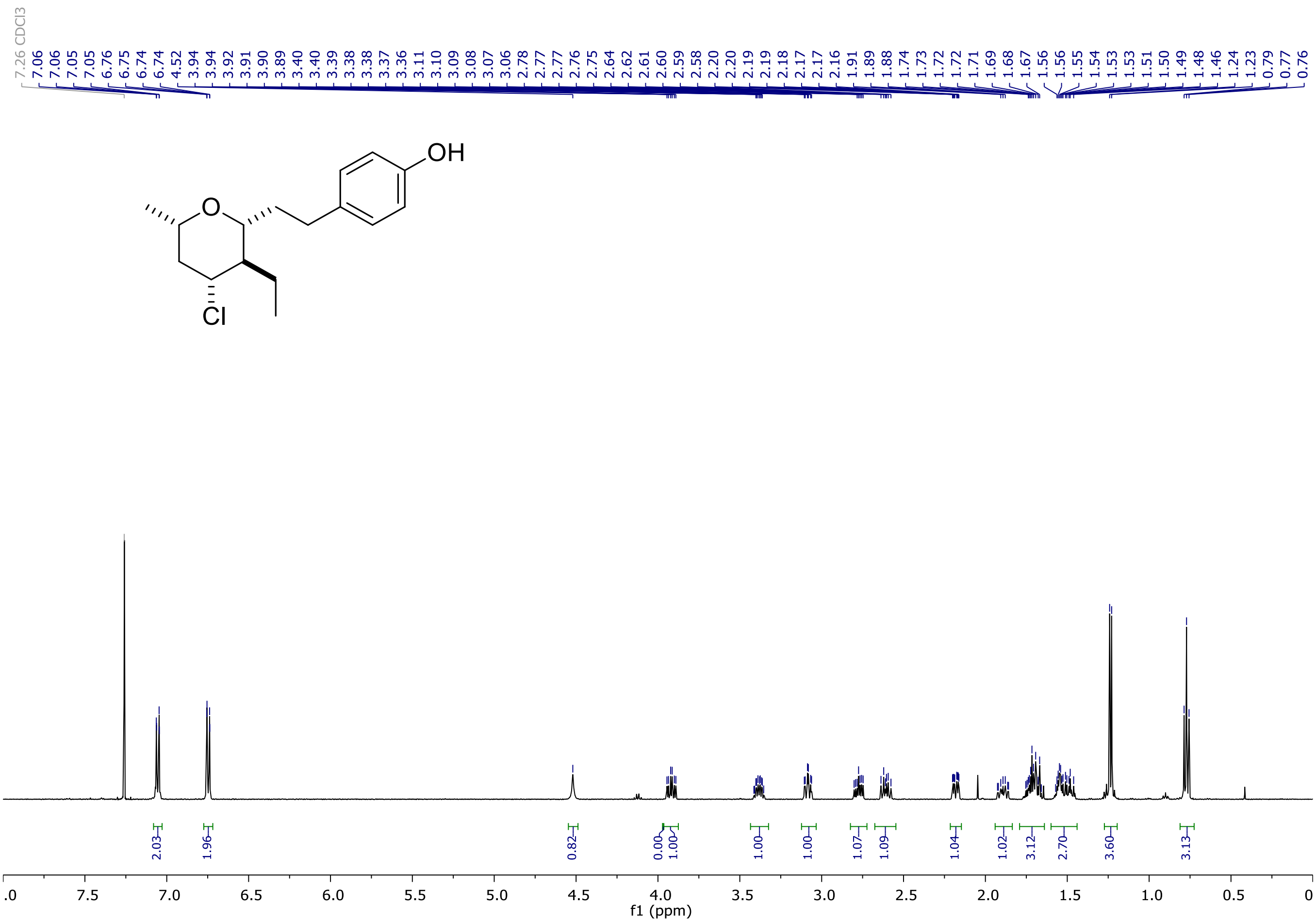

^{13}**C NMR** (101 MHz, $CDCl_3$)

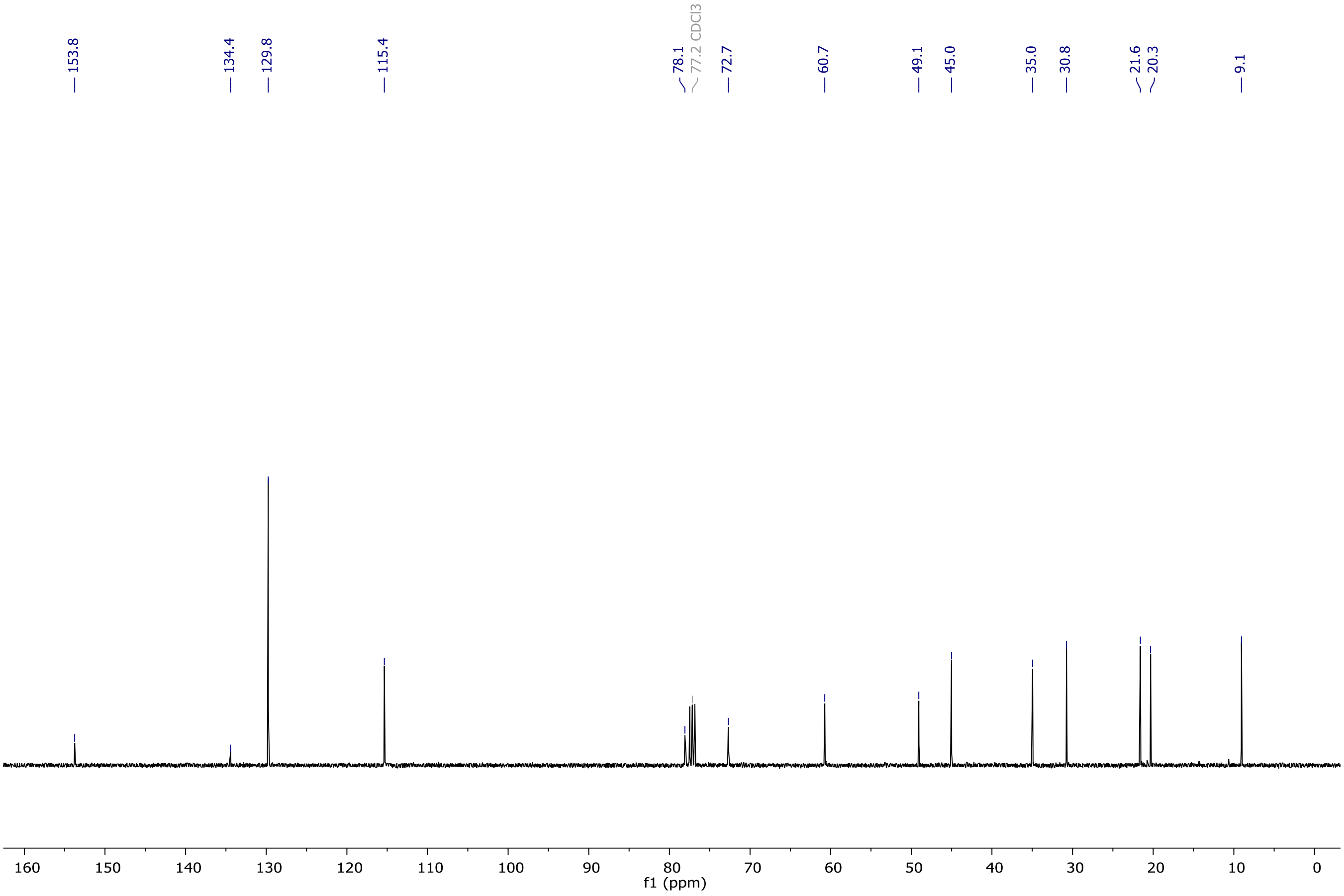

2D-COSY

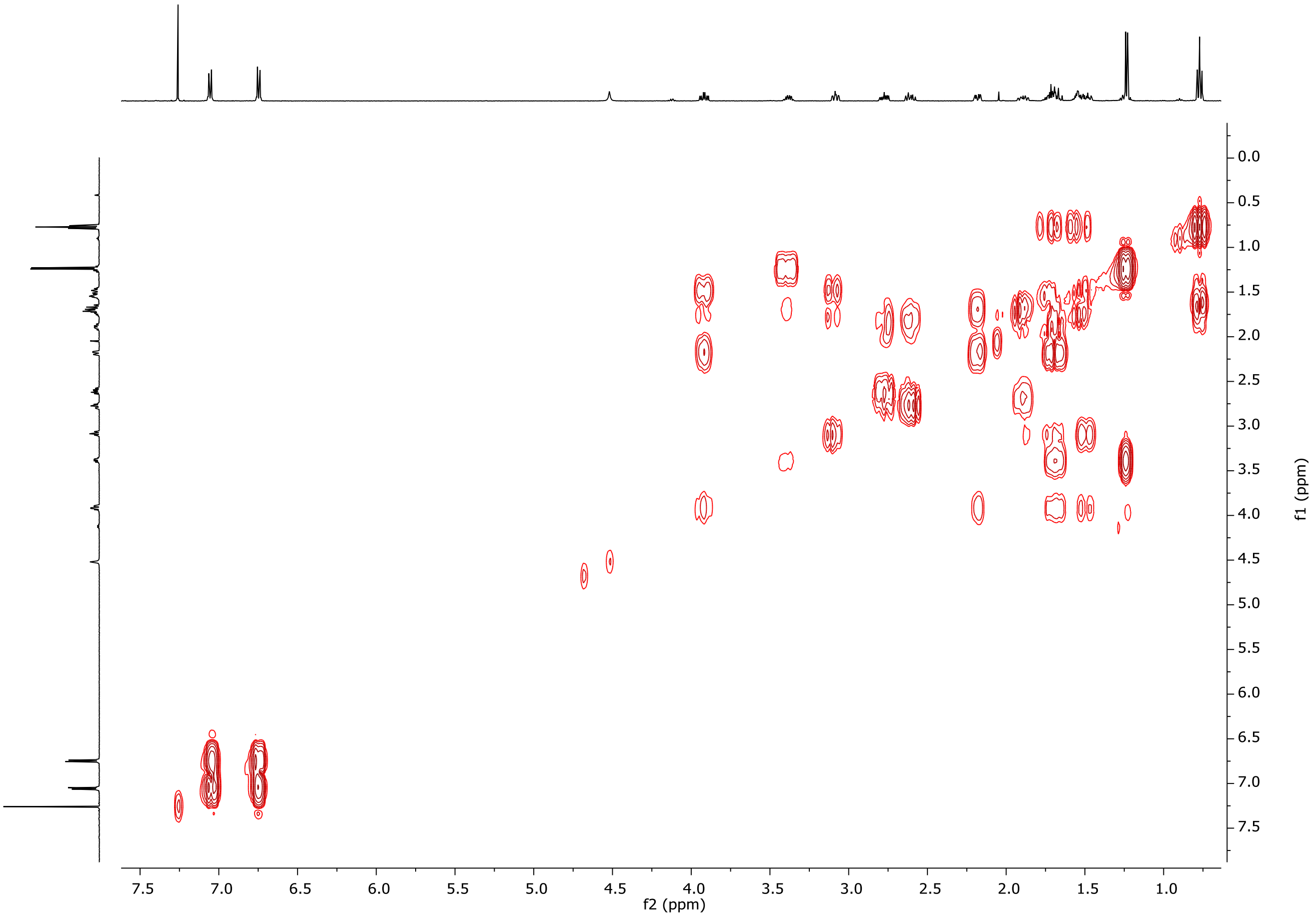

2D-HSQC

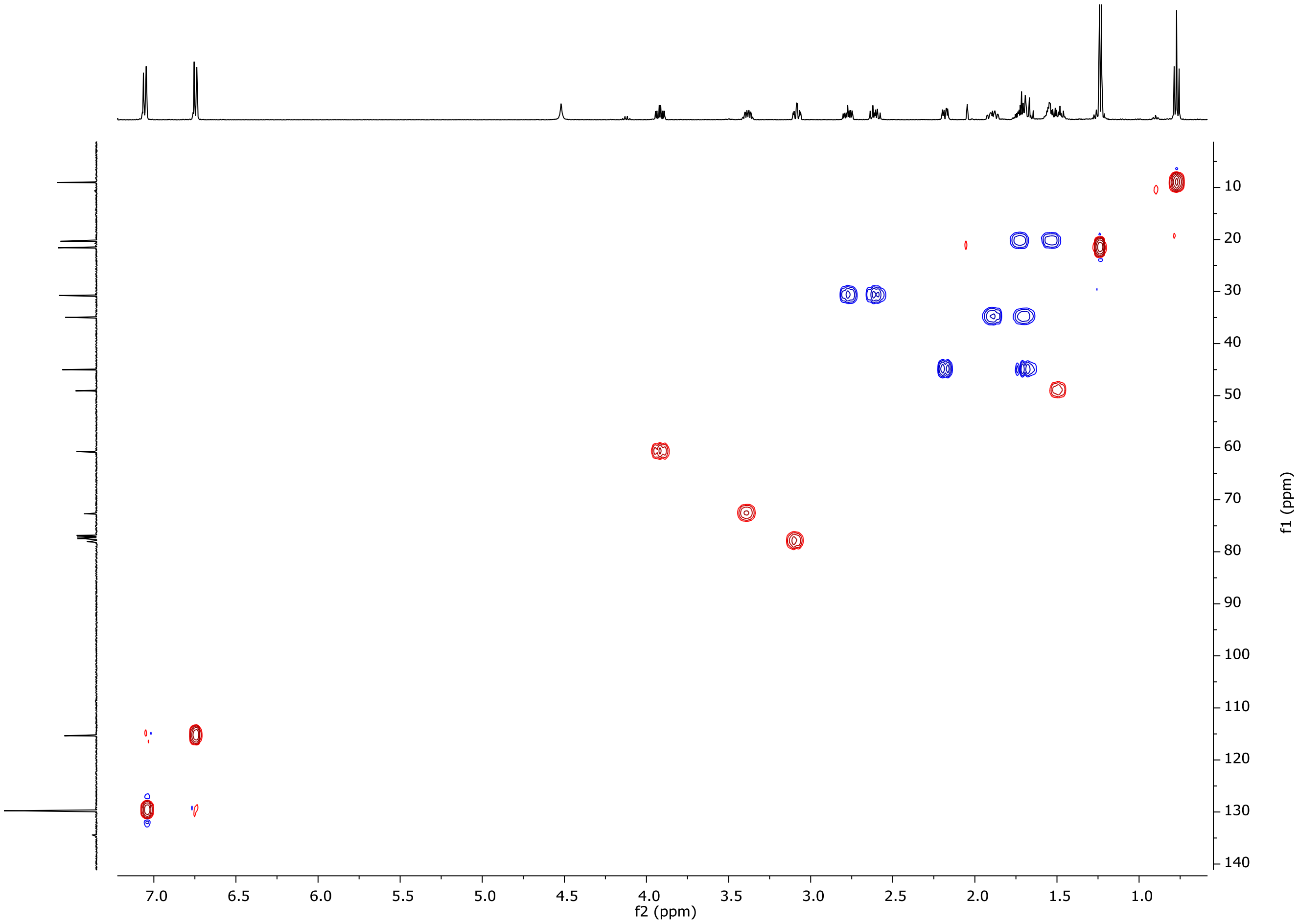

Compound 4e

^{1}H NMR (500 MHz, $CDCl_3$)

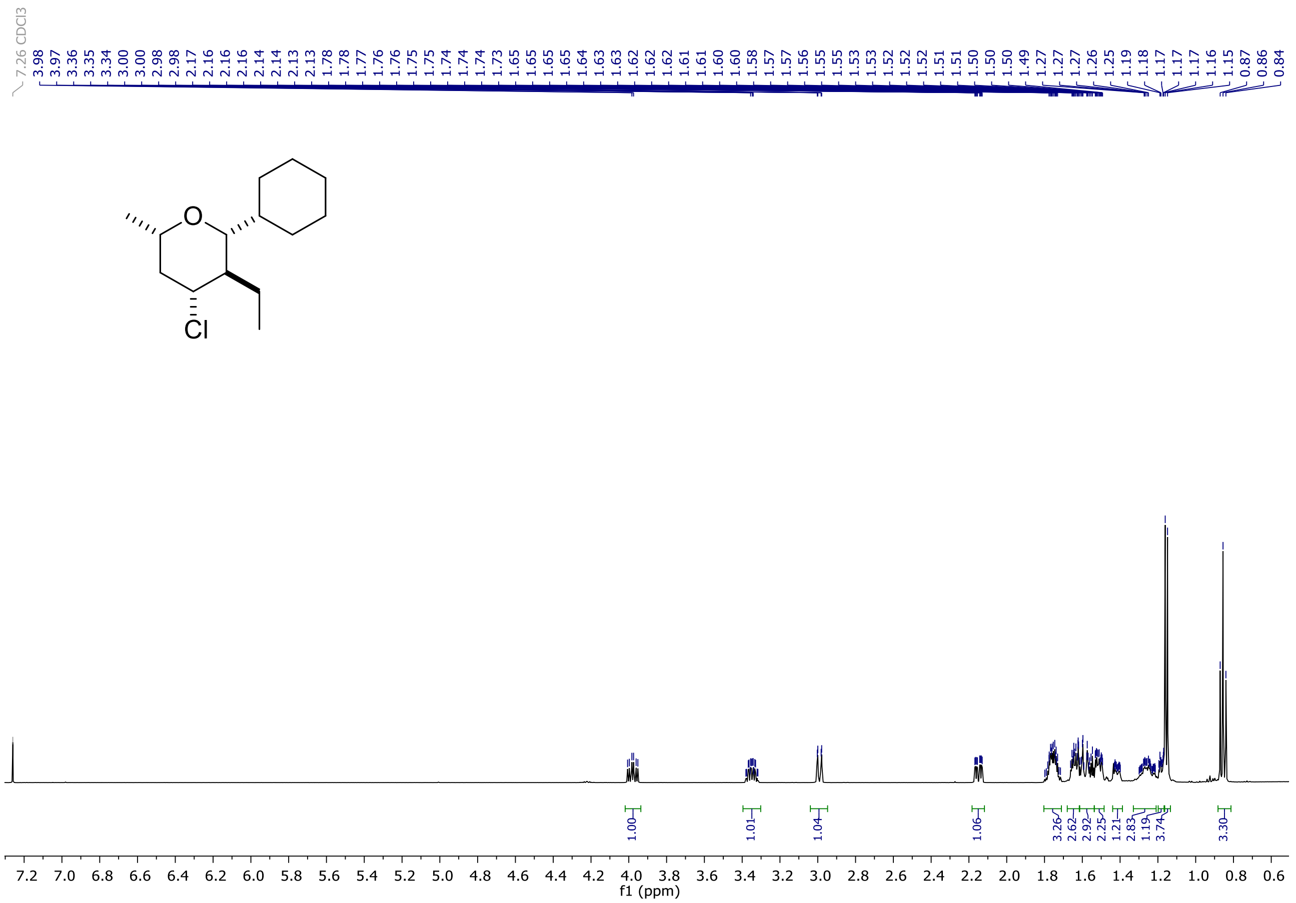

^{13}C NMR (101 MHz, $CDCl_3$)

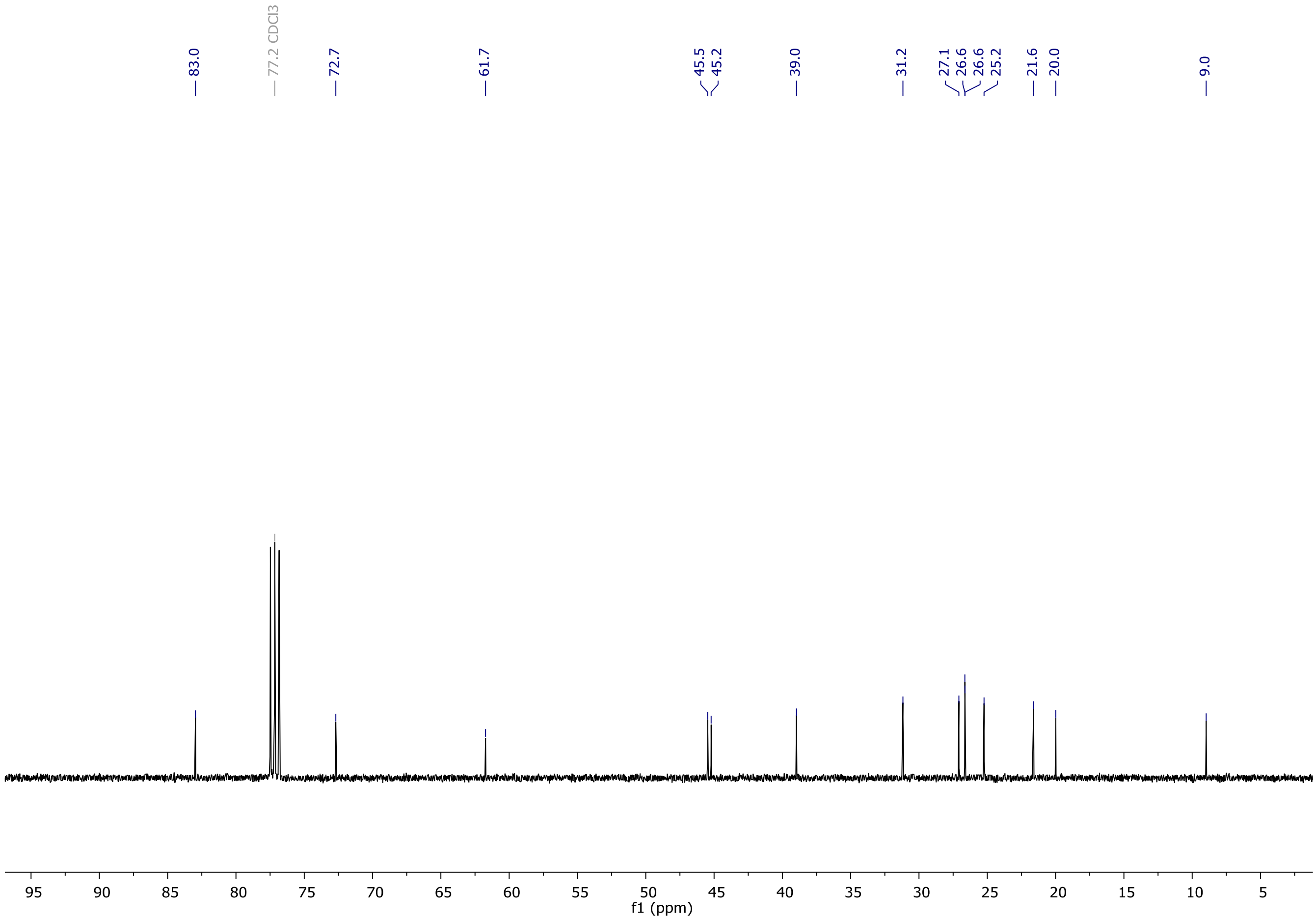

2D-COSY

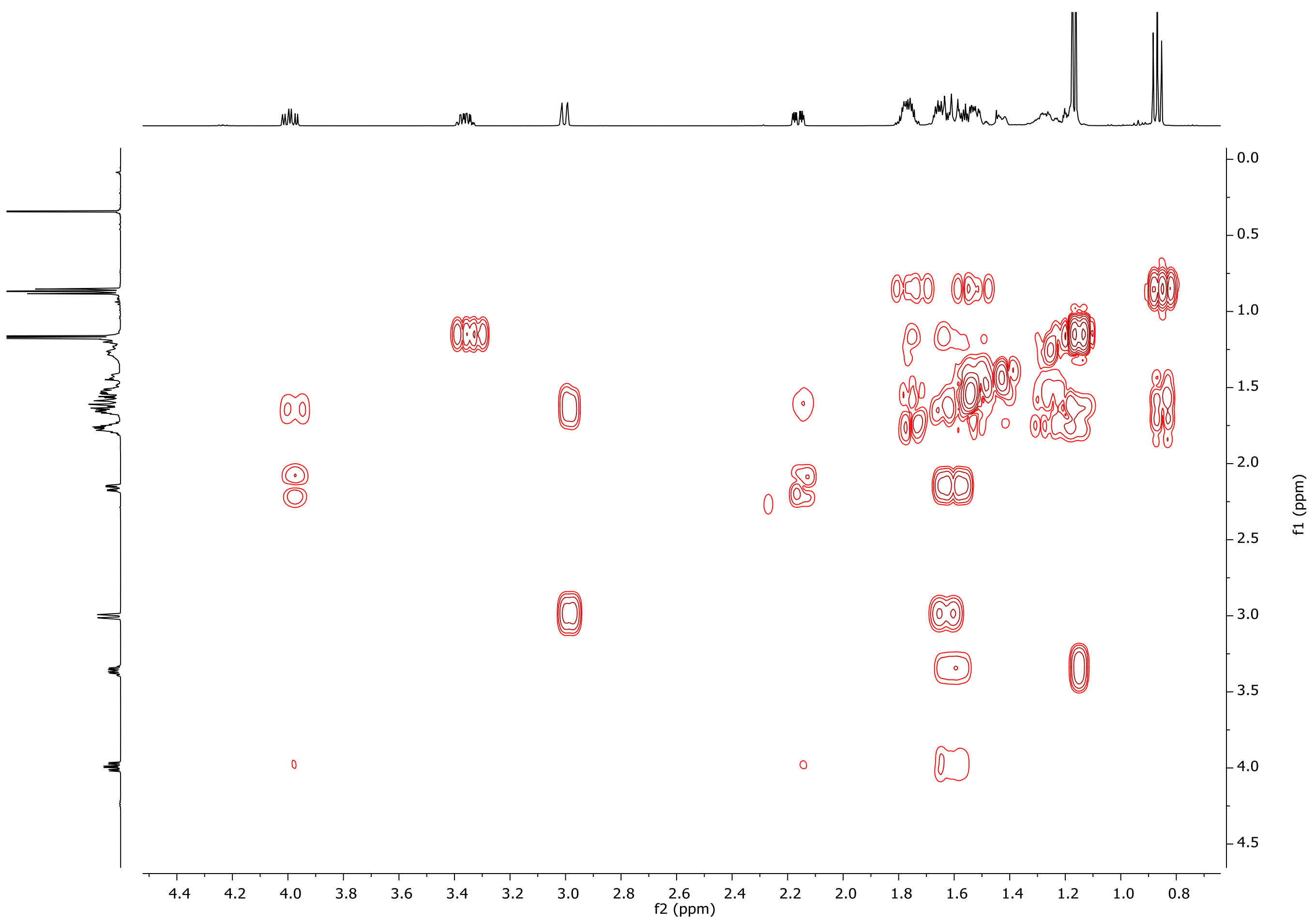

2D-HSQC

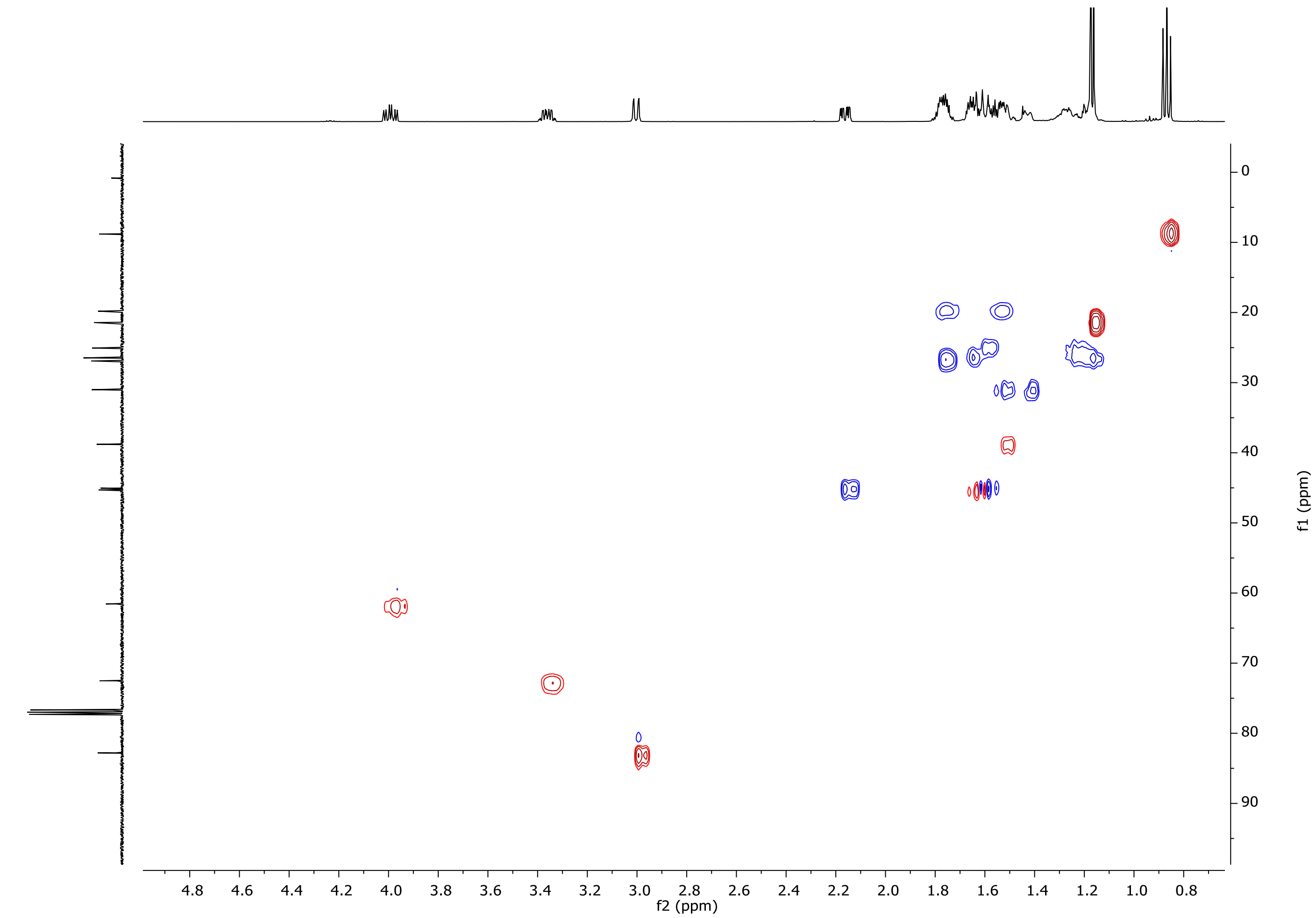

Compound 4f

^{1}H NMR (500 MHz, $CDCl_3$)

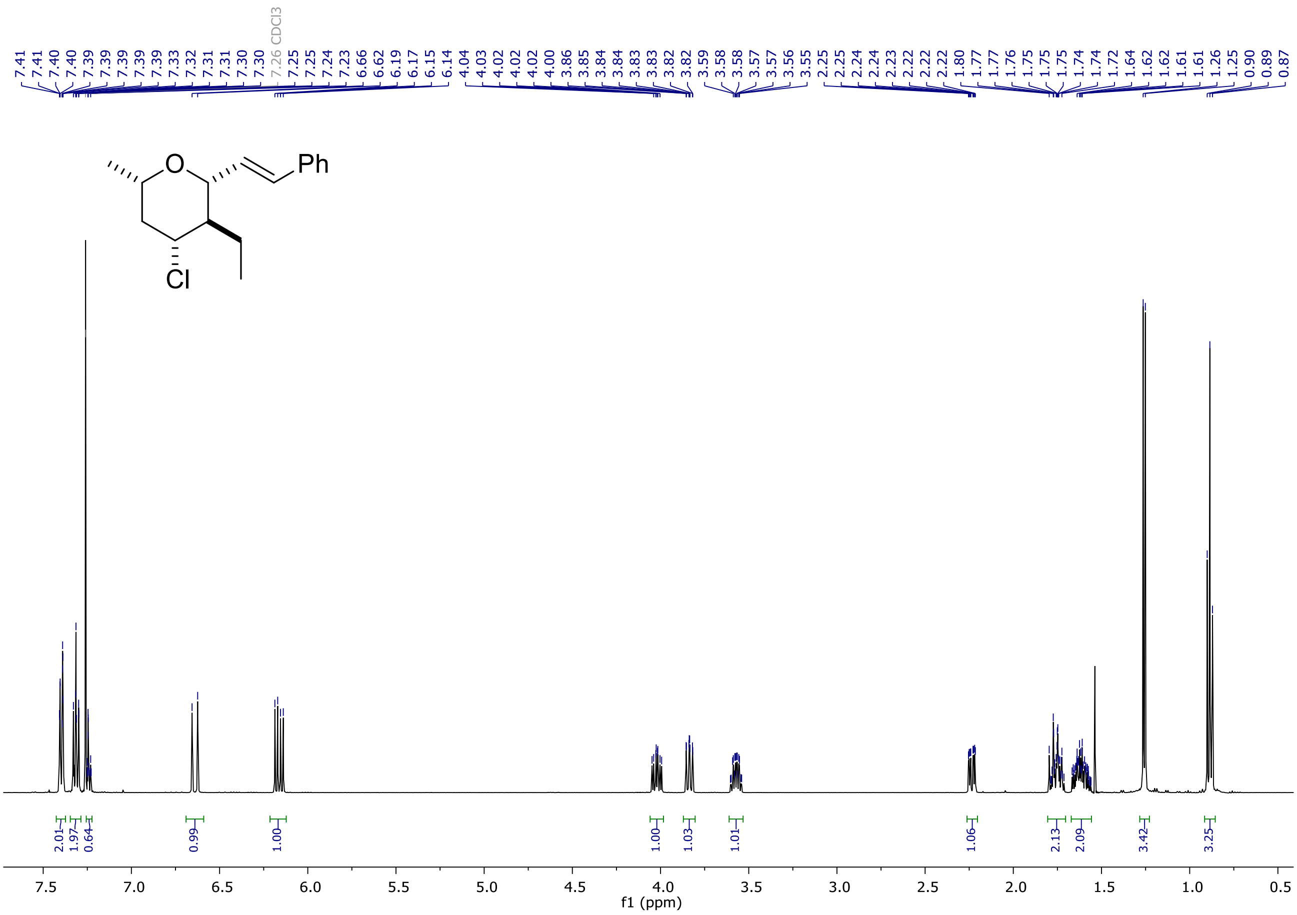

^{13}C NMR (101 MHz, $CDCl_3$)

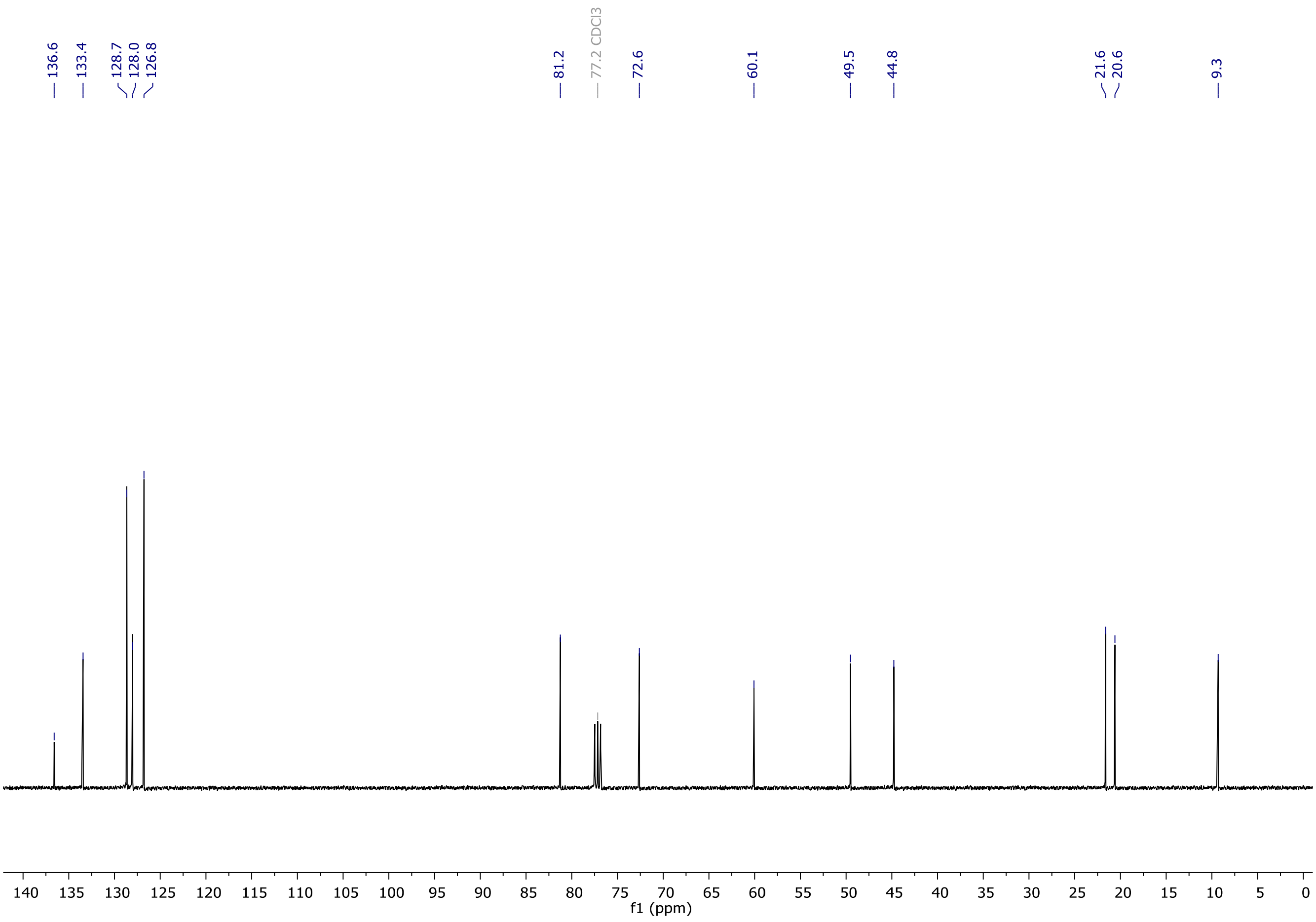

2D-COSY

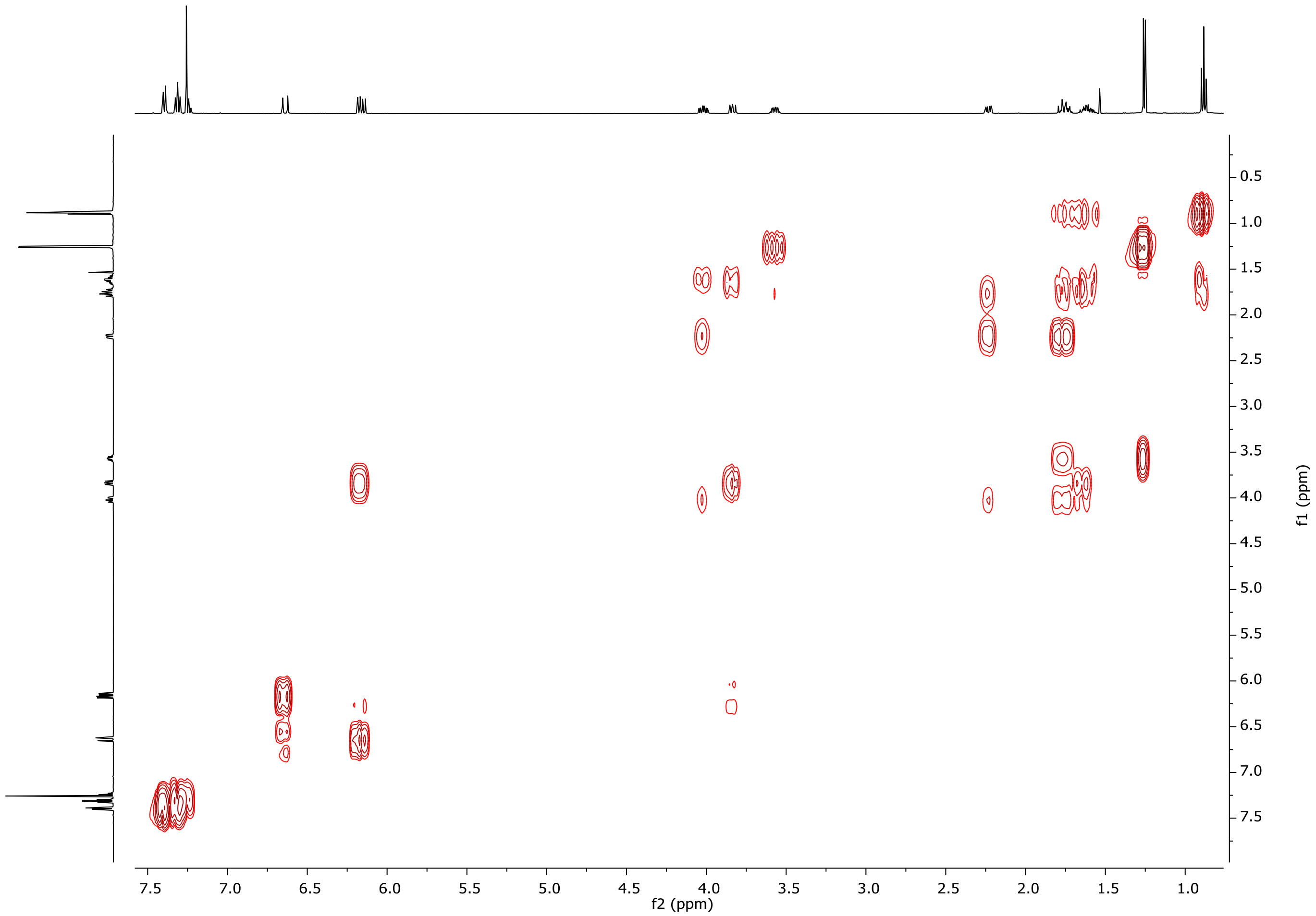

2D-HSQC

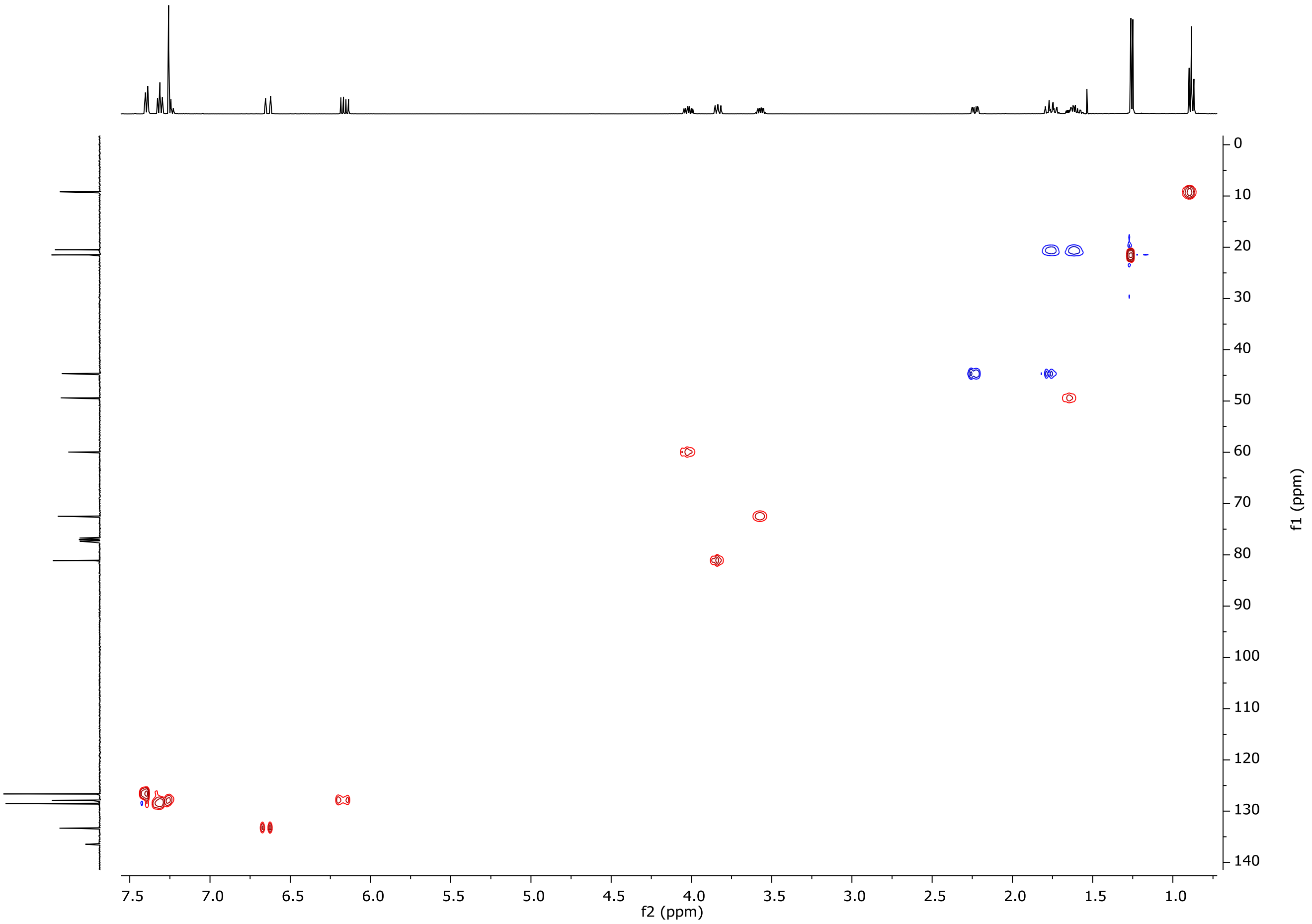

Compound 4g

[1]**H NMR** (500 MHz, $CDCl_3$)

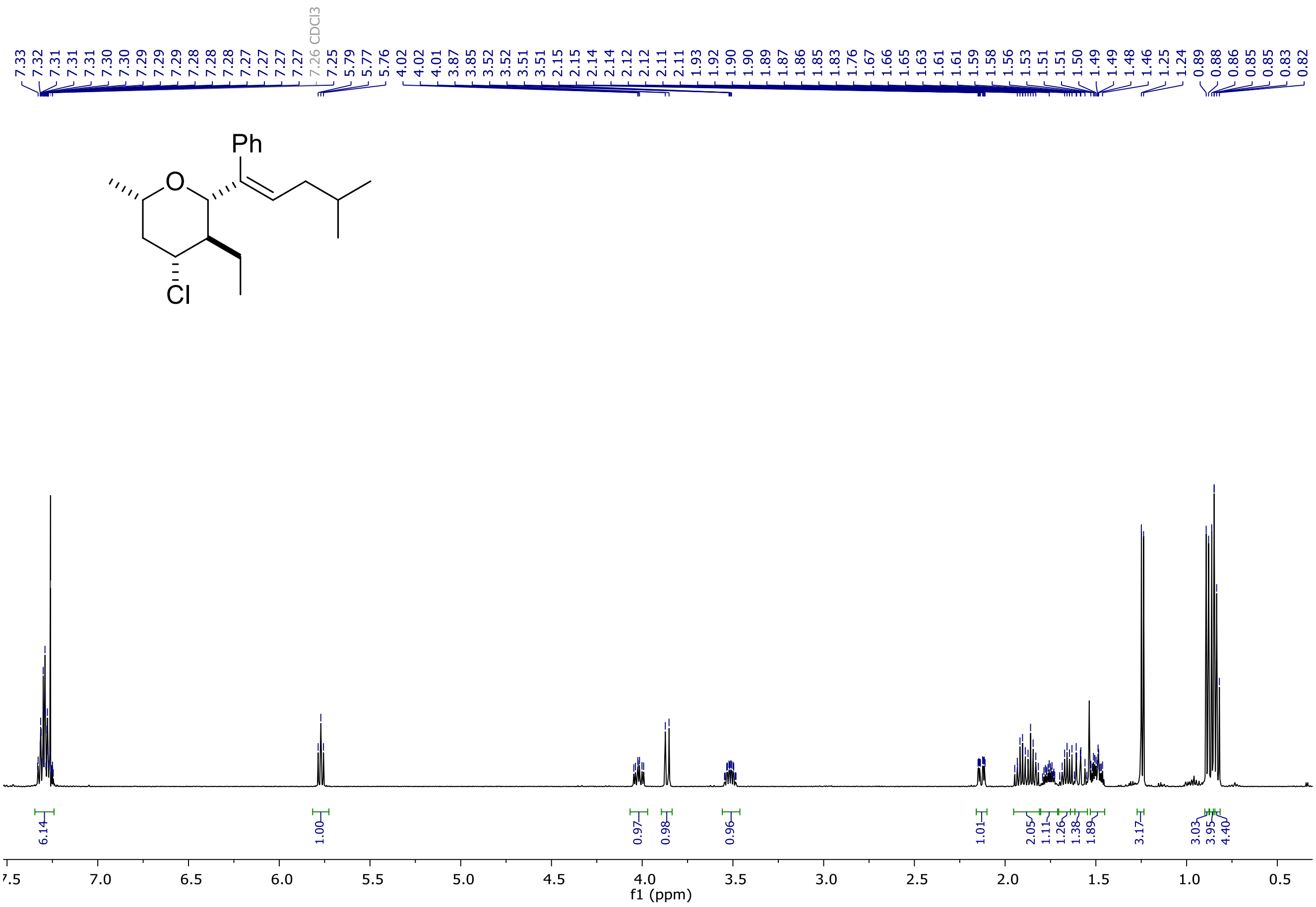

[13]**C NMR** (101 MHz, $CDCl_3$)

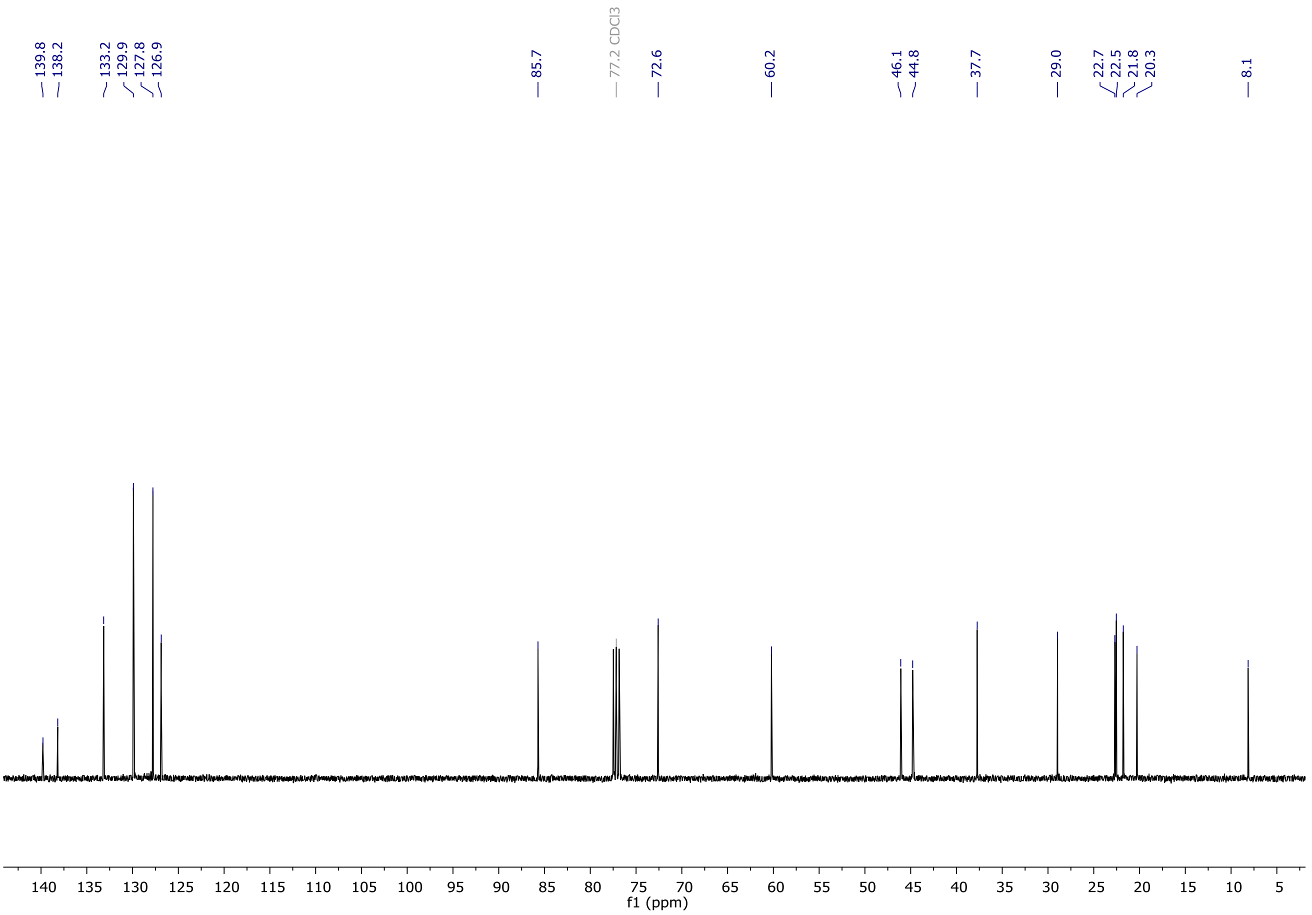

2D-COSY

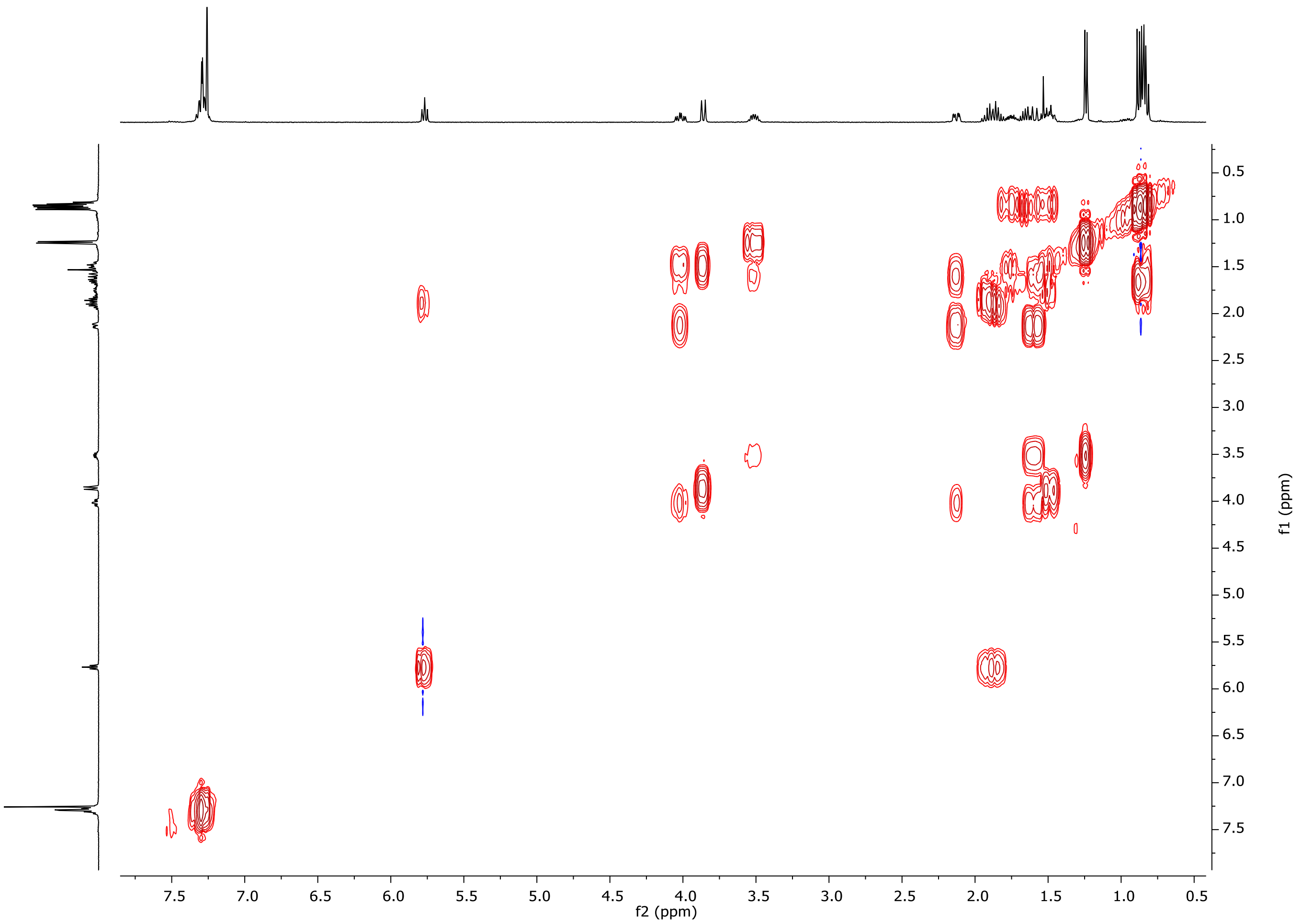

2D-HSQC

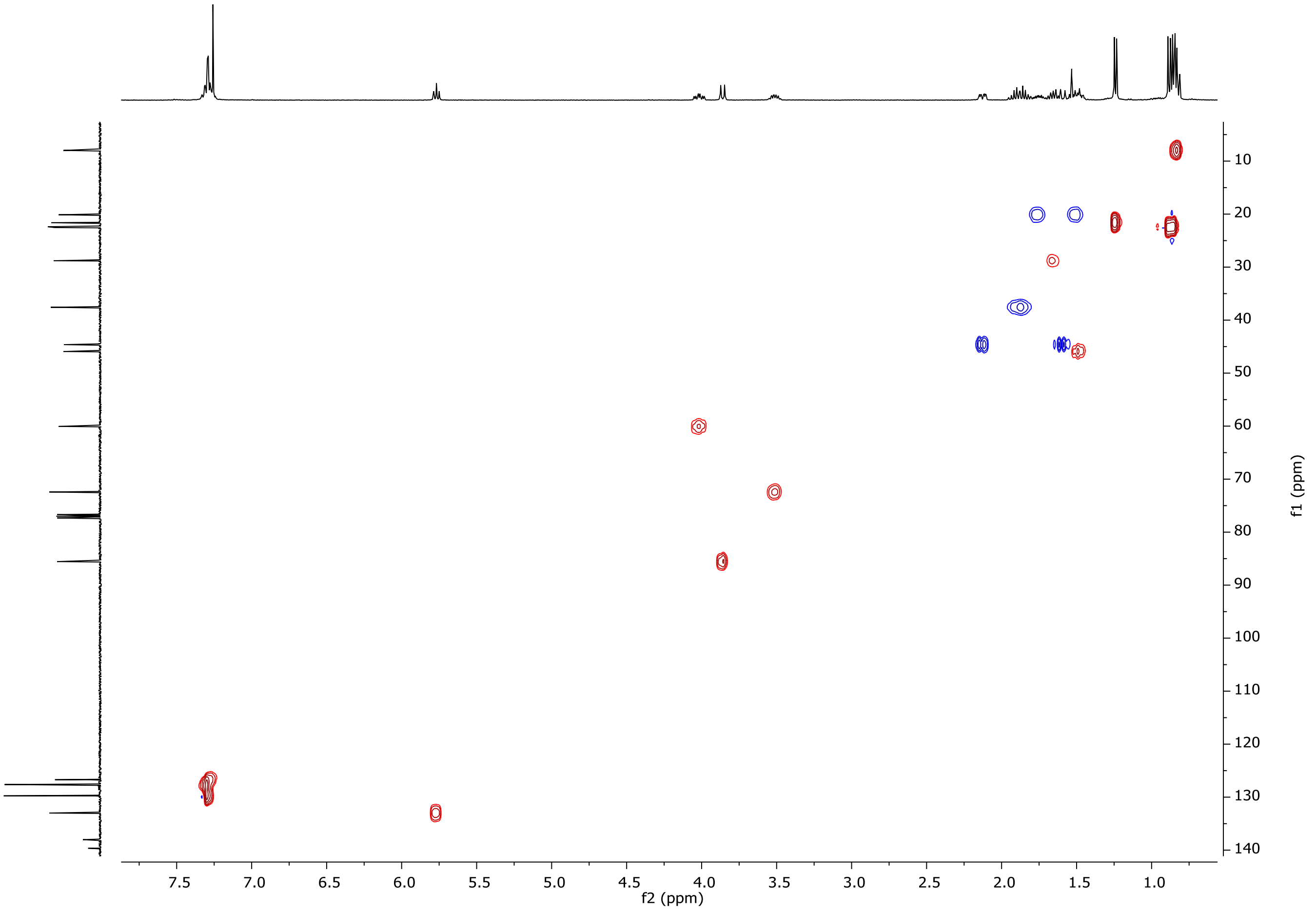

Compound 4h

^{1}H NMR (500 MHz, $CDCl_3$)

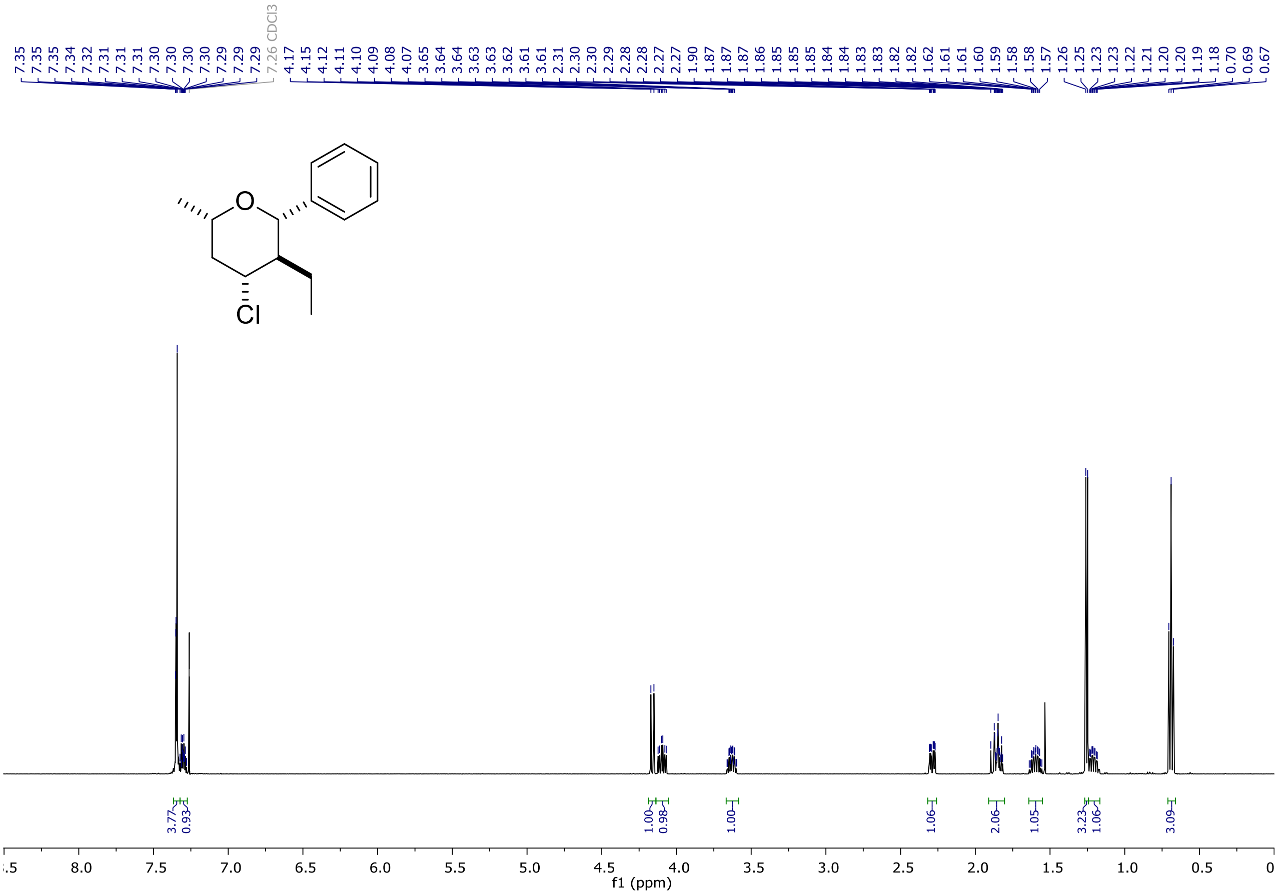

^{13}C NMR (101 MHz, $CDCl_3$)

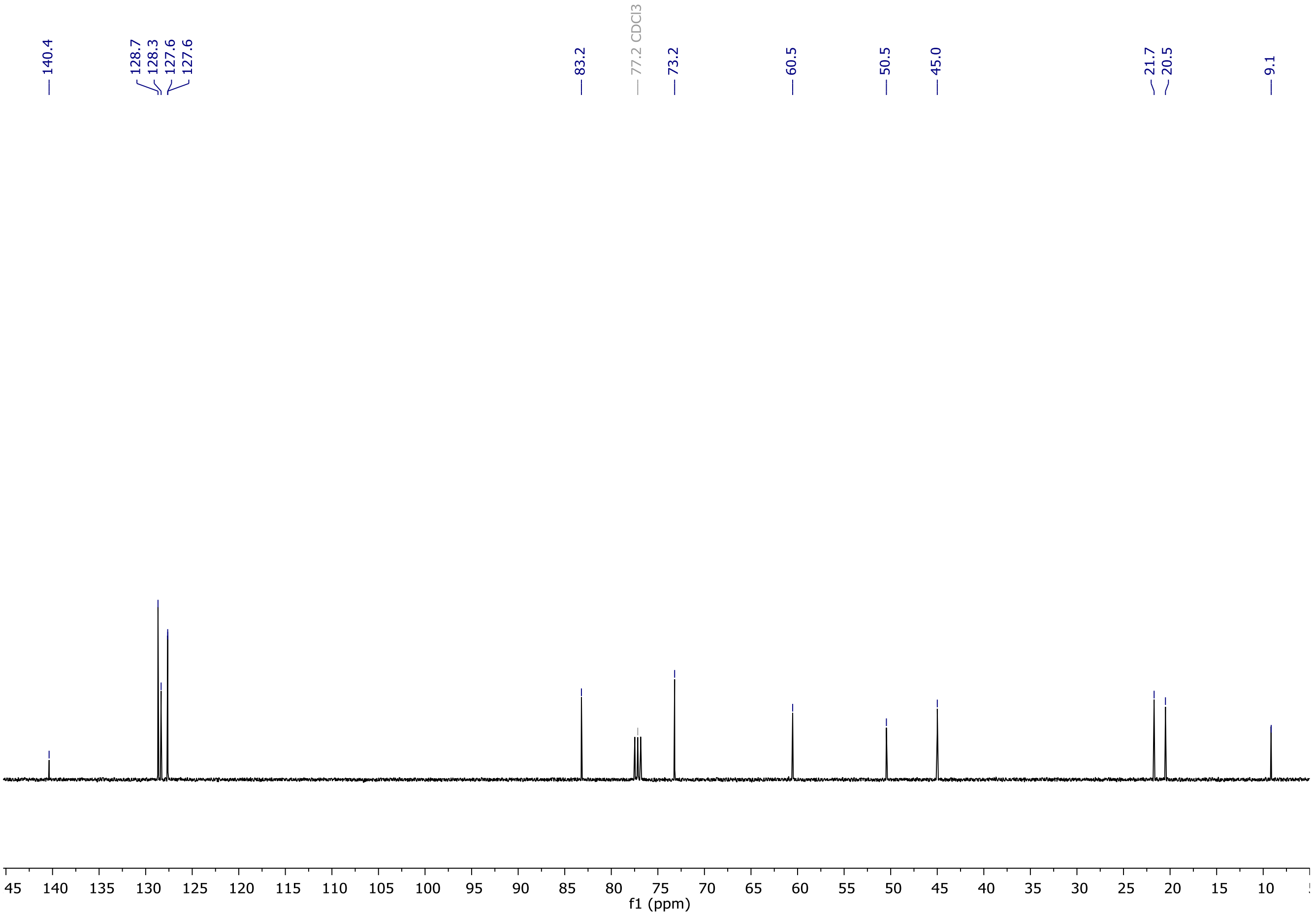

2D-COSY

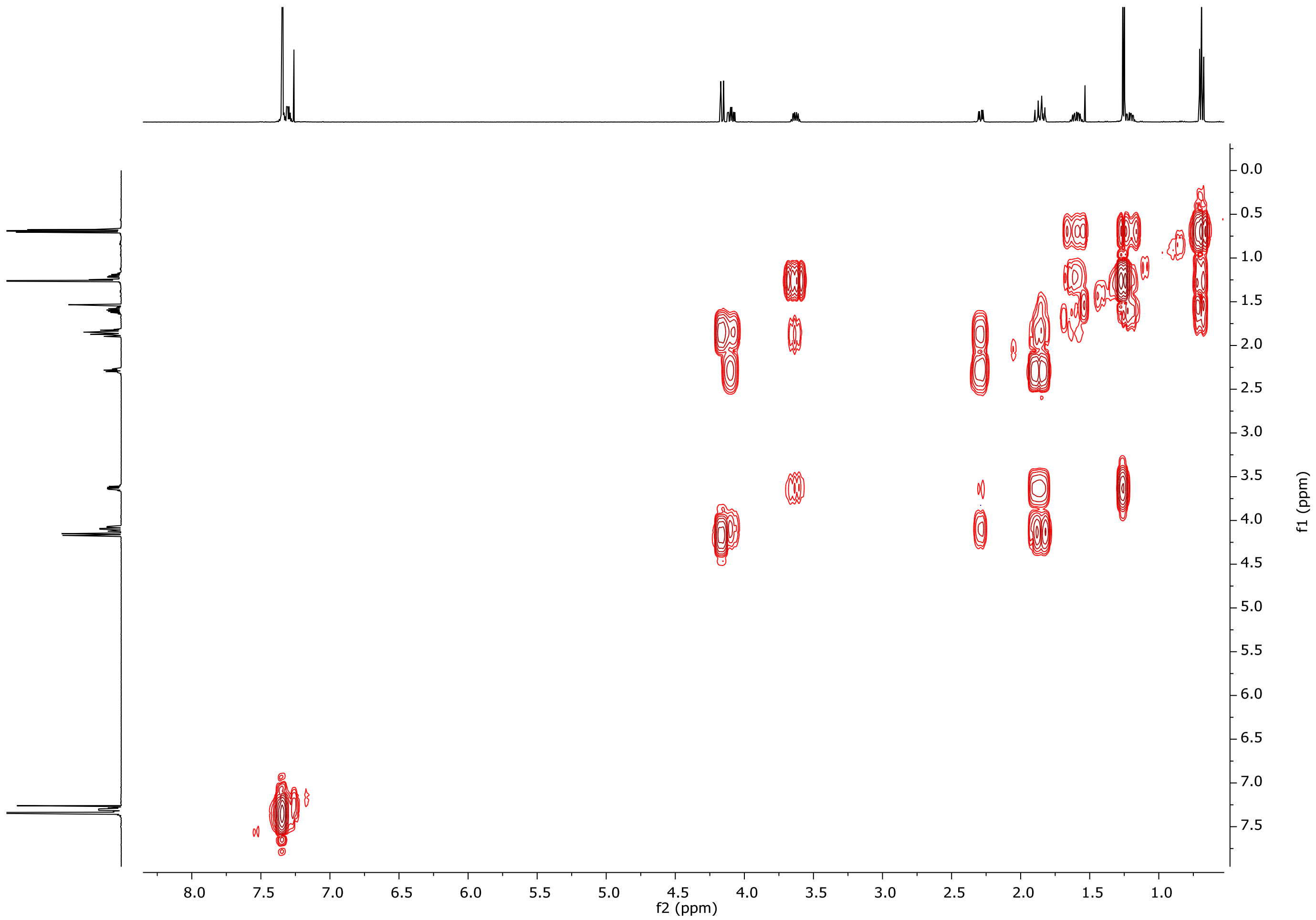

2D-HSQC

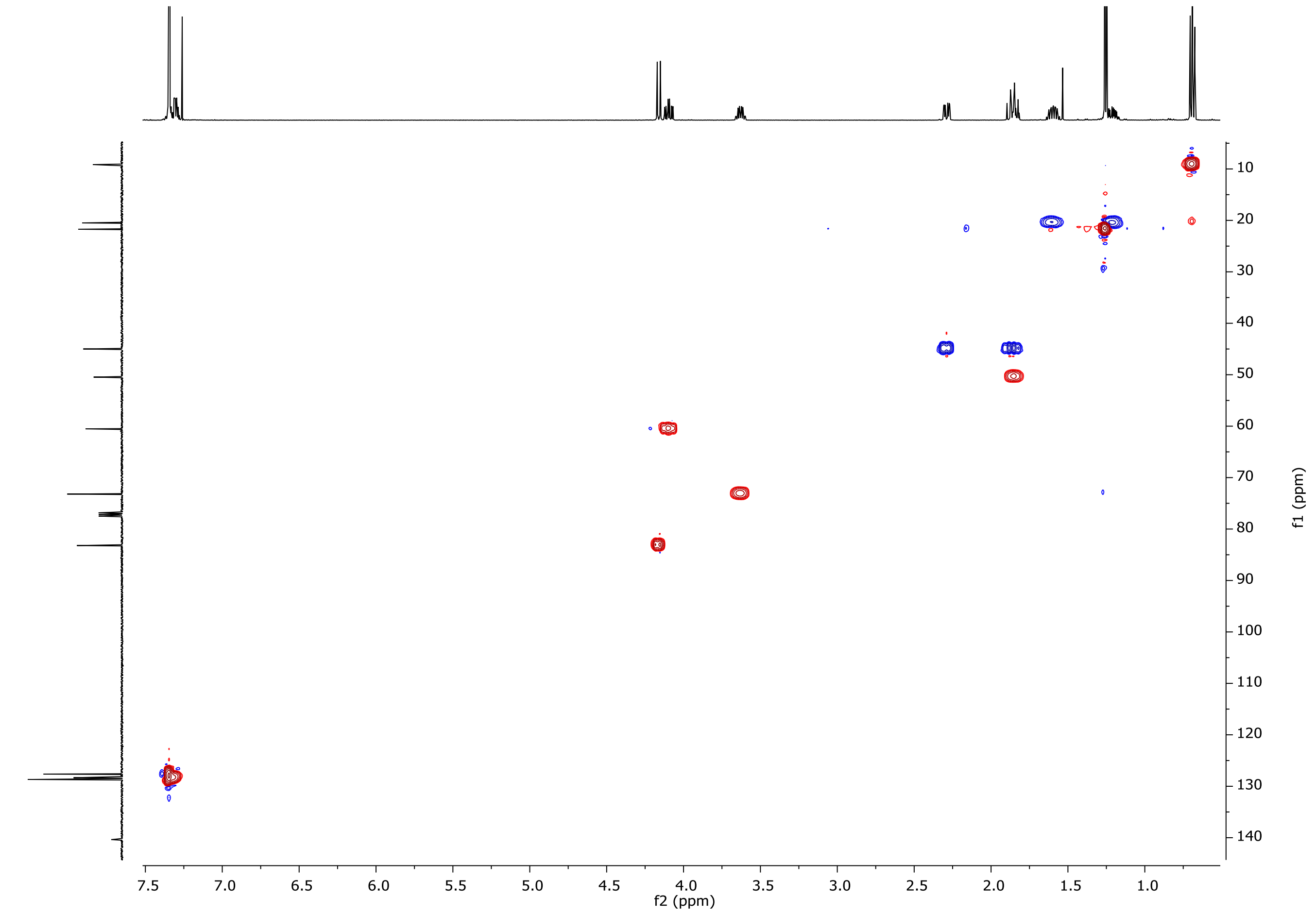

Compound 4i

^{1}H NMR (500 MHz, $CDCl_3$)

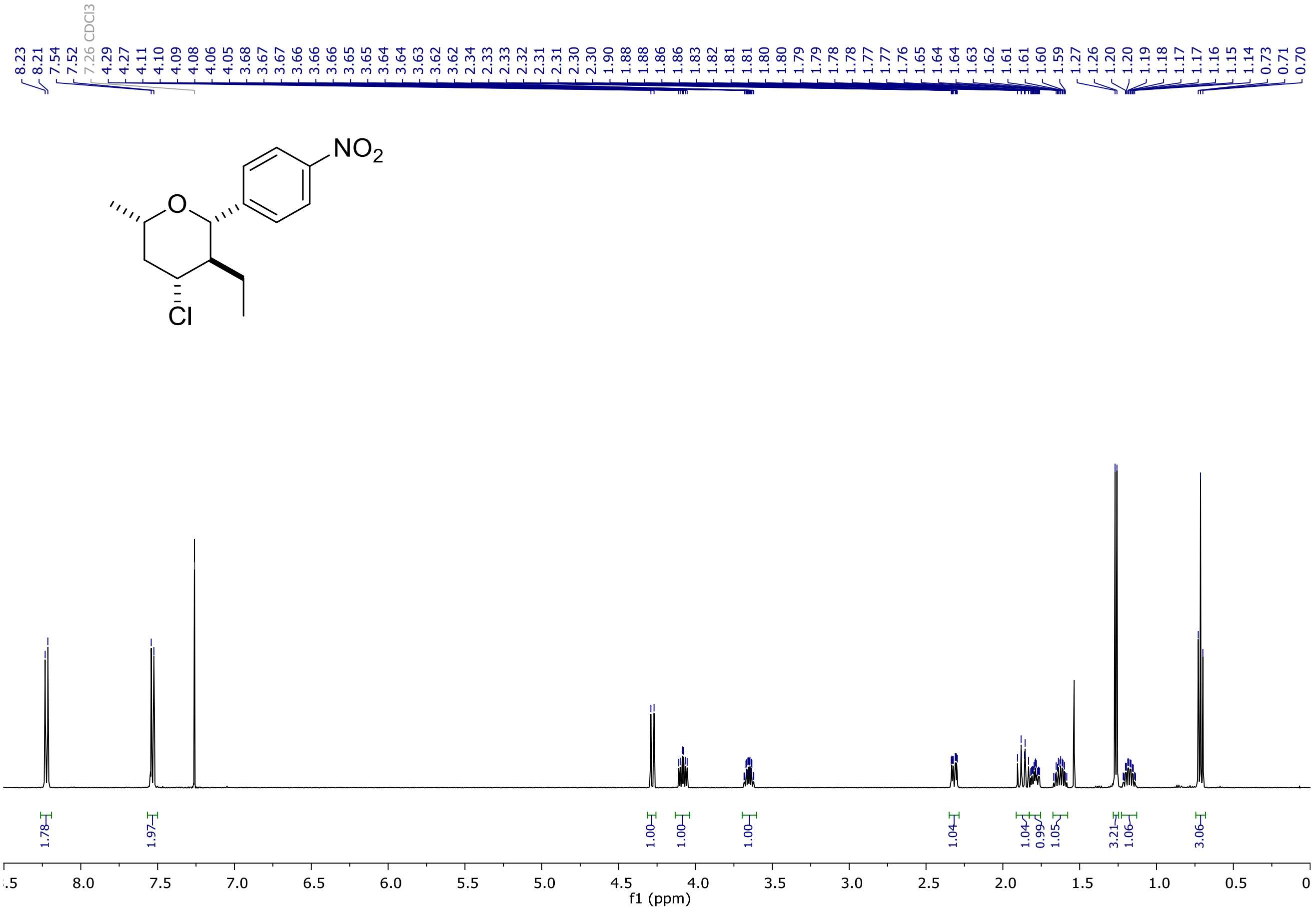

^{13}C NMR (101 MHz, $CDCl_3$)

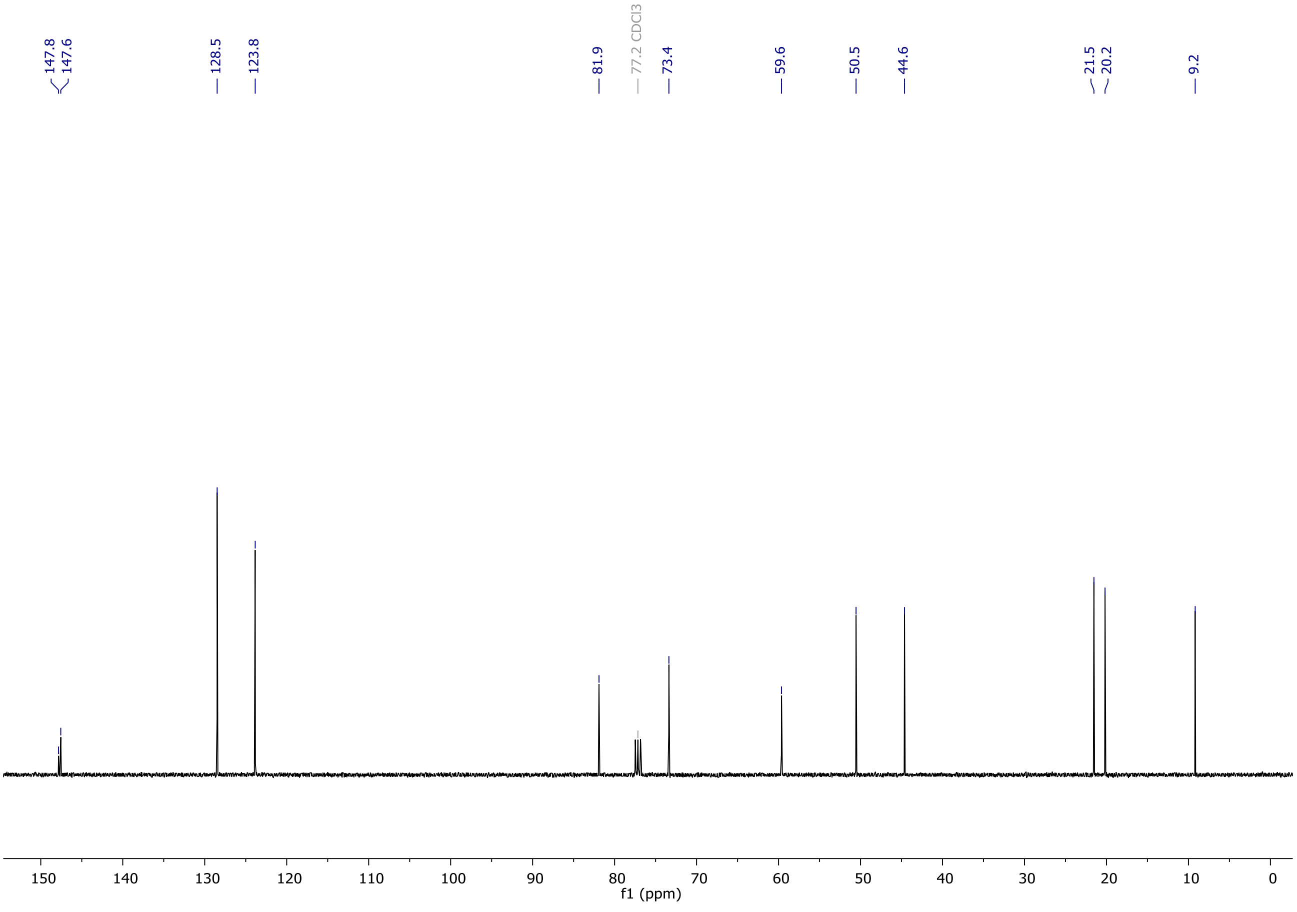

2D-COSY

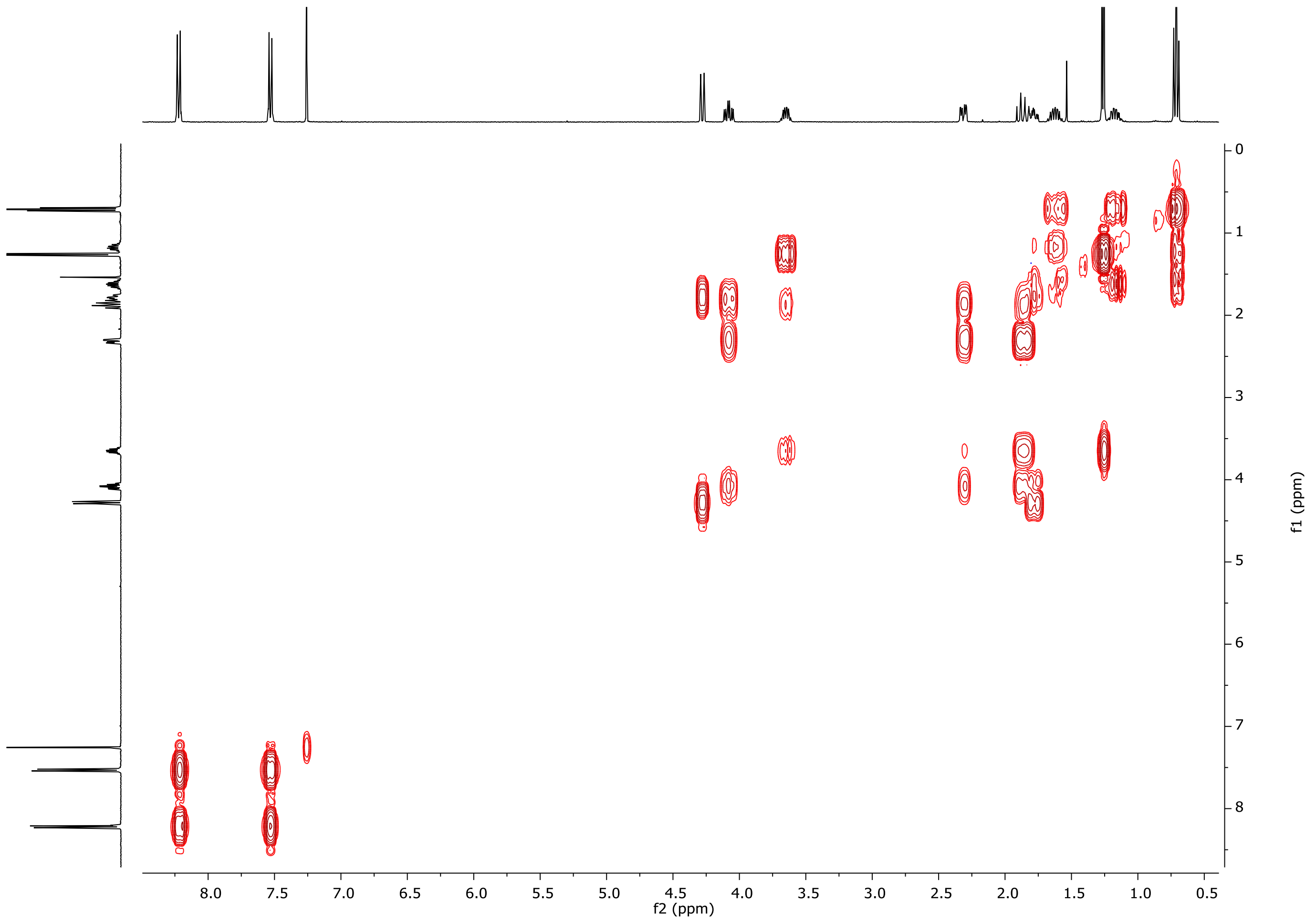

2D-HSQC

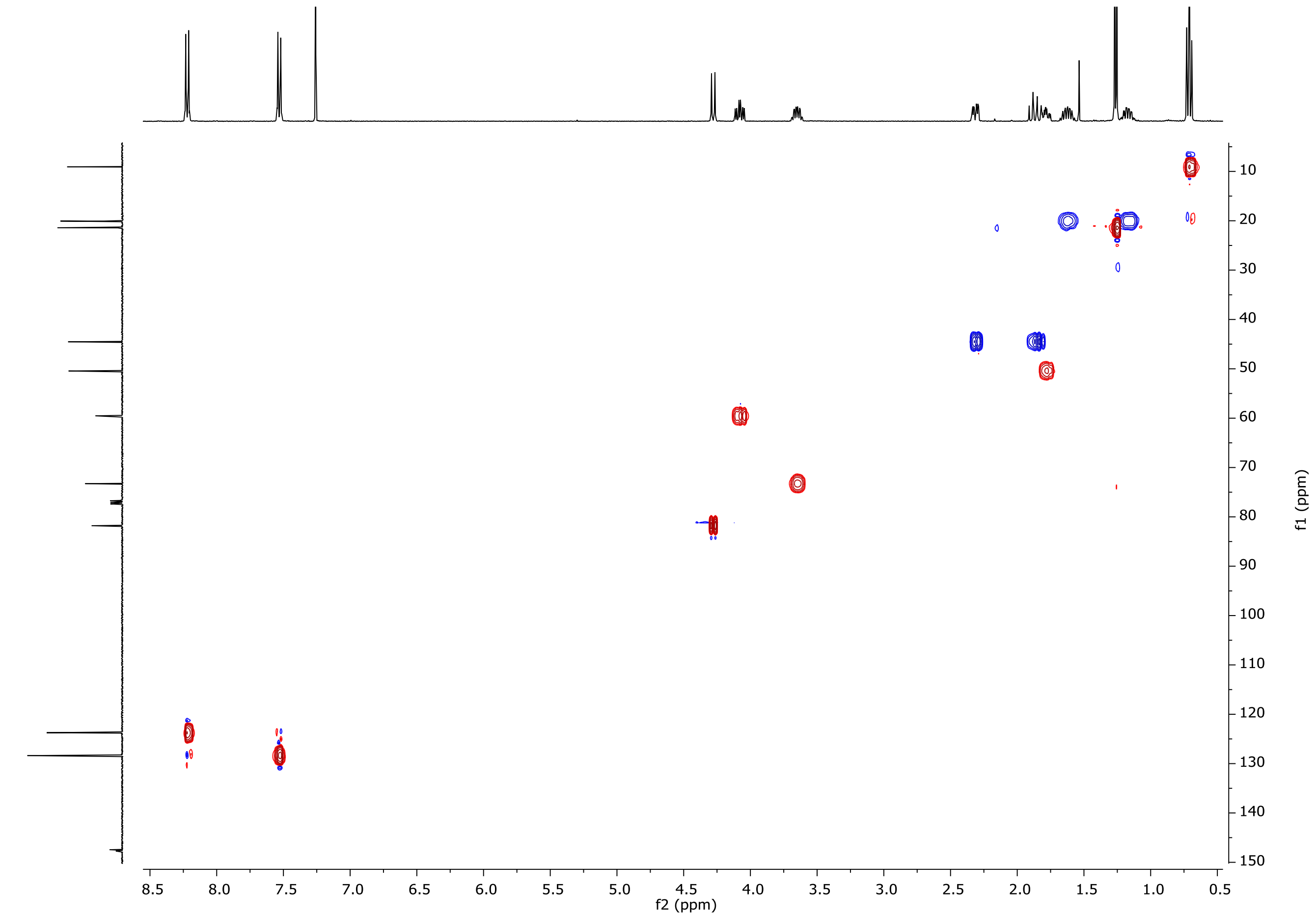

Compound 4j

^{1}H NMR (500 MHz, $CDCl_3$)

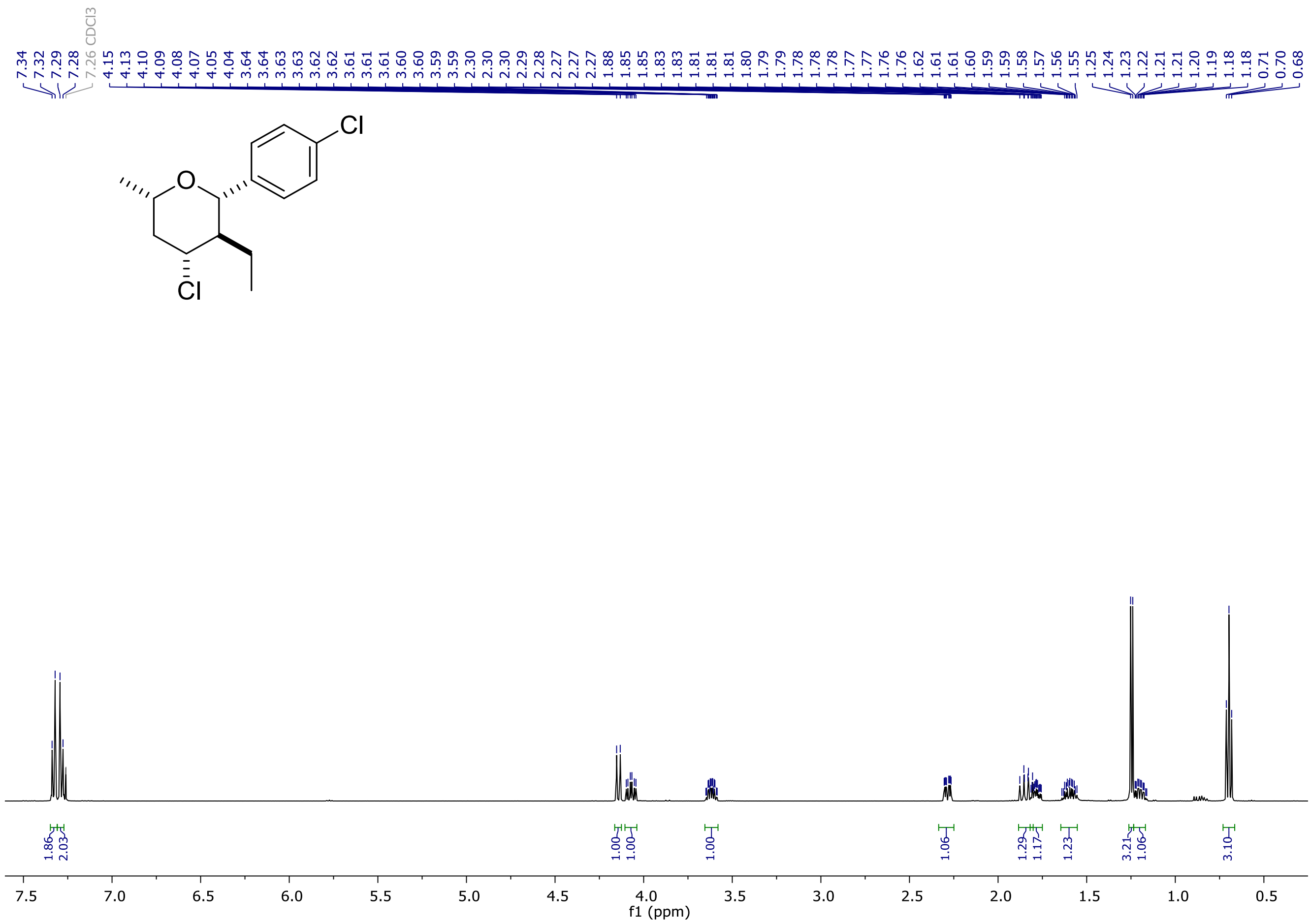

^{13}C NMR (101 MHz, $CDCl_3$)

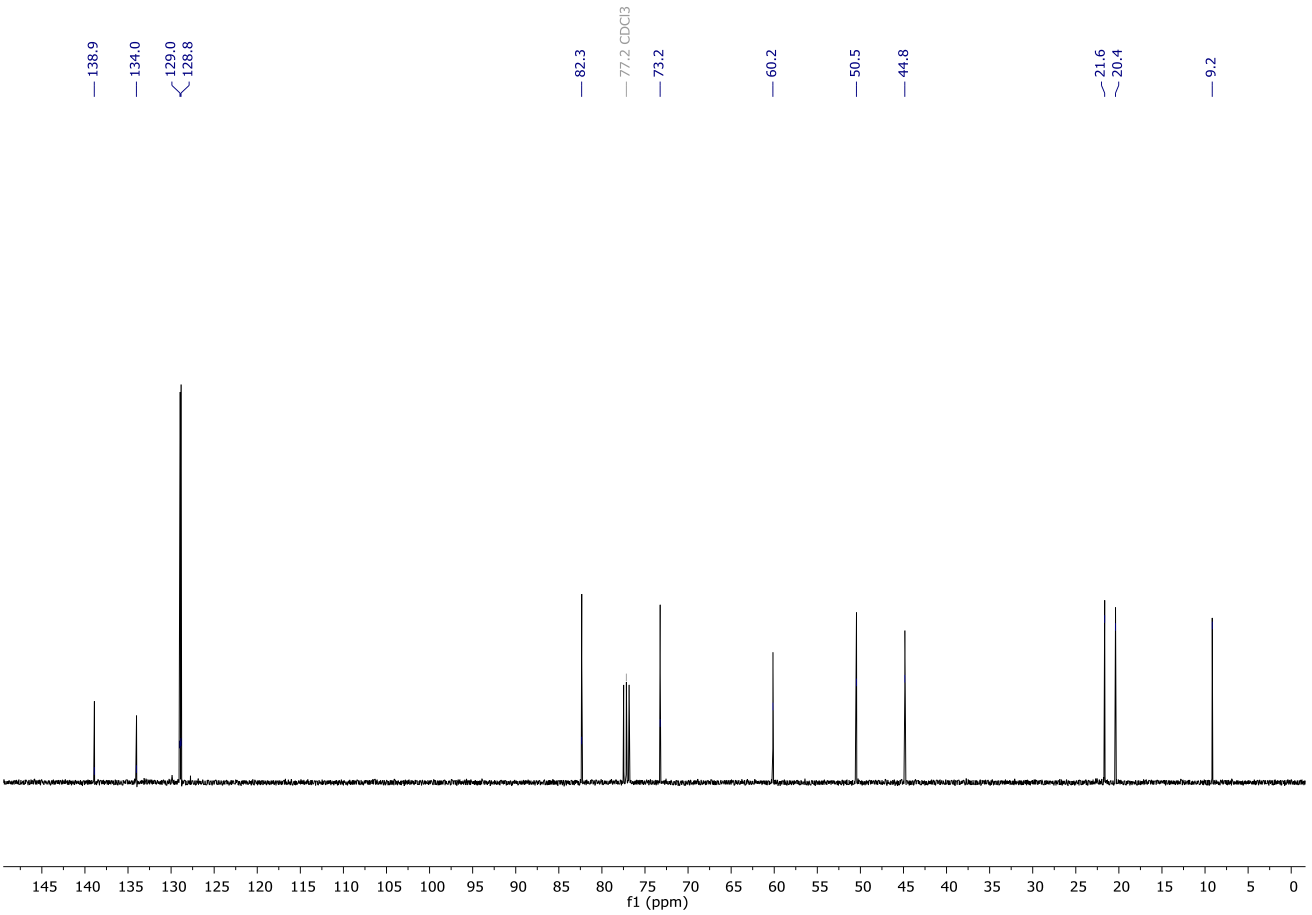

2D-COSY

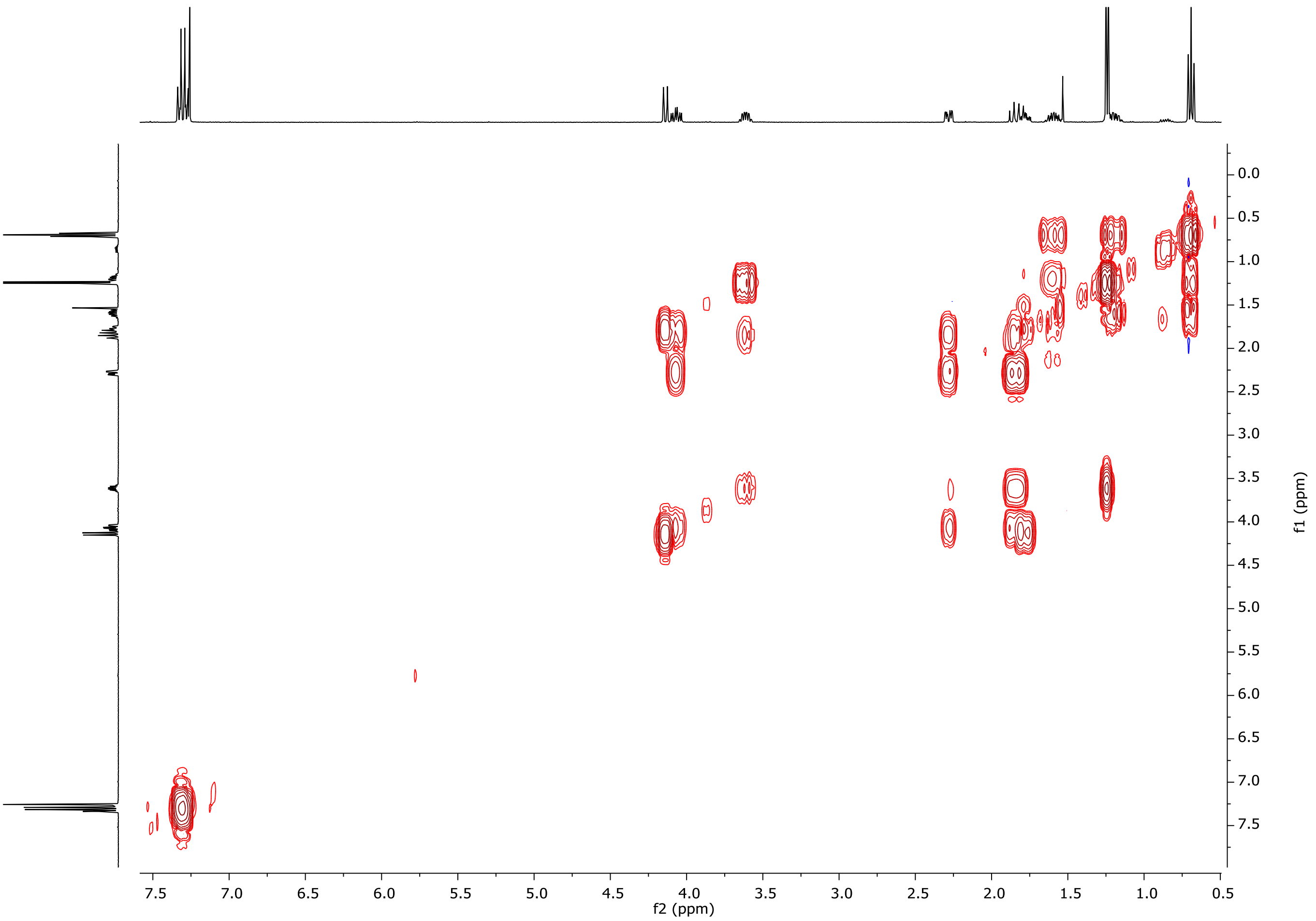

2D-HSQC

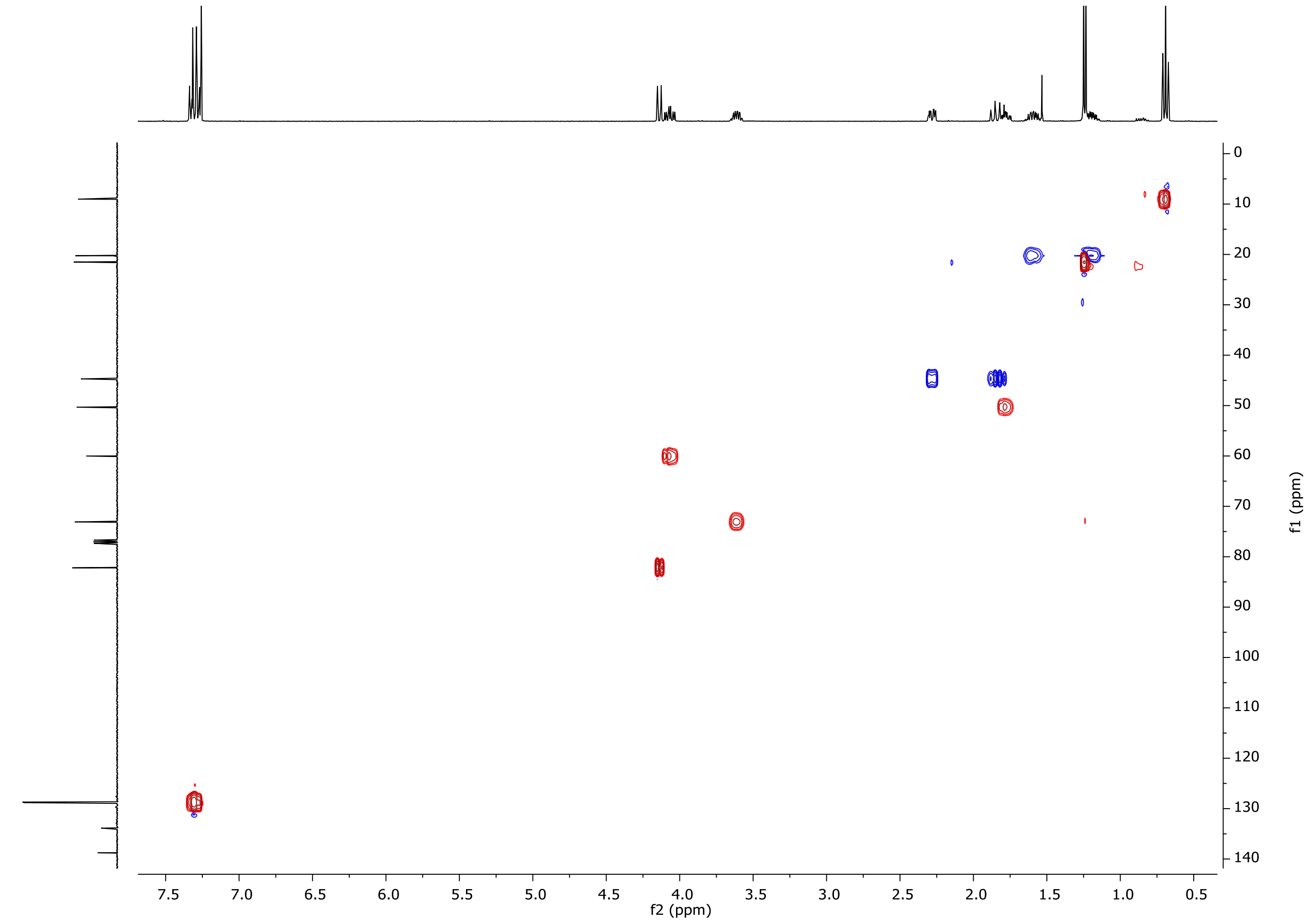

Compound 4k

[1]**H NMR** (500 MHz, $CDCl_3$)

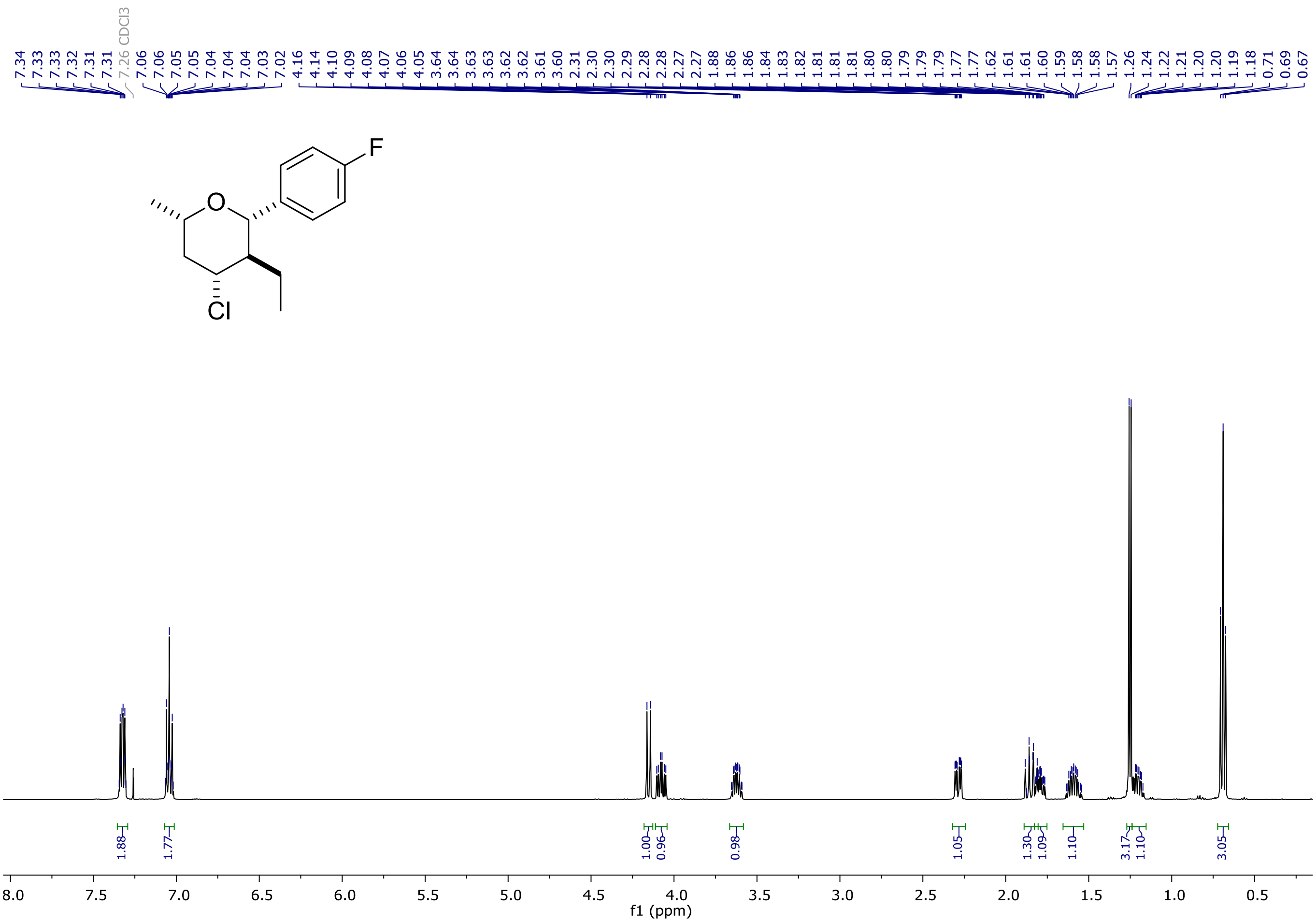

[13]**C NMR** (101 MHz, $CDCl_3$)

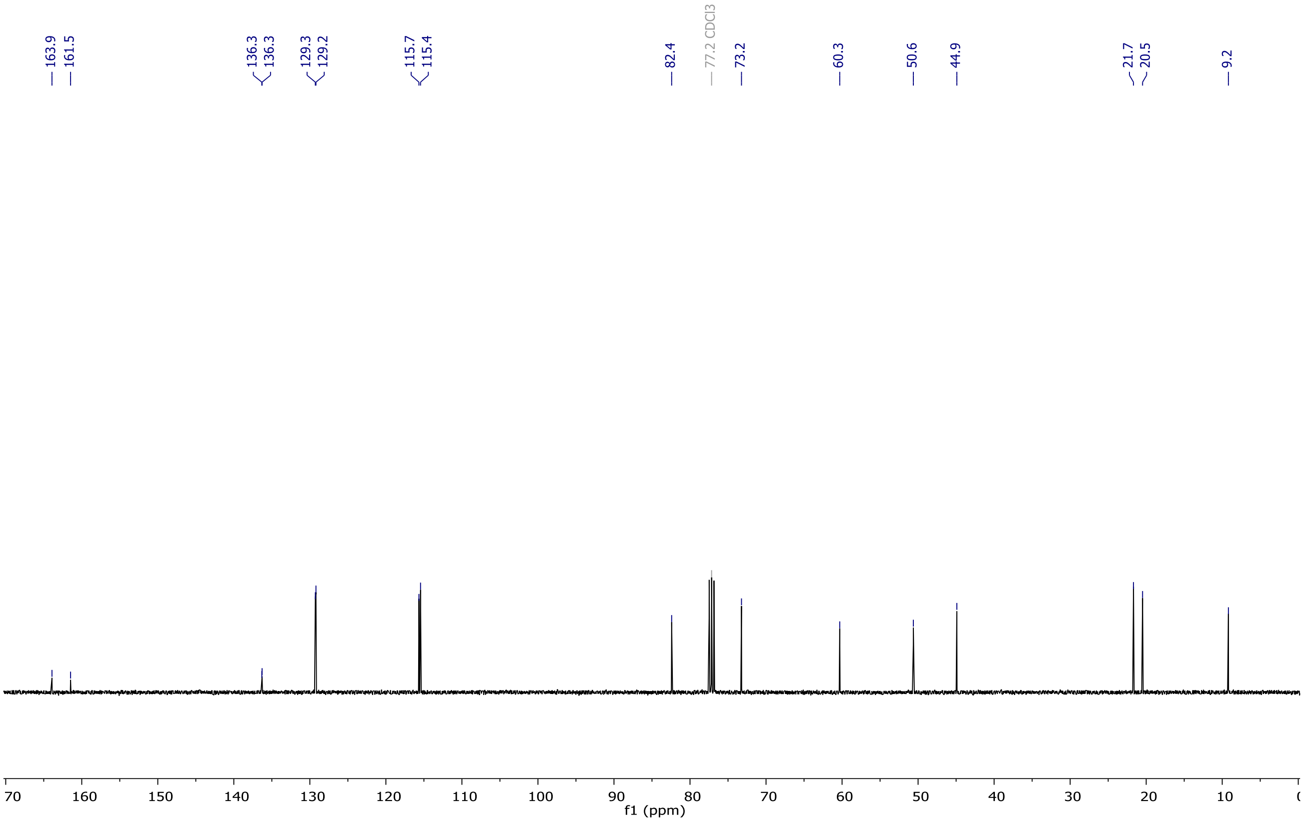

2D-COSY

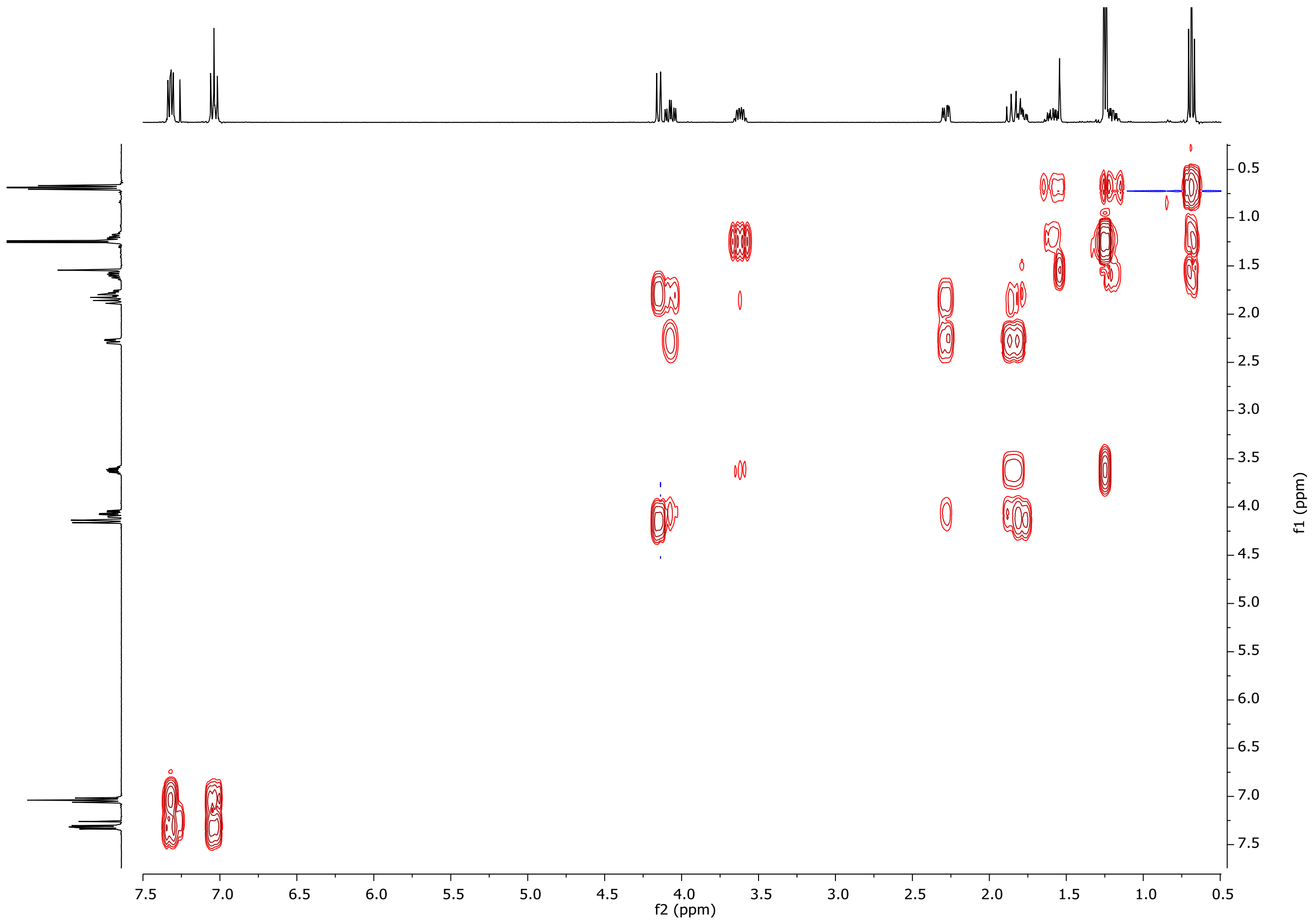

2D-HSQC

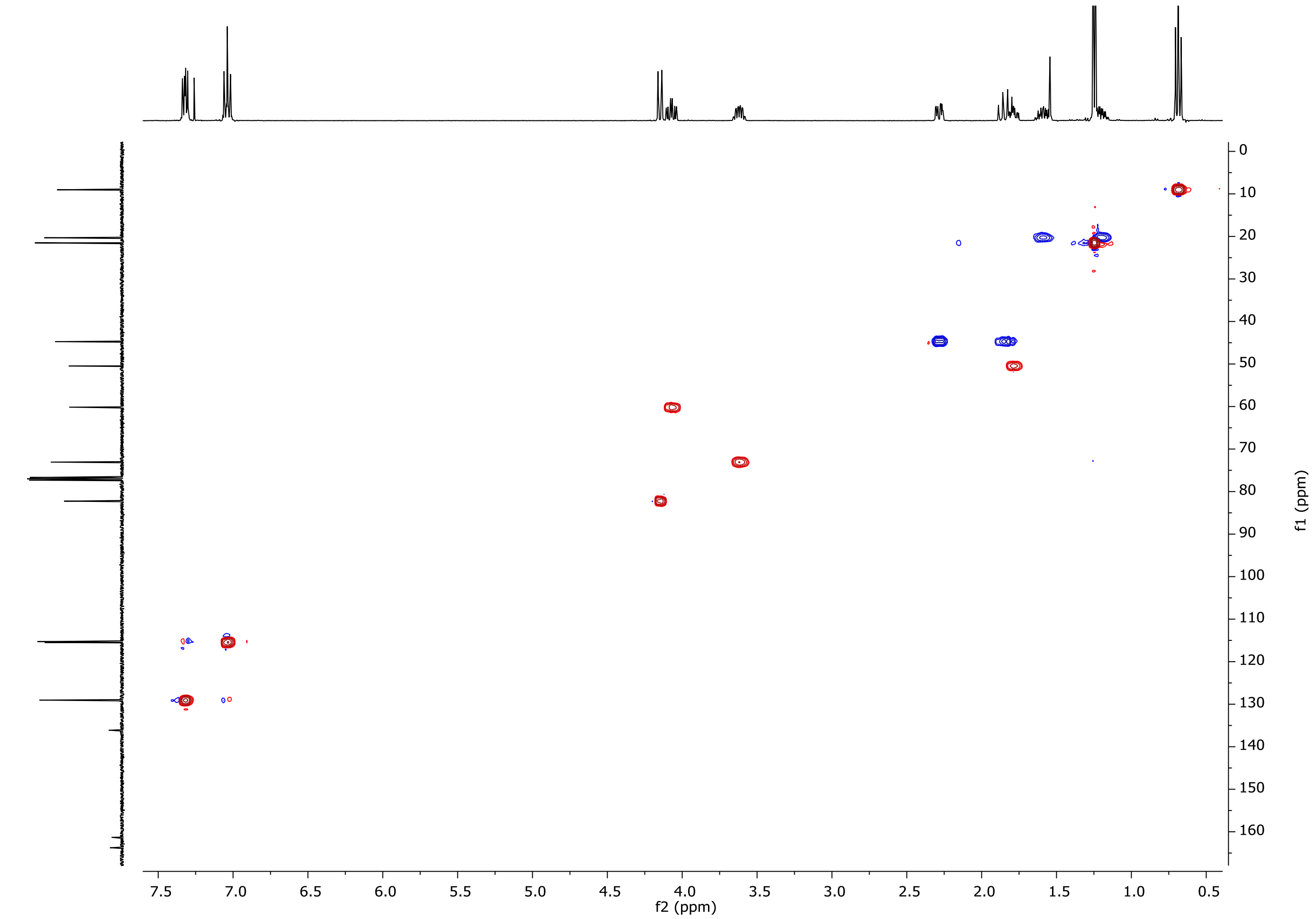

Compound 4l

^{1}H NMR (500 MHz, $CDCl_3$)

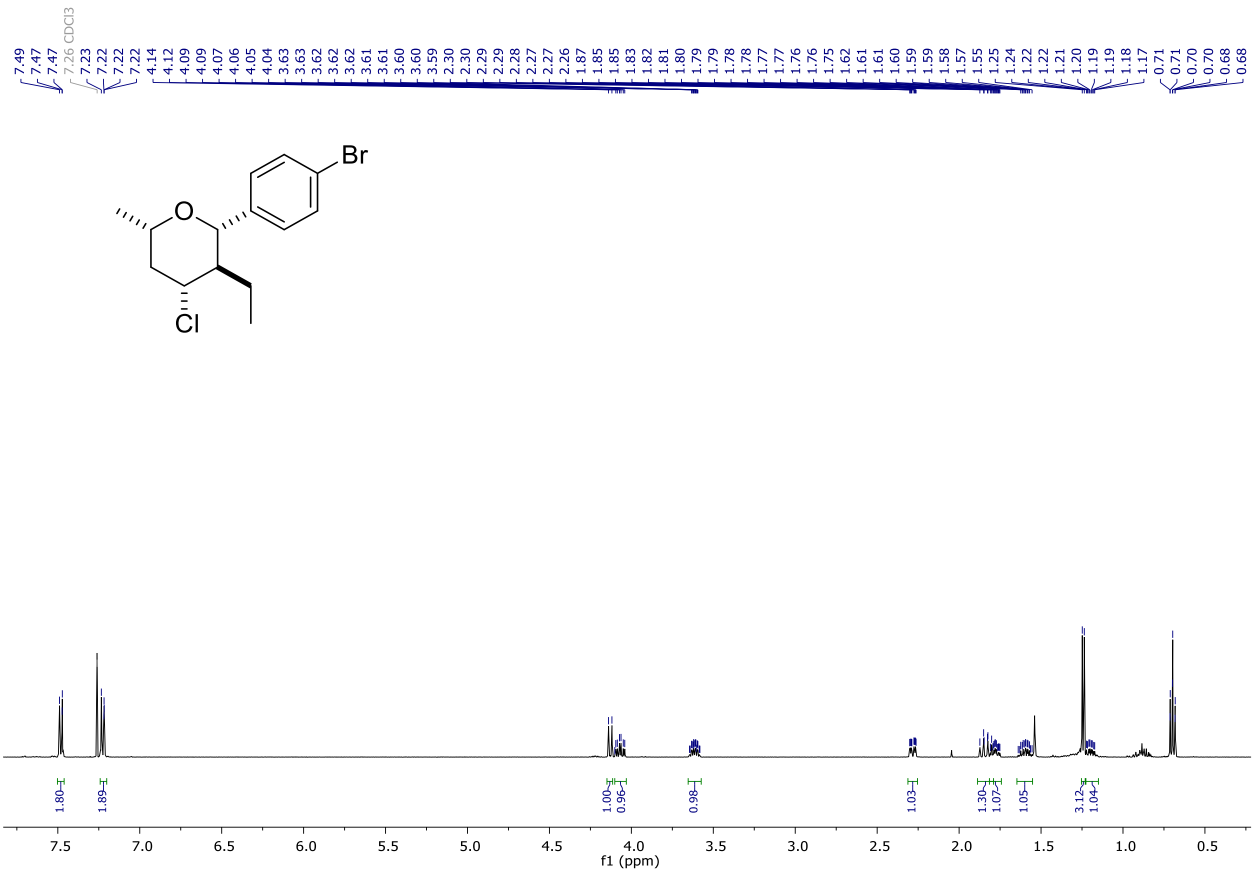

^{13}C NMR (101 MHz, $CDCl_3$)

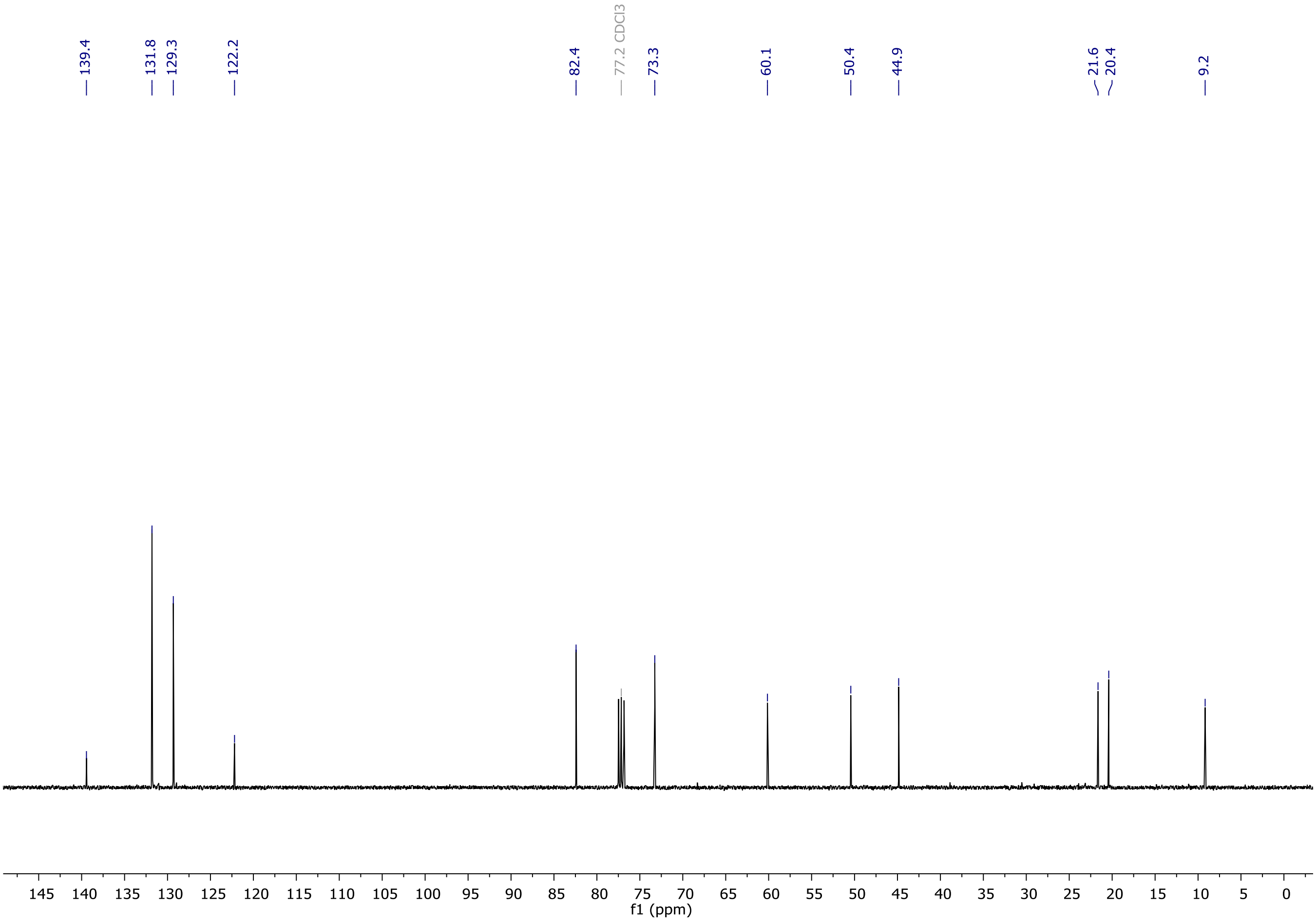

2D-COSY

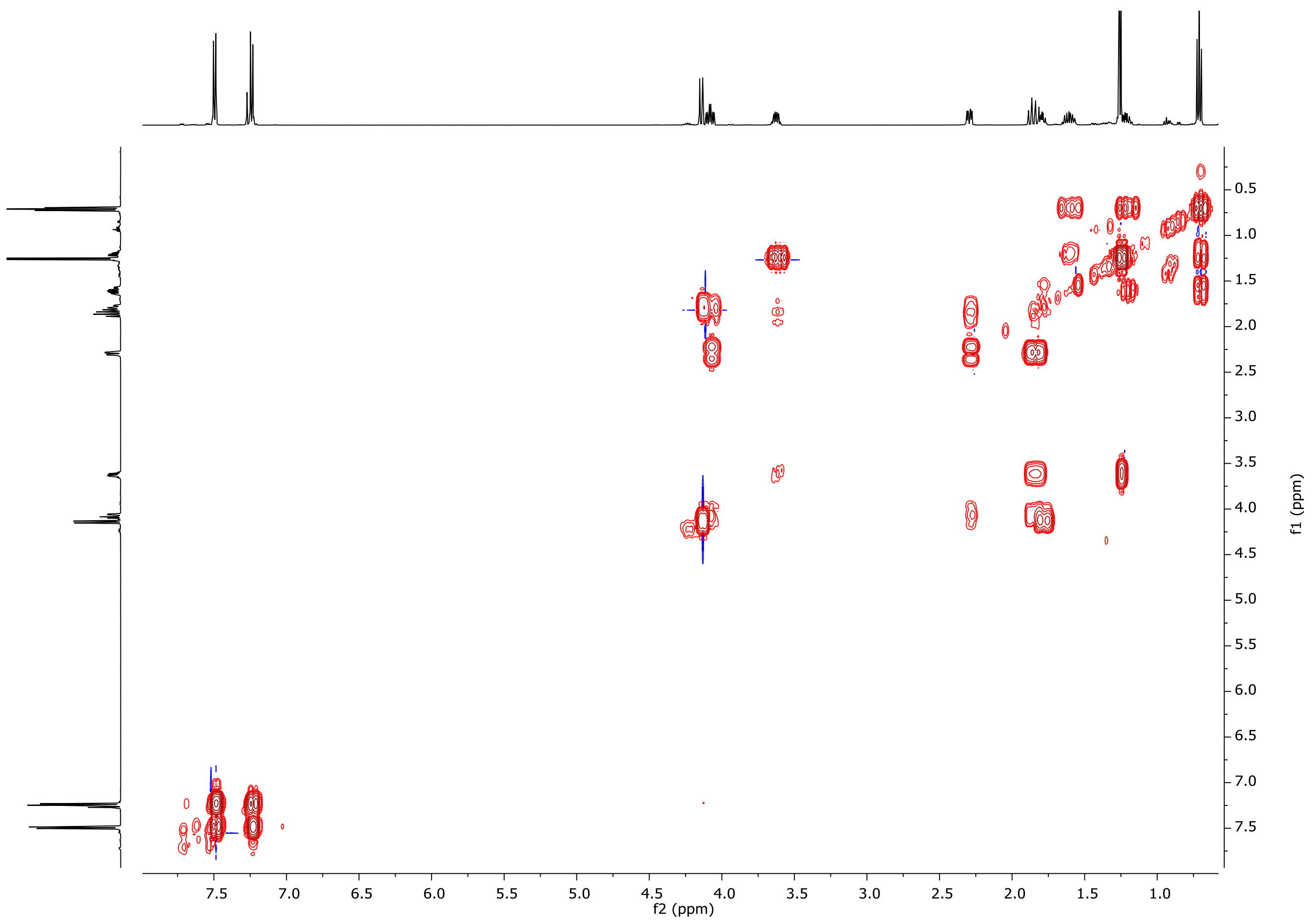

2D-HSQC

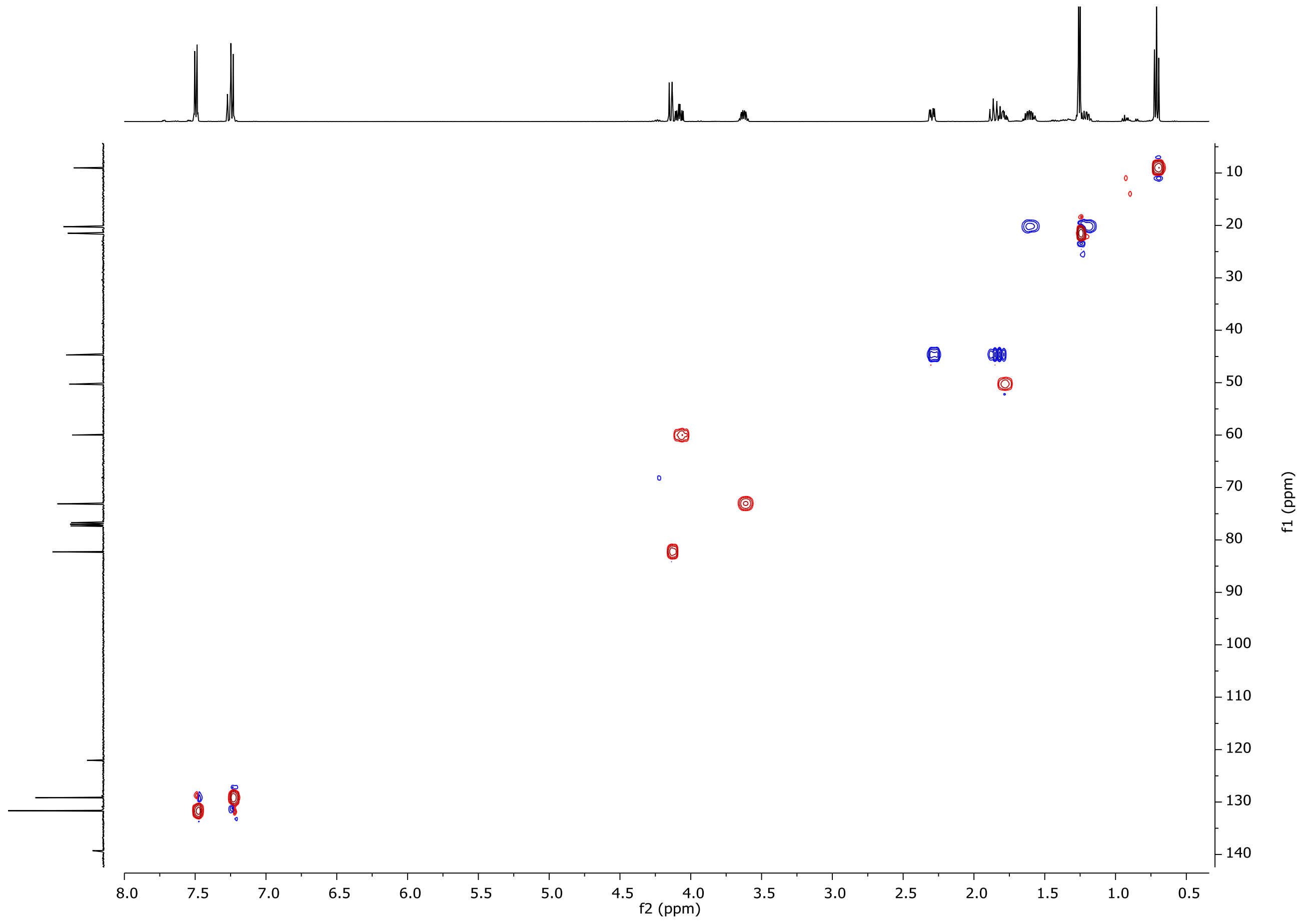

Compound 4m

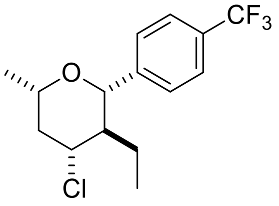

^{1}H NMR (500 MHz, $CDCl_3$)

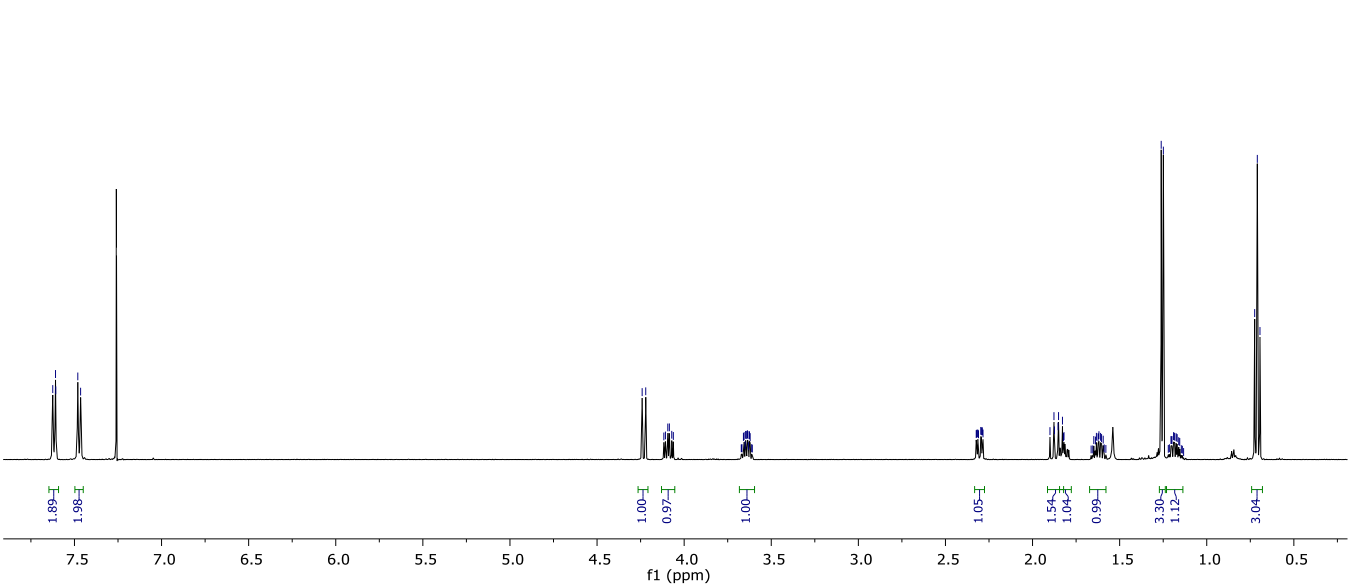

^{13}C NMR (101 MHz, $CDCl_3$)

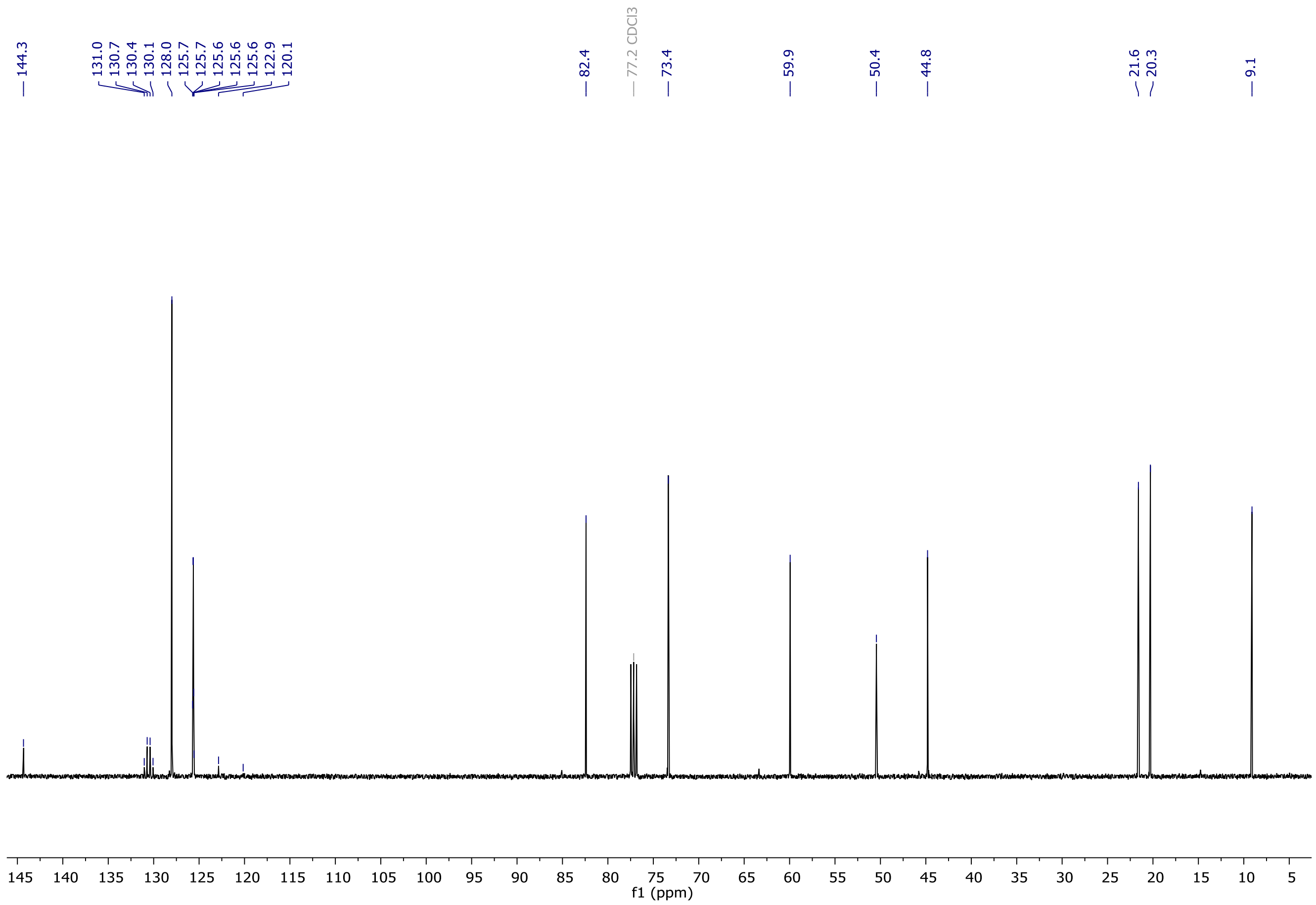

2D-COSY

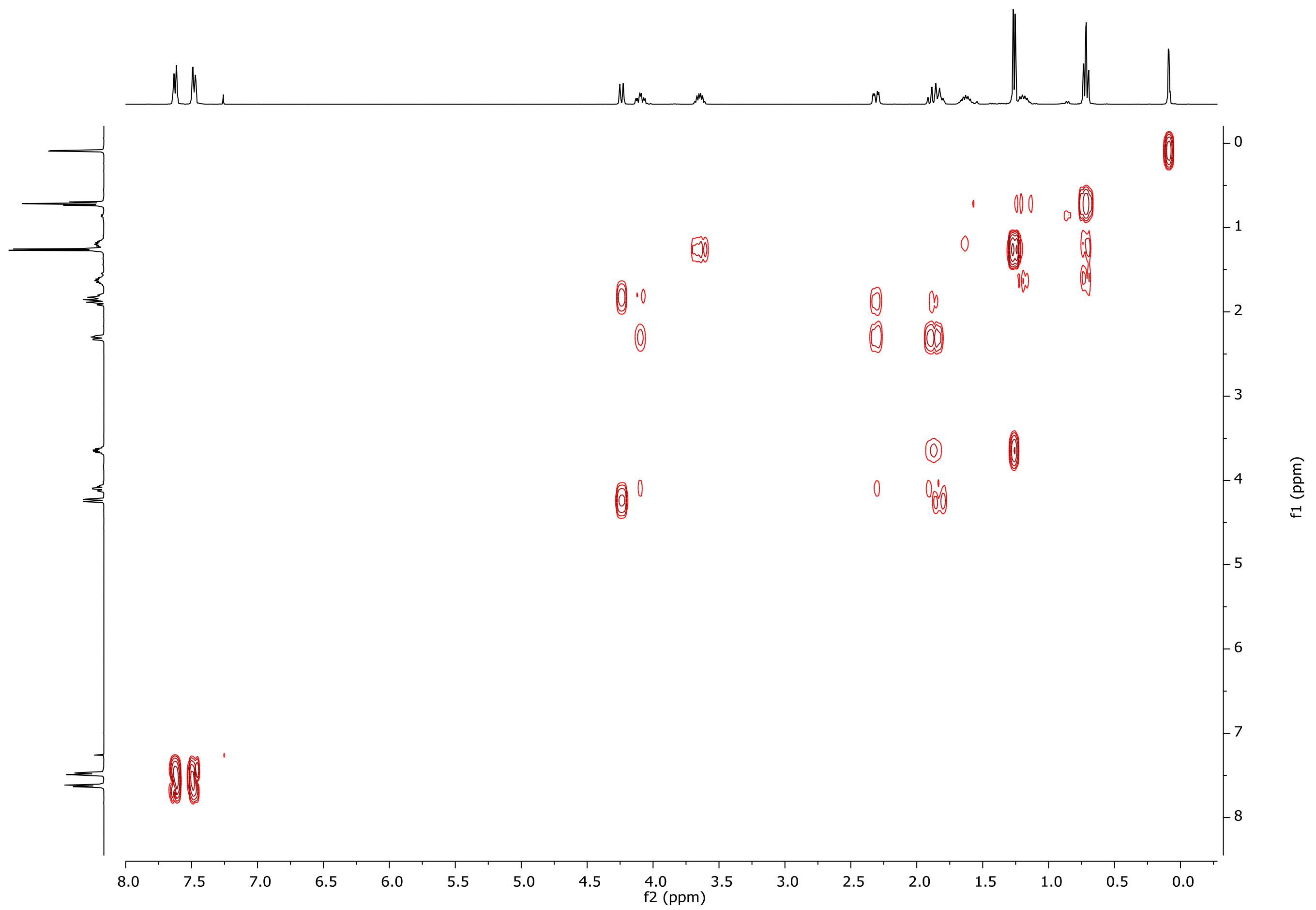

2D-HSQC

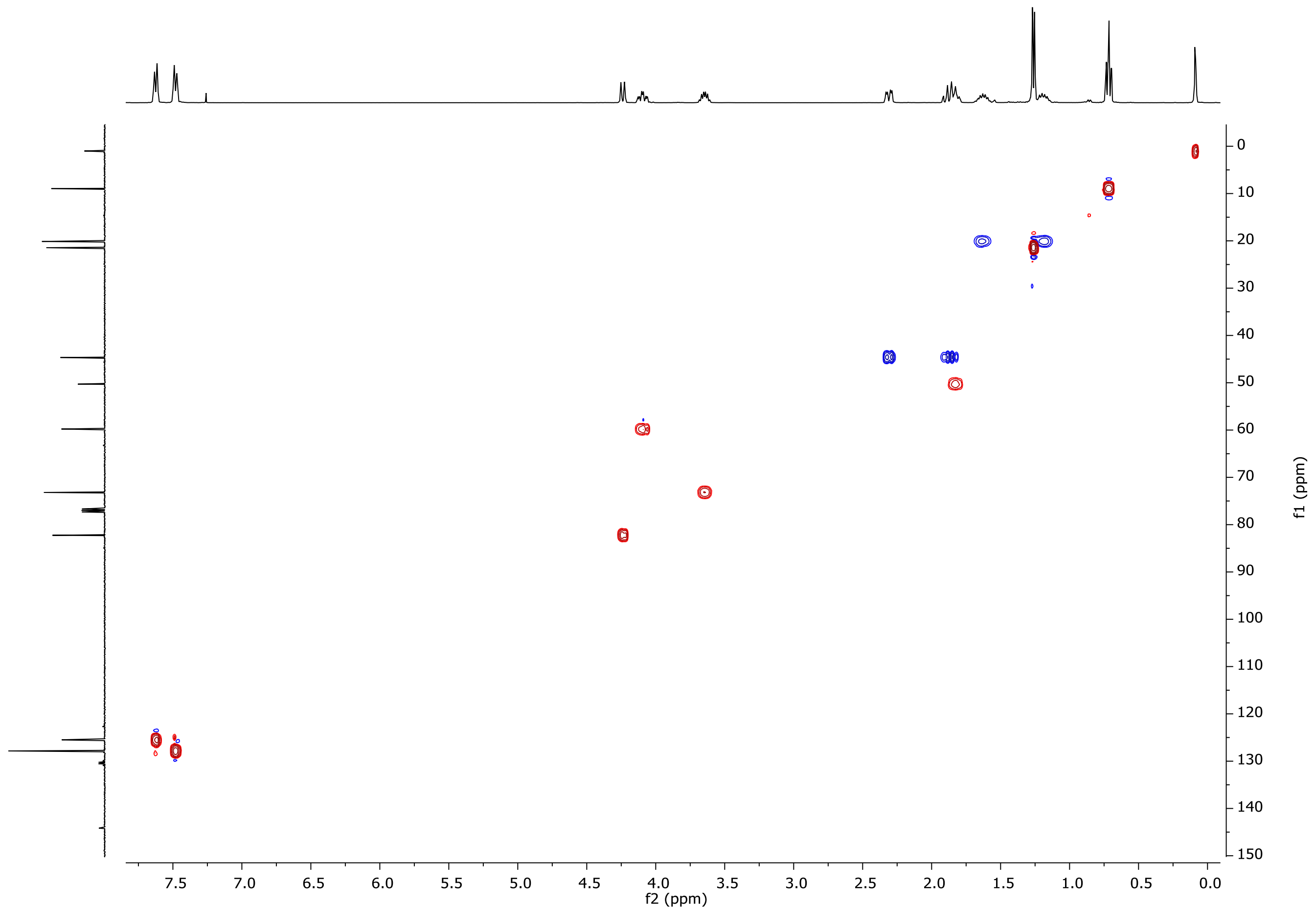

Compound 4n

^{1}H NMR (500 MHz, $CDCl_3$)

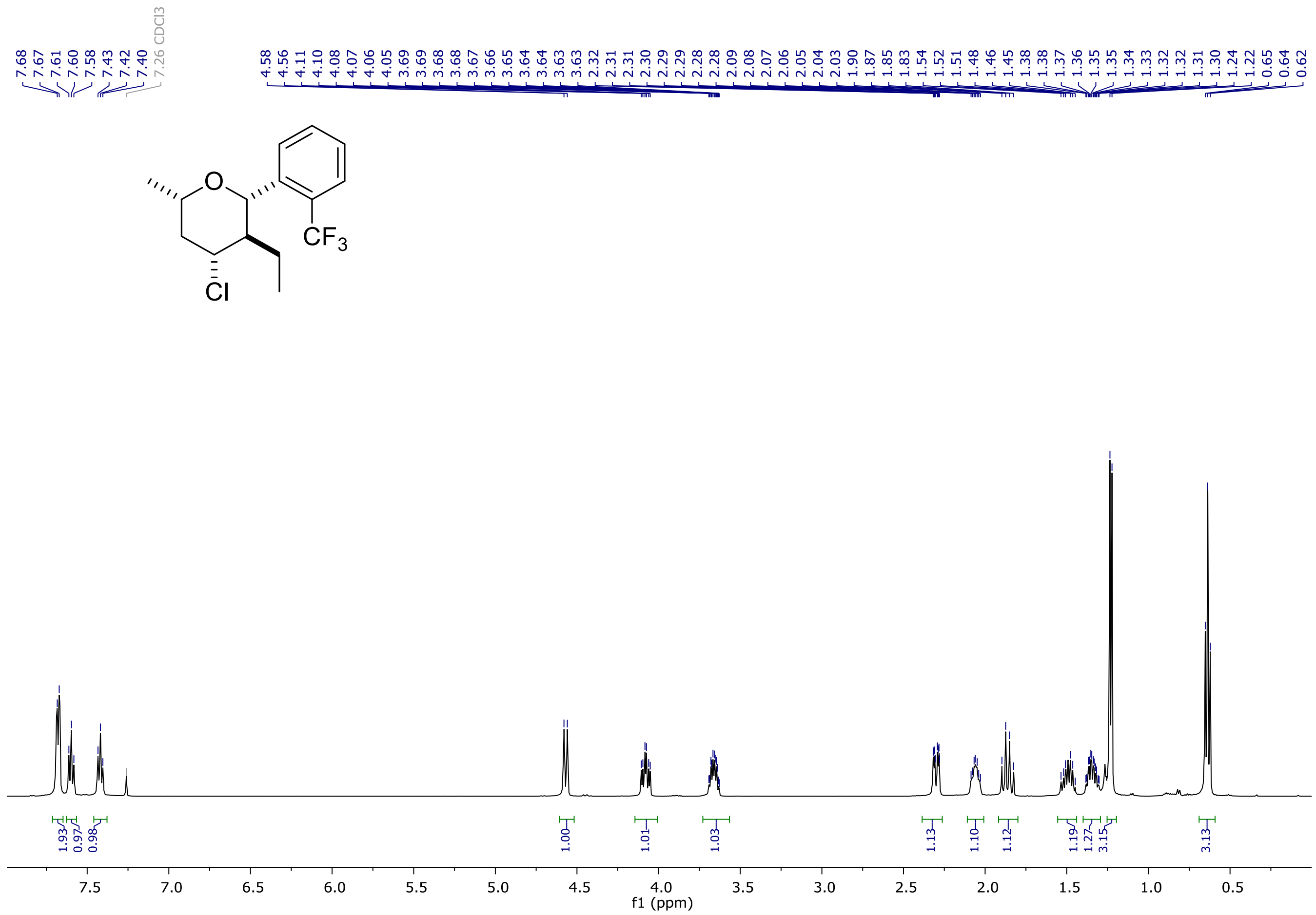

^{13}C NMR (101 MHz, $CDCl_3$)

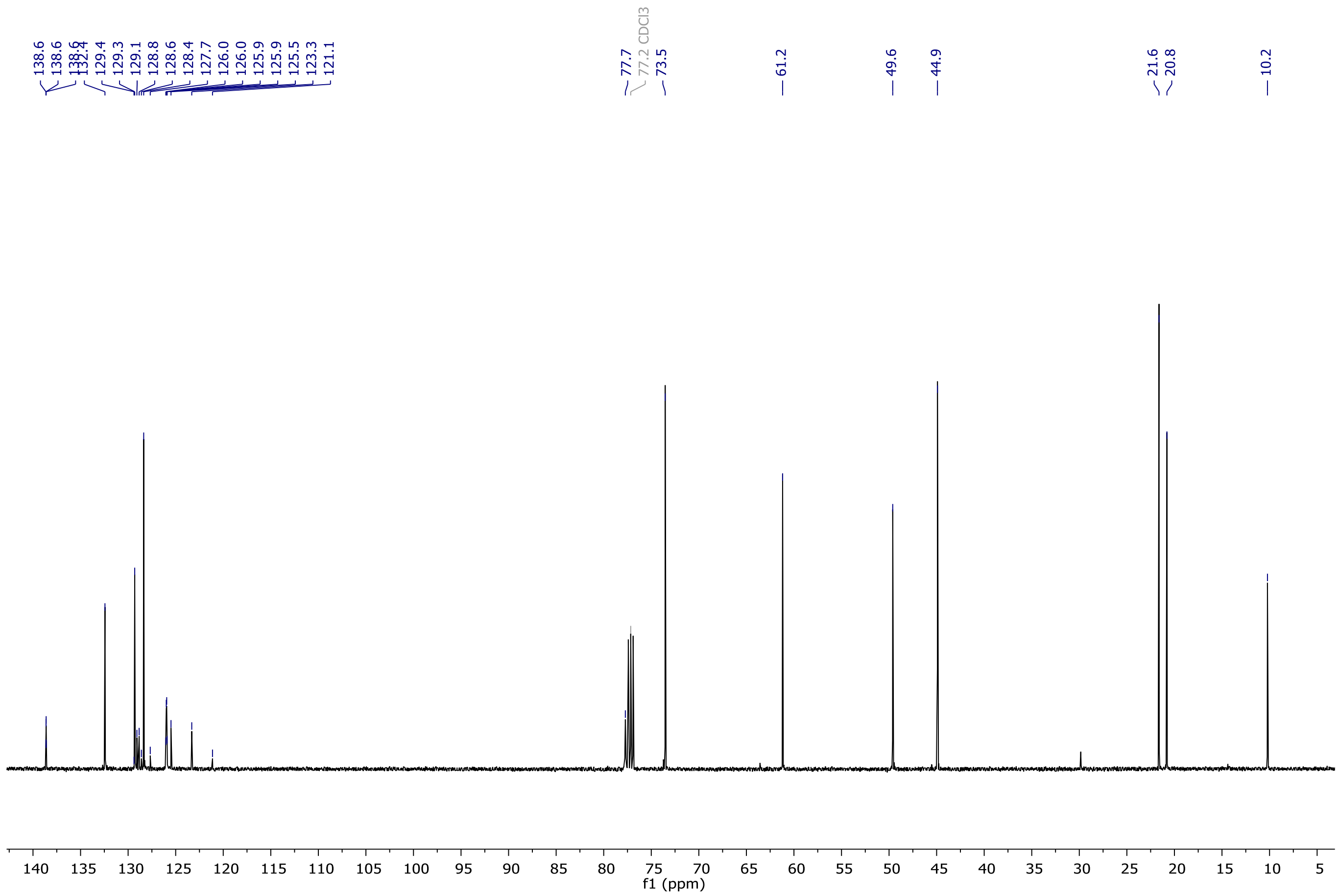

2D-COSY

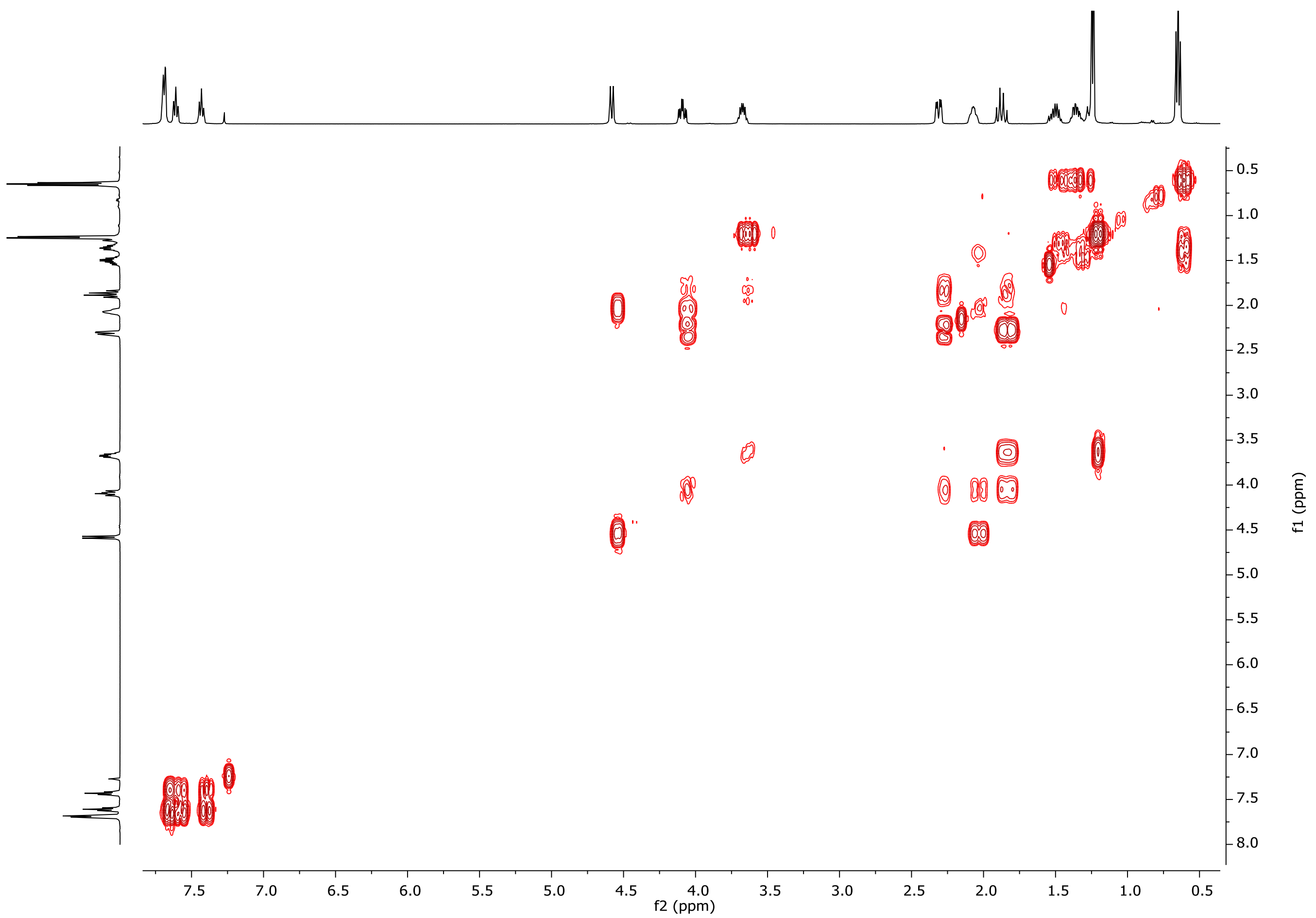

2D-HSQC

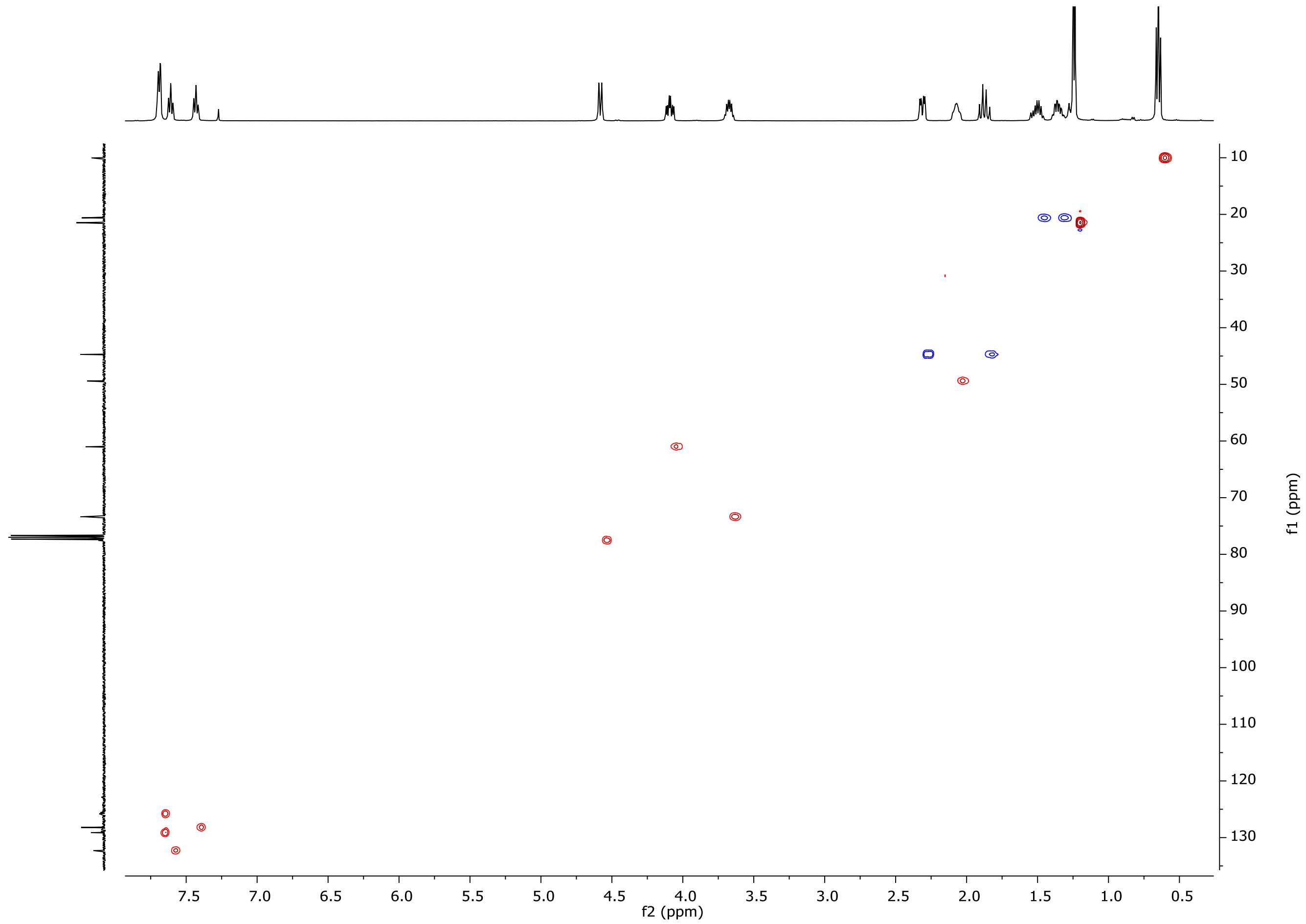

Compound 4o

^{1}H NMR (500 MHz, $CDCl_3$)

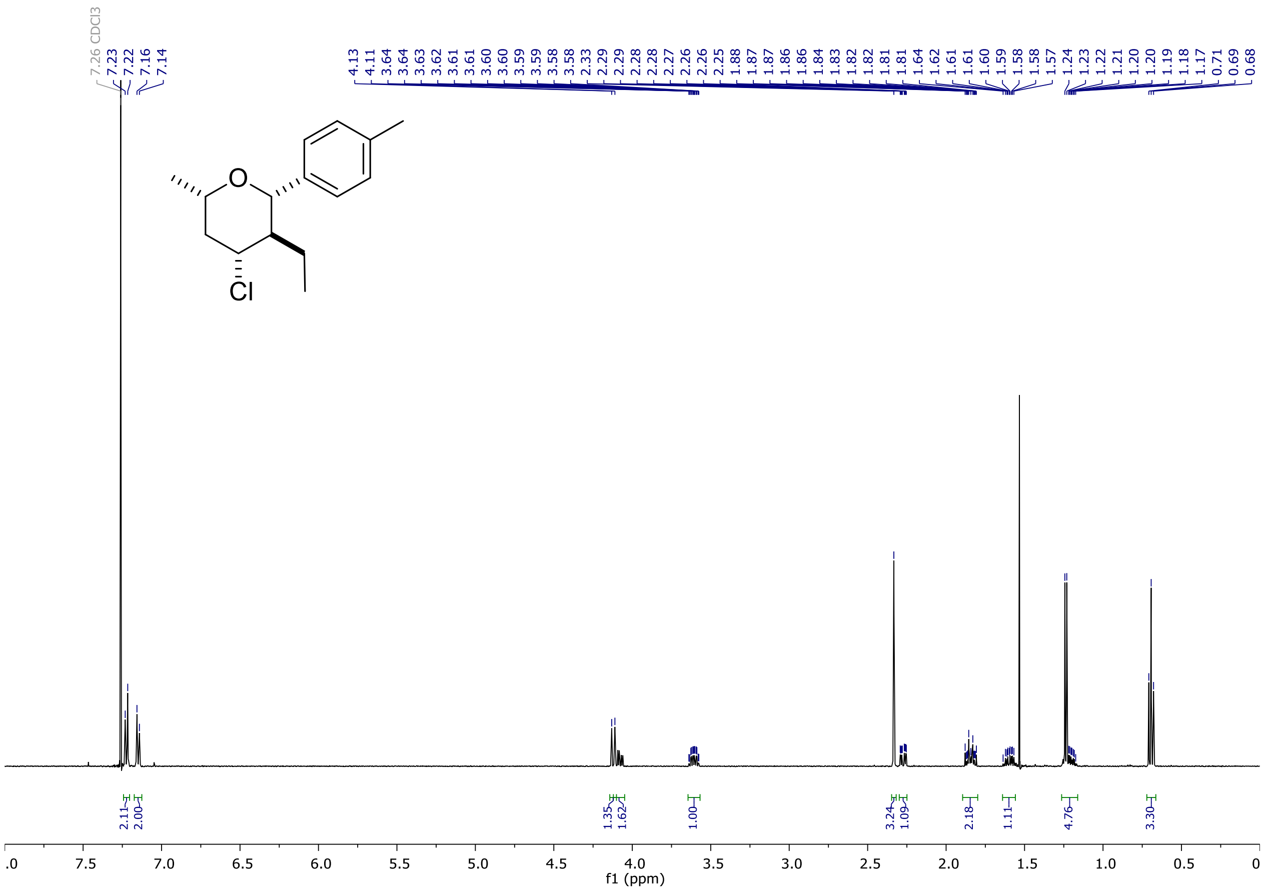

^{13}C NMR (101 MHz, $CDCl_3$)

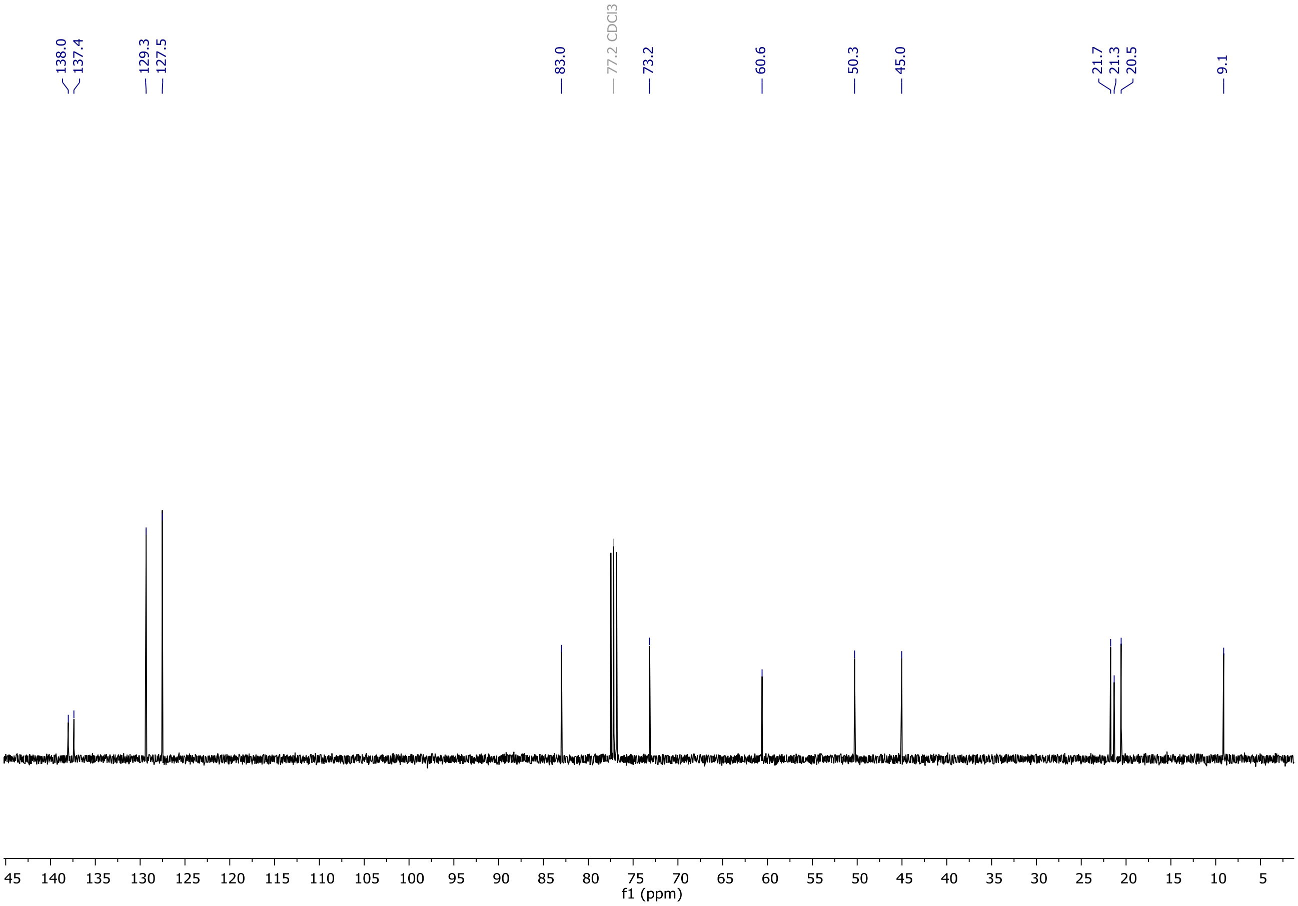

2D-COSY

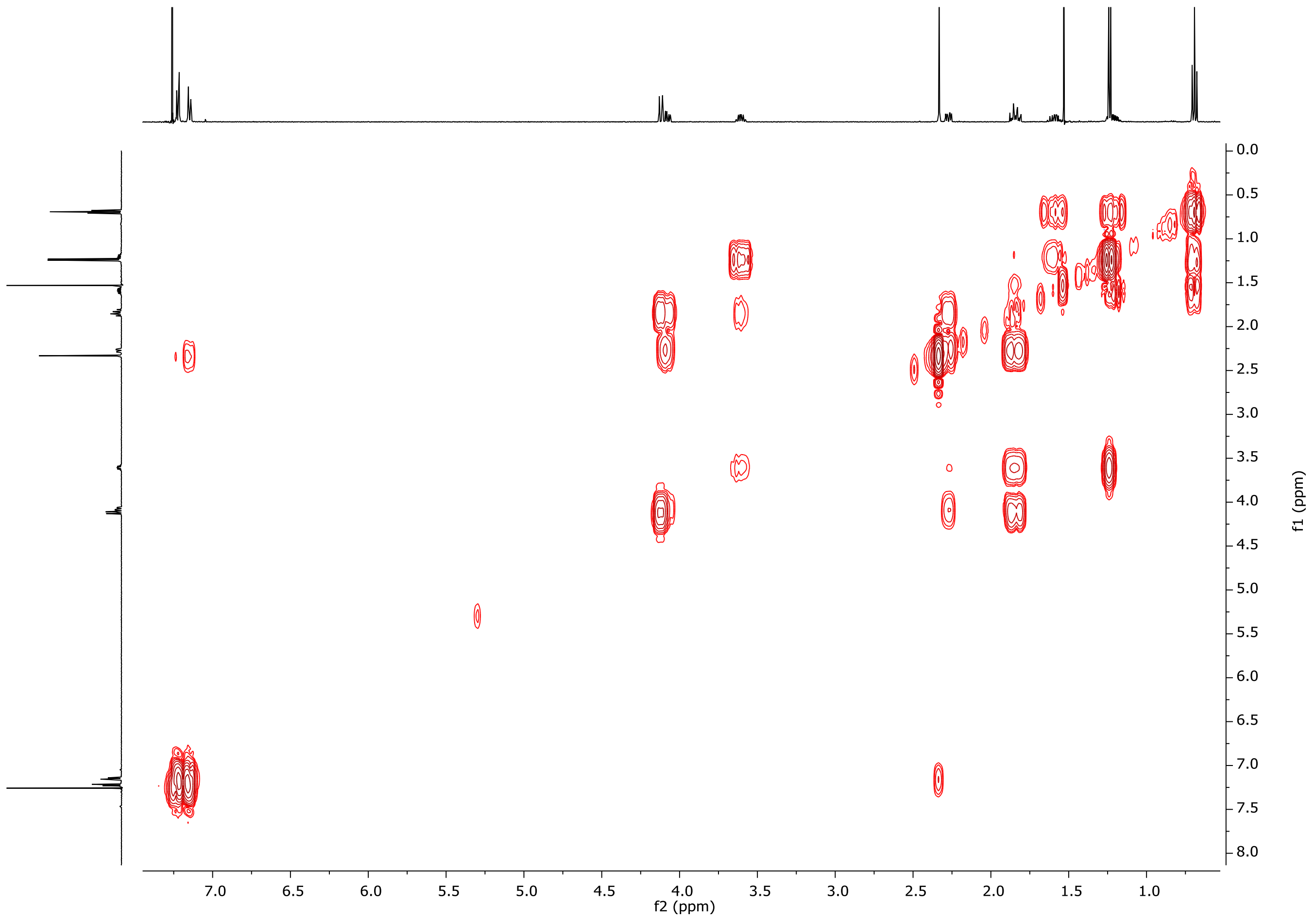

2D-HSQC

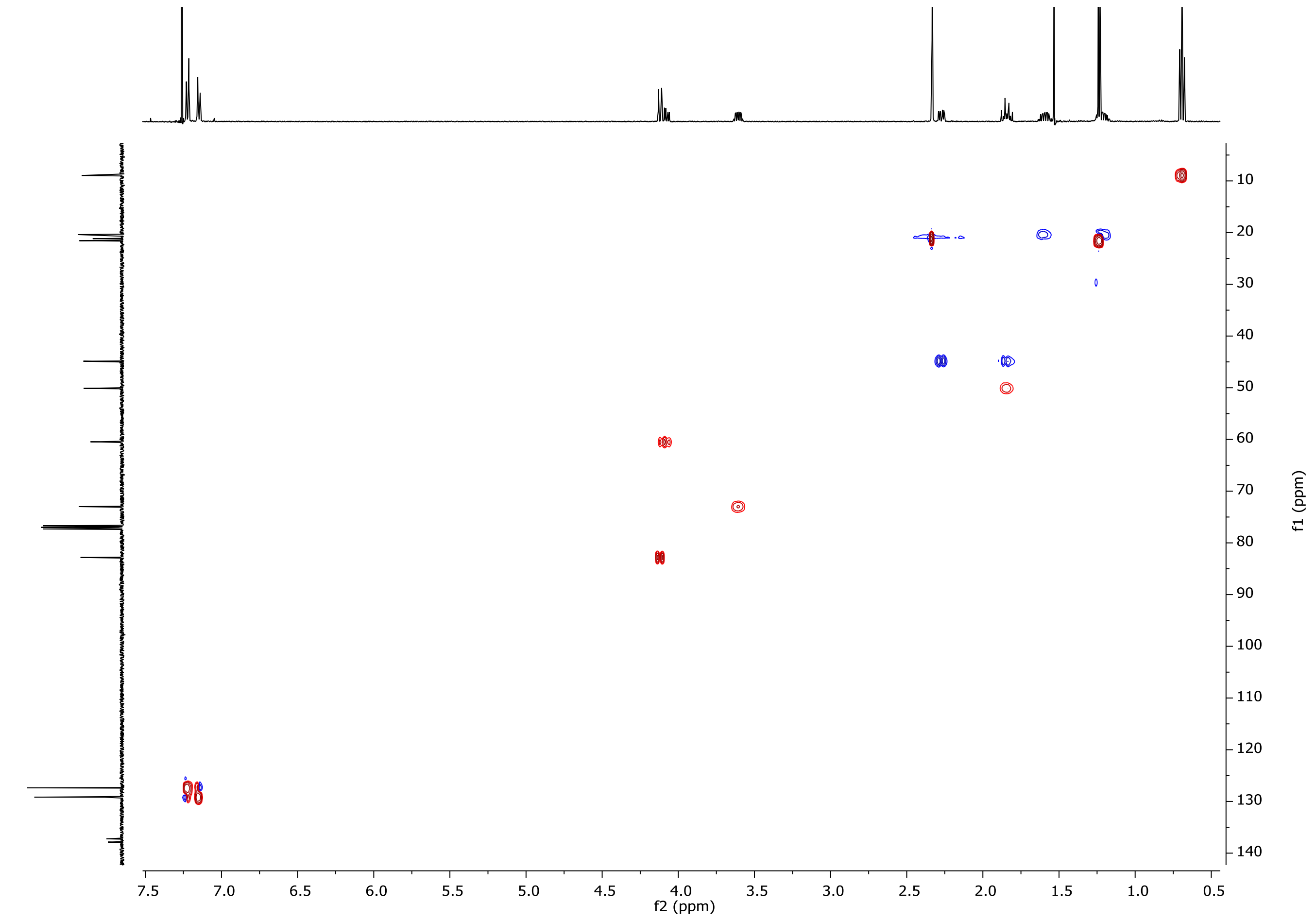

Compound 4p

^{1}H NMR (500 MHz, $CDCl_3$)

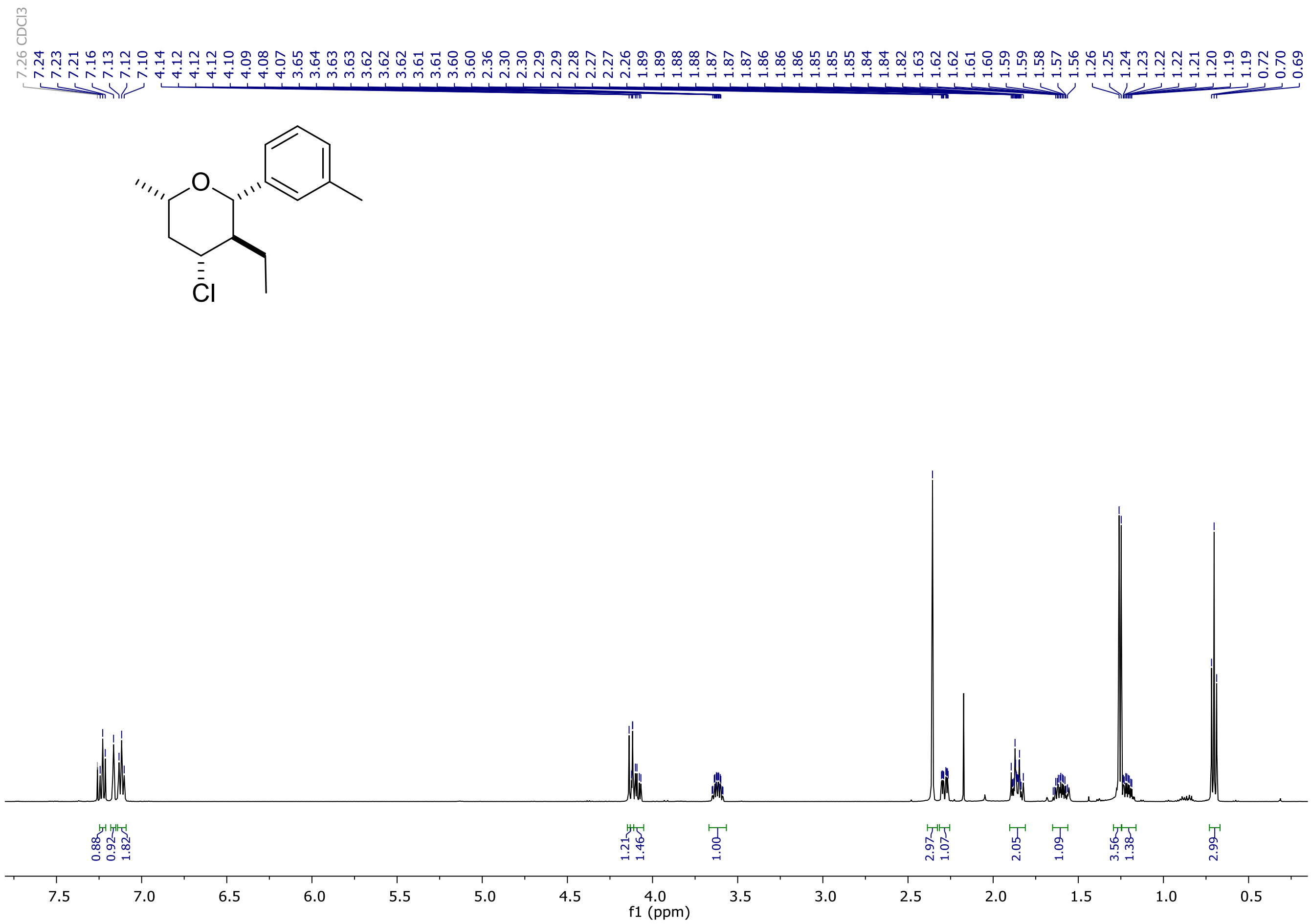

^{13}C NMR (101 MHz, $CDCl_3$)

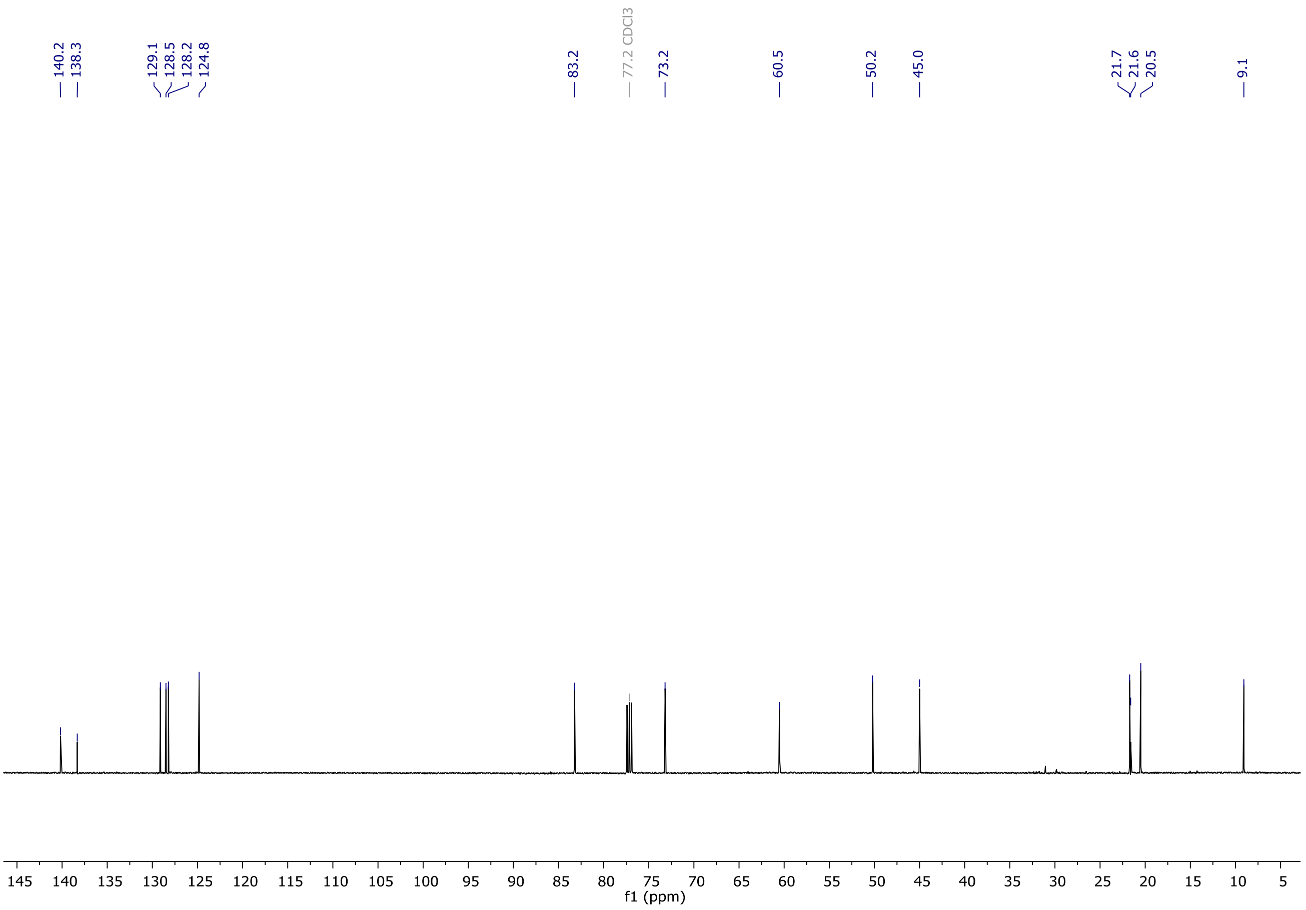

2D-COSY

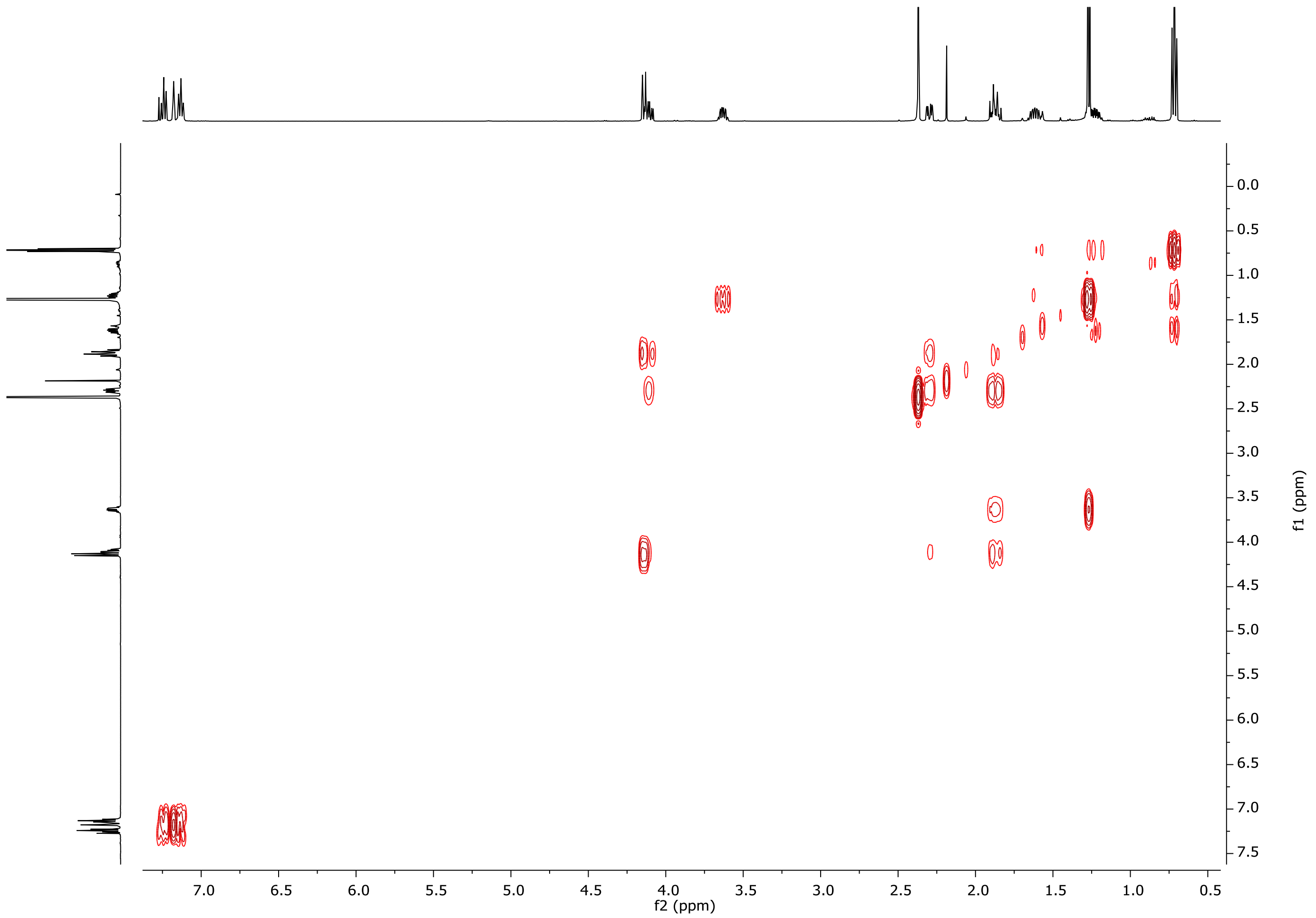

2D-HSQC

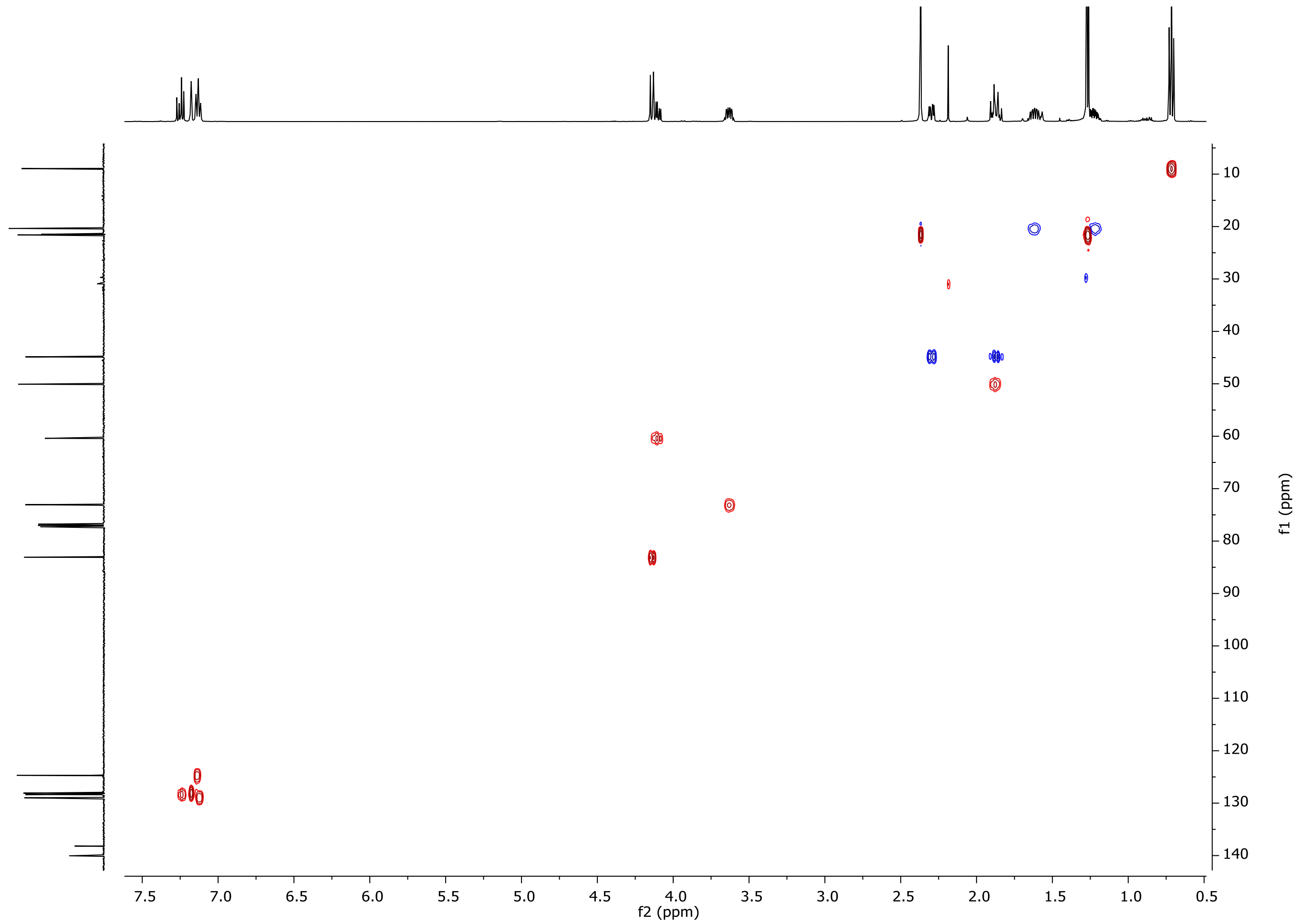

Compound 4q

^{1}**H NMR** (500 MHz, $CDCl_3$)

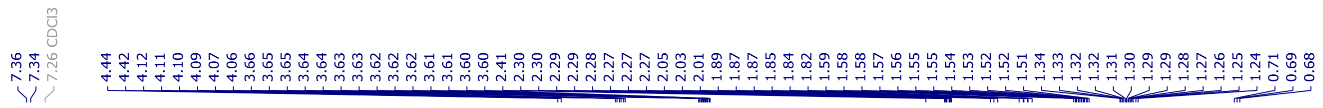

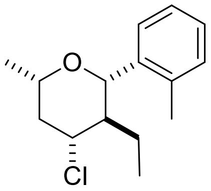

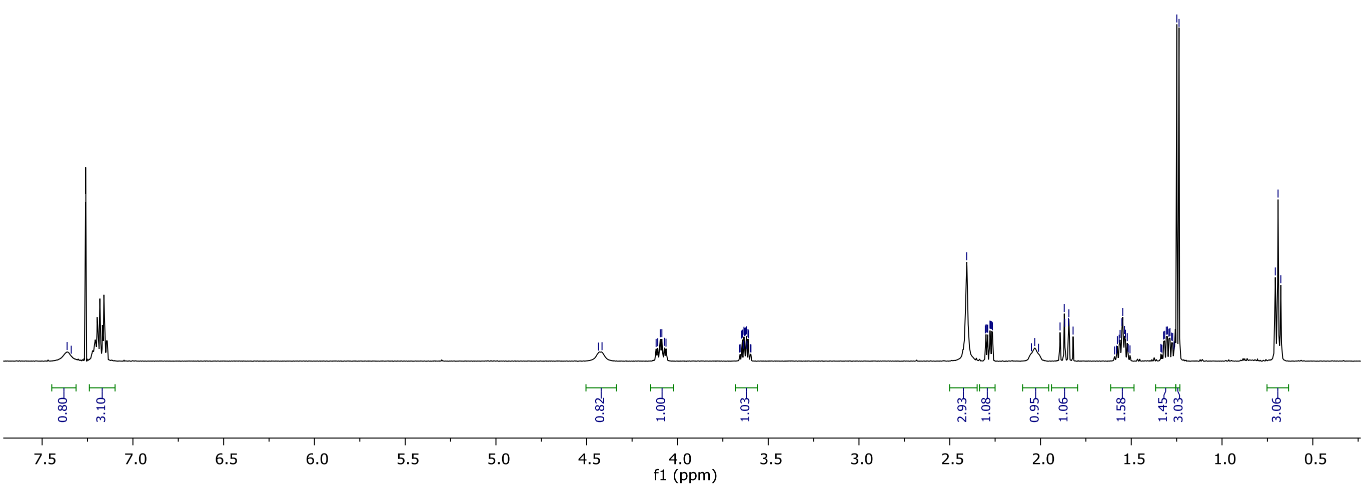

^{13}**C NMR** (101 MHz, $CDCl_3$)

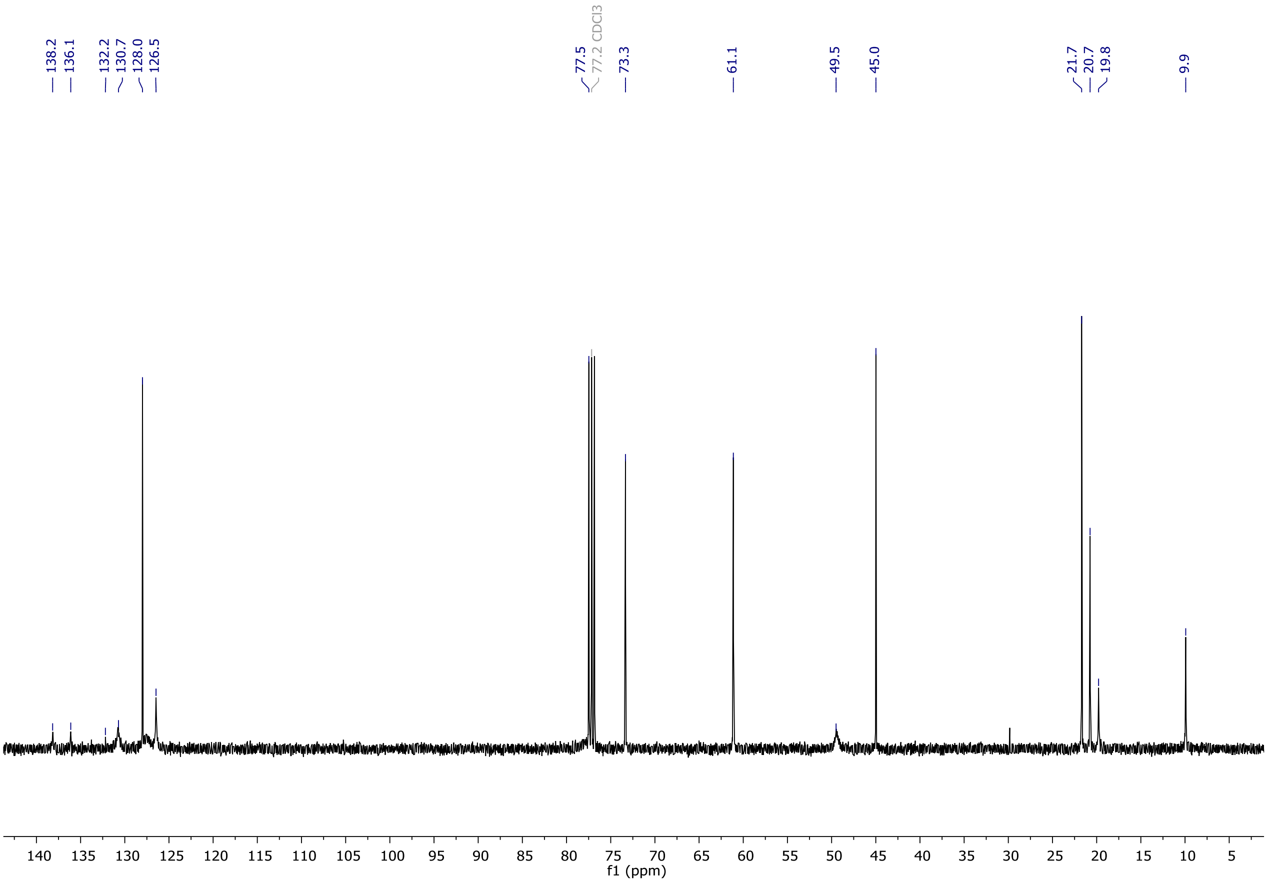

2D-COSY

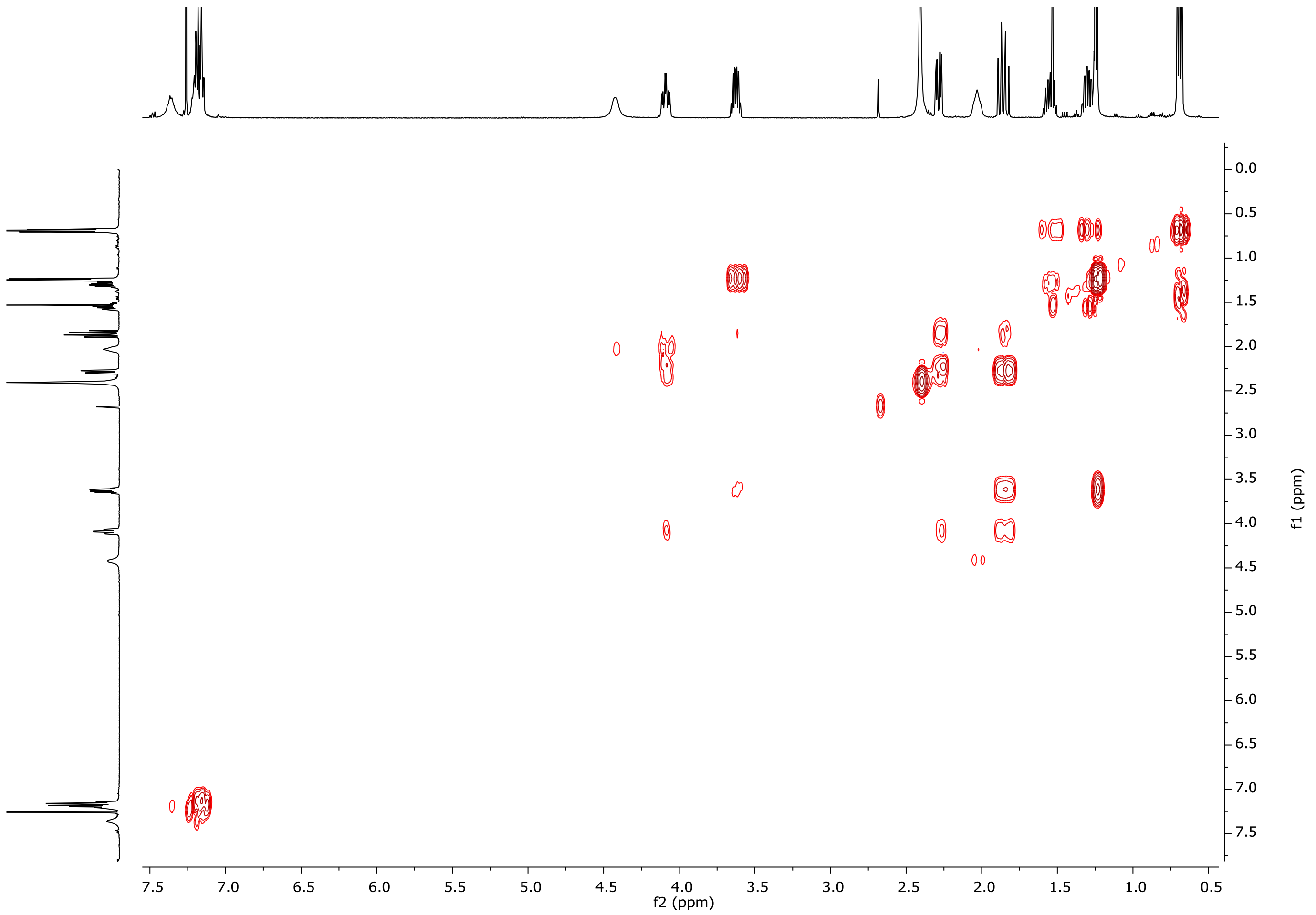

2D-HSQC

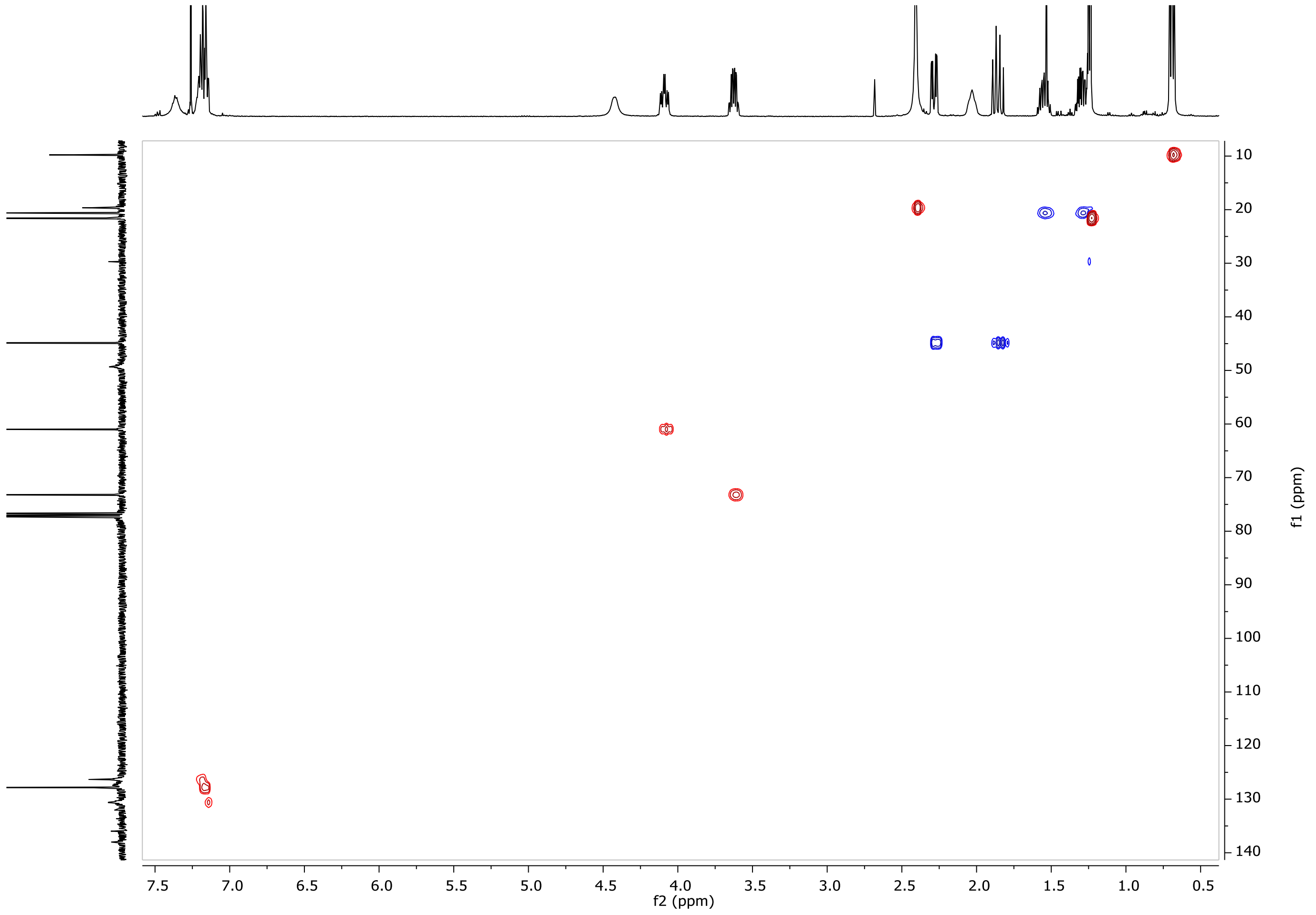

Compound 4r

^{1}**H NMR** (500 MHz, $CDCl_3$)

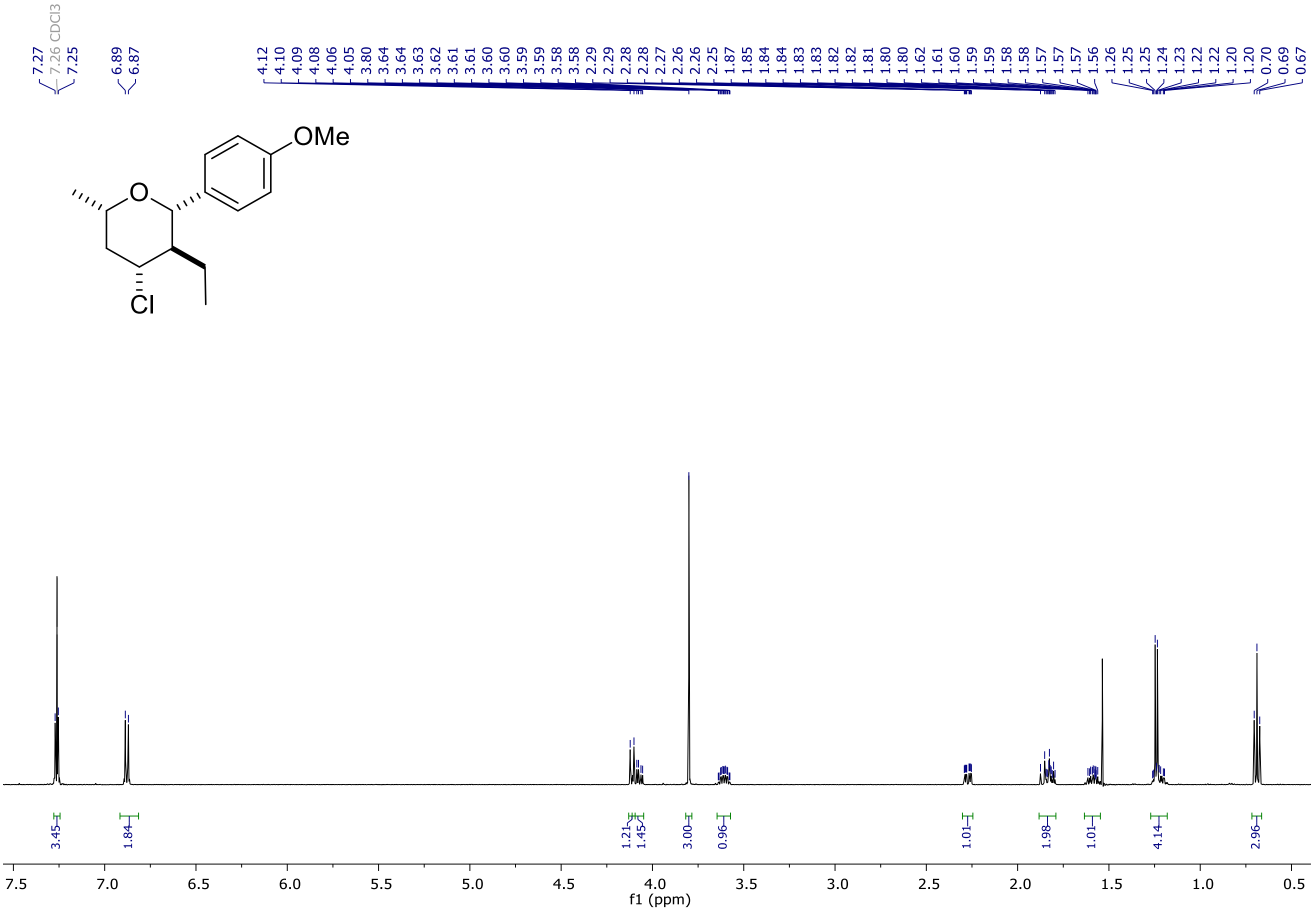

^{13}**C NMR** (101 MHz, $CDCl_3$)

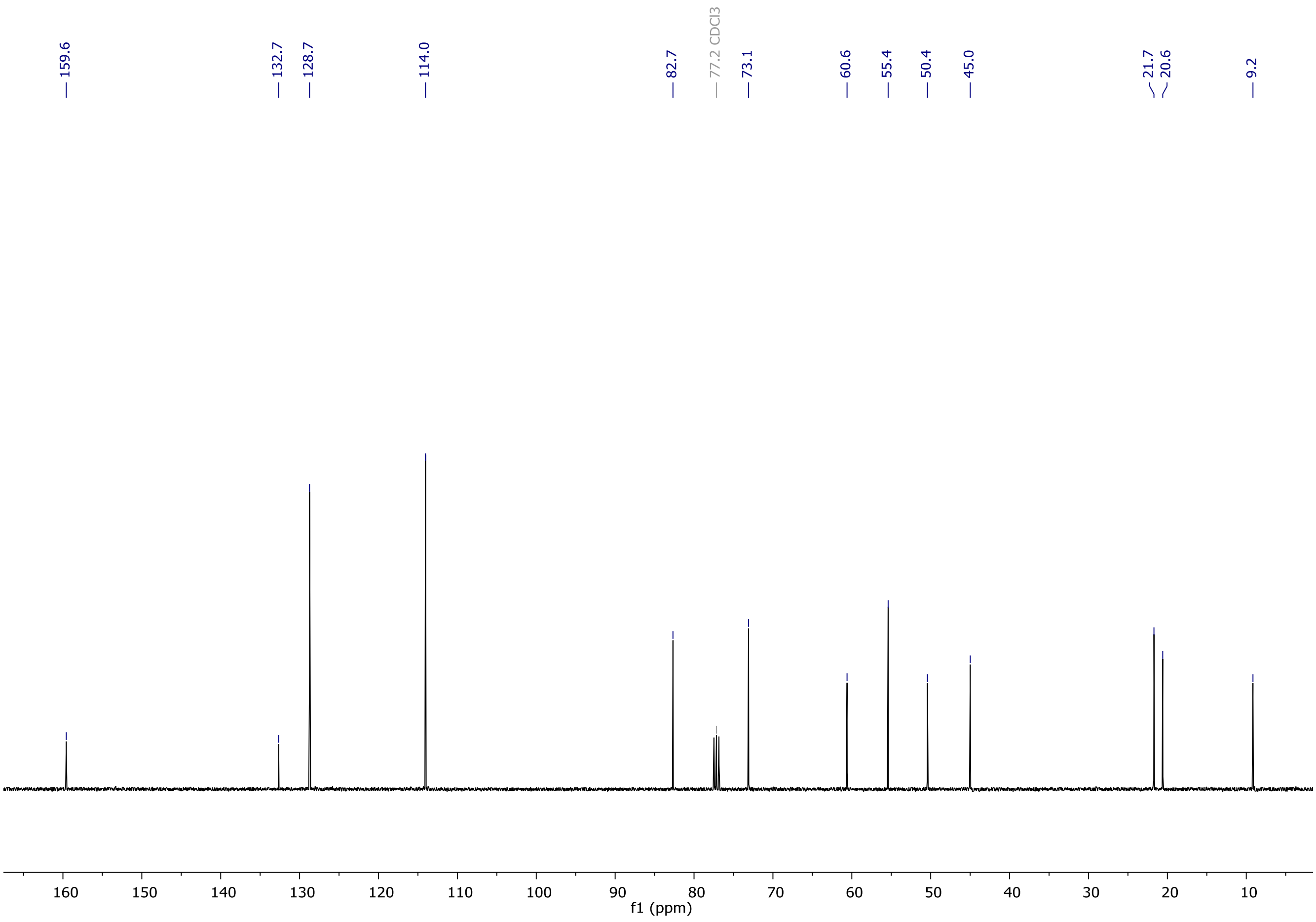

2D-COSY

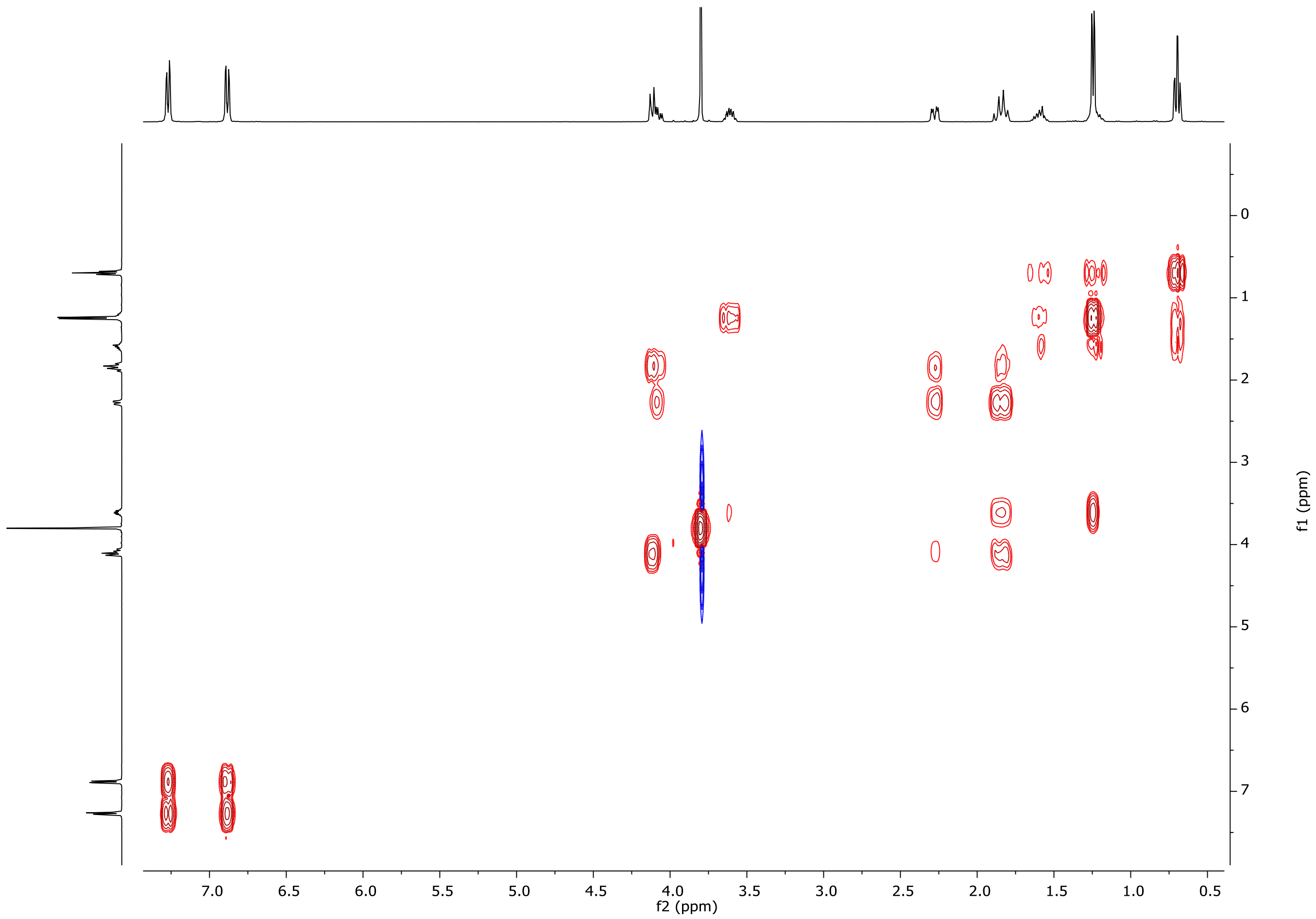

2D-HSQC

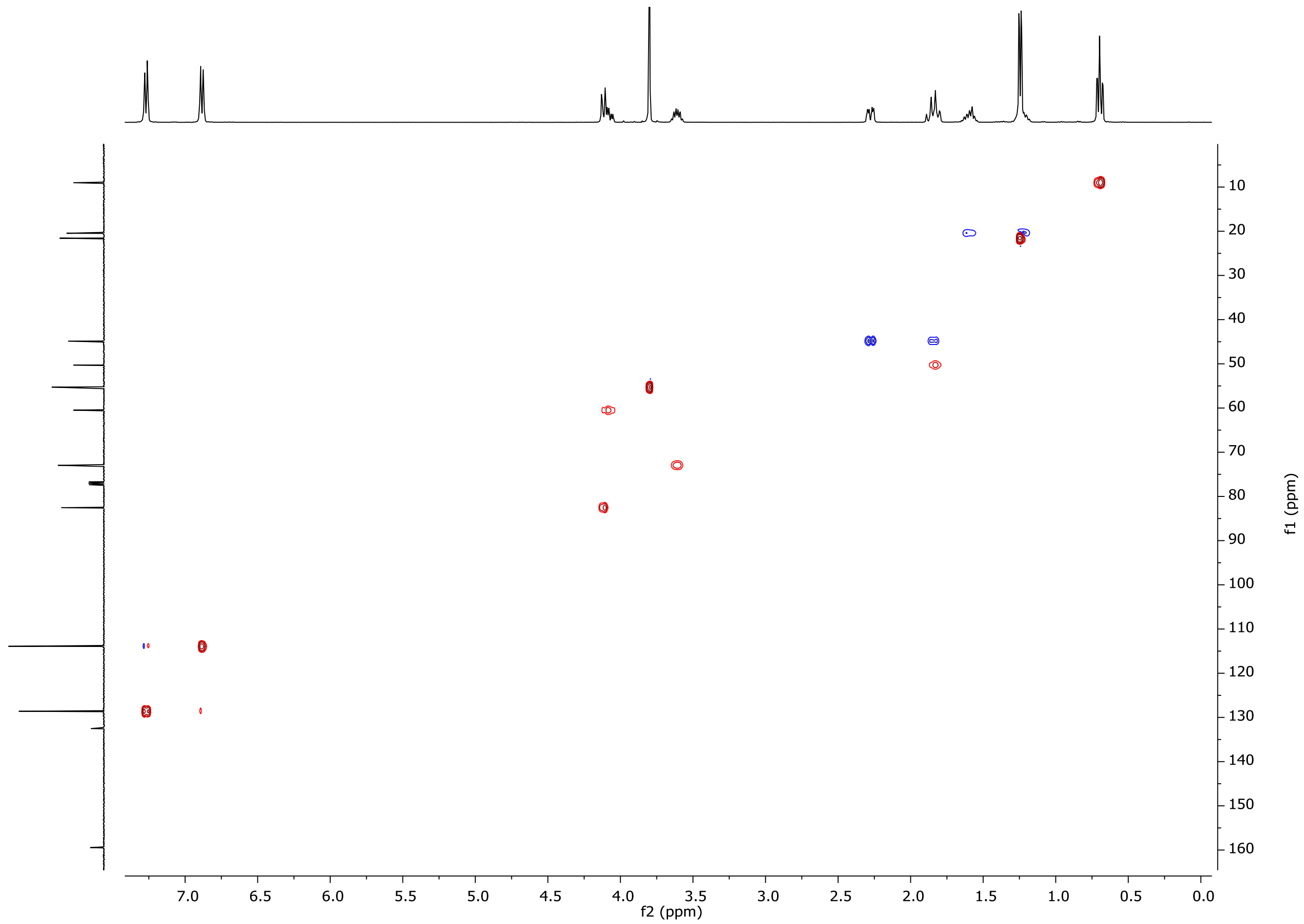

Compound 4s

^{1}H NMR (500 MHz, $CDCl_3$)

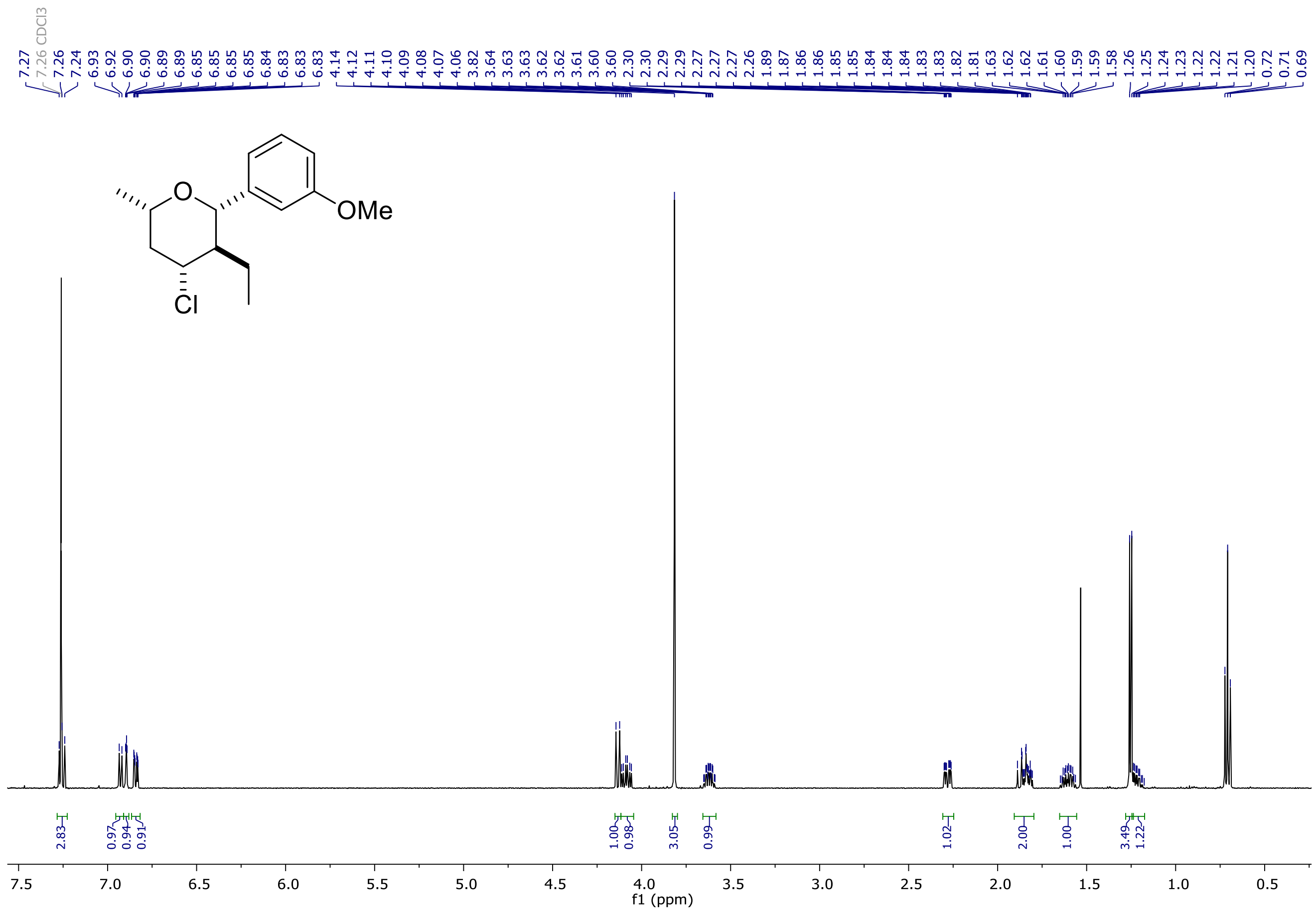

^{13}C NMR (101 MHz, $CDCl_3$)

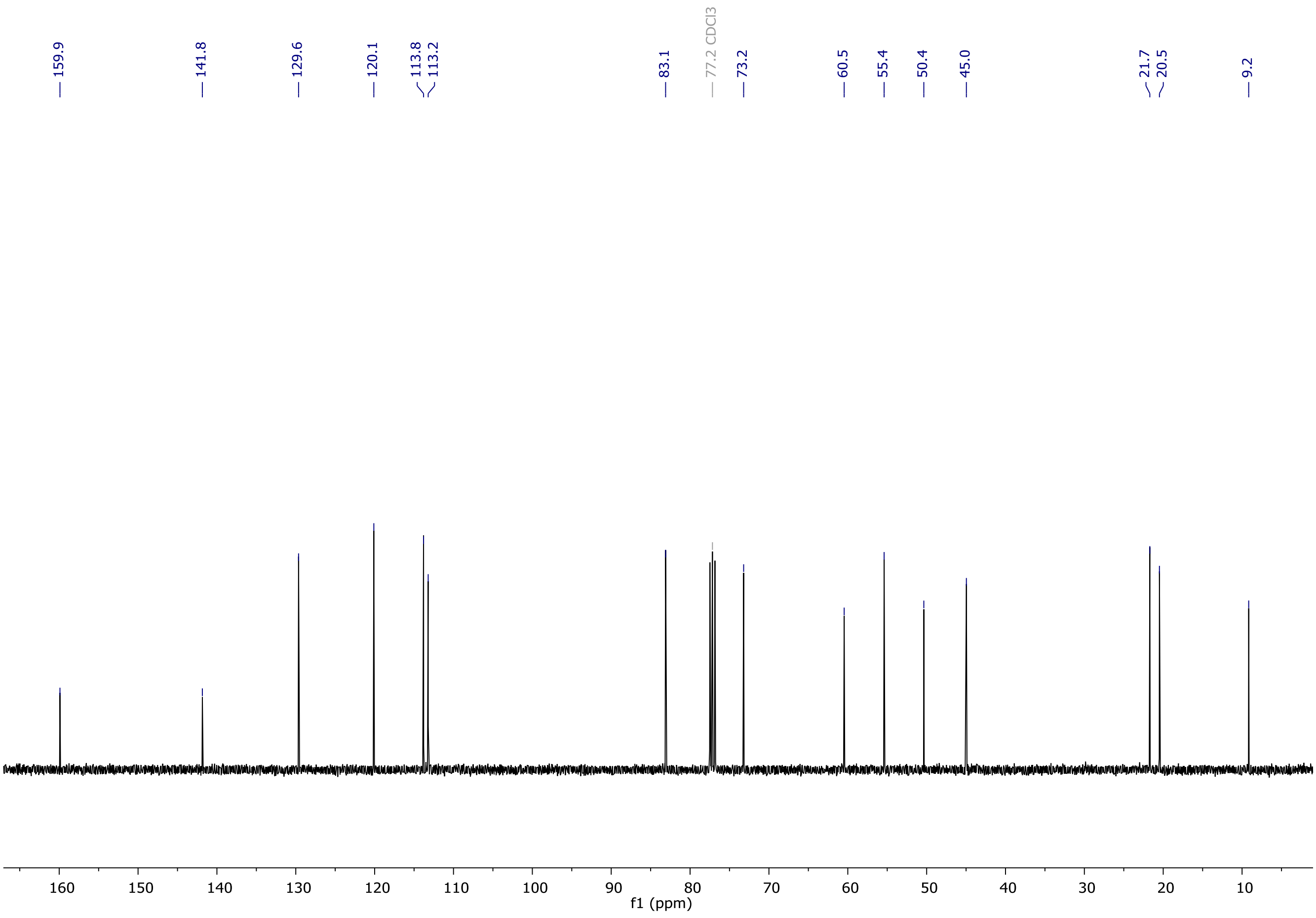

2D-COSY

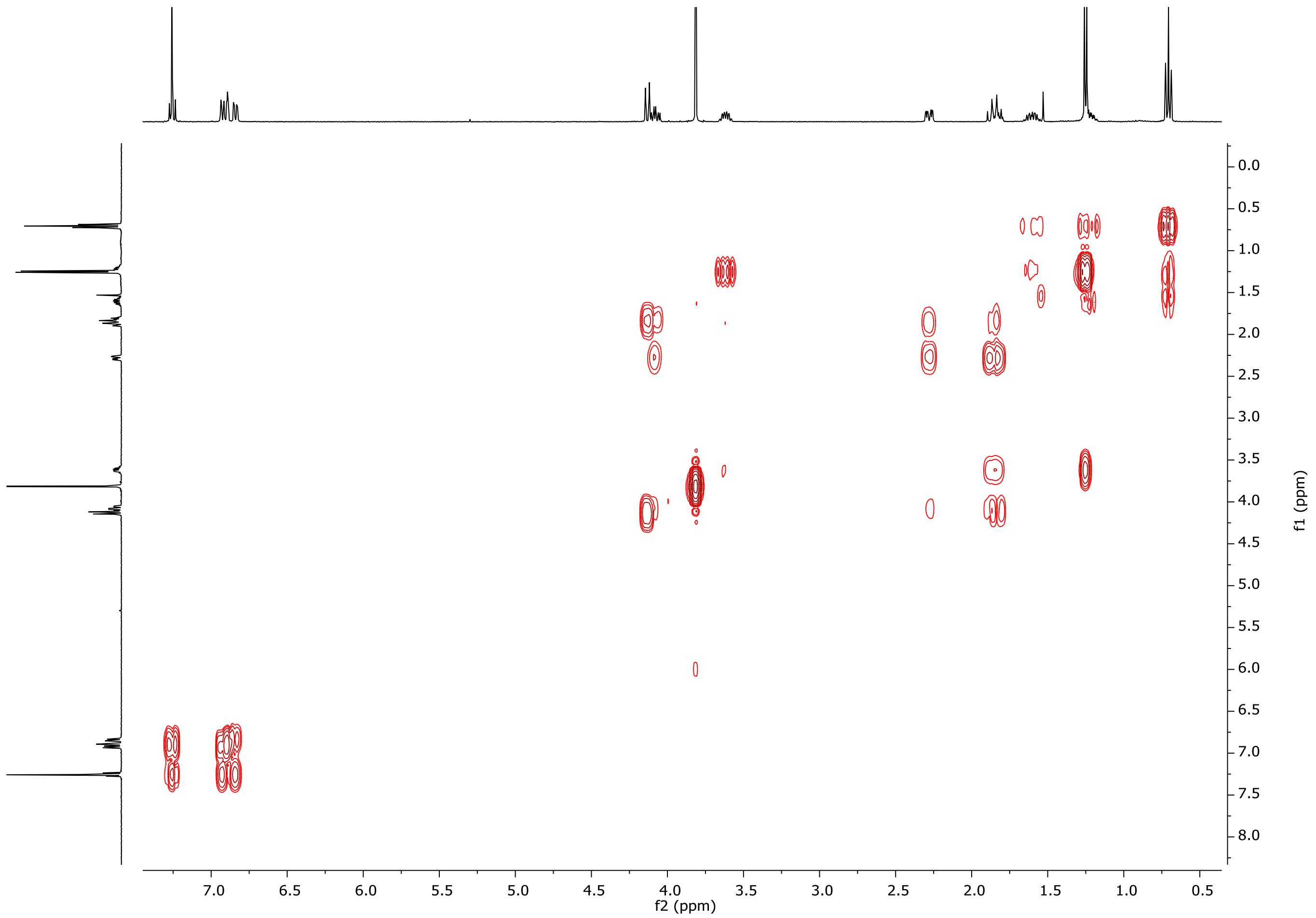

2D-HSQC

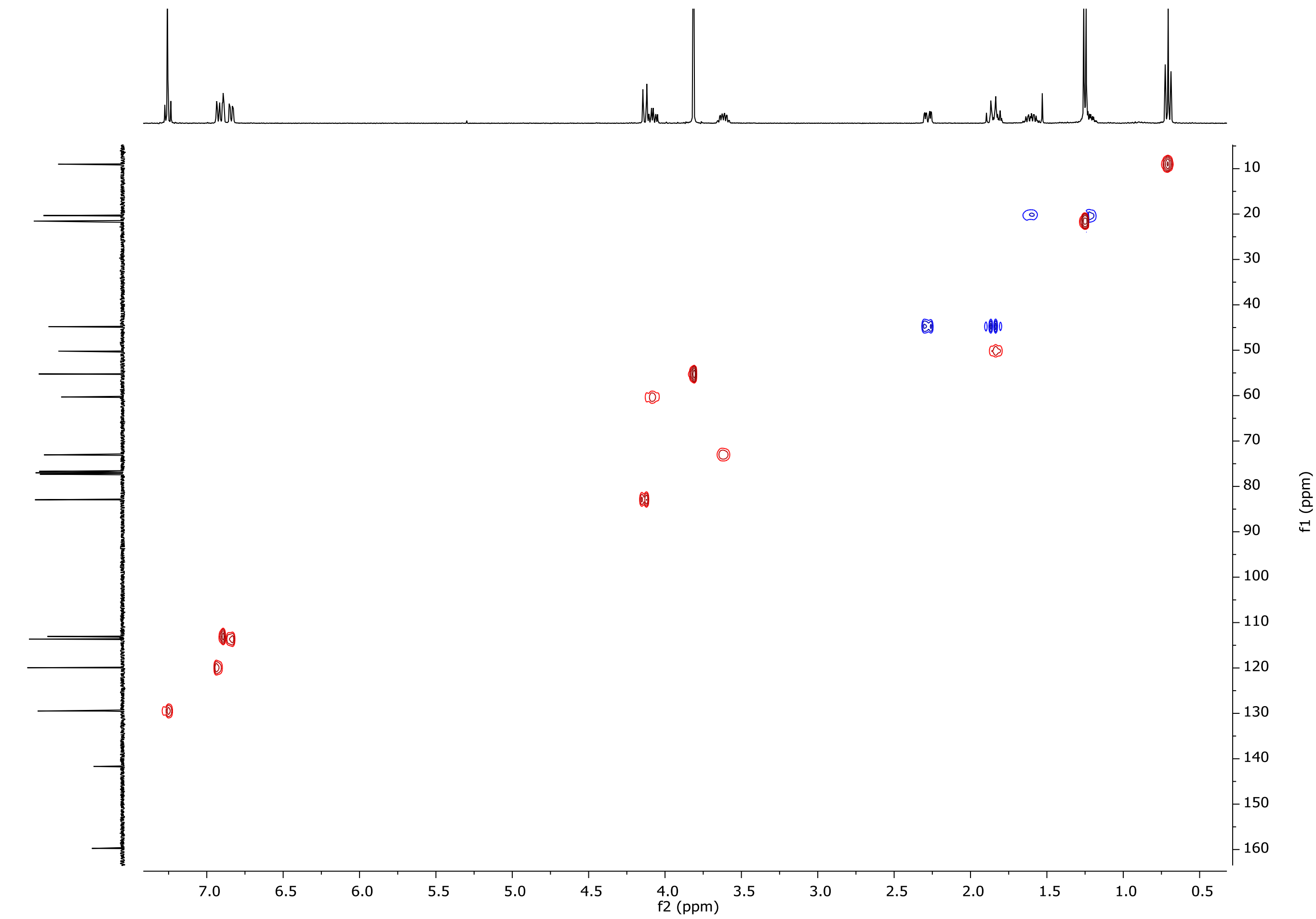

Compound 4t

^{1}**H NMR** (500 MHz, $CDCl_3$)

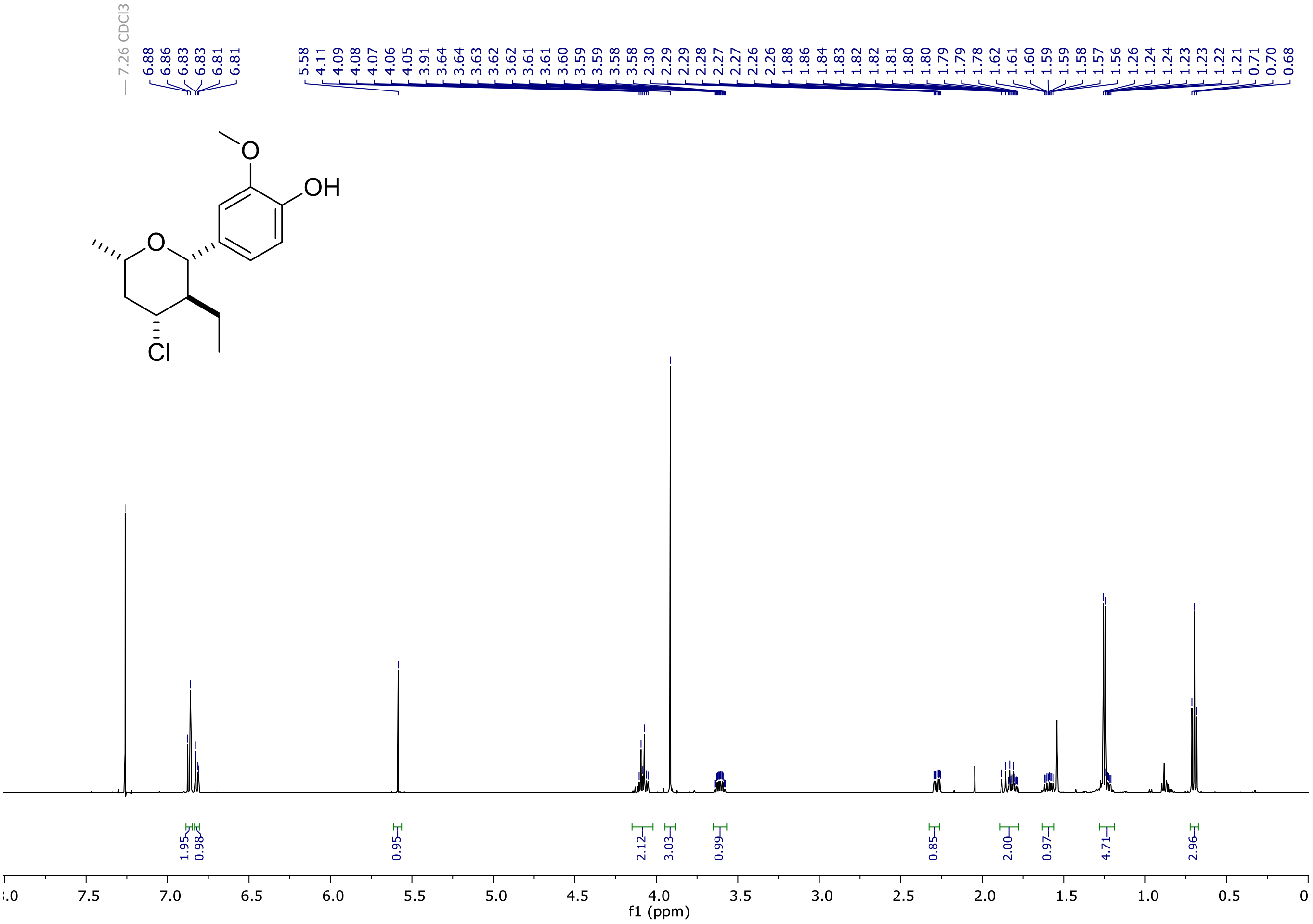

^{13}**C NMR** (101 MHz, $CDCl_3$)

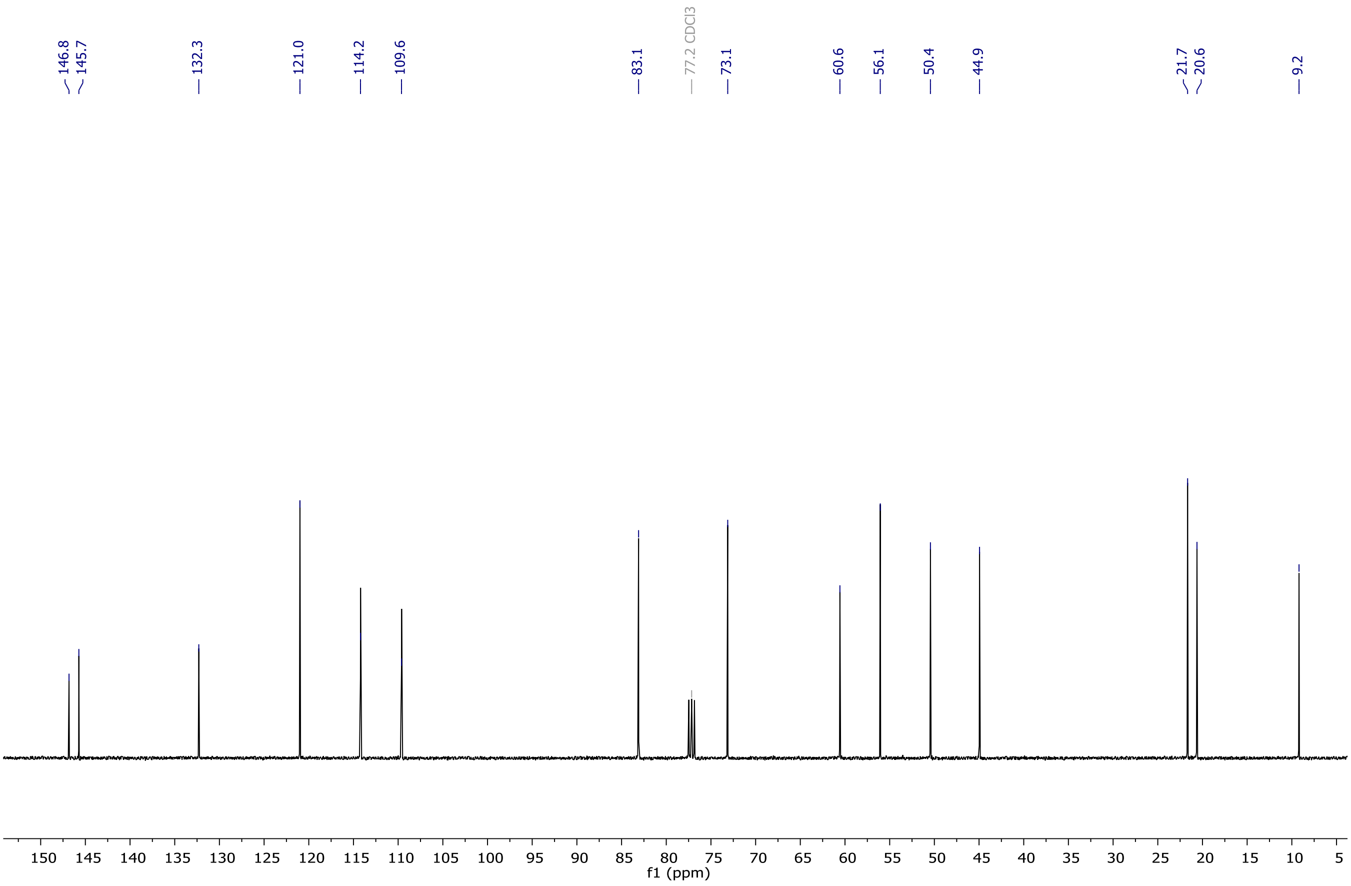

2D-COSY

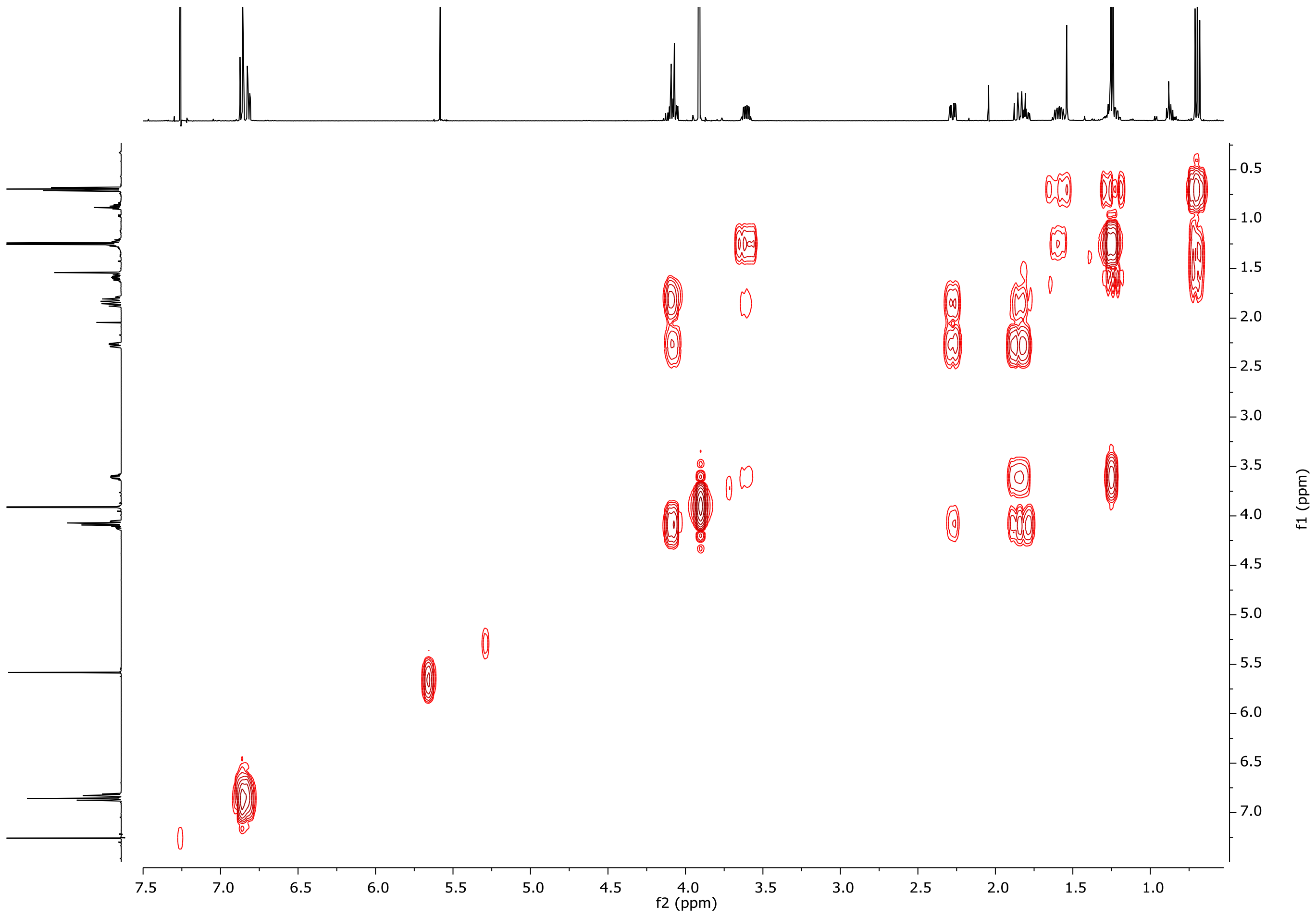

2D-HSQC

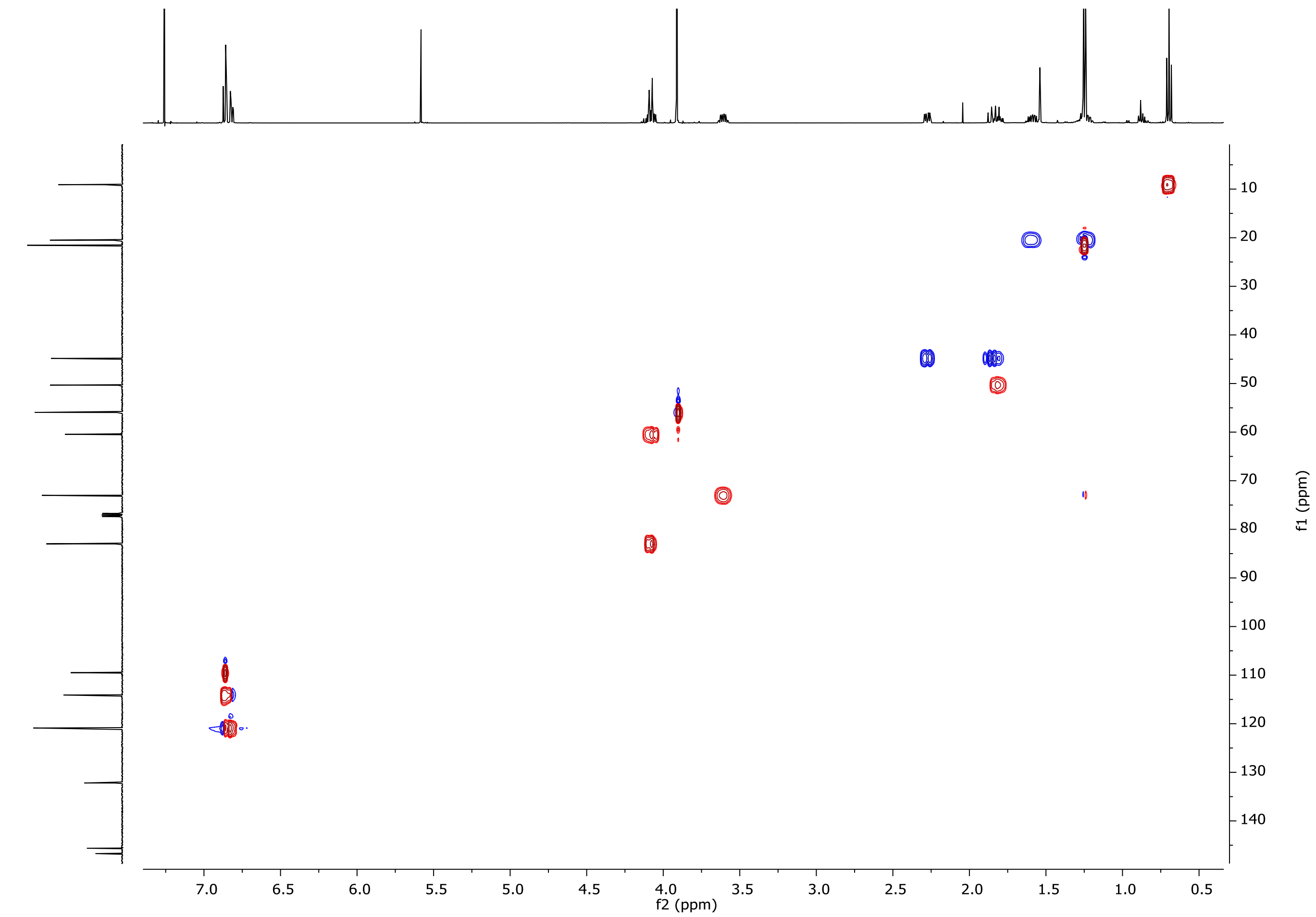

Compound 4u

^{1}H NMR (500 MHz, $CDCl_3$)

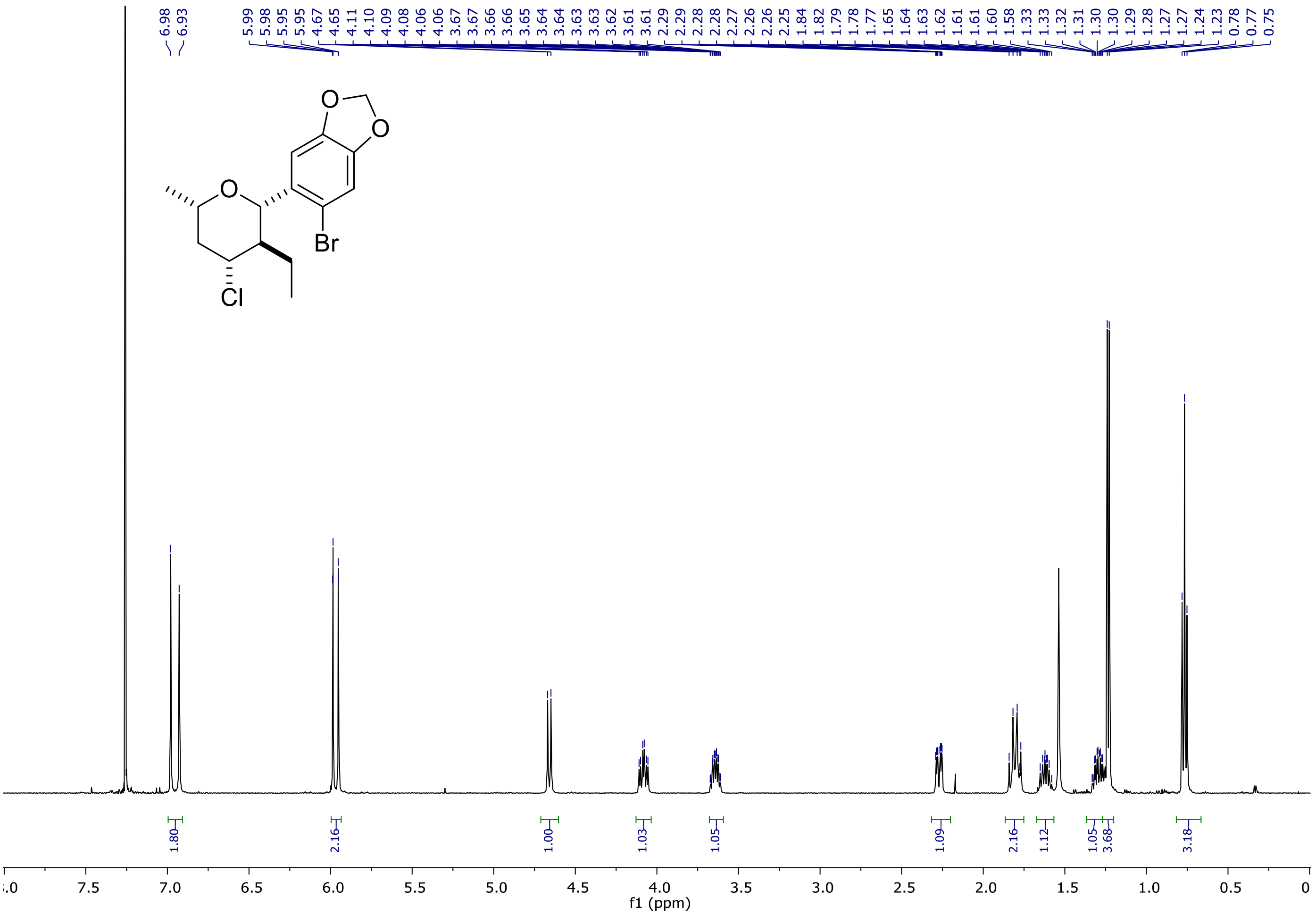

^{13}C NMR (101 MHz, $CDCl_3$)

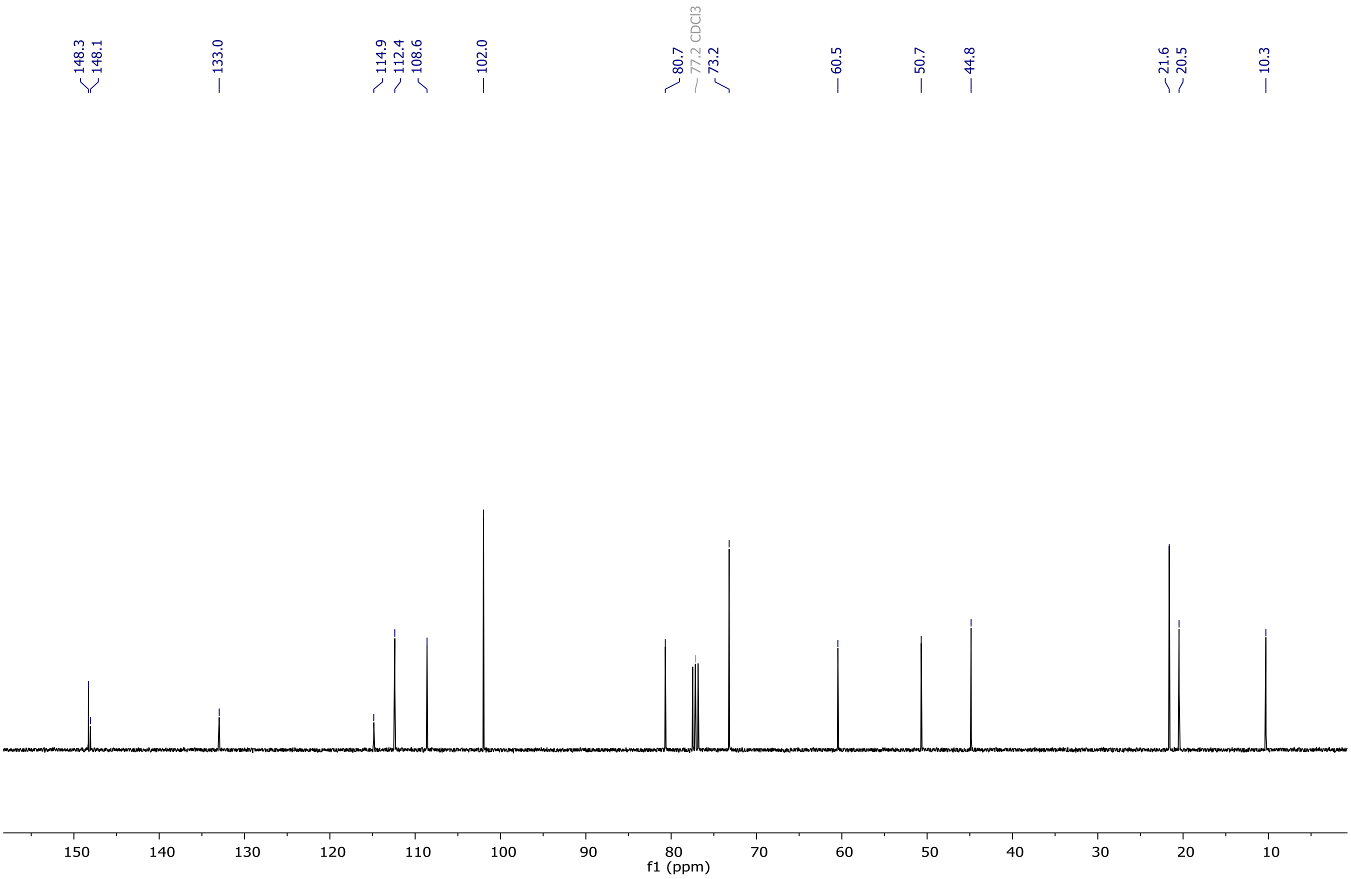

2D-COSY

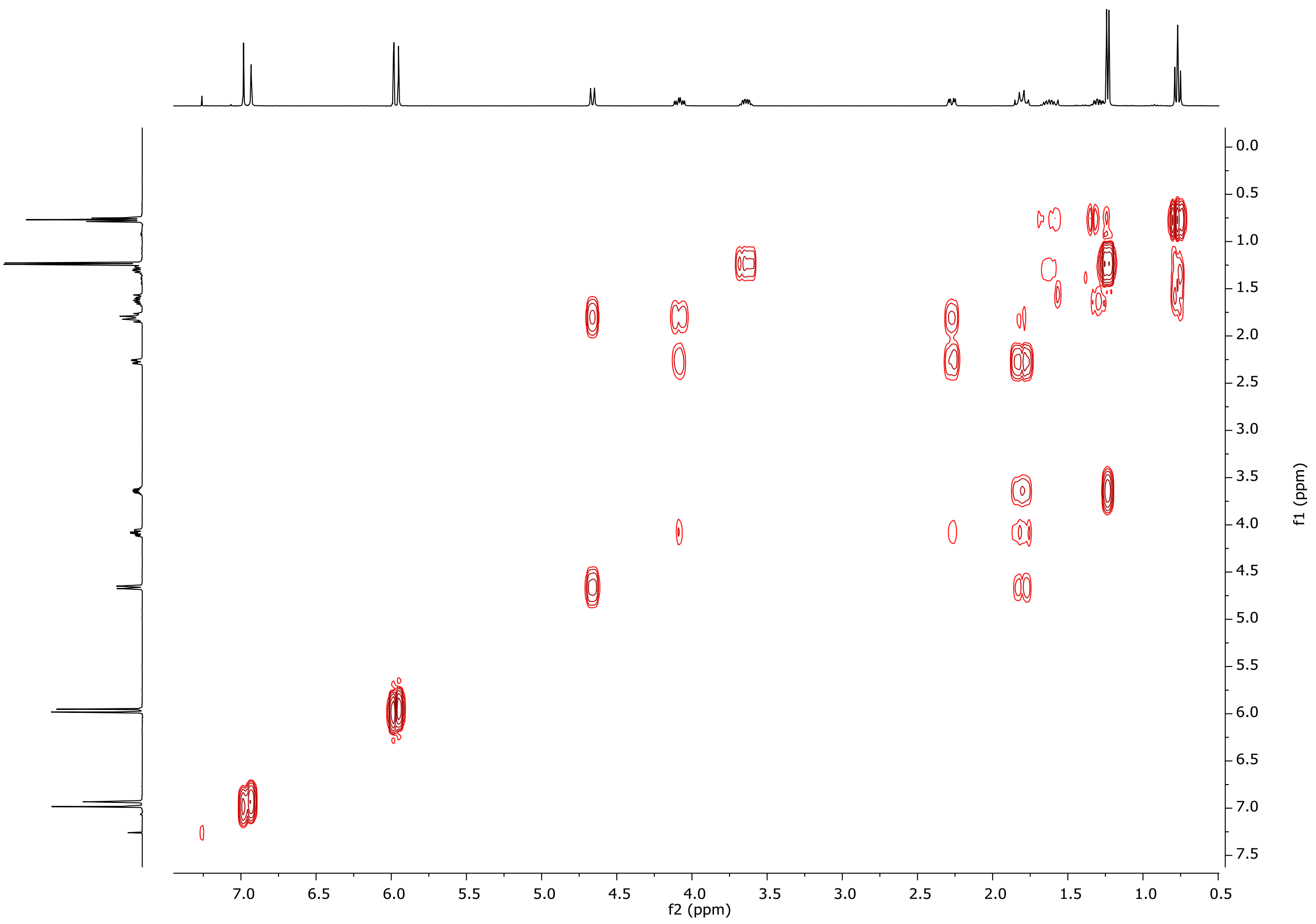

2D-HQSC

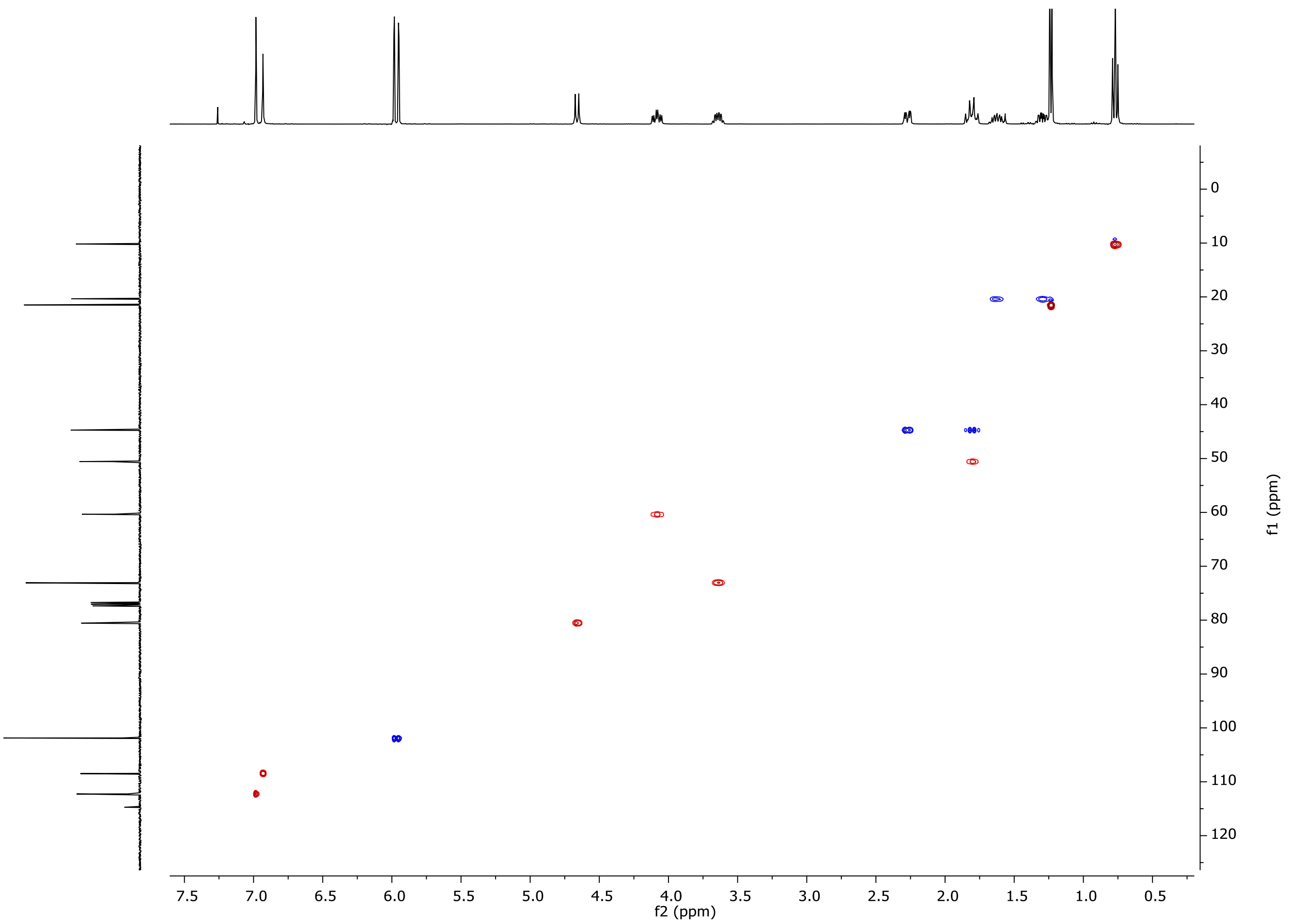

Compound 4v

^{1}H NMR (500 MHz, $CDCl_3$)

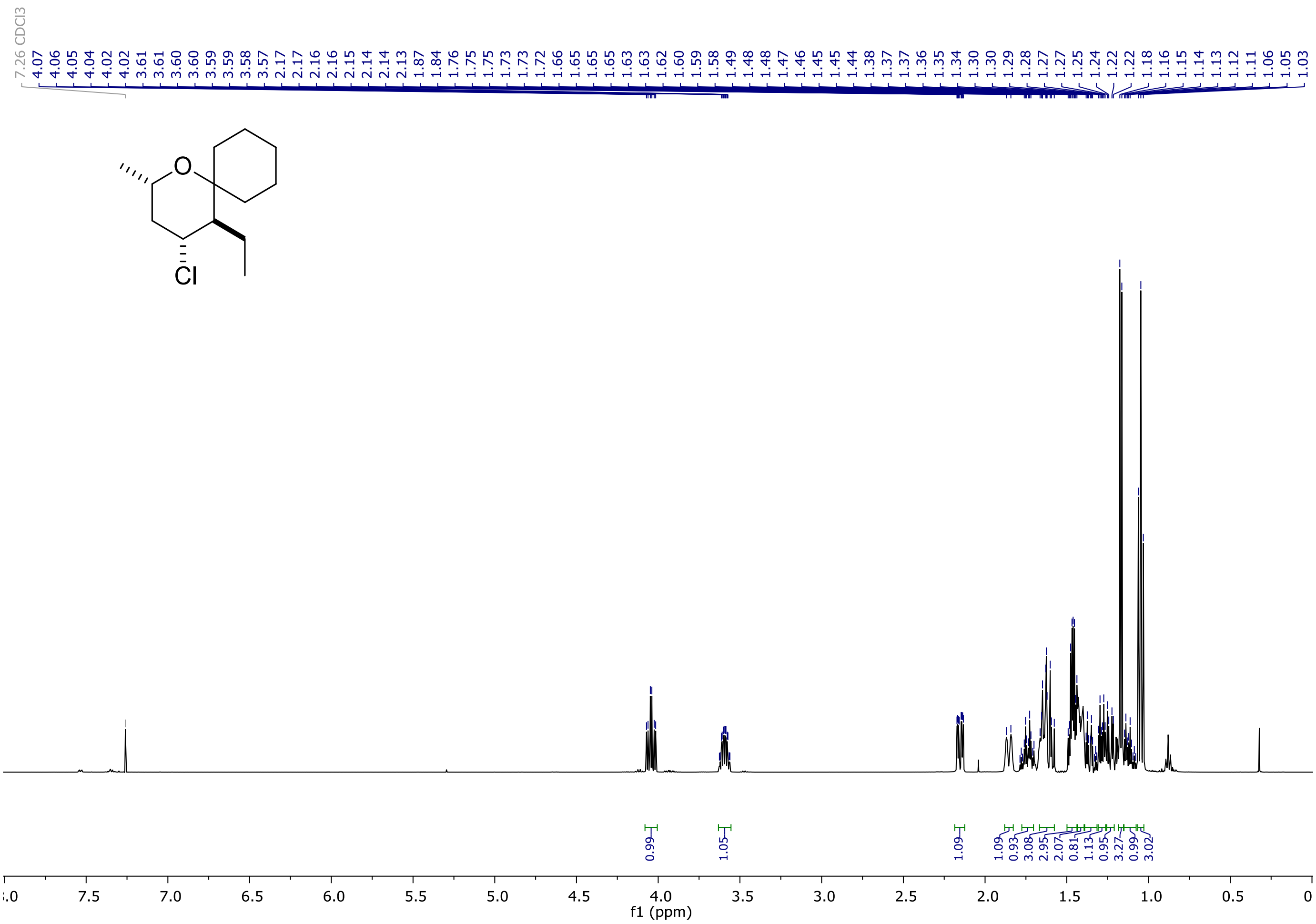

^{13}C NMR (101 MHz, $CDCl_3$)

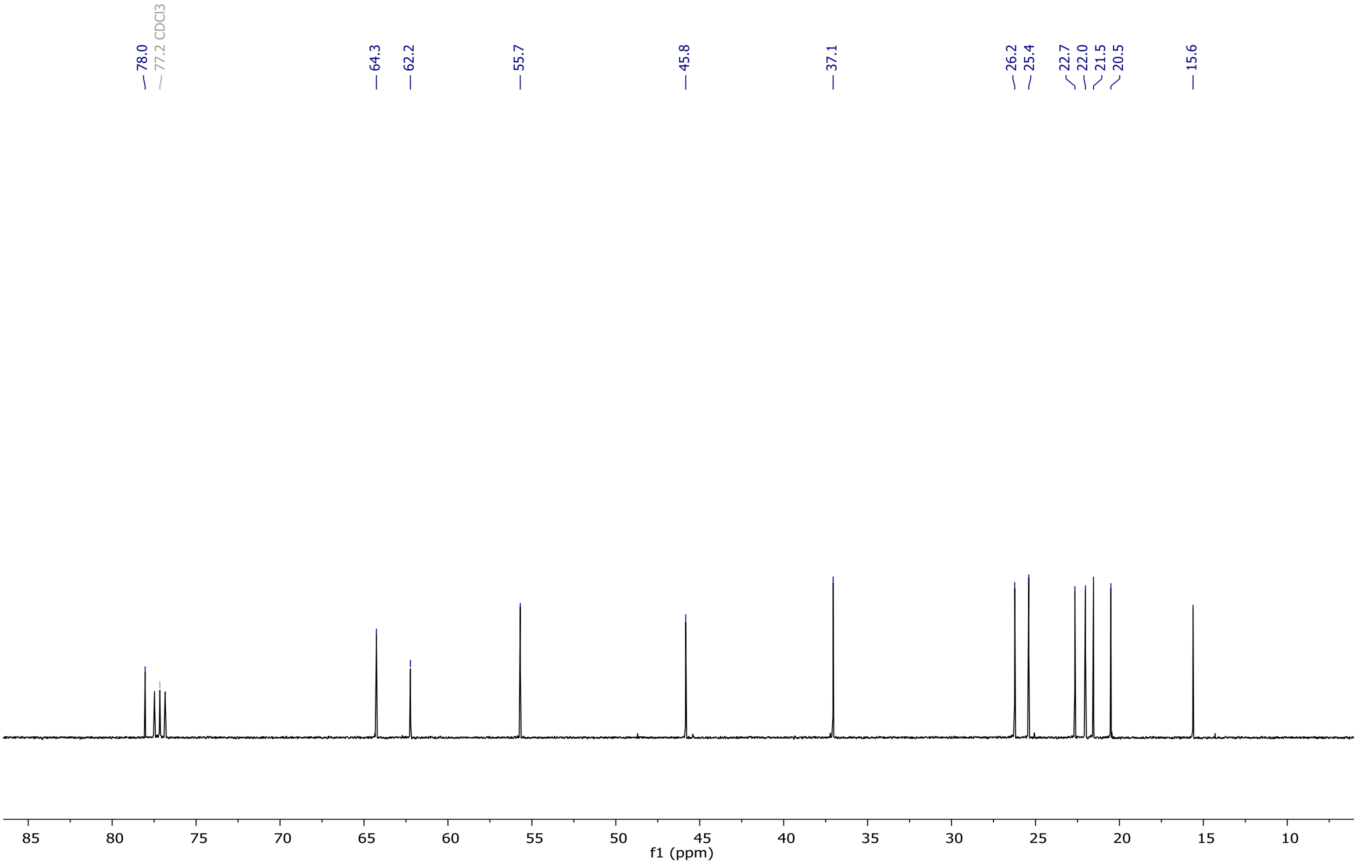

2D-COSY

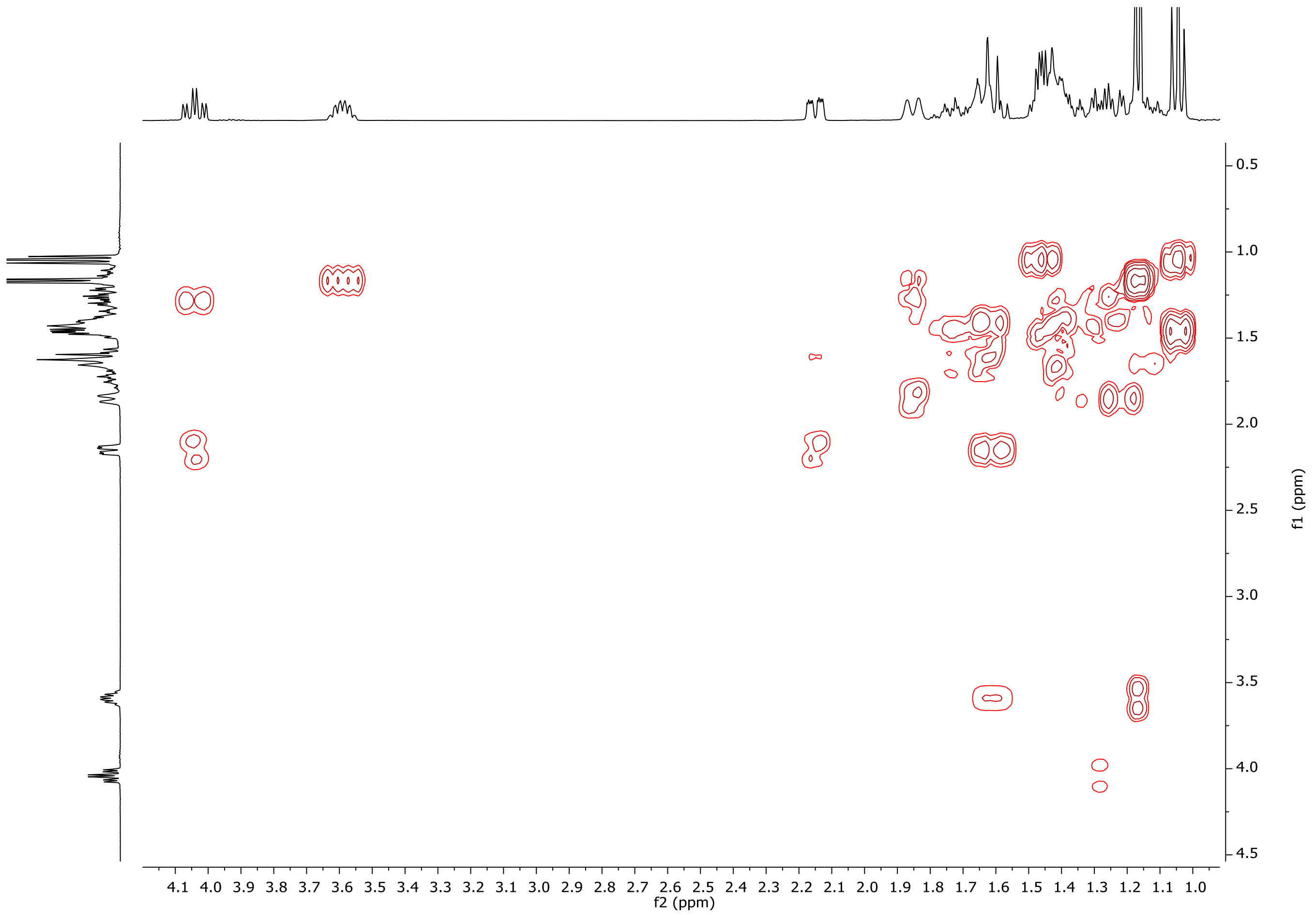

2D-HSQC

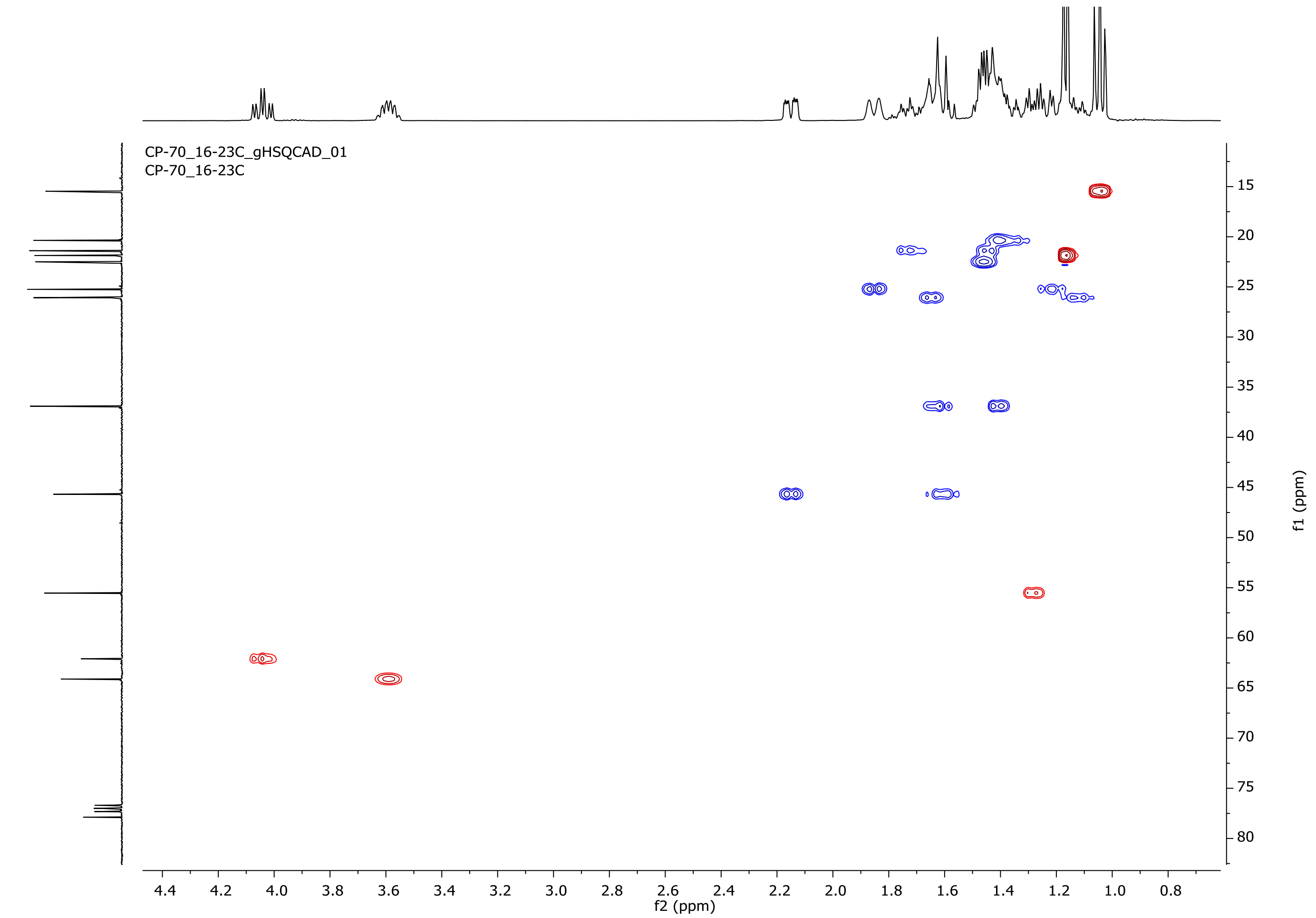

Compound 4w

^{1}H NMR (500 MHz, $CDCl_3$)

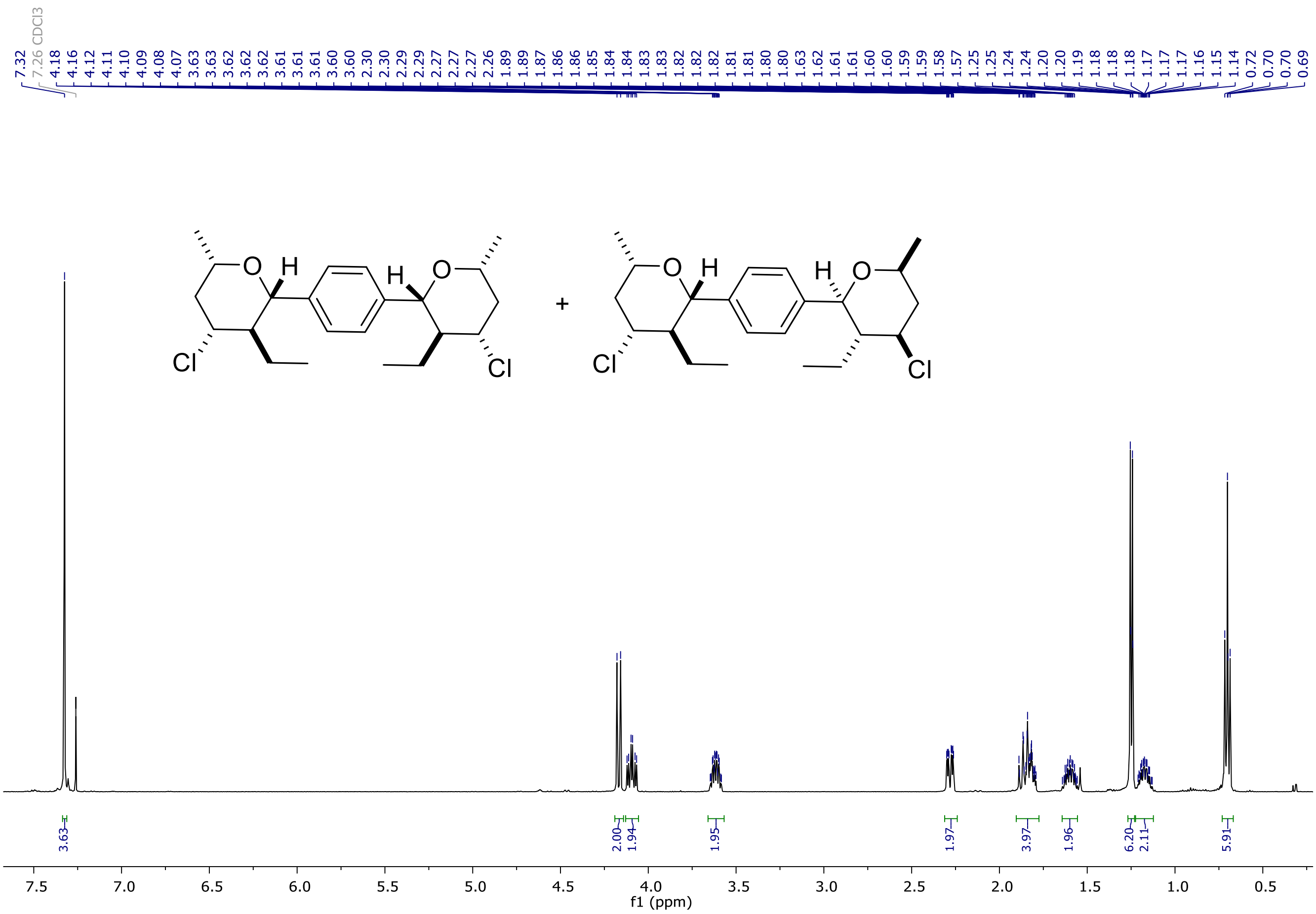

^{13}C NMR (101 MHz, $CDCl_3$)

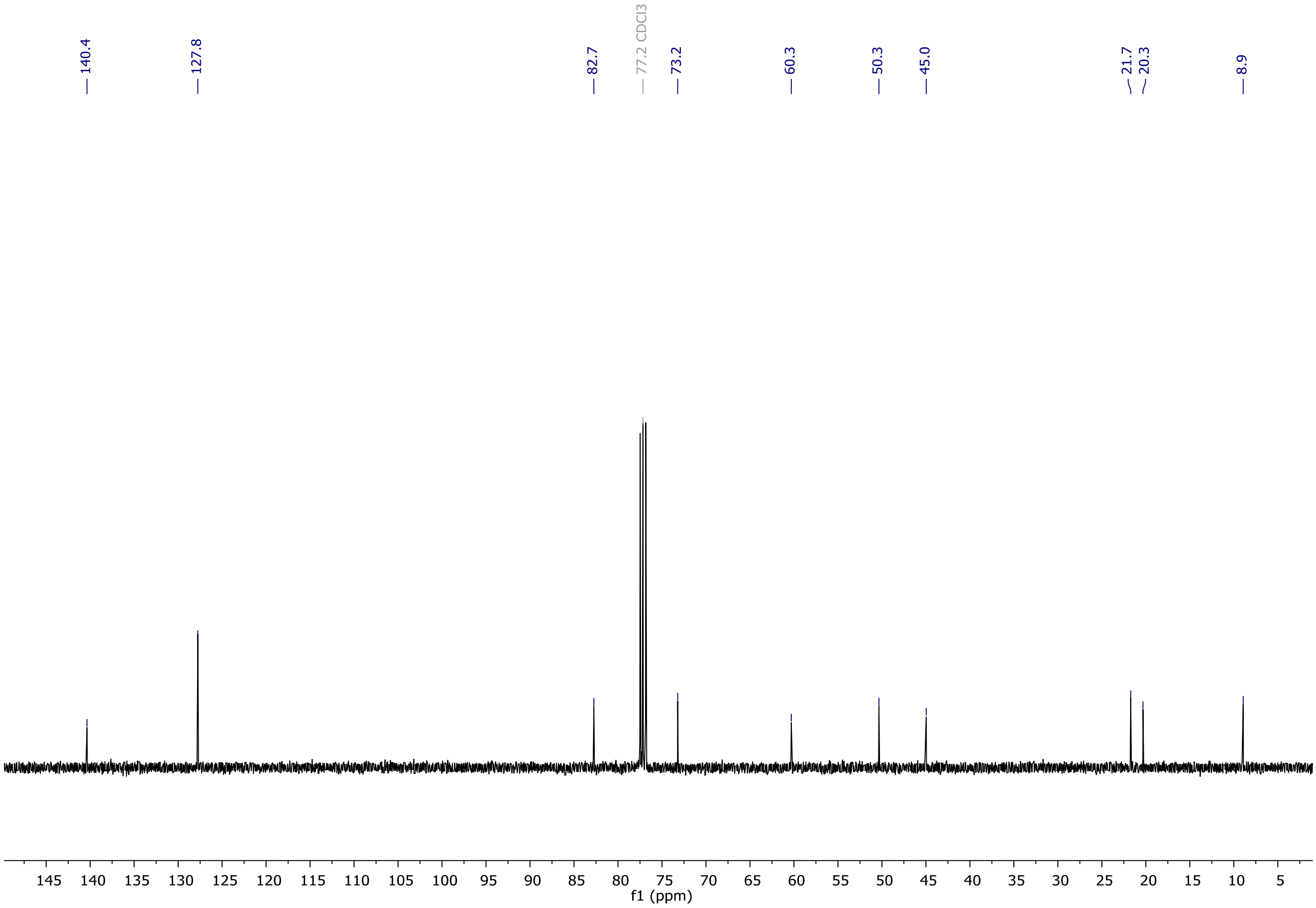

2D-COSY

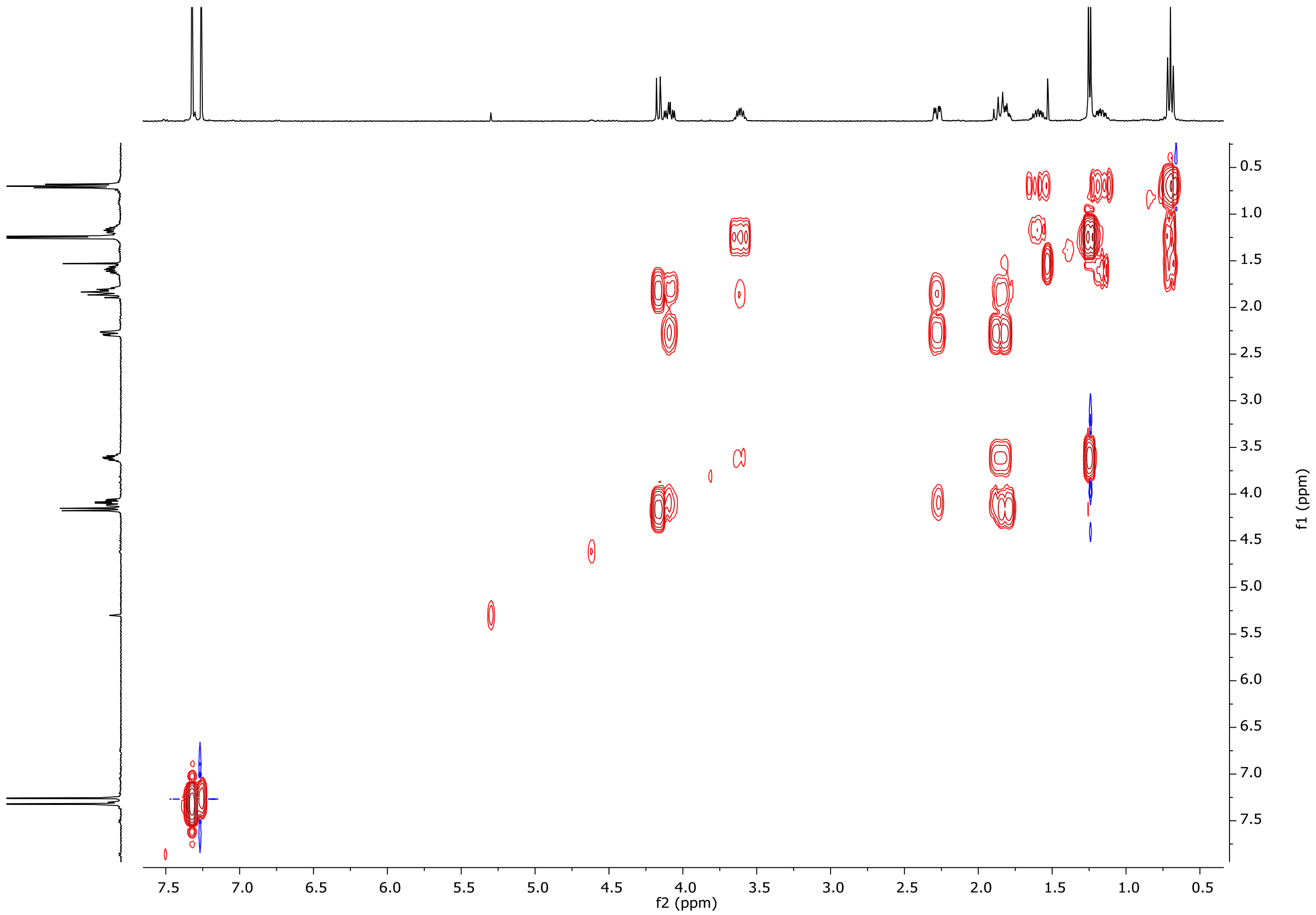

2D-HSQC

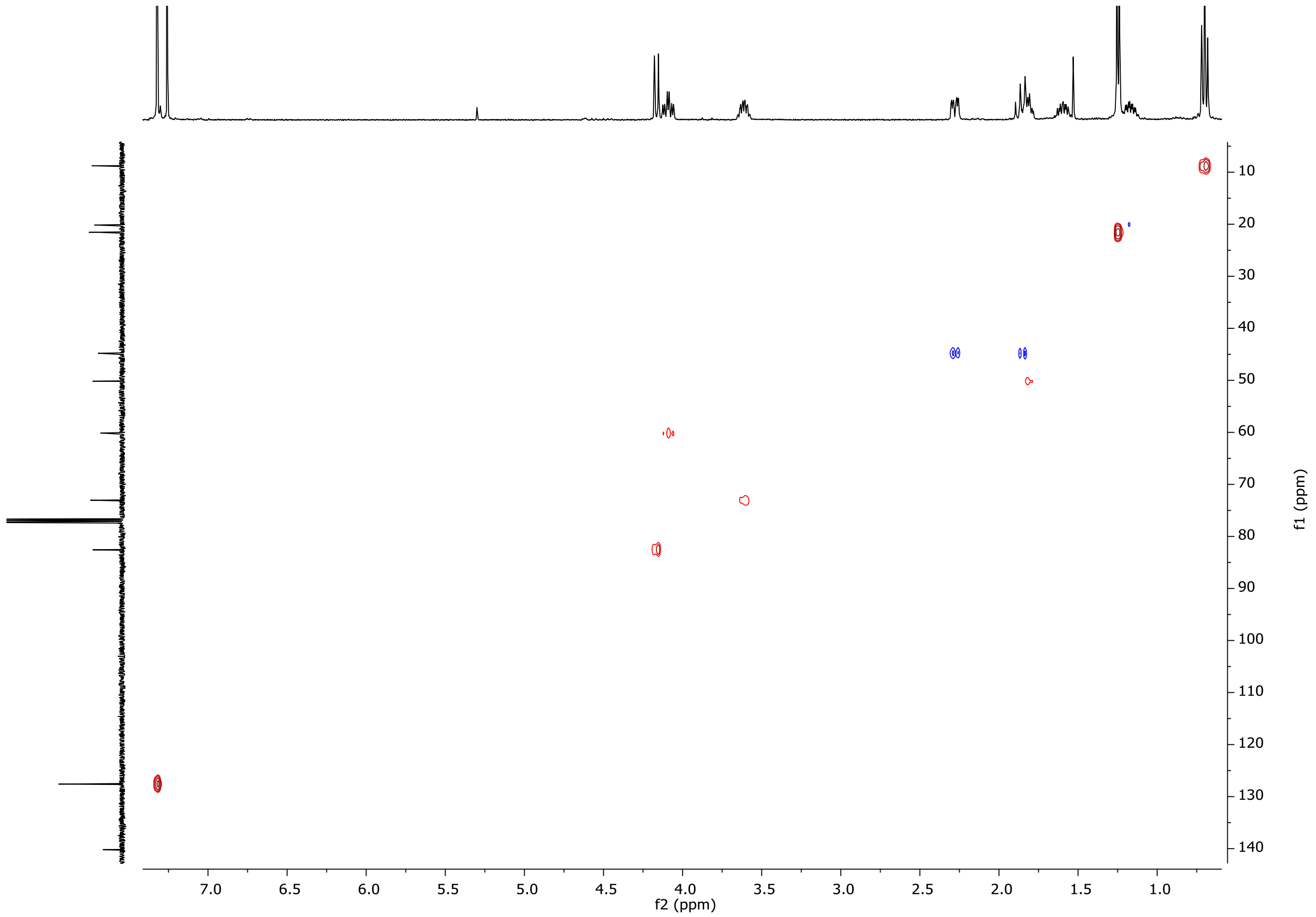

Compound 4x

^{1}H NMR (500 MHz, $CDCl_3$)

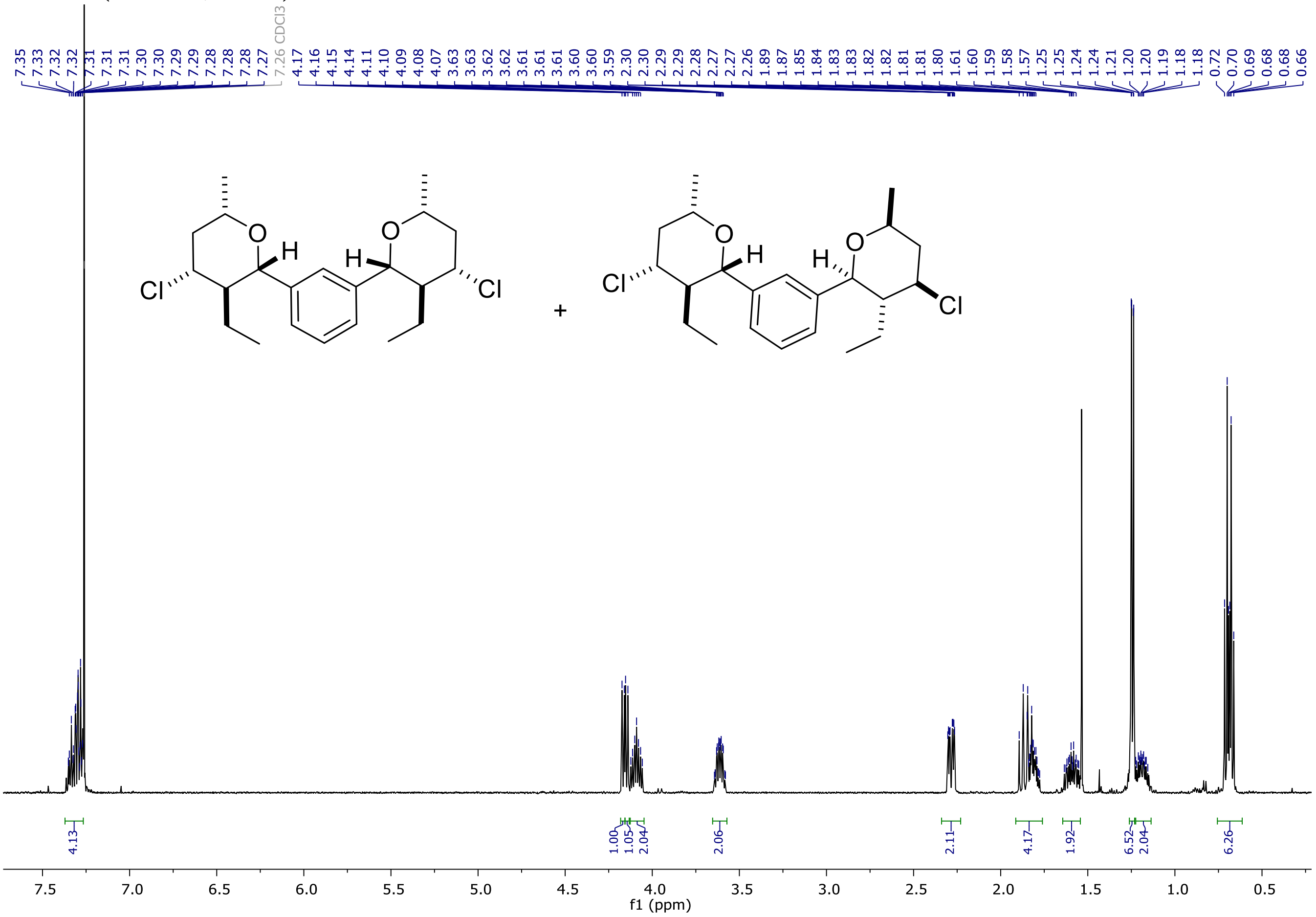

^{13}C NMR (101 MHz, $CDCl_3$)

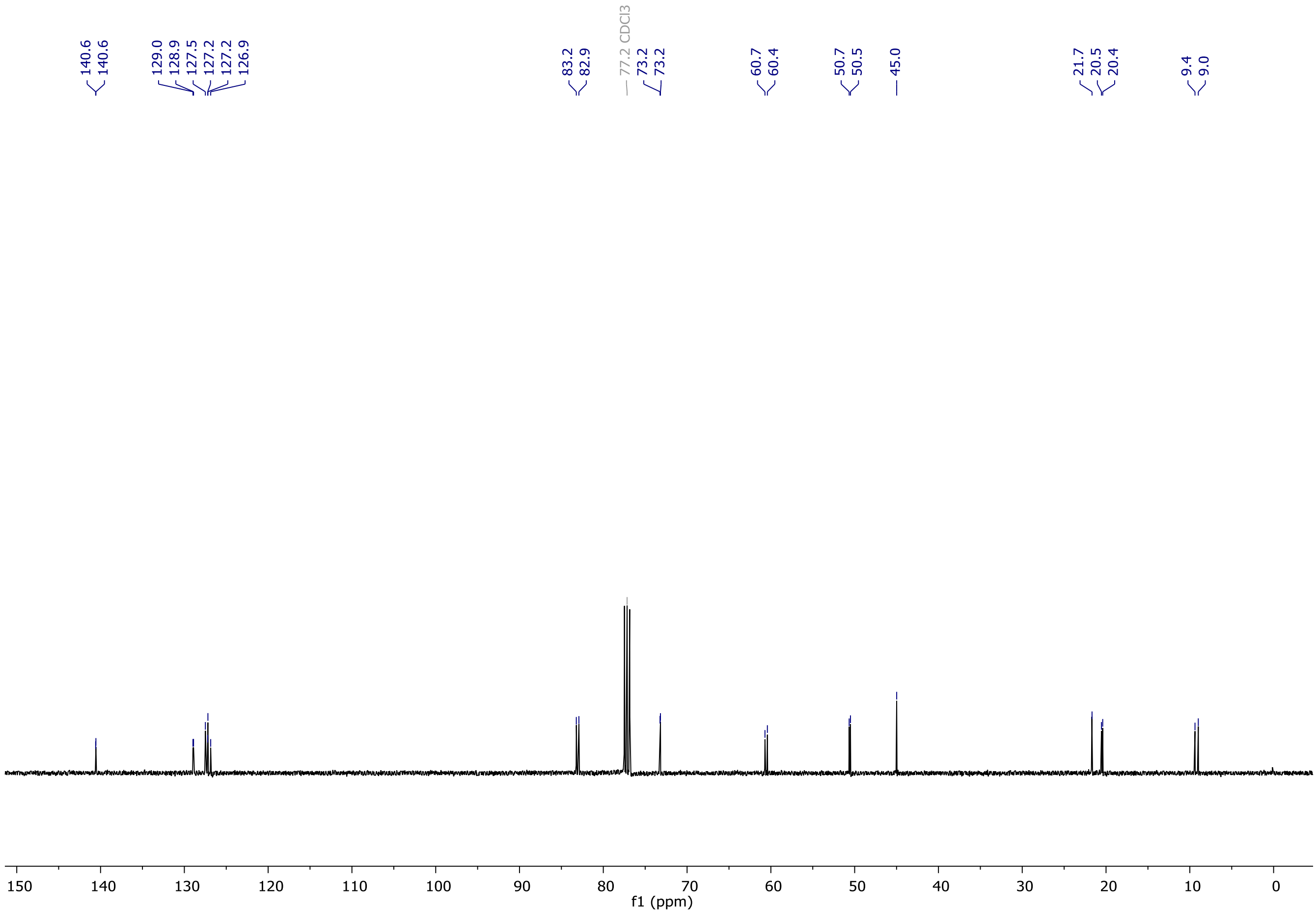

2D-COSY

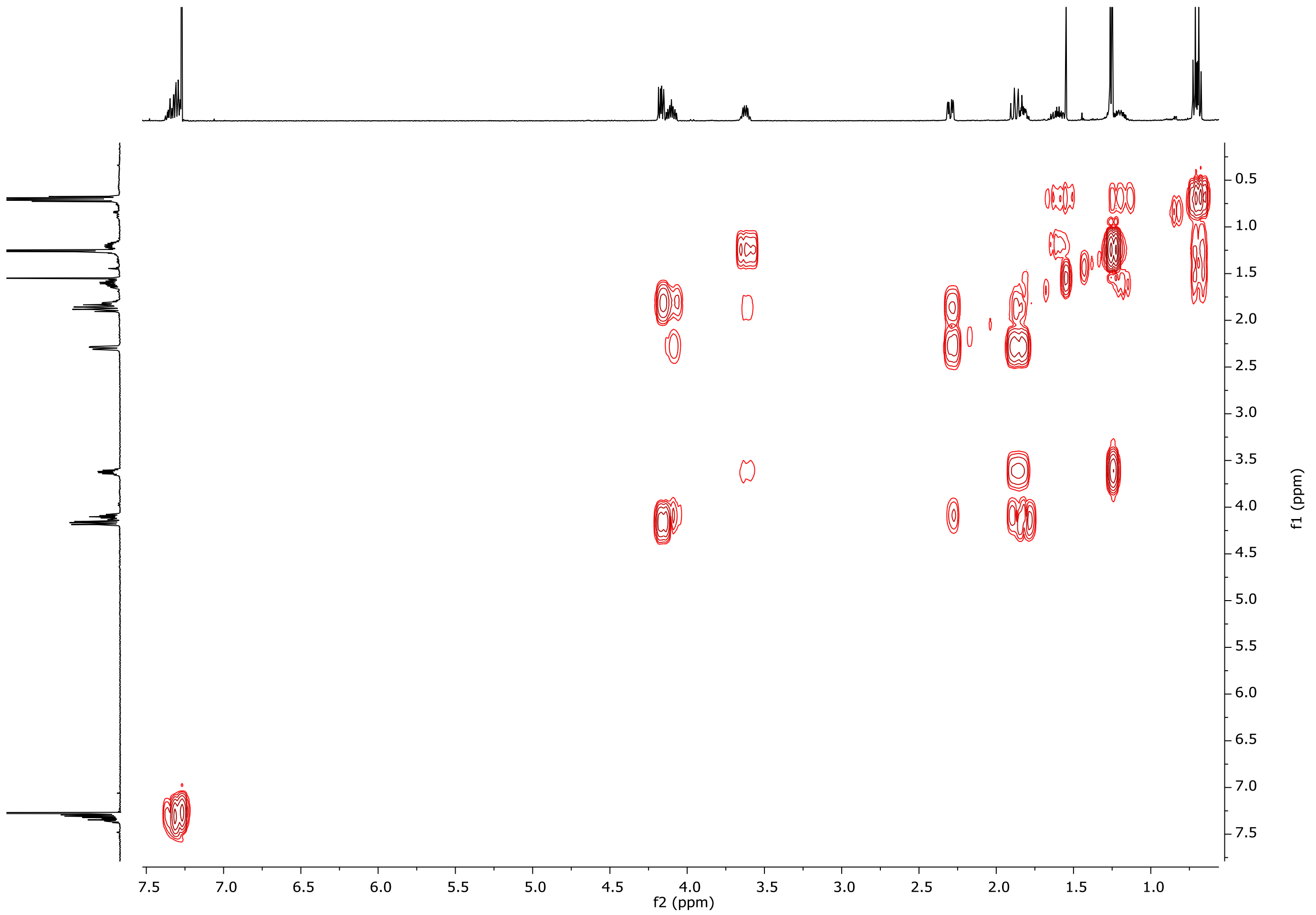

2D-HSQC

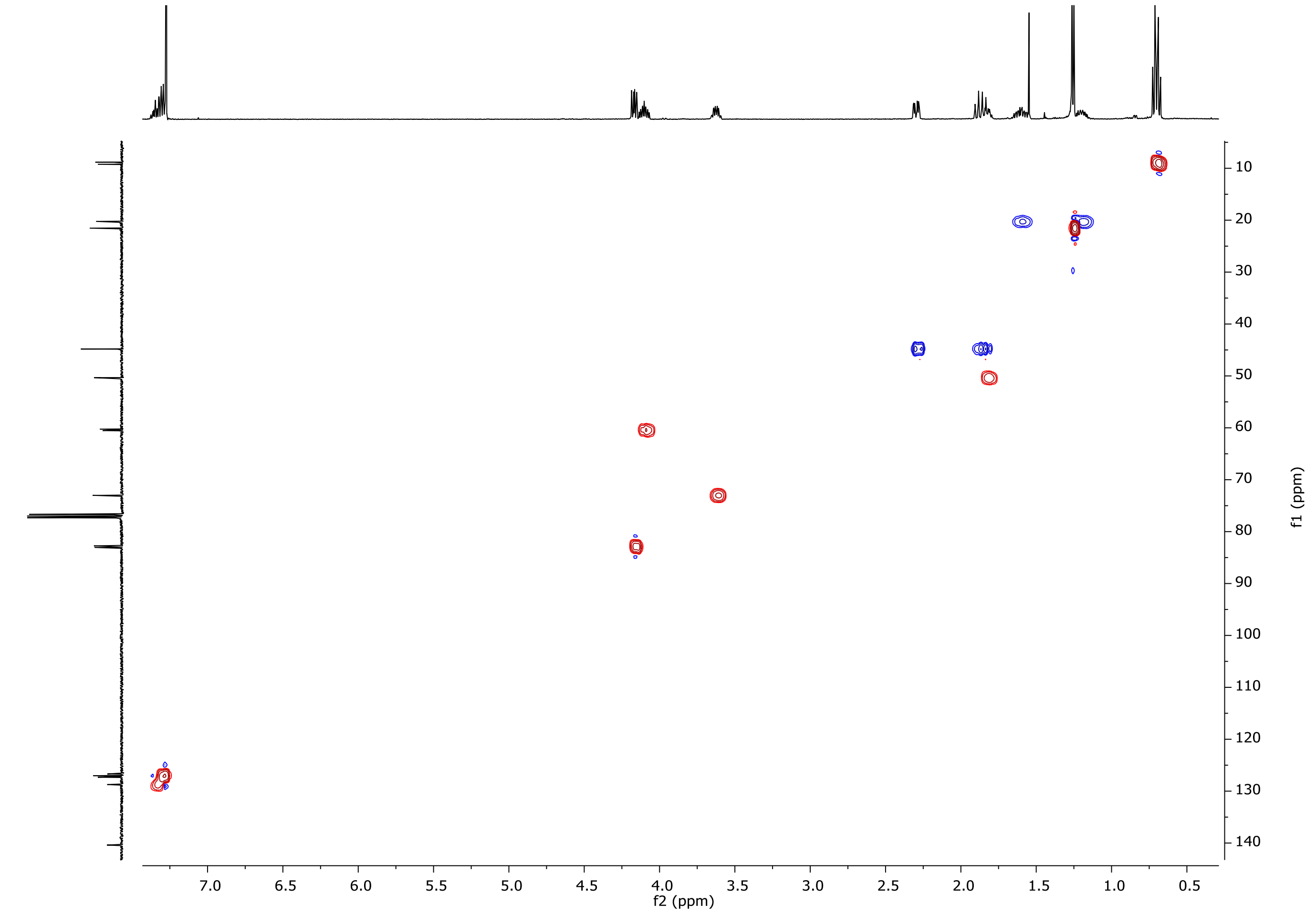

Compound 4y

[1]**H NMR** (500 MHz, $CDCl_3$)

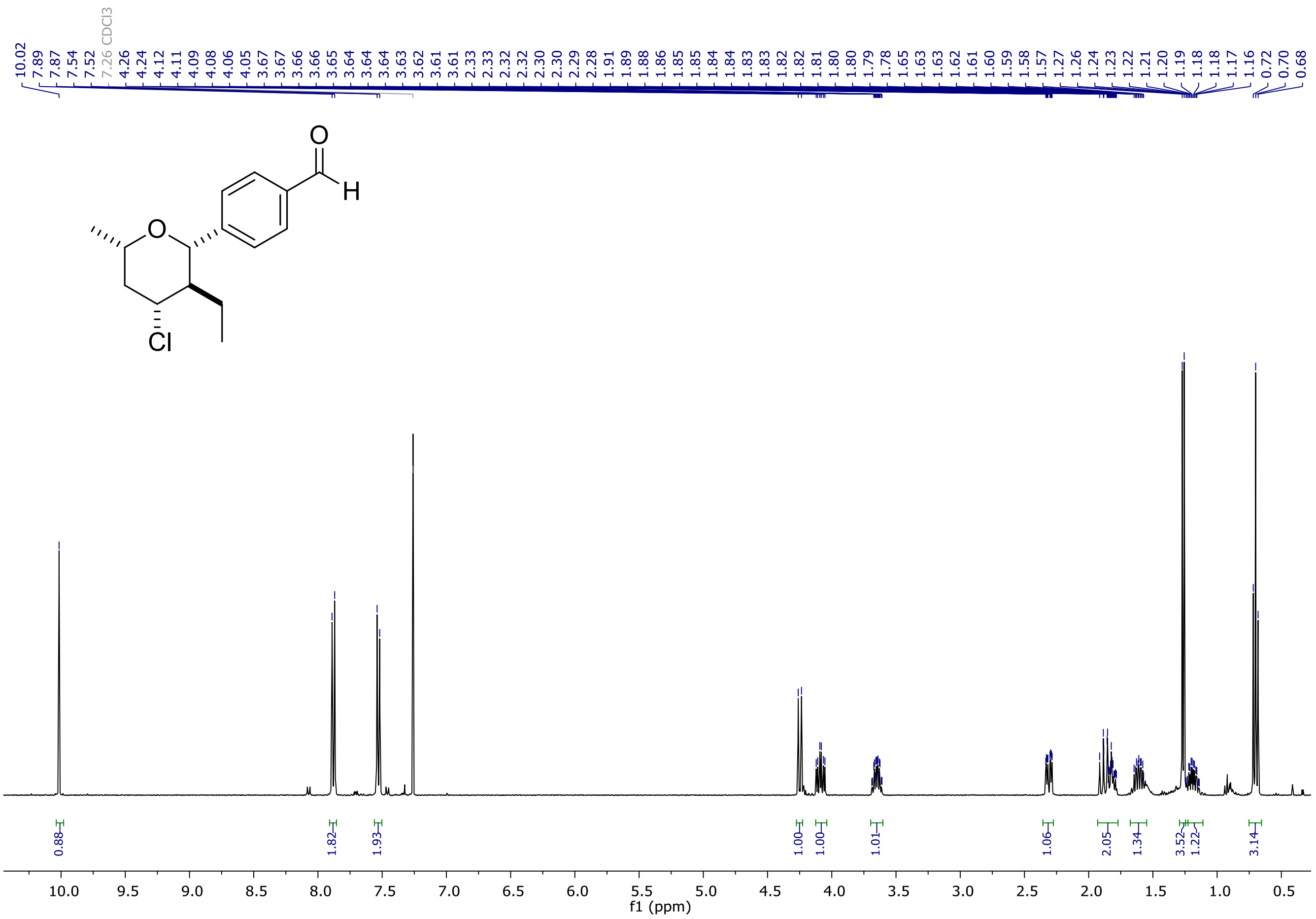

[13]**C NMR** (101 MHz, $CDCl_3$)

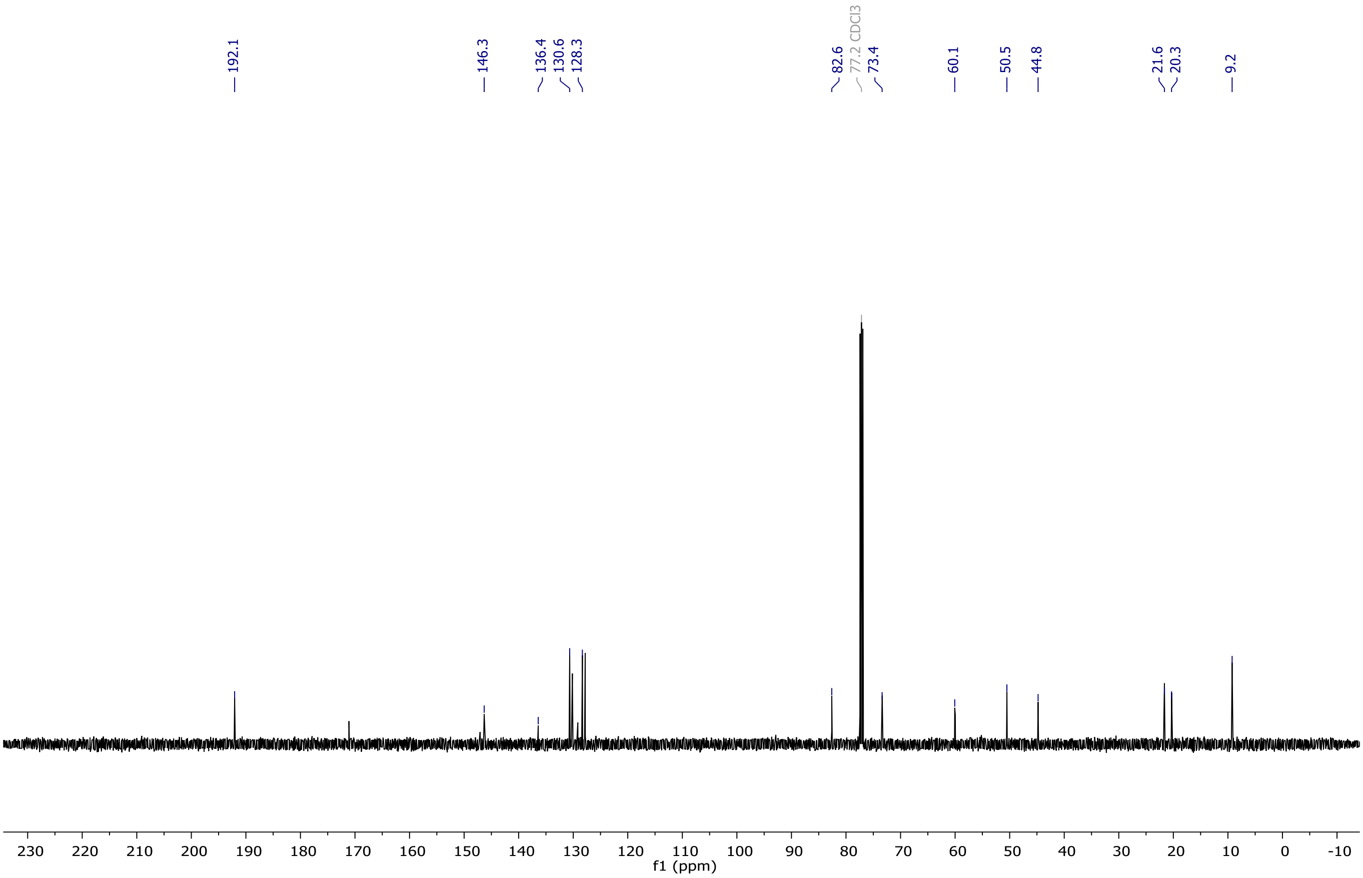

2D-COSY

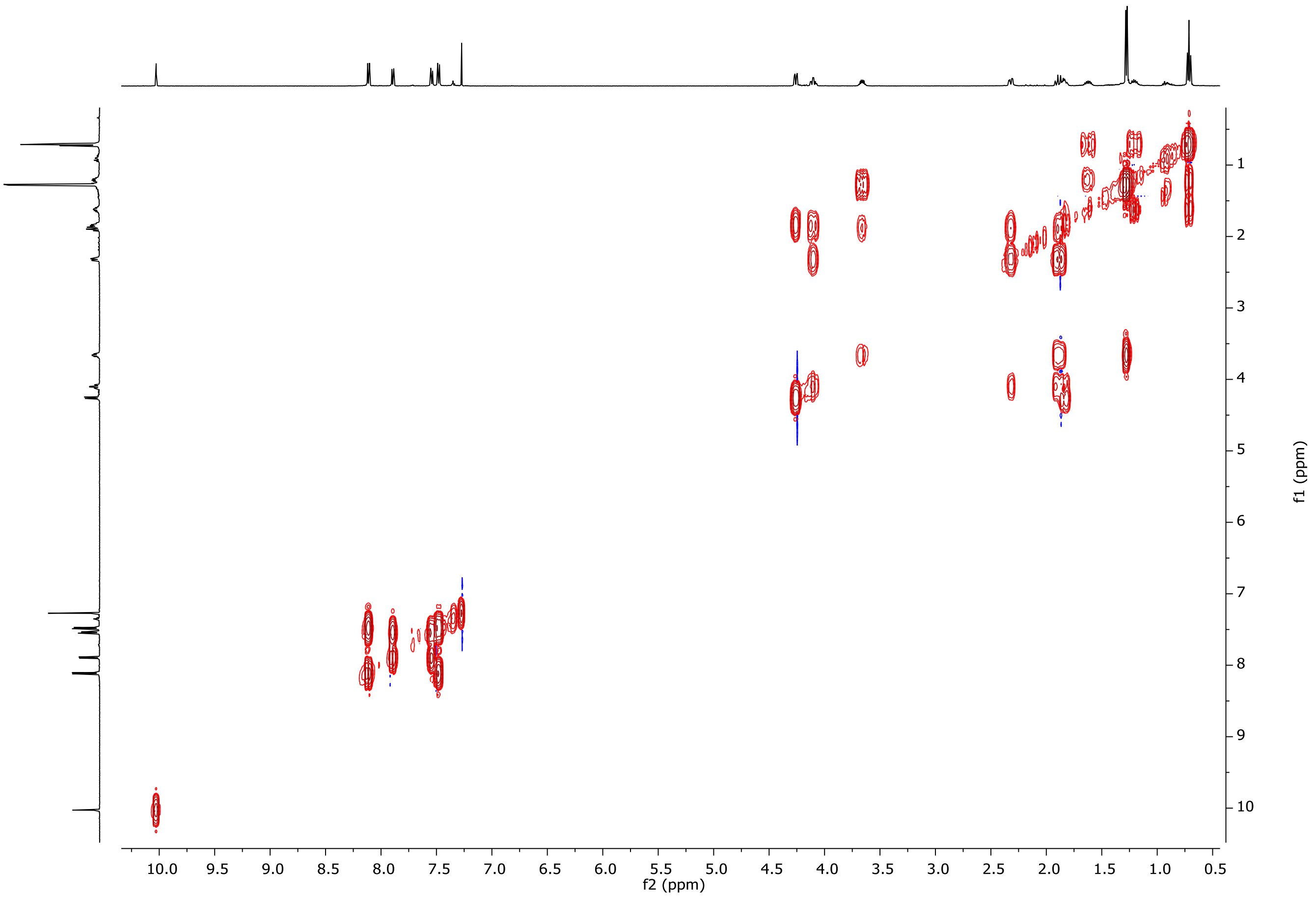

2D-HSQC

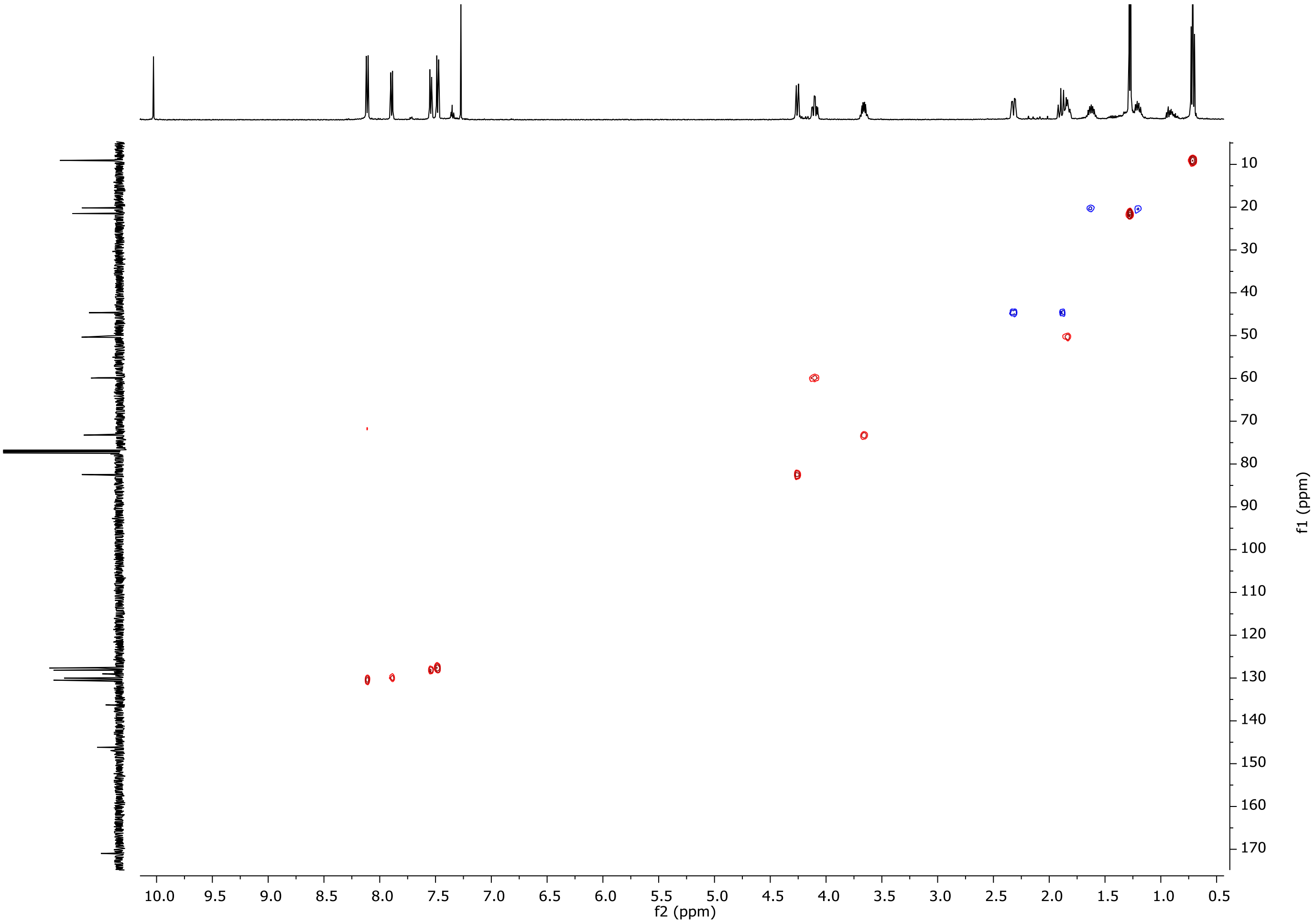

Compound 4z

^{1}H NMR (500 MHz, $CDCl_3$)

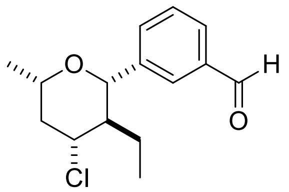

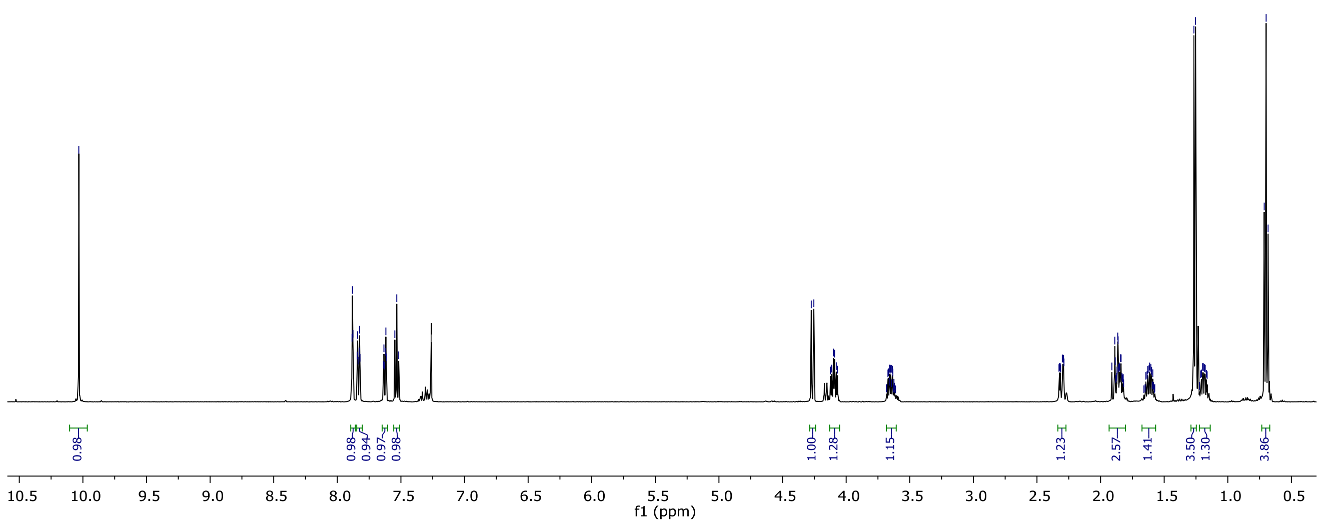

^{13}C NMR (101 MHz, $CDCl_3$)

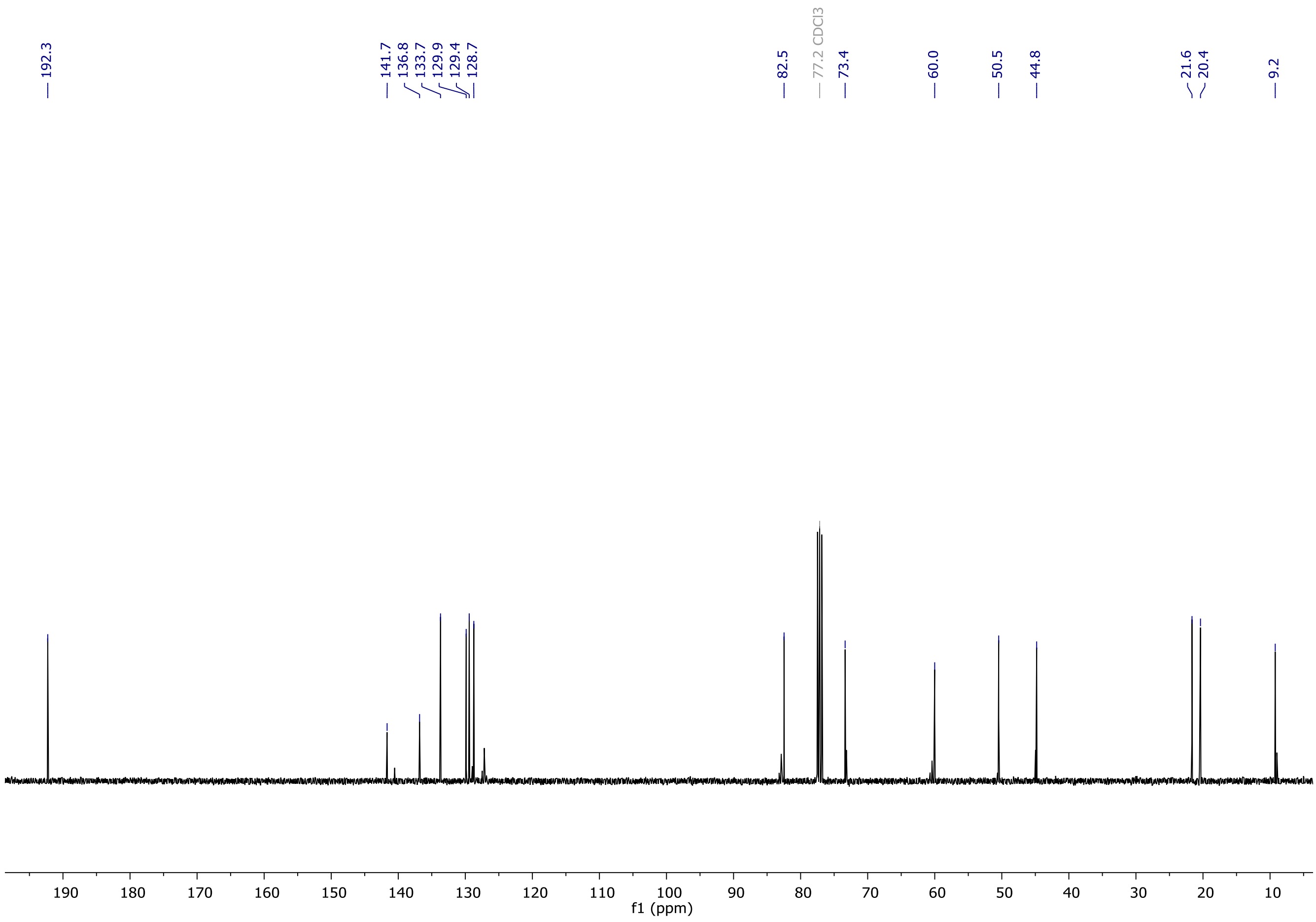

2D-COSY

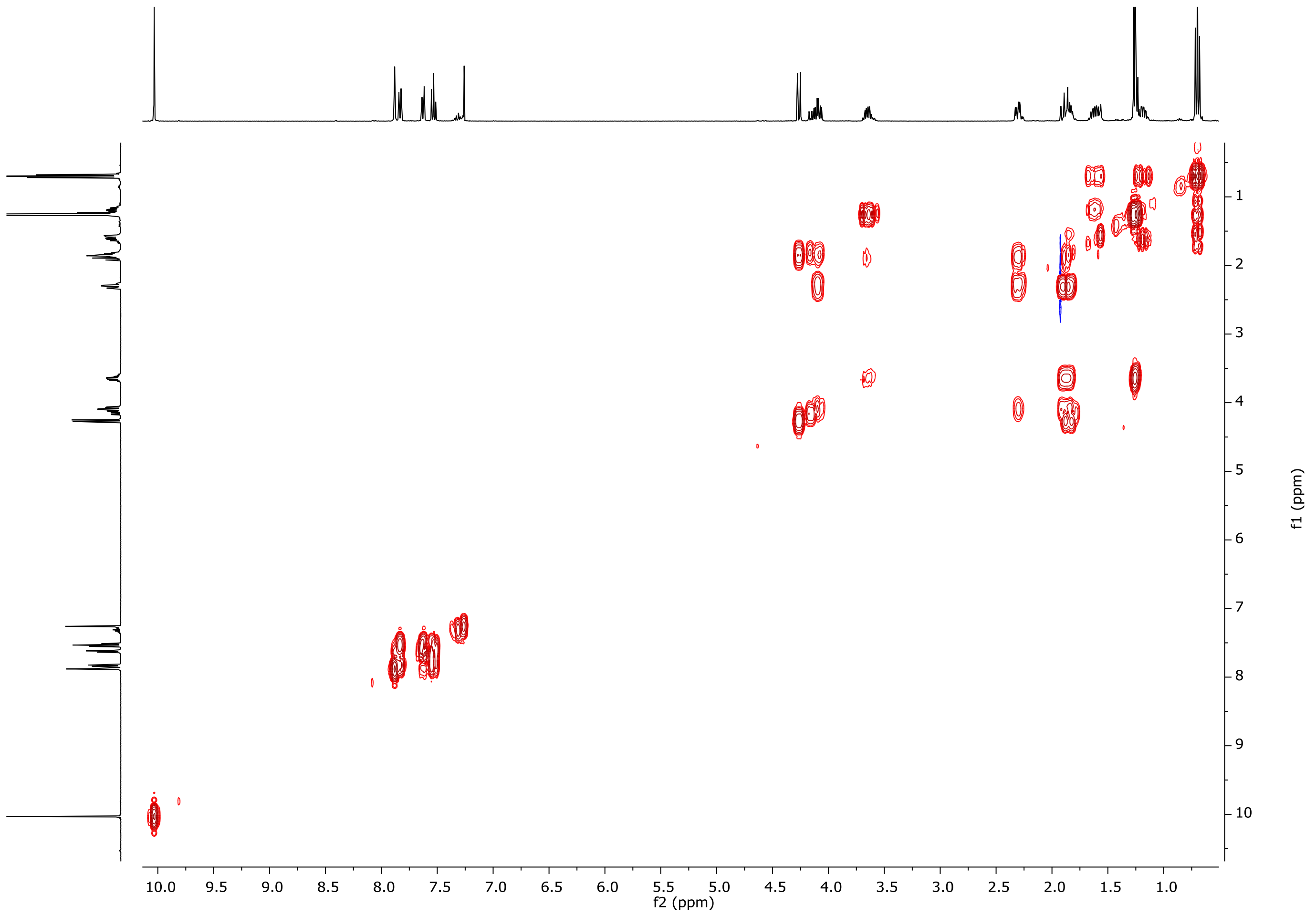

2D-HSQC

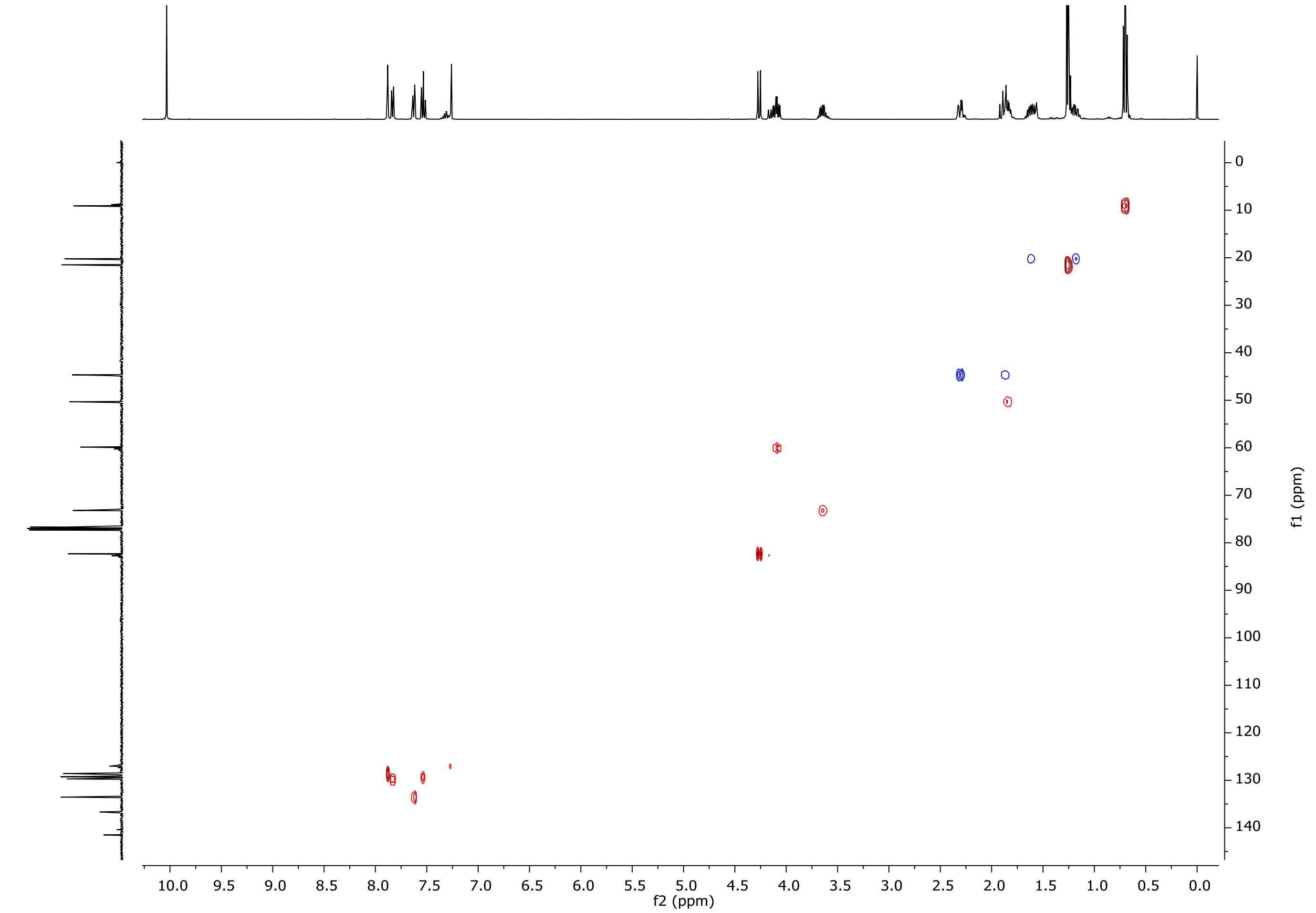

Compound 4aa

^{1}H NMR (500 MHz, $CDCl_3$)

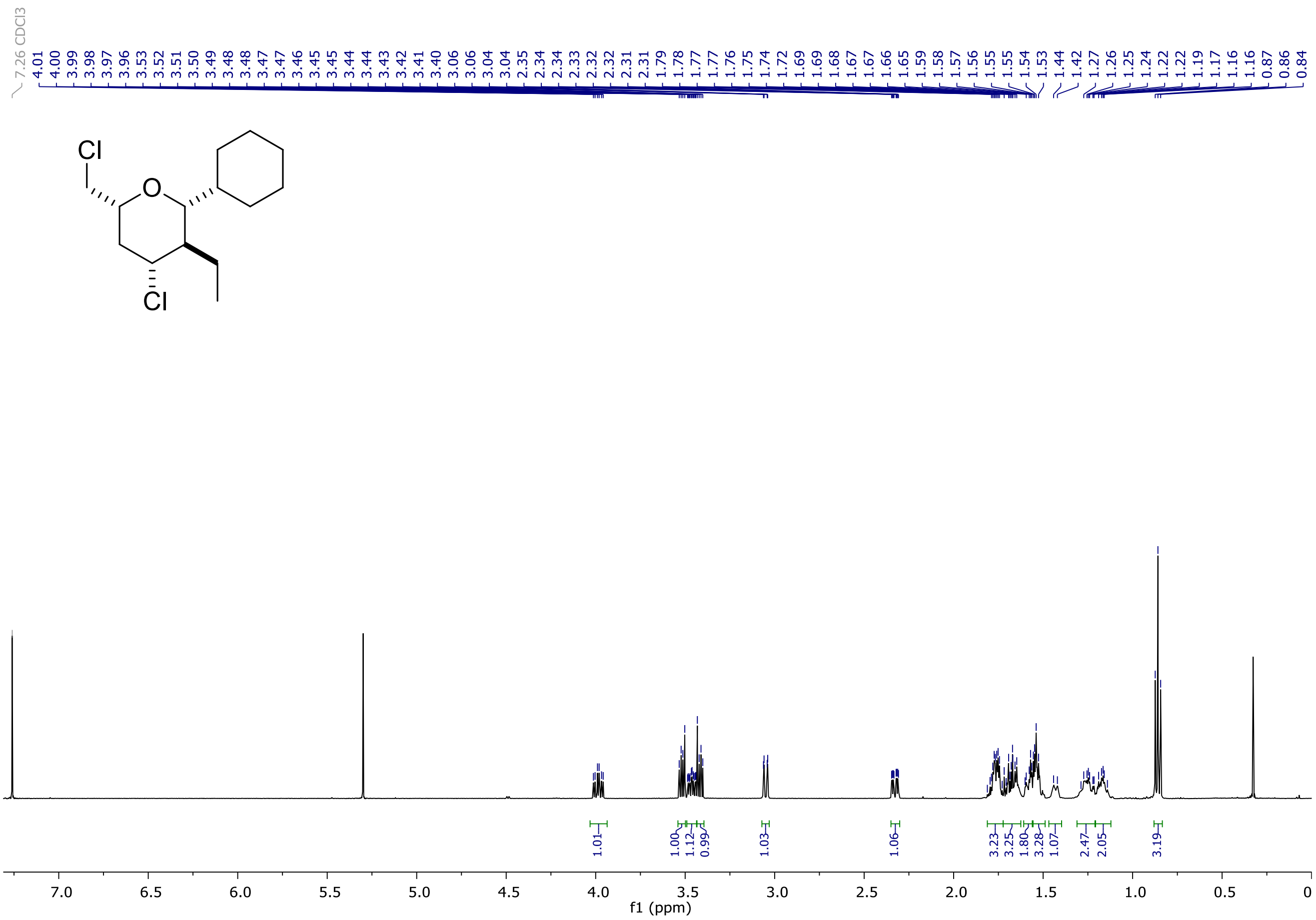

^{13}C NMR (101 MHz, $CDCl_3$)

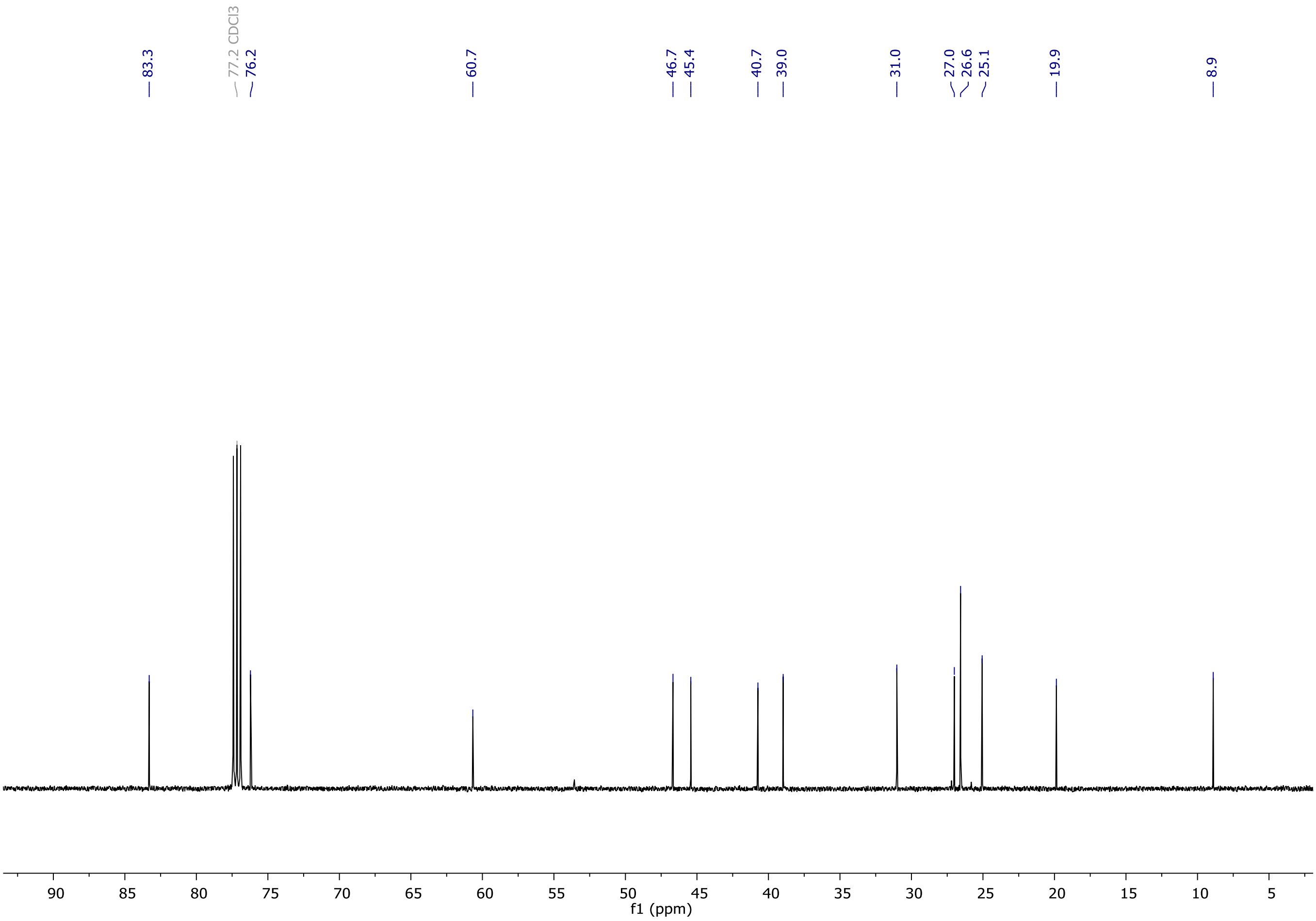

2D-COSY

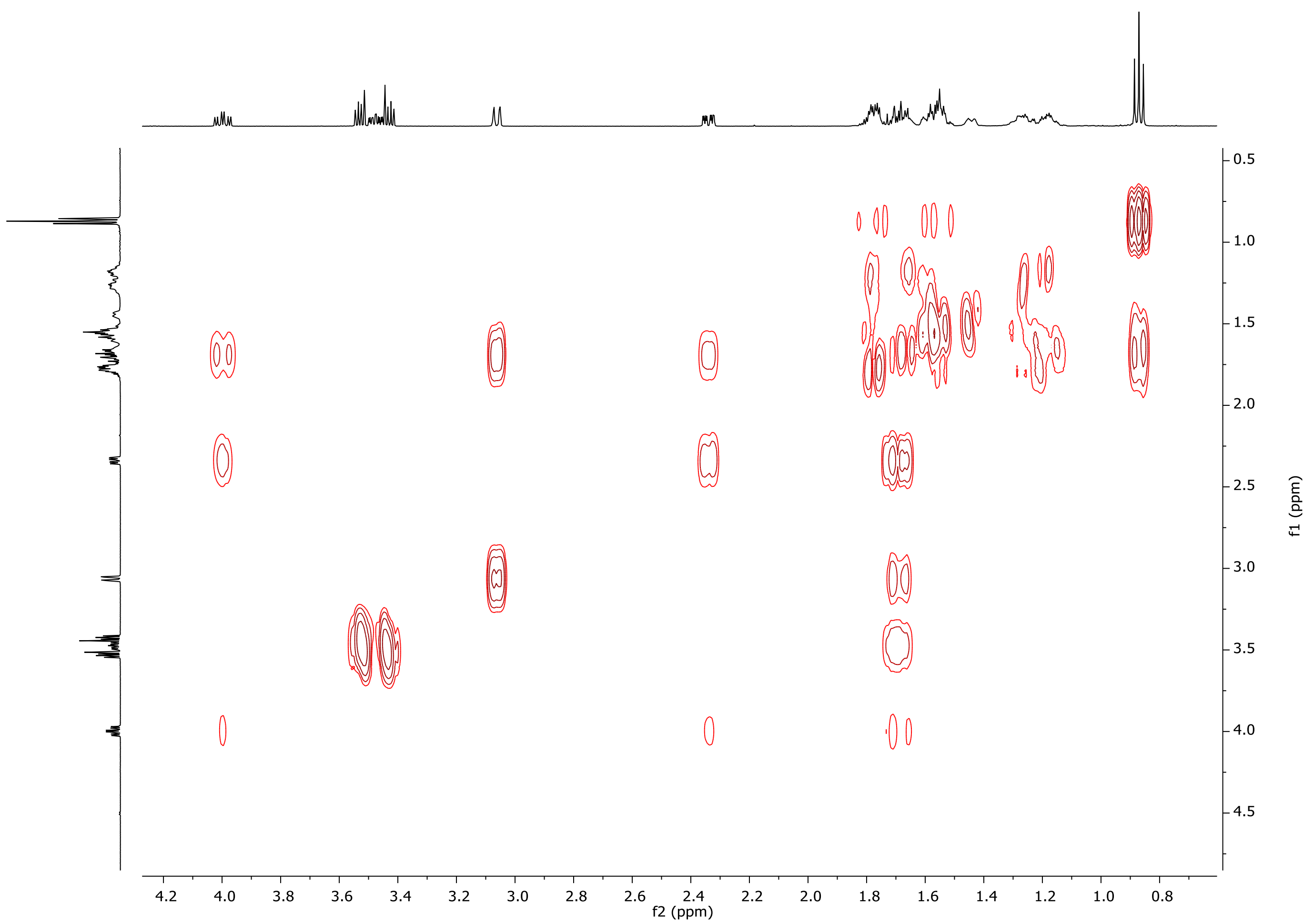

2D-HSQC

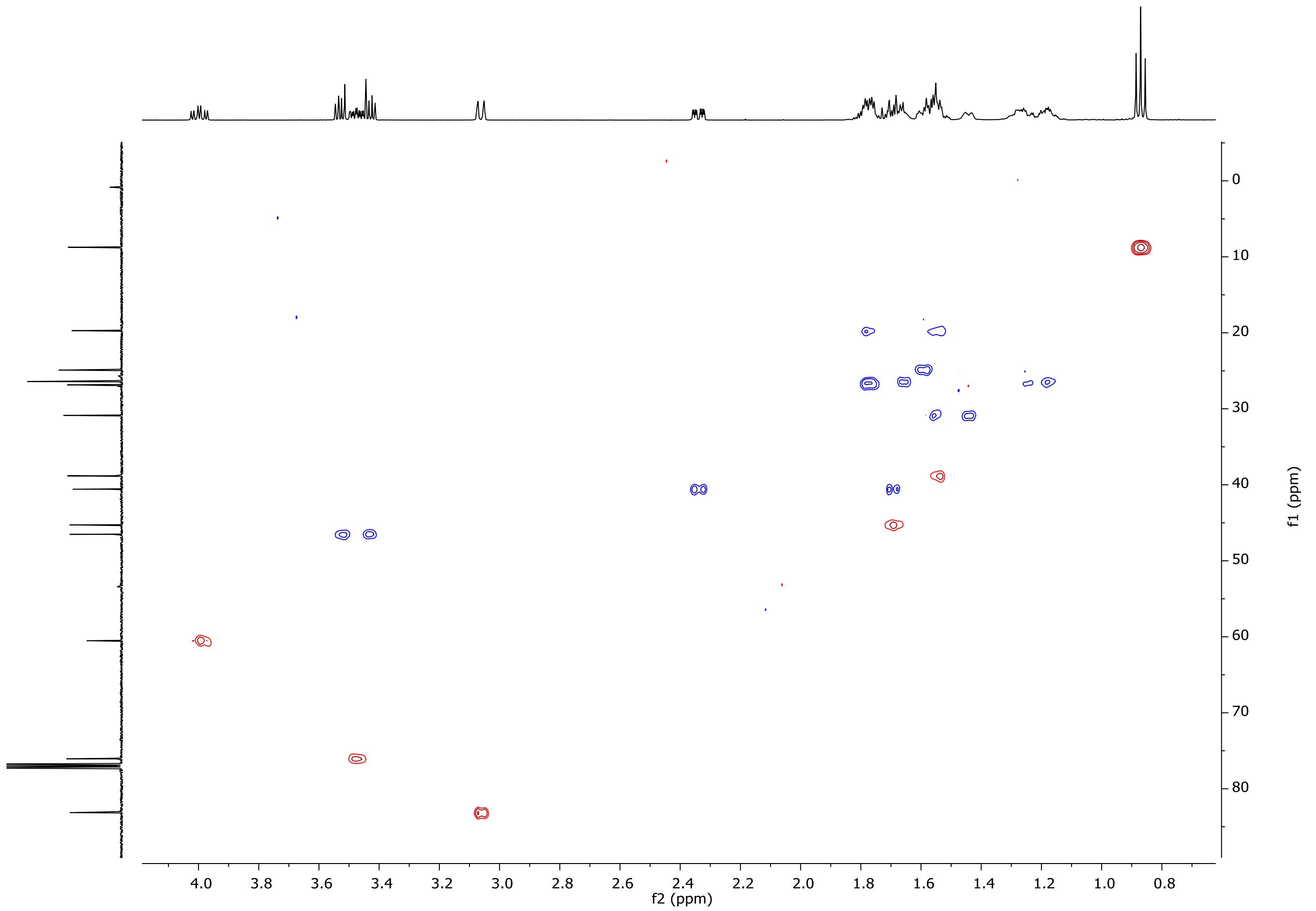

Compound 4ab

^{1}H NMR (500 MHz, $CDCl_3$)

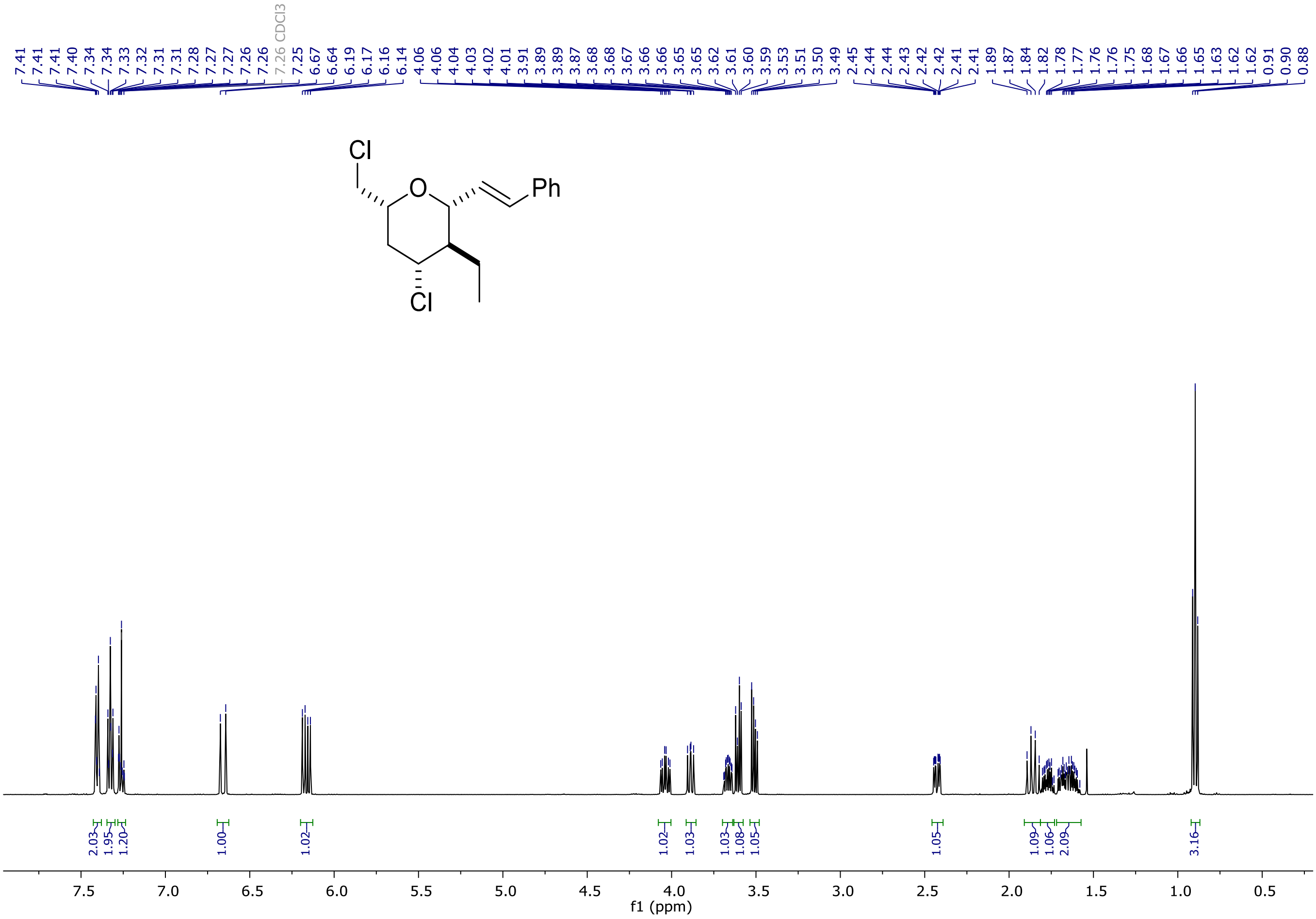

^{13}C NMR (101 MHz, $CDCl_3$)

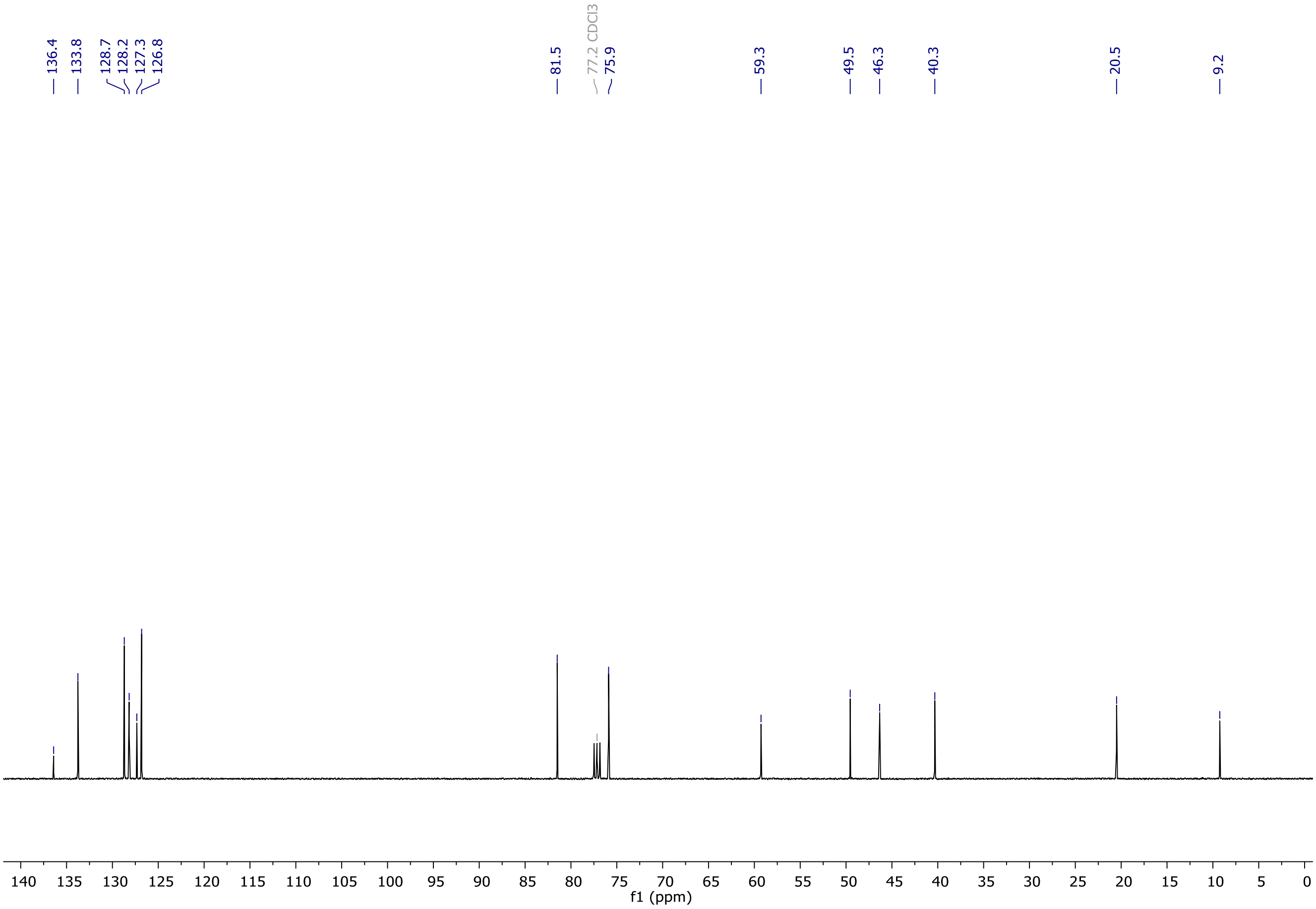

2D-COSY

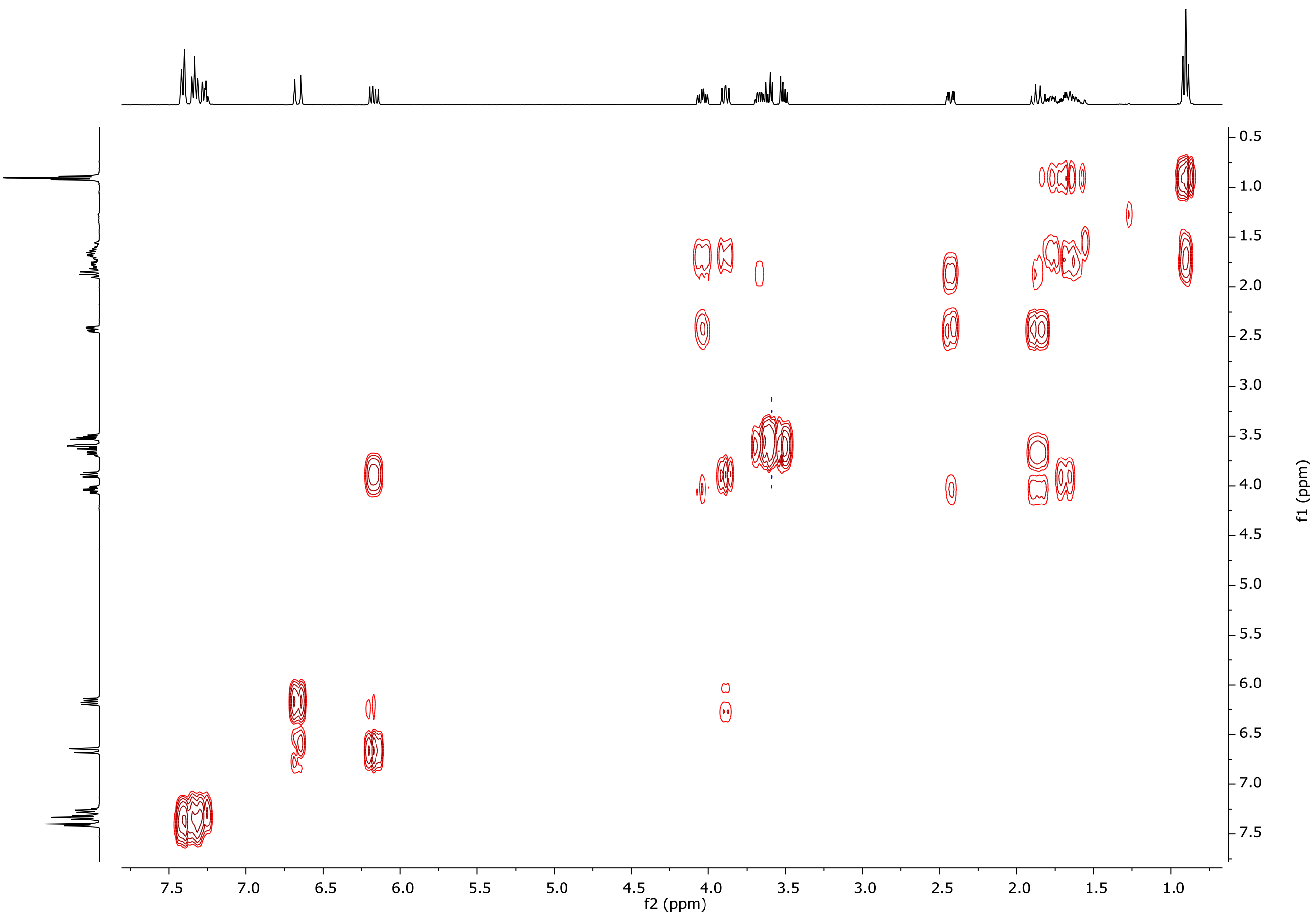

2D-HSQC

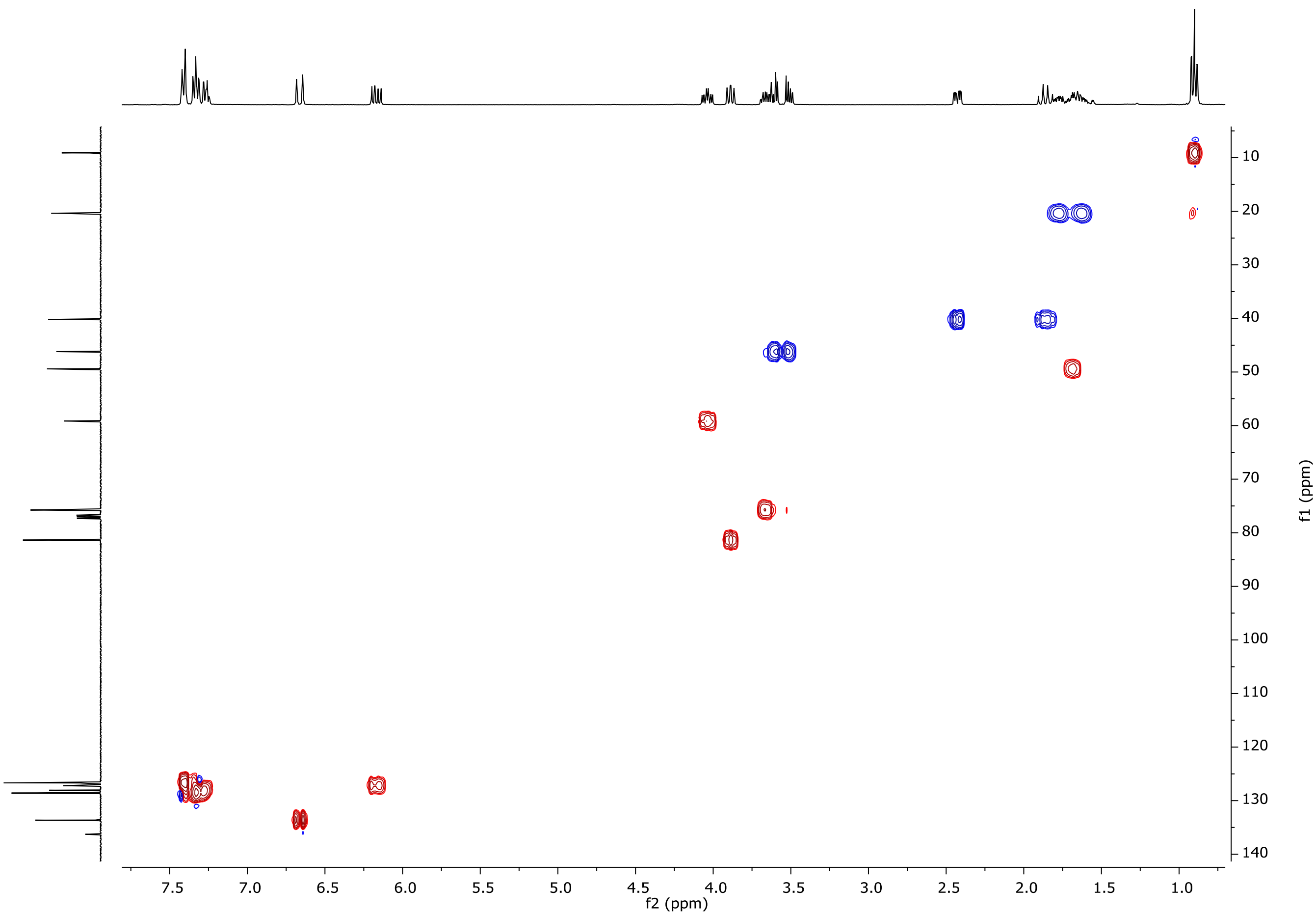

Compound 4ac

^{1}H NMR (500 MHz, $CDCl_3$)

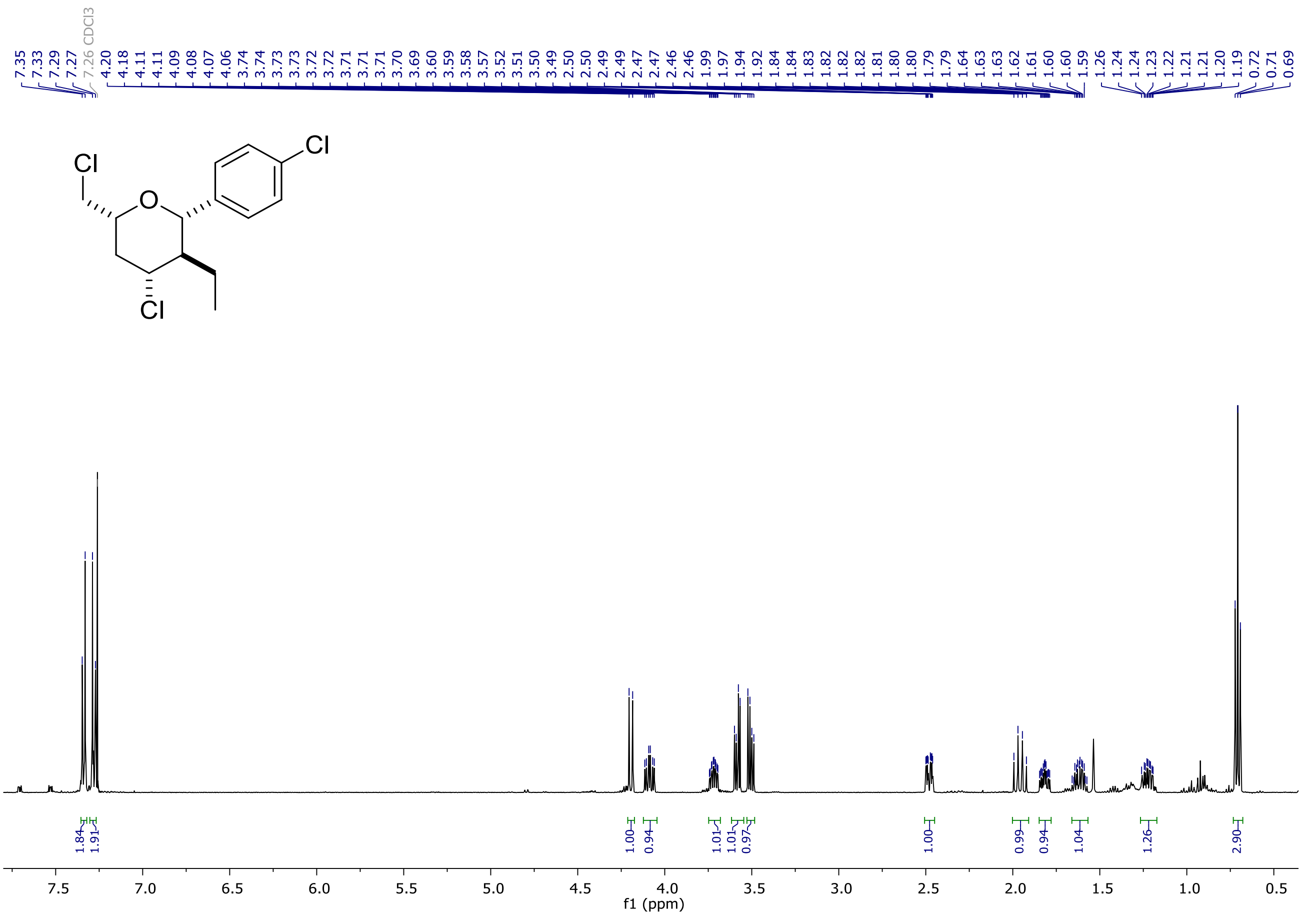

^{13}C NMR (101 MHz, $CDCl_3$)

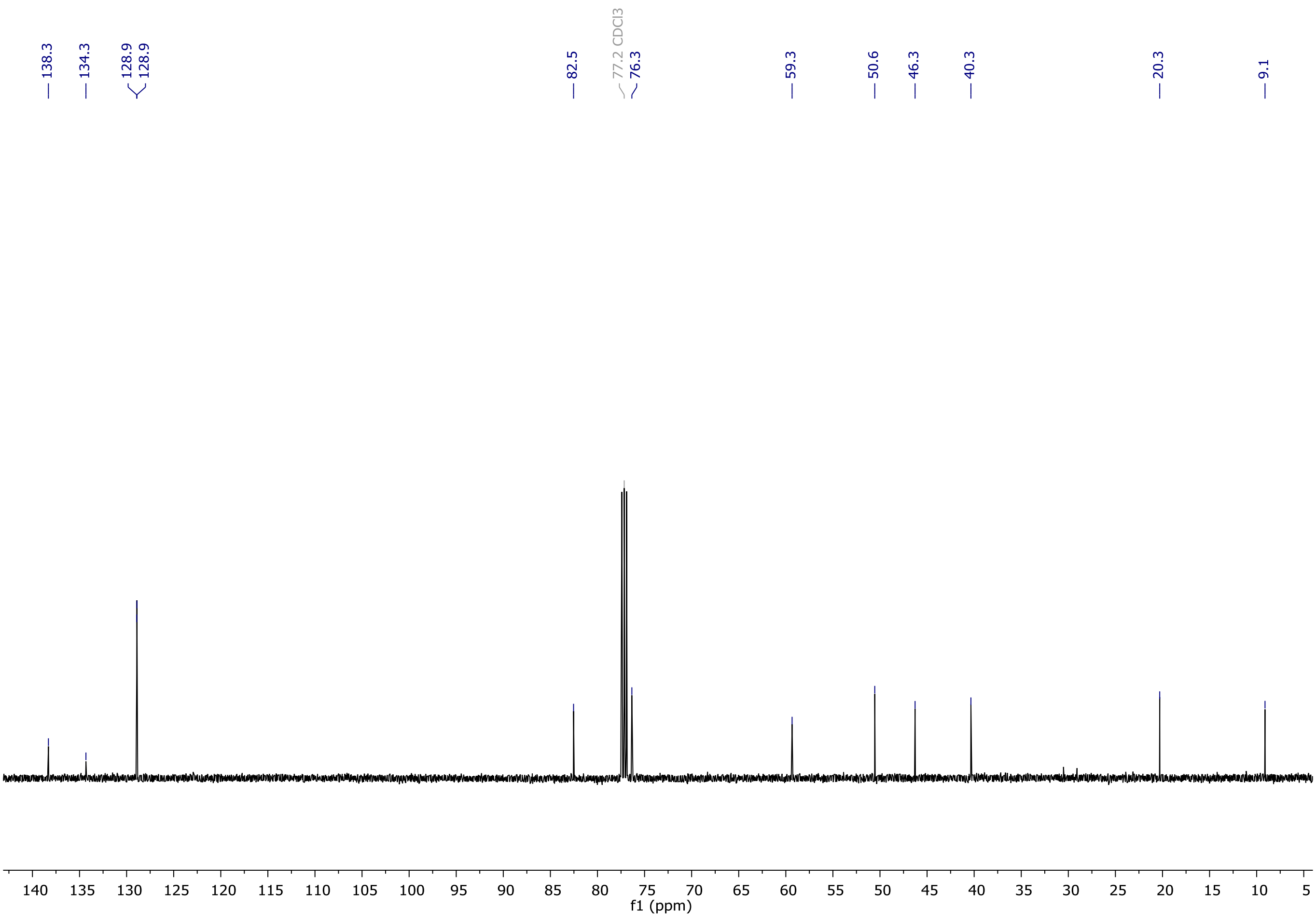

2D-COSY

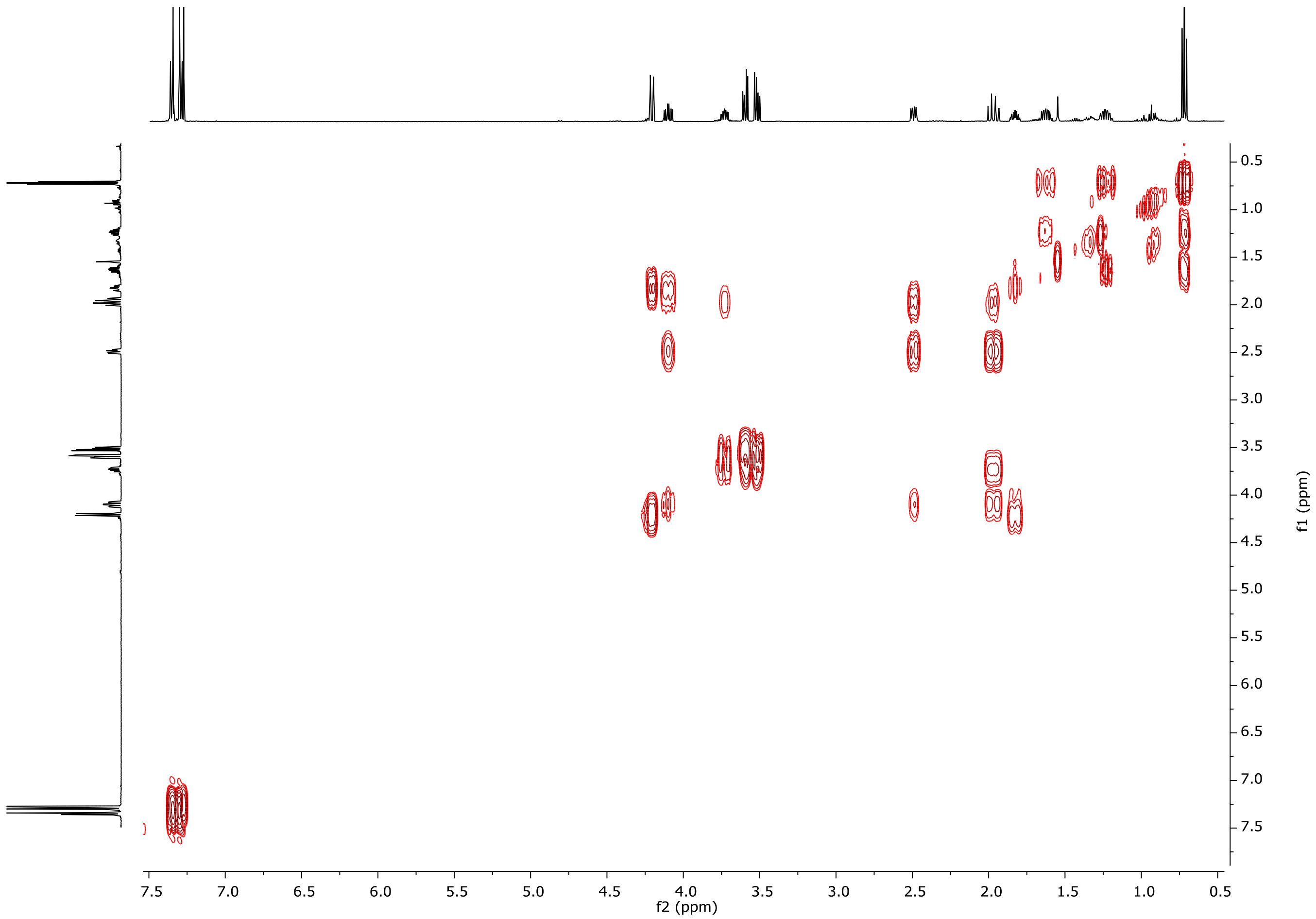

2D-HSQC

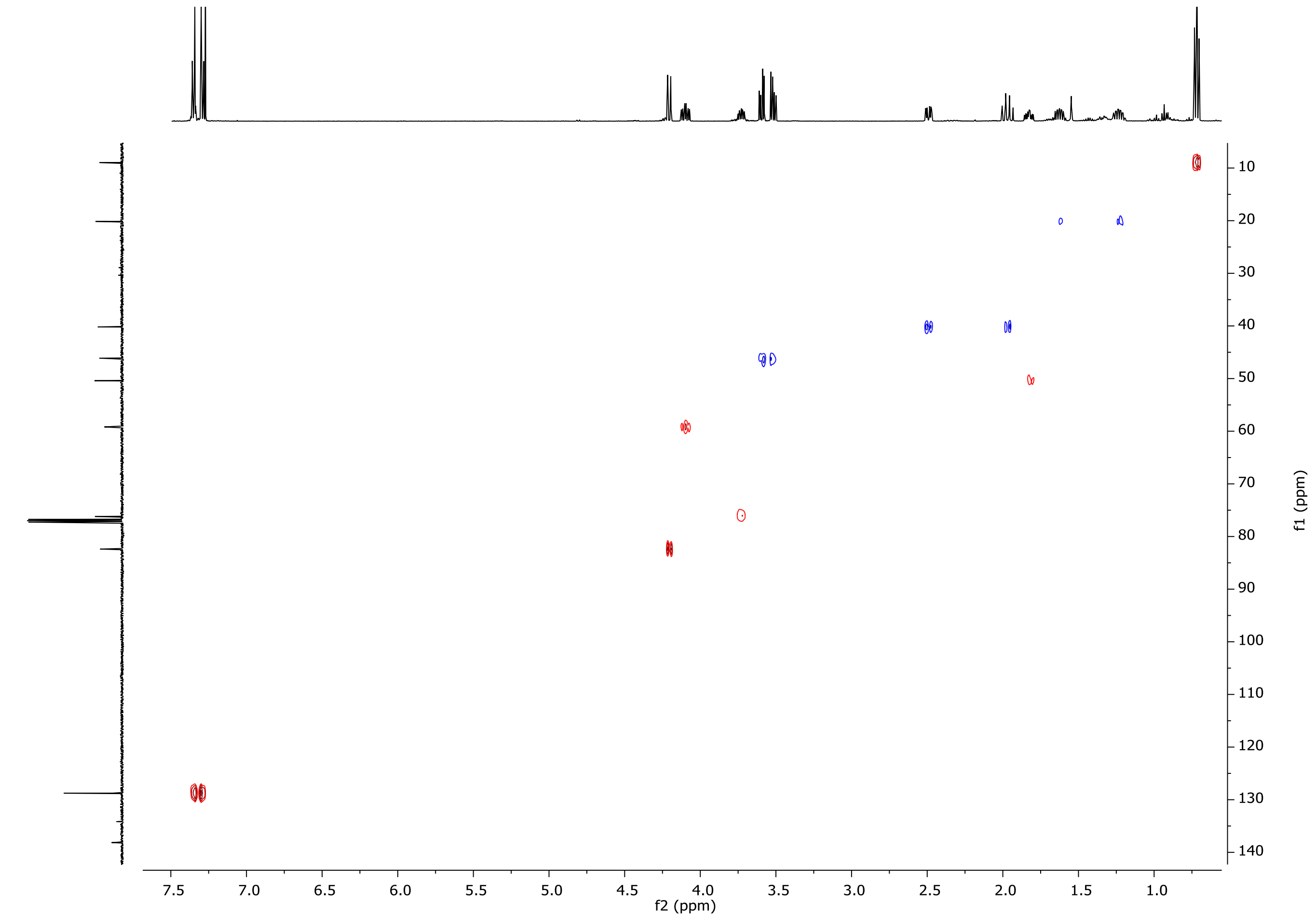

Compound 4ad

^{1}H NMR (500 MHz, $CDCl_3$)

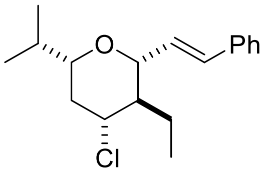

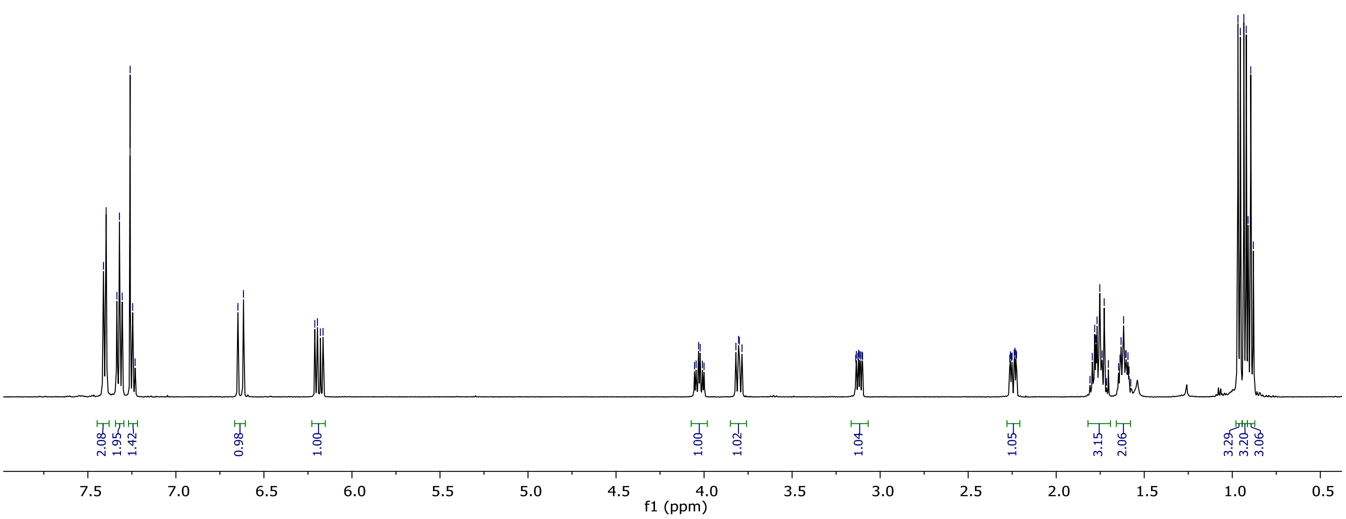

^{13}C NMR (101 MHz, $CDCl_3$)

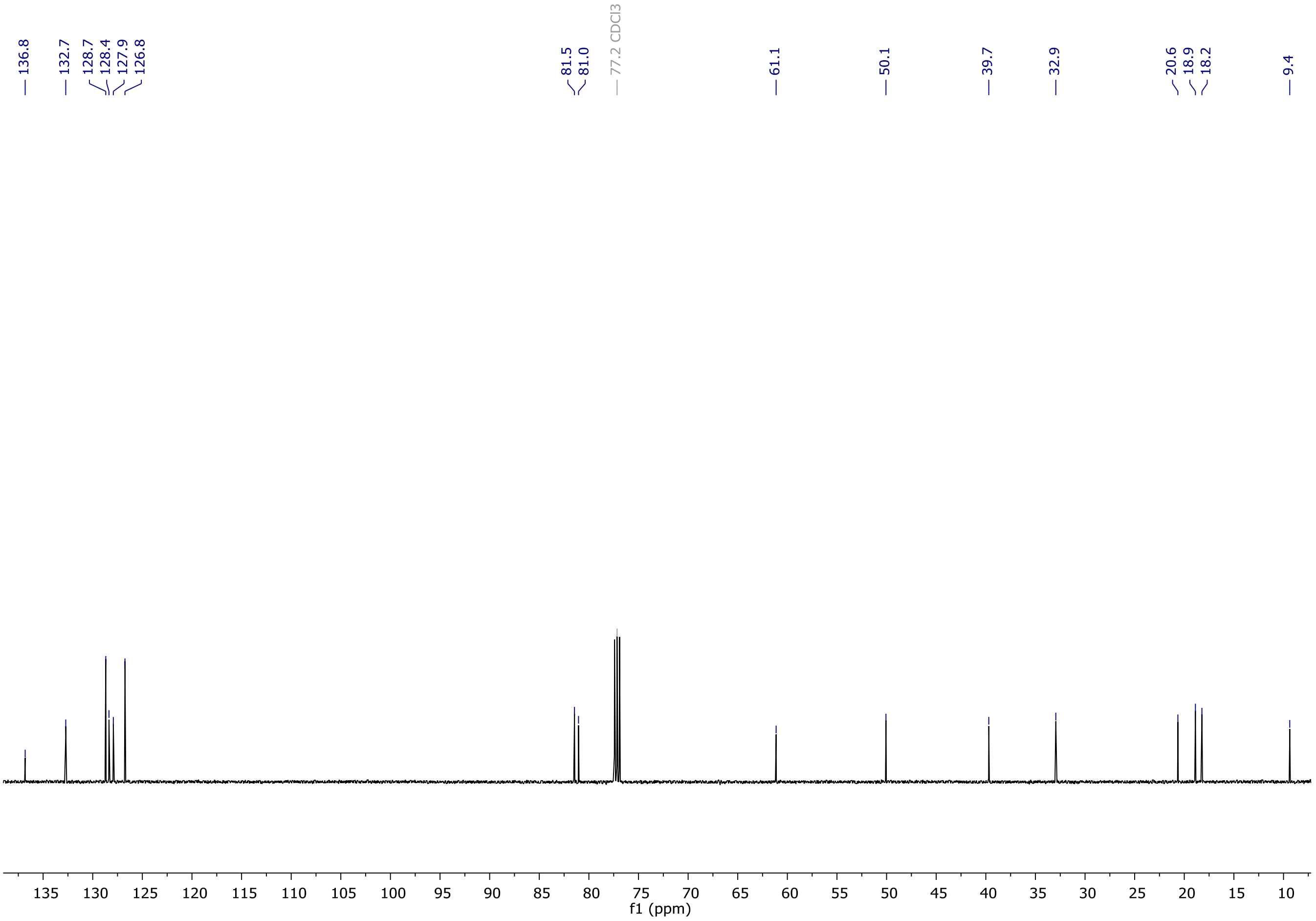

2D-COSY

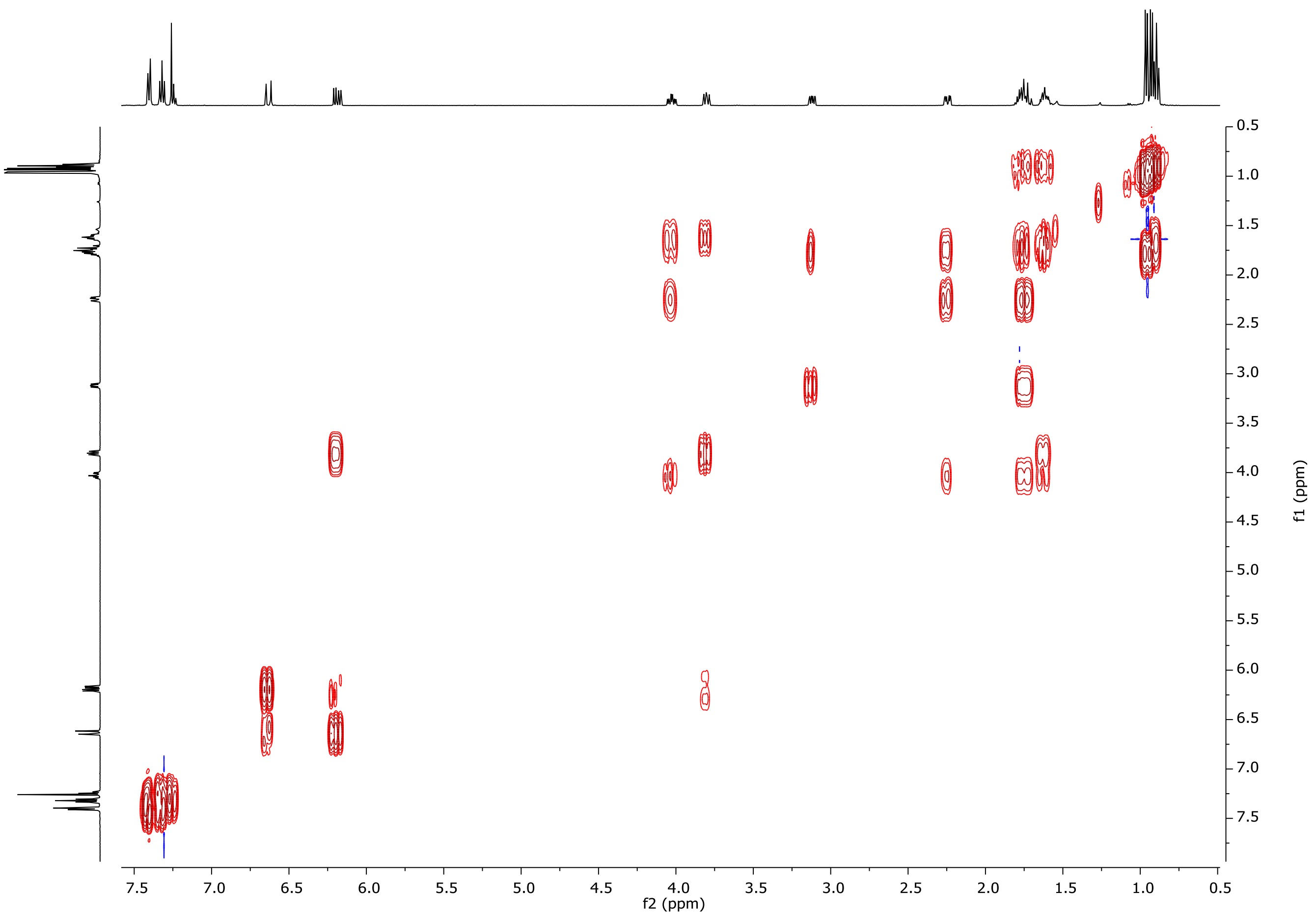

2D-HSQC

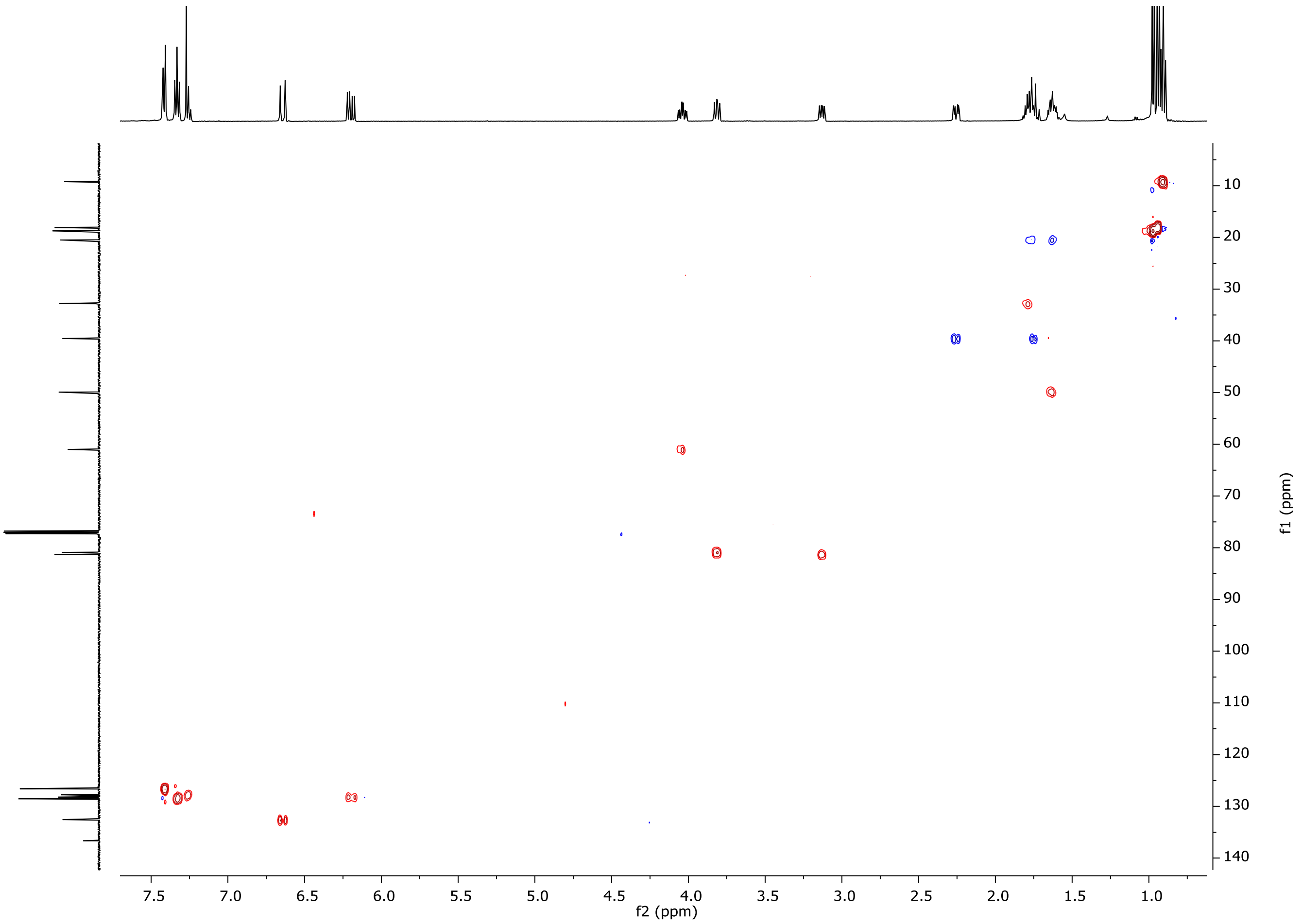

Compound 4ae

^{1}H NMR (500 MHz, $CDCl_3$)

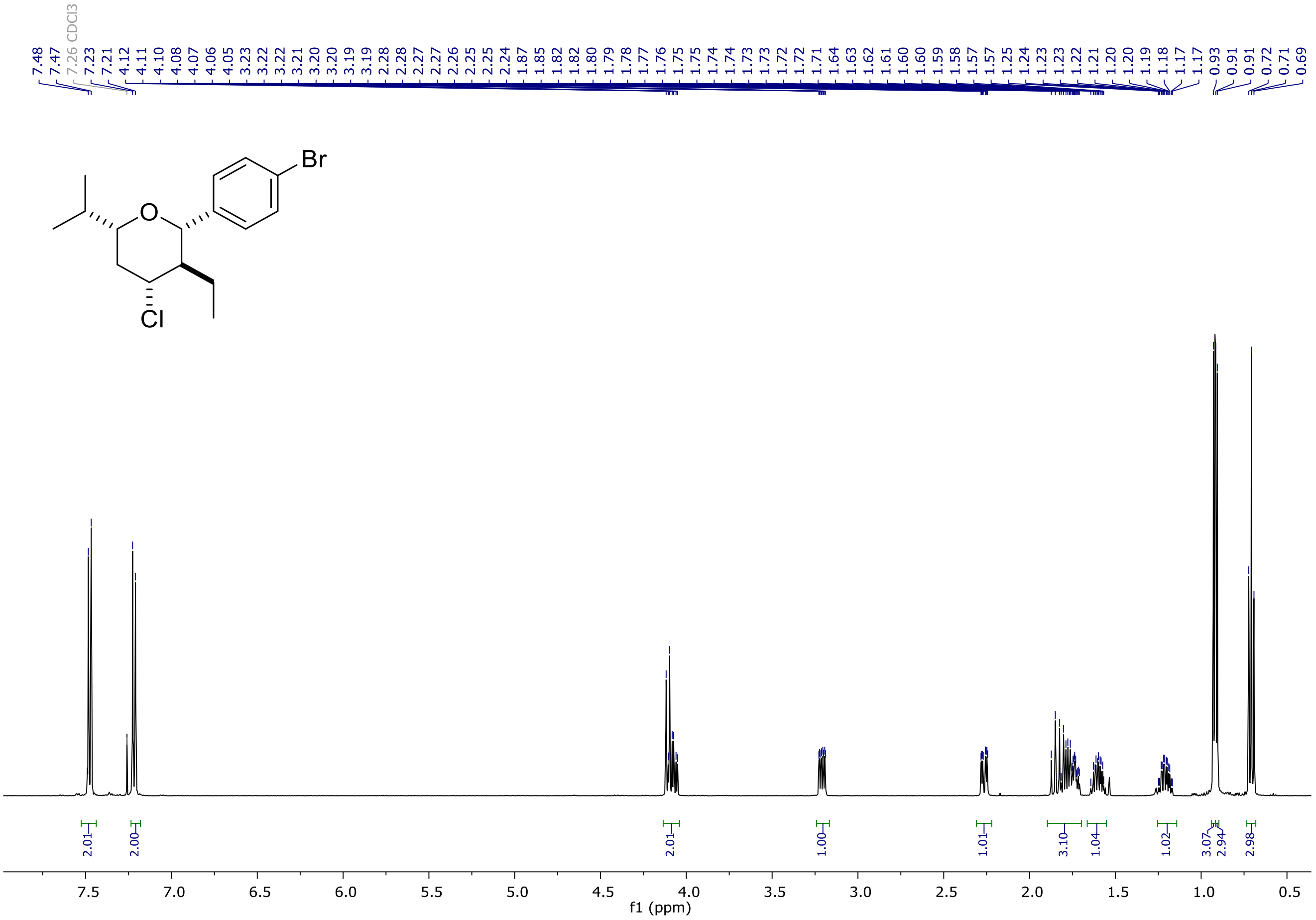

^{13}C NMR (101 MHz, $CDCl_3$)

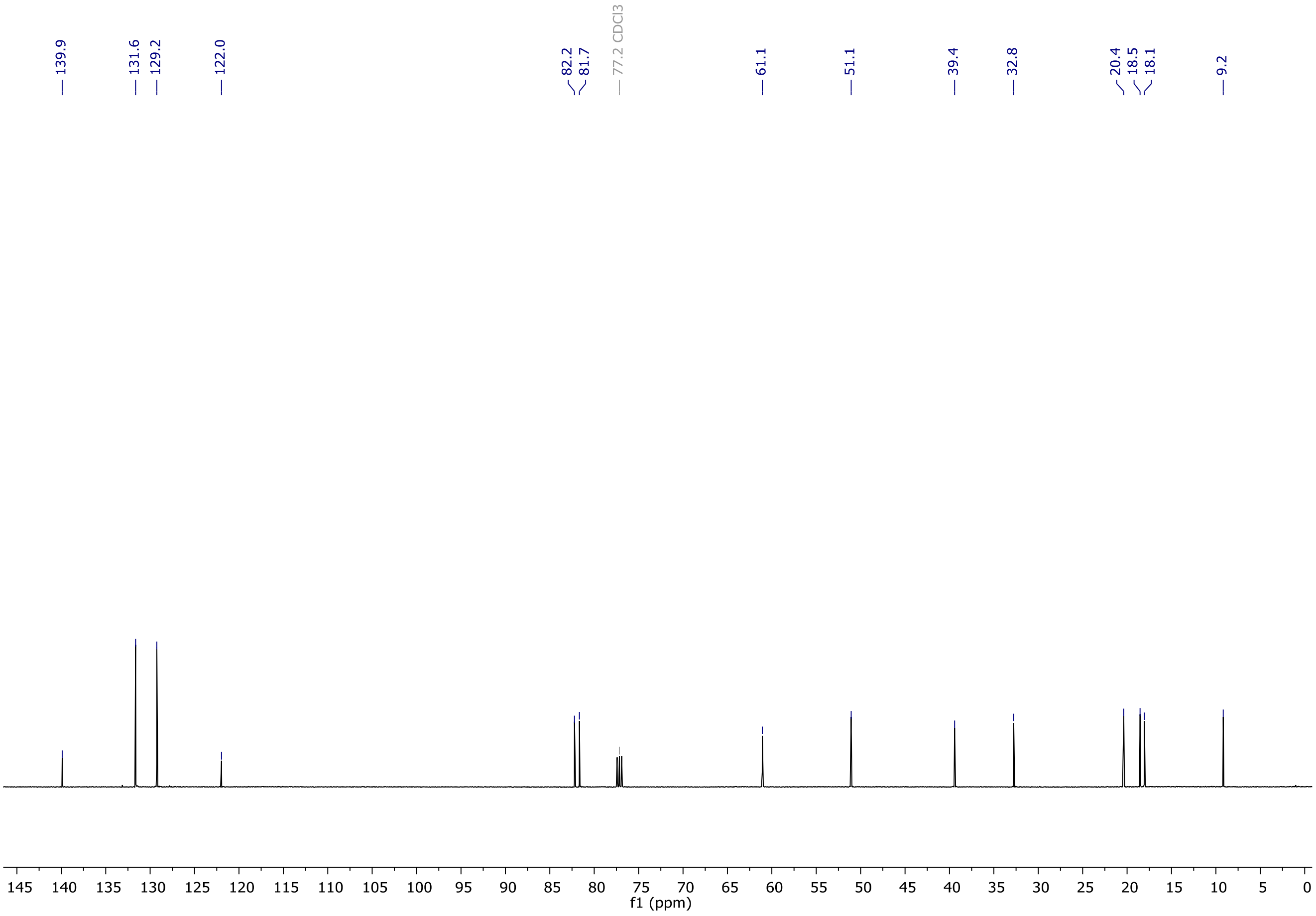

2D-COSY

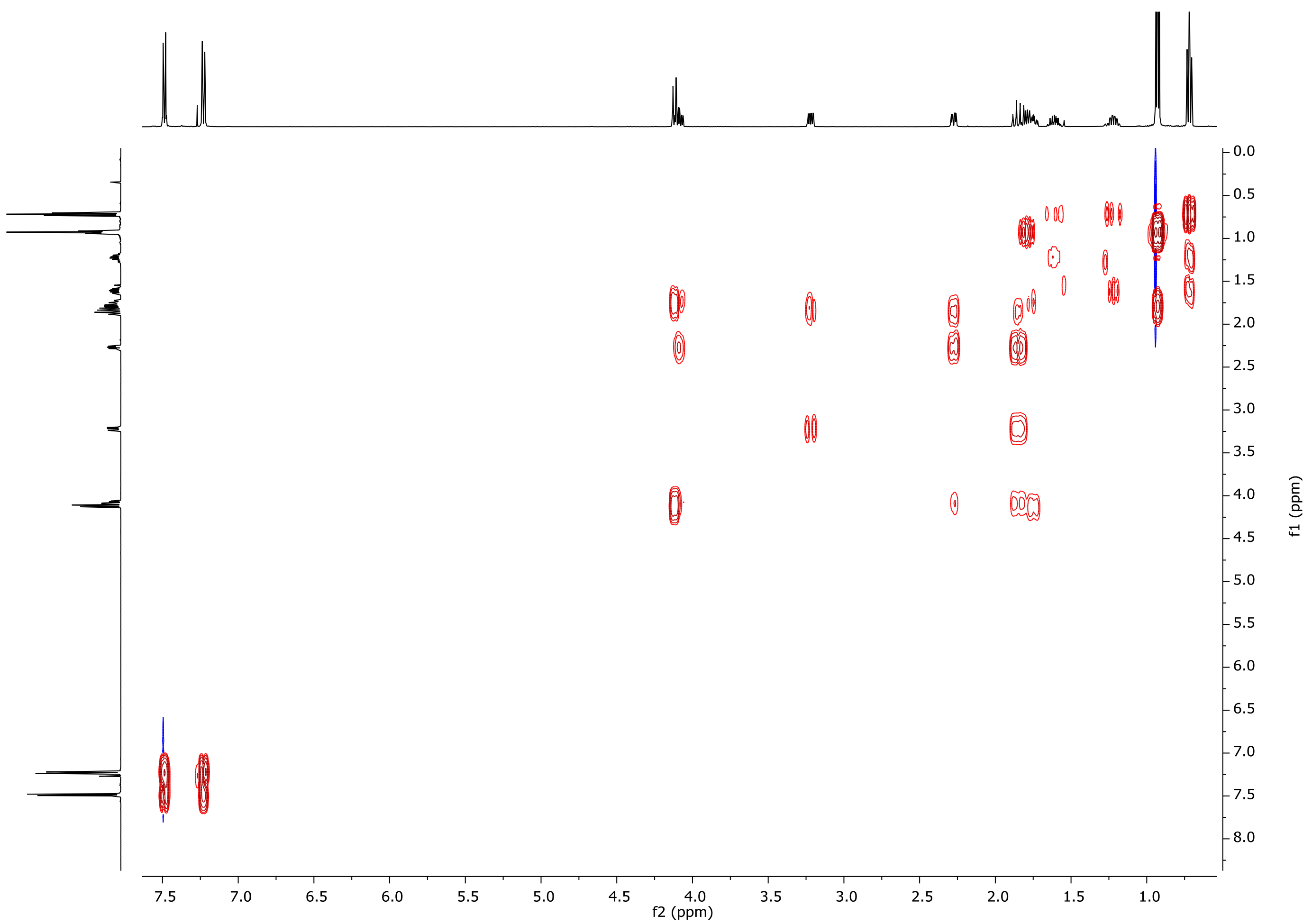

2D-HSQC

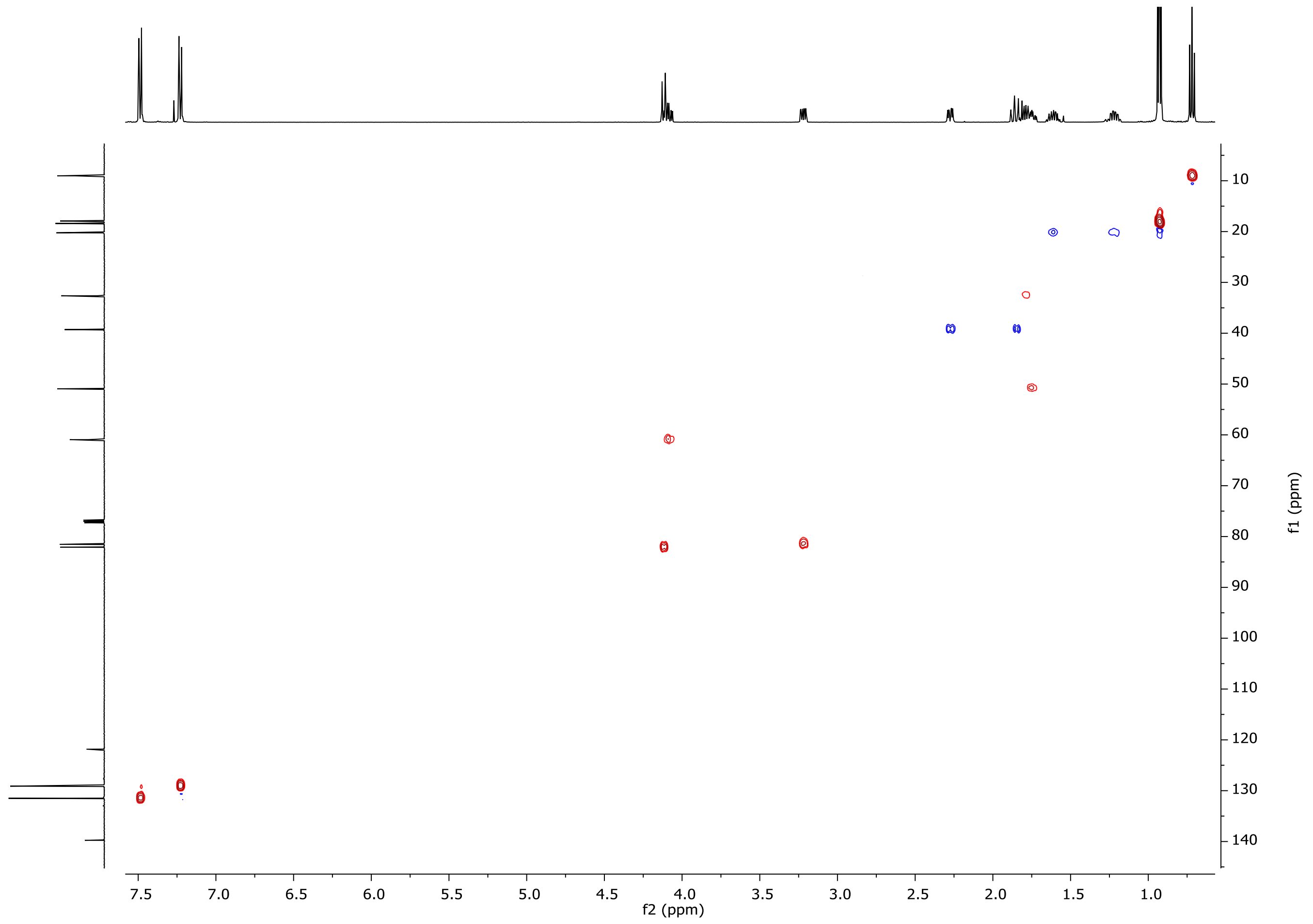

Compound 4af

^{1}H NMR (500 MHz, $CDCl_3$)

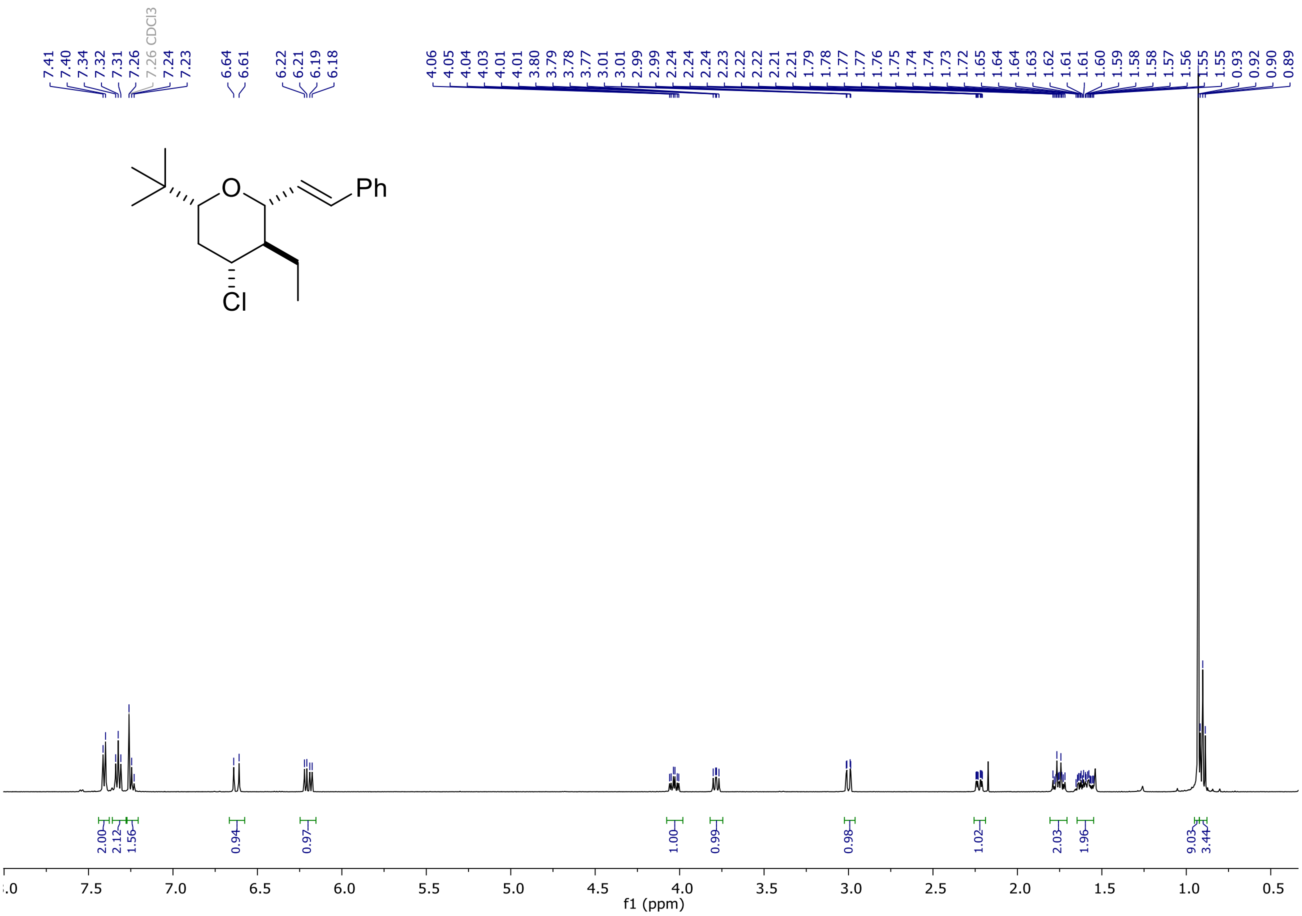

^{13}C NMR (101 MHz, $CDCl_3$)

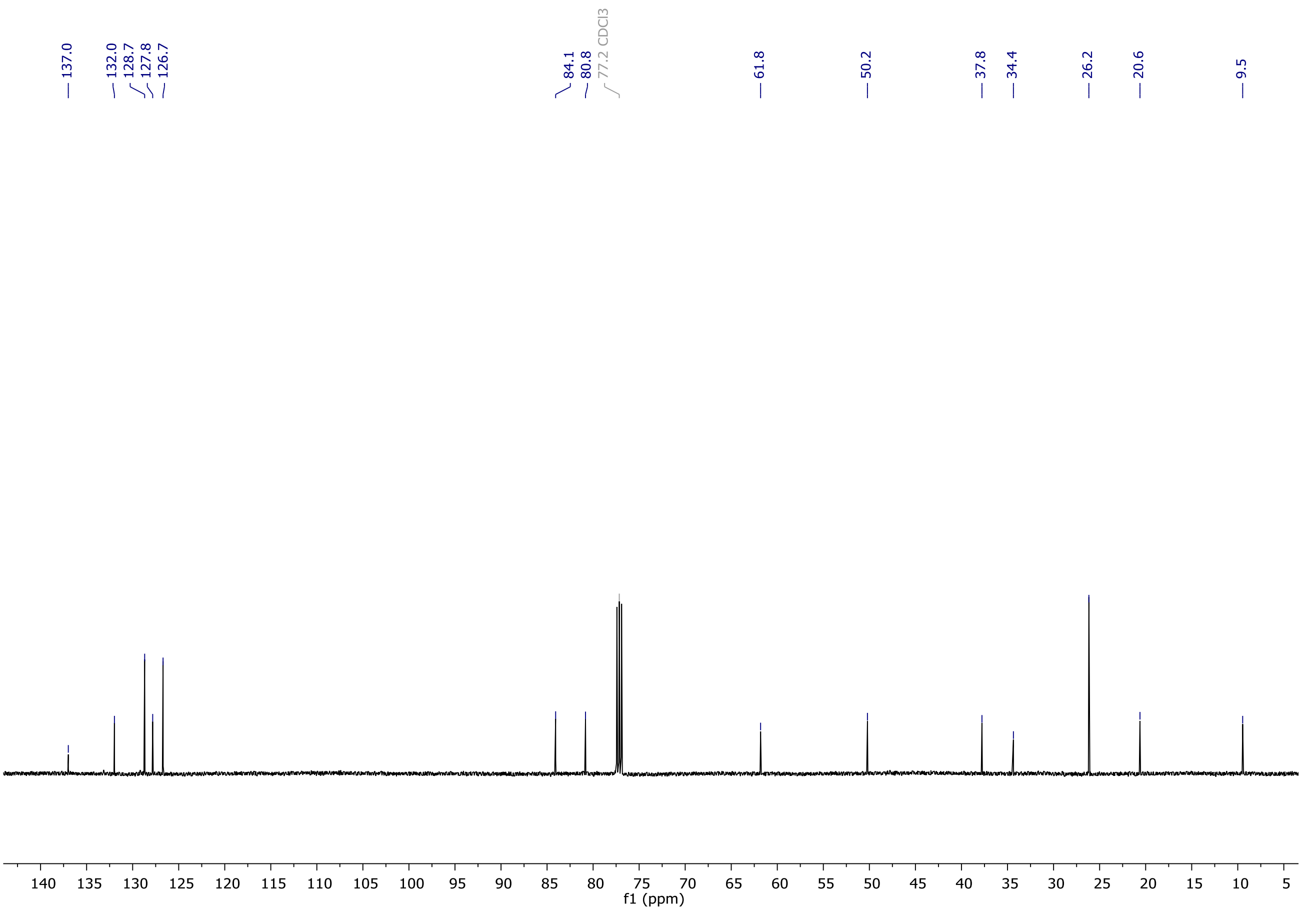

2D-COSY

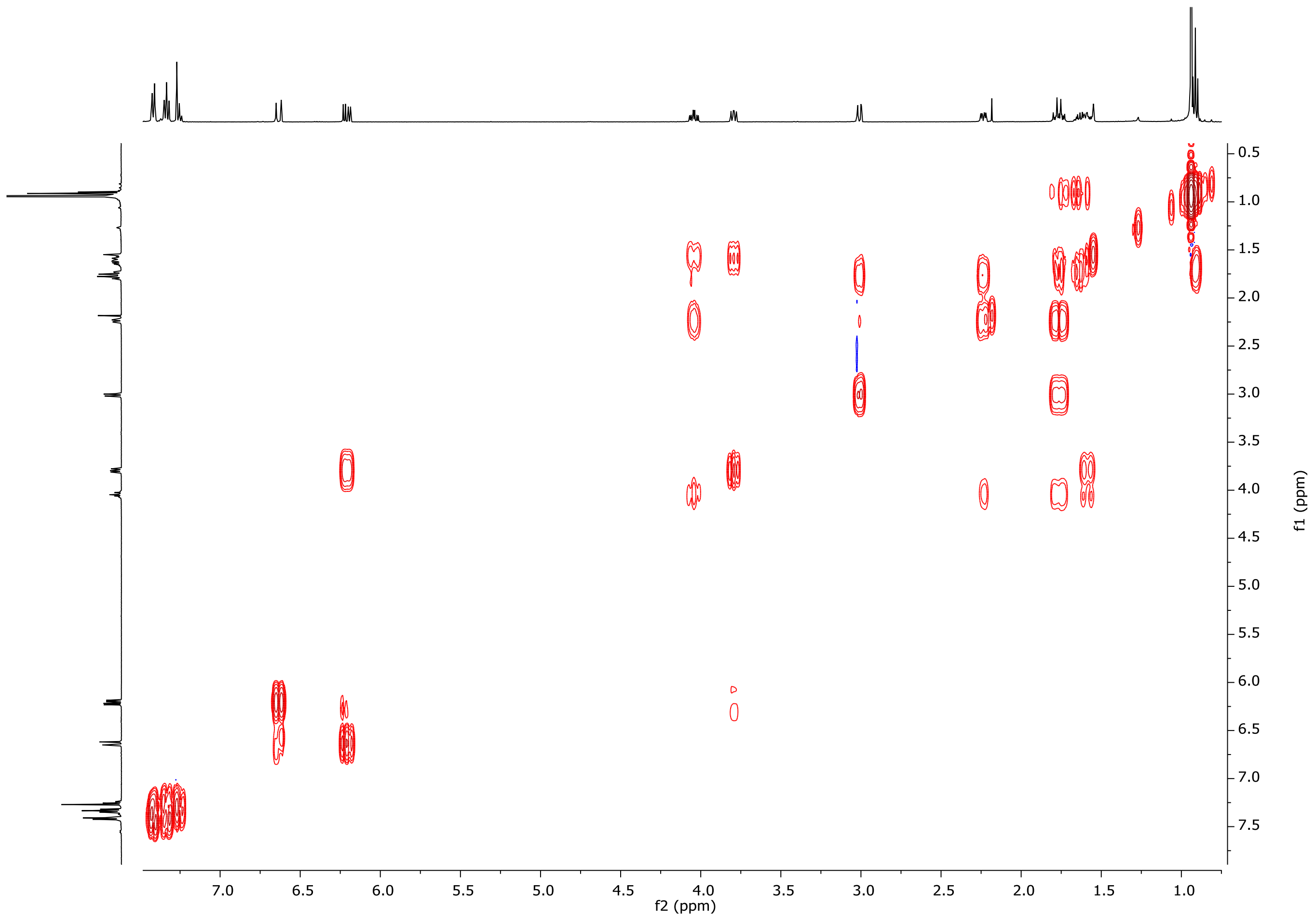

2D-HSQC

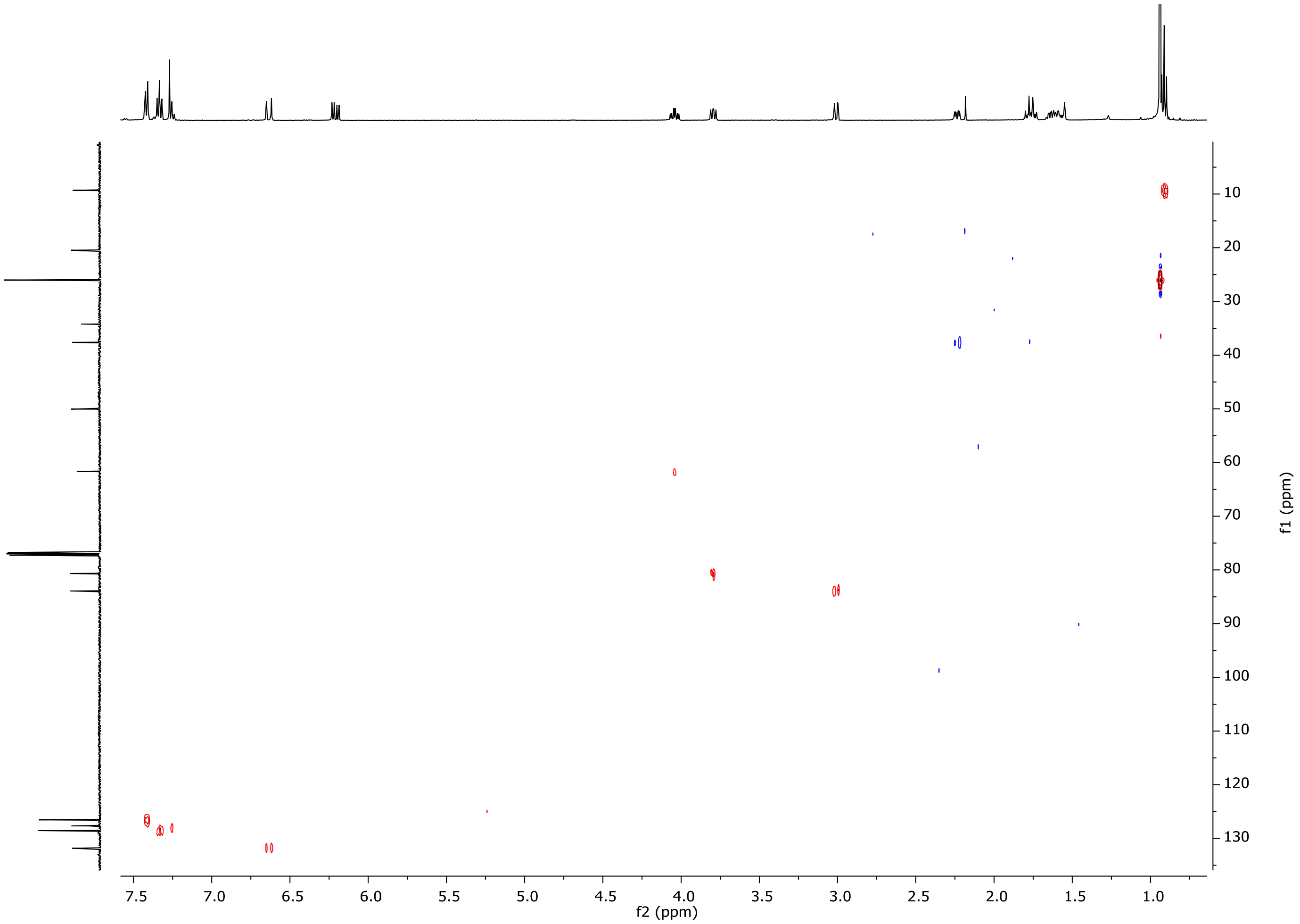

Compound 4ag

^{1}**H NMR** (500 MHz, $CDCl_3$)

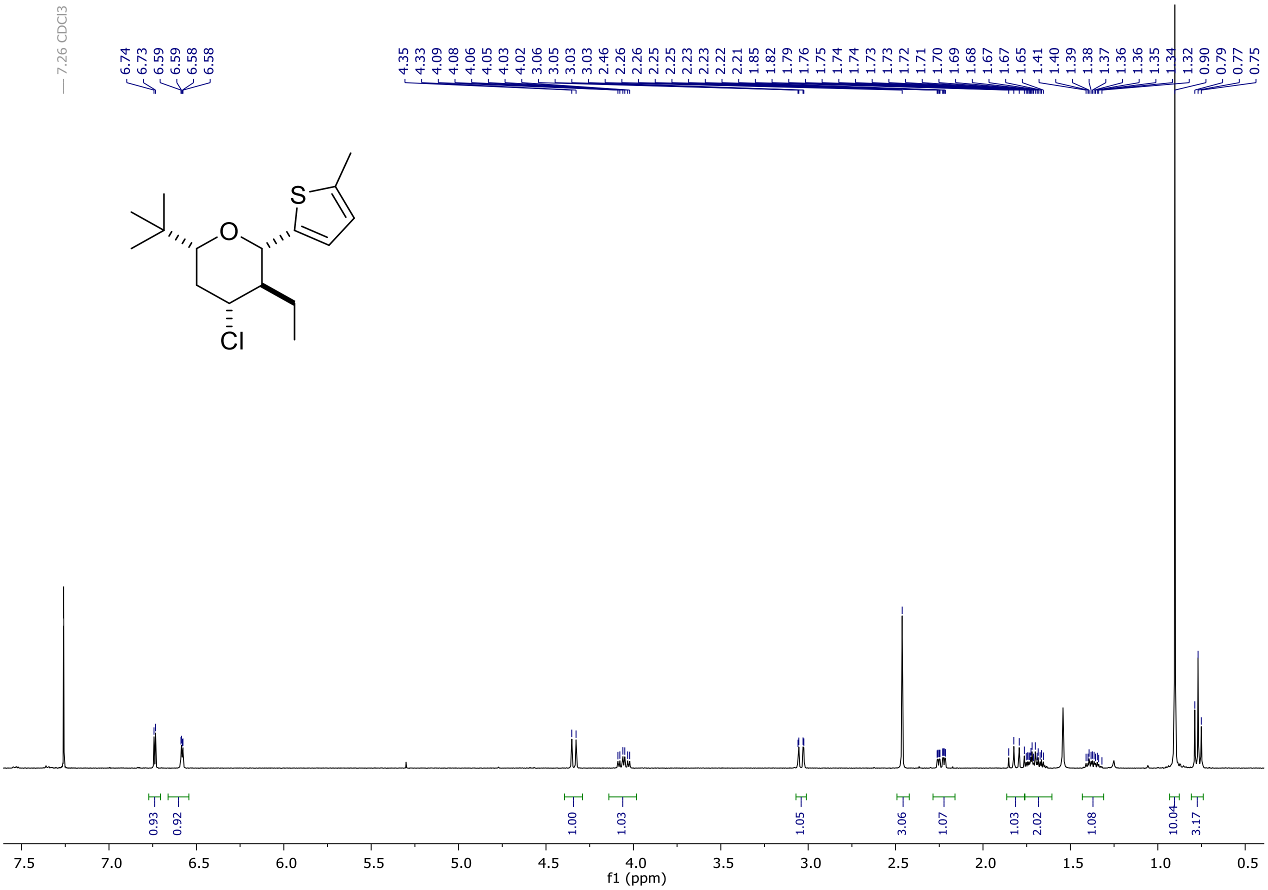

^{13}**C NMR** (101 MHz, $CDCl_3$)

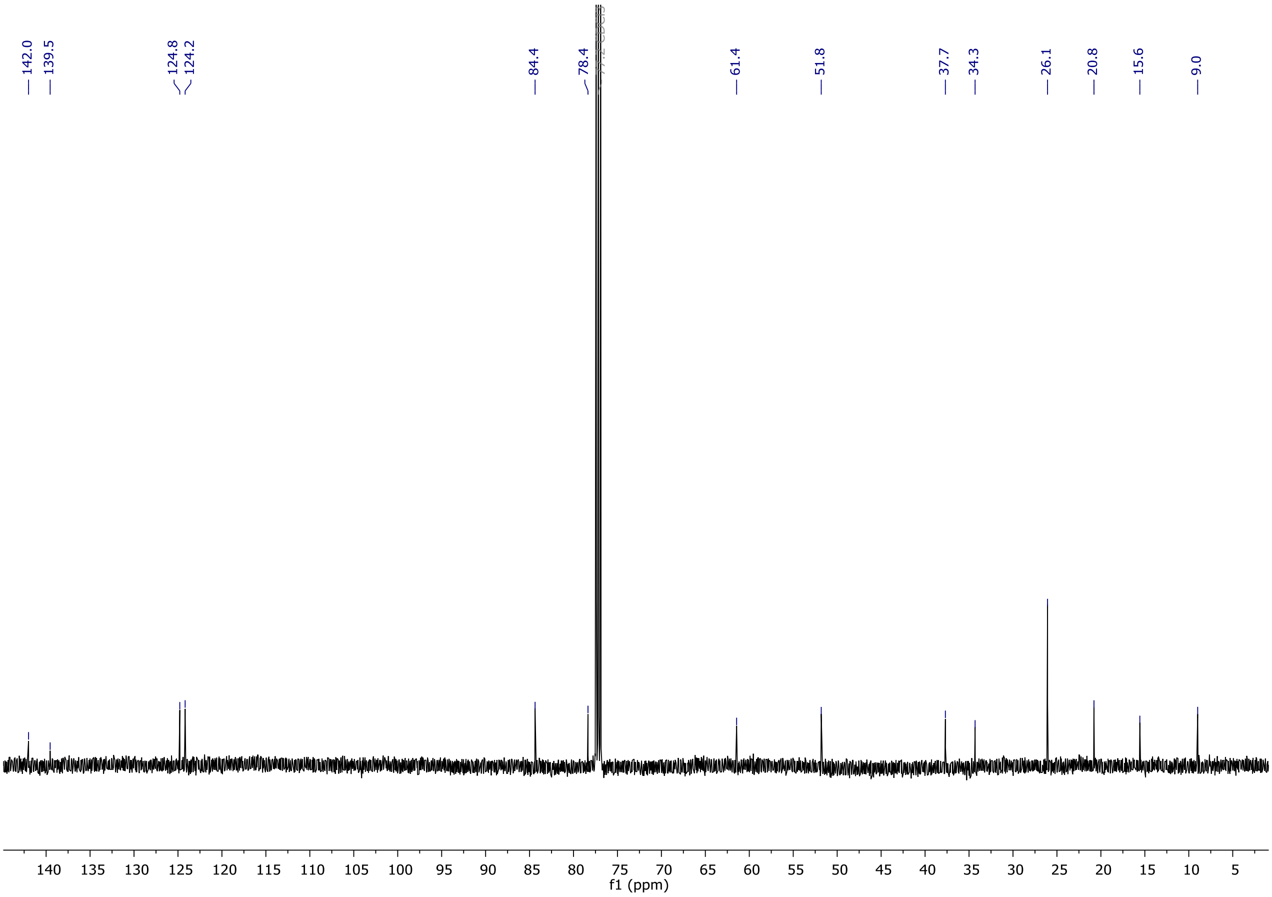

2D-COSY

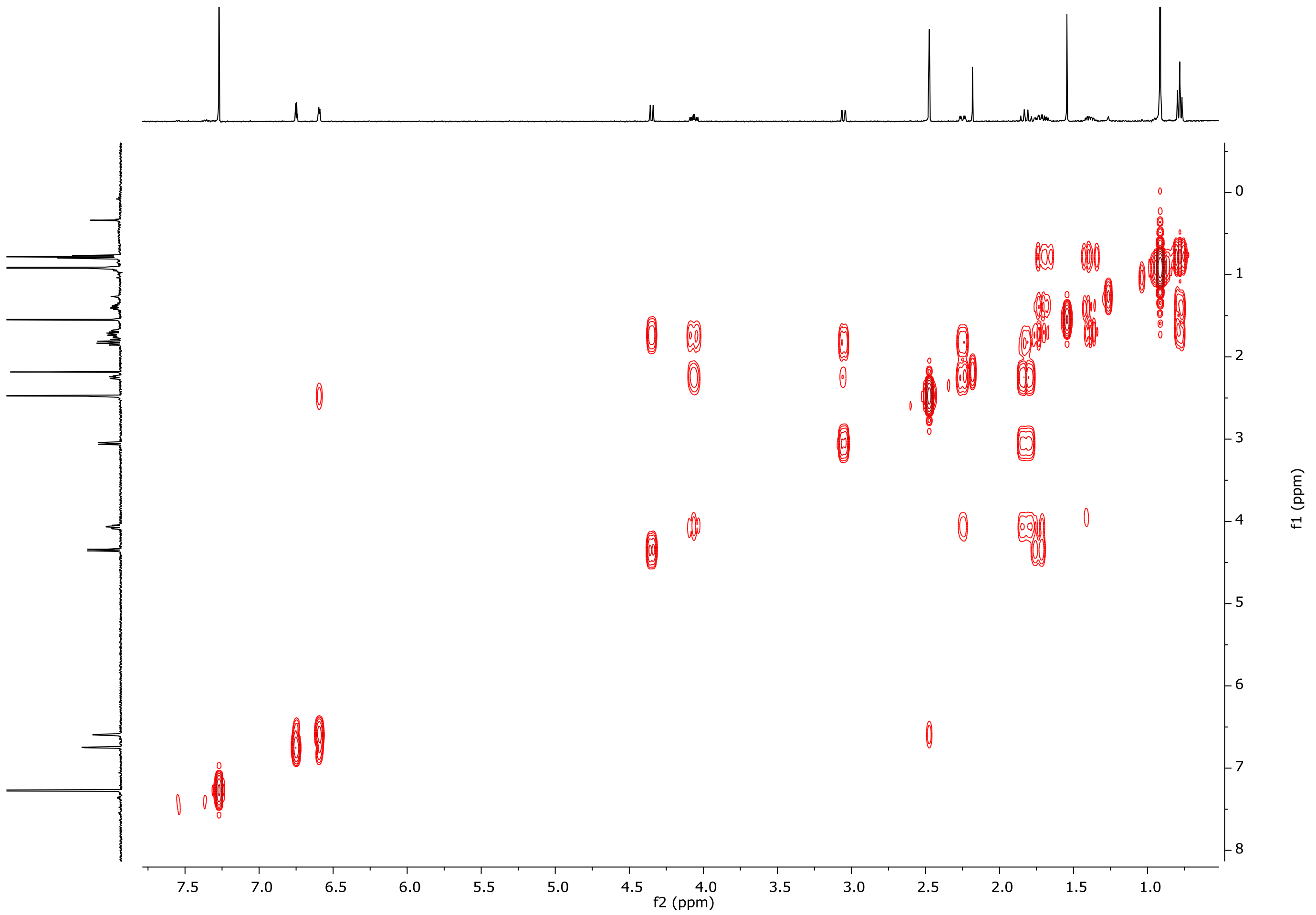

2D-HSQC

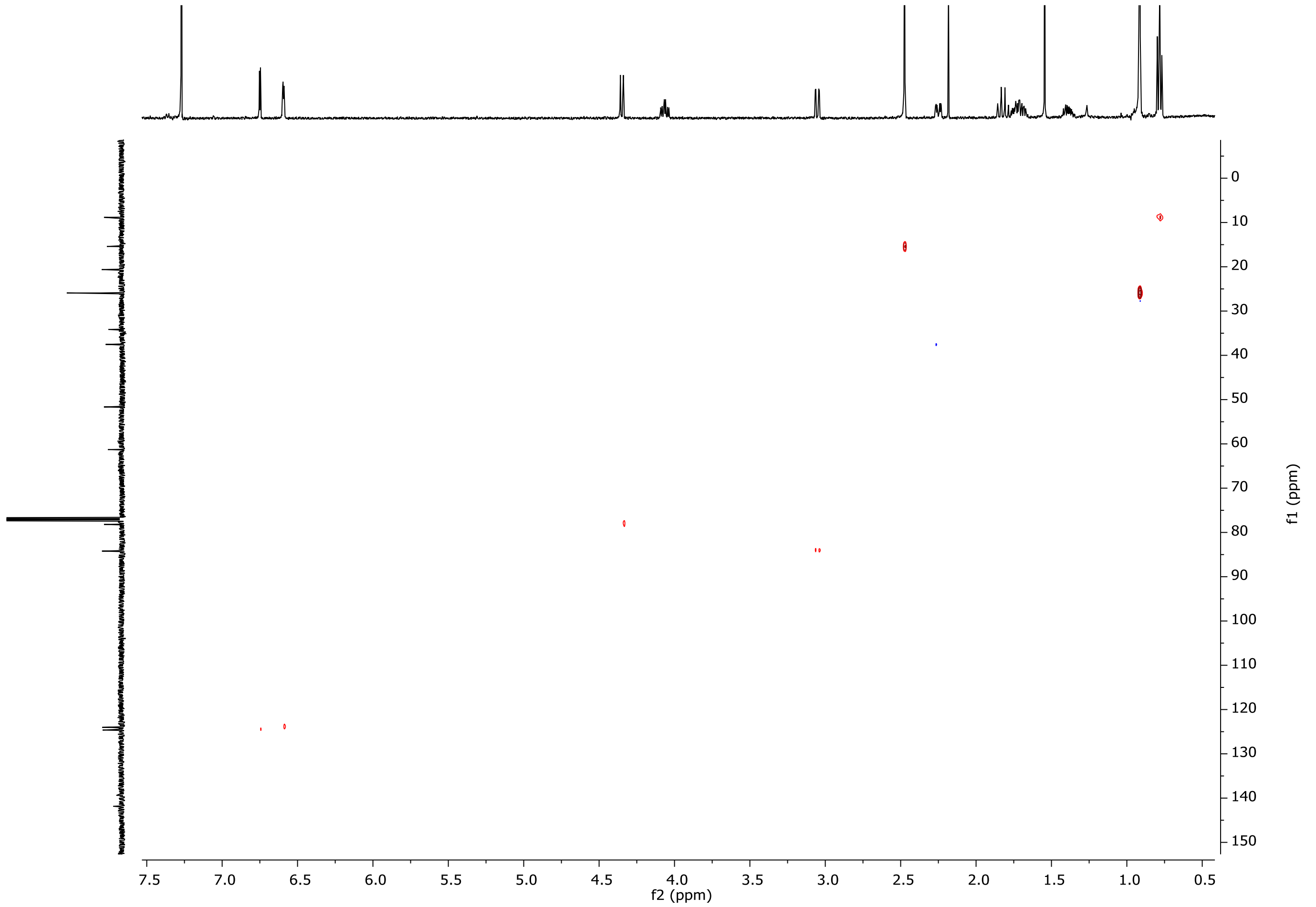

Compound 7a

^{1}H NMR (500 MHz, $CDCl_3$)

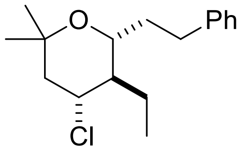

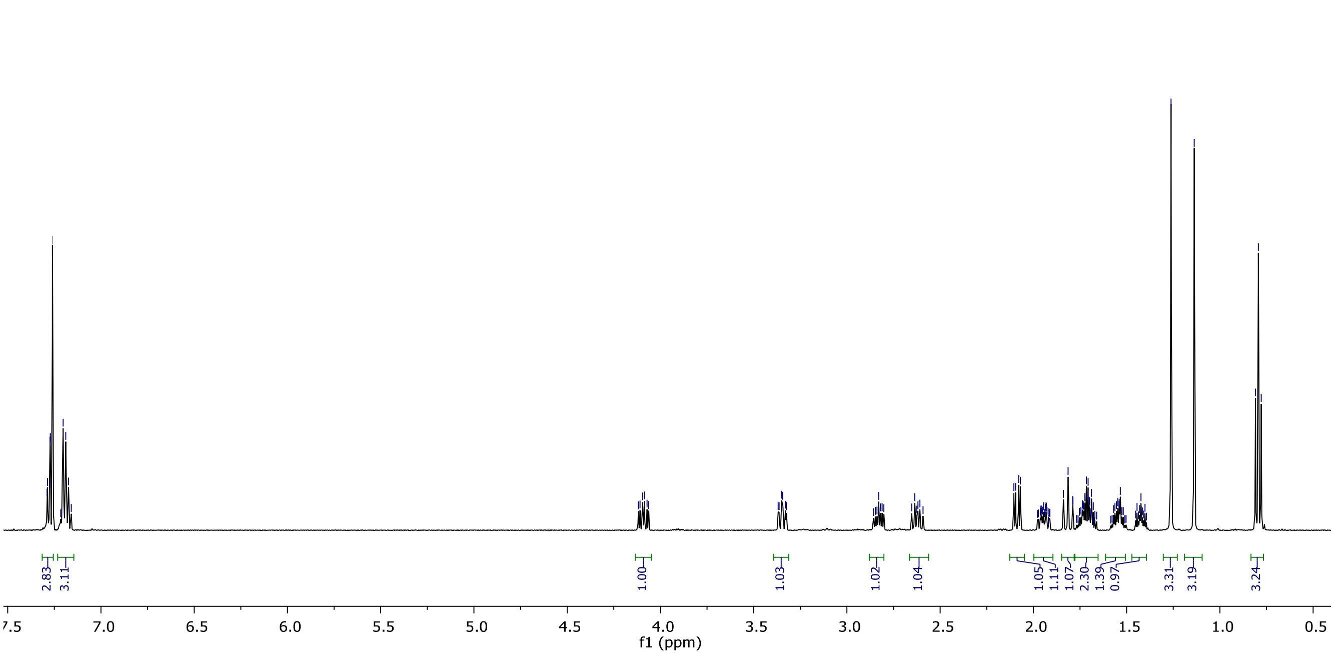

^{13}C NMR (101 MHz, $CDCl_3$)

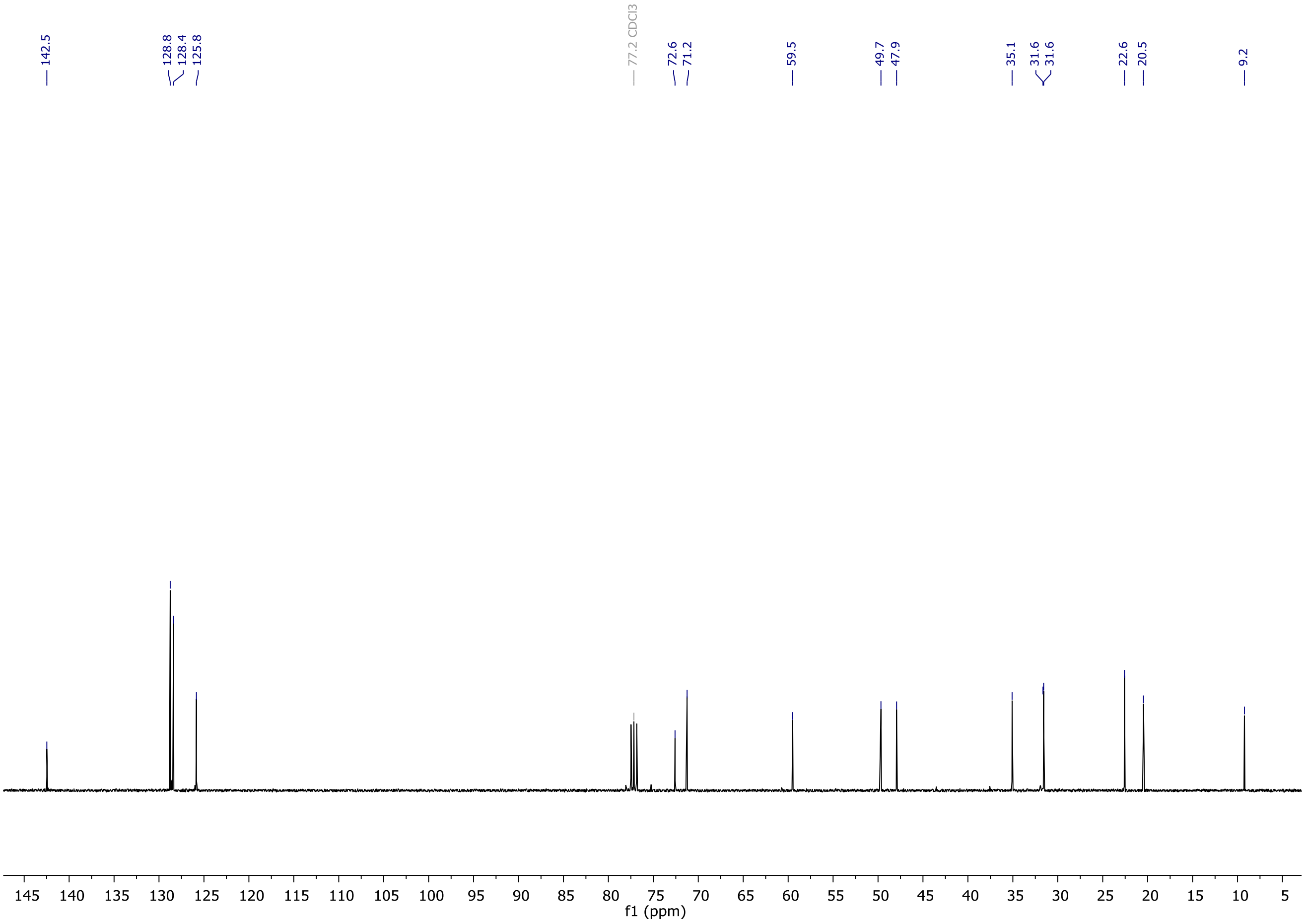

2D-COSY

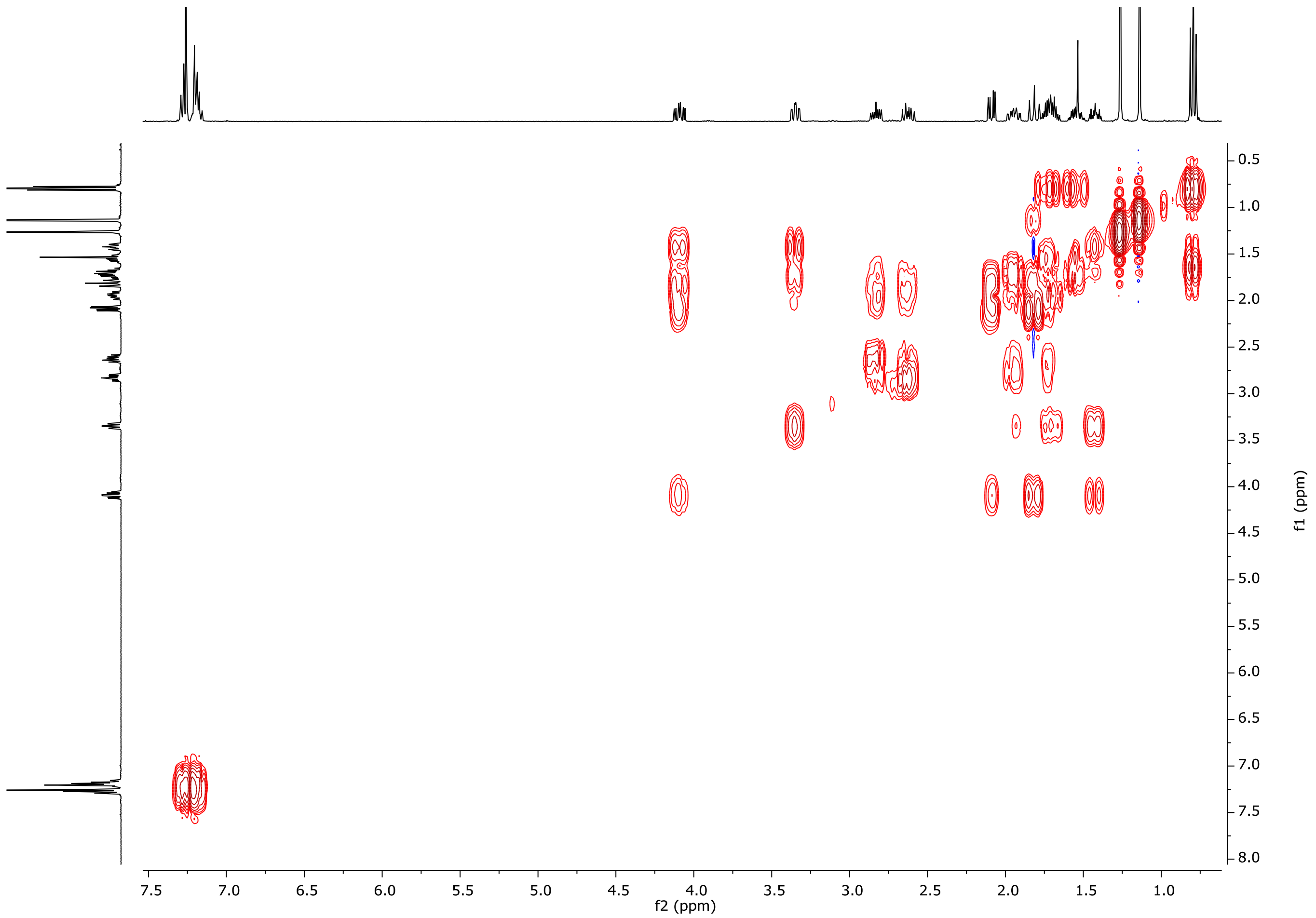

2D-HSQC

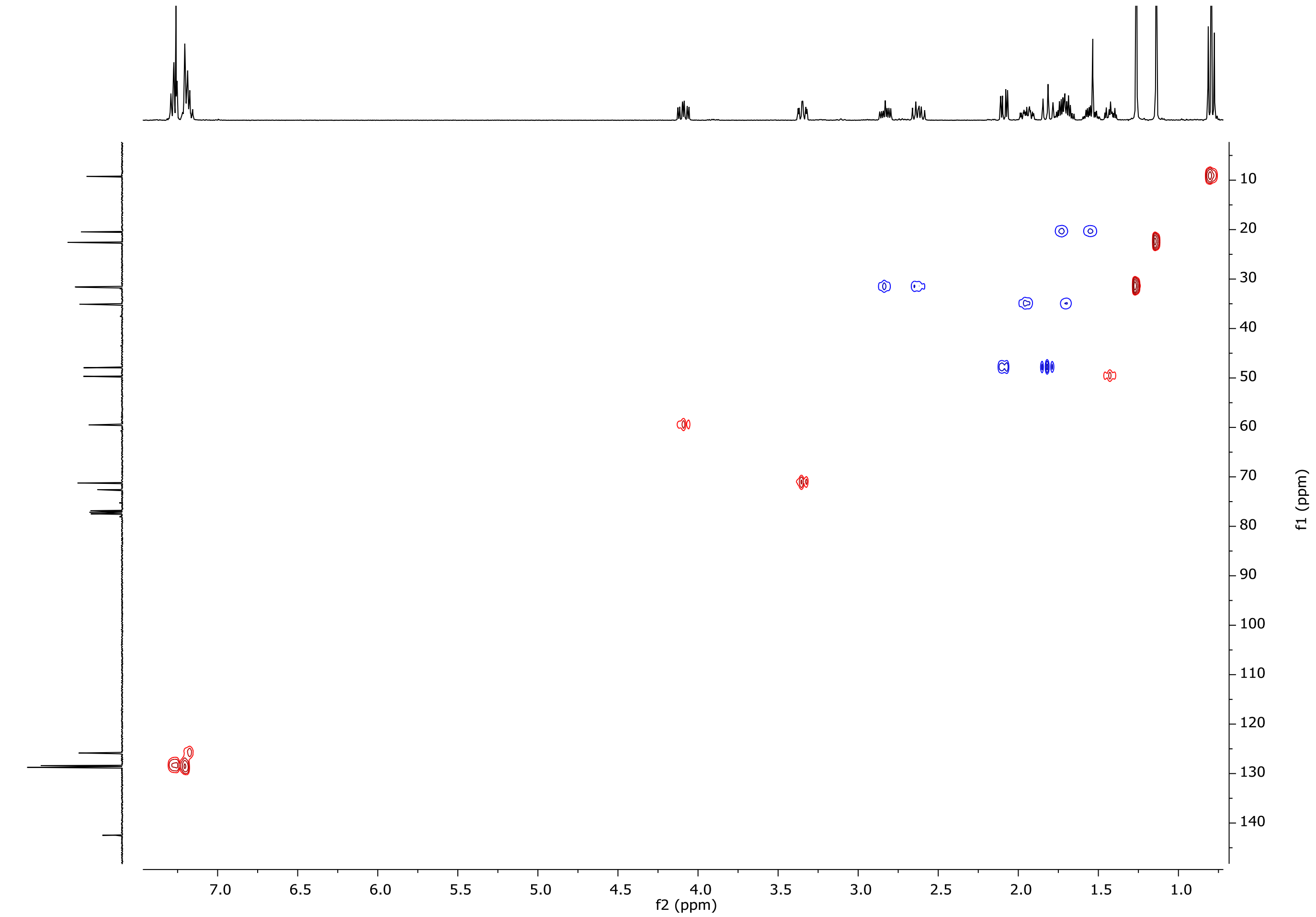

Compound 7b

^{1}**H NMR** (500 MHz, $CDCl_3$)

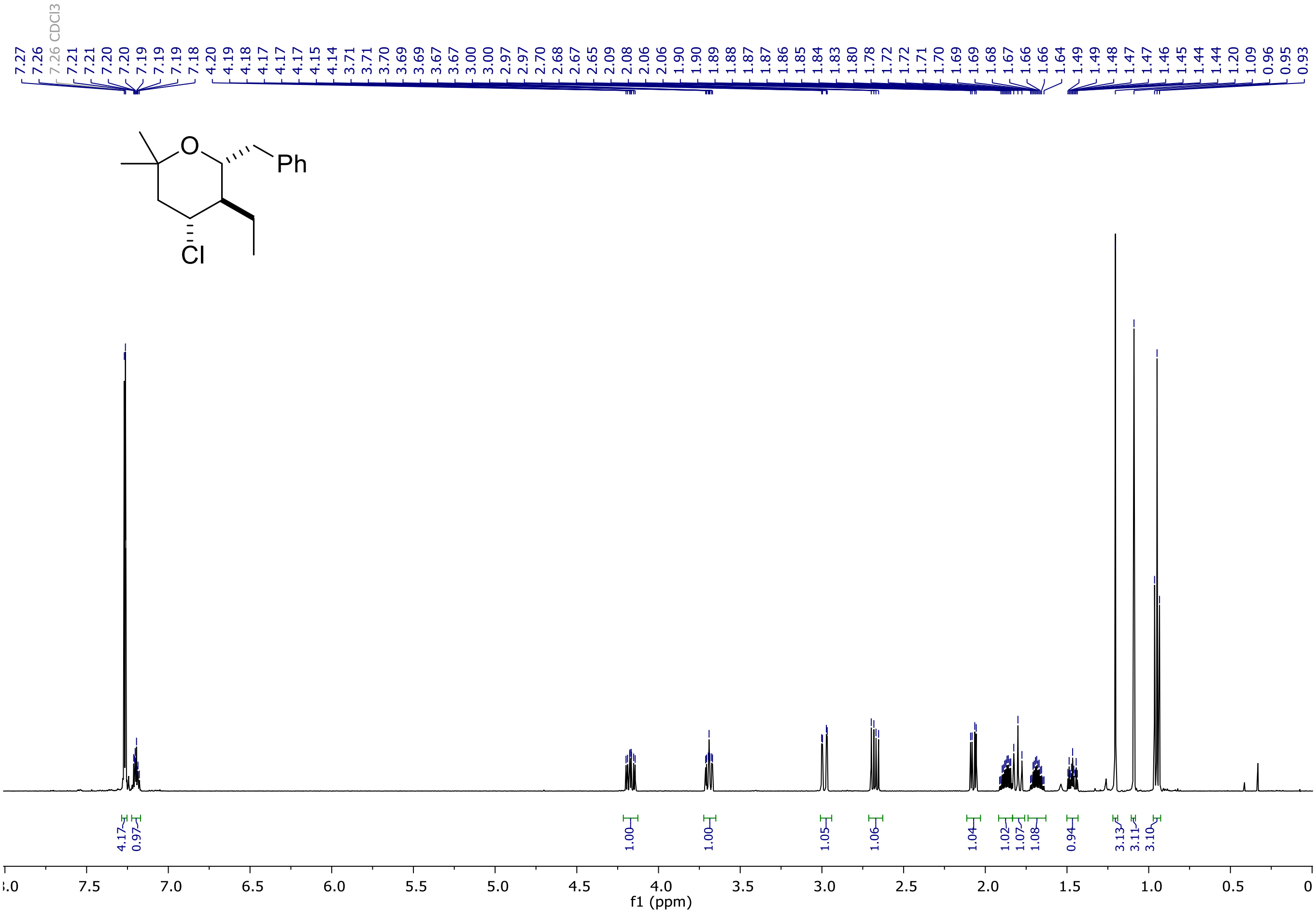

^{13}**C NMR** (101 MHz, $CDCl_3$)

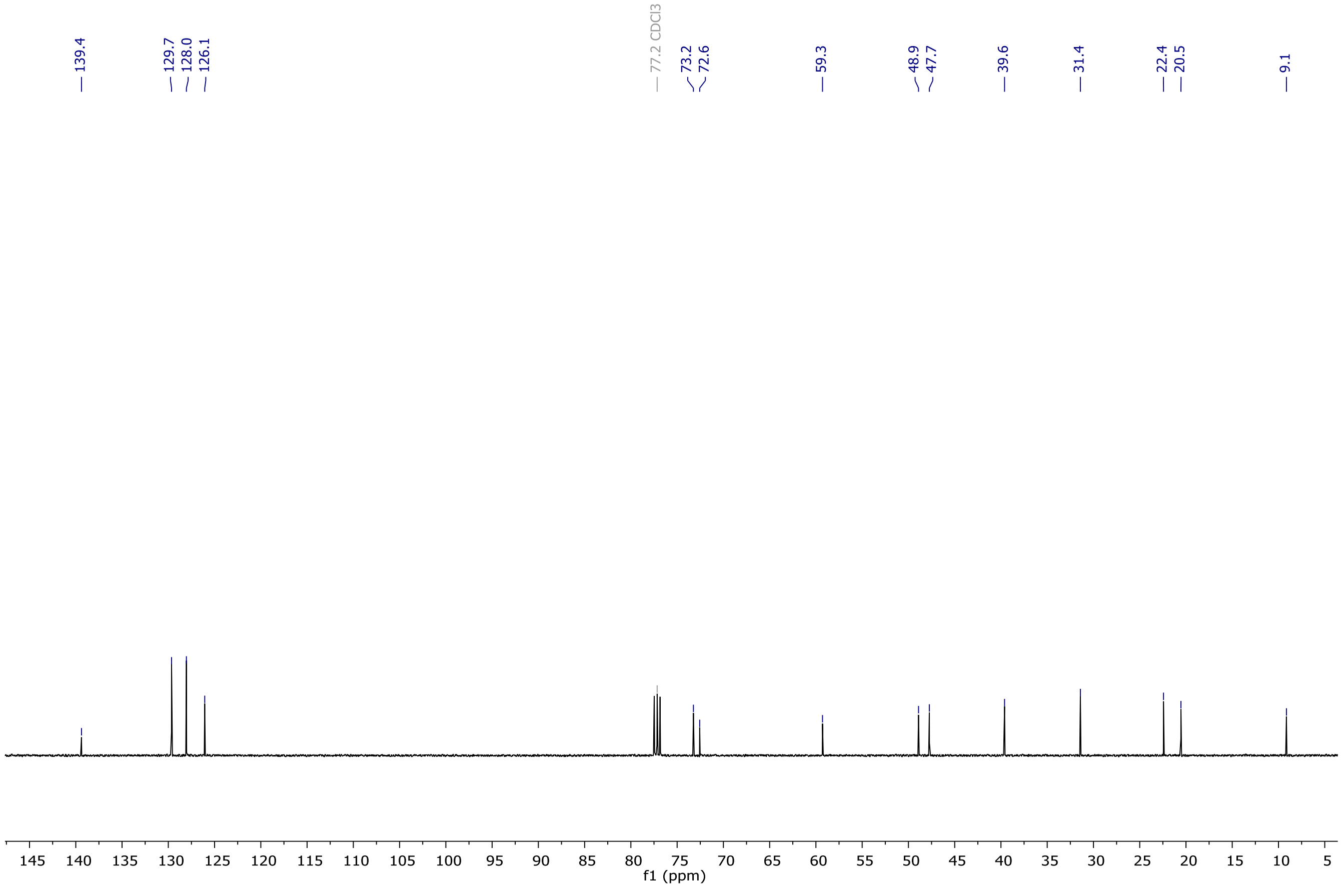

2D-COSY

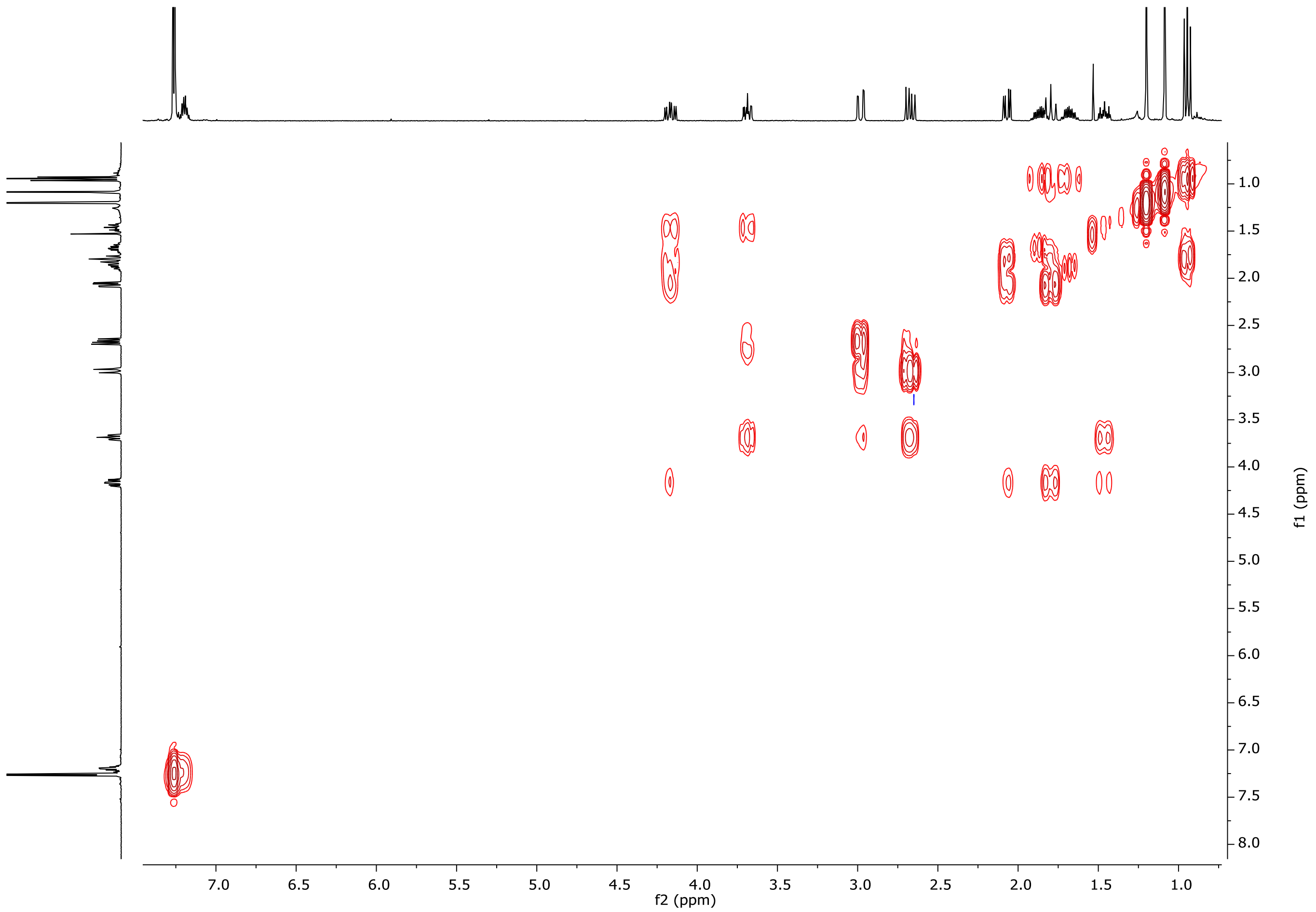

2D-HSQC

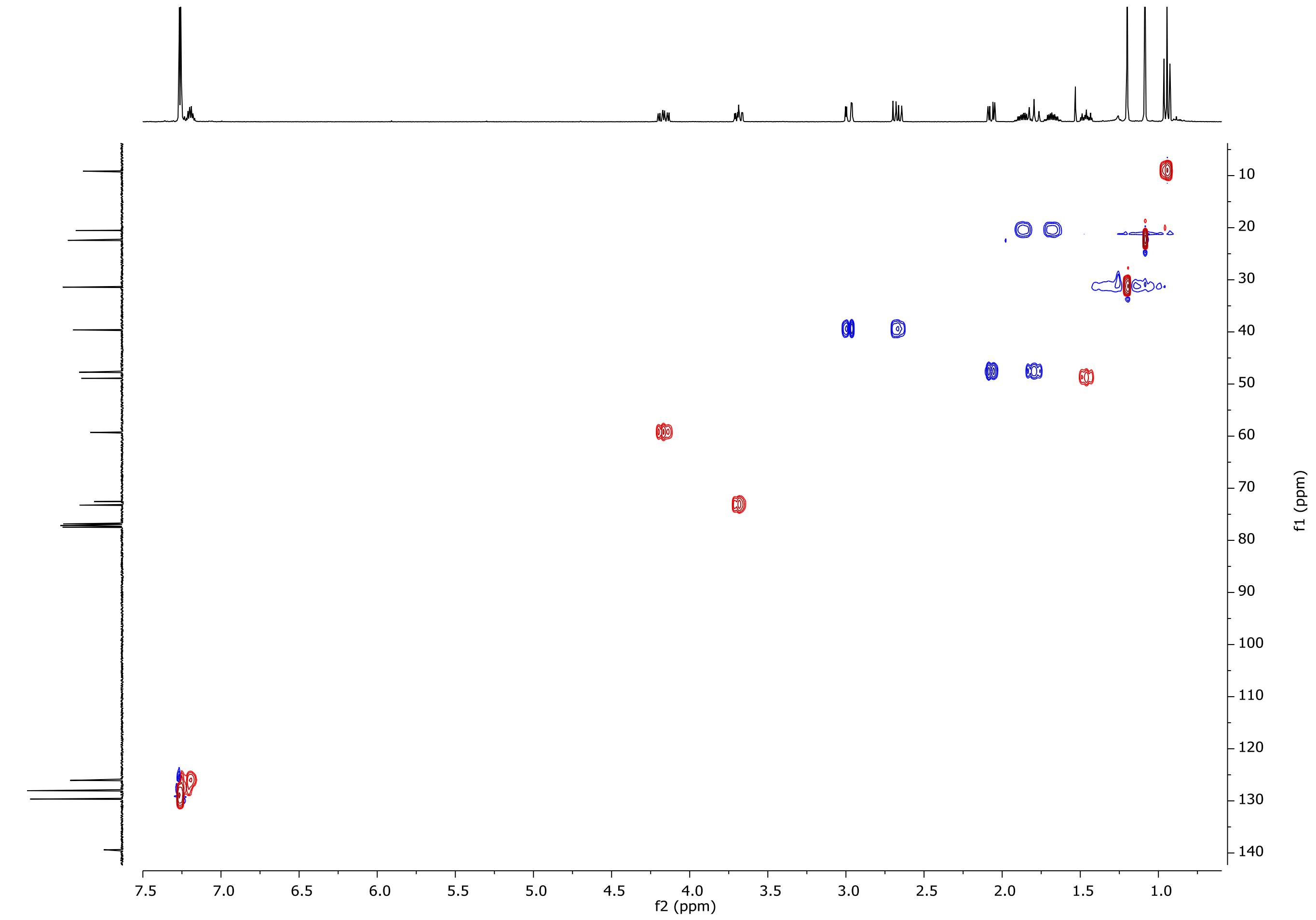

Compound 7c

^{1}H NMR (500 MHz, $CDCl_3$)

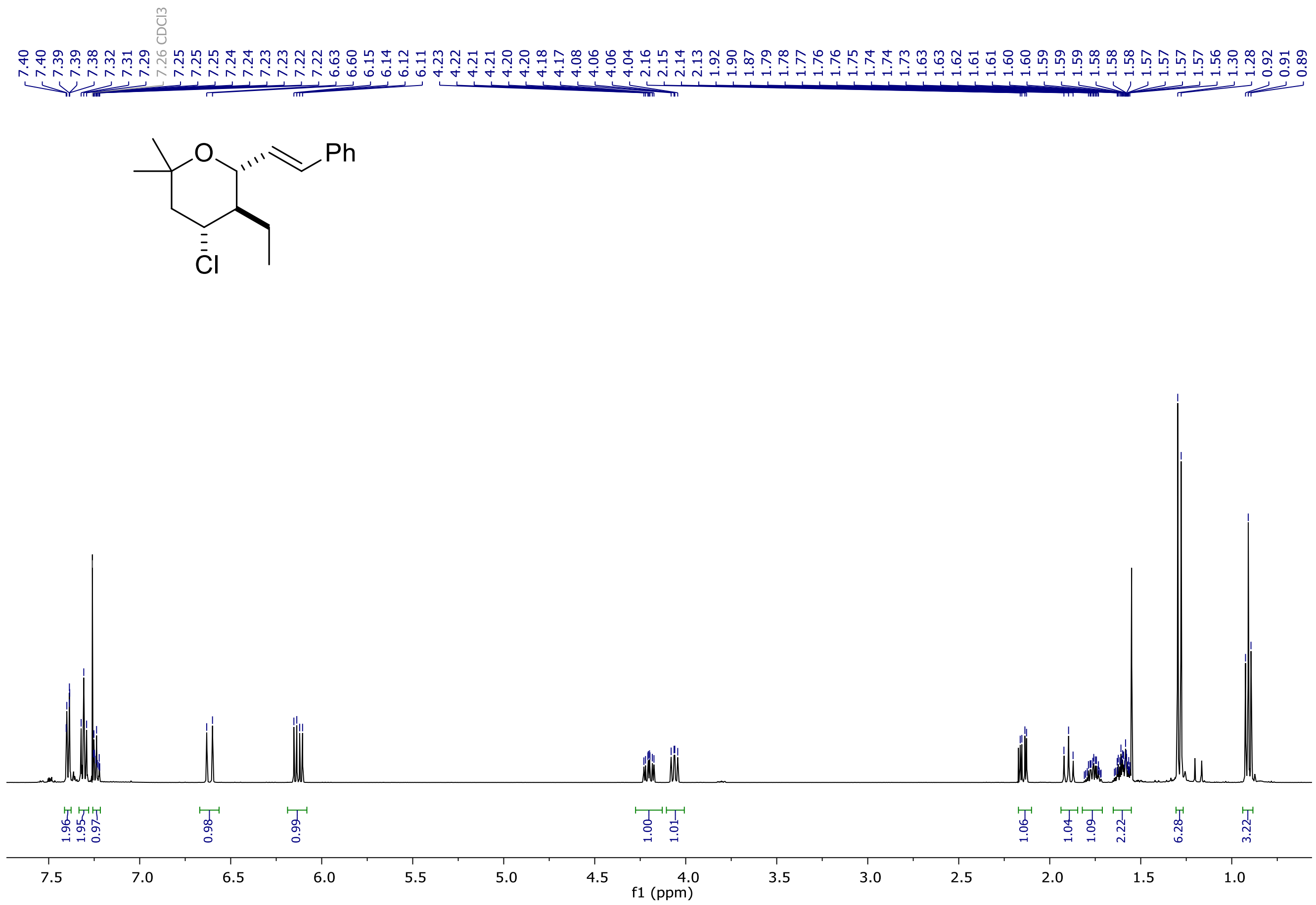

^{13}C NMR (101 MHz, $CDCl_3$)

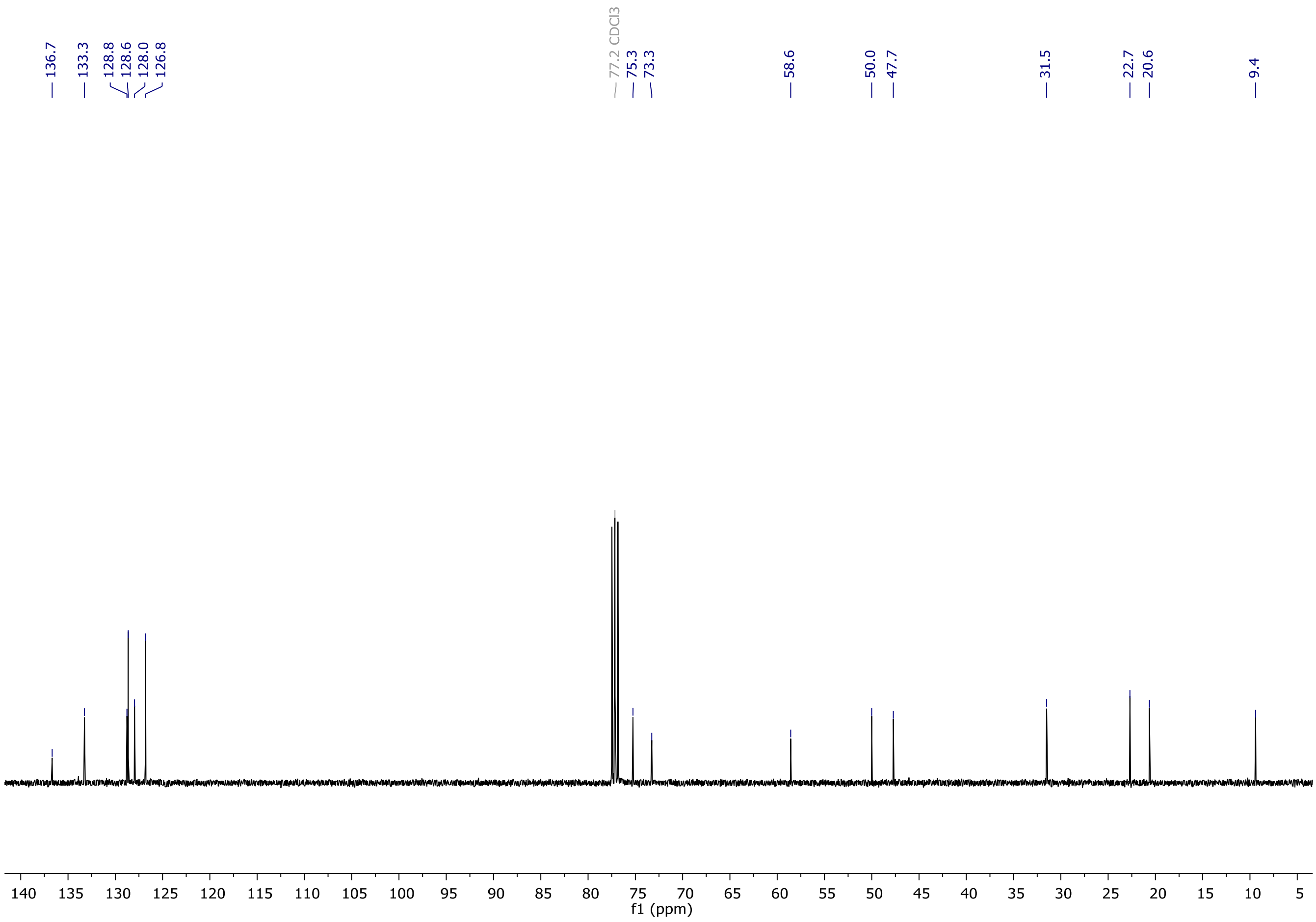

2D-COSY

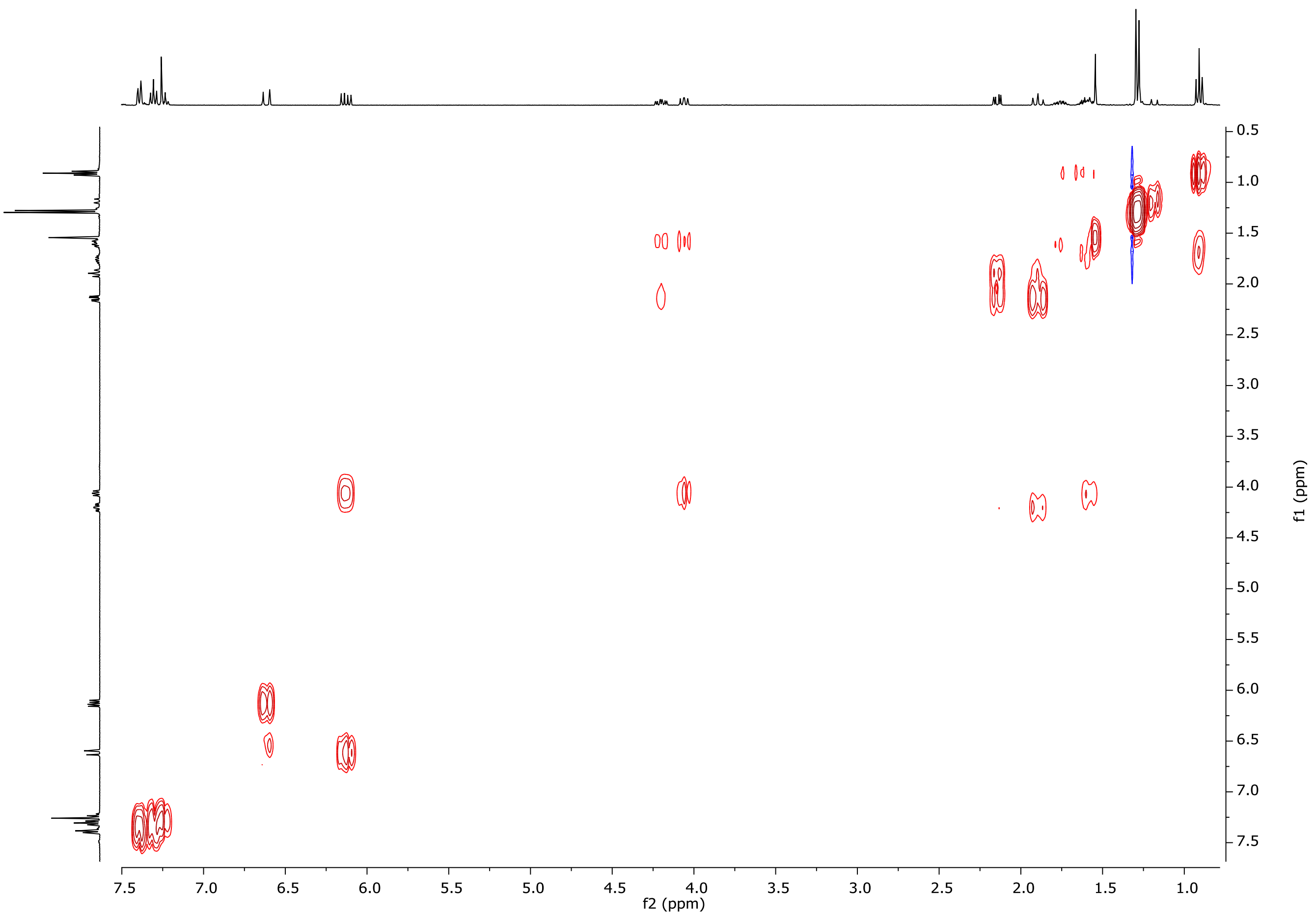

2D-HSQC

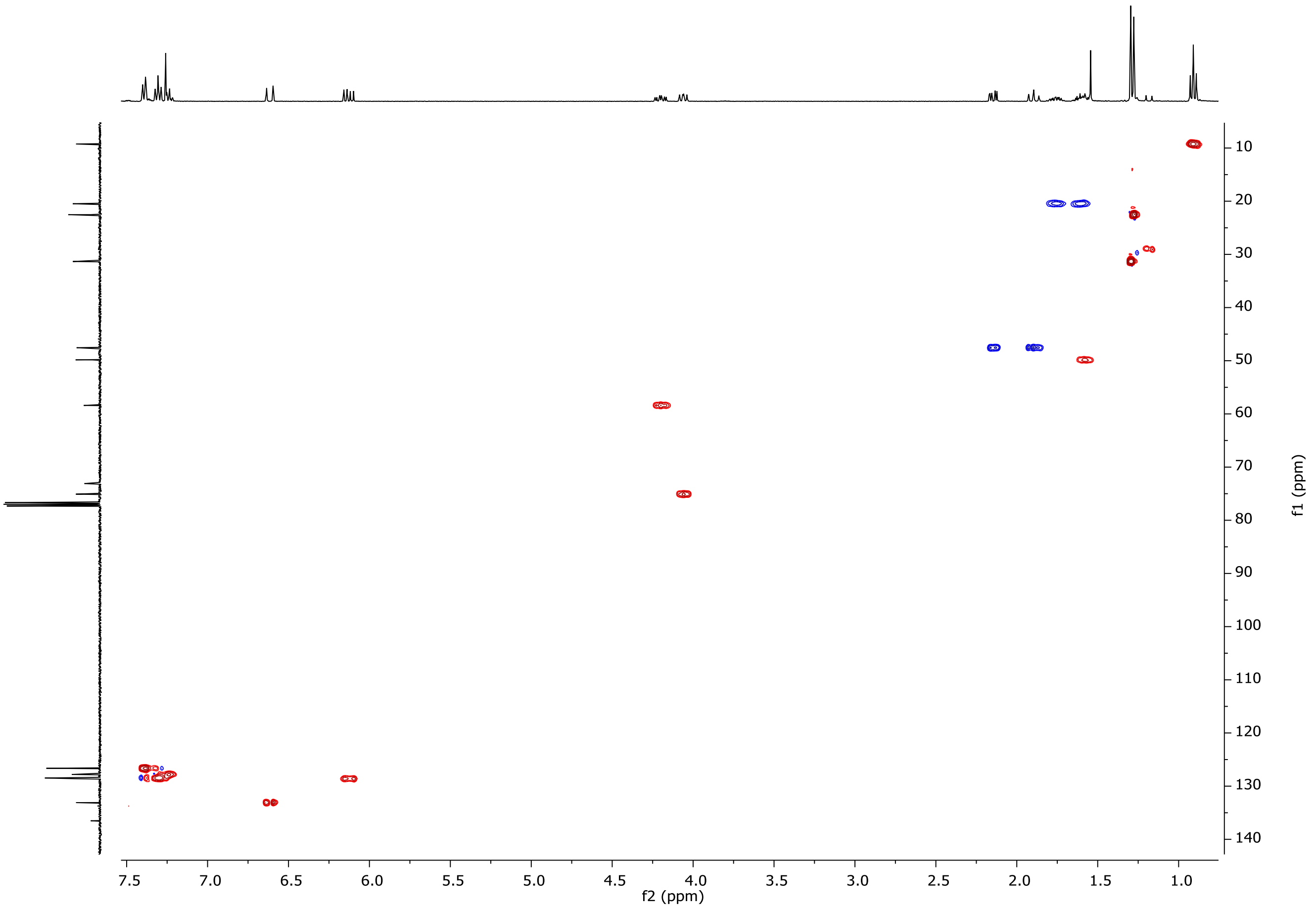

Compound 7d

^{1}H NMR (500 MHz, $CDCl_3$)

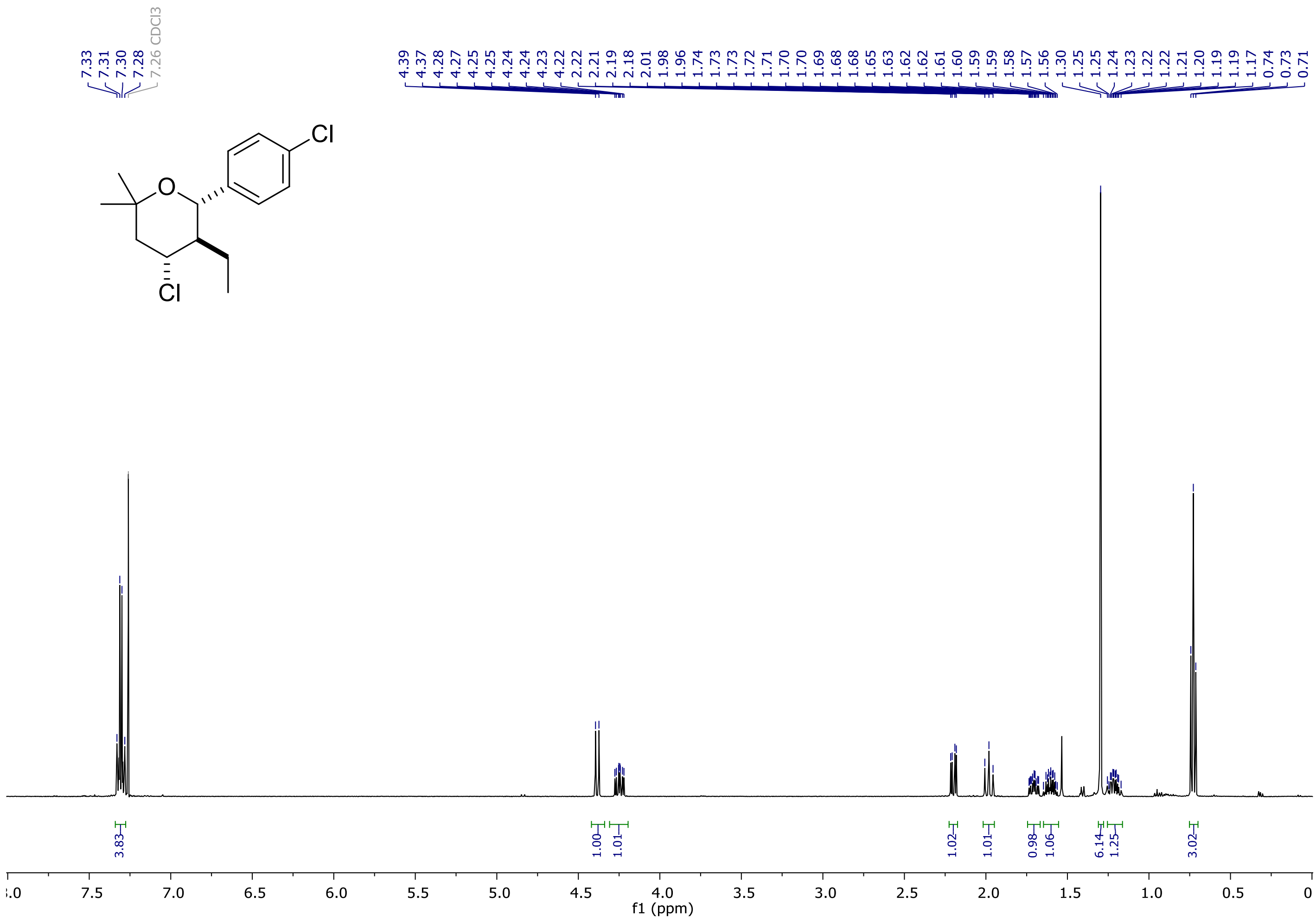

^{13}C NMR (101 MHz, $CDCl_3$)

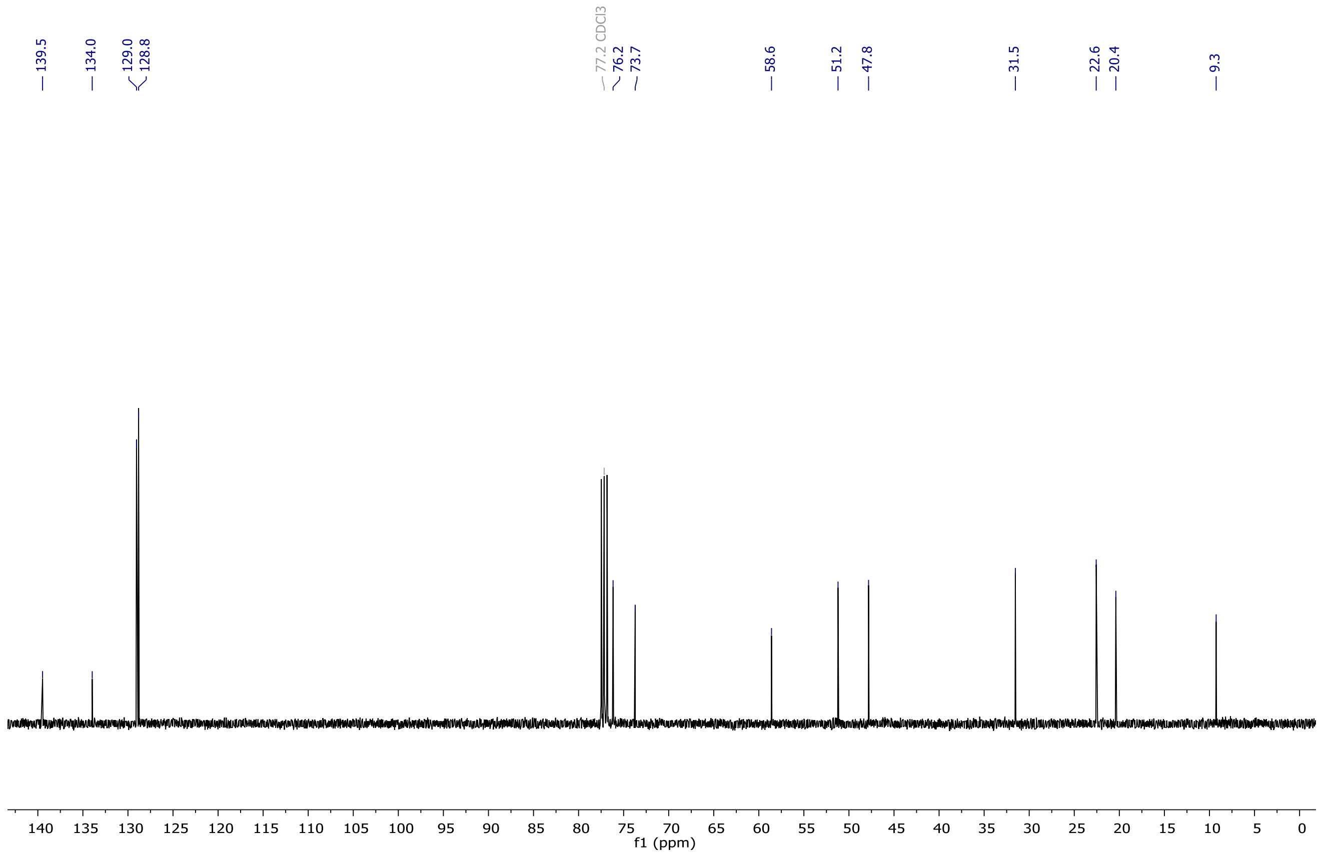

2D-COSY

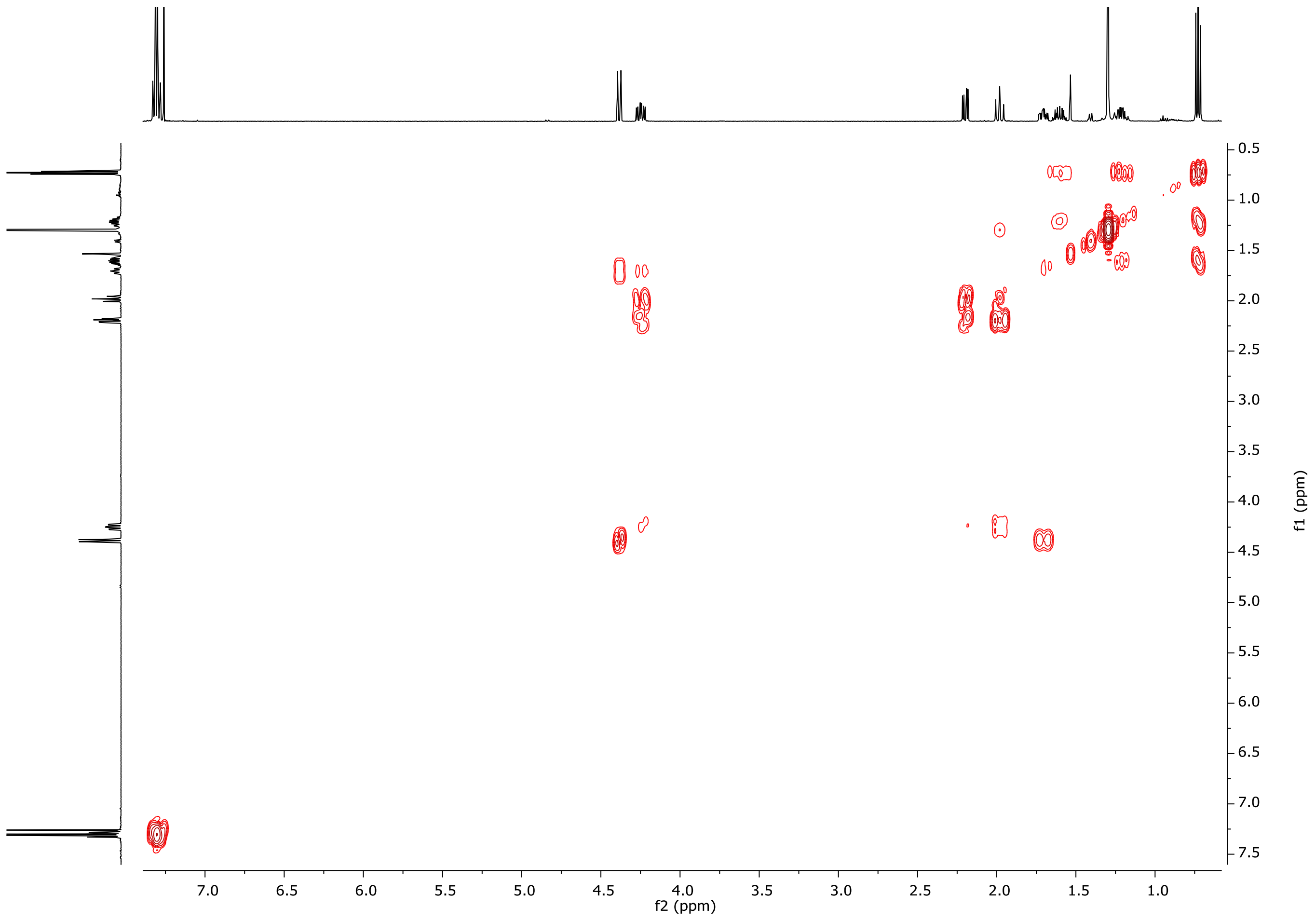

2D-HSQC

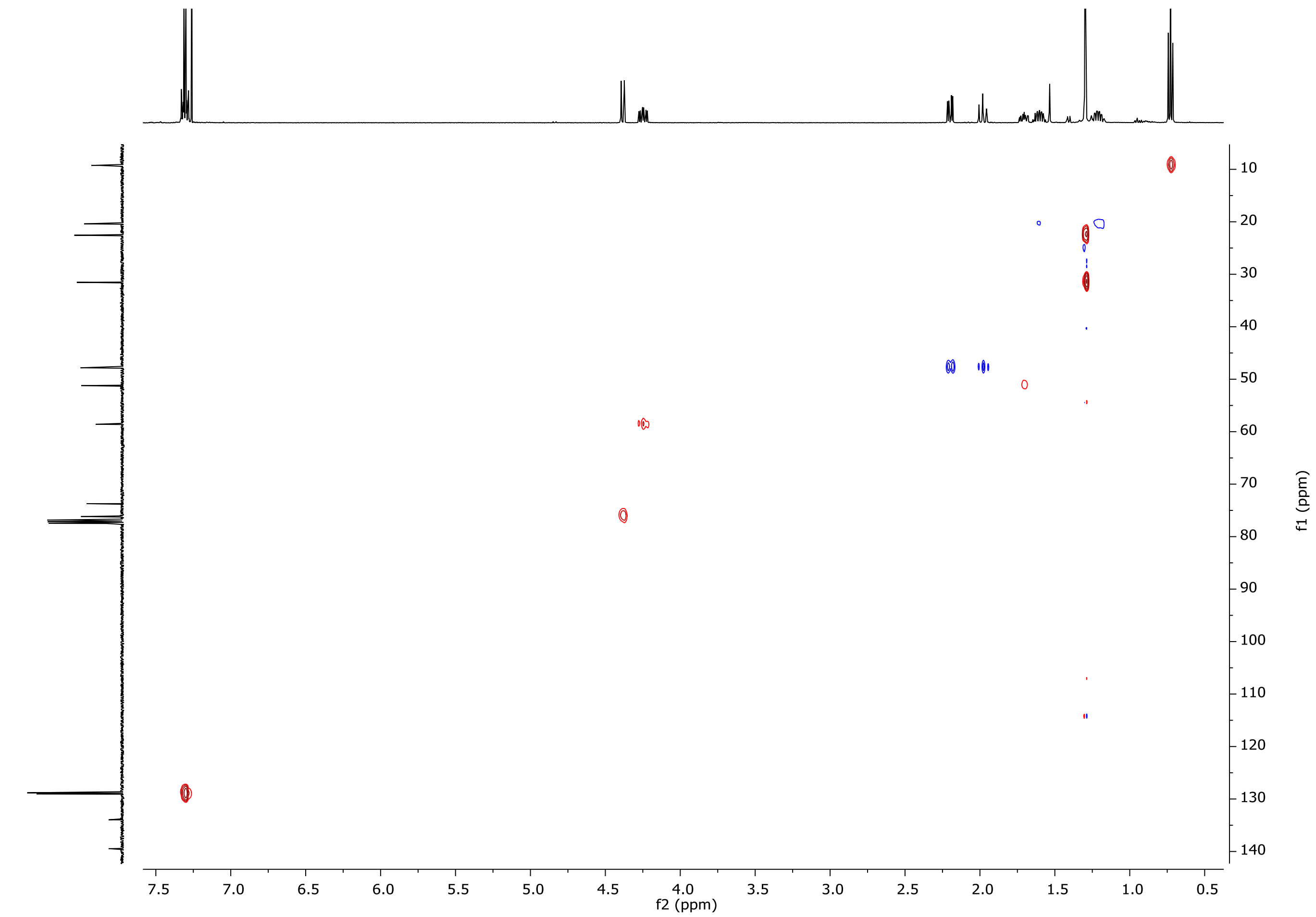

Compound 7e

^{1}H NMR (500 MHz, $CDCl_3$)

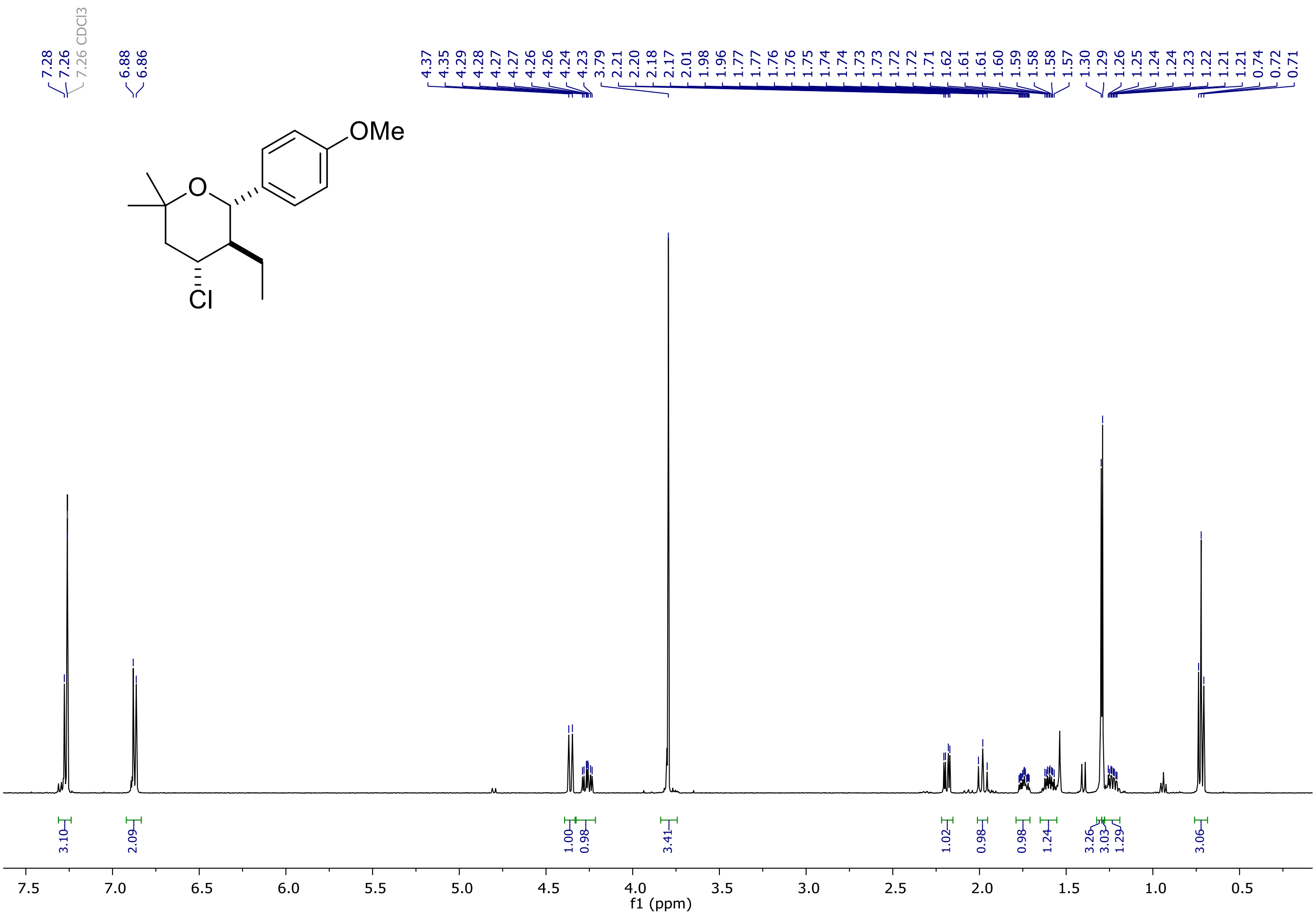

^{13}C NMR (101 MHz, $CDCl_3$)

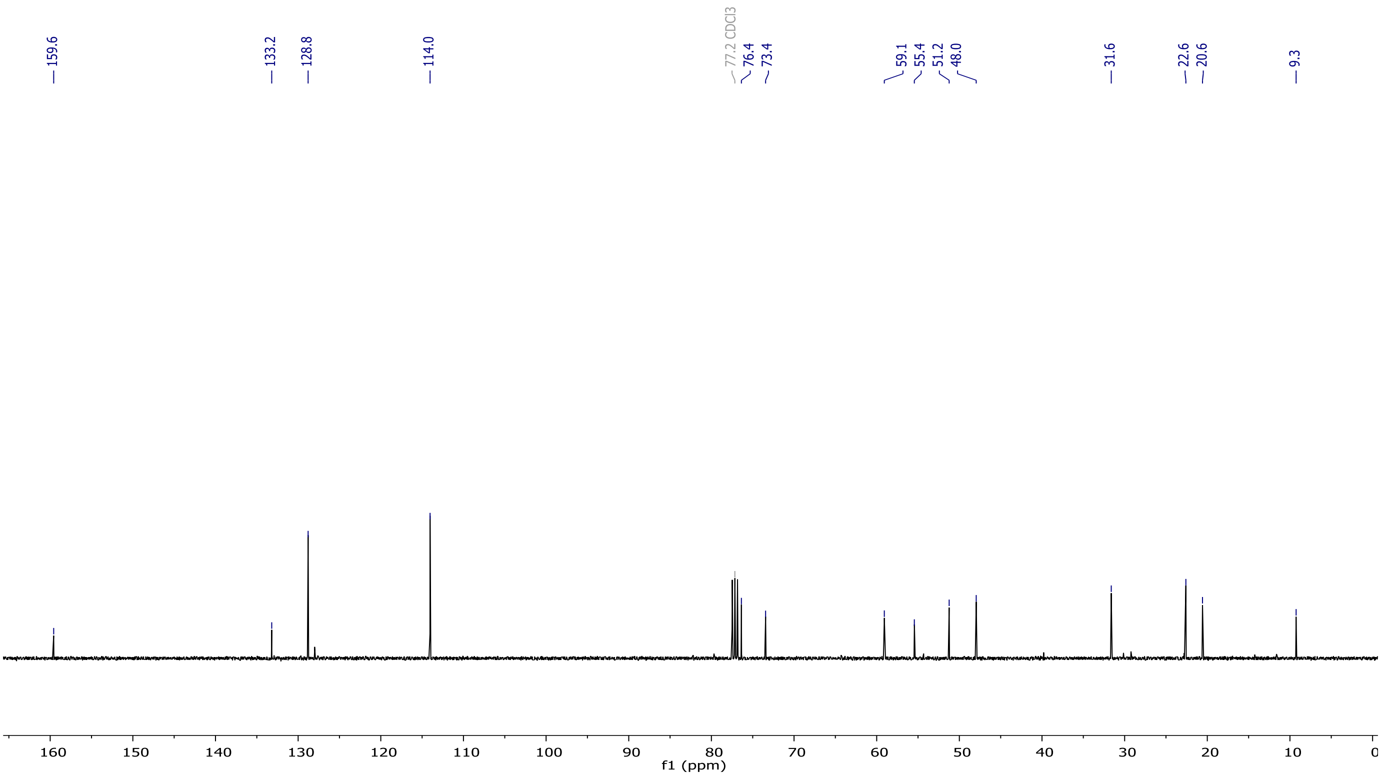

2D-COSY

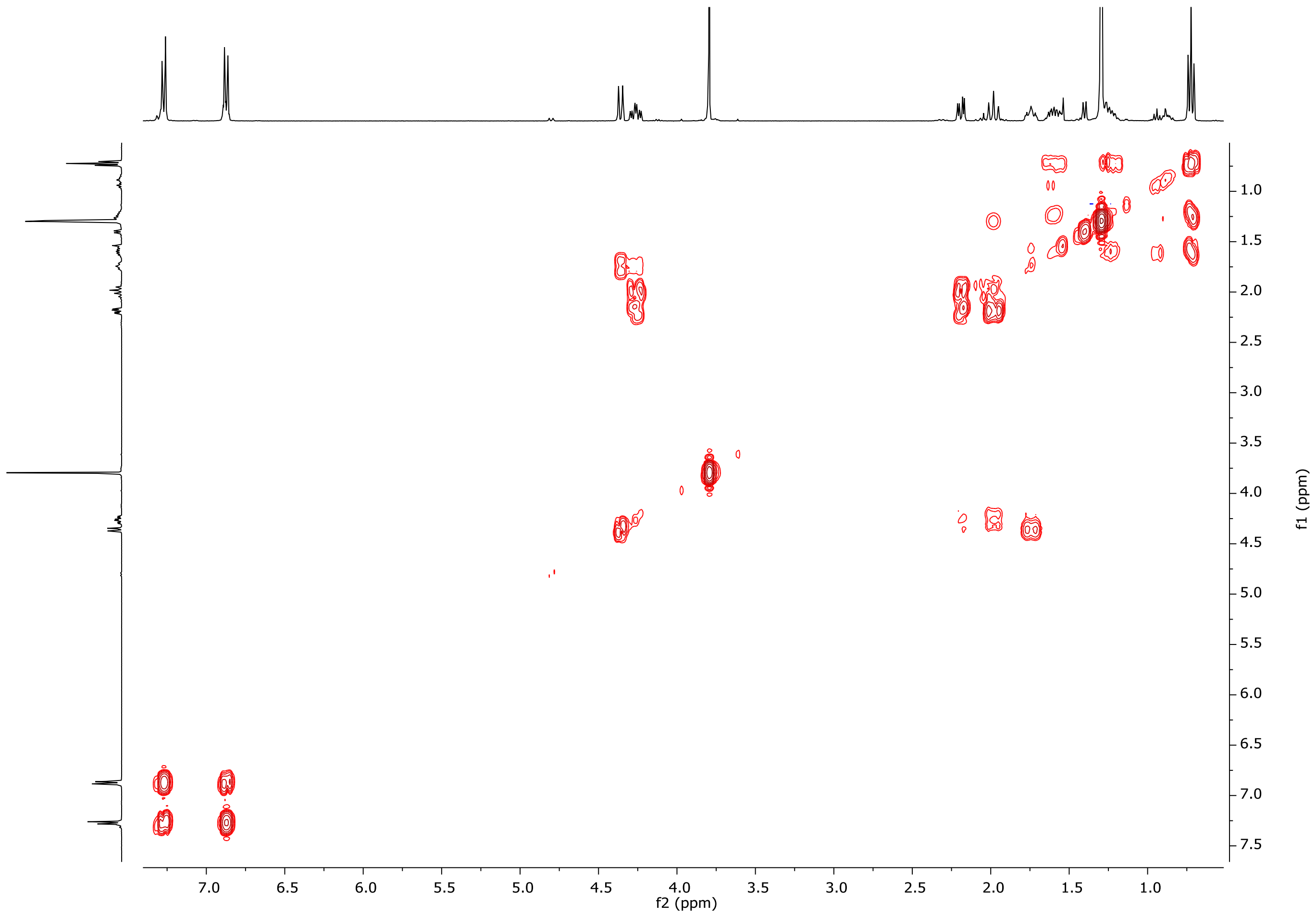

2D-HSQC

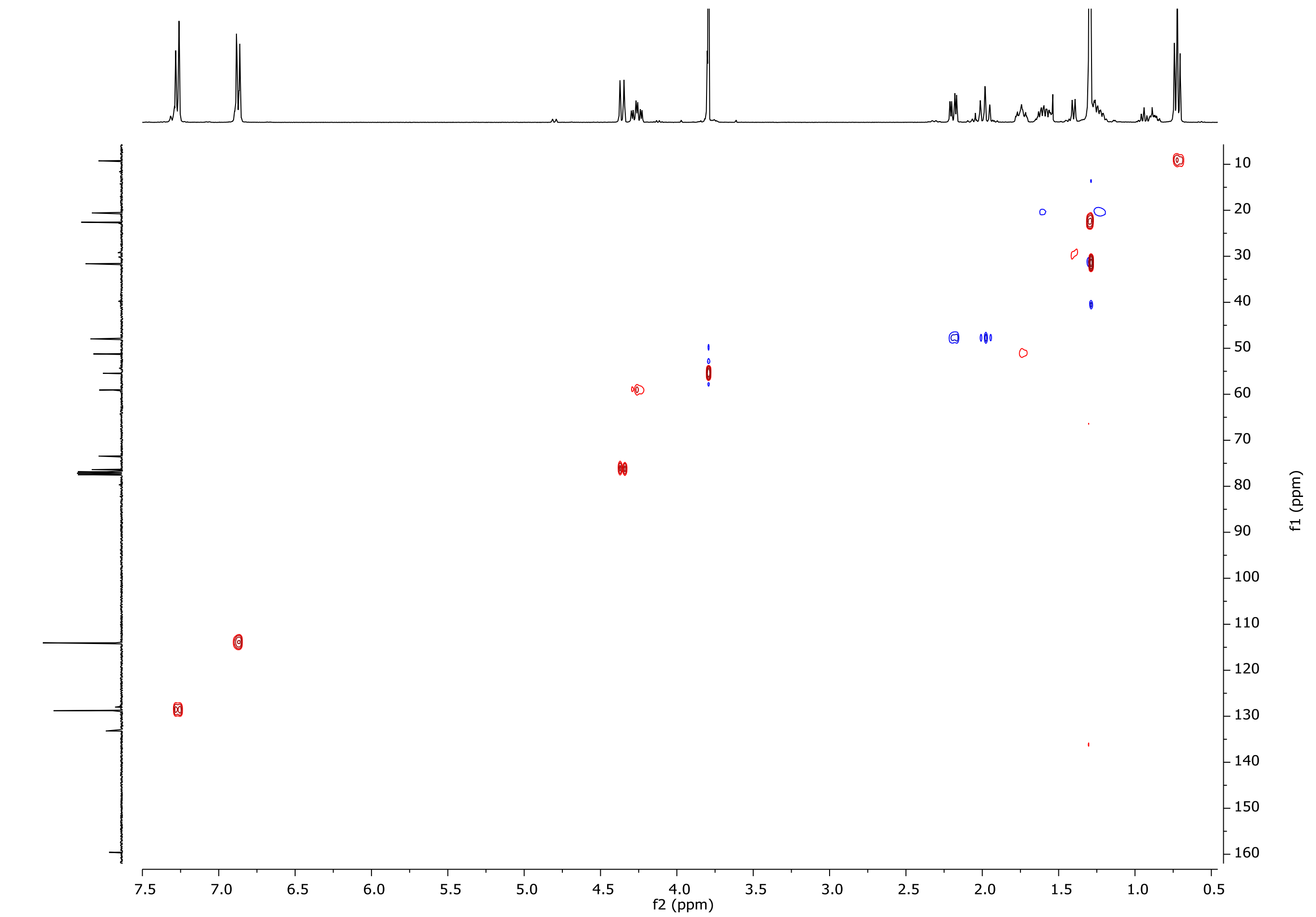

Compound 8a

^{1}**H NMR** (500 MHz, $CDCl_3$)

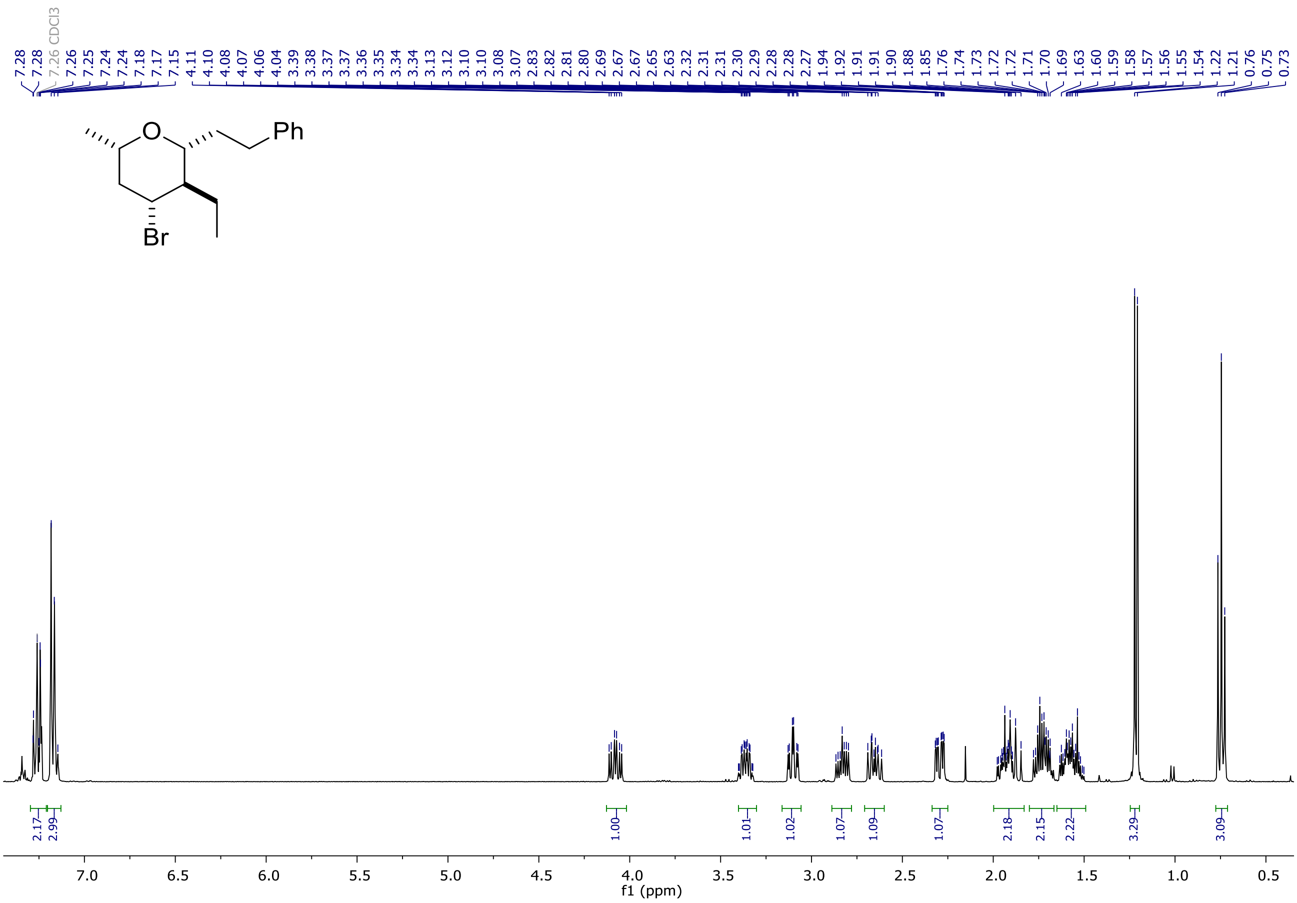

^{13}**C NMR** (101 MHz, $CDCl_3$)

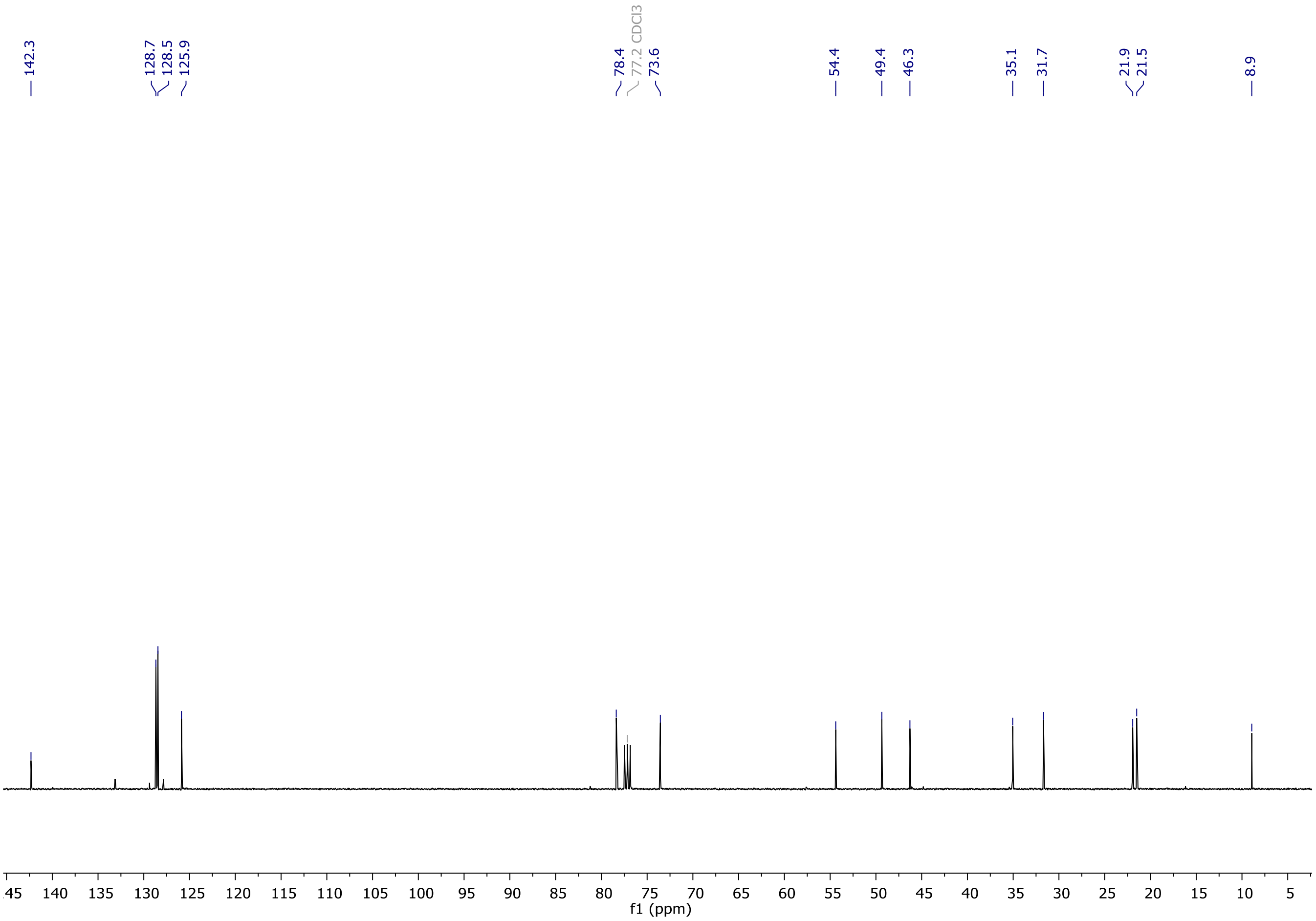

2D-COSY

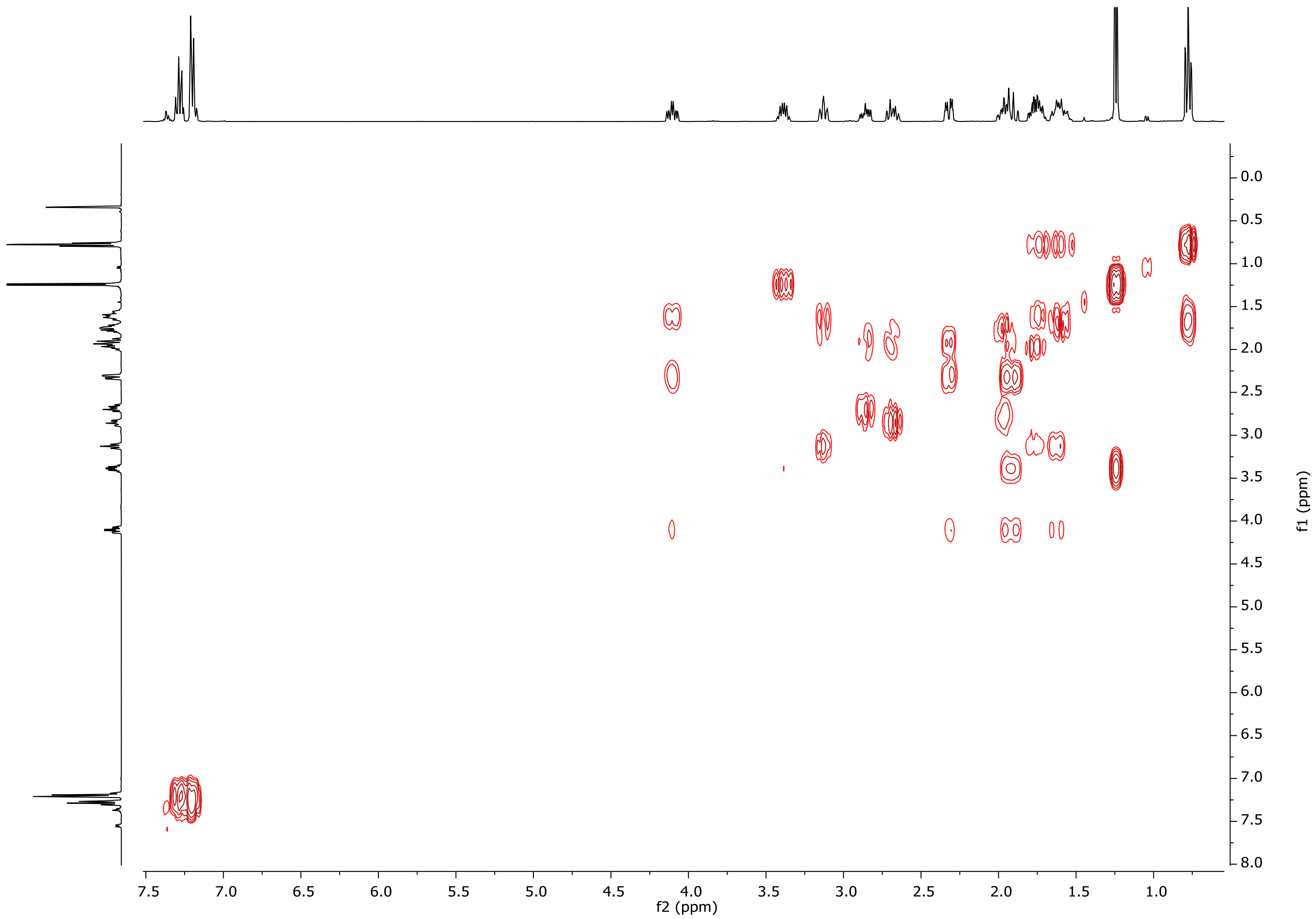

2D-HSQC

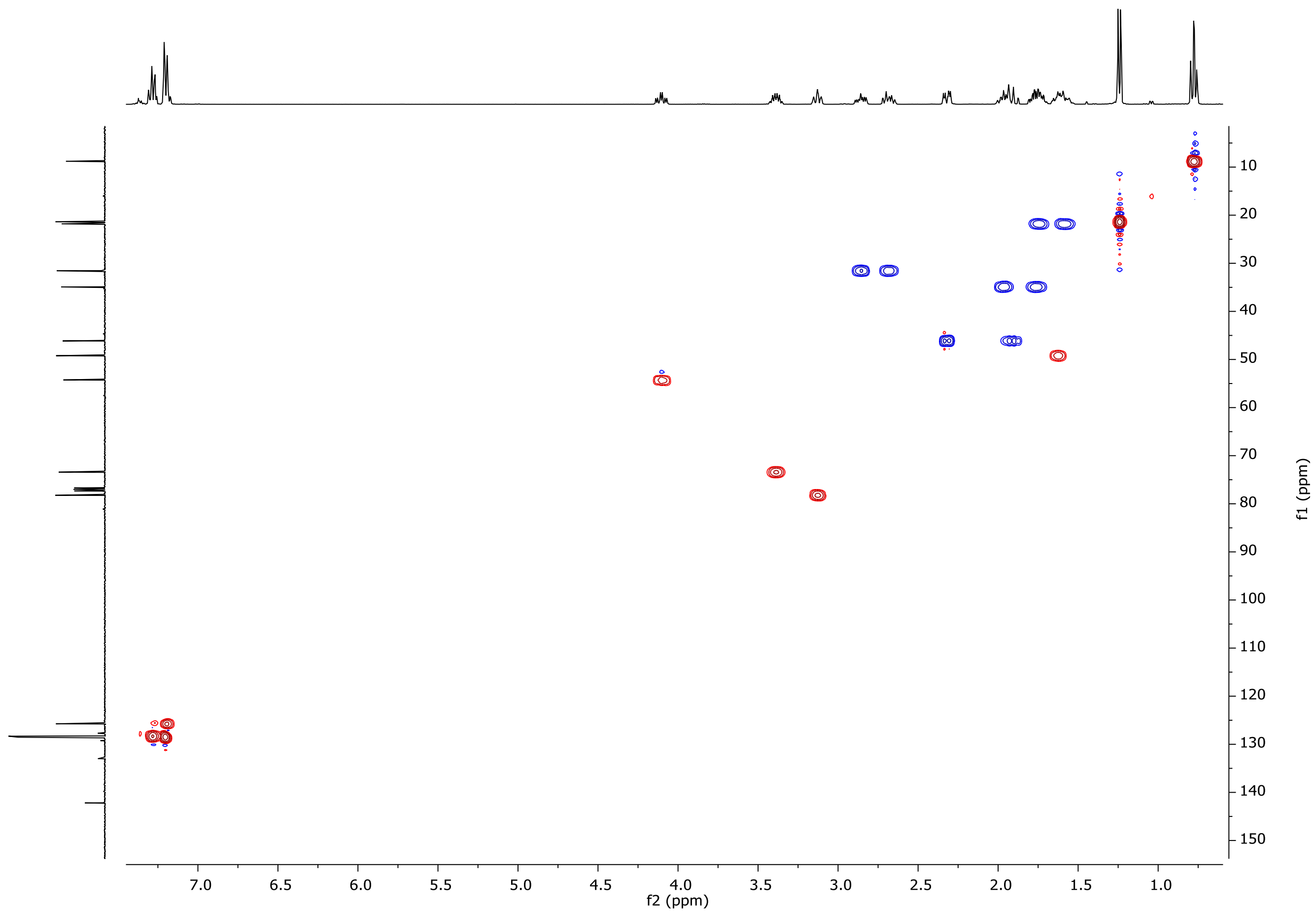

Compound 8b

^{1}H NMR (500 MHz, $CDCl_3$)

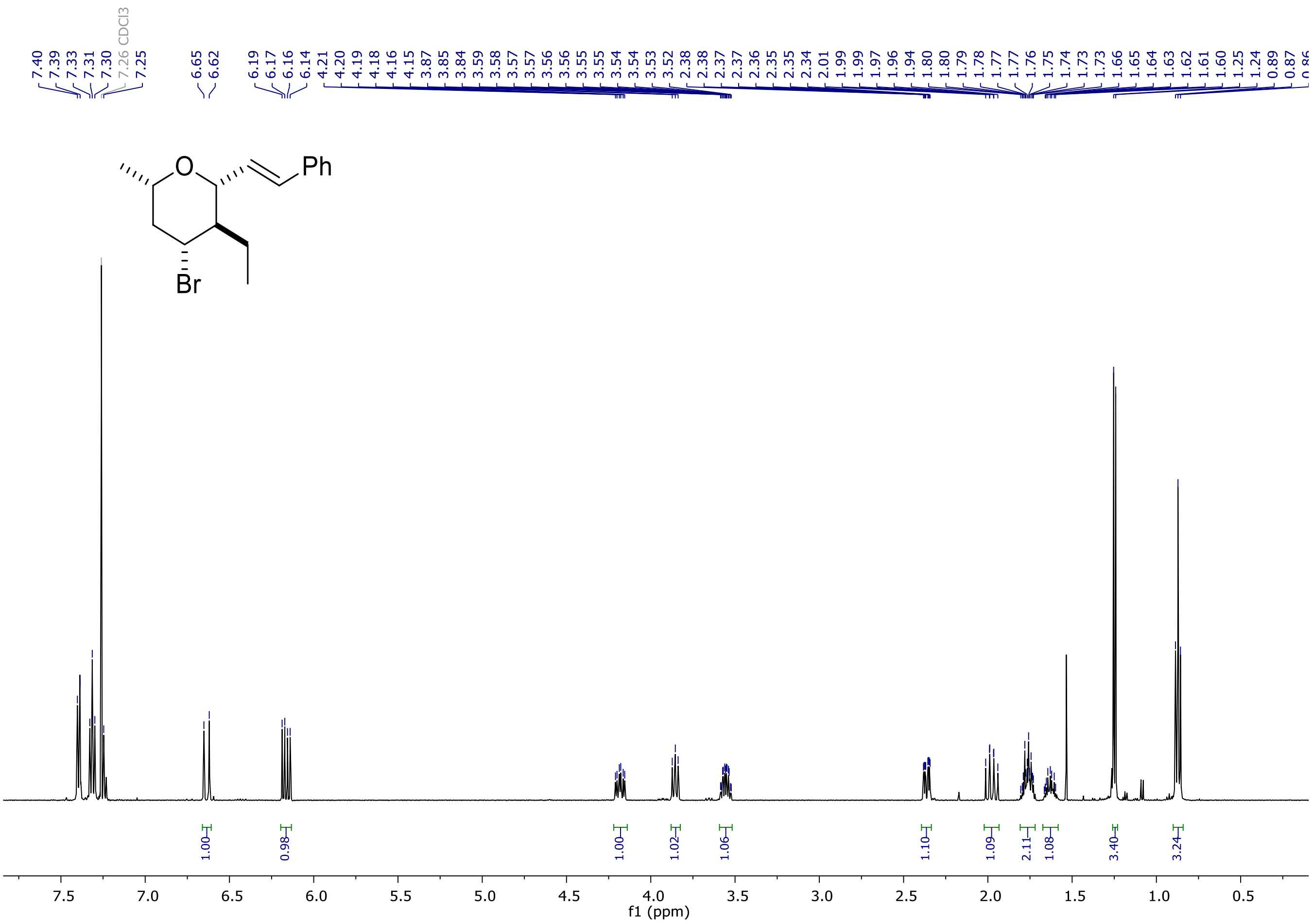

^{13}C NMR (101 MHz, $CDCl_3$)

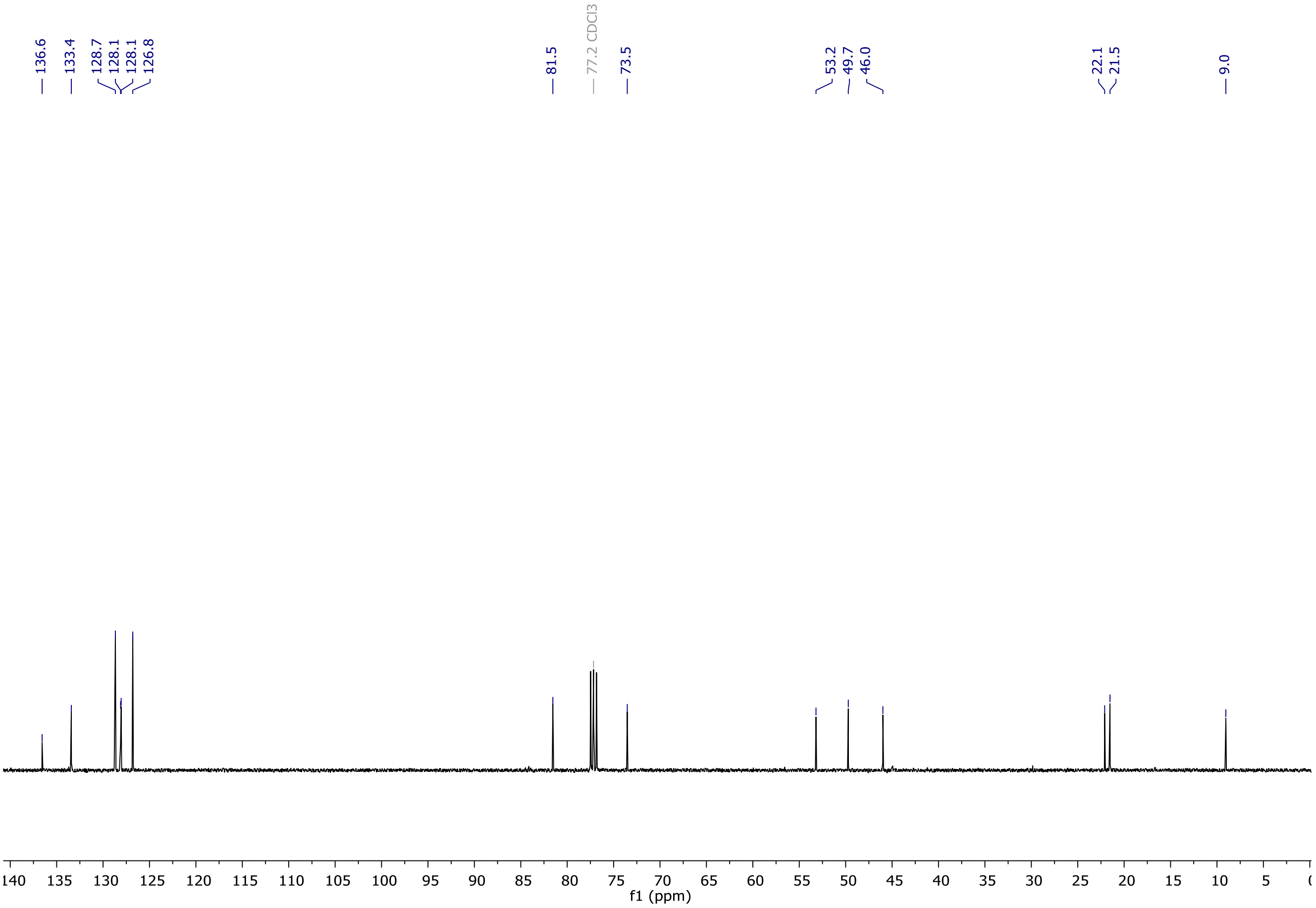

2D-COSY

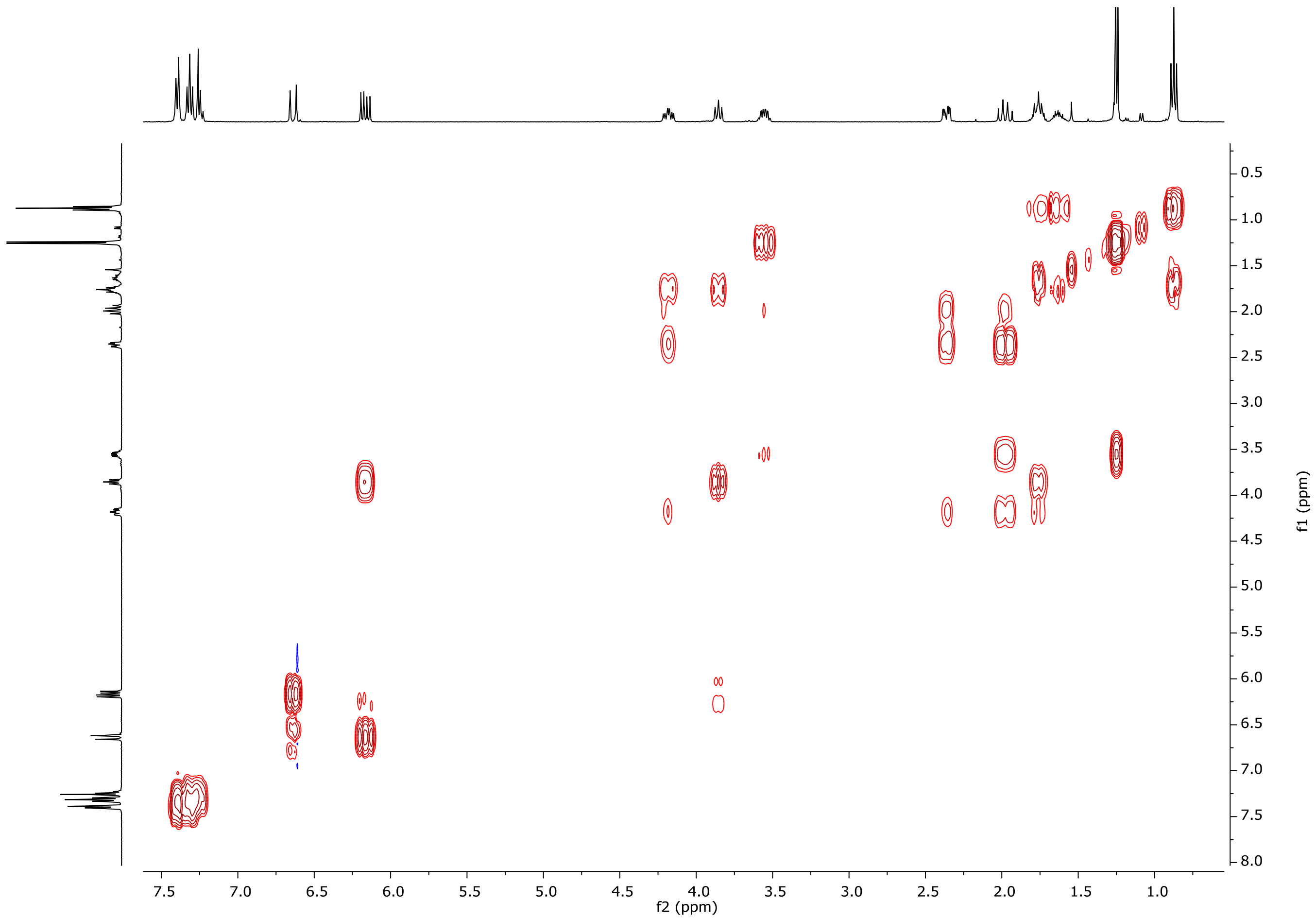

2D-HSQC

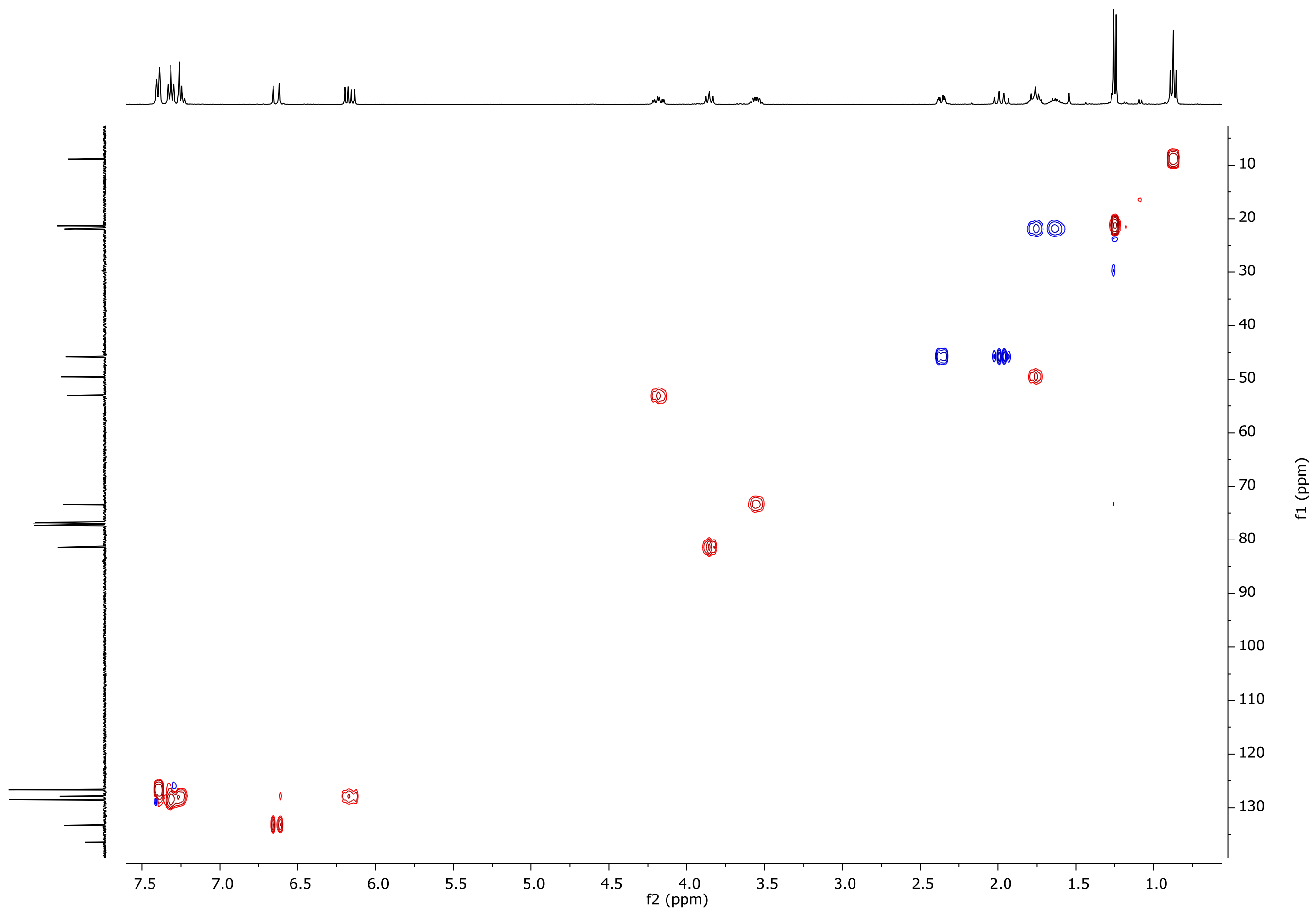

Compound 8c

^{1}H NMR (500 MHz, $CDCl_3$)

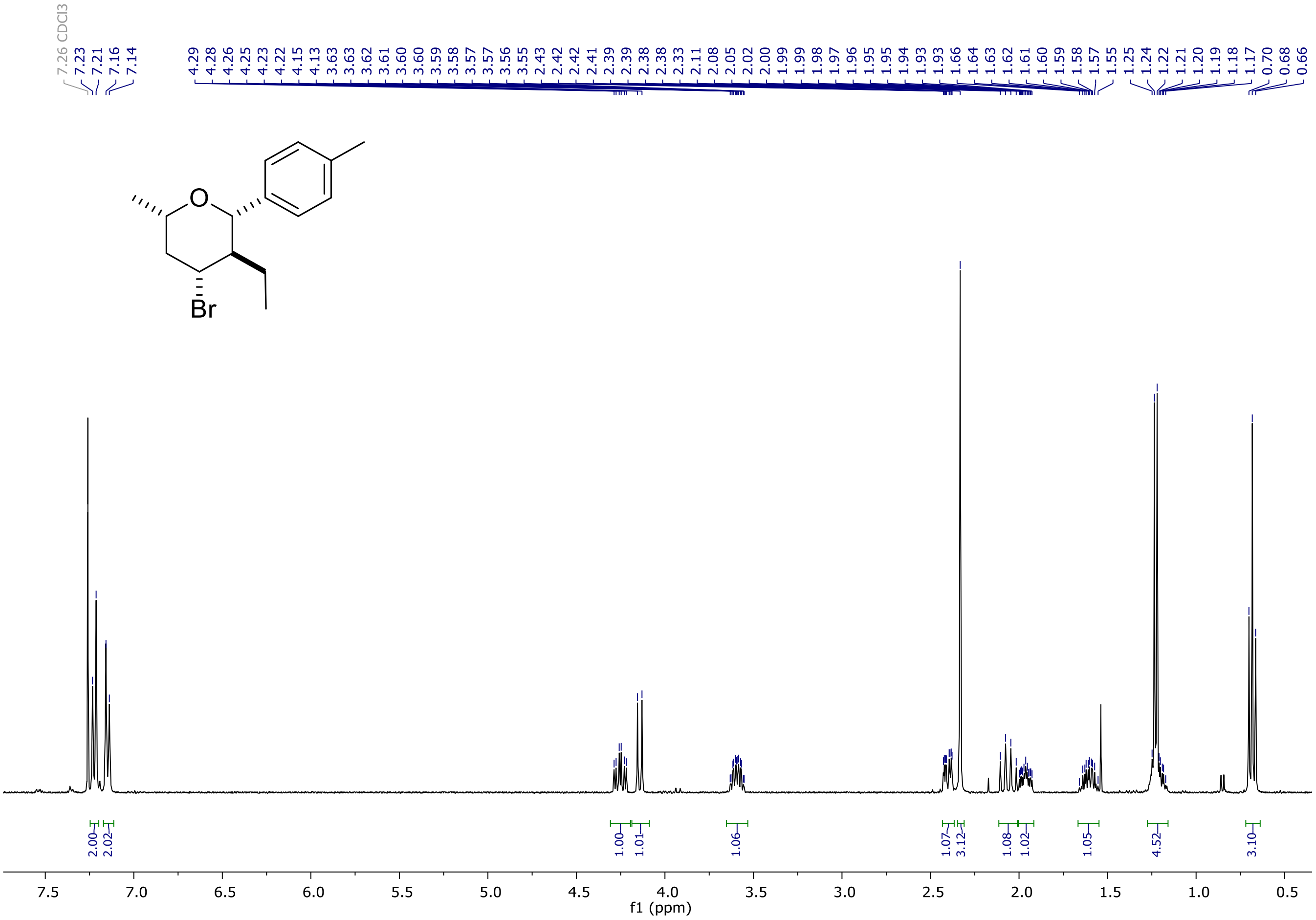

^{13}C NMR (101 MHz, $CDCl_3$)

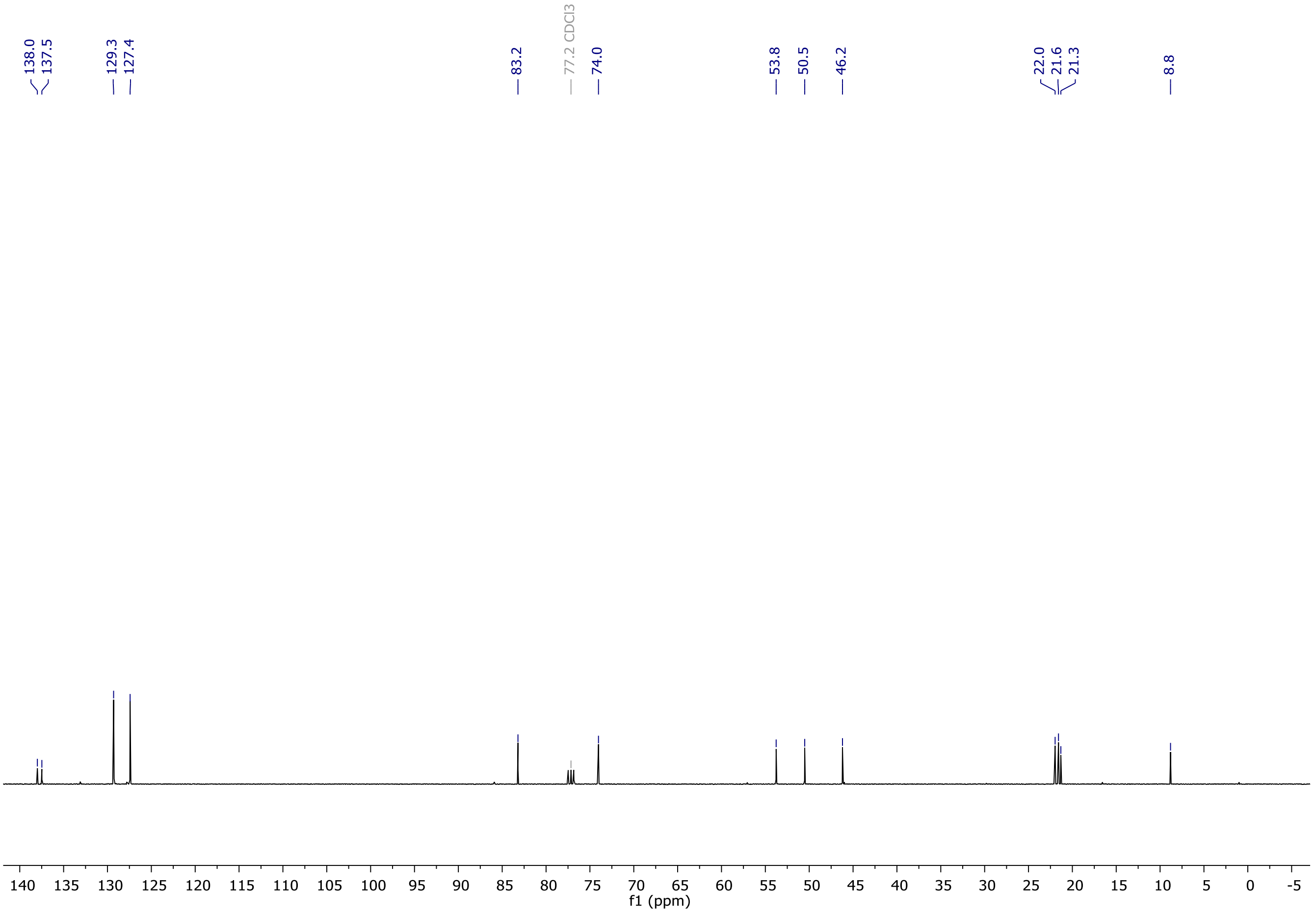

2D-COSY

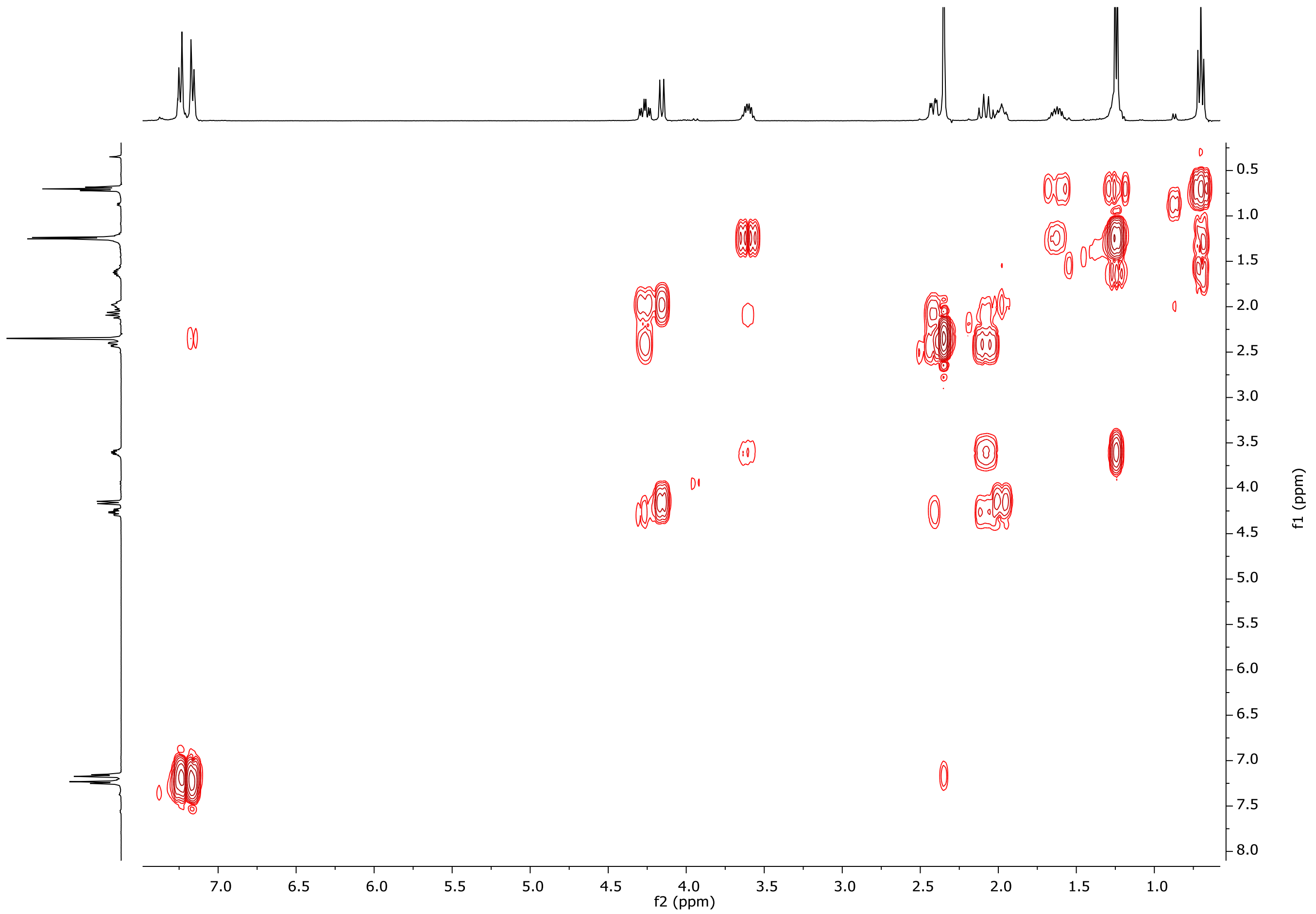

2D-HSQC

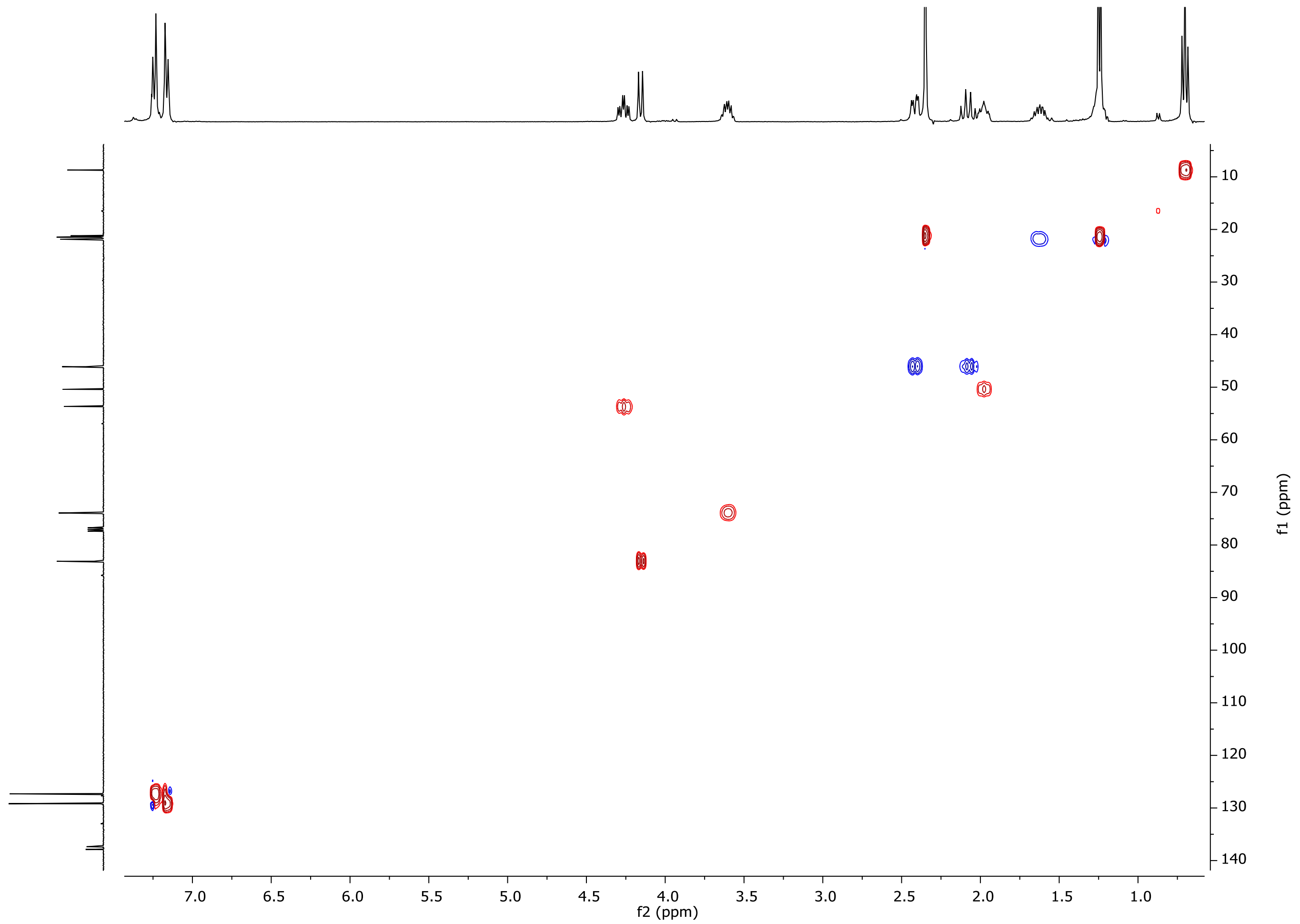

Compound 8d

¹H NMR (500 MHz, $CDCl_3$)

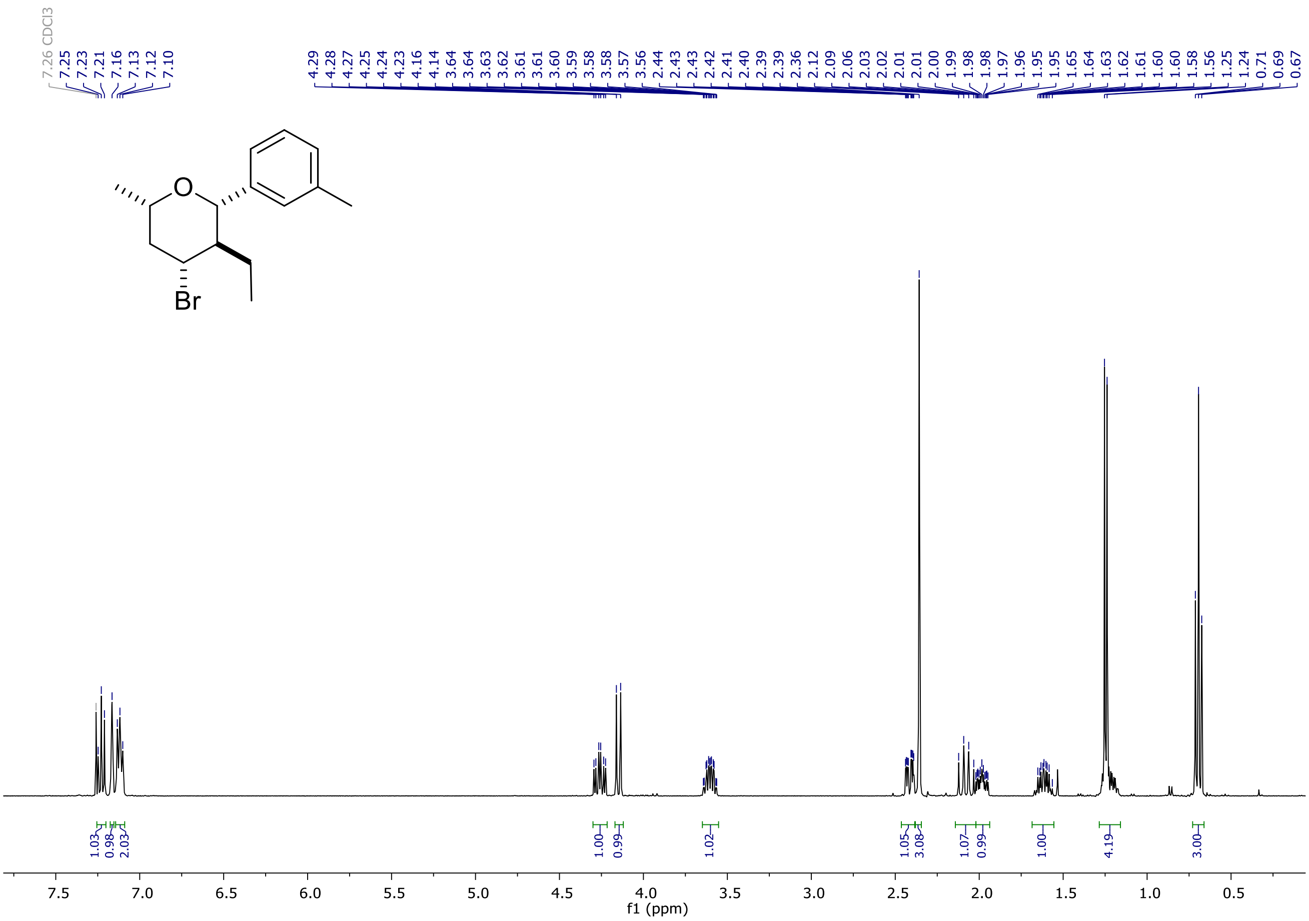

¹³C NMR (101 MHz, $CDCl_3$)

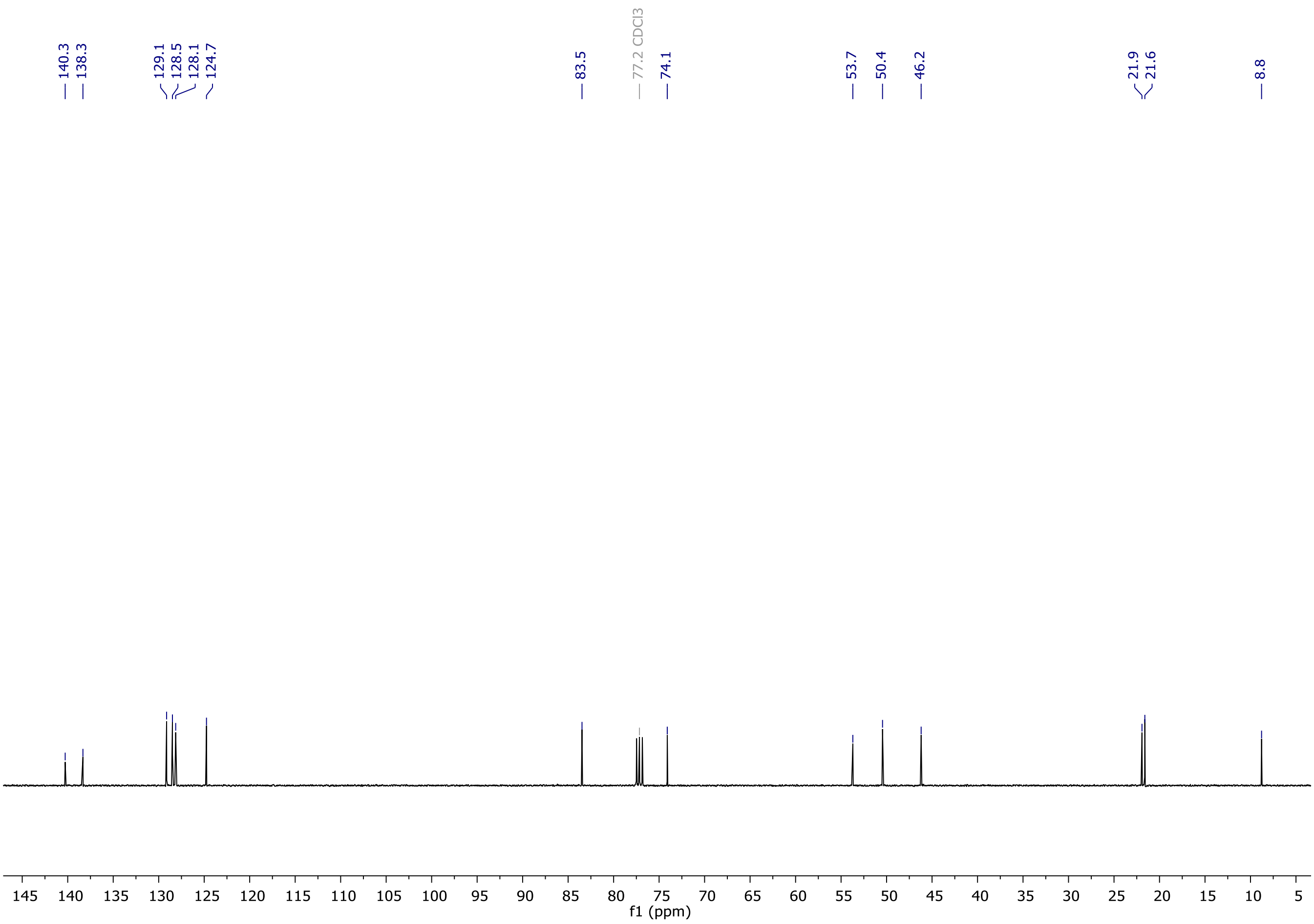

2D-COSY

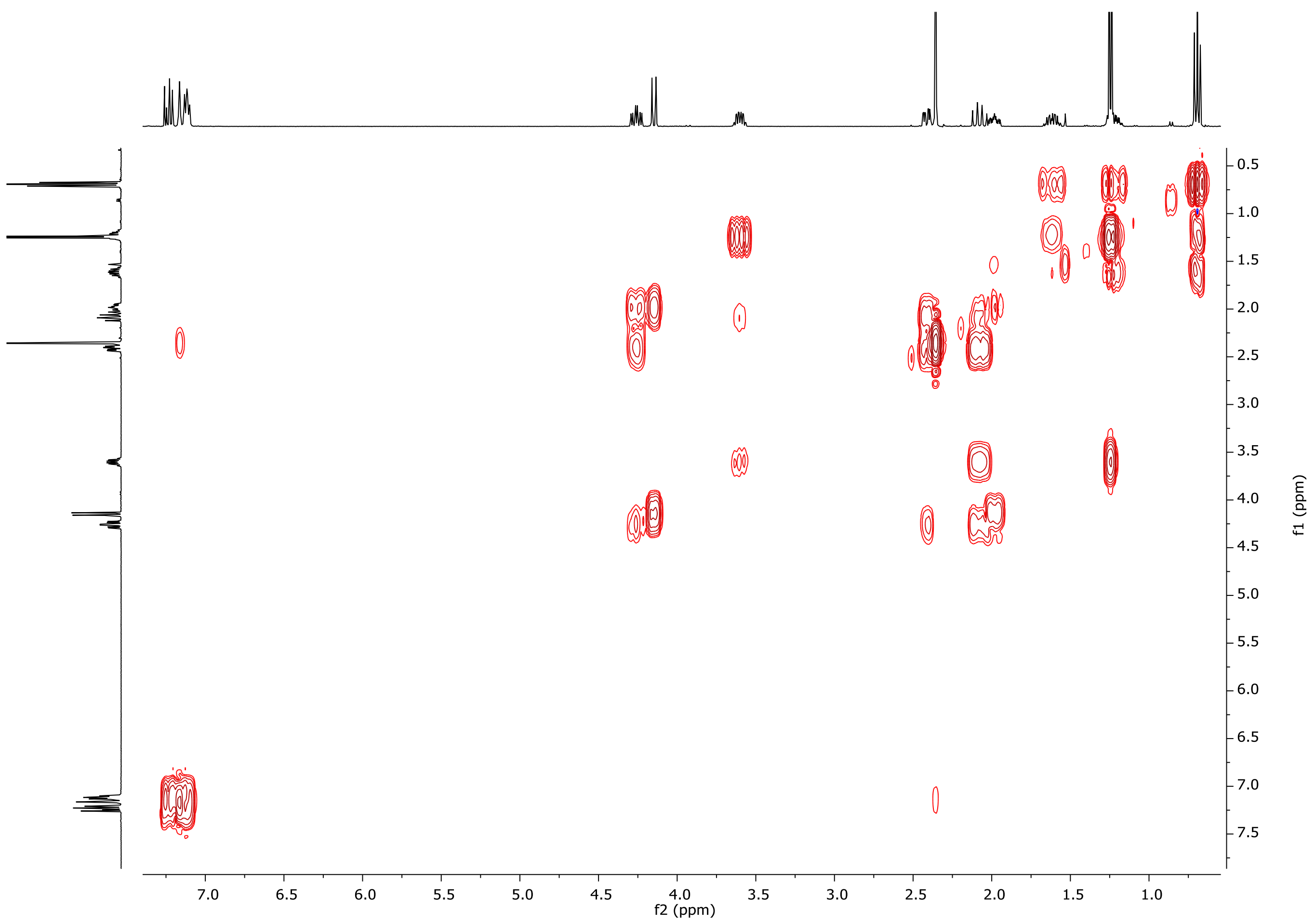

2D-HSQC

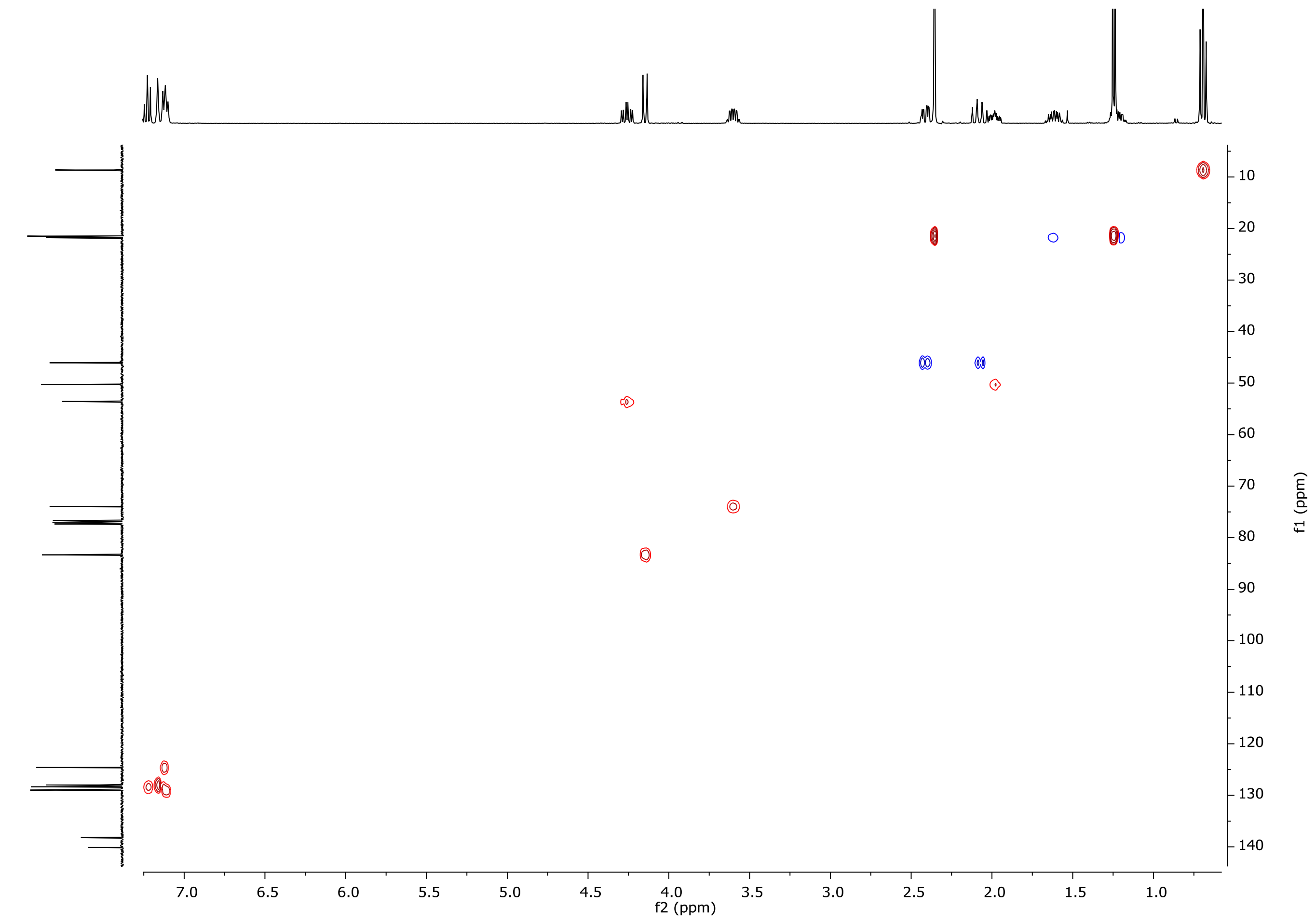

Compound 8e

^{1}H NMR (500 MHz, $CDCl_3$)

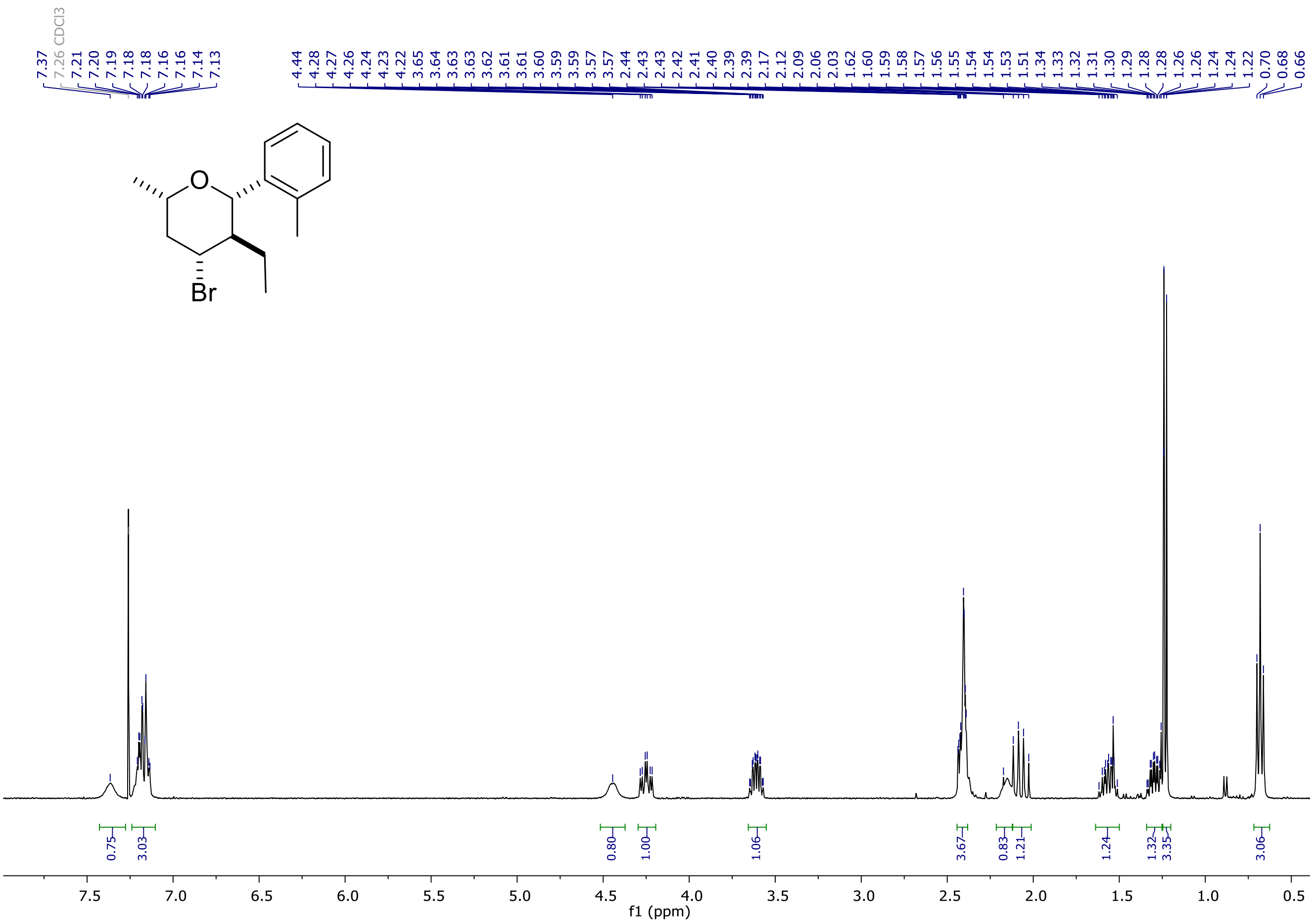

^{13}C NMR (101 MHz, $CDCl_3$)

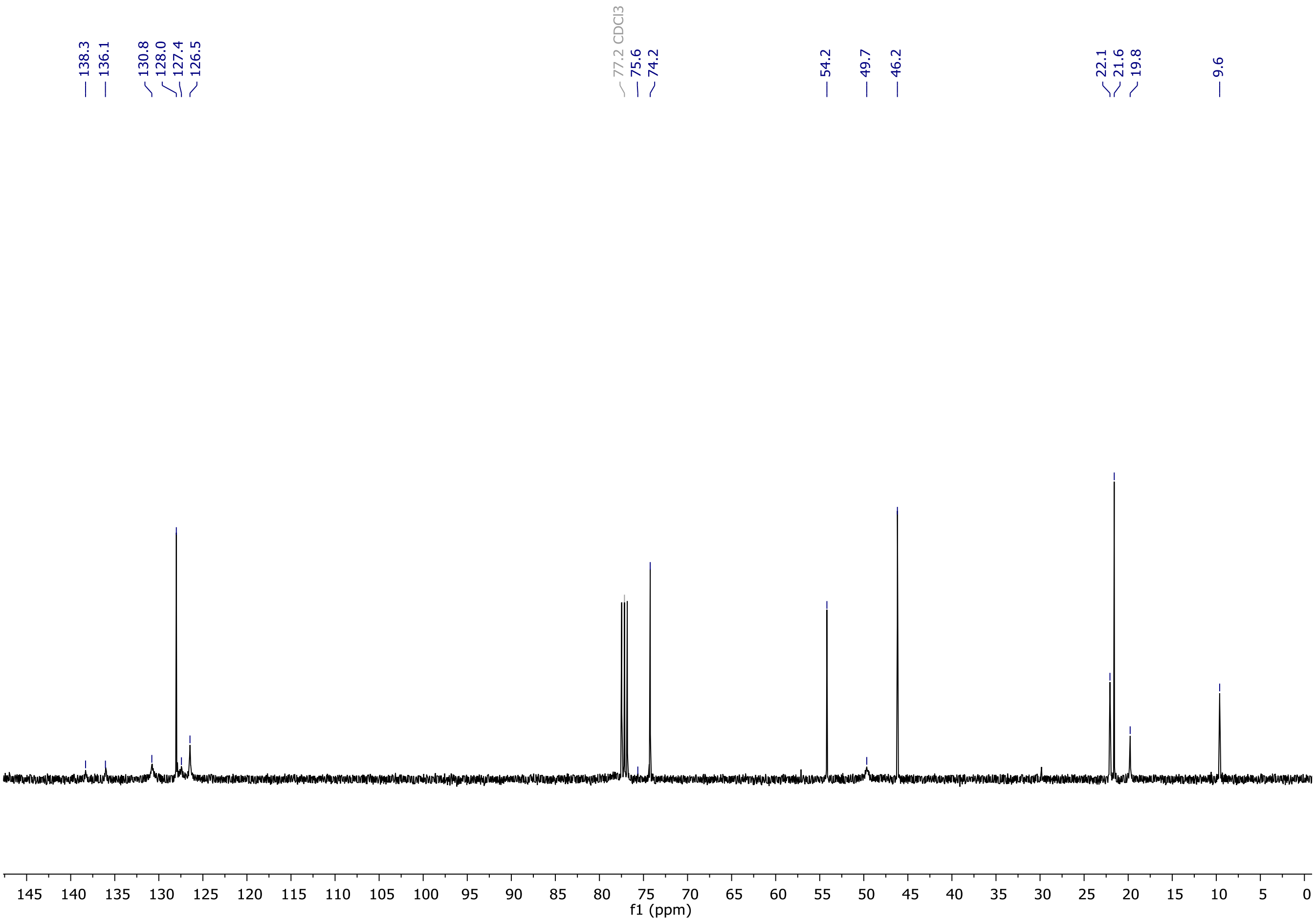

2D-COSY

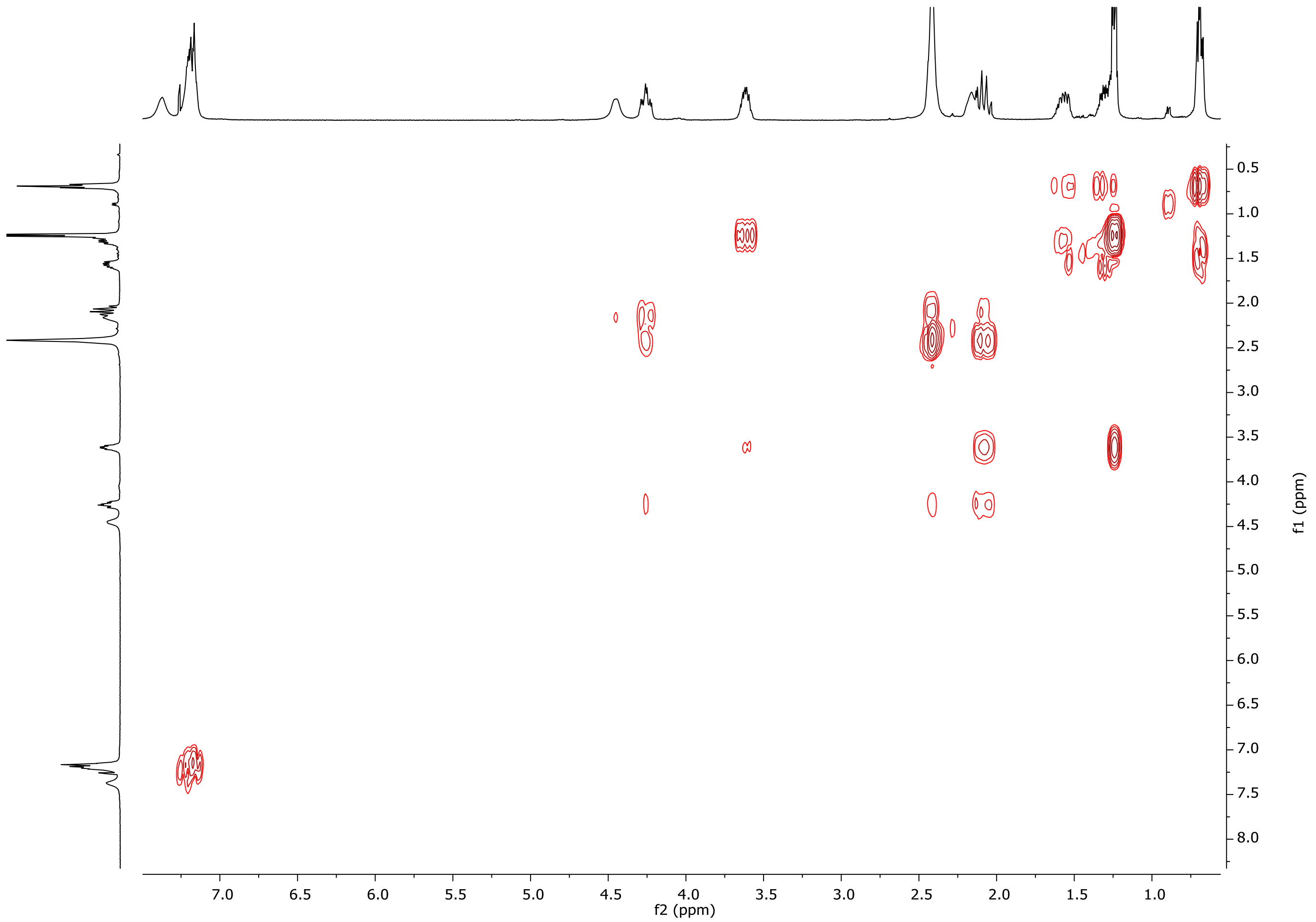

2D-HSQC

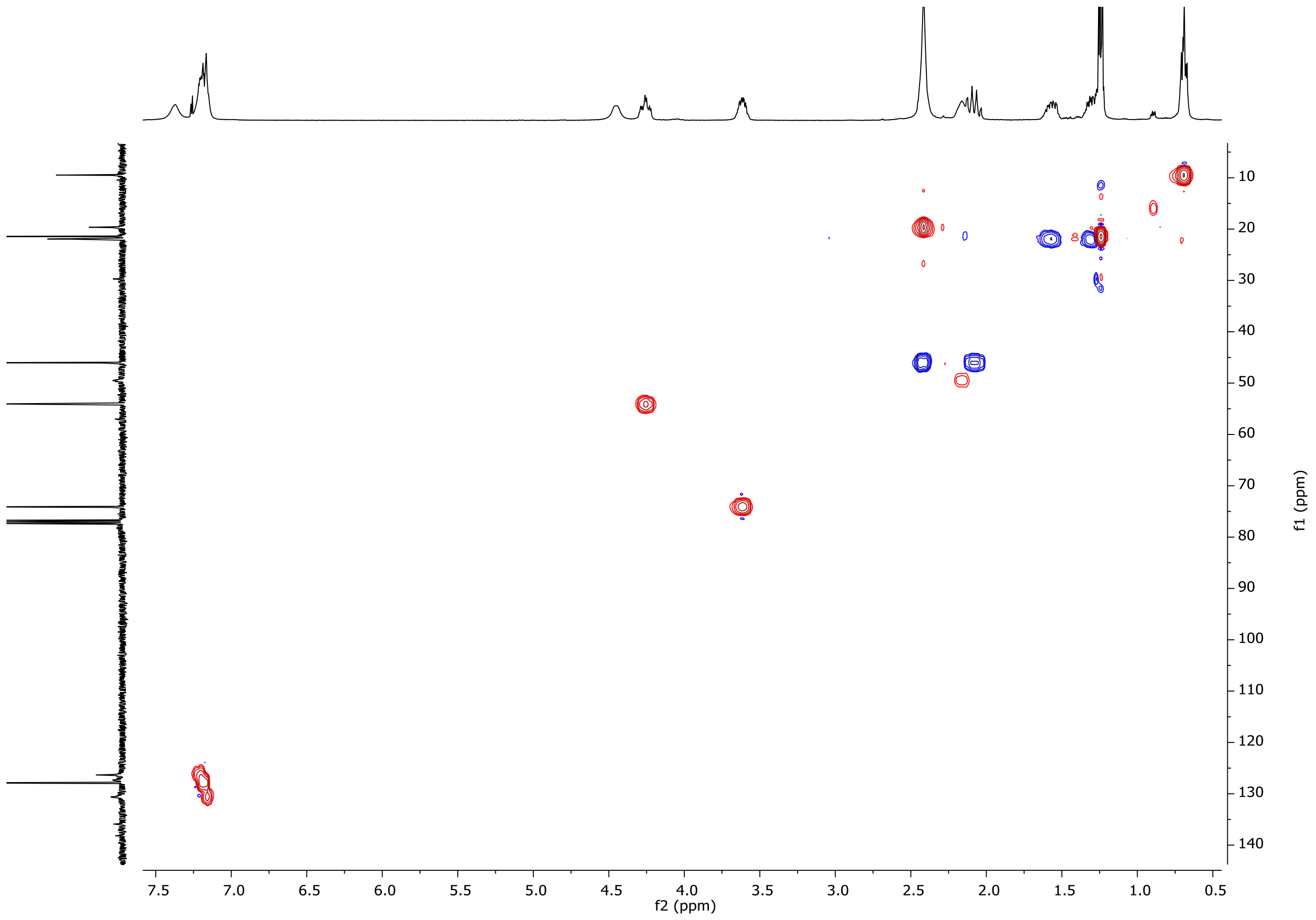

Compound 8f

^{1}H NMR (500 MHz, CDCl$_3$)

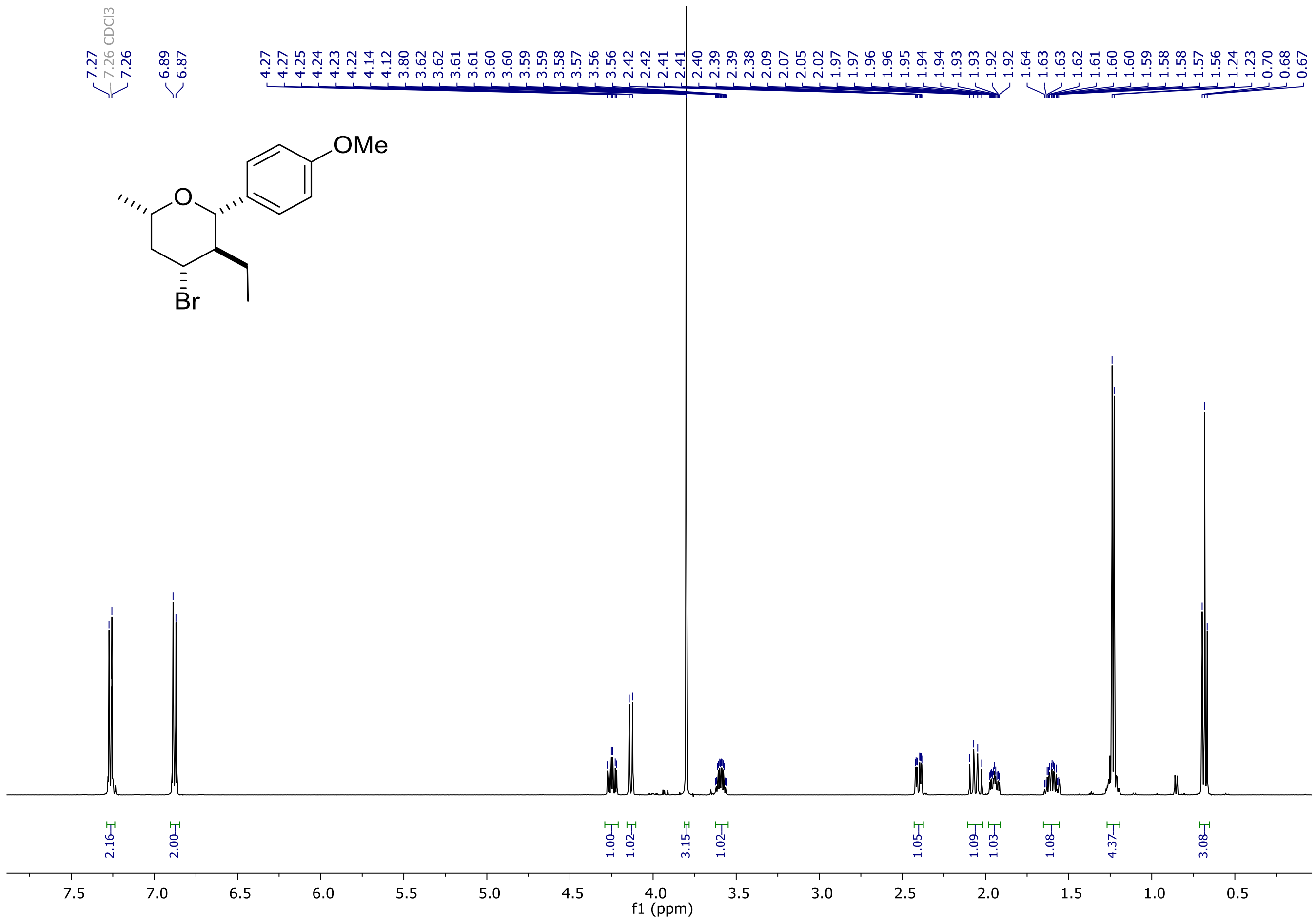

^{13}C NMR (101 MHz, CDCl$_3$)

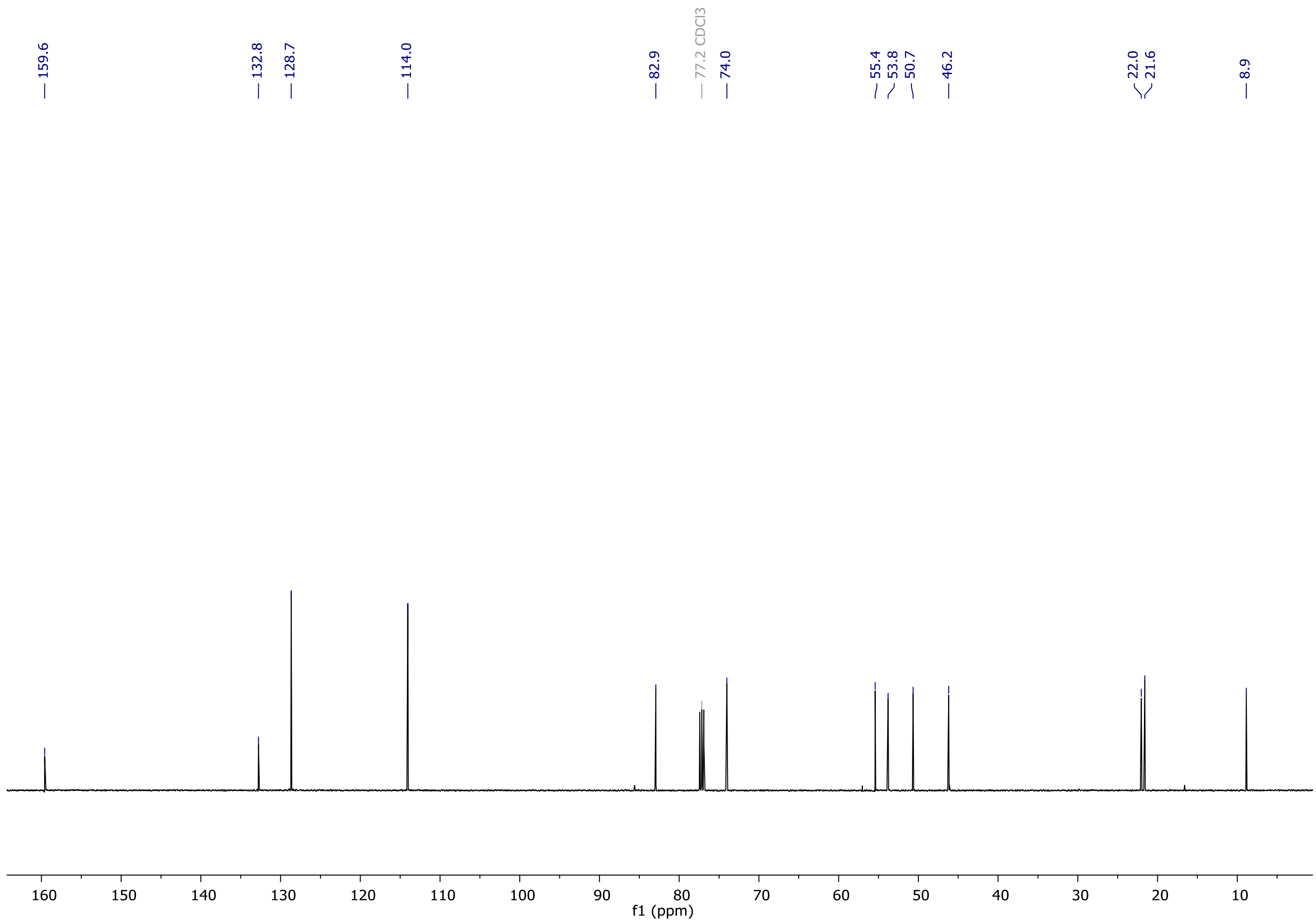

2D-COSY

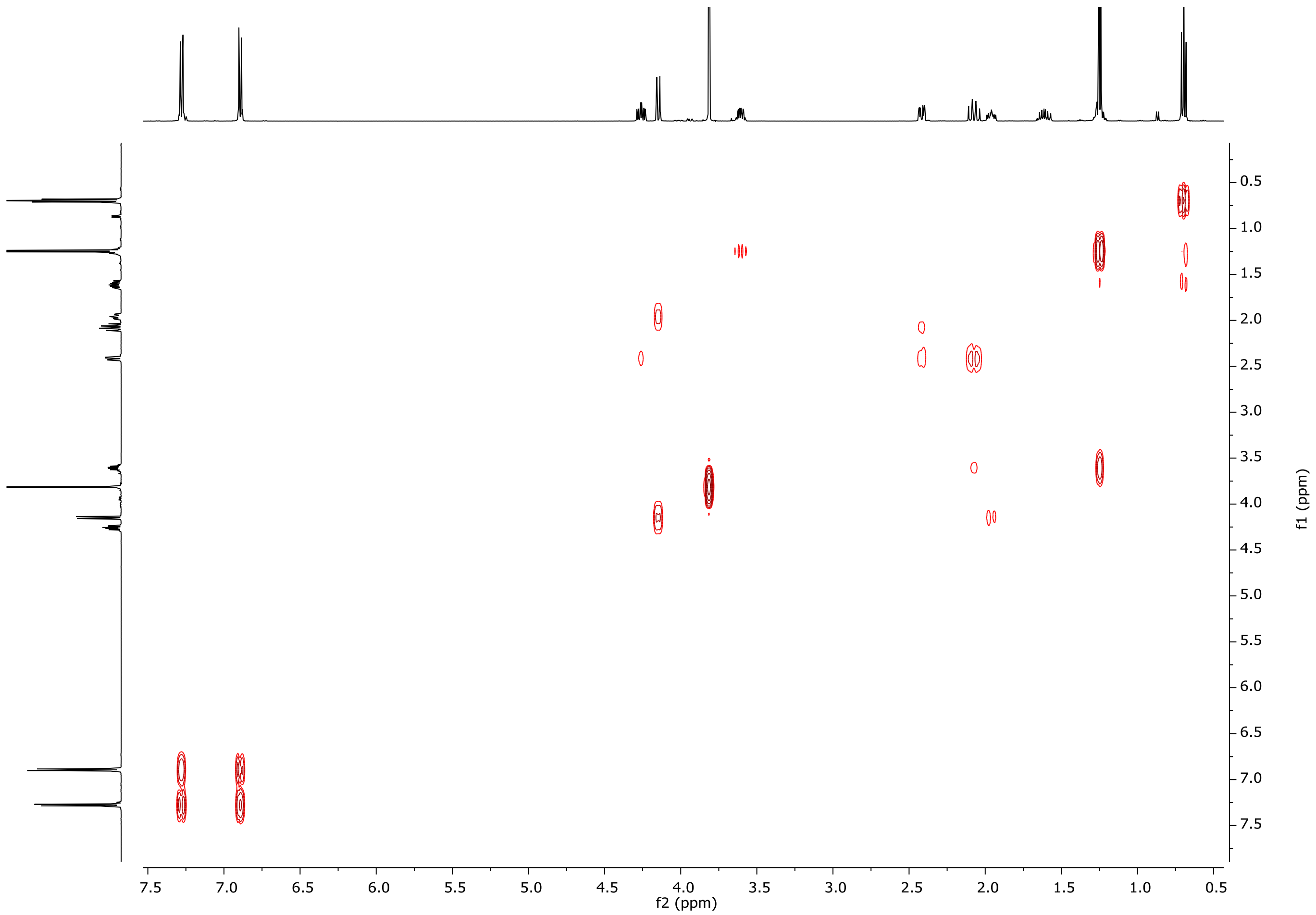

2D-HSQC

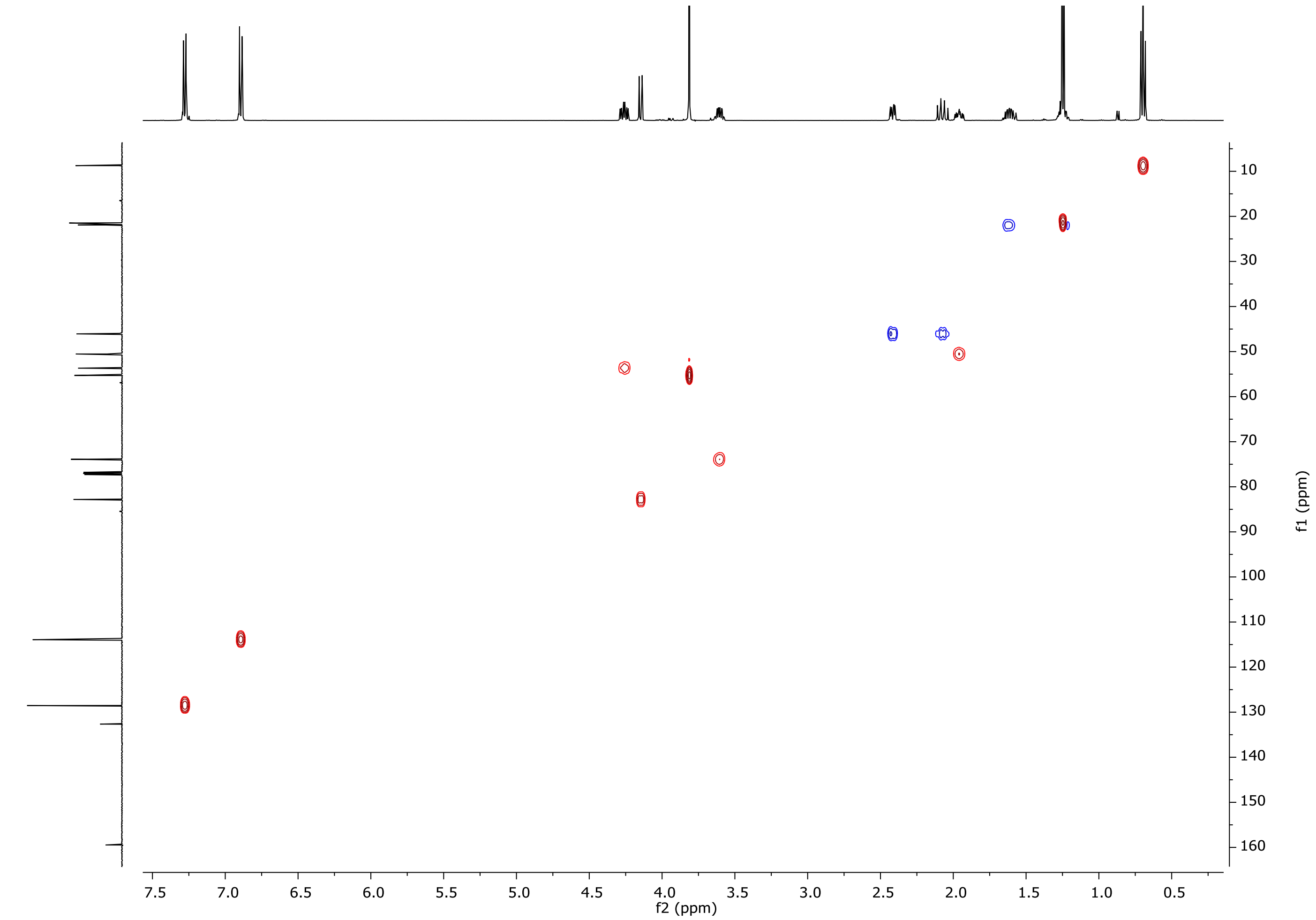

Compound 8g

^{1}H NMR (500 MHz, $CDCl_3$)

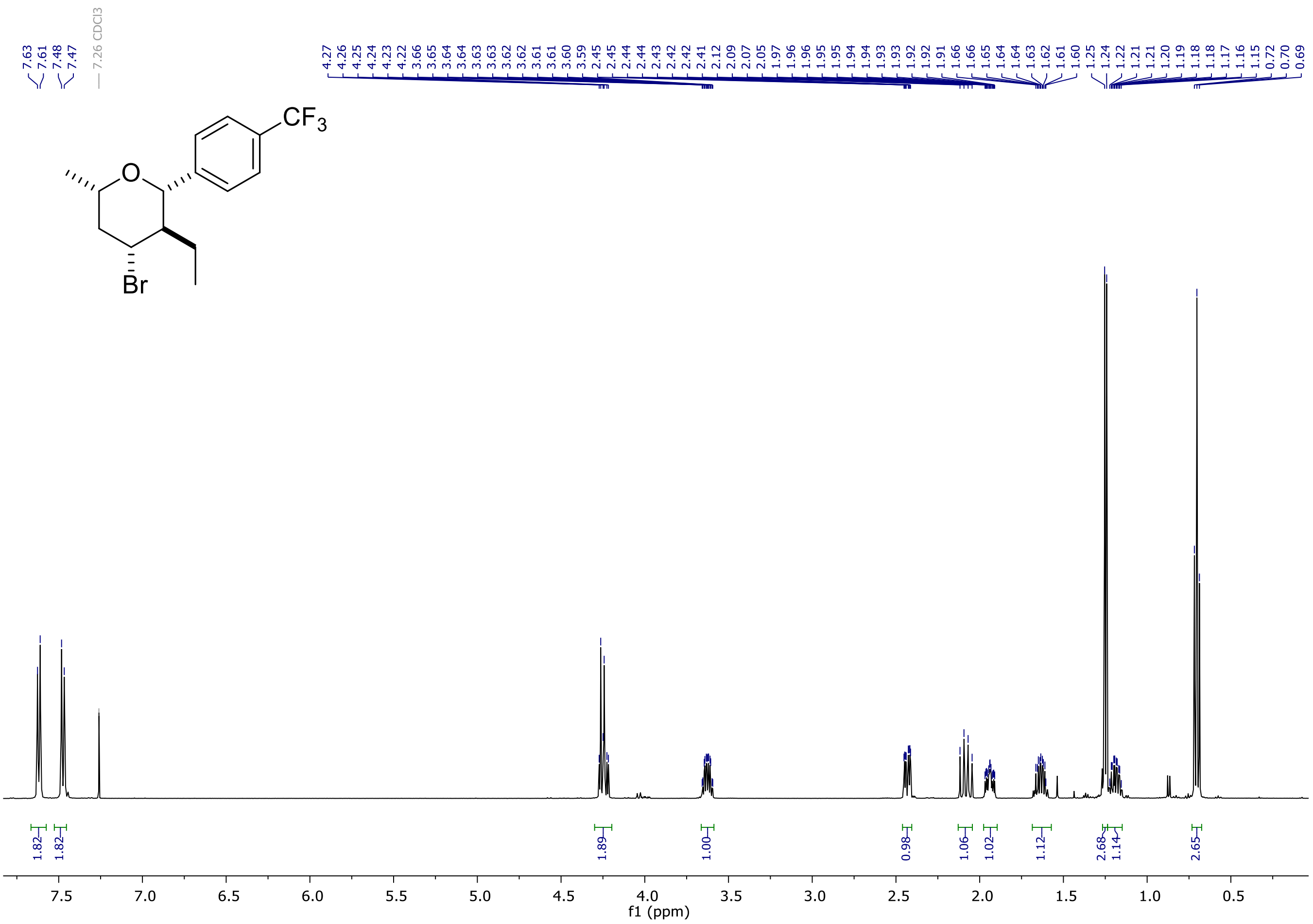

^{13}C NMR (101 MHz, $CDCl_3$)

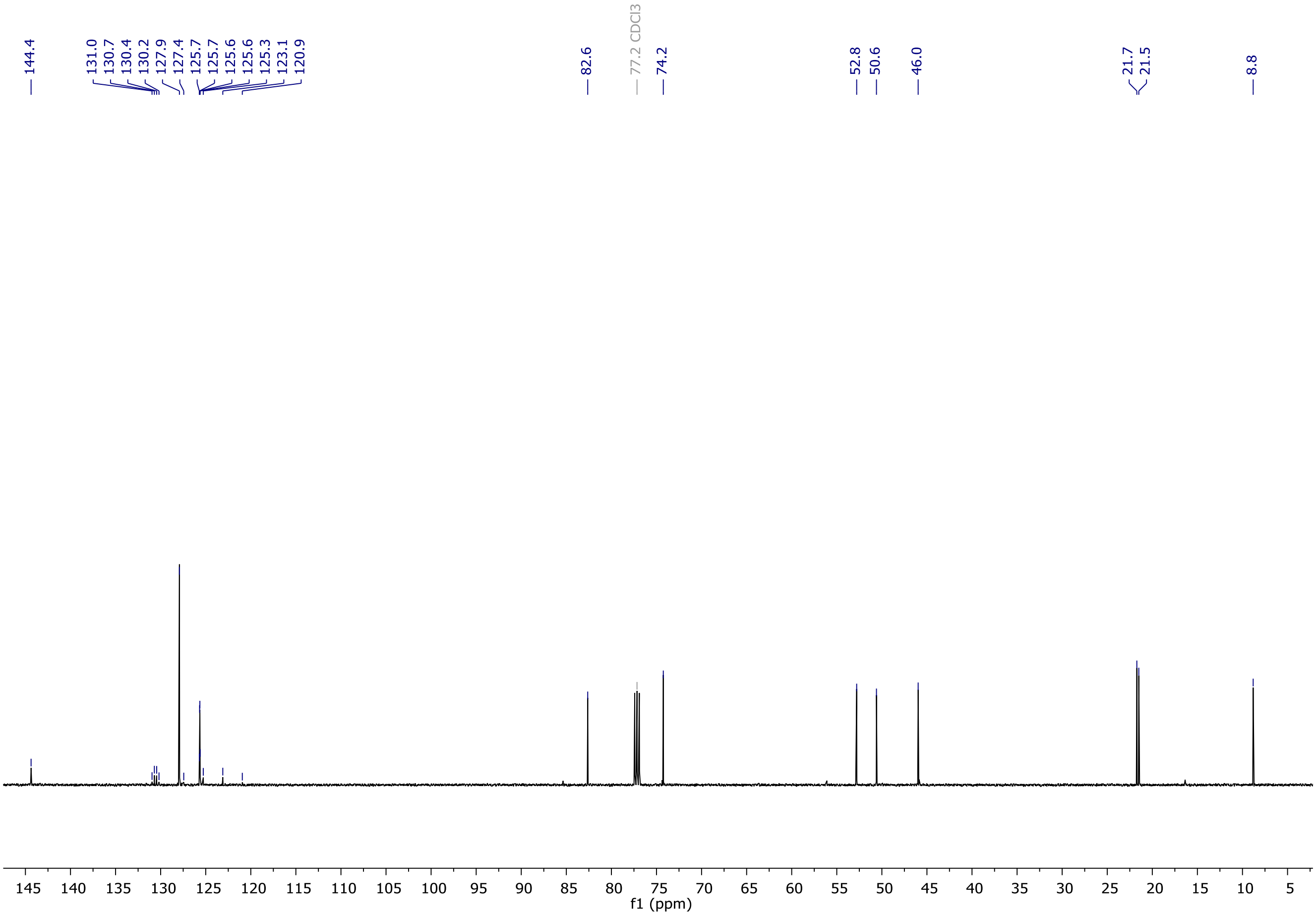

2D-COSY

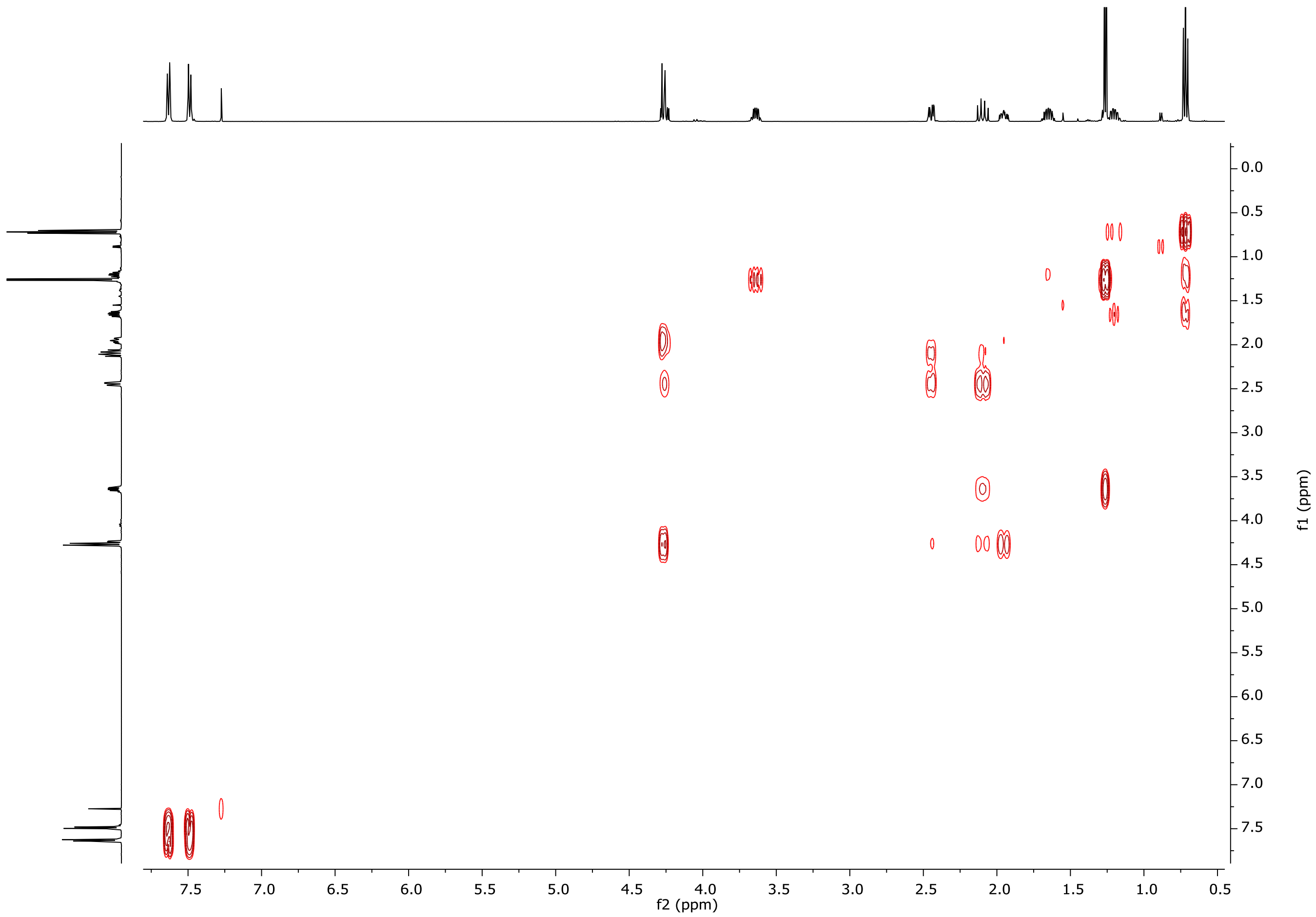

2D-HSQC

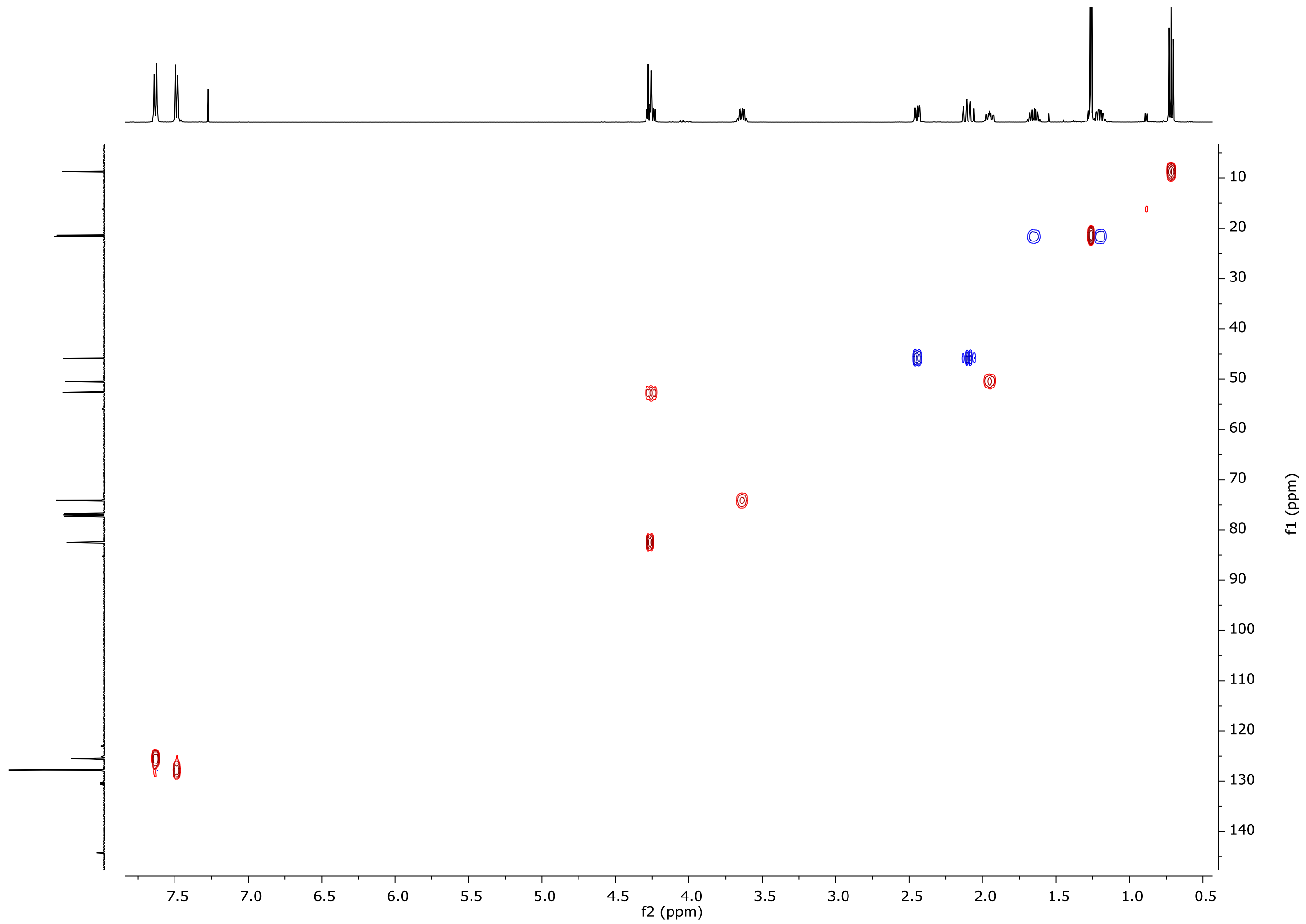

Compound 9a

^{1}H NMR (500 MHz, $CDCl_3$)

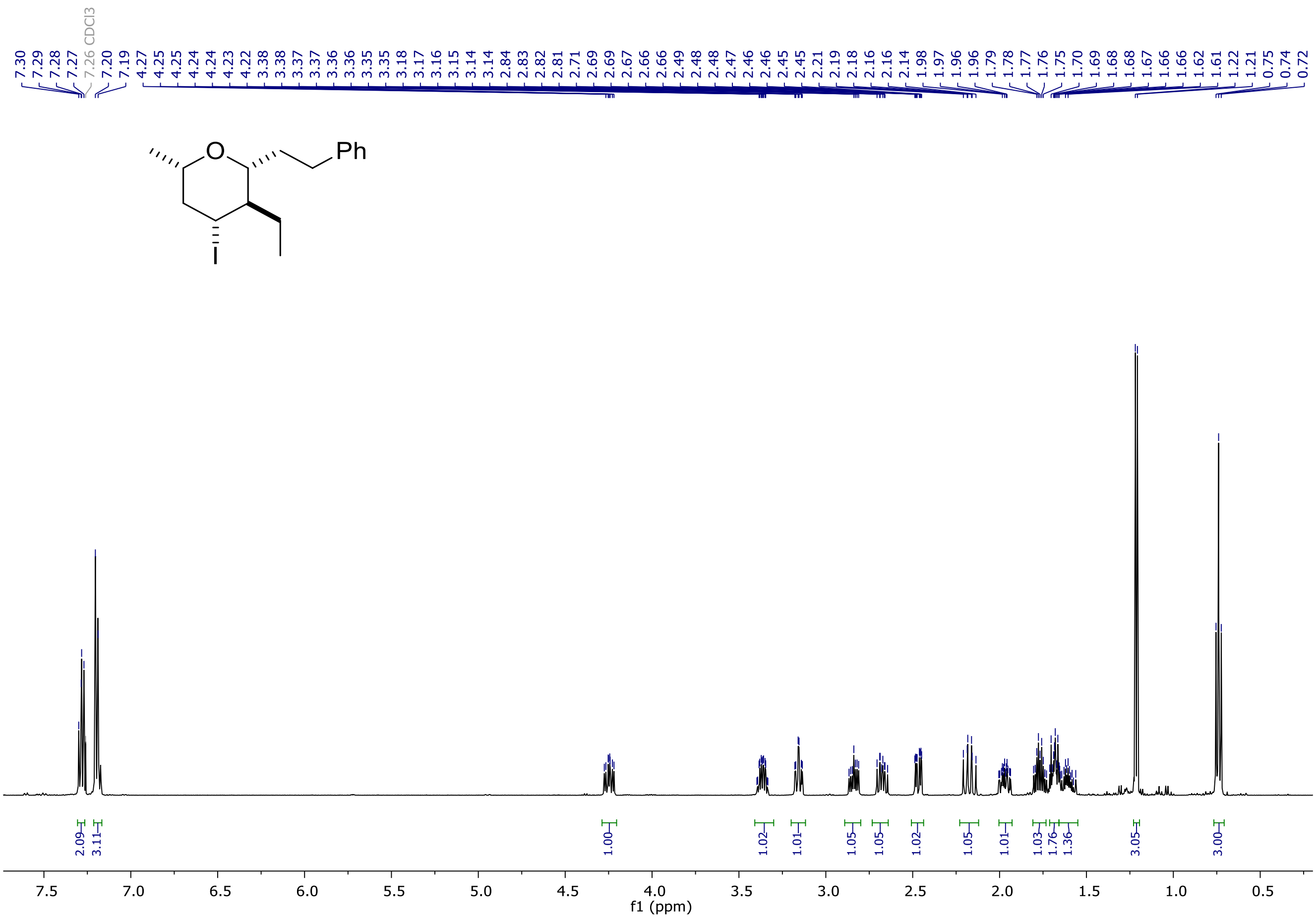

^{13}C NMR (101 MHz, $CDCl_3$)

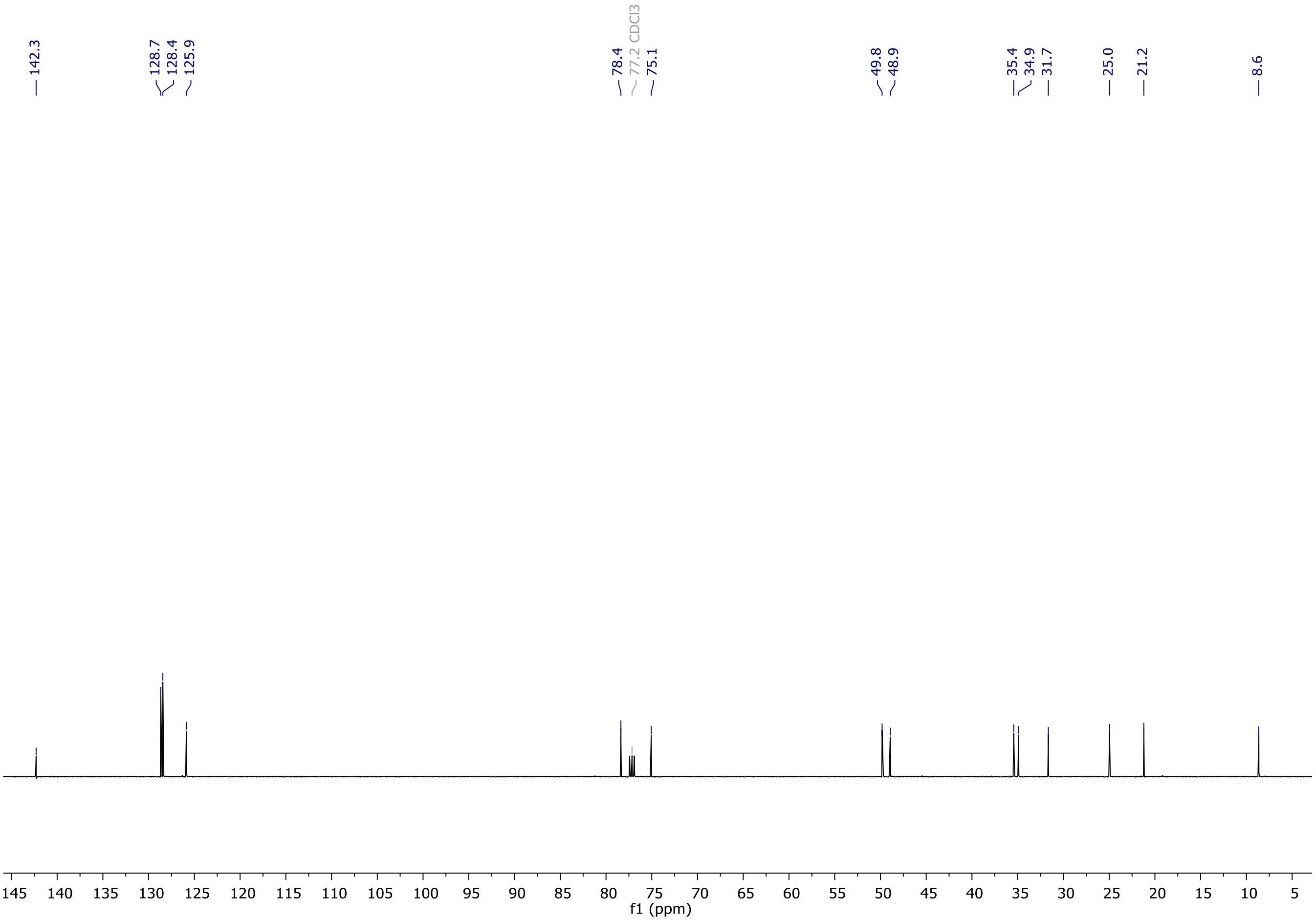

2D-COSY

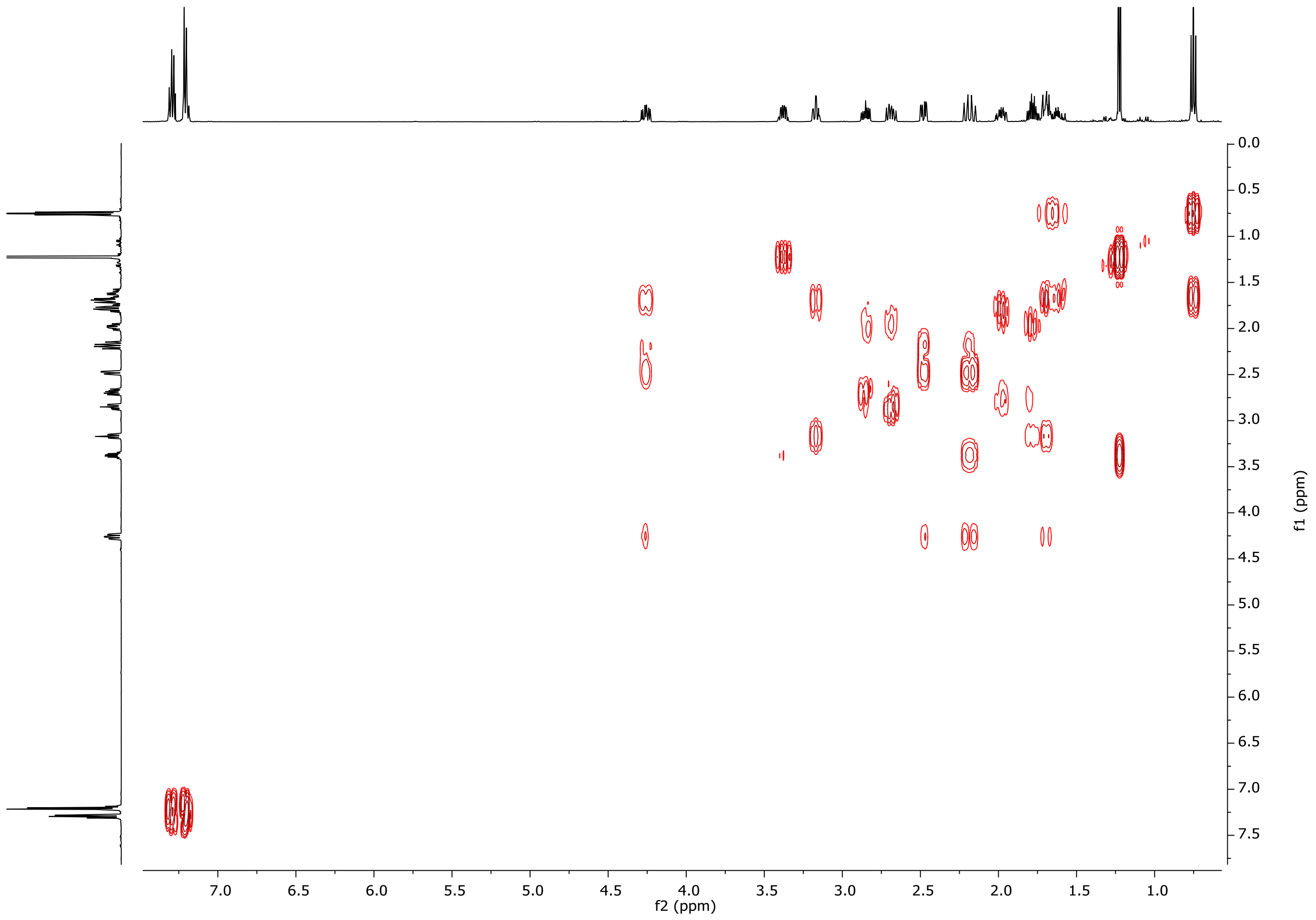

2D-HSQC

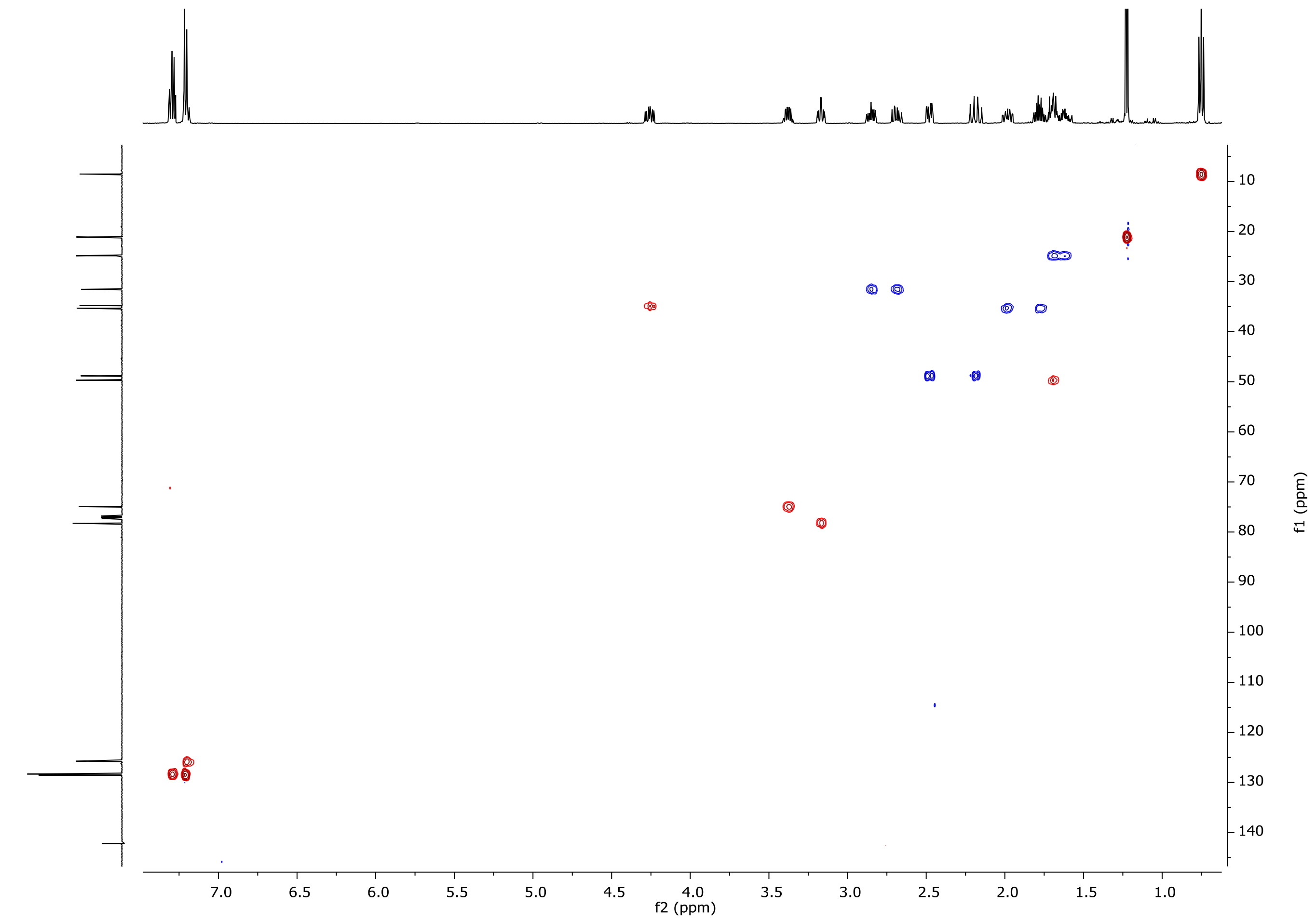

Compound 9b

¹H NMR (500 MHz, $CDCl_3$)

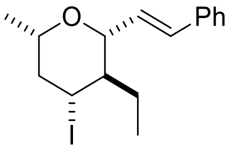

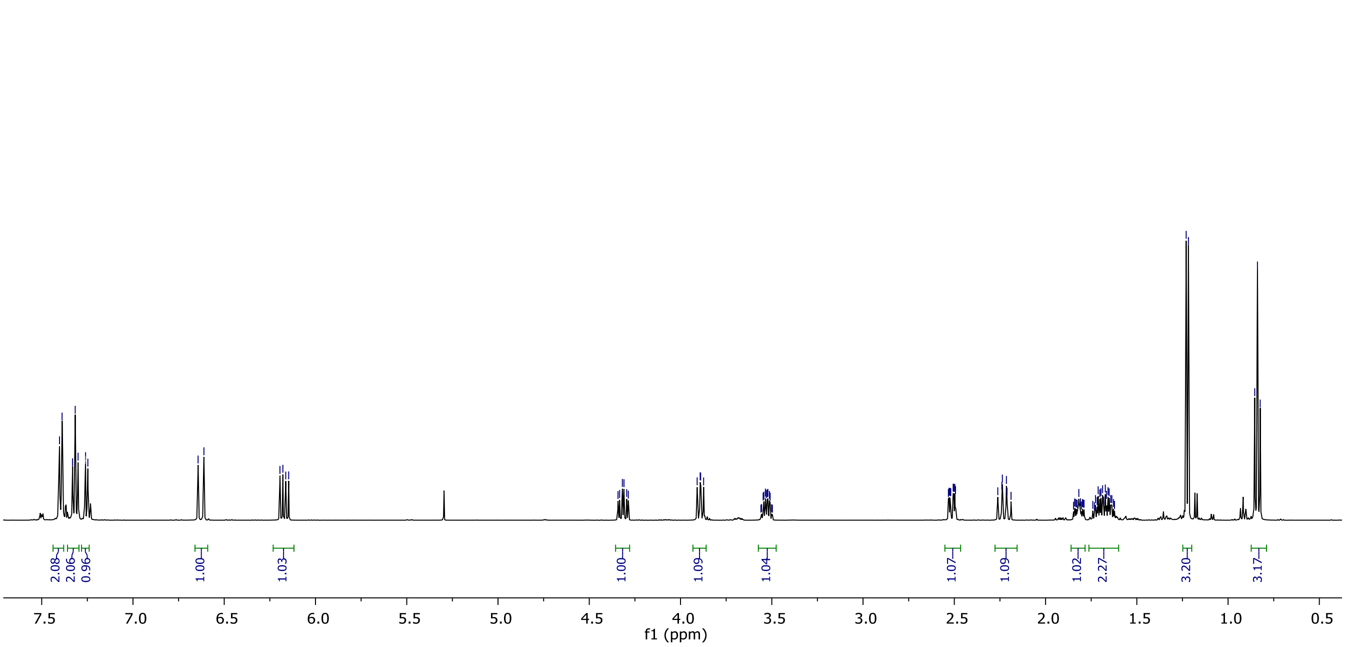

¹³C NMR (101 MHz, $CDCl_3$)

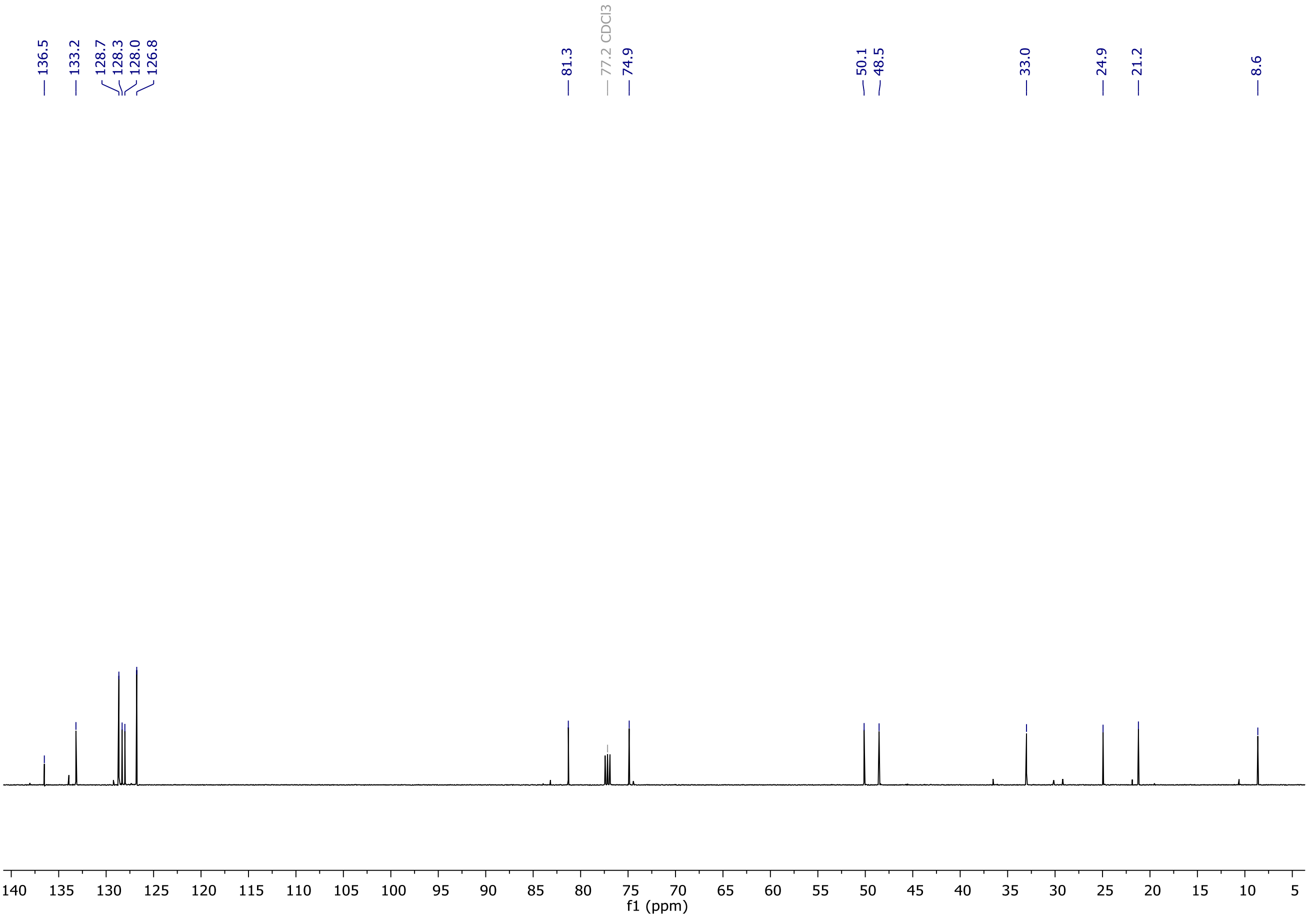

2D-COSY

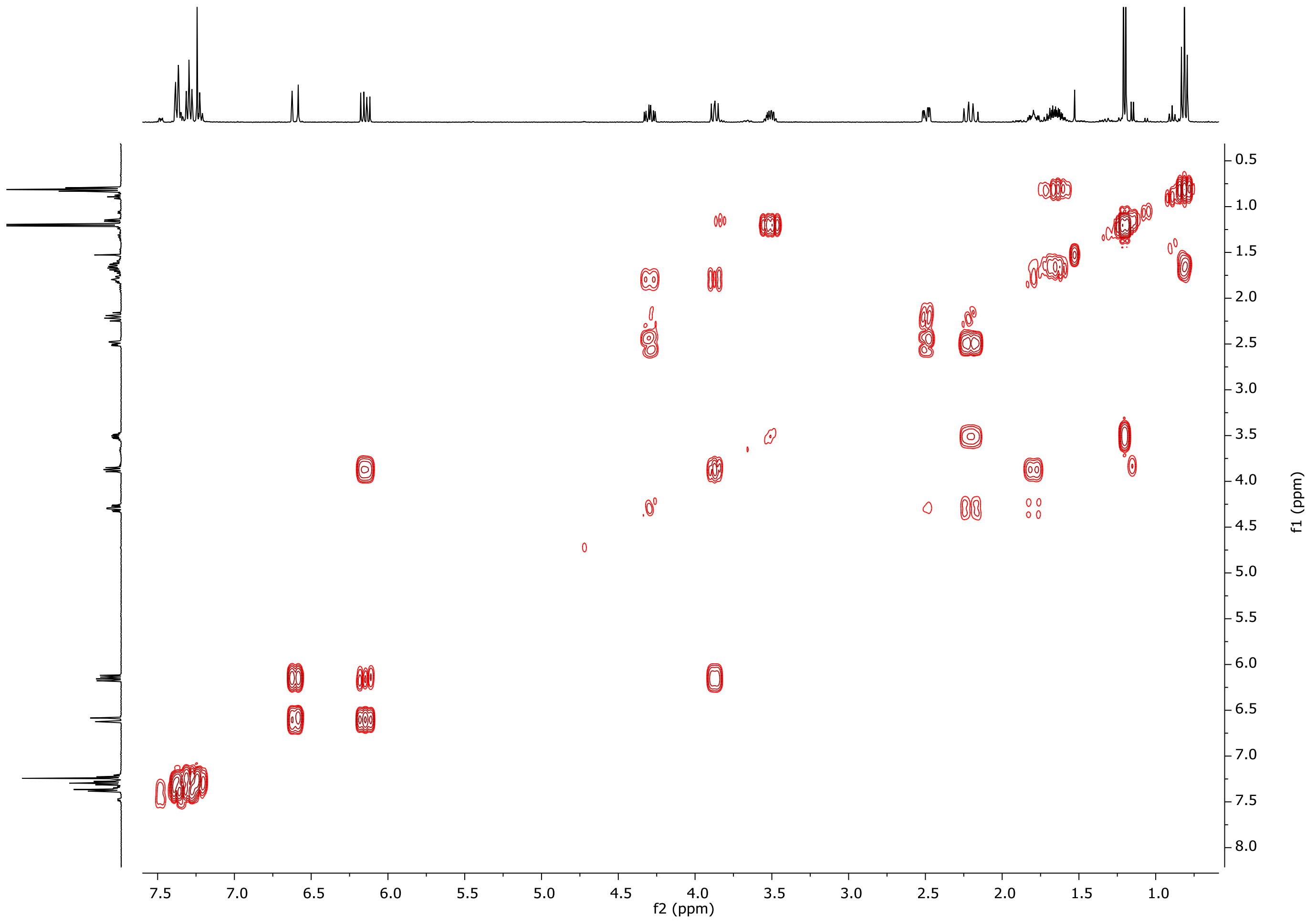

2D-HSQC

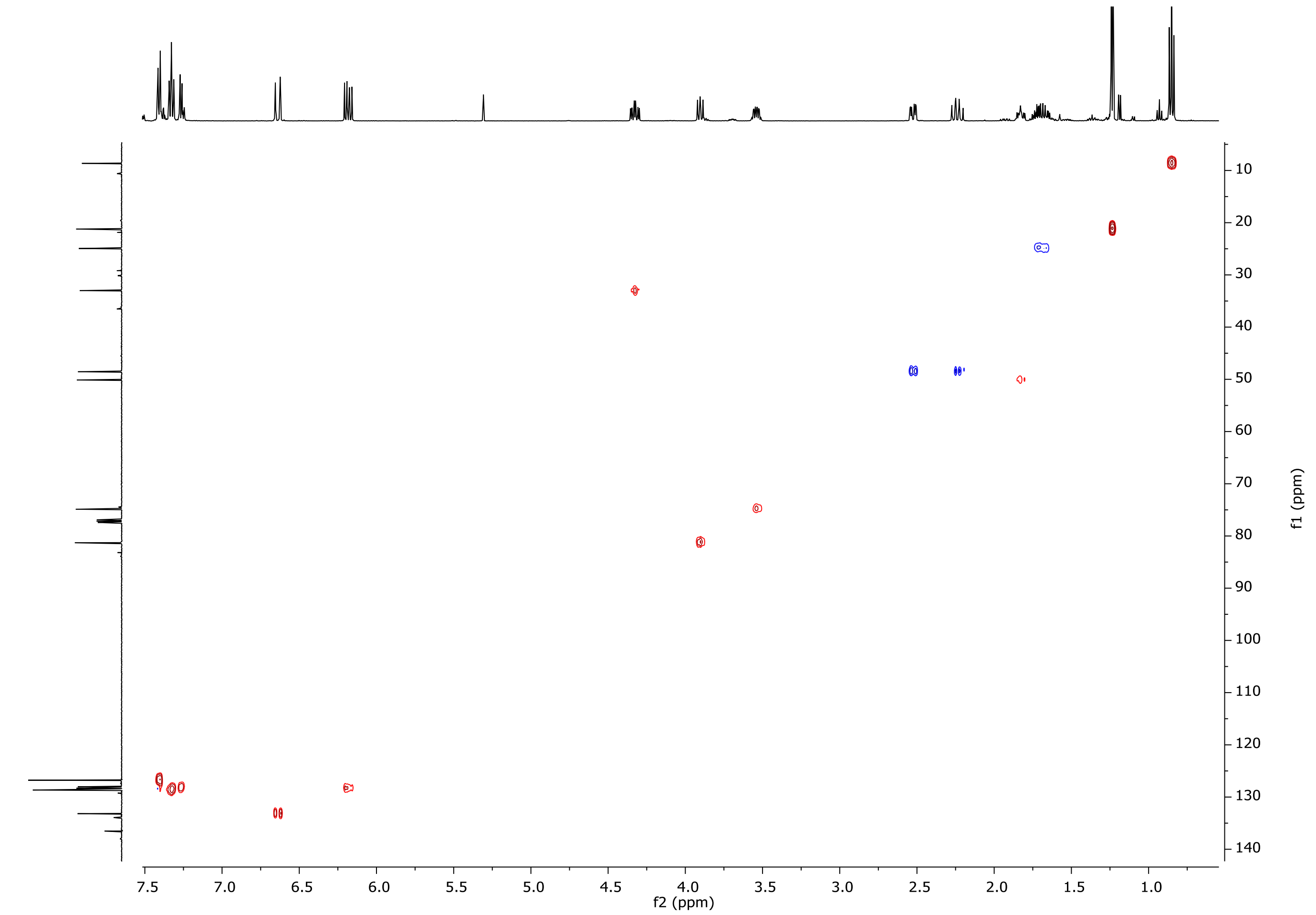

Compound 9c

^{1}H NMR (500 MHz, $CDCl_3$)

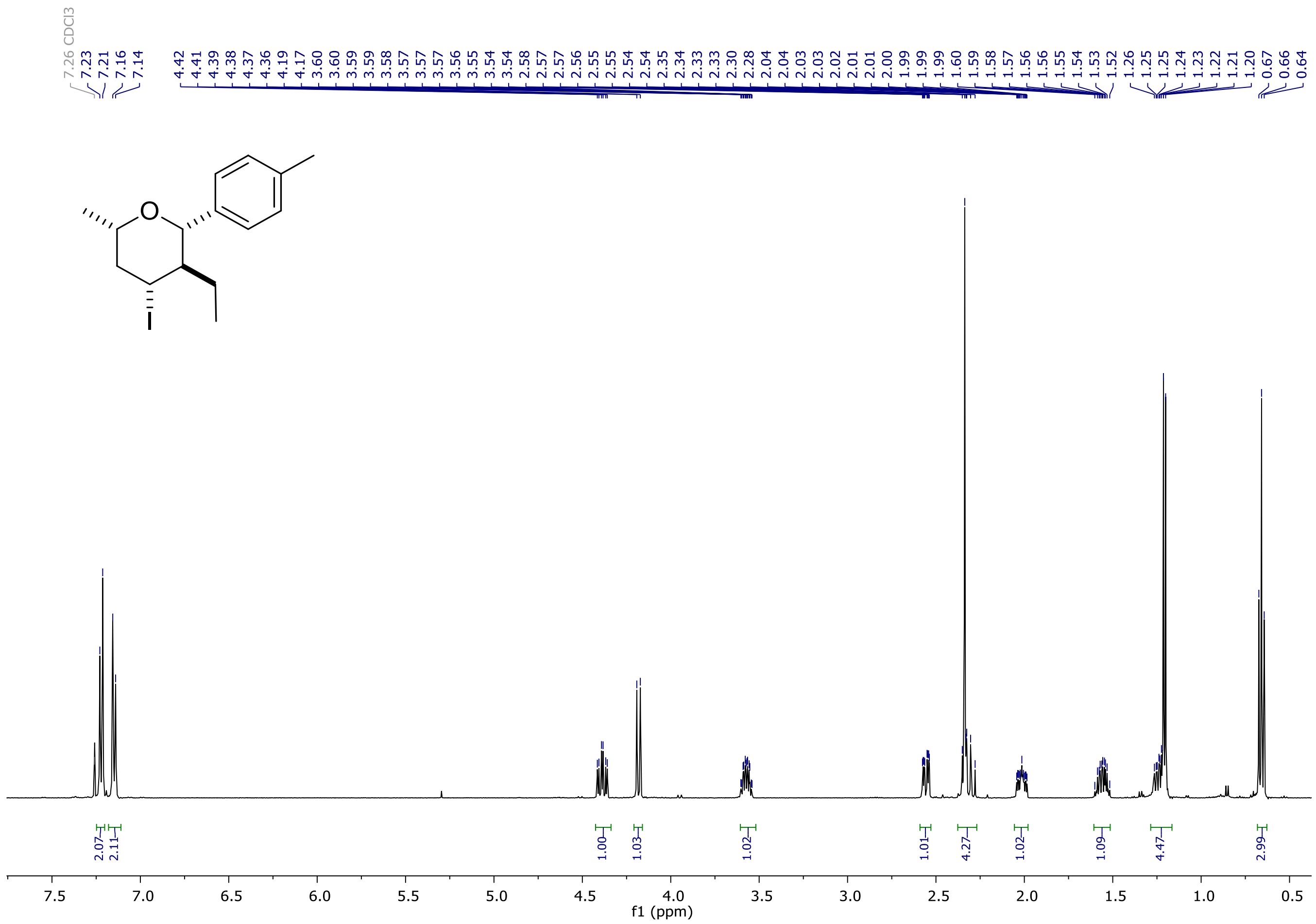

^{13}C NMR (101 MHz, $CDCl_3$)

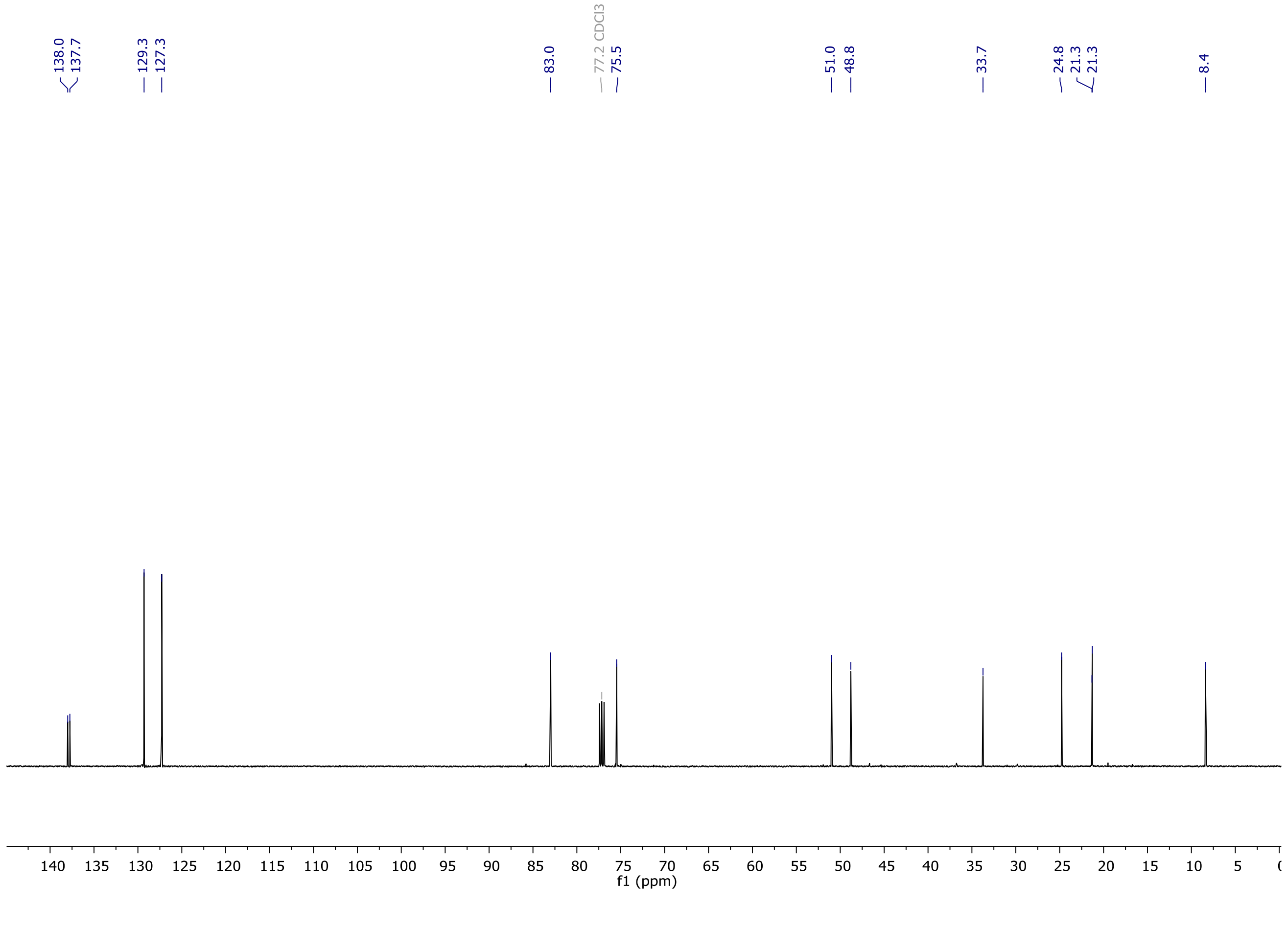

2D-COSY

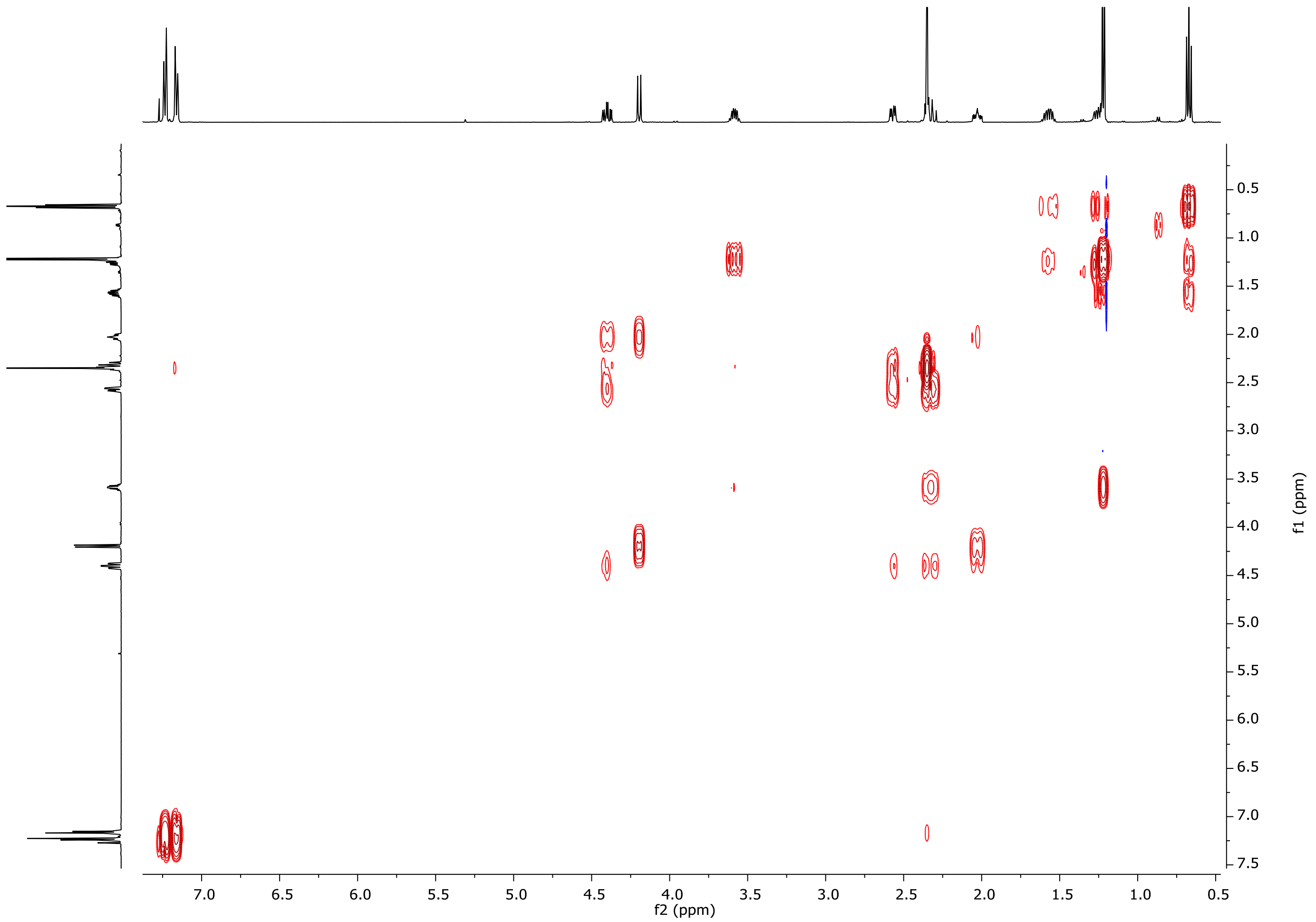

2D-HSQC

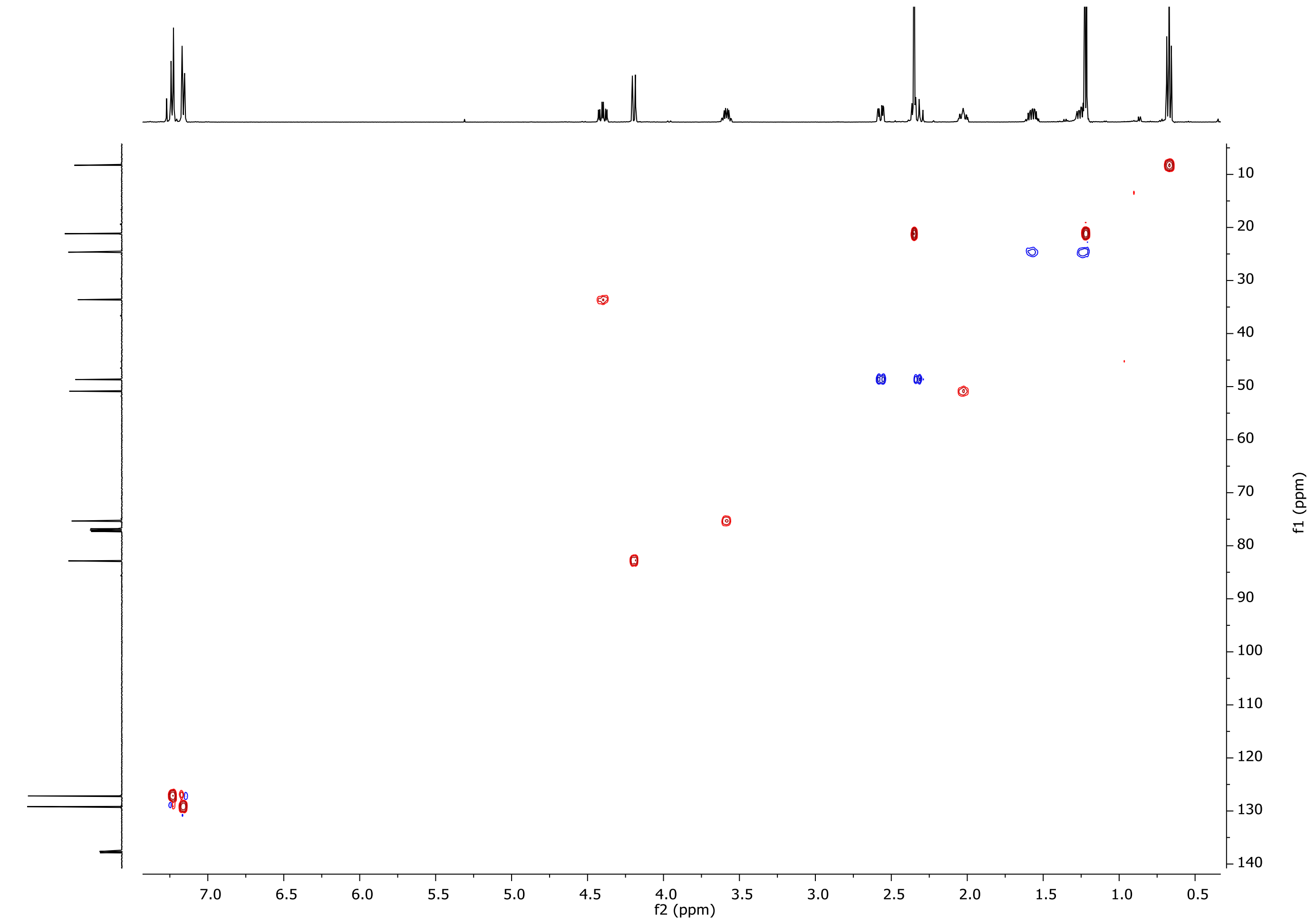

Compound 9d

^{1}H NMR (500 MHz, $CDCl_3$)

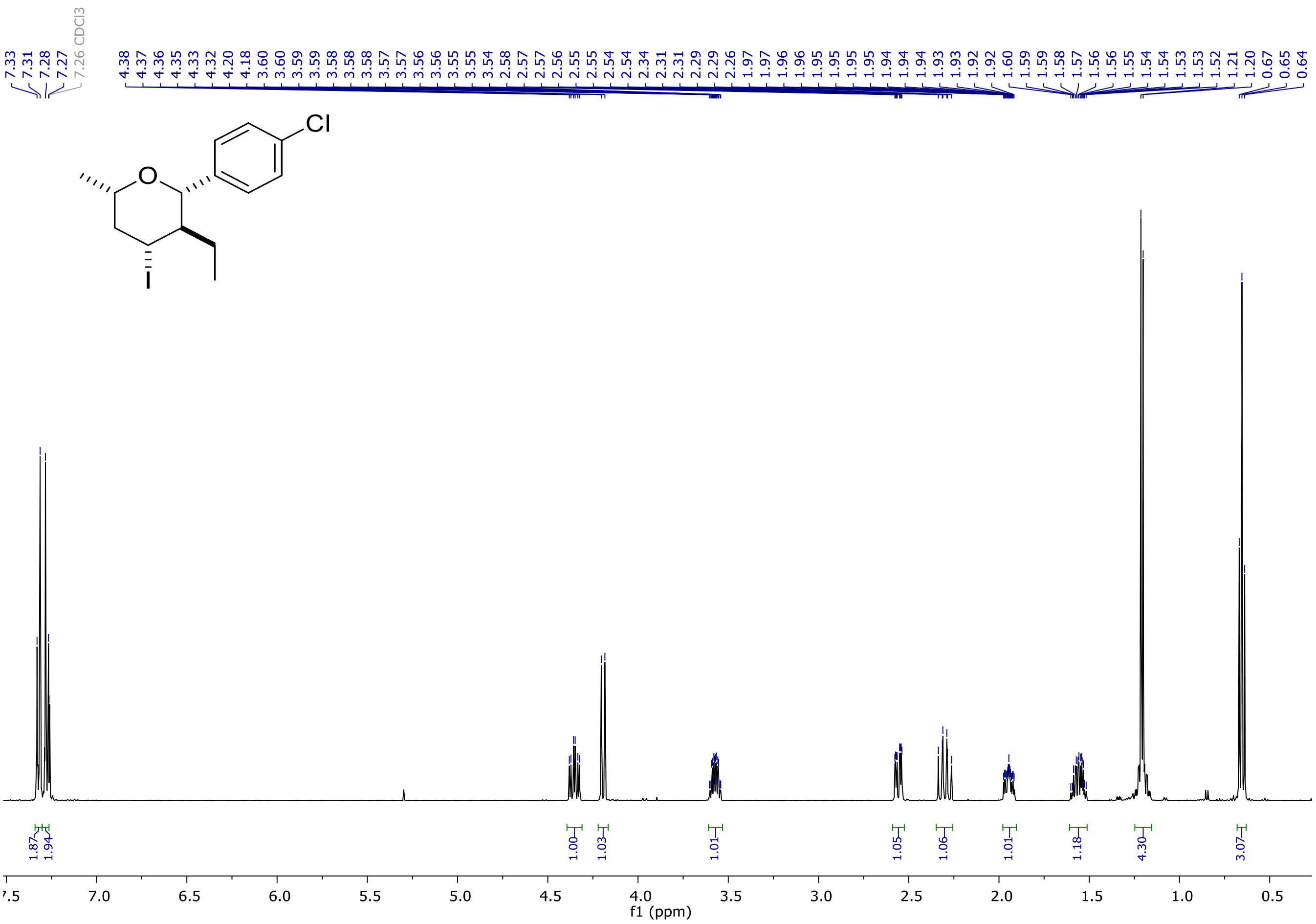

^{13}C NMR (101 MHz, $CDCl_3$)

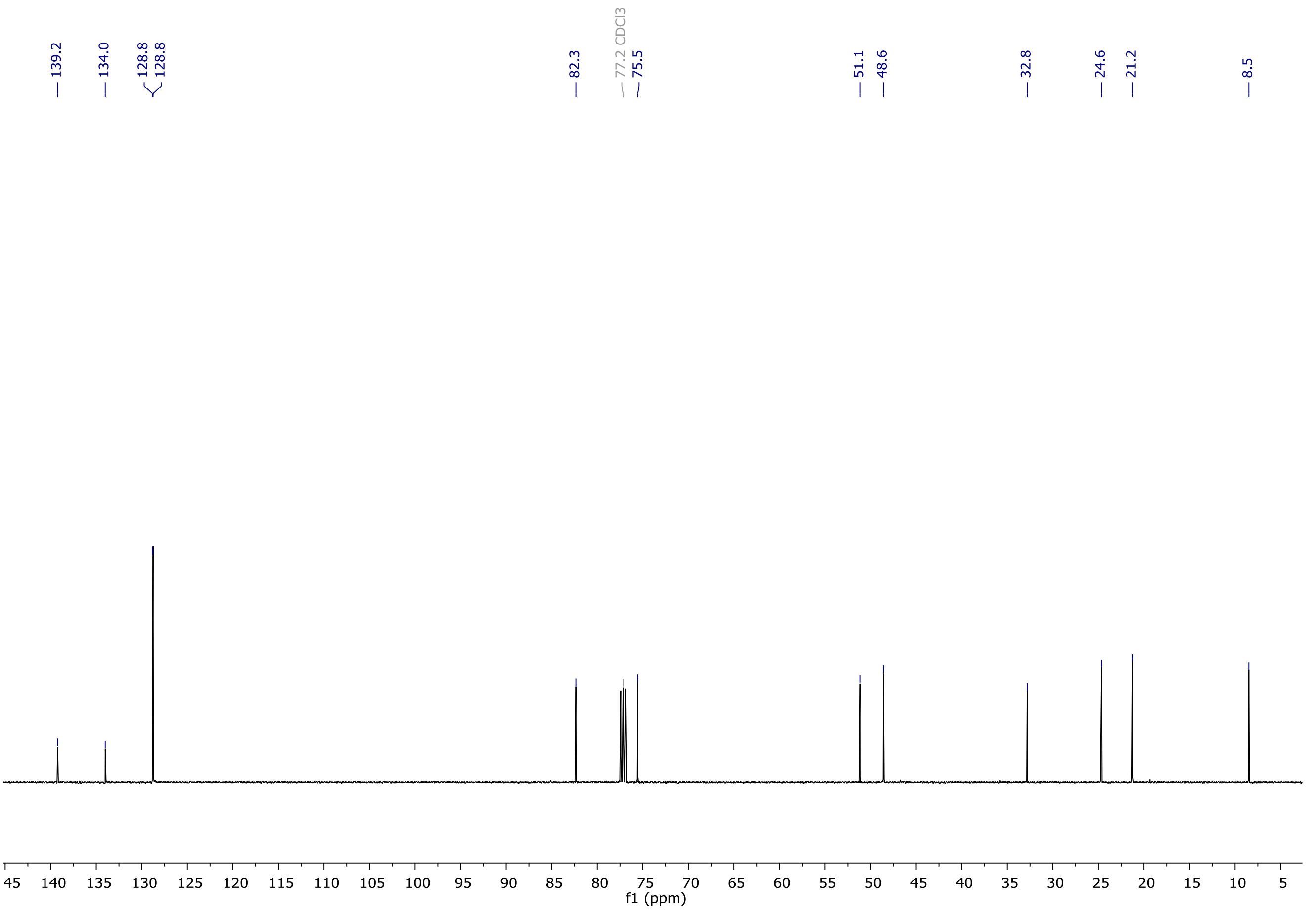

2D-COSY

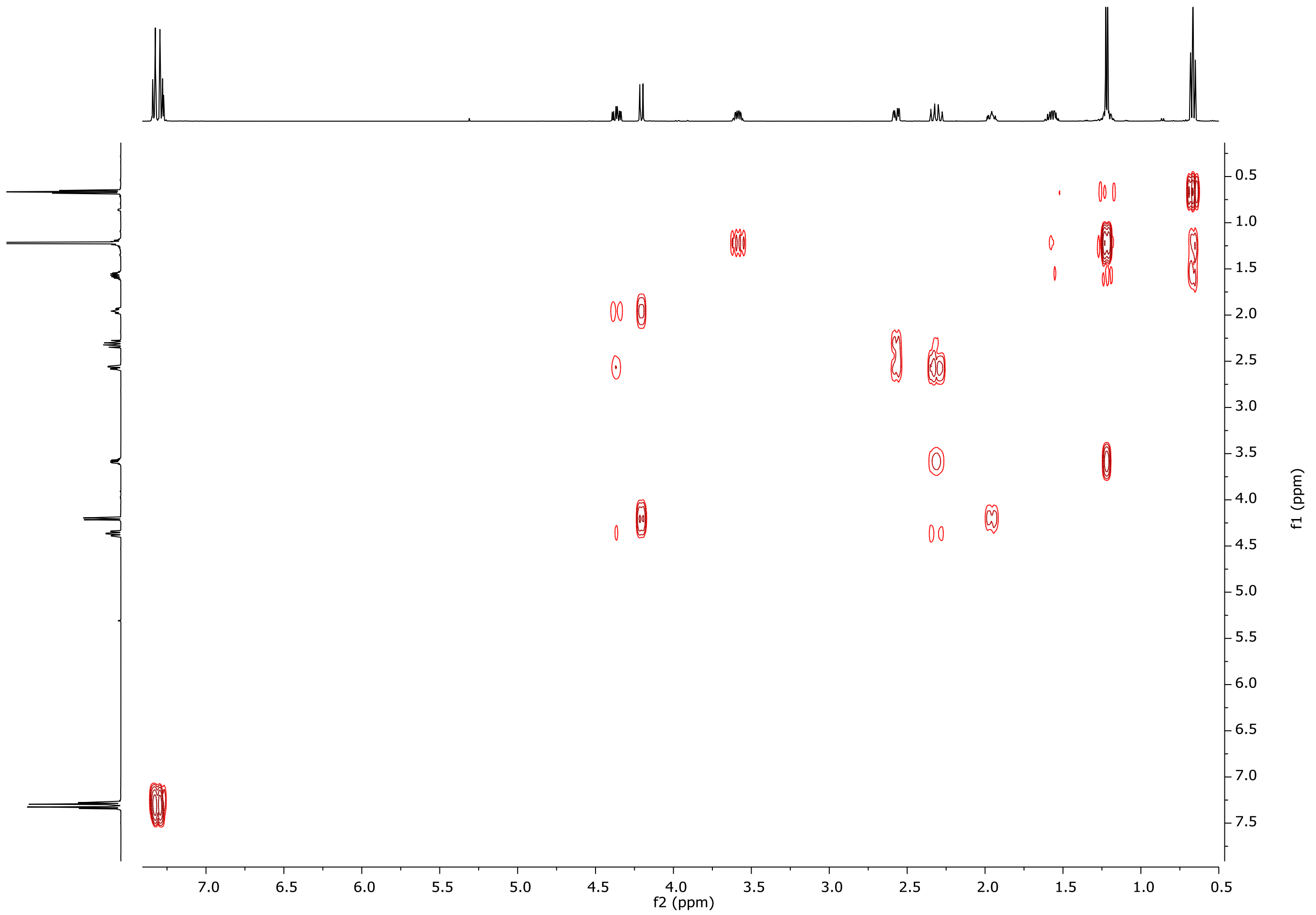

2D-HSQC

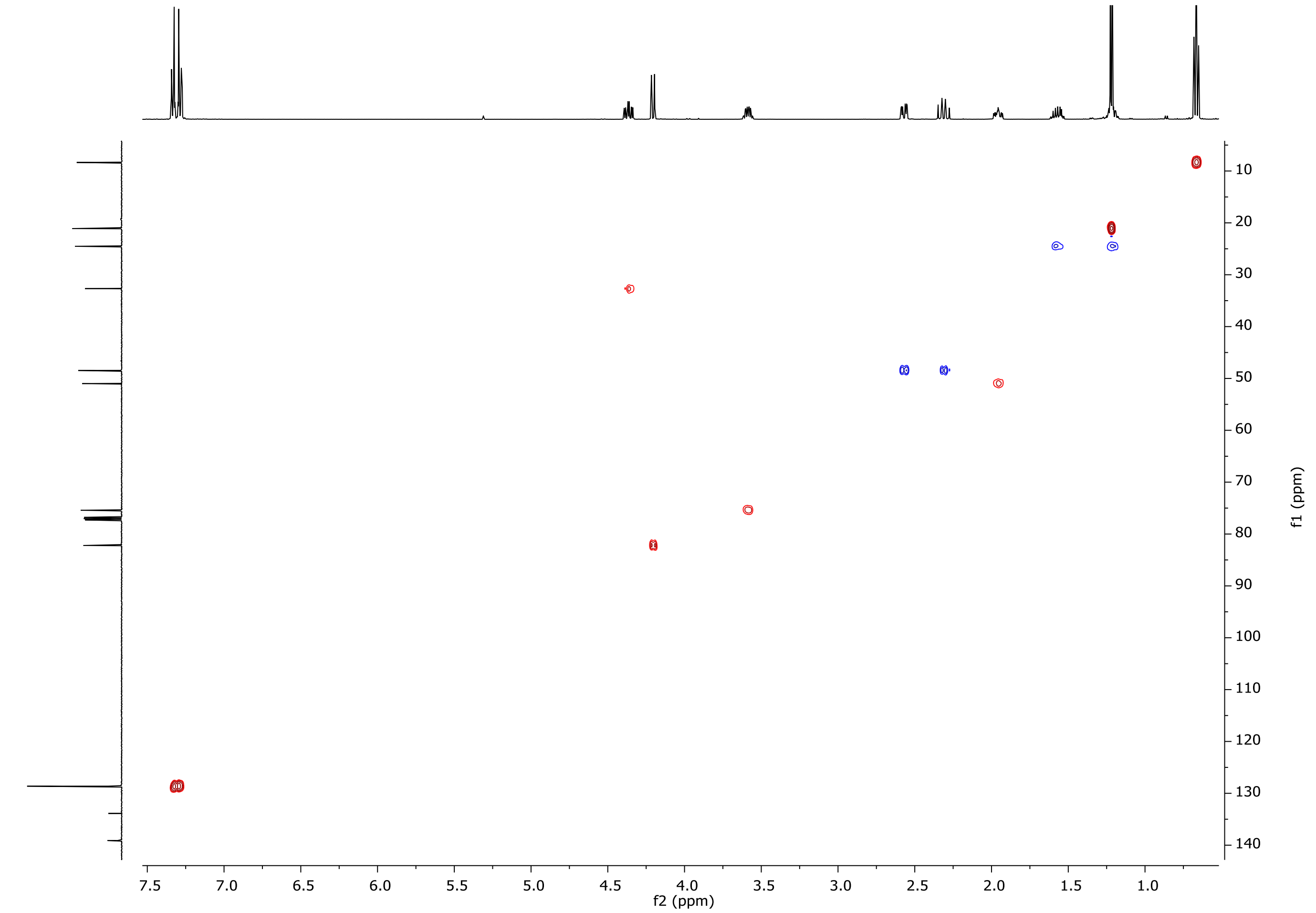

Compound 9e

^{1}**H NMR** (500 MHz, $CDCl_3$)

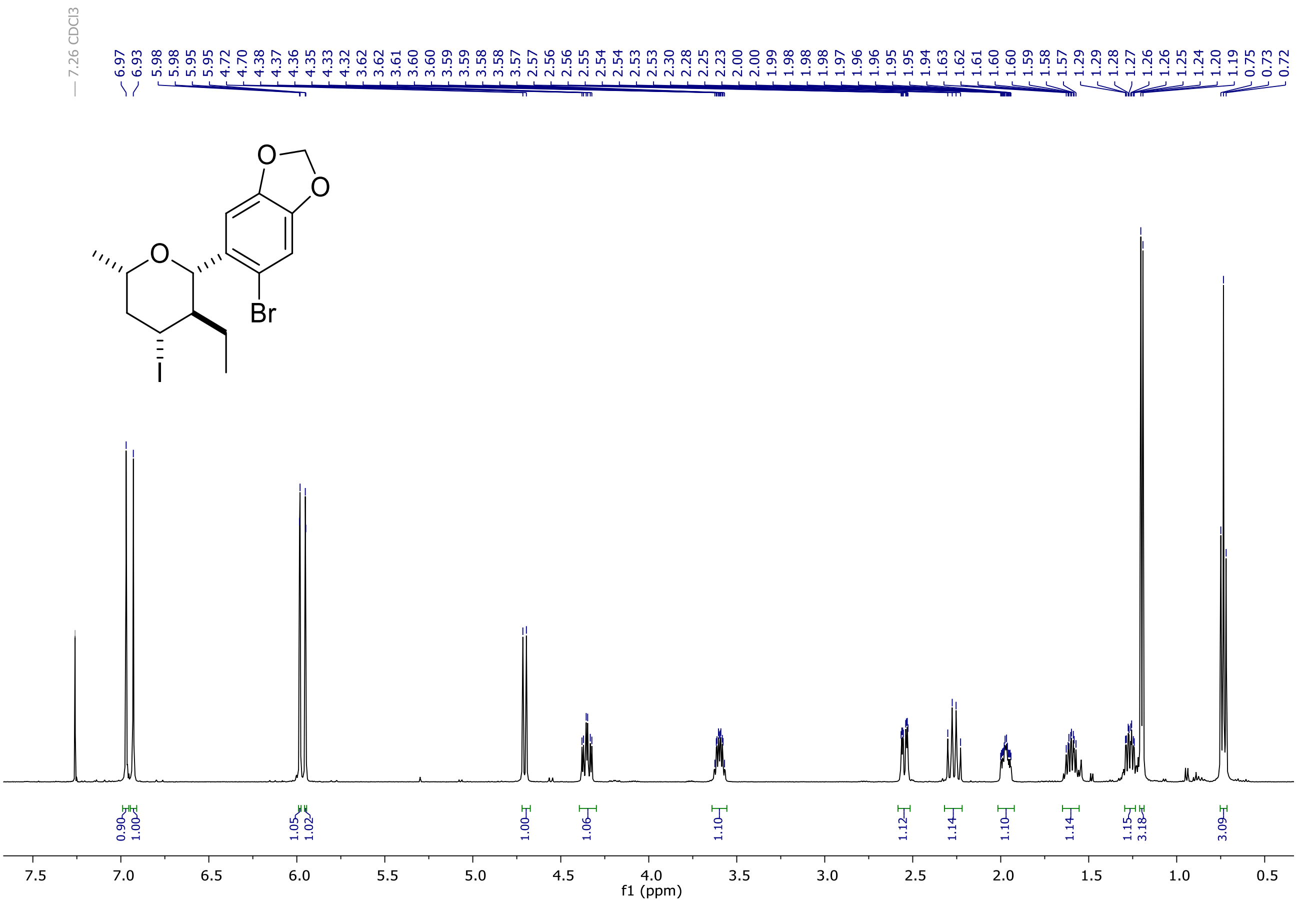

^{13}**C NMR** (101 MHz, $CDCl_3$)

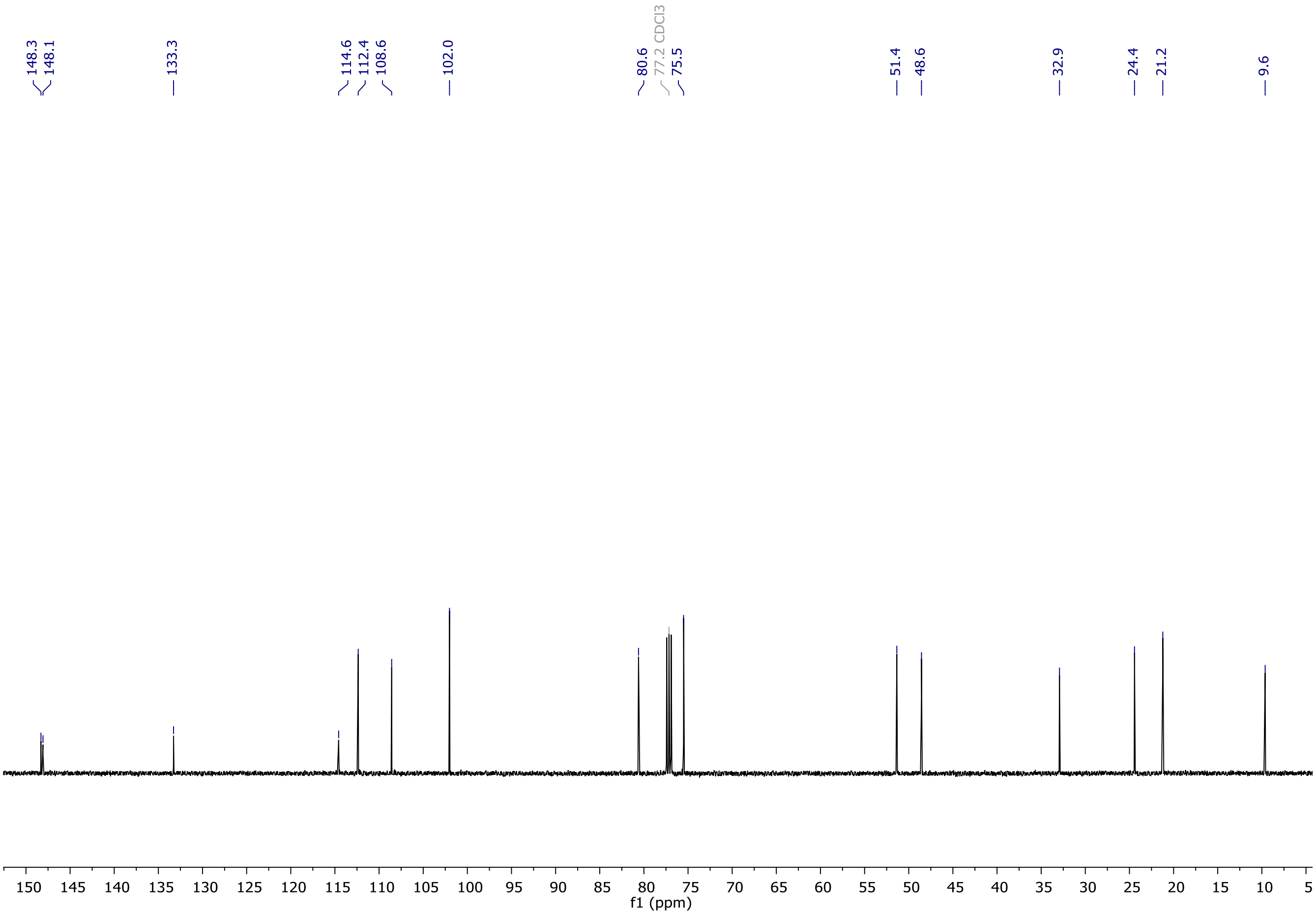

2D-COSY

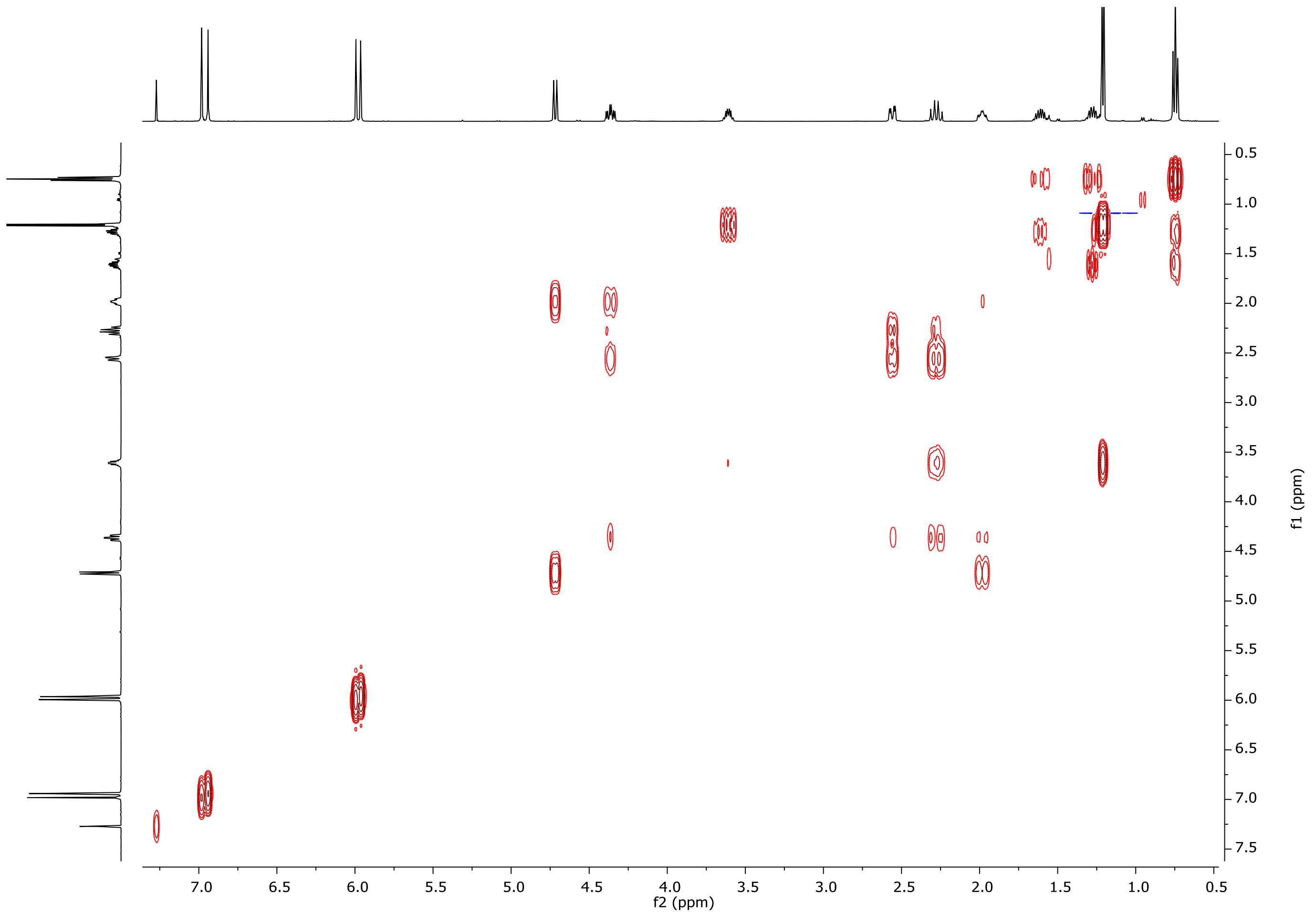

2D-HSQC

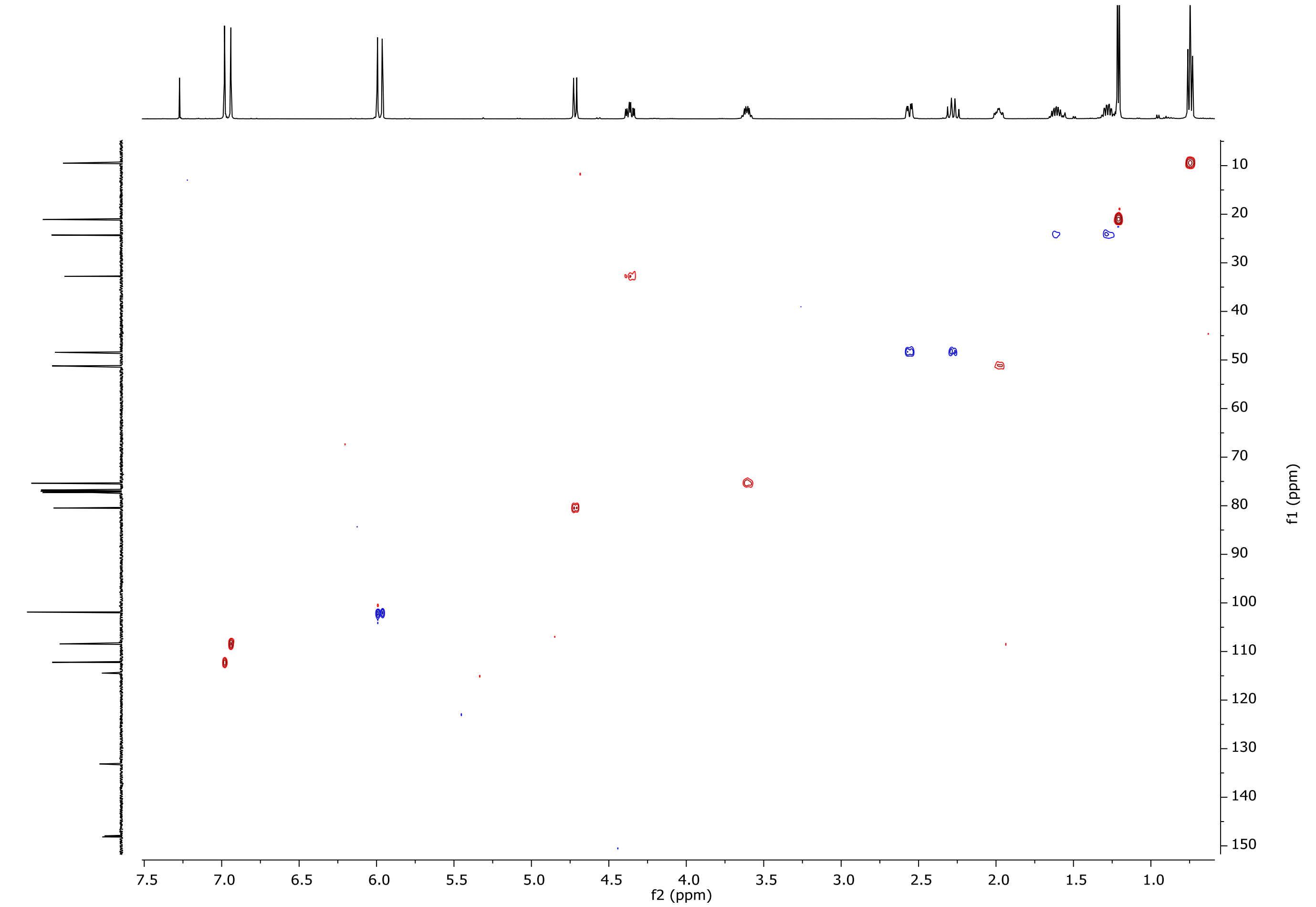

5. REFERENCES

[1] CrysAlisPro-Data Collection and Integration Software, Agilent Technologies UK Ltd, Oxford, UK, 2011.
[2] Dolomanov, O. V; Bourhis, L. J.; Gildea, R. J.; Howard, J. A. K.; Puschmann, H. OLEX2: A Complete Structure Solution, Refinement and Analysis Program. *J. Appl. Crystallogr.* **2009**, *42*, 339–341.
[3] Sheldrick, G. M. Crystal Structure Refinement with SHELXL. *Acta Crystallogr., Sect. C.* **2015**, *71*, 3–8.
[4] Macrae, C. F.; Sovago, I.; Cottrell, S. J.; Galek, P. T. A.; McCabe, P.; Pidcock, E.; Platings, M.; Shields, G. P.; Stevens, J. S.; Towler, M.; Wood, P. A. Mercury 4.0: from visualization to analysis, design and prediction. *J. Appl. Crystallogr.* **2020**, *53*, 226-235.
